# Evaluation of the potential of Pap test fluid and cervical swabs to serve as clinical diagnostic biospecimens for the detection of ovarian cancer by mass spectrometry-based proteomics

**DOI:** 10.1186/s12014-020-09309-3

**Published:** 2021-01-07

**Authors:** Kristin L. M. Boylan, Somaieh Afiuni-Zadeh, Melissa A. Geller, Peter A. Argenta, Timothy J. Griffin, Amy P. N. Skubitz

**Affiliations:** 1 Department of Laboratory Medicine & Pathology, University of Minnesota Medical School, MMC 395, 420 Delaware St. SE, Minneapolis, MN 55455 USA; 2 Department of Obstetrics, Gynecology, & Women’s Health, University of Minnesota Medical School, Minneapolis, MN USA; 3 Department of Biochemistry, Molecular Biology, & Biophysics, University of Minnesota Medical School, Minneapolis, MN USA; 4 Ovarian Cancer Early Detection Program, University of Minnesota Medical School, Minneapolis MN, USA; 5 Present Address: Lunenfeld-Tanenbaum Research Institute, Mount Sinai Health, Toronto, ON M5G 1X5 Canada

**Keywords:** Ovarian cancer detection, Pap test, Mass spectrometry based proteomics, Biomarker

## Abstract

**Background:**

The purpose of this study was to determine whether the residual fixative from a liquid-based Pap test or a swab of the cervix contained proteins that were also found in the primary tumor of a woman with high grade serous ovarian cancer. This study is the first step in determining the feasibility of using the liquid-based Pap test or a cervical swab for the detection of ovarian cancer protein biomarkers.

**Methods:**

Proteins were concentrated by acetone precipitation from the cell-free supernatant of the liquid-based Pap test fixative or eluted from the cervical swab. Protein was also extracted from the patient’s tumor tissue. The protein samples were digested into peptides with trypsin, then the peptides were run on 2D-liquid chromatography mass spectrometry (2D-LCMS). The data was searched against a human protein database for the identification of peptides and proteins in each biospecimen. The proteins that were identified were classified for cellular localization and molecular function by bioinformatics integration.

**Results:**

We identified almost 5000 proteins total in the three matched biospecimens. More than 2000 proteins were expressed in each of the three biospecimens, including several known ovarian cancer biomarkers such as CA125, HE4, and mesothelin. By Scaffold analysis of the protein Gene Ontology categories and functional analysis using PANTHER, the proteins were classified by cellular localization and molecular function, demonstrating that the Pap test fluid and cervical swab proteins are similar to each other, and also to the tumor extract.

**Conclusions:**

Our results suggest that Pap test fixatives and cervical swabs are a rich source of tumor-specific biomarkers for ovarian cancer, which could be developed as a test for ovarian cancer detection.

## Background

Early detection of ovarian cancer increases survival, yet a screening tool that is adequately sensitive and specific for use in the general population is lacking. Barriers to the development of a screening tool include: the low prevalence of ovarian cancer in the general population, the inaccessibility of the ovaries to direct evaluation, non-specificity of known tumor markers (such as CA125) [1], and the absence of known risk factors (such as high-risk genetic mutations) for the majority of patients. In contrast, cervical cancer screening by Pap tests has been routinely performed for over 50 years with a great reduction in the burden of human papilloma virus-related cancers [2].

In the liquid-based Pap test, cells collected from the cervix are placed in an alcohol-based fixative for examination [3]. Notably, ovarian cancer cells have been observed in Pap tests [4–6], suggesting that ovarian cancer peptide biomarkers may also be present. We hypothesized that proteins shed by ovarian cancer cells can be detected during routine Pap tests by mass spectrometry (MS)-based proteomics. The use of biospecimens proximal to the tumor site improves biomarker detection [7]; the proposed strategy takes advantage of the proximity of the cervix to the ovary (i.e. proteins may be secreted or shed from the tumor and flow through the fallopian tube into the uterus and out the cervical opening), and uses already-obtained diagnostic material, which may help with cost-containment and accessibility.

 To demonstrate the feasibility of using Pap tests as a biospecimen for proteomics, we previously examined the proteins present in residual Pap test fixative samples from women with normal cervical cytology by 2D-LCMS and described 153 core proteins in the “Normal Pap test Core Proteome” [8]. The objectives of this study were to identify the proteins present in three different biospecimens from a single patient with high grade serous ovarian cancer: (i) the residual Pap test fixative, (ii) a polyvinyl alcohol (Merocel™) swab of the cervix, and (iii) the primary tumor tissue. The goal was to determine whether the Pap test fluid or the swab could serve as a surrogate biospecimen for the tumor; providing proof of concept that these two biospecimens could be developed for use in the detection of ovarian cancer biomarkers prior to surgery.

## Methods

### Patient clinical information

The patient was a 72 year old, post-menopausal woman diagnosed with late stage (metastatic) high grade serous adenocarcinoma of ovarian or peritoneal origin that did not encompass the cervix. Cytologic interpretation of the SurePath™ liquid Pap test was negative for malignancy. Presurgical serum CA125 was 100 units/ml. Tumor immunohistochemical stains were positive for Cytokeratin-7, CK HMW, WT-1, and estrogen receptor, and negative for p53, p63, CDX-2, CK20, S-100, uroplakin, Calretinin, and progesterone receptor.

### Sample collection and preparation

**Fig. 1:**
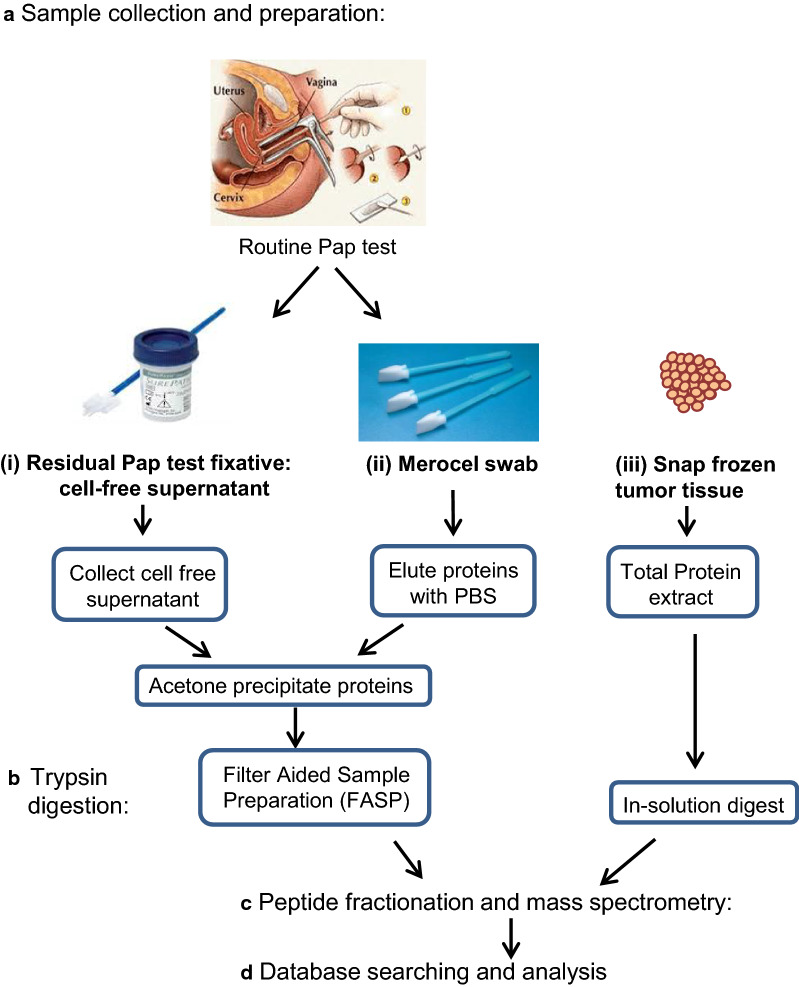
Work flow for the proteomic analysis of ovarian cancer biospecimens. **a** A liquid based Pap test, cervical swab (Merocel™) and snap frozen primary tumor tissue were collected from a patient with high grade serous ovarian cancer and proteins were prepared for mass spectrometry analysis as described in the Methods. **b** Proteins were digested with trypsin using an in-solution digest (tumor tissue extract) or FASP (Pap test fluid and swab proteins). **c** Peptides were HPLC fractionated and analyzed by mass spectrometry. **d** MS/MS data were searched against a forward and reverse sequence human Uniprot database using Sequest. Data was analyzed with Scaffold 4. Proteins were identified with at least 2 peptides at 99% probability and 0.2% decoy False Discovery Rate (FDR)

Following approval from the University of Minnesota Institutional Review Board (protocol 1112M07362), three different biospecimens were collected from the patient and processed for protein isolation and MS analysis (Fig. 1). A Merocel™ cervical swab and SurePath™ liquid-based Pap cytology test were collected prior to surgery in the University of Minnesota Gynecologic Oncology Clinic. Snap frozen primary ovarian cancer tumor tissue was obtained from the University of Minnesota BioNet Tissue Procurement facility.

Fluid from the patient’s cervix was absorbed with a Merocel™ swab by gently pressing it to the surface of the cervical opening for 5 sec. The swab was then placed into a 15 ml conical tube and stored at − 20 °C. Proteins were eluted from the swab by soaking it for 30 min in 300 μl of phosphate-buffered saline, pH 7.4 (PBS). The plastic handle of the swab was then cut off, and the swab plus its washings were added to a Spin-X microtube and centrifuged at 8,845 ×*g* in a microfuge for 20 min at room temperature. The eluted proteins were then used in the studies outlined below.

A standard Pap test was performed using a cervical broom (BD Ref 490,524) which was placed into a SurePath™ liquid-based cytology test (BD Ref 490,527). The Pap test was processed and evaluated by Fairview University cytopathology for abnormal cervical cells. The 2 ml of residual SurePath™ fixative was obtained from the University of Minnesota BioNet Tissue Procurement facility when it was scheduled to be discarded. The SurePath™ vial was vortexed and the SurePath™ fluid was centrifuged for 3 min at 800×*g* to pellet the cells. The cell-free Pap test fluid was then used in the studies outlined below.

The proteins eluted from the swab and the cell-free supernatant from the residual Pap test fixative were concentrated by acetone precipitation as previously described [8]. The protein pellets were resuspended in 10 mM Tris, pH 7.6 containing 0.4% sodium dodecyl sulfate (SDS). Protein concentration was determined using a BCA (Bicinchoninic Acid) assay (ThermoFisher Pierce).

A total protein extract was prepared from snap frozen tumor tissue by pressure cycling in a Barocycler. Frozen tumor tissue was ground into a powder on dry ice with a mortar and pestle, and reconstituted in extraction buffer [7 M urea, 2 M thiourea, 0.4 M triethylammonium bicarbonate (TEAB) pH 8.5, 20% acetonitrile and 4 mM tris(2-carboxyethyl)phosphine (TCEP)] at a ratio of 10 μl of extraction buffer per milligram of tissue. The sample was vortexed, then sonicated at 30% amplitude for 7 sec with a Branson Digital Sonifier 250 (Branson Ultrasonics, Danbury, CT). The sample was transferred to a Pressure Cycling Technology tube with a 150 μl cap for the Barocycler NEP2320 (Pressure Biosciences, Inc., South Easton, MA) and cycled between 35 kpsi for 20 sec and 0 kpsi for 10 sec for 60 cycles at 37 °C. The PCT tube was uncapped and 200 mM methyl methanethiosulfonate (MMTS) was added to a final concentration of 8 mM MMTS, recapped, inverted several times, and incubated 15 min at room temperature. The sample was transferred to a new 1.5 ml microfuge Eppendorf Protein LoBind tube and centrifuged at 13,000×*g* to remove insoluble material. Protein concentration was determination by the Bradford assay.

### Trypsin digestion, peptide fractionation and mass spectrometry

Proteins from concentrated Pap test and swab samples were trypsin digested and prepared for MS by the Filter Aided Sample Preparation (FASP) method using Nanosep Omega centrifugal devices with a 10K MW cut off (Pall Corp., Port Washington, NY) as previously described [8]. Concentrated Pap test and swab samples (~ 50 µg protein) were solubilized in 10 mM Tris, pH 7.6, 0.4% SDS, and reduced by the addition of 10 mM TCEP at room temperature, alkylated with 50 mM iodoacetamide (Sigma-Aldrich, St. Louis, MO) and digested overnight at 37 °C with sequencing grade trypsin (Promega, Madison, WI) using an enzyme:protein ratio of 1:100. Peptides were desalted with C18 stage tips (Thermo Scientific, West Palm Beach, FL) and dried under vacuum.

Tumor tissue proteins were digested “in solution” as follows: 200 µg of the tumor tissue extract was diluted five-fold with ultra-pure water. Trypsin was added in a 1:40 ratio of trypsin to total protein. The sample was incubated for 16 h at 37 °C. After incubation, the sample was frozen at − 80 °C for 30 min and dried in a vacuum centrifuge. The sample was then cleaned with a 4 ml Extract Clean™ C18 SPE cartridge from Grace–Davidson (Deerfield, IL) and the eluate was vacuum dried.

### Peptide liquid chromatography fractionation and mass spectrometry

Peptides were fractionated offline by high pH C18 reversed-phase (RP) chromatography followed by fraction concatenation for 2D proteomic analysis. Briefly, samples were resuspended in Buffer A (20 mM ammonium formate pH, 10 in 98:2 water:acetonitrile) and fractionated using a Shimadzu Promenance HPLC (Shimadzu, Columbia, MD) with a Hot Sleeve-25L Column Heater (Analytical Sales & Products, Inc., Pompton Plains, NJ) and a Security Guard pre-column housing a Gemini NX C18 cartridge (Phenomenex, Torrance, CA) attached to a C18 XBridge column, 150 mm × 2.1 mm internal diameter, 5 um particle size (Waters Corporation, Milford, MA). The flow rate was 200 µl/min with a gradient of 2–5% buffer B (20 mM ammonium formate, pH 10 in 10:90 water:acetonitrile) over 0.5 min, 5–35% buffer B over 57 min, and 35–60% buffer B over 8 min. Fractions were collected at 2 min intervals and UV absorbance was monitored at 215 nm and 280 nm, followed by fraction concatenation for analysis by 2D-LCMS [9]. Concatenated samples were dried in vacuo, resuspended in load solvent (98:1.99:0.01, water:acetonitrile:formic acid) and run on the LTQ Orbitrap Velos (Thermo Scientific) as previously described [10] except lock mass was not invoked.

### Database searching and data analysis

MS/MS data was searched against the human Uniprot [11] canonical and isoform database containing forward and reverse sequences and common contaminants (thegpm.org/crap/index) (Uniprot database version 20161213, containing 92,719 entries total), using Sequest (XCorr Only) version IseNode in Proteome Discoverer 2.2.0.388 (Thermo Scientific), with the following settings: Digestion enzyme, trypsin with one missed cleavage allowed; fragment ion mass tolerance of 0.1 Da; precursor ion tolerance of 50.0 ppm; carbamidomethyl of cysteine as a fixed modification. Variable modifications were pyroglutamic acid of glutamine, deamidation of asparagine, oxidation of methionine, and acetylation of the protein N-terminus.

Scaffold version 4.8.2 (Proteome Software Inc., Portland, OR) was used to validate MS/MS based peptide and protein identifications as previously described [8]. Peptide identifications were accepted if they could be established at greater than 95.0% probability by the Scaffold Local FDR algorithm. Protein identifications were accepted if they could be established at greater than 99.0% probability and contained at least 2 identified peptides and 0.2% decoy False Discovery Rate (FDR). Proteins identified in the decoy or contaminant database were filtered prior to analysis. Raw spectral counts were used as an estimate of the amount of each protein within the samples. The proteins identified by MS were classified by cellular localization using Gene Ontology (GO) annotation [12, 13] in Scaffold. Bioinformatic analysis of protein molecular function was done using PANTHER [14]. Briefly, gene lists for each sample were curated from the Scaffold samples report (proteins identified at 98% probability) and loaded into PANTHER (version 15) and mapped to Gene IDs. Functional classification of the gene lists for each biospecimen were examined using PANTHER Protein Class ontologies, with ~ 63% of the mapped Gene IDs yielding functional classification hits.

## Results

### Comparison of the proteins identified in the three biospecimens

**Fig. 2:**
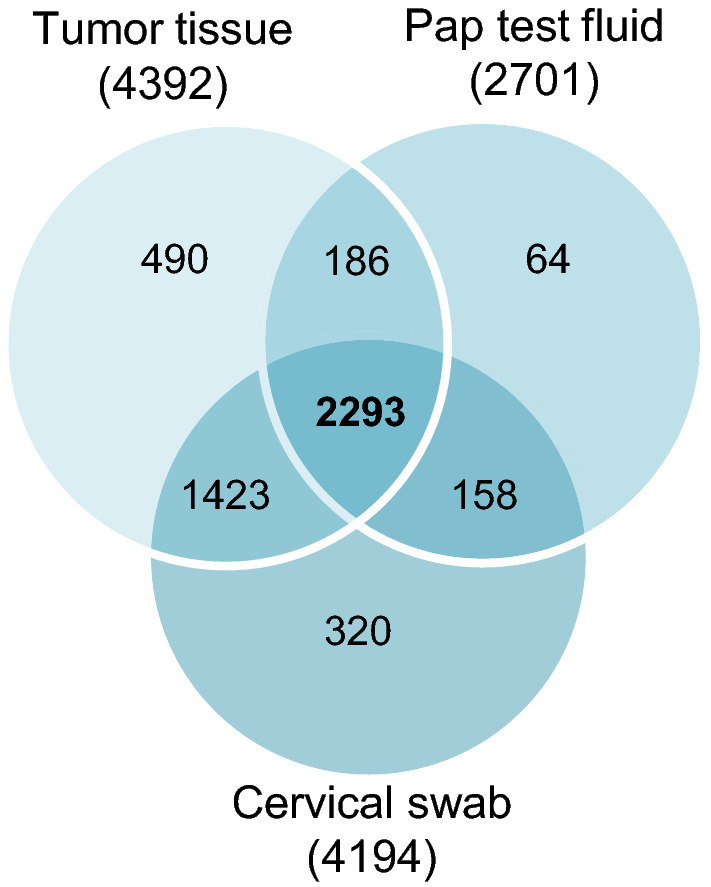
Identification of proteins in biospecimens from an ovarian cancer patient. The proteins identified by mass spectrometry in the tumor tissue extract, Pap test fluid, and cervical swab from an ovarian cancer patient were compared. The Venn diagram shows the intersection of proteins identified in each biospecimen; 2293 proteins were found in all three biospecimens from a total of 4934 proteins identified. The proteins identified in each sample are listed in Additional files 1 and 2

We compared the proteins identified by 2D-LC MS/MS from three biospecimens from a patient with high grade serous ovarian cancer: primary tumor tissue, a cervical swab, and the residual, cell-free fixative from a liquid-based SurePath™ Pap test. A total of almost 5000 proteins were identified in these three biospecimens. The tumor tissue extract yielded the most identified proteins (4392), while 4194 proteins were identified in the cervical swab. Fewer proteins were identified in the residual Pap test fluid (2701 proteins). The same 2293 proteins were identified in all three samples (Fig. 2). A complete list of the proteins identified in each biospecimen can be found in Additional file 1. Details of the protein identification can be found in Additional file 2, with the protein names listed alphabetically along with their corresponding accession number, molecular weight, identification probability, peptide count, spectral count, percentage of total spectra, and percentage sequence coverage.

**Fig. 3:**
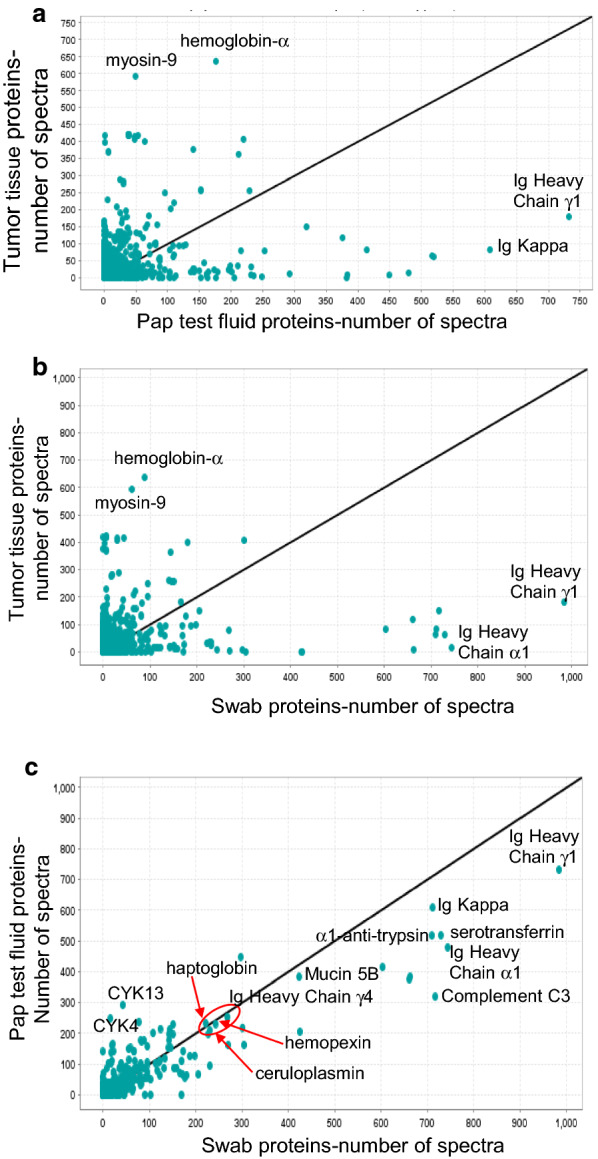
The total number of spectra for each protein was compared between biospecimens. Scatter plots of the total number of spectra identified for the proteins identified in all three biospecimens were created in Scaffold. Due to the large number of spectra from albumin identified in the Pap test fluid and cervical swab, this protein was omitted from the graphs. Comparison of the spectral counts for the proteins identified in: **a** tumor tissue extract and Pap test fluid, **b** tumor tissue extract and cervical swab, and **c** Pap test fluid and cervical swab. The Pap test fluid and swab samples were more similar to each other than to the tumor tissue extract. Red circle indicates serum proteins with similar spectral counts in the Pap test fluid and cervical swab. CYK4, cytokeratin-4; CYK13, cytokeratin-13

To explore the similarity between the biospecimens, the number of spectra assigned to each protein was compared using spectral counting as an estimate of the protein abundance [15]. Scatter plots of the total number of spectra identified for the 2293 proteins found in all three biospecimens are shown in Fig. 3. Comparing the tumor extract to both the Pap test fluid (Fig. 3a) and swab (Fig. 3b), the proteins with the highest number of spectra in the tumor extract were hemoglobin-alpha and myosin-9, while the proteins with the most spectra in the Pap test fluid and swab (after albumin, which was omitted from the analysis for scale) were immunoglobulins. The Pap test fluid and the cervical swab (Fig. 3c) were more similar to each other than to the tumor extract, with the most spectra assigned to immunoglobulin proteins, and also alpha-1-antitrypsin, serotransferrin and complement C3. The protein mucin 5B, a component of cervical mucus, had ~ 400 spectra assigned in both the Pap test and swab samples. An additional group of proteins, with between 200 and 300 spectra, were similarly expressed in the Pap test fluid and swab (Fig. 3c, circled). These proteins (haptoglobin, ceruloplasmin, and hemopexin) are components of serum, suggesting that the large number of serum proteins in both the Pap test fluid and swab underlies the similarity of these samples relative to the tumor sample. One difference we observed between the Pap test and swab samples was the relatively high expression of cytokeratins (CYK) 4 and 13 in the Pap test fluid compared to the swab (248 vs. 16 spectra for CYK4 and 292 vs. 43 spectra for CYK13).

### Cellular localization of the proteins identified in the three biospecimens

**Fig. 4:**
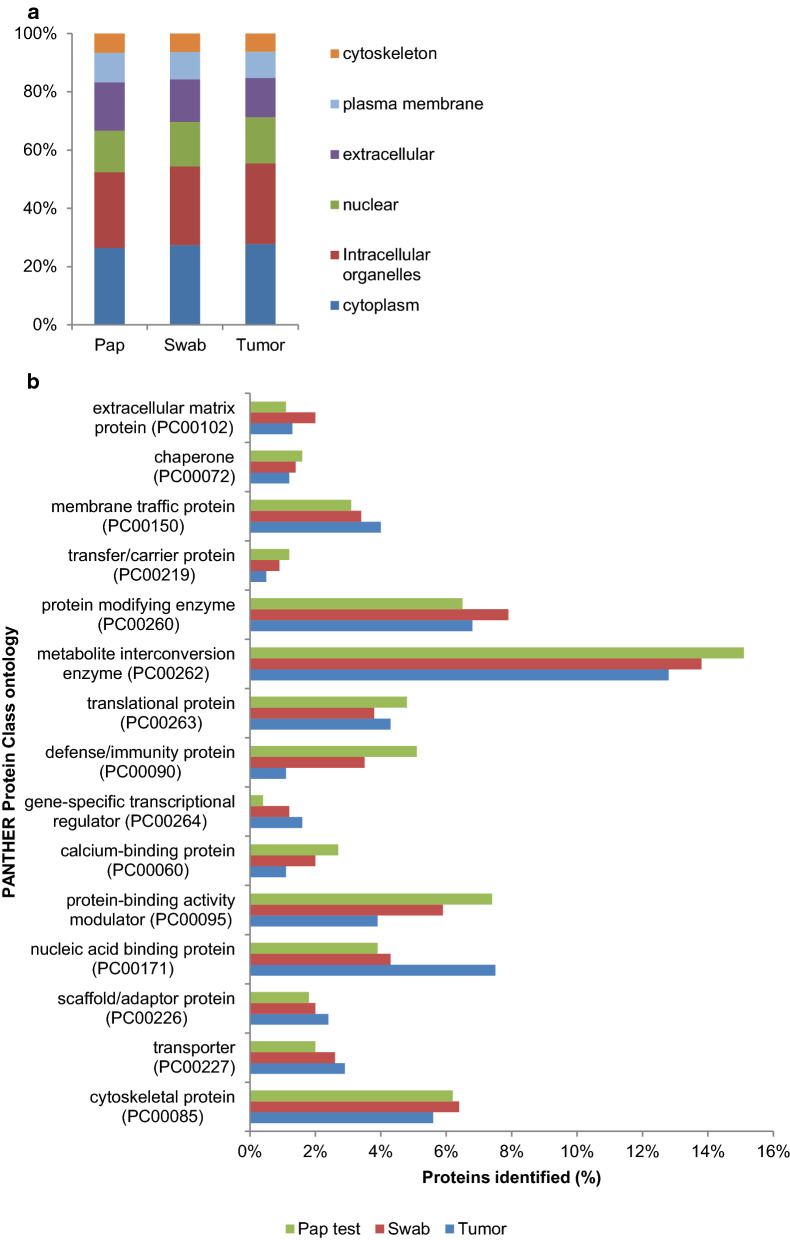
Cellular localization and molecular function of the proteins identified in the three biospecimens. **a** Stacked bar chart of the cellular localization of the proteins identified in the biospecimens from the ovarian cancer patient based on their GO annotation. The cellular localization of the proteins identified in the Pap test fluid (Pap), the cervical swab (Swab), and the tumor tissue (Tumor) is shown. The percentage of proteins in each of the GO categories is shown. From the bottom: cytoplasm (blue), intracellular organelles (brown), nuclear (green), extracellular (purple) plasma membrane (light blue) cytoskeleton (orange). Proteins which could not be assigned to a GO category (unknown) made up less than 20 percent of the proteins from each biospecimen and were excluded from the analysis. **b** Bar chart showing the PANTHER Protein Class ontologies of the proteins identified in biospecimens from an ovarian cancer patient based on the percentage of proteins mapped to a Gene ID in PANTHER. For each biospecimen: Pap test fluid (Pap test, green), 897 proteins; cervical swab (Swab, orange), 1214 proteins, and tumor tissue (Tumor, blue), 2755 proteins were used to assign PANTHER Protein Class ontology categories. PANTHER Protein Classes comprising less than 1% of proteins in all three biospecimens are not shown

We used Scaffold software to classify the proteins identified by cellular localization using Gene Ontology (GO) (Fig. 4a) [12, 13]. Slightly more than half of the total proteins identified in each biospecimen were localized to the cytoplasm or to intracellular organelles. The percentage of proteins in each category was quite similar for the swab and the tumor tissue; however, in the Pap test fluid, the percentage of nuclear proteins was lower and the percentage of extracellular proteins was higher than in either the swab or tumor tissue. This result is not unexpected, as the Pap test fluid was centrifuged prior to analysis to create a cell-free supernatant, while the swab may contain cellular components as well as extracellular secretions. The cellular localization of the 2293 proteins identified in all three biospecimens was similar to that found for the proteins found in the swab and tumor tissue (data not shown). We also examined the molecular function of the proteins identified using PANTHER to classify proteins into Protein Class Ontologies (Fig. 4b). PANTHER Protein Class ontology includes commonly used classes of protein functions, many of which are not covered by GO molecular function [14]. Overall, the protein functional classes are remarkably similar between the different biospecimens, with proteins mapped to 21 protein classes in all three biospecimens. However, the percentage of proteins in the defense/immunity class was higher in the Pap test fluid and swab compared to the tumor tissue. Conversely, more proteins in the nucleic acid binding protein class were identified in the tumor tissue than in the Pap test fluid and swab biospecimens.

### Identification of ovarian cancer biomarker proteins in the biospecimens

**Fig. 5:**
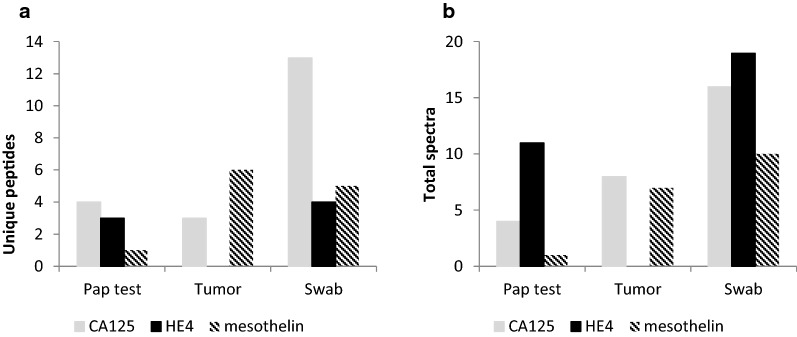
Ovarian cancer biomarker peptides were identified in biospecimens. Bar graph showing the number of **a** unique peptides, or **b** spectra identified in the Pap test fluid, the tumor tissue extract, and cervical swabs for three ovarian cancer biomarkers

Biomarker proteins known to be overexpressed in serum from ovarian cancer patients, such as CA125 (MUC16), HE4, and mesothelin, were found in the biospecimens (Fig. 5). Peptides from all three proteins were identified in both the Pap test fluid and swab (Fig. 5a). The tumor tissue contained peptides from CA125 and mesothelin, but not HE4 (Fig. 5a). Using the number of spectra as a rough estimate of biomarker protein abundance in the different biospecimens, we detected more of these three biomarkers in the swab than in the Pap test fluid or tumor tissue (Fig. 5b).

 Table 1 shows the peptides and spectra assigned to each sample type for 10 biomarker proteins that have been shown in the literature to have elevated expression in ovarian cancer serum or tissues. In addition to CA125 [16] and mesothelin [17], peptides to leucine-rich-alpha2-glycoprotein (LRG) [18] and CD44 [19] were found in all three biospecimens. Similar to the biomarker HE4 [20], peptides for Urokinase plasminogen activator surface receptor (UPAR) and folate-receptor-alpha (FOLR-a) [21, 22] were found in the swab and Pap test fluid, but not in the tumor tissue (although only a single peptide for FOLR-a was found in the Pap test fluid). Although expression of Nectin-4, Kallikrein-10, and Kallikrein-13 have been reported to be elevated in ovarian cancer tumor tissues and serum or ascites [23–26], peptides for these 3 biomarkers were only observed in the Pap test fluid, but not in the tumor tissue or swab.

**Table 1:** Ovarian cancer protein biomarkers identified in biospecimens from a patient with high grade serous ovarian cancer

Protein name	Pap test fluid		Tumor extract		Cervical swab		Citation
	Total unique peptides	Total spectra	Total unique peptides	Total spectra	Total unique peptides	Total spectra	
CA125	4	4	3	5	13	16	[16]
Mesothelin	1	1	6	7	5	10	[17]
LRG	8	25	3	5	6	50	[18]
CD44	1	1	7	13	1	1	[19]
HE4	3	11	0	0	4	19	[20]
Folate receptor alpha	1	2	0	0	2	4	[22]
UPAR	1	1	0	0	4	5	[21]
Nectin-4	3	3	0	0	0	0	[23]
Kallikrein-10	2	2	0	0	0	0	[24]
Kallikrein-13	3	3	0	0	0	0	[26]

## Discussion

Our data demonstrate that ovarian cancer biomarkers can be detected in Pap test fluid or a cervical swab by MS-based proteomics. In addition to identifying multiple known biomarkers, over 2000 proteins were detected in all three biospecimens, suggesting a potential role for novel biomarker discovery.

Several ovarian cancer serum biomarkers, such as mesothelin and LRG, were identified in all three biospecimens. Both mesothelin and LRG have been used in combination with CA125 to improve ovarian cancer detection [18, 27, 28]; both proteins have also been detected in urine of ovarian cancer patients [29, 30]. We also identified peptides from the cell adhesion molecule CD44 in all three biospecimens, with the highest number of CD44 peptides found in the tumor tissue. Although CD44 can be detected in serum from ovarian cancer patients [19], it has not been widely tested as a diagnostic biomarker, but rather as a marker of ovarian cancer stem cells [31].

HE4 and CA125 are FDA-approved serum biomarkers used for monitoring response to therapy in ovarian cancer patients [32–35]. We detected peptides from both of these proteins in the Pap test and swab samples, with the most peptides and spectra for both CA125 and HE4 identified in the swab sample. No peptides to HE4 were identified in the tumor tissue. HE4 is a small (~ 14 kDa) secreted protein, which could explain our inability to detect any HE4 peptides in the tumor tissue. While HE4 protein is overexpressed in over 90% of ovarian cancer tumors, it has also been detected in normal fallopian tubes, endometrium and cervix by immunohistochemistry [36], although we did not observe peptides to HE4 in the “Normal Pap test Core Proteome” defined in our previous analysis of normal Pap test fluid [8]. Interestingly, while the number of unique peptides identified for CA125 is larger than for HE4, the number of spectra for HE4 is larger than for CA125. Given that CA125 (MUC16) is a very large protein (over 1500 kDa) while HE4 is quite small (~ 14 kDa), this result is not unexpected. The fact that numerous spectra matching the HE4 protein were found is an indicator that this protein is rather high in abundance in the sample.

Several additional biomarkers identified in our study were also found in the Pap test fluid and the swab, but not in the tumor tissue. Nectin-4, UPAR, and FOLR-a are proteins expressed on the cell surface of the tumor, but can be cleaved by the action of proteases and shed into sera and other body fluids [21, 23, 37–39]. Kallikreins 10 and 13 were found only in the Pap test sample. Kallikreins are a family of secreted serine proteases that can be detected in the serum and tissues of ovarian cancer patients [24, 26]. While the expression of these proteins in the tumor extract would be expected, it is possible that, due to tumor heterogeneity, our MS analysis of a small piece of tumor was unable to detect them, while the Pap test fluid and the swab sample would detect proteins shed or secreted by the whole tumor. The absence of some biomarkers in the tumor tissue raises the possibility that some of the peptides identified in the Pap test fluid and swabs may be the result of protein expression in the cervical cells and not shed from the tumor. Indeed, in our previous study we detected peptides from the CA125 protein (MUC16) in Pap tests from women with normal cervical cytology [8]. While CA125, HE4 and mesothelin are not specific for ovarian cancer, as they are known to be expressed in the normal müllerian tract, we did not detect peptides from either HE4 or mesothelin in the “Normal Pap test Core Proteome” [8]. Further investigation using Pap tests or swabs from both normal and ovarian cancer specimens and quantitative MS will be necessary to determine if these proteins/peptides are detected at higher levels in ovarian cancer Pap tests/swabs compared to controls. Their presence alone is not sufficient for diagnosis.

The use of biospecimens proximal to the tumor site has the potential to improve biomarker detection [7]. The Pap test has previously been investigated for ovarian cancer detection using DNA [40, 41], but has never been examined for the presence of protein biomarkers. Using a sensitive method of DNA sequencing, Wang and colleagues were able to identify mutations in 29% of Pap brush samples from ovarian cancer patients, including from 28% of patients with early stage disease. When they examined samples collected from the intrauterine cavity of patients using a Tao brush, the detection of mutations in ovarian cancer samples increased to 45% [41]. Forty three percent of ovarian cancer patients in their study had detectable circulating tumor DNA (ctDNA), compared to 40% of Pap brush samples from the same patient cohort. It is possible that combining protein biomarkers with a DNA test in a Pap test or a vaginal/cervical swab could improve the sensitivity of ovarian cancer detection, allowing women to be tested for both cervical and ovarian cancers simultaneously. A combination of DNA mutation testing and multiple protein biomarkers in serum samples was shown to increase the sensitivity of pancreatic cancer detection [42]. A similar approach used a combination of DNA sequencing of ctDNA with serum protein biomarkers to test for several cancer types, including ovarian cancer [43], lending further credence to the possibility that by using both DNA and protein biomarkers, the Pap test could be developed to test for the presence of multiple gynecologic cancers.

Recently, a comprehensive proteomic analysis of microvesicles isolated from uterine lavage samples was used to construct a multi-protein classifier for ovarian cancer detection [44]. In our study of three biospecimens from an ovarian cancer patient, we identified seven of the nine proteins in the multiprotein classifier in at least one of our biospecimens. Five of the proteins (involucrin, CLCA4, S100A14, Serpin B5 and myosin-11) were found in the Pap test fluid; four of these proteins were also found in either the swab or tumor samples. The protein Nicotinamide N-methyltransferase (NNMT) was found in both the tumor tissue and the swab, but not the Pap test fluid. Myosin-11 peptides were found in all three of our biospecimens. Given that we have detected these and other ovarian cancer biomarkers in Pap test fluid and swabs, in addition to the relative ease of collecting a Pap test or vaginal swab in comparison to a uterine lavage, it may be accepted in more readily in clinics to use the Pap test for screening both ovarian and cervical cancers.

We have previously shown that the cell-free supernatant from residual Pap tests contained sufficient protein for analysis by 2D-LCMS, and identified 153 proteins from patients with normal cervical cytology in a “Normal Pap test Core Proteome” [8]. As might be expected, all of the proteins listed as components of the “Normal Pap test Core Proteome” were also identified in the Pap test fixative from the case of ovarian cancer analyzed in this study. Here, we also show that using a swab to collect proteins from the cervix is similar to the residual Pap test fluid in many regards; over 90% of the proteins identified in the Pap test fixative were also found in the cervical swab. In both the Pap test fluid and cervical swab, the most abundant protein identified was serum albumin; many other highly abundant serum proteins, such as immunoglobulins and complement proteins, were prevalent in both biospecimens. In addition, components of cervical mucus, such as mucin 5B, were also found at comparable levels in both biospecimens. In order to determine whether some of these proteins are biologically meaningful as ovarian cancer biomarkers, future studies will require a targeted approach, e.g. selected reaction monitoring (SRM) or parallel reaction monitoring (PRM), to quantify proteins that are elevated in ovarian cancer. For example, Elschenbroich, et al. [45] performed a comprehensive proteomic analysis of ovarian cancer ascites to identify candidate biomarkers, followed by relative biomarker quantification in an independent set of ascites and sera using stable isotope dilution-SRM assays [45]. Our observation that several proteins of interest (HE4, CA125) were detected with multiple peptides in both the Pap test and swab samples, using purely a discovery-based approach, bodes well that these proteins are present in high enough abundance that they could be detected robustly using targeted methods (SRM or PRM). Targeted MS methods are well known to be much more sensitive than discovery-based methods, and may also be more amenable to multiplexing than antibody based assays [46]. The similarity between the proteins identified in the Pap test and swab samples further suggests the possibility of using a swab sample to develop an “at home test” that would allow women to collect a cervical swab that could be sent to a reference laboratory for biomarker testing.

One notable difference between the proteins identified in the Pap test fluid compared to the swab was the relatively large number of spectra assigned to cytokeratin-4 and cytokeratin-13 in the Pap test fluid compared to the swab. These two cytokeratin proteins form a complex and are widely expressed in the exocervix [47]. These results may indicate that the two cervical sampling methods differ in the way that the proteins are collected. In this study, we also identified 1493 more proteins in the swab than in the Pap test fluid. Future studies are needed to determine whether the difference in the number of proteins identified between these two biospecimens is dependent upon the sample collection method, or varies with each individual or is dependent upon the physician who collects the clinical sample.

In contrast, the most abundant proteins detected in the ovarian cancer tumor tissue were hemoglobin-alpha and myosin-9. Both proteins are expressed in whole blood, suggesting that the tumor tissue was highly vascularized. Myosin-9 has also been identified as part of a gene signature from ovarian cancer stroma [48]. Stromal cells and other cell types in the tumor microenvironment are a component of the tumor tissue extract and the identification of these proteins would be expected. Alternatively, as the sample preparation method between the Pap test and swab proteins was different from the sample preparation of the tumor tissue, this could have affected the number of proteins identified. However, since more proteins were identified in the tumor tissue than either the Pap test fixative or the swab sample, this suggests that the lower ratio of trypsin used to digest the tumor tissue did not adversely affect the number of peptides recovered. Thus, the large number of proteins identified in the tumor tissue is more likely due to the multiple different cell types present in this sample compared to the Pap test and swab samples.

Future clinical applications may include ELISA tests using Pap test fluid or cervical swabs as the diagnostic biospecimen, or multiplexed proximity extension assays could be developed for use on Pap tests to sensitively quantify multiple proteins of interest [49, 50]. Alternatively, SRM-based targeted proteomic assays are increasingly being used in the measurement of clinically significant proteins, allowing for cost effective, high throughput, sensitive, robust, multiplexed analysis and quantification [46, 51]. Furthermore, since self-sampling improves participation in screening for human papilloma virus and is as sensitive as physician-obtained samples [52, 53], in the future it may be possible that this method could be translated into a self-administered home test, where swabs collected by women at home are sent to a central laboratory for analysis of proteins that would diagnose ovarian cancer.

## Conclusions

In summary, we have shown that several known ovarian cancer biomarker proteins are detectable in Pap test fluid and swab samples. Because Pap test screening is widely accepted, the development of the Pap test as a screening tool for both cervical and ovarian cancers might improve the efficacy of testing for a lethal but elusive disease. While our samples were from a single patient, the results are proof of concept: that Pap test fluid or cervical swabs could be used for detection of ovarian cancer biomarker proteins, and this approach warrants further investigation.

## Supplementary Information

**Additional file 1: Supplemental Excel file.** Lists of the proteins identified in each subset of the Venn diagram (Fig 2). Each tab shows the Protein accession numbers, Entry, Entry name, Protein names, and Gene names for proteins that were identified in each sample set: (i) 158 unique to the Pap and Swab samples, (ii) 186 unique to the Pap and Tumor samples, (iii) 1423 unique to the Swab and Tumor samples, (iv) 2293 present in the Pap, Swab and Tumor samples, (v) 64 unique to the Pap test sample, (vi) 320 unique to the Swab sample, and (vii) 490 unique to the Tumor sample.

**Additional file 2: Supplemental Table.** Protein identification details for proteins identified in the three biological samples (Pap test, cervical swab and tumor tissue). The protein names are listed alphabetically and show the corresponding protein accession number, protein molecular weight (Da), protein identification probability, exclusive unique peptide count, exclusive unique spectrum count, total spectrum count, percentage of total spectra, and percentage sequence coverage as determined by Scaffold analysis.

## Data Availability

The MS proteomics data in this paper have been deposited in the ProteomeXchange Consortium via the PRIDE [54] partner repository with the dataset identifier PXD023272.

## Abbreviations

2D: Two dimensional
BCA: Bicinchoninic acid
CA125: Cancer antigen 125
CDX-2: Homeobox protein CDX2
CK HMW: Cytokeratin high molecular weight
CLCA4: Calcium-activated chloride channel regulator 4
ctDNA: Circulating tumor DNA
CYK: Cytokeratin
Da: Dalton
FASP: Filter aided sample preparation
FDA: Food and Drug Administration
FDR: False discovery rate
FOLRa: Folate receptor alpha
GO: Gene Ontology
HE4: Human epididymis protein 4
HPLC: High pressure liquid chromatography
LRG: Leucine rich alpha 2 glycoprotein
LTQ: Linear trap quadrupole
LCMS: Liquid chromatography mass spectrometry
M: Molar
mM: Millimolar
MMTS: Methyl methanethiosulfonate
MS: Mass spectrometry
NNMT: Nicotinamide *N*-methyltransferase
PBS: Phosphate buffered saline
RP: Reversed phase
SPE: Solid phase extraction
SRM: Selected reaction monitoring
TEAB: Triethylammonium bicarbonate
TCEP: Tris(2-carboxyethyl)phosphine
UPAR: Urokinase plasminogen activator surface receptor
WT-1: Wilm’s tumor protein
