# Supplementary material for: Evaluation of the potential of Pap test fluid and cervical swabs to serve as clinical diagnostic biospecimens for the detection of ovarian cancer by mass spectrometry-based proteomics
Source: Clin Proteomics. 2021 Jan 7;18:4. doi: 10.1186/s12014-020-09309-3 (PMC7792339; doi:10.1186/s12014-020-09309-3)
Supplement: Supplementary file 2 — Additional file 2: Supplemental Table. Protein identification details for proteins identified in the three biological samples (Pap test, cervical swab and tumor tissue). The protein names are listed alphabetically and show the corresponding protein accession number, protein molecular weight (Da), protein identification probability, exclusive unique peptide count, exclusive unique spectrum count, total spectrum count, percentage of total spectra, and percentage sequence coverage as determined by Scaffold analysis. [file 12014_2020_9309_MOESM2_ESM.pdf]

Biological sample	Protein name	Protein accession numbers	Protein molecular weight (Da)	Protein identification probability	Exclusive unique peptide count	Exclusive unique spectrum count	Total spectrum count	Percentage of total spectra	Percentage sequence coverage
Tumor tissue	1,2-dihydroxy-3-keto-5-methylthiopentene dioxygenase GN=ADI1 PE=1 SV=1	sp|Q9BV57|MTND_HUMAN	21,499.20	100.00%	3	3	3	0.00230%	22.90%
Swab	1,4-alpha-glucan-branching enzyme GN=GBE1 PE=1 SV=3	GLGB_HUMAN	80,477.10	100.00%	4	4	4	0.00283%	8.40%
Tumor tissue	1,4-alpha-glucan-branching enzyme GN=GBE1 PE=1 SV=3	GLGB_HUMAN	80,477.10	100.00%	2	2	2	0.00153%	4.56%
Tumor tissue	10 kDa heat shock protein, mitochondrial GN=HSPE1 PE=1 SV=2	CH10_HUMAN	10,931.80	100.00%	2	3	17	0.01300%	43.10%
Pap test	14-3-3 protein epsilon GN=YWHAE PE=1 SV=1	sp|P62258|1433E_HUMAN	29,175.00	100.00%	5	8	34	0.05310%	45.90%
Swab	14-3-3 protein epsilon GN=YWHAE PE=1 SV=1	sp|P62258|1433E_HUMAN	29,175.00	100.00%	7	12	61	0.04320%	57.30%
Tumor tissue	14-3-3 protein epsilon GN=YWHAE PE=1 SV=1	sp|P62258|1433E_HUMAN	29,175.00	100.00%	8	14	46	0.03520%	60.40%
Pap test	14-3-3 protein eta GN=YWHAH PE=1 SV=4	1433F_HUMAN	28,219.60	100.00%	2	2	18	0.02810%	19.10%
Swab	14-3-3 protein eta GN=YWHAH PE=1 SV=4	1433F_HUMAN	28,219.60	100.00%	4	6	25	0.01770%	31.70%
Tumor tissue	14-3-3 protein eta GN=YWHAH PE=1 SV=4	1433F_HUMAN	28,219.60	100.00%	7	9	16	0.01220%	28.50%
Pap test	14-3-3 protein gamma GN=YWHAG PE=1 SV=2	1433G_HUMAN	28,303.10	100.00%	3	4	22	0.03430%	24.70%
Swab	14-3-3 protein gamma GN=YWHAG PE=1 SV=2	1433G_HUMAN	28,303.10	100.00%	3	4	25	0.01770%	28.30%
Tumor tissue	14-3-3 protein gamma GN=YWHAG PE=1 SV=2	1433G_HUMAN	28,303.10	100.00%	4	8	16	0.01220%	25.50%
Pap test	14-3-3 protein theta GN=YWHAQ PE=1 SV=1	1433T_HUMAN	27,765.40	100.00%	3	4	32	0.04990%	29.00%
Swab	14-3-3 protein theta GN=YWHAQ PE=1 SV=1	1433T_HUMAN	27,765.40	100.00%	8	10	33	0.02340%	46.90%
Tumor tissue	14-3-3 protein theta GN=YWHAQ PE=1 SV=1	1433T_HUMAN	27,765.40	100.00%	6	11	23	0.01760%	33.50%
Pap test	14-3-3 protein zeta/delta GN=YWHAZ PE=1 SV=1	sp|P63104|1433Z_HUMAN	27,745.90	100.00%	8	14	48	0.07490%	45.30%

Biological sample	Protein name	Protein accession numbers	Protein molecular weight (Da)	Protein identification probability	Exclusive unique peptide count	Exclusive unique spectrum count	Total spectrum count	Percentage of total spectra	Percentage sequence coverage
Swab	14-3-3 protein zeta/delta GN=YWHAZ PE=1 SV=1	sp|P63104|1433Z_HUMAN	27,745.90	100.00%	8	14	52	0.03690%	51.00%
Tumor tissue	14-3-3 protein zeta/delta GN=YWHAZ PE=1 SV=1	sp|P63104|1433Z_HUMAN	27,745.90	100.00%	9	16	50	0.03830%	44.50%
Tumor tissue	15 kDa selenoprotein GN=SEP15 PE=1 SV=1	SEP15_HUMAN	18,045.40	100.00%	3	4	6	0.00459%	19.50%
Pap test	15-hydroxyprostaglandin dehydrogenase [NAD(+)] GN=HPGD PE=1 SV=1	sp|P15428|PGDH_HUMAN	28,978.00	100.00%	1	1	6	0.00936%	17.70%
Swab	15-hydroxyprostaglandin dehydrogenase [NAD(+)] GN=HPGD PE=1 SV=1	sp|P15428|PGDH_HUMAN	28,978.00	100.00%	1	2	11	0.00780%	22.20%
Tumor tissue	182 kDa tankyrase-1-binding protein GN=TNKS1BP1 PE=1 SV=4	sp|Q9C0C2|TB182_HUMAN	181,795.00	100.00%	25	26	26	0.01990%	21.20%
Tumor tissue	1-acyl-sn-glycerol-3-phosphate acyltransferase alpha GN=AGPAT1 PE=1 SV=2	PLCA_HUMAN	31,717.10	100.00%	2	2	2	0.00153%	10.60%
Tumor tissue	2',5'-phosphodiesterase 12 GN=PDE12 PE=1 SV=1	sp|Q6L8Q7|PDE12_HUMAN	67,351.70	100.00%	2	2	2	0.00153%	4.66%
Tumor tissue	2'-5'-oligoadenylate synthase 2 GN=OAS2 PE=1 SV=1	sp|P29728|OAS2_HUMAN	83,238.20	100.00%	6	7	7	0.00536%	10.10%
Tumor tissue	2'-5'-oligoadenylate synthase 3 GN=OAS3 PE=1 SV=3	OAS3_HUMAN	121,172.00	100.00%	8	9	9	0.00689%	8.28%
Pap test	26S protease regulatory subunit 10B GN=PSMC6 PE=1 SV=1	PRS10_HUMAN	45,799.10	100.00%	1	1	2	0.00312%	7.20%
Swab	26S protease regulatory subunit 10B GN=PSMC6 PE=1 SV=1	PRS10_HUMAN	45,799.10	100.00%	1	2	4	0.00283%	14.40%
Tumor tissue	26S protease regulatory subunit 10B GN=PSMC6 PE=1 SV=1	PRS10_HUMAN	45,799.10	100.00%	8	13	27	0.02070%	32.00%
Swab	26S protease regulatory subunit 4 GN=PSMC1 PE=1 SV=1	sp|P62191|PRS4_HUMAN	49,186.40	100.00%	2	2	2	0.00142%	7.50%
Tumor tissue	26S protease regulatory subunit 4 GN=PSMC1 PE=1 SV=1	sp|P62191|PRS4_HUMAN	49,186.40	100.00%	9	13	17	0.01300%	32.00%
Pap test	26S protease regulatory subunit 6A GN=PSMC3 PE=1 SV=3	PRS6A_HUMAN	49,205.00	100.00%	1	1	2	0.00312%	5.69%

Biological sample	Protein name	Protein accession numbers	Protein molecular weight (Da)	Protein identification probability	Exclusive unique peptide count	Exclusive unique spectrum count	Total spectrum count	Percentage of total spectra	Percentage sequence coverage
Swab	26S protease regulatory subunit 6A GN=PSMC3 PE=1 SV=3	PRS6A_HUMAN	49,205.00	100.00%	2	2	6	0.00425%	12.50%
Tumor tissue	26S protease regulatory subunit 6A GN=PSMC3 PE=1 SV=3	PRS6A_HUMAN	49,205.00	100.00%	4	5	37	0.02830%	44.20%
Tumor tissue	26S protease regulatory subunit 6B GN=PSMC4 PE=1 SV=2	sp|P43686|PRS6B_HUMAN	47,367.70	100.00%	6	9	12	0.00919%	20.60%
Pap test	26S protease regulatory subunit 7 GN=PSMC2 PE=1 SV=3	sp|P35998|PRS7_HUMAN	48,635.60	99.40%	1	1	1	0.00156%	2.54%
Swab	26S protease regulatory subunit 7 GN=PSMC2 PE=1 SV=3	sp|P35998|PRS7_HUMAN	48,635.60	100.00%	3	3	4	0.00283%	10.60%
Tumor tissue	26S protease regulatory subunit 7 GN=PSMC2 PE=1 SV=3	sp|P35998|PRS7_HUMAN	48,635.60	100.00%	12	14	17	0.01300%	33.90%
Swab	26S protease regulatory subunit 8 GN=PSMC5 PE=1 SV=1	sp|P62195|PRS8_HUMAN	45,627.20	99.70%	1	1	1	0.00071%	3.45%
Tumor tissue	26S protease regulatory subunit 8 GN=PSMC5 PE=1 SV=1	sp|P62195|PRS8_HUMAN	45,627.20	100.00%	13	16	21	0.01610%	42.40%
Swab	26S proteasome non-ATPase regulatory subunit 1 GN=PSMD1 PE=1 SV=1	sp|Q99460|PSMD1_HUMAN	105,854.20	100.00%	1	1	1	0.00071%	1.99%
Tumor tissue	26S proteasome non-ATPase regulatory subunit 1 GN=PSMD1 PE=1 SV=1	sp|Q99460|PSMD1_HUMAN	105,854.20	100.00%	11	15	16	0.01220%	16.40%
Swab	26S proteasome non-ATPase regulatory subunit 10 GN=PSMD10 PE=1 SV=1	sp|O75832|PSD10_HUMAN	20,217.50	100.00%	3	4	5	0.00354%	26.50%
Tumor tissue	26S proteasome non-ATPase regulatory subunit 10 GN=PSMD10 PE=1 SV=1	sp|O75832|PSD10_HUMAN	20,217.50	100.00%	2	3	3	0.00230%	15.70%
Swab	26S proteasome non-ATPase regulatory subunit 12 GN=PSMD12 PE=1 SV=3	sp|O00232|PSD12_HUMAN	52,906.20	100.00%	1	1	1	0.00071%	4.39%
Tumor tissue	26S proteasome non-ATPase regulatory subunit 12 GN=PSMD12 PE=1 SV=3	sp|O00232|PSD12_HUMAN	52,906.20	100.00%	7	8	10	0.00766%	19.50%

Biological sample	Protein name	Protein accession numbers	Protein molecular weight (Da)	Protein identification probability	Exclusive unique peptide count	Exclusive unique spectrum count	Total spectrum count	Percentage of total spectra	Percentage sequence coverage
Tumor tissue	26S proteasome non-ATPase regulatory subunit 14 GN=PSMD14 PE=1 SV=1	PSDE_HUMAN	34,576.60	100.00%	2	3	3	0.00230%	9.68%
Swab	26S proteasome non-ATPase regulatory subunit 2 GN=PSMD2 PE=1 SV=3	sp|Q13200|PSMD2_HUMAN	100,202.20	100.00%	4	5	5	0.00354%	6.28%
Tumor tissue	26S proteasome non-ATPase regulatory subunit 2 GN=PSMD2 PE=1 SV=3	sp|Q13200|PSMD2_HUMAN	100,202.20	100.00%	13	15	31	0.02370%	25.60%
Pap test	26S proteasome non-ATPase regulatory subunit 3 GN=PSMD3 PE=1 SV=2	sp|O43242|PSMD3_HUMAN	60,979.60	100.00%	2	2	2	0.00312%	5.62%
Swab	26S proteasome non-ATPase regulatory subunit 3 GN=PSMD3 PE=1 SV=2	sp|O43242|PSMD3_HUMAN	60,979.60	100.00%	1	1	1	0.00071%	2.06%
Tumor tissue	26S proteasome non-ATPase regulatory subunit 3 GN=PSMD3 PE=1 SV=2	sp|O43242|PSMD3_HUMAN	60,979.60	100.00%	8	13	22	0.01680%	23.20%
Swab	26S proteasome non-ATPase regulatory subunit 4 GN=PSMD4 PE=1 SV=1	sp|P55036|PSMD4_HUMAN	40,737.20	100.00%	2	2	3	0.00213%	14.30%
Tumor tissue	26S proteasome non-ATPase regulatory subunit 4 GN=PSMD4 PE=1 SV=1	sp|P55036|PSMD4_HUMAN	40,737.20	100.00%	6	7	13	0.00995%	24.40%
Tumor tissue	26S proteasome non-ATPase regulatory subunit 7 GN=PSMD7 PE=1 SV=2	PSMD7_HUMAN	37,025.60	100.00%	5	6	24	0.01840%	41.70%
Swab	28 kDa heat- and acid-stable phosphoprotein GN=PDAP1 PE=1 SV=1	HAP28_HUMAN	20,630.80	99.10%	1	1	1	0.00071%	8.84%
Tumor tissue	28 kDa heat- and acid-stable phosphoprotein GN=PDAP1 PE=1 SV=1	HAP28_HUMAN	20,630.80	100.00%	5	6	9	0.00689%	28.20%
Tumor tissue	28S ribosomal protein S14, mitochondrial GN=MRPS14 PE=1 SV=1	RT14_HUMAN	15,139.10	100.00%	2	2	2	0.00153%	21.90%

Biological sample	Protein name	Protein accession numbers	Protein molecular weight (Da)	Protein identification probability	Exclusive unique peptide count	Exclusive unique spectrum count	Total spectrum count	Percentage of total spectra	Percentage sequence coverage
Tumor tissue	28S ribosomal protein S21, mitochondrial GN=MRPS21 PE=1 SV=1	RT21_HUMAN	10,688.90	100.00%	2	2	2	0.00153%	29.90%
Tumor tissue	28S ribosomal protein S22, mitochondrial GN=MRPS22 PE=1 SV=1	sp|P82650|RT22_HUMAN	41,210.50	100.00%	3	3	3	0.00230%	12.50%
Tumor tissue	28S ribosomal protein S23, mitochondrial GN=MRPS23 PE=1 SV=1	RT23_HUMAN	17,518.00	100.00%	2	2	2	0.00153%	13.20%
Tumor tissue	28S ribosomal protein S27, mitochondrial GN=MRPS27 PE=1 SV=1	sp|P82650|RT22_HUMAN	41,330.20	100.00%	4	5	5	0.00383%	14.50%
Tumor tissue	28S ribosomal protein S31, mitochondrial GN=MRPS31 PE=1 SV=3	RT31_HUMAN	45,320.10	100.00%	2	2	3	0.00230%	6.58%
Tumor tissue	28S ribosomal protein S33, mitochondrial GN=MRPS33 PE=1 SV=1	RT33_HUMAN	11,457.10	100.00%	2	2	2	0.00153%	21.90%
Tumor tissue	28S ribosomal protein S35, mitochondrial GN=MRPS35 PE=1 SV=1	sp|P82673|RT35_HUMAN	36,845.00	100.00%	4	4	4	0.00306%	21.70%
Tumor tissue	28S ribosomal protein S36, mitochondrial GN=MRPS36 PE=1 SV=2	RT36_HUMAN	11,466.40	100.00%	2	3	3	0.00230%	28.20%
Tumor tissue	28S ribosomal protein S7, mitochondrial GN=MRPS7 PE=1 SV=1	RT07_HUMAN	31,705.10	100.00%	3	3	3	0.00230%	13.30%
Tumor tissue	28S ribosomal protein S9, mitochondrial GN=MRPS9 PE=1 SV=2	RT09_HUMAN	45,836.00	100.00%	2	2	2	0.00153%	8.33%
Tumor tissue	2-methoxy-6-polyprenyl-1,4-benzoquinol methylase, mitochondrial GN=COQ5 PE=1 SV=1	sp|Q5HYK3|COQ5_HUMAN	28,533.40	100.00%	1	1	2	0.00153%	12.60%

Biological sample	Protein name	Protein accession numbers	Protein molecular weight (Da)	Protein identification probability	Exclusive unique peptide count	Exclusive unique spectrum count	Total spectrum count	Percentage of total spectra	Percentage sequence coverage
Tumor tissue	2-oxoglutarate dehydrogenase, mitochondrial GN=OGDH PE=1 SV=3	sp|Q02218|ODO1_HUMAN	115,936.70	100.00%	10	10	14	0.01070%	13.80%
Pap test	3'(2'),5'-bisphosphate nucleotidase 1 GN=BPNT1 PE=1 SV=2	sp|O95861|BPNT1_HUMAN	31,707.50	99.90%	1	1	1	0.00156%	3.78%
Swab	3'(2'),5'-bisphosphate nucleotidase 1 GN=BPNT1 PE=1 SV=2	sp|O95861|BPNT1_HUMAN	31,707.50	100.00%	5	5	6	0.00425%	27.10%
Tumor tissue	3'(2'),5'-bisphosphate nucleotidase 1 GN=BPNT1 PE=1 SV=2	sp|O95861|BPNT1_HUMAN	31,707.50	100.00%	2	2	2	0.00153%	8.59%
Tumor tissue	39S ribosomal protein L1, mitochondrial GN=MRPL1 PE=1 SV=2	RM01_HUMAN	36,910.00	100.00%	2	2	2	0.00153%	6.77%
Tumor tissue	39S ribosomal protein L13, mitochondrial GN=MRPL13 PE=1 SV=1	RM13_HUMAN	20,692.70	100.00%	2	2	2	0.00153%	14.00%
Tumor tissue	39S ribosomal protein L14, mitochondrial GN=MRPL14 PE=1 SV=1	RM14_HUMAN	15,947.70	99.90%	2	2	2	0.00153%	16.60%
Tumor tissue	39S ribosomal protein L27, mitochondrial GN=MRPL27 PE=1 SV=1	RM27_HUMAN	10,473.90	100.00%	2	3	3	0.00230%	28.00%
Tumor tissue	39S ribosomal protein L44, mitochondrial GN=MRPL44 PE=1 SV=1	RM44_HUMAN	37,536.10	100.00%	3	3	3	0.00230%	9.94%
Tumor tissue	39S ribosomal protein L46, mitochondrial GN=MRPL46 PE=1 SV=1	RM46_HUMAN	31,706.30	100.00%	2	2	2	0.00153%	9.68%
Tumor tissue	39S ribosomal protein L50, mitochondrial GN=MRPL50 PE=1 SV=2	sp|Q8N5N7|RM50_HUMAN	18,325.00	100.00%	3	5	5	0.00383%	29.70%
Tumor tissue	39S ribosomal protein L9, mitochondrial GN=MRPL9 PE=1 SV=2	RM09_HUMAN	30,243.20	100.00%	2	2	2	0.00153%	9.36%
Pap test	3-hydroxyacyl-CoA dehydrogenase type-2 GN=HSD17B10 PE=1 SV=3	sp|Q99714|HCD2_HUMAN	26,923.10	99.00%	1	1	1	0.00156%	7.66%

Biological sample	Protein name	Protein accession numbers	Protein molecular weight (Da)	Protein identification probability	Exclusive unique peptide count	Exclusive unique spectrum count	Total spectrum count	Percentage of total spectra	Percentage sequence coverage
Tumor tissue	3-hydroxyacyl-CoA dehydrogenase type-2 GN=HSD17B10 PE=1 SV=3	sp|Q99714|HCD2_HUMAN	26,923.10	100.00%	9	16	38	0.02910%	54.80%
Swab	3-hydroxybutyrate dehydrogenase type 2 GN=BDH2 PE=1 SV=2	sp|Q9BUT1|BDH2_HUMAN	26,723.90	99.60%	1	1	1	0.00071%	4.90%
Tumor tissue	3-hydroxybutyrate dehydrogenase type 2 GN=BDH2 PE=1 SV=2	sp|Q9BUT1|BDH2_HUMAN	26,723.90	100.00%	1	1	4	0.00306%	19.60%
Tumor tissue	3-hydroxyisobutyrate dehydrogenase, mitochondrial GN=HIBADH PE=1 SV=2	3HIDH_HUMAN	35,330.00	100.00%	8	11	14	0.01070%	32.10%
Pap test	3-hydroxyisobutyryl-CoA hydrolase, mitochondrial GN=HIBCH PE=1 SV=2	sp|Q6NVY1|HIBCH_HUMAN	43,484.00	99.90%	1	1	1	0.00156%	3.11%
Tumor tissue	3-hydroxyisobutyryl-CoA hydrolase, mitochondrial GN=HIBCH PE=1 SV=2	sp|Q6NVY1|HIBCH_HUMAN	43,484.00	100.00%	4	4	6	0.00459%	18.90%
Tumor tissue	3-ketoacyl-CoA thiolase, mitochondrial GN=ACAA2 PE=1 SV=1	THIM_HUMAN	41,601.70	100.00%	7	13	15	0.01150%	25.60%
Tumor tissue	3-ketodihydrosphingosine reductase (Fragment) GN=KDSR PE=1 SV=1	K7ERC8_HUMAN	22,620.10	99.80%	1	1	2	0.00153%	12.30%
Tumor tissue	3-oxoacyl-[acyl-carrier-protein] synthase, mitochondrial GN=OXSM PE=1 SV=1	sp|Q9NWU1|OXSM_HUMAN	48,843.40	100.00%	2	2	2	0.00153%	9.37%
Tumor tissue	3-phosphoinositide-dependent protein kinase 1 GN=PDPK1 PE=1 SV=1	sp|O15530|PDPK1_HUMAN	60,330.70	100.00%	2	2	2	0.00153%	6.43%
Tumor tissue	40S ribosomal protein S10 GN=RPS10 PE=1 SV=1	RS10_HUMAN	18,898.30	100.00%	2	3	22	0.01680%	33.30%
Pap test	40S ribosomal protein S11 GN=RPS11 PE=1 SV=3	RS11_HUMAN	18,431.30	98.40%	1	1	1	0.00156%	6.33%
Tumor tissue	40S ribosomal protein S11 GN=RPS11 PE=1 SV=3	RS11_HUMAN	18,431.30	100.00%	4	4	4	0.00306%	18.40%
Pap test	40S ribosomal protein S12 GN=RPS12 PE=1 SV=3	RS12_HUMAN	14,514.80	100.00%	4	4	4	0.00624%	33.30%

Biological sample	Protein name	Protein accession numbers	Protein molecular weight (Da)	Protein identification probability	Exclusive unique peptide count	Exclusive unique spectrum count	Total spectrum count	Percentage of total spectra	Percentage sequence coverage
Swab	40S ribosomal protein S12 GN=RPS12 PE=1 SV=3	RS12_HUMAN	14,514.80	99.90%	1	1	1	0.00071%	7.58%
Tumor tissue	40S ribosomal protein S12 GN=RPS12 PE=1 SV=3	RS12_HUMAN	14,514.80	100.00%	1	1	1	0.00077%	7.58%
Pap test	40S ribosomal protein S14 GN=RPS14 PE=1 SV=3	RS14_HUMAN	16,272.90	99.20%	1	1	1	0.00156%	8.61%
Swab	40S ribosomal protein S14 GN=RPS14 PE=1 SV=3	RS14_HUMAN	16,272.90	100.00%	2	2	3	0.00213%	15.90%
Tumor tissue	40S ribosomal protein S14 GN=RPS14 PE=1 SV=3	RS14_HUMAN	16,272.90	100.00%	6	9	12	0.00919%	37.10%
Tumor tissue	40S ribosomal protein S15 GN=RPS15 PE=1 SV=1	A0A0B4J2B4_HUMAN	13,742.80	100.00%	3	5	5	0.00383%	33.90%
Swab	40S ribosomal protein S15a GN=RPS15A PE=1 SV=2	RS15A_HUMAN	14,840.00	100.00%	1	1	1	0.00071%	10.80%
Tumor tissue	40S ribosomal protein S15a GN=RPS15A PE=1 SV=2	RS15A_HUMAN	14,840.00	100.00%	6	11	17	0.01300%	46.90%
Swab	40S ribosomal protein S16 GN=RPS16 PE=1 SV=2	RS16_HUMAN	16,445.90	100.00%	2	2	6	0.00425%	34.20%
Tumor tissue	40S ribosomal protein S16 GN=RPS16 PE=1 SV=2	RS16_HUMAN	16,445.90	100.00%	4	6	26	0.01990%	49.30%
Pap test	40S ribosomal protein S17 GN=RPS17 PE=1 SV=2	RS17_HUMAN	15,550.50	99.20%	1	1	1	0.00156%	16.30%
Swab	40S ribosomal protein S17 GN=RPS17 PE=1 SV=2	RS17_HUMAN	15,550.50	100.00%	2	2	3	0.00213%	24.40%
Tumor tissue	40S ribosomal protein S17 GN=RPS17 PE=1 SV=2	RS17_HUMAN	15,550.50	100.00%	1	1	5	0.00383%	14.80%
Pap test	40S ribosomal protein S18 GN=RPS18 PE=1 SV=3	RS18_HUMAN	17,719.30	100.00%	1	1	1	0.00156%	5.92%
Swab	40S ribosomal protein S18 GN=RPS18 PE=1 SV=3	RS18_HUMAN	17,719.30	100.00%	2	2	3	0.00213%	17.10%
Tumor tissue	40S ribosomal protein S18 GN=RPS18 PE=1 SV=3	RS18_HUMAN	17,719.30	100.00%	4	6	12	0.00919%	30.30%
Pap test	40S ribosomal protein S19 GN=RPS19 PE=1 SV=2	RS19_HUMAN	16,060.50	100.00%	4	4	4	0.00624%	29.70%
Swab	40S ribosomal protein S19 GN=RPS19 PE=1 SV=2	RS19_HUMAN	16,060.50	100.00%	6	7	8	0.00567%	42.10%

Biological sample	Protein name	Protein accession numbers	Protein molecular weight (Da)	Protein identification probability	Exclusive unique peptide count	Exclusive unique spectrum count	Total spectrum count	Percentage of total spectra	Percentage sequence coverage
Tumor tissue	40S ribosomal protein S19 GN=RPS19 PE=1 SV=2	RS19_HUMAN	16,060.50	100.00%	10	13	17	0.01300%	51.70%
Tumor tissue	40S ribosomal protein S2 GN=RPS2 PE=1 SV=2	RS2_HUMAN	31,325.20	100.00%	2	2	17	0.01300%	21.80%
Pap test	40S ribosomal protein S21 GN=RPS21 PE=1 SV=1	RS21_HUMAN	9,111.60	99.20%	1	1	1	0.00156%	12.30%
Swab	40S ribosomal protein S21 GN=RPS21 PE=1 SV=1	RS21_HUMAN	8,850.30	99.60%	1	1	2	0.00142%	12.30%
Tumor tissue	40S ribosomal protein S21 GN=RPS21 PE=1 SV=1	RS21_HUMAN	8,850.30	100.00%	4	6	7	0.00536%	49.40%
Tumor tissue	40S ribosomal protein S23 GN=RPS23 PE=1 SV=3	RS23_HUMAN	15,807.70	100.00%	5	6	9	0.00689%	22.40%
Tumor tissue	40S ribosomal protein S24 GN=RPS24 PE=1 SV=1	sp|P62847|RS24_HUMAN	15,197.50	100.00%	5	10	16	0.01220%	35.60%
Tumor tissue	40S ribosomal protein S25 GN=RPS25 PE=1 SV=1	RS25_HUMAN	13,743.00	100.00%	2	2	4	0.00306%	16.80%
Tumor tissue	40S ribosomal protein S26 GN=RPS26 PE=1 SV=3	RS26_HUMAN	13,015.50	100.00%	2	2	2	0.00153%	20.90%
Tumor tissue	40S ribosomal protein S27 GN=RPS27 PE=1 SV=1	RS27_HUMAN	7,356.20	100.00%	2	2	7	0.00536%	37.90%
Tumor tissue	40S ribosomal protein S27 GN=RPS27L PE=1 SV=1	RS27L_HUMAN	10,885.80	99.30%	1	1	4	0.00306%	24.70%
Pap test	40S ribosomal protein S28 GN=RPS28 PE=1 SV=1	RS28_HUMAN	7,840.90	100.00%	2	2	2	0.00312%	30.40%
Swab	40S ribosomal protein S28 GN=RPS28 PE=1 SV=1	RS28_HUMAN	7,840.90	100.00%	3	3	6	0.00425%	46.40%
Tumor tissue	40S ribosomal protein S28 GN=RPS28 PE=1 SV=1	RS28_HUMAN	7,840.90	100.00%	3	4	7	0.00536%	36.20%
Pap test	40S ribosomal protein S3 GN=RPS3 PE=1 SV=2	sp|P23396|RS3_HUMAN	26,688.60	100.00%	5	6	9	0.01400%	21.80%
Swab	40S ribosomal protein S3 GN=RPS3 PE=1 SV=2	sp|P23396|RS3_HUMAN	26,688.60	100.00%	6	6	9	0.00638%	30.50%
Tumor tissue	40S ribosomal protein S3 GN=RPS3 PE=1 SV=2	sp|P23396|RS3_HUMAN	26,688.60	100.00%	12	20	33	0.02530%	56.80%
Pap test	40S ribosomal protein S3a GN=RPS3A PE=1 SV=2	RS3A_HUMAN	29,945.30	100.00%	2	2	2	0.00312%	7.95%

Biological sample	Protein name	Protein accession numbers	Protein molecular weight (Da)	Protein identification probability	Exclusive unique peptide count	Exclusive unique spectrum count	Total spectrum count	Percentage of total spectra	Percentage sequence coverage
Swab	40S ribosomal protein S3a GN=RPS3A PE=1 SV=2	RS3A_HUMAN	29,945.30	100.00%	6	7	7	0.00496%	28.40%
Tumor tissue	40S ribosomal protein S3a GN=RPS3A PE=1 SV=2	RS3A_HUMAN	29,945.30	100.00%	8	13	18	0.01380%	36.00%
Pap test	40S ribosomal protein S4, X isoform GN=RPS4X PE=1 SV=2	RS4X_HUMAN	29,599.30	100.00%	2	2	3	0.00468%	7.22%
Swab	40S ribosomal protein S4, X isoform GN=RPS4X PE=1 SV=2	RS4X_HUMAN	29,599.30	100.00%	6	6	6	0.00425%	29.70%
Tumor tissue	40S ribosomal protein S4, X isoform GN=RPS4X PE=1 SV=2	RS4X_HUMAN	29,599.30	100.00%	8	13	21	0.01610%	31.60%
Pap test	40S ribosomal protein S5 GN=RPS5 PE=1 SV=1	M0R0R2_HUMAN	25,333.80	100.00%	2	3	5	0.00780%	12.40%
Swab	40S ribosomal protein S5 GN=RPS5 PE=1 SV=1	M0R0R2_HUMAN	25,333.80	100.00%	1	1	2	0.00142%	6.67%
Tumor tissue	40S ribosomal protein S5 GN=RPS5 PE=1 SV=1	M0R0R2_HUMAN	25,333.80	100.00%	3	5	6	0.00459%	12.40%
Swab	40S ribosomal protein S6 GN=RPS6 PE=1 SV=1	RS6_HUMAN	28,681.70	100.00%	2	2	3	0.00213%	8.03%
Tumor tissue	40S ribosomal protein S6 GN=RPS6 PE=1 SV=1	RS6_HUMAN	28,681.70	100.00%	5	8	11	0.00842%	21.70%
Pap test	40S ribosomal protein S7 GN=RPS7 PE=1 SV=1	RS7_HUMAN	22,127.50	98.60%	1	1	2	0.00312%	10.80%
Tumor tissue	40S ribosomal protein S7 GN=RPS7 PE=1 SV=1	RS7_HUMAN	22,127.50	100.00%	1	3	22	0.01680%	47.40%
Pap test	40S ribosomal protein S8 GN=RPS8 PE=1 SV=1	RS8_HUMAN	21,880.70	100.00%	2	2	2	0.00312%	12.20%
Swab	40S ribosomal protein S8 GN=RPS8 PE=1 SV=1	RS8_HUMAN	21,880.70	100.00%	2	2	3	0.00213%	14.90%
Tumor tissue	40S ribosomal protein S8 GN=RPS8 PE=1 SV=1	RS8_HUMAN	21,880.70	100.00%	6	9	18	0.01380%	33.00%
Pap test	40S ribosomal protein S9 GN=RPS9 PE=1 SV=3	RS9_HUMAN	22,592.50	100.00%	3	3	5	0.00780%	14.40%
Swab	40S ribosomal protein S9 GN=RPS9 PE=1 SV=3	RS9_HUMAN	22,592.50	100.00%	2	2	2	0.00142%	10.30%
Tumor tissue	40S ribosomal protein S9 GN=RPS9 PE=1 SV=3	RS9_HUMAN	22,592.50	100.00%	3	8	15	0.01150%	25.30%

Biological sample	Protein name	Protein accession numbers	Protein molecular weight (Da)	Protein identification probability	Exclusive unique peptide count	Exclusive unique spectrum count	Total spectrum count	Percentage of total spectra	Percentage sequence coverage
Pap test	40S ribosomal protein SA GN=RPSA PE=1 SV=1	RSSA_HUMAN	33,313.50	100.00%	4	4	4	0.00624%	21.70%
Swab	40S ribosomal protein SA GN=RPSA PE=1 SV=1	RSSA_HUMAN	33,313.50	100.00%	3	3	4	0.00283%	13.70%
Tumor tissue	40S ribosomal protein SA GN=RPSA PE=1 SV=1	RSSA_HUMAN	33,313.50	100.00%	6	11	19	0.01450%	26.30%
Tumor tissue	45 kDa calcium-binding protein GN=SDF4 PE=1 SV=1	sp|Q9BRK5|CAB45_HUMAN	41,806.90	100.00%	3	3	3	0.00230%	11.30%
Tumor tissue	4F2 cell-surface antigen heavy chain GN=SLC3A2 PE=1 SV=1	sp|P08195|4F2_HUMAN	68,103.30	100.00%	9	10	15	0.01150%	17.70%
Pap test	4-trimethylaminobutyraldehyde dehydrogenase GN=ALDH9A1 PE=1 SV=3	sp|P49189|AL9A1_HUMAN	53,802.00	100.00%	5	5	6	0.00936%	11.90%
Swab	4-trimethylaminobutyraldehyde dehydrogenase GN=ALDH9A1 PE=1 SV=3	sp|P49189|AL9A1_HUMAN	53,802.00	100.00%	8	9	15	0.01060%	20.60%
Tumor tissue	4-trimethylaminobutyraldehyde dehydrogenase GN=ALDH9A1 PE=1 SV=3	sp|P49189|AL9A1_HUMAN	53,802.00	100.00%	5	6	7	0.00536%	13.00%
Tumor tissue	5'(3')-deoxyribonucleotidase, cytosolic type GN=NT5C PE=1 SV=1	NT5C_HUMAN	13,618.60	100.00%	2	2	2	0.00153%	19.10%
Tumor tissue	5'-AMP-activated protein kinase subunit beta-1 GN=PRKAB1 PE=1 SV=4	AAKB1_HUMAN	30,382.70	100.00%	2	2	2	0.00153%	11.90%
Tumor tissue	5'-AMP-activated protein kinase subunit gamma-1 GN=PRKAG1 PE=1 SV=1	sp|P54619|AAKG1_HUMAN	37,579.80	100.00%	1	1	2	0.00153%	7.25%
Tumor tissue	5'-nucleotidase GN=NT5C3A PE=1 SV=1	sp|Q9H0P0|5NT3A_HUMAN	37,406.20	100.00%	2	2	2	0.00153%	8.16%
Swab	5'-nucleotidase GN=NT5E PE=1 SV=1	sp|P21589|5NTD_HUMAN	63,369.00	100.00%	5	5	7	0.00496%	10.10%
Tumor tissue	5'-nucleotidase GN=NT5E PE=1 SV=1	sp|P21589|5NTD_HUMAN	63,369.00	100.00%	10	12	13	0.00995%	23.70%
Tumor tissue	5-oxoprolinase GN=OPLAH PE=1 SV=3	OPLA_HUMAN	137,456.10	100.00%	8	8	8	0.00612%	9.55%

Biological sample	Protein name	Protein accession numbers	Protein molecular weight (Da)	Protein identification probability	Exclusive unique peptide count	Exclusive unique spectrum count	Total spectrum count	Percentage of total spectra	Percentage sequence coverage
Pap test	5-phosphohydroxy-L-lysine phospho-lyase GN=PHYKPL PE=1 SV=1	sp|Q8IUZ5|AT2L2_HUMAN	49,710.10	100.00%	2	2	2	0.00312%	5.33%
Swab	5-phosphohydroxy-L-lysine phospho-lyase GN=PHYKPL PE=1 SV=1	sp|Q8IUZ5|AT2L2_HUMAN	49,710.10	100.00%	2	2	2	0.00142%	5.33%
Pap test	60 kDa heat shock protein, mitochondrial GN=HSPD1 PE=1 SV=2	sp|P10809|CH60_HUMAN	61,055.70	100.00%	4	4	6	0.00936%	14.10%
Swab	60 kDa heat shock protein, mitochondrial GN=HSPD1 PE=1 SV=2	sp|P10809|CH60_HUMAN	61,055.70	100.00%	4	4	5	0.00354%	16.80%
Tumor tissue	60 kDa heat shock protein, mitochondrial GN=HSPD1 PE=1 SV=2	sp|P10809|CH60_HUMAN	61,055.70	100.00%	21	38	81	0.06200%	45.90%
Pap test	60S acidic ribosomal protein P0 GN=RPLP0 PE=1 SV=1	sp|P05388|RLA0_HUMAN	34,274.30	100.00%	3	3	4	0.00624%	13.90%
Swab	60S acidic ribosomal protein P0 GN=RPLP0 PE=1 SV=1	sp|P05388|RLA0_HUMAN	34,274.30	100.00%	3	3	4	0.00283%	10.40%
Tumor tissue	60S acidic ribosomal protein P0 GN=RPLP0 PE=1 SV=1	sp|P05388|RLA0_HUMAN	34,274.30	100.00%	10	14	28	0.02140%	38.80%
Pap test	60S acidic ribosomal protein P2 GN=RPLP2 PE=1 SV=1	RLA2_HUMAN	11,665.50	99.20%	1	1	2	0.00312%	10.40%
Swab	60S acidic ribosomal protein P2 GN=RPLP2 PE=1 SV=1	RLA2_HUMAN	11,665.50	100.00%	2	3	5	0.00354%	24.30%
Tumor tissue	60S acidic ribosomal protein P2 GN=RPLP2 PE=1 SV=1	RLA2_HUMAN	11,665.50	100.00%	8	16	28	0.02140%	77.40%
Pap test	60S ribosomal protein L10 GN=RPL10 PE=1 SV=2	F8W7C6_HUMAN	18,565.10	99.80%	1	2	3	0.00468%	19.00%
Swab	60S ribosomal protein L10 GN=RPL10 PE=1 SV=4	RL10_HUMAN	22,975.60	100.00%	1	1	2	0.00142%	18.20%
Swab	60S ribosomal protein L10a GN=RPL10A PE=1 SV=2	RL10A_HUMAN	24,832.00	100.00%	1	1	1	0.00071%	5.99%
Tumor tissue	60S ribosomal protein L10a GN=RPL10A PE=1 SV=2	RL10A_HUMAN	24,832.00	100.00%	7	12	16	0.01220%	24.00%

Biological sample	Protein name	Protein accession numbers	Protein molecular weight (Da)	Protein identification probability	Exclusive unique peptide count	Exclusive unique spectrum count	Total spectrum count	Percentage of total spectra	Percentage sequence coverage
Pap test	60S ribosomal protein L11 GN=RPL11 PE=1 SV=2	sp|P62913|RL11_HUMAN	20,253.20	99.90%	1	1	3	0.00468%	12.90%
Tumor tissue	60S ribosomal protein L11 GN=RPL11 PE=1 SV=2	sp|P62913|RL11_HUMAN	20,253.20	100.00%	1	1	5	0.00383%	12.90%
Pap test	60S ribosomal protein L12 GN=RPL12 PE=1 SV=1	sp|P30050|RL12_HUMAN	17,819.10	100.00%	4	5	6	0.00936%	33.90%
Swab	60S ribosomal protein L12 GN=RPL12 PE=1 SV=1	sp|P30050|RL12_HUMAN	17,819.10	100.00%	4	5	5	0.00354%	31.50%
Tumor tissue	60S ribosomal protein L12 GN=RPL12 PE=1 SV=1	sp|P30050|RL12_HUMAN	17,819.10	100.00%	3	6	17	0.01300%	24.20%
Tumor tissue	60S ribosomal protein L13 GN=RPL13 PE=1 SV=4	sp|P26373|RL13_HUMAN	24,262.20	100.00%	1	1	6	0.00459%	21.30%
Pap test	60S ribosomal protein L13a (Fragment) GN=RPL13A PE=1 SV=2	RL13A_HUMAN	24,216.30	99.70%	1	1	1	0.00156%	5.24%
Tumor tissue	60S ribosomal protein L13a (Fragment) GN=RPL13A PE=1 SV=2	RL13A_HUMAN	24,216.30	100.00%	4	5	6	0.00459%	18.60%
Tumor tissue	60S ribosomal protein L15 GN=RPL15 PE=1 SV=2	sp|P61313|RL15_HUMAN	24,146.50	100.00%	2	2	20	0.01530%	29.40%
Pap test	60S ribosomal protein L17 (Fragment) GN=RPL17 PE=3 SV=1	sp|P18621|RL17_HUMAN	19,786.20	100.00%	2	2	2	0.00312%	14.20%
Tumor tissue	60S ribosomal protein L17 (Fragment) GN=RPL17 PE=3 SV=1	sp|P18621|RL17_HUMAN	19,786.20	100.00%	7	11	15	0.01150%	36.10%
Pap test	60S ribosomal protein L18 (Fragment) GN=RPL18 PE=1 SV=1	sp|Q07020|RL18_HUMAN	21,840.20	100.00%	5	5	9	0.01400%	30.50%
Swab	60S ribosomal protein L18 (Fragment) GN=RPL18 PE=1 SV=1	sp|Q07020|RL18_HUMAN	21,840.20	100.00%	3	3	4	0.00283%	18.90%
Tumor tissue	60S ribosomal protein L18 (Fragment) GN=RPL18 PE=1 SV=1	sp|Q07020|RL18_HUMAN	21,840.20	100.00%	3	4	9	0.00689%	21.10%
Pap test	60S ribosomal protein L18a GN=RPL18A PE=1 SV=1	RL18A_HUMAN	17,477.60	100.00%	2	2	2	0.00312%	17.50%
Swab	60S ribosomal protein L18a GN=RPL18A PE=1 SV=1	RL18A_HUMAN	17,477.60	99.90%	1	1	1	0.00071%	8.44%
Tumor tissue	60S ribosomal protein L18a GN=RPL18A PE=1 SV=1	RL18A_HUMAN	18,079.40	100.00%	3	3	4	0.00306%	16.20%
Tumor tissue	60S ribosomal protein L21 GN=RPL21 PE=1 SV=2	RL21_HUMAN	18,565.00	100.00%	1	2	9	0.00689%	27.50%

Biological sample	Protein name	Protein accession numbers	Protein molecular weight (Da)	Protein identification probability	Exclusive unique peptide count	Exclusive unique spectrum count	Total spectrum count	Percentage of total spectra	Percentage sequence coverage
Swab	60S ribosomal protein L22 GN=RPL22 PE=1 SV=1	RL22_HUMAN	11,168.90	99.60%	1	1	1	0.00071%	13.70%
Tumor tissue	60S ribosomal protein L22 GN=RPL22 PE=1 SV=1	RL22_HUMAN	11,168.90	100.00%	2	2	2	0.00153%	25.30%
Tumor tissue	60S ribosomal protein L23 GN=RPL23 PE=1 SV=1	RL23_HUMAN	14,865.90	100.00%	3	7	14	0.01070%	18.60%
Pap test	60S ribosomal protein L23a GN=RPL23A PE=1 SV=1	RL23A_HUMAN	17,696.20	100.00%	3	4	7	0.01090%	21.80%
Swab	60S ribosomal protein L23a GN=RPL23A PE=1 SV=1	RL23A_HUMAN	17,696.20	100.00%	2	2	3	0.00213%	16.00%
Tumor tissue	60S ribosomal protein L23a GN=RPL23A PE=1 SV=1	RL23A_HUMAN	17,696.20	100.00%	7	10	13	0.00995%	42.90%
Tumor tissue	60S ribosomal protein L24 GN=RPL24 PE=1 SV=1	C9JNW5_HUMAN	17,543.40	100.00%	2	3	6	0.00459%	14.70%
Pap test	60S ribosomal protein L27 GN=RPL27 PE=1 SV=2	RL27_HUMAN	15,798.40	100.00%	1	1	1	0.00156%	6.62%
Swab	60S ribosomal protein L27 GN=RPL27 PE=1 SV=2	RL27_HUMAN	15,798.40	100.00%	2	3	3	0.00213%	22.10%
Tumor tissue	60S ribosomal protein L27 GN=RPL27 PE=1 SV=2	RL27_HUMAN	15,798.40	100.00%	6	10	15	0.01150%	44.90%
Pap test	60S ribosomal protein L27a GN=RPL27A PE=1 SV=2	RL27A_HUMAN	16,561.40	100.00%	2	2	2	0.00312%	14.20%
Tumor tissue	60S ribosomal protein L27a GN=RPL27A PE=1 SV=2	RL27A_HUMAN	16,561.40	100.00%	2	2	6	0.00459%	14.20%
Tumor tissue	60S ribosomal protein L29 GN=RPL29 PE=1 SV=2	RL29_HUMAN	17,753.00	100.00%	2	2	4	0.00306%	14.50%
Pap test	60S ribosomal protein L3 GN=RPL3 PE=1 SV=2	RL3_HUMAN	46,109.50	99.60%	1	1	1	0.00156%	2.98%
Swab	60S ribosomal protein L3 GN=RPL3 PE=1 SV=2	RL3_HUMAN	46,109.50	100.00%	2	2	2	0.00142%	4.96%
Tumor tissue	60S ribosomal protein L3 GN=RPL3 PE=1 SV=2	RL3_HUMAN	46,109.50	100.00%	12	19	27	0.02070%	28.80%
Pap test	60S ribosomal protein L30 (Fragment) GN=RPL30 PE=1 SV=1	RL30_HUMAN	12,656.50	100.00%	2	2	3	0.00468%	24.60%
Swab	60S ribosomal protein L30 (Fragment) GN=RPL30 PE=1 SV=1	RL30_HUMAN	12,656.50	100.00%	2	2	4	0.00283%	24.60%

Biological sample	Protein name	Protein accession numbers	Protein molecular weight (Da)	Protein identification probability	Exclusive unique peptide count	Exclusive unique spectrum count	Total spectrum count	Percentage of total spectra	Percentage sequence coverage
Tumor tissue	60S ribosomal protein L30 (Fragment) GN=RPL30 PE=1 SV=1	RL30_HUMAN	12,656.50	100.00%	2	4	4	0.00306%	18.40%
Swab	60S ribosomal protein L31 GN=RPL31 PE=1 SV=1	sp|P62899|RL31_HUMAN	14,463.20	98.50%	1	1	1	0.00071%	7.20%
Tumor tissue	60S ribosomal protein L31 GN=RPL31 PE=1 SV=1	sp|P62899|RL31_HUMAN	14,463.20	100.00%	2	3	7	0.00536%	17.60%
Tumor tissue	60S ribosomal protein L32 (Fragment) GN=RPL32 PE=1 SV=1	RL32_HUMAN	17,962.70	100.00%	4	5	7	0.00536%	18.80%
Tumor tissue	60S ribosomal protein L35 GN=RPL35 PE=1 SV=1	F2Z388_HUMAN	10,645.10	100.00%	3	5	6	0.00459%	28.10%
Pap test	60S ribosomal protein L35a GN=RPL35A PE=1 SV=2	RL35A_HUMAN	12,538.10	100.00%	1	1	1	0.00156%	8.18%
Swab	60S ribosomal protein L35a GN=RPL35A PE=1 SV=2	RL35A_HUMAN	12,538.10	100.00%	1	1	1	0.00071%	8.18%
Tumor tissue	60S ribosomal protein L35a GN=RPL35A PE=1 SV=2	RL35A_HUMAN	12,538.10	100.00%	2	3	3	0.00230%	19.10%
Tumor tissue	60S ribosomal protein L36 GN=RPL36 PE=1 SV=3	RL36_HUMAN	12,254.20	100.00%	1	1	3	0.00230%	19.00%
Tumor tissue	60S ribosomal protein L36a-like GN=RPL36AL PE=1 SV=3	RL36L_HUMAN	12,469.10	100.00%	2	2	2	0.00153%	17.90%
Tumor tissue	60S ribosomal protein L38 GN=RPL38 PE=1 SV=2	RL38_HUMAN	8,218.50	100.00%	3	4	6	0.00459%	48.60%
Pap test	60S ribosomal protein L4 GN=RPL4 PE=1 SV=5	RL4_HUMAN	47,699.10	100.00%	8	9	13	0.02030%	23.70%
Swab	60S ribosomal protein L4 GN=RPL4 PE=1 SV=5	RL4_HUMAN	47,699.10	100.00%	3	3	4	0.00283%	7.03%
Tumor tissue	60S ribosomal protein L4 GN=RPL4 PE=1 SV=5	RL4_HUMAN	47,699.10	100.00%	9	14	22	0.01680%	24.80%
Pap test	60S ribosomal protein L5 GN=RPL5 PE=1 SV=3	RL5_HUMAN	34,363.50	100.00%	5	5	5	0.00780%	24.60%
Swab	60S ribosomal protein L5 GN=RPL5 PE=1 SV=3	RL5_HUMAN	34,363.50	100.00%	2	4	5	0.00354%	8.75%
Tumor tissue	60S ribosomal protein L5 GN=RPL5 PE=1 SV=3	RL5_HUMAN	34,363.50	100.00%	9	17	21	0.01610%	23.90%
Pap test	60S ribosomal protein L6 GN=RPL6 PE=1 SV=3	RL6_HUMAN	32,729.30	100.00%	5	5	6	0.00936%	20.50%

Biological sample	Protein name	Protein accession numbers	Protein molecular weight (Da)	Protein identification probability	Exclusive unique peptide count	Exclusive unique spectrum count	Total spectrum count	Percentage of total spectra	Percentage sequence coverage
Swab	60S ribosomal protein L6 GN=RPL6 PE=1 SV=3	RL6_HUMAN	32,729.30	100.00%	2	2	3	0.00213%	4.51%
Tumor tissue	60S ribosomal protein L6 GN=RPL6 PE=1 SV=3	RL6_HUMAN	32,729.30	100.00%	11	22	45	0.03440%	38.50%
Pap test	60S ribosomal protein L7 GN=RPL7 PE=1 SV=1	RL7_HUMAN	29,227.70	100.00%	6	7	9	0.01400%	33.50%
Tumor tissue	60S ribosomal protein L7 GN=RPL7 PE=1 SV=1	RL7_HUMAN	29,227.70	100.00%	10	14	19	0.01450%	35.50%
Pap test	60S ribosomal protein L7a GN=RPL7A PE=1 SV=2	RL7A_HUMAN	29,996.30	100.00%	1	2	4	0.00624%	13.90%
Tumor tissue	60S ribosomal protein L7a GN=RPL7A PE=1 SV=2	RL7A_HUMAN	29,996.30	100.00%	6	9	25	0.01910%	29.30%
Pap test	60S ribosomal protein L8 GN=RPL8 PE=1 SV=2	RL8_HUMAN	28,024.80	99.90%	1	1	1	0.00156%	4.28%
Swab	60S ribosomal protein L8 GN=RPL8 PE=1 SV=2	RL8_HUMAN	28,024.80	98.50%	1	1	1	0.00071%	4.28%
Tumor tissue	60S ribosomal protein L8 GN=RPL8 PE=1 SV=2	RL8_HUMAN	28,024.80	100.00%	5	11	12	0.00919%	24.10%
Tumor tissue	60S ribosomal protein L9 GN=RPL9 PE=1 SV=1	RL9_HUMAN	21,863.70	100.00%	4	6	10	0.00766%	22.90%
Pap test	6-phosphogluconolactonase GN=PGLS PE=1 SV=2	6PGL_HUMAN	27,547.50	100.00%	2	2	2	0.00312%	10.50%
Swab	6-phosphogluconolactonase GN=PGLS PE=1 SV=2	6PGL_HUMAN	27,547.50	100.00%	7	7	15	0.01060%	42.60%
Tumor tissue	6-phosphogluconolactonase GN=PGLS PE=1 SV=2	6PGL_HUMAN	27,547.50	100.00%	7	7	17	0.01300%	41.10%
Pap test	78 kDa glucose-regulated protein GN=HSPA5 PE=1 SV=2	GRP78_HUMAN	72,334.70	100.00%	16	19	37	0.05770%	36.20%
Swab	78 kDa glucose-regulated protein GN=HSPA5 PE=1 SV=2	GRP78_HUMAN	72,334.70	100.00%	17	19	37	0.02620%	30.40%
Tumor tissue	78 kDa glucose-regulated protein GN=HSPA5 PE=1 SV=2	GRP78_HUMAN	72,334.70	100.00%	34	62	146	0.11200%	54.70%
Tumor tissue	7-dehydrocholesterol reductase GN=DHCR7 PE=1 SV=1	DHCR7_HUMAN	54,491.00	100.00%	1	1	2	0.00153%	4.00%
Tumor tissue	Abl interactor 1 GN=ABI1 PE=1 SV=1	sp|Q8IZP0|ABI1_HUMAN	43,295.50	100.00%	1	1	4	0.00306%	11.80%

Biological sample	Protein name	Protein accession numbers	Protein molecular weight (Da)	Protein identification probability	Exclusive unique peptide count	Exclusive unique spectrum count	Total spectrum count	Percentage of total spectra	Percentage sequence coverage
Pap test	Abl interactor 2 GN=ABI2 PE=1 SV=1	sp|Q9NYB9-3|ABI2_HUMAN	43,088.50	99.20%	1	1	2	0.00312%	5.13%
Tumor tissue	Abl interactor 2 GN=ABI2 PE=1 SV=1	sp|Q9NYB9-3|ABI2_HUMAN	43,088.50	100.00%	1	1	2	0.00153%	6.41%
Tumor tissue	Acetolactate synthase-like protein GN=ILVBL PE=1 SV=2	ILVBL_HUMAN	56,732.10	100.00%	2	3	4	0.00306%	6.80%
Pap test	Acetyl-CoA acetyltransferase, cytosolic GN=ACAT2 PE=1 SV=2	sp|Q9BWD1|THIC_HUMAN	41,350.40	99.20%	1	1	1	0.00156%	6.80%
Tumor tissue	Acetyl-CoA acetyltransferase, cytosolic GN=ACAT2 PE=1 SV=2	sp|Q9BWD1|THIC_HUMAN	41,350.40	100.00%	3	4	4	0.00306%	10.60%
Tumor tissue	Acetyl-CoA acetyltransferase, mitochondrial GN=ACAT1 PE=1 SV=1	sp|P24752|THIL_HUMAN	45,199.80	100.00%	4	4	16	0.01220%	24.80%
Pap test	Acid ceramidase GN=ASAH1 PE=1 SV=1	A0A1B0GVG2_HUMAN	38,016.60	100.00%	2	5	9	0.01400%	11.40%
Swab	Acid ceramidase GN=ASAH1 PE=1 SV=1	A0A1B0GVG2_HUMAN	38,016.60	100.00%	1	1	1	0.00071%	4.99%
Tumor tissue	Acid ceramidase GN=ASAH1 PE=1 SV=1	A0A1B0GVG2_HUMAN	38,016.60	100.00%	2	2	3	0.00230%	8.21%
Pap test	Acid ceramidase (Fragment) GN=ASAH1 PE=1 SV=1	A0A1B0GV06_HUMAN	22,949.10	100.00%	6	6	17	0.02650%	36.50%
Swab	Acid ceramidase (Fragment) GN=ASAH1 PE=1 SV=1	A0A1B0GV06_HUMAN	22,949.10	100.00%	6	6	8	0.00567%	36.50%
Tumor tissue	Acid ceramidase (Fragment) GN=ASAH1 PE=1 SV=1	A0A1B0GV06_HUMAN	22,949.10	100.00%	5	5	7	0.00536%	32.00%
Tumor tissue	Acid sphingomyelinase-like phosphodiesterase 3b GN=SMPDL3B PE=1 SV=1	sp|Q92485|ASM3B_HUMAN	45,315.00	100.00%	2	2	2	0.00153%	6.88%
Swab	Acidic leucine-rich nuclear phosphoprotein 32 family member A GN=ANP32A PE=1 SV=1	AN32A_HUMAN	19,998.50	100.00%	1	2	11	0.00780%	21.30%
Pap test	Aconitate hydratase, mitochondrial GN=ACO2 PE=1 SV=1	ACON_HUMAN	87,822.50	100.00%	8	9	10	0.01560%	13.90%
Swab	Aconitate hydratase, mitochondrial GN=ACO2 PE=1 SV=1	ACON_HUMAN	87,822.50	100.00%	6	6	7	0.00496%	10.40%

Biological sample	Protein name	Protein accession numbers	Protein molecular weight (Da)	Protein identification probability	Exclusive unique peptide count	Exclusive unique spectrum count	Total spectrum count	Percentage of total spectra	Percentage sequence coverage
Tumor tissue	Aconitate hydratase, mitochondrial GN=ACO2 PE=1 SV=1	ACON_HUMAN	87,822.50	100.00%	23	31	48	0.03670%	35.50%
Tumor tissue	Actin, aortic smooth muscle GN=ACTA2 PE=1 SV=1	ACTA_HUMAN	42,010.10	100.00%	3	20	249	0.19100%	63.70%
Pap test	Actin, cytoplasmic 1 GN=ACTB PE=1 SV=1	ACTB_HUMAN	41,737.80	100.00%	3	16	219	0.34200%	56.50%
Swab	Actin, cytoplasmic 1 GN=ACTB PE=1 SV=1	ACTB_HUMAN	41,737.80	100.00%	3	13	301	0.21300%	56.80%
Tumor tissue	Actin, cytoplasmic 1 GN=ACTB PE=1 SV=1	ACTB_HUMAN	41,793.90	100.00%	2	12	407	0.31200%	64.00%
Tumor tissue	Actin, cytoplasmic 1 (Fragment) GN=ACTB PE=1 SV=8	E7EVS6_HUMAN	17,878.20	98.60%	1	1	103	0.07880%	75.50%
Tumor tissue	Actin-like protein 6A GN=ACTL6A PE=1 SV=1	sp|O96019|ACL6A_HUMAN	47,461.10	100.00%	8	10	11	0.00842%	29.10%
Tumor tissue	Actin-like protein 8 GN=ACTL8 PE=1 SV=1	ACTL8_HUMAN	41,360.30	100.00%	3	3	3	0.00230%	14.50%
Tumor tissue	Actin-related protein 10 GN=ACTR10 PE=1 SV=1	ARP10_HUMAN	46,308.40	100.00%	1	1	2	0.00153%	7.91%
Pap test	Actin-related protein 2 GN=ACTR2 PE=1 SV=1	sp|P61160|ARP2_HUMAN	44,761.70	100.00%	6	9	13	0.02030%	19.80%
Swab	Actin-related protein 2 GN=ACTR2 PE=1 SV=1	sp|P61160|ARP2_HUMAN	44,761.70	100.00%	3	5	11	0.00780%	9.64%
Tumor tissue	Actin-related protein 2 GN=ACTR2 PE=1 SV=1	sp|P61160|ARP2_HUMAN	44,761.70	100.00%	8	23	41	0.03140%	26.10%
Tumor tissue	Actin-related protein 2/3 complex subunit 1A GN=ARPC1A PE=1 SV=2	sp|Q92747|ARC1A_HUMAN	41,569.20	100.00%	4	6	6	0.00459%	16.20%
Swab	Actin-related protein 2/3 complex subunit 1B GN=ARPC1B PE=1 SV=3	ARC1B_HUMAN	40,949.80	100.00%	1	1	2	0.00142%	4.84%
Tumor tissue	Actin-related protein 2/3 complex subunit 1B GN=ARPC1B PE=1 SV=3	ARC1B_HUMAN	40,949.80	100.00%	7	14	16	0.01220%	22.00%
Pap test	Actin-related protein 2/3 complex subunit 2 GN=ARPC2 PE=1 SV=1	ARPC2_HUMAN	34,333.70	100.00%	3	6	8	0.01250%	10.30%
Swab	Actin-related protein 2/3 complex subunit 2 GN=ARPC2 PE=1 SV=1	ARPC2_HUMAN	34,333.70	100.00%	2	2	3	0.00213%	8.67%
Tumor tissue	Actin-related protein 2/3 complex subunit 2 GN=ARPC2 PE=1 SV=1	ARPC2_HUMAN	34,333.70	100.00%	12	17	21	0.01610%	40.00%

Biological sample	Protein name	Protein accession numbers	Protein molecular weight (Da)	Protein identification probability	Exclusive unique peptide count	Exclusive unique spectrum count	Total spectrum count	Percentage of total spectra	Percentage sequence coverage
Pap test	Actin-related protein 2/3 complex subunit 3 GN=ARPC3 PE=1 SV=3	ARPC3_HUMAN	20,547.80	99.90%	1	1	3	0.00468%	7.30%
Tumor tissue	Actin-related protein 2/3 complex subunit 3 GN=ARPC3 PE=1 SV=3	ARPC3_HUMAN	20,547.80	100.00%	5	8	14	0.01070%	27.00%
Pap test	Actin-related protein 2/3 complex subunit 4 GN=ARPC4-TTLL3 PE=3 SV=1	sp|P59998|ARPC4_HUMAN	21,058.90	100.00%	1	1	5	0.00780%	14.90%
Tumor tissue	Actin-related protein 2/3 complex subunit 4 GN=ARPC4-TTLL3 PE=3 SV=1	sp|P59998|ARPC4_HUMAN	21,058.90	100.00%	2	3	11	0.00842%	33.70%
Pap test	Actin-related protein 2/3 complex subunit 5 GN=ARPC5 PE=1 SV=3	sp|O15511|ARPC5_HUMAN	16,320.60	99.40%	1	1	1	0.00156%	7.95%
Swab	Actin-related protein 2/3 complex subunit 5 GN=ARPC5 PE=1 SV=3	sp|O15511|ARPC5_HUMAN	16,320.60	99.90%	1	1	1	0.00071%	7.95%
Tumor tissue	Actin-related protein 2/3 complex subunit 5 GN=ARPC5 PE=1 SV=3	sp|O15511|ARPC5_HUMAN	16,320.60	100.00%	3	6	10	0.00766%	29.10%
Pap test	Actin-related protein 2/3 complex subunit 5-like protein GN=ARPC5L PE=1 SV=1	ARP5L_HUMAN	16,941.30	100.00%	2	2	2	0.00312%	16.30%
Swab	Actin-related protein 2/3 complex subunit 5-like protein GN=ARPC5L PE=1 SV=1	ARP5L_HUMAN	16,941.30	100.00%	2	2	3	0.00213%	16.30%
Tumor tissue	Actin-related protein 2/3 complex subunit 5-like protein GN=ARPC5L PE=1 SV=1	ARP5L_HUMAN	16,941.30	100.00%	3	3	3	0.00230%	28.80%
Pap test	Actin-related protein 3 GN=ACTR3 PE=1 SV=3	ARP3_HUMAN	47,371.90	100.00%	11	13	20	0.03120%	43.50%
Swab	Actin-related protein 3 GN=ACTR3 PE=1 SV=3	ARP3_HUMAN	47,371.90	100.00%	10	11	20	0.01420%	34.70%
Tumor tissue	Actin-related protein 3 GN=ACTR3 PE=1 SV=3	ARP3_HUMAN	47,371.90	100.00%	9	14	38	0.02910%	33.50%
Pap test	Activated RNA polymerase II transcriptional coactivator p15 GN=SUB1 PE=1 SV=3	TCP4_HUMAN	14,395.90	99.20%	1	1	1	0.00156%	10.20%

Biological sample	Protein name	Protein accession numbers	Protein molecular weight (Da)	Protein identification probability	Exclusive unique peptide count	Exclusive unique spectrum count	Total spectrum count	Percentage of total spectra	Percentage sequence coverage
Swab	Activated RNA polymerase II transcriptional coactivator p15 GN=SUB1 PE=1 SV=3	TCP4_HUMAN	14,395.90	99.90%	1	1	2	0.00142%	8.66%
Tumor tissue	Activated RNA polymerase II transcriptional coactivator p15 GN=SUB1 PE=1 SV=3	TCP4_HUMAN	14,395.90	100.00%	5	8	20	0.01530%	45.70%
Tumor tissue	Activating signal cointegrator 1 complex subunit 3 GN=ASCC3 PE=1 SV=3	sp|Q8N3C0|ASCC3_HUMAN	251,466.80	100.00%	2	2	3	0.00230%	1.68%
Tumor tissue	Activator of 90 kDa heat shock protein ATPase homolog 1 GN=AHSA1 PE=1 SV=1	sp|O95433|AHSA1_HUMAN	38,274.40	100.00%	6	9	14	0.01070%	27.50%
Tumor tissue	Active breakpoint cluster region-related protein (Fragment) GN=ABR PE=1 SV=1	sp|Q12979|ABR_HUMAN	88,861.40	100.00%	2	2	5	0.00383%	9.78%
Tumor tissue	Activity-dependent neuroprotector homeobox protein GN=ADNP PE=1 SV=1	ADNP_HUMAN	123,564.30	100.00%	3	3	3	0.00230%	4.54%
Tumor tissue	Acyl carrier protein, mitochondrial GN=NDUFAB1 PE=1 SV=3	I3L505_HUMAN	8,060.90	100.00%	2	2	2	0.00153%	11.50%
Pap test	Acylamino-acid-releasing enzyme GN=APEH PE=1 SV=4	C9JIF9_HUMAN	81,673.50	100.00%	3	4	13	0.02030%	12.70%
Swab	Acylamino-acid-releasing enzyme GN=APEH PE=1 SV=4	C9JIF9_HUMAN	81,673.50	100.00%	2	3	9	0.00638%	6.69%
Tumor tissue	Acylamino-acid-releasing enzyme GN=APEH PE=1 SV=4	C9JIF9_HUMAN	81,673.50	100.00%	4	4	8	0.00612%	13.10%
Tumor tissue	Acyl-CoA dehydrogenase family member 9, mitochondrial GN=ACAD9 PE=1 SV=1	ACAD9_HUMAN	68,762.80	100.00%	6	8	10	0.00766%	10.60%
Tumor tissue	Acyl-CoA synthetase family member 2, mitochondrial GN=ACSF2 PE=1 SV=1	sp|Q96CM8|ACSF2_HUMAN	63,652.90	100.00%	4	5	5	0.00383%	11.20%
Tumor tissue	Acyl-CoA synthetase family member 3, mitochondrial (Fragment) GN=ACSF3 PE=1 SV=1	F5H755_HUMAN	26,566.50	100.00%	1	1	2	0.00153%	11.90%

Biological sample	Protein name	Protein accession numbers	Protein molecular weight (Da)	Protein identification probability	Exclusive unique peptide count	Exclusive unique spectrum count	Total spectrum count	Percentage of total spectra	Percentage sequence coverage
Tumor tissue	Acyl-CoA synthetase short-chain family member 3, mitochondrial GN=ACSS3 PE=1 SV=1	sp|Q9H6R3|ACSS3_HUMAN	74,592.50	100.00%	3	3	3	0.00230%	6.72%
Pap test	Acyl-CoA-binding protein GN=DBI PE=1 SV=1	sp|P07108|ACBP_HUMAN	11,149.90	100.00%	1	2	6	0.00936%	25.20%
Swab	Acyl-CoA-binding protein GN=DBI PE=1 SV=1	sp|P07108|ACBP_HUMAN	11,149.90	100.00%	2	5	8	0.00567%	32.20%
Tumor tissue	Acyl-CoA-binding protein GN=DBI PE=1 SV=1	sp|P07108|ACBP_HUMAN	11,149.90	100.00%	1	3	7	0.00536%	25.20%
Tumor tissue	Acyl-coenzyme A thioesterase 13 GN=ACOT13 PE=1 SV=1	sp|Q9NPJ3|ACO13_HUMAN	14,960.60	100.00%	2	2	2	0.00153%	15.70%
Tumor tissue	Acyl-coenzyme A thioesterase 2, mitochondrial GN=ACOT2 PE=1 SV=1	A0A087WT95_HUMAN	50,930.70	100.00%	6	8	11	0.00842%	16.80%
Tumor tissue	Acyl-coenzyme A thioesterase 8 GN=ACOT8 PE=1 SV=1	ACOT8_HUMAN	35,913.40	100.00%	2	2	3	0.00230%	15.00%
Tumor tissue	Acylglycerol kinase, mitochondrial GN=AGK PE=1 SV=1	E9PC15_HUMAN	43,797.80	100.00%	4	4	5	0.00383%	14.70%
Tumor tissue	Acylphosphatase-1 GN=ACYP1 PE=1 SV=2	ACYP1_HUMAN	14,125.60	100.00%	2	2	2	0.00153%	25.30%
Swab	Acyl-protein thioesterase 1 GN=LYPLA1 PE=1 SV=1	A0A087X1K9_HUMAN	17,981.40	100.00%	1	1	3	0.00213%	14.50%
Tumor tissue	Acyl-protein thioesterase 1 GN=LYPLA1 PE=1 SV=1	A0A087X1K9_HUMAN	17,981.40	98.80%	1	1	3	0.00230%	14.50%
Pap test	Adapter molecule crk GN=CRK PE=1 SV=2	sp|P46108|CRK_HUMAN	33,830.80	100.00%	3	4	4	0.00624%	14.50%
Swab	Adapter molecule crk GN=CRK PE=1 SV=2	sp|P46108|CRK_HUMAN	33,830.80	99.60%	1	1	1	0.00071%	3.62%
Tumor tissue	Adapter molecule crk GN=CRK PE=1 SV=2	sp|P46108|CRK_HUMAN	33,830.80	100.00%	4	4	4	0.00306%	20.40%
Pap test	Adaptin ear-binding coat-associated protein 2 GN=NECAP2 PE=1 SV=1	sp|Q9NVZ3|NECP2_HUMAN	29,464.50	100.00%	3	3	3	0.00468%	26.30%
Swab	Adenine phosphoribosyltransferase GN=APRT PE=1 SV=2	sp|P07741|APT_HUMAN	19,608.50	100.00%	2	2	10	0.00709%	42.80%

Biological sample	Protein name	Protein accession numbers	Protein molecular weight (Da)	Protein identification probability	Exclusive unique peptide count	Exclusive unique spectrum count	Total spectrum count	Percentage of total spectra	Percentage sequence coverage
Tumor tissue	Adenosine deaminase GN=ADA PE=1 SV=3	ADA_HUMAN	40,765.10	100.00%	2	2	4	0.00306%	16.50%
Pap test	Adenosylhomocysteinase GN=AHCY PE=1 SV=4	sp|P23526|SAHH_HUMAN	47,717.10	100.00%	5	5	7	0.01090%	13.70%
Swab	Adenosylhomocysteinase GN=AHCY PE=1 SV=4	sp|P23526|SAHH_HUMAN	47,717.10	100.00%	8	9	16	0.01130%	21.80%
Tumor tissue	Adenosylhomocysteinase GN=AHCY PE=1 SV=4	sp|P23526|SAHH_HUMAN	47,717.10	100.00%	10	12	16	0.01220%	26.40%
Swab	Adenosylhomocysteinase 2 GN=AHCYL1 PE=1 SV=2	sp|O43865|SAHH2_HUMAN	58,951.50	100.00%	1	1	2	0.00142%	5.28%
Tumor tissue	Adenosylhomocysteinase 2 GN=AHCYL1 PE=1 SV=2	sp|O43865|SAHH2_HUMAN	58,951.50	100.00%	3	3	5	0.00383%	10.00%
Pap test	Adenylate kinase 2, mitochondrial GN=AK2 PE=1 SV=1	sp|P54819|KAD2_HUMAN	25,631.80	100.00%	2	3	11	0.01720%	38.40%
Swab	Adenylate kinase 2, mitochondrial GN=AK2 PE=1 SV=1	sp|P54819|KAD2_HUMAN	25,631.80	100.00%	2	2	7	0.00496%	28.00%
Tumor tissue	Adenylate kinase 2, mitochondrial GN=AK2 PE=1 SV=1	sp|P54819|KAD2_HUMAN	25,631.80	100.00%	2	4	21	0.01610%	31.50%
Tumor tissue	Adenylate kinase 4, mitochondrial GN=AK4 PE=1 SV=1	KAD4_HUMAN	25,268.50	100.00%	8	10	11	0.00842%	53.80%
Swab	Adenylate kinase isoenzyme 1 GN=AK1 PE=1 SV=3	KAD1_HUMAN	23,411.10	100.00%	2	2	3	0.00213%	11.90%
Tumor tissue	Adenylate kinase isoenzyme 1 GN=AK1 PE=1 SV=3	KAD1_HUMAN	23,411.10	100.00%	3	4	6	0.00459%	23.20%
Tumor tissue	Adenylosuccinate lyase GN=ADSL PE=1 SV=1	sp|P30566|PUR8_HUMAN	54,434.90	100.00%	5	8	9	0.00689%	18.10%
Swab	Adenylosuccinate synthetase isozyme 2 GN=ADSS PE=1 SV=3	PURA2_HUMAN	50,097.80	100.00%	6	8	10	0.00709%	19.30%
Tumor tissue	Adenylosuccinate synthetase isozyme 2 GN=ADSS PE=1 SV=3	PURA2_HUMAN	50,097.80	100.00%	6	7	7	0.00536%	17.50%
Tumor tissue	Adenylyl cyclase-associated protein 2 GN=CAP2 PE=1 SV=1	sp|P40123|CAP2_HUMAN	52,824.40	100.00%	5	5	6	0.00459%	16.10%
Tumor tissue	Adipocyte enhancer-binding protein 1 GN=AEBP1 PE=1 SV=1	sp|Q8IUX7|AEBP1_HUMAN	130,930.90	100.00%	15	23	37	0.02830%	17.20%

Biological sample	Protein name	Protein accession numbers	Protein molecular weight (Da)	Protein identification probability	Exclusive unique peptide count	Exclusive unique spectrum count	Total spectrum count	Percentage of total spectra	Percentage sequence coverage
Tumor tissue	Adipocyte plasma membrane-associated protein GN=APMAP PE=1 SV=2	sp|Q9HDC9|APMAP_HUMAN	46,481.50	100.00%	2	2	11	0.00842%	21.90%
Swab	Adipogenesis regulatory factor GN=ADIRF PE=1 SV=1	ADIRF_HUMAN	7,854.70	100.00%	2	4	9	0.00638%	59.20%
Tumor tissue	Adipogenesis regulatory factor GN=ADIRF PE=1 SV=1	ADIRF_HUMAN	7,854.70	100.00%	3	3	3	0.00230%	30.30%
Tumor tissue	ADP/ATP translocase 1 GN=SLC25A4 PE=1 SV=4	ADT1_HUMAN	33,065.40	100.00%	3	3	35	0.02680%	32.90%
Pap test	ADP/ATP translocase 2 GN=SLC25A5 PE=1 SV=7	ADT2_HUMAN	32,853.50	100.00%	2	2	6	0.00936%	20.10%
Tumor tissue	ADP/ATP translocase 2 GN=SLC25A5 PE=1 SV=7	ADT2_HUMAN	32,853.50	100.00%	3	4	40	0.03060%	33.20%
Pap test	ADP/ATP translocase 3 GN=SLC25A6 PE=1 SV=4	ADT3_HUMAN	32,867.10	100.00%	1	1	5	0.00780%	16.80%
Tumor tissue	ADP/ATP translocase 3 GN=SLC25A6 PE=1 SV=4	ADT3_HUMAN	32,867.10	100.00%	2	2	39	0.02990%	32.60%
Pap test	ADP-ribosyl cyclase/cyclic ADP-ribose hydrolase 2 GN=BST1 PE=1 SV=1	A6NC48_HUMAN	37,483.20	100.00%	2	2	2	0.00312%	6.61%
Swab	ADP-ribosyl cyclase/cyclic ADP-ribose hydrolase 2 GN=BST1 PE=1 SV=1	A6NC48_HUMAN	37,483.20	100.00%	2	2	3	0.00213%	5.41%
Tumor tissue	ADP-ribosyl cyclase/cyclic ADP-ribose hydrolase 2 GN=BST1 PE=1 SV=1	A6NC48_HUMAN	37,483.20	100.00%	1	2	2	0.00153%	3.30%
Pap test	ADP-ribosylation factor 3 GN=ARF3 PE=1 SV=2	sp|P61204|ARF3_HUMAN	20,601.70	100.00%	2	2	15	0.02340%	43.60%
Swab	ADP-ribosylation factor 3 GN=ARF3 PE=1 SV=2	sp|P61204|ARF3_HUMAN	20,601.70	100.00%	2	2	29	0.02060%	32.00%
Tumor tissue	ADP-ribosylation factor 3 GN=ARF3 PE=1 SV=2	sp|P61204|ARF3_HUMAN	20,601.70	100.00%	4	6	45	0.03440%	52.50%
Pap test	ADP-ribosylation factor 4 GN=ARF4 PE=1 SV=3	ARF4_HUMAN	20,511.60	100.00%	1	1	8	0.01250%	23.30%
Swab	ADP-ribosylation factor 4 GN=ARF4 PE=1 SV=3	ARF4_HUMAN	20,511.60	100.00%	4	4	15	0.01060%	32.20%

Biological sample	Protein name	Protein accession numbers	Protein molecular weight (Da)	Protein identification probability	Exclusive unique peptide count	Exclusive unique spectrum count	Total spectrum count	Percentage of total spectra	Percentage sequence coverage
Tumor tissue	ADP-ribosylation factor 4 GN=ARF4 PE=1 SV=3	ARF4_HUMAN	20,511.60	100.00%	3	6	29	0.02220%	35.60%
Swab	ADP-ribosylation factor 5 GN=ARF5 PE=1 SV=2	ARF5_HUMAN	20,530.10	99.90%	1	1	16	0.01130%	16.10%
Tumor tissue	ADP-ribosylation factor 5 GN=ARF5 PE=1 SV=2	ARF5_HUMAN	20,530.10	100.00%	3	5	26	0.01990%	41.10%
Pap test	ADP-ribosylation factor 6 GN=ARF6 PE=1 SV=2	ARF6_HUMAN	20,083.00	100.00%	2	2	3	0.00468%	18.30%
Swab	ADP-ribosylation factor 6 GN=ARF6 PE=1 SV=2	ARF6_HUMAN	20,083.00	99.30%	1	1	2	0.00142%	12.00%
Tumor tissue	ADP-ribosylation factor 6 GN=ARF6 PE=1 SV=2	ARF6_HUMAN	20,083.00	100.00%	4	6	8	0.00612%	38.90%
Tumor tissue	ADP-ribosylation factor GTPase-activating protein 2 GN=ARFGAP2 PE=1 SV=1	A0A0D9SF70_HUMAN	42,153.50	100.00%	3	4	4	0.00306%	13.50%
Pap test	ADP-ribosylation factor-like protein 1 GN=ARL1 PE=1 SV=1	sp|P40616|ARL1_HUMAN	21,778.40	98.70%	1	1	1	0.00156%	5.67%
Swab	ADP-ribosylation factor-like protein 1 GN=ARL1 PE=1 SV=1	sp|P40616|ARL1_HUMAN	21,778.40	100.00%	3	4	5	0.00354%	12.90%
Tumor tissue	ADP-ribosylation factor-like protein 1 GN=ARL1 PE=1 SV=1	sp|P40616|ARL1_HUMAN	20,418.20	100.00%	4	6	8	0.00612%	17.50%
Tumor tissue	ADP-ribosylation factor-like protein 2 GN=ARL2 PE=1 SV=4	sp|P36404|ARL2_HUMAN	20,879.30	100.00%	4	4	4	0.00306%	22.80%
Swab	ADP-ribosylation factor-like protein 3 GN=ARL3 PE=1 SV=2	ARL3_HUMAN	20,456.30	100.00%	3	4	5	0.00354%	31.90%
Tumor tissue	ADP-ribosylation factor-like protein 3 GN=ARL3 PE=1 SV=2	ARL3_HUMAN	20,456.30	100.00%	5	5	8	0.00612%	43.40%
Tumor tissue	ADP-ribosylation factor-like protein 8A GN=ARL8A PE=1 SV=1	ARL8A_HUMAN	21,416.80	100.00%	2	4	18	0.01380%	47.80%
Pap test	ADP-ribosylation factor-like protein 8B GN=ARL8B PE=1 SV=1	sp|Q9NVJ2|ARL8B_HUMAN	21,540.00	100.00%	2	2	4	0.00624%	22.00%
Tumor tissue	ADP-ribosylation factor-like protein 8B GN=ARL8B PE=1 SV=1	sp|Q9NVJ2|ARL8B_HUMAN	21,540.00	100.00%	2	3	18	0.01380%	40.30%
Pap test	ADP-sugar pyrophosphatase GN=NUDT5 PE=1 SV=1	NUDT5_HUMAN	25,895.90	100.00%	4	4	4	0.00624%	22.40%

Biological sample	Protein name	Protein accession numbers	Protein molecular weight (Da)	Protein identification probability	Exclusive unique peptide count	Exclusive unique spectrum count	Total spectrum count	Percentage of total spectra	Percentage sequence coverage
Swab	ADP-sugar pyrophosphatase GN=NUDT5 PE=1 SV=1	NUDT5_HUMAN	25,895.90	100.00%	2	3	5	0.00354%	12.10%
Tumor tissue	ADP-sugar pyrophosphatase GN=NUDT5 PE=1 SV=1	NUDT5_HUMAN	25,895.90	100.00%	4	6	11	0.00842%	24.10%
Tumor tissue	Afadin GN=AFDN PE=1 SV=1	J3KN01_HUMAN	207,622.00	100.00%	1	1	10	0.00766%	7.32%
Pap test	Afamin GN=AFM PE=1 SV=1	AFAM_HUMAN	69,070.10	100.00%	11	14	22	0.03430%	24.90%
Swab	Afamin GN=AFM PE=1 SV=1	AFAM_HUMAN	69,070.10	100.00%	15	20	36	0.02550%	32.10%
Tumor tissue	Afamin GN=AFM PE=1 SV=1	AFAM_HUMAN	69,070.10	100.00%	5	6	6	0.00459%	10.00%
Tumor tissue	AFG3-like protein 2 GN=AFG3L2 PE=1 SV=2	AFG32_HUMAN	88,586.80	100.00%	4	5	6	0.00459%	5.40%
Swab	Aflatoxin B1 aldehyde reductase member 2 GN=AKR7A2 PE=1 SV=3	ARK72_HUMAN	39,589.00	99.90%	1	1	1	0.00071%	3.34%
Tumor tissue	Aflatoxin B1 aldehyde reductase member 2 GN=AKR7A2 PE=1 SV=3	ARK72_HUMAN	39,589.00	100.00%	4	5	8	0.00612%	19.20%
Tumor tissue	AH receptor-interacting protein GN=AIP PE=1 SV=2	AIP_HUMAN	37,636.40	100.00%	4	4	8	0.00612%	30.90%
Pap test	A-kinase anchor protein 13 GN=AKAP13 PE=1 SV=2	sp|Q12802|AKP13_HUMAN	307,548.60	99.80%	1	1	1	0.00156%	0.71%
Tumor tissue	A-kinase anchor protein 13 GN=AKAP13 PE=1 SV=2	sp|Q12802|AKP13_HUMAN	307,548.60	100.00%	3	3	3	0.00230%	1.64%
Pap test	Alcohol dehydrogenase [NADP(+)] GN=AKR1A1 PE=1 SV=3	AK1A1_HUMAN	36,573.60	100.00%	6	7	12	0.01870%	25.20%
Swab	Alcohol dehydrogenase [NADP(+)] GN=AKR1A1 PE=1 SV=3	AK1A1_HUMAN	36,573.60	100.00%	9	14	32	0.02270%	47.40%
Tumor tissue	Alcohol dehydrogenase [NADP(+)] GN=AKR1A1 PE=1 SV=3	AK1A1_HUMAN	36,573.60	100.00%	10	13	28	0.02140%	41.80%
Tumor tissue	Alcohol dehydrogenase 1B GN=ADH1B PE=1 SV=2	sp|P00325|ADH1B_HUMAN	39,854.30	100.00%	2	5	18	0.01380%	22.90%
Swab	Alcohol dehydrogenase 1C GN=ADH1C PE=1 SV=2	ADH1G_HUMAN	39,867.60	99.70%	1	1	2	0.00142%	4.53%
Pap test	Alcohol dehydrogenase class-3 GN=ADH5 PE=1 SV=4	ADHX_HUMAN	39,723.80	99.90%	1	1	1	0.00156%	2.14%
Swab	Alcohol dehydrogenase class-3 GN=ADH5 PE=1 SV=4	ADHX_HUMAN	39,723.80	100.00%	2	2	3	0.00213%	6.68%

Biological sample	Protein name	Protein accession numbers	Protein molecular weight (Da)	Protein identification probability	Exclusive unique peptide count	Exclusive unique spectrum count	Total spectrum count	Percentage of total spectra	Percentage sequence coverage
Tumor tissue	Alcohol dehydrogenase class-3 GN=ADH5 PE=1 SV=4	ADHX_HUMAN	39,723.80	100.00%	7	11	13	0.00995%	19.00%
Pap test	Aldehyde dehydrogenase family 1 member A3 GN=ALDH1A3 PE=1 SV=2	AL1A3_HUMAN	56,109.30	100.00%	5	6	7	0.01090%	11.90%
Swab	Aldehyde dehydrogenase family 1 member A3 GN=ALDH1A3 PE=1 SV=2	AL1A3_HUMAN	56,109.30	100.00%	4	5	5	0.00354%	12.70%
Tumor tissue	Aldehyde dehydrogenase family 1 member A3 GN=ALDH1A3 PE=1 SV=2	AL1A3_HUMAN	56,109.30	100.00%	5	5	6	0.00459%	15.80%
Swab	Aldehyde dehydrogenase family 16 member A1 GN=ALDH16A1 PE=1 SV=2	sp|Q8IZ83|A16A1_HUMAN	85,126.80	100.00%	4	4	4	0.00283%	5.99%
Tumor tissue	Aldehyde dehydrogenase family 16 member A1 GN=ALDH16A1 PE=1 SV=2	sp|Q8IZ83|A16A1_HUMAN	85,126.80	100.00%	5	6	6	0.00459%	9.48%
Tumor tissue	Aldehyde dehydrogenase X, mitochondrial GN=ALDH1B1 PE=1 SV=3	AL1B1_HUMAN	57,206.60	100.00%	15	23	41	0.03140%	33.70%
Pap test	Aldehyde dehydrogenase, dimeric NADP-preferring GN=ALDH3A1 PE=1 SV=3	AL3A1_HUMAN	44,204.70	100.00%	3	3	4	0.00624%	8.83%
Swab	Aldehyde dehydrogenase, dimeric NADP-preferring GN=ALDH3A1 PE=1 SV=3	AL3A1_HUMAN	44,204.70	100.00%	1	1	2	0.00142%	4.19%
Pap test	Aldehyde dehydrogenase, mitochondrial GN=ALDH2 PE=1 SV=2	sp|P05091|ALDH2_HUMAN	56,381.40	100.00%	1	1	2	0.00312%	5.61%
Swab	Aldehyde dehydrogenase, mitochondrial GN=ALDH2 PE=1 SV=2	sp|P05091|ALDH2_HUMAN	56,381.40	100.00%	3	3	4	0.00283%	7.54%
Tumor tissue	Aldehyde dehydrogenase, mitochondrial GN=ALDH2 PE=1 SV=2	sp|P05091|ALDH2_HUMAN	56,381.40	100.00%	7	8	21	0.01610%	27.30%
Pap test	Aldo-keto reductase family 1 member B10 GN=AKR1B10 PE=1 SV=2	AK1BA_HUMAN	36,020.40	100.00%	5	6	6	0.00936%	18.00%

Biological sample	Protein name	Protein accession numbers	Protein molecular weight (Da)	Protein identification probability	Exclusive unique peptide count	Exclusive unique spectrum count	Total spectrum count	Percentage of total spectra	Percentage sequence coverage
Swab	Aldo-keto reductase family 1 member B10 GN=AKR1B10 PE=1 SV=2	AK1BA_HUMAN	36,020.40	100.00%	4	4	4	0.00283%	16.50%
Pap test	Aldose 1-epimerase GN=GALM PE=1 SV=1	GALM_HUMAN	37,765.20	99.20%	1	1	1	0.00156%	4.09%
Swab	Aldose 1-epimerase GN=GALM PE=1 SV=1	GALM_HUMAN	37,765.20	100.00%	2	2	2	0.00142%	7.02%
Tumor tissue	Aldose 1-epimerase GN=GALM PE=1 SV=1	GALM_HUMAN	37,765.20	100.00%	2	2	2	0.00153%	8.19%
Swab	Aldose reductase GN=AKR1B1 PE=1 SV=3	ALDR_HUMAN	35,853.90	99.10%	1	1	3	0.00213%	5.70%
Tumor tissue	Aldose reductase GN=AKR1B1 PE=1 SV=3	ALDR_HUMAN	35,853.90	100.00%	4	6	17	0.01300%	19.00%
Tumor tissue	Alkaline phosphatase, tissue-nonspecific isozyme GN=ALPL PE=1 SV=4	sp|P05186|PPBT_HUMAN	57,305.00	100.00%	10	11	12	0.00919%	23.70%
Tumor tissue	Alkyldihydroxyacetonephosphate synthase, peroxisomal GN=AGPS PE=1 SV=1	ADAS_HUMAN	72,913.30	100.00%	6	6	6	0.00459%	14.90%
Swab	Allograft inflammatory factor 1 (Fragment) GN=AIF1 PE=1 SV=1	sp|P55008|AIF1_HUMAN	18,039.30	100.00%	3	4	6	0.00425%	27.30%
Tumor tissue	Allograft inflammatory factor 1 (Fragment) GN=AIF1 PE=1 SV=1	sp|P55008|AIF1_HUMAN	18,039.30	100.00%	2	3	3	0.00230%	21.70%
Tumor tissue	All-trans-retinol 13,14-reductase GN=RETSAT PE=1 SV=2	sp|Q6NUM9|RETST_HUMAN	66,821.30	100.00%	2	2	5	0.00383%	7.05%
Tumor tissue	Alpha-(1,6)-fucosyltransferase GN=FUT8 PE=1 SV=2	sp|Q9BYC5|FUT8_HUMAN	66,516.90	100.00%	3	3	4	0.00306%	9.57%
Tumor tissue	Alpha-1,3-mannosyl-glycoprotein 2-beta-N-acetylglucosaminyltransferase GN=MGAT1 PE=1 SV=2	MGAT1_HUMAN	50,879.20	100.00%	2	2	4	0.00306%	12.80%
Pap test	Alpha-1-acid glycoprotein 1 GN=ORM1 PE=1 SV=1	A1AG1_HUMAN	23,512.10	100.00%	6	17	201	0.31400%	41.30%
Swab	Alpha-1-acid glycoprotein 1 GN=ORM1 PE=1 SV=1	A1AG1_HUMAN	23,512.10	100.00%	5	12	107	0.07580%	29.40%

Biological sample	Protein name	Protein accession numbers	Protein molecular weight (Da)	Protein identification probability	Exclusive unique peptide count	Exclusive unique spectrum count	Total spectrum count	Percentage of total spectra	Percentage sequence coverage
Tumor tissue	Alpha-1-acid glycoprotein 1 GN=ORM1 PE=1 SV=1	A1AG1_HUMAN	23,512.10	100.00%	5	7	16	0.01220%	34.30%
Pap test	Alpha-1-acid glycoprotein 2 GN=ORM2 PE=1 SV=2	A1AG2_HUMAN	23,603.30	100.00%	7	13	73	0.11400%	40.30%
Swab	Alpha-1-acid glycoprotein 2 GN=ORM2 PE=1 SV=2	A1AG2_HUMAN	23,603.30	100.00%	6	10	54	0.03830%	35.80%
Tumor tissue	Alpha-1-acid glycoprotein 2 GN=ORM2 PE=1 SV=2	A1AG2_HUMAN	23,603.30	100.00%	2	3	10	0.00766%	17.90%
Pap test	Alpha-1-antichymotrypsin GN=SERPINA3 PE=1 SV=2	sp|P01011|AACT_HUMAN	47,653.00	100.00%	6	12	95	0.14800%	34.30%
Swab	Alpha-1-antichymotrypsin GN=SERPINA3 PE=1 SV=2	sp|P01011|AACT_HUMAN	47,653.00	100.00%	7	13	230	0.16300%	34.30%
Tumor tissue	Alpha-1-antichymotrypsin GN=SERPINA3 PE=1 SV=2	sp|P01011|AACT_HUMAN	47,653.00	100.00%	5	7	24	0.01840%	25.50%
Pap test	Alpha-1-antitrypsin GN=SERPINA1 PE=1 SV=3	sp|P01009|A1AT_HUMAN	46,737.90	100.00%	24	56	517	0.80700%	59.60%
Swab	Alpha-1-antitrypsin GN=SERPINA1 PE=1 SV=3	sp|P01009|A1AT_HUMAN	46,737.90	100.00%	20	49	710	0.50300%	54.30%
Tumor tissue	Alpha-1-antitrypsin GN=SERPINA1 PE=1 SV=3	sp|P01009|A1AT_HUMAN	46,737.90	100.00%	15	26	64	0.04900%	45.90%
Pap test	Alpha-1B-glycoprotein GN=A1BG PE=1 SV=4	sp|P04217|A1BG_HUMAN	54,253.30	100.00%	5	7	63	0.09830%	40.40%
Swab	Alpha-1B-glycoprotein GN=A1BG PE=1 SV=4	sp|P04217|A1BG_HUMAN	54,253.30	100.00%	5	10	122	0.08650%	39.00%
Tumor tissue	Alpha-1B-glycoprotein GN=A1BG PE=1 SV=4	sp|P04217|A1BG_HUMAN	54,253.30	100.00%	3	4	15	0.01150%	24.80%
Pap test	Alpha-2-antiplasmin GN=SERPINF2 PE=1 SV=3	sp|P08697|A2AP_HUMAN	54,566.60	100.00%	7	8	13	0.02030%	18.50%
Swab	Alpha-2-antiplasmin GN=SERPINF2 PE=1 SV=3	sp|P08697|A2AP_HUMAN	54,566.60	100.00%	6	9	15	0.01060%	16.70%
Tumor tissue	Alpha-2-antiplasmin GN=SERPINF2 PE=1 SV=3	sp|P08697|A2AP_HUMAN	54,566.60	100.00%	2	2	2	0.00153%	5.30%
Pap test	Alpha-2-HS-glycoprotein GN=AHSG PE=1 SV=1	FETUA_HUMAN	39,410.50	100.00%	6	10	20	0.03120%	25.00%
Swab	Alpha-2-HS-glycoprotein GN=AHSG PE=1 SV=1	FETUA_HUMAN	39,410.50	100.00%	6	10	35	0.02480%	27.20%

Biological sample	Protein name	Protein accession numbers	Protein molecular weight (Da)	Protein identification probability	Exclusive unique peptide count	Exclusive unique spectrum count	Total spectrum count	Percentage of total spectra	Percentage sequence coverage
Tumor tissue	Alpha-2-HS-glycoprotein GN=AHSG PE=1 SV=1	FETUA_HUMAN	39,410.50	100.00%	2	5	7	0.00536%	8.15%
Pap test	Alpha-2-macroglobulin GN=A2M PE=1 SV=3	A2MG_HUMAN	163,289.90	100.00%	41	71	215	0.33500%	46.70%
Swab	Alpha-2-macroglobulin GN=A2M PE=1 SV=3	A2MG_HUMAN	163,289.90	100.00%	29	46	123	0.08720%	33.40%
Tumor tissue	Alpha-2-macroglobulin GN=A2M PE=1 SV=3	A2MG_HUMAN	163,289.90	100.00%	26	43	80	0.06120%	28.20%
Tumor tissue	Alpha-2-macroglobulin receptor-associated protein GN=LRPAP1 PE=1 SV=1	AMRP_HUMAN	41,467.60	100.00%	7	7	7	0.00536%	22.40%
Pap test	Alpha-2-macroglobulin-like protein 1 GN=A2ML1 PE=1 SV=3	sp|A8K2U0|A2ML1_HUMAN	161,108.20	100.00%	19	24	38	0.05930%	17.10%
Swab	Alpha-2-macroglobulin-like protein 1 GN=A2ML1 PE=1 SV=3	sp|A8K2U0|A2ML1_HUMAN	161,108.20	100.00%	7	8	10	0.00709%	6.60%
Swab	Alpha-actinin-1 GN=ACTN1 PE=1 SV=2	sp|P12814|ACTN1_HUMAN	103,061.10	100.00%	1	3	146	0.10300%	62.40%
Pap test	Alpha-actinin-4 GN=ACTN4 PE=1 SV=2	sp|O43707|ACTN4_HUMAN	104,857.20	100.00%	24	45	229	0.35700%	59.60%
Swab	Alpha-actinin-4 GN=ACTN4 PE=1 SV=2	sp|O43707|ACTN4_HUMAN	104,857.20	100.00%	23	42	150	0.10600%	55.50%
Tumor tissue	Alpha-actinin-4 GN=ACTN4 PE=1 SV=2	sp|O43707|ACTN4_HUMAN	104,857.20	100.00%	26	56	257	0.19700%	59.70%
Tumor tissue	Alpha-adducin GN=ADD1 PE=1 SV=1	sp|P35611|ADDA_HUMAN	70,056.70	100.00%	2	2	11	0.00842%	20.50%
Tumor tissue	Alpha-aminoadipic semialdehyde synthase, mitochondrial GN=AASS PE=1 SV=1	AASS_HUMAN	102,135.40	100.00%	2	2	2	0.00153%	3.46%
Swab	Alpha-centractin GN=ACTR1A PE=1 SV=1	ACTZ_HUMAN	42,615.20	99.60%	1	1	1	0.00071%	5.85%
Tumor tissue	Alpha-centractin GN=ACTR1A PE=1 SV=1	ACTZ_HUMAN	42,615.20	100.00%	5	6	13	0.00995%	27.90%
Pap test	Alpha-crystallin B chain GN=CRYAB PE=1 SV=2	CRYAB_HUMAN	17,925.70	99.20%	1	1	1	0.00156%	7.43%
Tumor tissue	Alpha-crystallin B chain GN=CRYAB PE=1 SV=2	CRYAB_HUMAN	20,031.30	100.00%	3	3	4	0.00306%	18.30%

Biological sample	Protein name	Protein accession numbers	Protein molecular weight (Da)	Protein identification probability	Exclusive unique peptide count	Exclusive unique spectrum count	Total spectrum count	Percentage of total spectra	Percentage sequence coverage
Tumor tissue	Alpha-endosulfine GN=ENSA PE=1 SV=2	sp|O43768|ENSA_HUMAN	20,994.60	100.00%	2	2	3	0.00230%	25.70%
Pap test	Alpha-enolase GN=ENO1 PE=1 SV=2	sp|P06733|ENOA_HUMAN	47,170.20	100.00%	11	18	36	0.05620%	43.80%
Swab	Alpha-enolase GN=ENO1 PE=1 SV=2	sp|P06733|ENOA_HUMAN	47,170.20	100.00%	14	22	82	0.05810%	52.80%
Tumor tissue	Alpha-enolase GN=ENO1 PE=1 SV=2	sp|P06733|ENOA_HUMAN	47,170.20	100.00%	17	43	178	0.13600%	50.70%
Tumor tissue	Alpha-internexin GN=INA PE=1 SV=2	AINX_HUMAN	55,392.30	100.00%	1	1	2	0.00153%	5.01%
Tumor tissue	Alpha-mannosidase 2 GN=MAN2A1 PE=1 SV=2	MA2A1_HUMAN	131,145.70	100.00%	4	4	4	0.00306%	4.20%
Swab	Alpha-N-acetylgalactosaminidase GN=NAGA PE=1 SV=2	NAGAB_HUMAN	46,566.30	100.00%	2	2	2	0.00142%	7.79%
Tumor tissue	Alpha-N-acetylgalactosaminidase GN=NAGA PE=1 SV=2	NAGAB_HUMAN	46,566.30	99.90%	1	2	2	0.00153%	4.14%
Tumor tissue	Alpha-N-acetylglucosaminidase GN=NAGLU PE=1 SV=2	ANAG_HUMAN	82,266.60	100.00%	3	3	3	0.00230%	6.46%
Tumor tissue	Alpha-parvin GN=PARVA PE=1 SV=1	J3KNQ4_HUMAN	46,591.30	100.00%	5	7	16	0.01220%	20.40%
Pap test	Alpha-soluble NSF attachment protein GN=NAPA PE=1 SV=1	M0R0Y2_HUMAN	29,164.60	99.20%	1	1	1	0.00156%	3.91%
Swab	Alpha-soluble NSF attachment protein GN=NAPA PE=1 SV=1	M0R0Y2_HUMAN	29,164.60	100.00%	2	3	6	0.00425%	12.10%
Tumor tissue	Alpha-soluble NSF attachment protein GN=NAPA PE=1 SV=1	M0R0Y2_HUMAN	29,164.60	100.00%	8	10	15	0.01150%	51.20%
Tumor tissue	Alpha-synuclein GN=SNCA PE=1 SV=1	sp|P37840|SYUA_HUMAN	11,775.80	100.00%	2	2	2	0.00153%	32.20%
Tumor tissue	Alpha-taxilin GN=TXLNA PE=1 SV=3	TXLNA_HUMAN	61,891.20	100.00%	5	7	7	0.00536%	16.80%
Tumor tissue	Alternative protein SLC35A4 GN=SLC35A4 PE=1 SV=1	L0R6Q1_HUMAN	11,133.20	100.00%	2	2	3	0.00230%	32.00%
Tumor tissue	Amidophosphoribosyltransferase GN=PPAT PE=1 SV=1	PUR1_HUMAN	57,399.30	100.00%	2	2	2	0.00153%	6.00%
Tumor tissue	Amine oxidase [flavin-containing] B GN=MAOB PE=1 SV=3	sp|P27338|AOFB_HUMAN	58,763.80	100.00%	10	14	17	0.01300%	26.90%

Biological sample	Protein name	Protein accession numbers	Protein molecular weight (Da)	Protein identification probability	Exclusive unique peptide count	Exclusive unique spectrum count	Total spectrum count	Percentage of total spectra	Percentage sequence coverage
Tumor tissue	Aminoacyl tRNA synthase complex-interacting multifunctional protein 2 GN=AIMP2 PE=1 SV=2	AIMP2_HUMAN	35,349.30	100.00%	4	5	9	0.00689%	20.60%
Pap test	Aminopeptidase B GN=RNPEP PE=1 SV=2	AMPB_HUMAN	72,597.40	100.00%	3	3	4	0.00624%	4.92%
Swab	Aminopeptidase B GN=RNPEP PE=1 SV=2	AMPB_HUMAN	72,597.40	100.00%	8	8	14	0.00992%	18.20%
Tumor tissue	Aminopeptidase B GN=RNPEP PE=1 SV=2	AMPB_HUMAN	72,597.40	100.00%	10	12	19	0.01450%	24.00%
Pap test	Aminopeptidase N GN=ANPEP PE=1 SV=4	AMPN_HUMAN	109,542.40	100.00%	2	2	2	0.00312%	2.48%
Swab	Aminopeptidase N GN=ANPEP PE=1 SV=4	AMPN_HUMAN	109,542.40	100.00%	3	4	4	0.00283%	4.24%
Tumor tissue	Aminopeptidase N GN=ANPEP PE=1 SV=4	AMPN_HUMAN	109,542.40	100.00%	3	3	4	0.00306%	4.14%
Tumor tissue	AMP deaminase 2 (Fragment) GN=AMPD2 PE=1 SV=1	H0Y360_HUMAN	98,514.90	100.00%	2	2	2	0.00153%	2.67%
Swab	Anamorsin GN=CIAPIN1 PE=1 SV=1	H3BT65_HUMAN	28,910.50	99.80%	1	1	1	0.00071%	4.51%
Tumor tissue	Anamorsin GN=CIAPIN1 PE=1 SV=1	H3BT65_HUMAN	28,910.50	100.00%	3	3	3	0.00230%	9.77%
Tumor tissue	Anaphase-promoting complex subunit 1 GN=ANAPC1 PE=1 SV=1	APC1_HUMAN	216,503.30	100.00%	3	3	3	0.00230%	1.70%
Tumor tissue	Anaphase-promoting complex subunit 7 GN=ANAPC7 PE=1 SV=4	sp|Q9UJX3|APC7_HUMAN	66,857.80	100.00%	2	2	2	0.00153%	4.17%
Tumor tissue	Anaphase-promoting complex subunit CDC26 GN=CDC26 PE=1 SV=1	CDC26_HUMAN	9,777.50	100.00%	2	2	2	0.00153%	42.40%
Tumor tissue	Angiopoietin-related protein 2 GN=ANGPTL2 PE=2 SV=1	sp|Q9UKU9|ANGL2_HUMAN	57,104.90	100.00%	3	3	4	0.00306%	11.00%
Pap test	Angiotensinogen GN=AGT PE=1 SV=1	ANGT_HUMAN	53,154.80	100.00%	8	10	19	0.02960%	21.40%
Swab	Angiotensinogen GN=AGT PE=1 SV=1	ANGT_HUMAN	53,154.80	100.00%	5	7	14	0.00992%	12.60%
Tumor tissue	Angiotensinogen GN=AGT PE=1 SV=1	ANGT_HUMAN	53,154.80	100.00%	3	4	5	0.00383%	9.07%

Biological sample	Protein name	Protein accession numbers	Protein molecular weight (Da)	Protein identification probability	Exclusive unique peptide count	Exclusive unique spectrum count	Total spectrum count	Percentage of total spectra	Percentage sequence coverage
Tumor tissue	Ankyrin repeat and LEM domain-containing protein 2 GN=ANKLE2 PE=1 SV=4	sp|Q86XL3|ANKL2_HUMAN	104,116.80	100.00%	3	3	3	0.00230%	5.65%
Tumor tissue	Ankyrin repeat domain-containing protein 17 (Fragment) GN=ANKRD17 PE=1 SV=1	sp|O75179|ANR17_HUMAN	263,364.40	100.00%	1	1	2	0.00153%	1.17%
Tumor tissue	Ankyrin-3 GN=ANK3 PE=1 SV=3	sp|Q12955|ANK3_HUMAN	480,407.20	99.70%	1	1	2	0.00153%	0.50%
Tumor tissue	Annexin GN=ANXA6 PE=1 SV=1	E5RK69_HUMAN	51,779.70	99.90%	1	1	62	0.04750%	49.30%
Pap test	Annexin A1 GN=ANXA1 PE=1 SV=2	ANXA1_HUMAN	38,715.90	100.00%	15	22	53	0.08270%	51.20%
Swab	Annexin A1 GN=ANXA1 PE=1 SV=2	ANXA1_HUMAN	38,715.90	100.00%	9	13	25	0.01770%	29.20%
Tumor tissue	Annexin A1 GN=ANXA1 PE=1 SV=2	ANXA1_HUMAN	38,715.90	100.00%	16	30	66	0.05050%	49.70%
Pap test	Annexin A3 GN=ANXA3 PE=1 SV=3	ANXA3_HUMAN	36,377.40	100.00%	11	11	27	0.04210%	45.50%
Swab	Annexin A3 GN=ANXA3 PE=1 SV=3	ANXA3_HUMAN	36,377.40	100.00%	8	9	23	0.01630%	39.00%
Tumor tissue	Annexin A3 GN=ANXA3 PE=1 SV=3	ANXA3_HUMAN	36,377.40	100.00%	4	5	10	0.00766%	25.10%
Pap test	Annexin A4 GN=ANXA4 PE=1 SV=4	sp|P09525|ANXA4_HUMAN	35,884.30	100.00%	7	8	9	0.01400%	26.60%
Swab	Annexin A4 GN=ANXA4 PE=1 SV=4	sp|P09525|ANXA4_HUMAN	35,884.30	100.00%	7	7	10	0.00709%	30.40%
Tumor tissue	Annexin A4 GN=ANXA4 PE=1 SV=4	sp|P09525|ANXA4_HUMAN	35,884.30	100.00%	13	20	30	0.02300%	42.30%
Pap test	Annexin A5 GN=ANXA5 PE=1 SV=2	ANXA5_HUMAN	35,938.60	100.00%	4	5	13	0.02030%	22.20%
Swab	Annexin A5 GN=ANXA5 PE=1 SV=2	ANXA5_HUMAN	35,938.60	100.00%	3	3	17	0.01200%	34.70%
Tumor tissue	Annexin A5 GN=ANXA5 PE=1 SV=2	ANXA5_HUMAN	35,938.60	100.00%	10	15	75	0.05740%	50.00%
Pap test	Annexin A6 GN=ANXA6 PE=1 SV=3	sp|P08133|ANXA6_HUMAN	75,877.50	100.00%	2	2	5	0.00780%	5.05%
Swab	Annexin A6 GN=ANXA6 PE=1 SV=3	sp|P08133|ANXA6_HUMAN	75,877.50	100.00%	2	2	5	0.00354%	9.96%
Tumor tissue	Annexin A6 GN=ANXA6 PE=1 SV=3	sp|P08133|ANXA6_HUMAN	75,877.50	100.00%	13	18	89	0.06810%	51.70%
Pap test	Anterior gradient protein 2 homolog GN=AGR2 PE=1 SV=1	AGR2_HUMAN	19,980.30	100.00%	1	1	3	0.00468%	16.00%
Swab	Anterior gradient protein 2 homolog GN=AGR2 PE=1 SV=1	AGR2_HUMAN	19,980.30	100.00%	3	3	7	0.00496%	22.90%
Tumor tissue	Antigen peptide transporter 1 GN=TAP1 PE=1 SV=1	TAP1_HUMAN	87,177.40	100.00%	7	7	8	0.00612%	16.30%

Biological sample	Protein name	Protein accession numbers	Protein molecular weight (Da)	Protein identification probability	Exclusive unique peptide count	Exclusive unique spectrum count	Total spectrum count	Percentage of total spectra	Percentage sequence coverage
Pap test	Antileukoproteinase GN=SLPI PE=1 SV=2	SLPI_HUMAN	14,326.00	100.00%	3	4	7	0.01090%	25.80%
Swab	Antileukoproteinase GN=SLPI PE=1 SV=2	SLPI_HUMAN	14,326.00	100.00%	4	4	10	0.00709%	31.80%
Pap test	Antithrombin-III GN=SERPINC1 PE=1 SV=1	ANT3_HUMAN	52,604.10	100.00%	14	28	80	0.12500%	39.20%
Swab	Antithrombin-III GN=SERPINC1 PE=1 SV=1	ANT3_HUMAN	52,604.10	100.00%	15	24	116	0.08220%	36.40%
Tumor tissue	Antithrombin-III GN=SERPINC1 PE=1 SV=1	ANT3_HUMAN	52,604.10	100.00%	8	11	15	0.01150%	25.00%
Tumor tissue	AP-2 complex subunit mu GN=AP2M1 PE=1 SV=2	sp|Q96CW1|AP2M1_HUMAN	49,655.70	100.00%	6	7	9	0.00689%	14.00%
Tumor tissue	AP-2 complex subunit sigma GN=AP2S1 PE=1 SV=1	sp|P53680|AP2S1_HUMAN	18,929.40	100.00%	2	2	2	0.00153%	13.30%
Tumor tissue	AP2-associated protein kinase 1 (Fragment) GN=AAK1 PE=1 SV=1	A0A096LP25_HUMAN	54,448.80	100.00%	3	3	3	0.00230%	10.80%
Swab	AP-3 complex subunit beta-1 GN=AP3B1 PE=1 SV=3	sp|O00203|AP3B1_HUMAN	121,324.00	100.00%	1	1	1	0.00071%	1.74%
Tumor tissue	AP-3 complex subunit beta-1 GN=AP3B1 PE=1 SV=3	sp|O00203|AP3B1_HUMAN	121,324.00	100.00%	9	9	14	0.01070%	10.70%
Tumor tissue	AP-3 complex subunit mu-1 GN=AP3M1 PE=1 SV=1	AP3M1_HUMAN	46,940.00	100.00%	6	7	10	0.00766%	27.50%
Tumor tissue	AP-5 complex subunit beta-1 GN=AP5B1 PE=1 SV=4	AP5B1_HUMAN	93,952.50	100.00%	2	2	4	0.00306%	3.19%
Pap test	Apolipoprotein A-I GN=APOA1 PE=1 SV=1	APOA1_HUMAN	30,778.50	100.00%	14	19	87	0.13600%	52.80%
Swab	Apolipoprotein A-I GN=APOA1 PE=1 SV=1	APOA1_HUMAN	30,778.50	100.00%	21	33	131	0.09280%	73.00%
Tumor tissue	Apolipoprotein A-I GN=APOA1 PE=1 SV=1	APOA1_HUMAN	30,778.50	100.00%	20	29	59	0.04520%	69.70%
Pap test	Apolipoprotein A-II GN=APOA2 PE=1 SV=1	V9GYM3_HUMAN	14,914.40	100.00%	3	5	7	0.01090%	30.80%
Swab	Apolipoprotein A-II GN=APOA2 PE=1 SV=1	V9GYM3_HUMAN	14,914.40	100.00%	2	3	6	0.00425%	15.80%
Tumor tissue	Apolipoprotein A-II GN=APOA2 PE=1 SV=1	V9GYM3_HUMAN	14,914.40	100.00%	2	3	3	0.00230%	15.80%

Biological sample	Protein name	Protein accession numbers	Protein molecular weight (Da)	Protein identification probability	Exclusive unique peptide count	Exclusive unique spectrum count	Total spectrum count	Percentage of total spectra	Percentage sequence coverage
Pap test	Apolipoprotein A-IV GN=APOA4 PE=1 SV=3	APOA4_HUMAN	45,399.40	100.00%	8	8	8	0.01250%	22.20%
Swab	Apolipoprotein A-IV GN=APOA4 PE=1 SV=3	APOA4_HUMAN	45,399.40	100.00%	9	10	13	0.00921%	24.00%
Tumor tissue	Apolipoprotein A-IV GN=APOA4 PE=1 SV=3	APOA4_HUMAN	45,399.40	100.00%	9	9	9	0.00689%	26.80%
Pap test	Apolipoprotein B-100 GN=APOB PE=1 SV=2	APOB_HUMAN	515,614.80	100.00%	8	8	9	0.01400%	2.06%
Swab	Apolipoprotein B-100 GN=APOB PE=1 SV=2	APOB_HUMAN	515,614.80	100.00%	7	7	7	0.00496%	1.86%
Tumor tissue	Apolipoprotein B-100 GN=APOB PE=1 SV=2	APOB_HUMAN	515,614.80	100.00%	91	108	125	0.09570%	27.10%
Swab	Apolipoprotein C-I GN=APOC1 PE=1 SV=1	APOC1_HUMAN	8,647.40	100.00%	2	2	2	0.00142%	24.10%
Tumor tissue	Apolipoprotein C-I GN=APOC1 PE=1 SV=1	APOC1_HUMAN	8,647.40	99.90%	1	1	1	0.00077%	13.30%
Pap test	Apolipoprotein C-III GN=APOC3 PE=1 SV=1	APOC3_HUMAN	12,815.60	99.20%	1	1	1	0.00156%	16.20%
Swab	Apolipoprotein C-III GN=APOC3 PE=1 SV=1	APOC3_HUMAN	12,815.60	99.60%	1	2	2	0.00142%	16.20%
Tumor tissue	Apolipoprotein C-III GN=APOC3 PE=1 SV=1	APOC3_HUMAN	12,815.60	100.00%	2	3	4	0.00306%	27.30%
Pap test	Apolipoprotein D GN=APOD PE=1 SV=1	APOD_HUMAN	24,158.60	100.00%	5	9	26	0.04060%	30.70%
Swab	Apolipoprotein D GN=APOD PE=1 SV=1	APOD_HUMAN	24,158.60	100.00%	6	7	20	0.01420%	31.20%
Tumor tissue	Apolipoprotein D GN=APOD PE=1 SV=1	APOD_HUMAN	24,158.60	100.00%	2	2	3	0.00230%	11.60%
Pap test	Apolipoprotein E GN=APOE PE=1 SV=1	APOE_HUMAN	36,153.50	100.00%	4	4	4	0.00624%	16.40%
Swab	Apolipoprotein E GN=APOE PE=1 SV=1	APOE_HUMAN	36,153.50	100.00%	8	11	14	0.00992%	33.10%
Tumor tissue	Apolipoprotein E GN=APOE PE=1 SV=1	APOE_HUMAN	36,153.50	100.00%	9	10	12	0.00919%	33.80%
Tumor tissue	Apolipoprotein L2 GN=APOL2 PE=1 SV=2	J3KQL8_HUMAN	48,915.40	100.00%	7	9	12	0.00919%	17.80%

Biological sample	Protein name	Protein accession numbers	Protein molecular weight (Da)	Protein identification probability	Exclusive unique peptide count	Exclusive unique spectrum count	Total spectrum count	Percentage of total spectra	Percentage sequence coverage
Pap test	Apolipoprotein(a) GN=LPA PE=1 SV=1	APOA_HUMAN	501,288.90	100.00%	2	2	2	0.00312%	0.51%
Tumor tissue	Apoptosis-inducing factor 1, mitochondrial GN=AIFM1 PE=1 SV=1	sp|O95831|AIFM1_HUMAN	66,901.50	100.00%	15	20	30	0.02300%	30.50%
Tumor tissue	Apoptotic chromatin condensation inducer in the nucleus GN=ACIN1 PE=1 SV=2	sp|Q9UKV3|ACINU_HUMAN	145,442.50	100.00%	4	4	4	0.00306%	4.92%
Pap test	Arachidonate 12-lipoxygenase, 12S-type GN=ALOX12 PE=1 SV=4	LOX12_HUMAN	75,695.80	100.00%	4	4	8	0.01250%	13.30%
Tumor tissue	Arachidonate 5-lipoxygenase GN=ALOX5 PE=1 SV=2	sp|P09917|LOX5_HUMAN	77,984.80	100.00%	3	5	6	0.00459%	6.23%
Tumor tissue	ARAF protein GN=ARAF PE=1 SV=1	Q96II5_HUMAN	67,926.00	100.00%	1	1	2	0.00153%	3.45%
Pap test	Archain 1, isoform CRA_a GN=ARCN1 PE=1 SV=1	sp|P48444|COPD_HUMAN	61,627.40	100.00%	2	2	3	0.00468%	5.98%
Swab	Archain 1, isoform CRA_a GN=ARCN1 PE=1 SV=1	sp|P48444|COPD_HUMAN	61,627.40	100.00%	3	3	5	0.00354%	6.52%
Tumor tissue	Archain 1, isoform CRA_a GN=ARCN1 PE=1 SV=1	sp|P48444|COPD_HUMAN	61,627.40	100.00%	11	13	24	0.01840%	27.40%
Tumor tissue	Arfaptin-1 GN=ARFIP1 PE=1 SV=2	sp|P53367|ARFP1_HUMAN	41,739.50	100.00%	7	9	9	0.00689%	30.30%
Tumor tissue	Arf-GAP with coiled-coil, ANK repeat and PH domain-containing protein 2 GN=ACAP2 PE=1 SV=1	ACAP2_HUMAN	88,022.40	100.00%	3	3	3	0.00230%	5.02%
Tumor tissue	Arginine--tRNA ligase, cytoplasmic GN=RARS PE=1 SV=2	sp|P54136|SYRC_HUMAN	75,382.00	100.00%	13	16	16	0.01220%	23.50%
Pap test	Argininosuccinate synthase GN=ASS1 PE=1 SV=2	ASSY_HUMAN	46,531.30	100.00%	4	4	4	0.00624%	13.80%
Swab	Argininosuccinate synthase GN=ASS1 PE=1 SV=2	ASSY_HUMAN	46,531.30	100.00%	13	16	27	0.01910%	32.50%
Tumor tissue	Argininosuccinate synthase GN=ASS1 PE=1 SV=2	ASSY_HUMAN	46,531.30	100.00%	10	15	19	0.01450%	25.20%
Tumor tissue	Armadillo repeat protein deleted in velo-cardio-facial syndrome GN=ARVCF PE=1 SV=1	sp|O00192|ARVC_HUMAN	103,785.50	100.00%	4	4	6	0.00459%	4.93%

Biological sample	Protein name	Protein accession numbers	Protein molecular weight (Da)	Protein identification probability	Exclusive unique peptide count	Exclusive unique spectrum count	Total spectrum count	Percentage of total spectra	Percentage sequence coverage
Tumor tissue	Armadillo repeat-containing protein 1 GN=ARMC1 PE=1 SV=1	sp|Q9NVT9|ARMC1_HUMAN	31,281.00	100.00%	2	2	2	0.00153%	10.60%
Tumor tissue	Armadillo repeat-containing X-linked protein 3 GN=ARMCX3 PE=1 SV=1	ARMX3_HUMAN	42,501.80	100.00%	3	3	3	0.00230%	11.30%
Tumor tissue	Arylamine N-acetyltransferase 1 GN=NAT1 PE=1 SV=1	F5H5R8_HUMAN	40,847.30	100.00%	2	2	2	0.00153%	8.81%
Pap test	Arylsulfatase A GN=ARSA PE=1 SV=1	A0A0C4DFZ2_HUMAN	53,807.50	100.00%	2	2	2	0.00312%	8.06%
Swab	Arylsulfatase A GN=ARSA PE=1 SV=1	A0A0C4DFZ2_HUMAN	53,807.50	99.90%	1	2	3	0.00213%	3.34%
Tumor tissue	Arylsulfatase A GN=ARSA PE=1 SV=1	A0A0C4DFZ2_HUMAN	53,807.50	98.40%	1	1	2	0.00153%	3.34%
Pap test	Asparagine--tRNA ligase, cytoplasmic GN=NARS PE=1 SV=1	sp|O43776|SYNC_HUMAN	62,944.30	99.90%	1	1	1	0.00156%	2.01%
Tumor tissue	Asparagine--tRNA ligase, cytoplasmic GN=NARS PE=1 SV=1	sp|O43776|SYNC_HUMAN	62,944.30	100.00%	7	7	8	0.00612%	15.10%
Pap test	Aspartate aminotransferase, cytoplasmic GN=GOT1 PE=1 SV=3	sp|P17174|AATC_HUMAN	46,248.10	100.00%	13	15	31	0.04840%	43.60%
Swab	Aspartate aminotransferase, cytoplasmic GN=GOT1 PE=1 SV=3	sp|P17174|AATC_HUMAN	46,248.10	100.00%	8	9	15	0.01060%	26.90%
Tumor tissue	Aspartate aminotransferase, cytoplasmic GN=GOT1 PE=1 SV=3	sp|P17174|AATC_HUMAN	46,248.10	100.00%	10	11	14	0.01070%	35.40%
Pap test	Aspartate aminotransferase, mitochondrial GN=GOT2 PE=1 SV=3	sp|P00505|AATM_HUMAN	47,518.60	100.00%	15	22	37	0.05770%	39.50%
Swab	Aspartate aminotransferase, mitochondrial GN=GOT2 PE=1 SV=3	sp|P00505|AATM_HUMAN	47,518.60	100.00%	7	8	11	0.00780%	19.10%
Tumor tissue	Aspartate aminotransferase, mitochondrial GN=GOT2 PE=1 SV=3	sp|P00505|AATM_HUMAN	47,518.60	100.00%	10	12	22	0.01680%	25.30%
Pap test	Aspartate--tRNA ligase, cytoplasmic GN=DARS PE=1 SV=2	sp|P14868|SYDC_HUMAN	57,136.70	100.00%	1	1	1	0.00156%	2.00%
Swab	Aspartate--tRNA ligase, cytoplasmic GN=DARS PE=1 SV=2	sp|P14868|SYDC_HUMAN	57,136.70	100.00%	1	1	2	0.00142%	6.19%
Tumor tissue	Aspartate--tRNA ligase, cytoplasmic GN=DARS PE=1 SV=2	sp|P14868|SYDC_HUMAN	57,136.70	100.00%	5	6	28	0.02140%	40.10%
Tumor tissue	Aspartate--tRNA ligase, mitochondrial GN=DARS2 PE=1 SV=1	SYDM_HUMAN	73,565.00	100.00%	5	6	6	0.00459%	13.80%

Biological sample	Protein name	Protein accession numbers	Protein molecular weight (Da)	Protein identification probability	Exclusive unique peptide count	Exclusive unique spectrum count	Total spectrum count	Percentage of total spectra	Percentage sequence coverage
Tumor tissue	Aspartyl aminopeptidase GN=DNPEP PE=1 SV=1	DNPEP_HUMAN	52,428.60	100.00%	4	5	9	0.00689%	16.00%
Tumor tissue	Aspartyl/asparaginyl beta-hydroxylase GN=ASPH PE=1 SV=3	sp|Q12797|ASPH_HUMAN	85,862.60	100.00%	11	15	18	0.01380%	20.40%
Tumor tissue	Asporin GN=ASPN PE=1 SV=2	ASPN_HUMAN	43,419.80	100.00%	5	6	7	0.00536%	18.20%
Tumor tissue	Astrocytic phosphoprotein PEA-15 GN=PEA15 PE=1 SV=1	B1AKZ5_HUMAN	12,531.40	99.60%	1	2	7	0.00536%	32.40%
Swab	Ataxin-10 GN=ATXN10 PE=1 SV=1	sp|Q9UBB4|ATX10_HUMAN	53,490.80	100.00%	1	1	1	0.00071%	3.58%
Tumor tissue	Ataxin-10 GN=ATXN10 PE=1 SV=1	sp|Q9UBB4|ATX10_HUMAN	53,490.80	100.00%	6	7	8	0.00612%	18.90%
Tumor tissue	Atlastin-3 GN=ATL3 PE=1 SV=1	ATLA3_HUMAN	60,543.20	100.00%	11	16	20	0.01530%	32.90%
Pap test	ATP synthase F(0) complex subunit B1, mitochondrial GN=ATP5F1 PE=1 SV=2	AT5F1_HUMAN	28,908.80	100.00%	1	1	1	0.00156%	4.69%
Tumor tissue	ATP synthase F(0) complex subunit B1, mitochondrial GN=ATP5F1 PE=1 SV=2	AT5F1_HUMAN	22,275.10	100.00%	8	12	14	0.01070%	30.90%
Pap test	ATP synthase subunit alpha, mitochondrial GN=ATP5A1 PE=1 SV=1	sp|P25705|ATPA_HUMAN	59,752.10	100.00%	5	5	10	0.01560%	12.50%
Tumor tissue	ATP synthase subunit alpha, mitochondrial GN=ATP5A1 PE=1 SV=1	sp|P25705|ATPA_HUMAN	59,752.10	100.00%	19	34	71	0.05440%	40.70%
Pap test	ATP synthase subunit beta, mitochondrial GN=ATP5B PE=1 SV=3	ATPB_HUMAN	56,560.60	100.00%	7	12	37	0.05770%	41.00%
Tumor tissue	ATP synthase subunit beta, mitochondrial GN=ATP5B PE=1 SV=3	ATPB_HUMAN	56,560.60	100.00%	7	18	116	0.08880%	46.50%
Pap test	ATP synthase subunit d, mitochondrial GN=ATP5H PE=1 SV=3	sp|O75947|ATP5H_HUMAN	18,491.90	100.00%	2	2	2	0.00312%	18.00%

Biological sample	Protein name	Protein accession numbers	Protein molecular weight (Da)	Protein identification probability	Exclusive unique peptide count	Exclusive unique spectrum count	Total spectrum count	Percentage of total spectra	Percentage sequence coverage
Tumor tissue	ATP synthase subunit d, mitochondrial GN=ATP5H PE=1 SV=3	sp|O75947|ATP5H_HUMAN	18,491.90	100.00%	6	7	8	0.00612%	37.30%
Tumor tissue	ATP synthase subunit e, mitochondrial GN=ATP5I PE=1 SV=2	ATP5I_HUMAN	7,933.70	100.00%	2	2	4	0.00306%	37.70%
Pap test	ATP synthase subunit g, mitochondrial GN=ATP5L PE=1 SV=3	ATP5L_HUMAN	11,428.50	99.20%	1	1	1	0.00156%	10.70%
Tumor tissue	ATP synthase subunit g, mitochondrial GN=ATP5L PE=1 SV=3	ATP5L_HUMAN	11,428.50	100.00%	5	7	11	0.00842%	46.60%
Pap test	ATP synthase subunit gamma, mitochondrial GN=ATP5C1 PE=1 SV=1	sp|P36542|ATPG_HUMAN	32,998.00	100.00%	3	3	3	0.00468%	11.40%
Tumor tissue	ATP synthase subunit gamma, mitochondrial GN=ATP5C1 PE=1 SV=1	sp|P36542|ATPG_HUMAN	32,998.00	100.00%	5	6	8	0.00612%	19.80%
Pap test	ATP synthase subunit O, mitochondrial GN=ATP5O PE=1 SV=1	ATPO_HUMAN	23,277.40	100.00%	4	5	5	0.00780%	27.70%
Tumor tissue	ATP synthase subunit O, mitochondrial GN=ATP5O PE=1 SV=1	ATPO_HUMAN	23,277.40	100.00%	8	12	27	0.02070%	47.90%
Pap test	ATPase ASNA1 GN=ASNA1 PE=1 SV=1	A0A087WXS7_HUMAN	37,120.00	99.20%	1	1	1	0.00156%	3.02%
Swab	ATPase ASNA1 GN=ASNA1 PE=1 SV=1	A0A087WXS7_HUMAN	37,120.00	100.00%	2	2	3	0.00213%	5.74%
Tumor tissue	ATPase ASNA1 GN=ASNA1 PE=1 SV=1	A0A087WXS7_HUMAN	37,120.00	100.00%	5	5	7	0.00536%	24.50%
Tumor tissue	ATPase family AAA domain-containing protein 1 GN=ATAD1 PE=1 SV=1	sp|Q8NBU5|ATAD1_HUMAN	40,745.10	100.00%	5	6	7	0.00536%	23.50%
Tumor tissue	ATPase family AAA domain-containing protein 2 GN=ATAD2 PE=1 SV=1	A0A0B4J211_HUMAN	80,610.10	100.00%	2	2	2	0.00153%	5.08%

Biological sample	Protein name	Protein accession numbers	Protein molecular weight (Da)	Protein identification probability	Exclusive unique peptide count	Exclusive unique spectrum count	Total spectrum count	Percentage of total spectra	Percentage sequence coverage
Tumor tissue	ATP-binding cassette sub-family B member 7, mitochondrial GN=ABCB7 PE=1 SV=1	sp|O75027|ABCB7_HUMAN	77,122.80	100.00%	2	3	3	0.00230%	4.77%
Tumor tissue	ATP-binding cassette sub-family D member 3 GN=ABCD3 PE=1 SV=1	sp|P28288|ABCD3_HUMAN	75,479.20	100.00%	3	3	3	0.00230%	6.37%
Tumor tissue	ATP-binding cassette sub-family E member 1 GN=ABCE1 PE=1 SV=1	ABCE1_HUMAN	67,316.40	100.00%	6	7	10	0.00766%	14.00%
Tumor tissue	ATP-binding cassette sub-family F member 1 GN=ABCF1 PE=1 SV=2	sp|Q8NE71|ABCF1_HUMAN	95,928.20	100.00%	16	17	17	0.01300%	24.30%
Tumor tissue	ATP-dependent (S)-NAD(P)H-hydrate dehydratase GN=NAXD PE=1 SV=1	sp|Q8IW45|NNRD_HUMAN	36,575.60	100.00%	3	4	4	0.00306%	15.30%
Swab	ATP-dependent 6-phosphofructokinase, platelet type GN=PFKP PE=1 SV=2	sp|Q01813|PFKAP_HUMAN	85,597.40	100.00%	1	1	5	0.00354%	7.65%
Tumor tissue	ATP-dependent 6-phosphofructokinase, platelet type GN=PFKP PE=1 SV=2	sp|Q01813|PFKAP_HUMAN	85,597.40	100.00%	2	2	29	0.02220%	25.40%
Tumor tissue	ATP-dependent Clp protease ATP-binding subunit clpX-like, mitochondrial GN=CLPX PE=1 SV=2	CLPX_HUMAN	69,224.70	100.00%	2	2	2	0.00153%	3.63%
Tumor tissue	ATP-dependent Clp protease proteolytic subunit, mitochondrial GN=CLPP PE=1 SV=1	CLPP_HUMAN	30,180.50	100.00%	5	6	7	0.00536%	26.00%
Tumor tissue	ATP-dependent DNA helicase Q1 GN=RECQL PE=1 SV=3	RECQ1_HUMAN	73,458.90	100.00%	12	17	23	0.01760%	24.70%
Pap test	ATP-dependent RNA helicase A GN=DHX9 PE=1 SV=4	sp|Q08211|DHX9_HUMAN	140,961.50	100.00%	3	3	3	0.00468%	3.07%
Swab	ATP-dependent RNA helicase A GN=DHX9 PE=1 SV=4	sp|Q08211|DHX9_HUMAN	140,961.50	100.00%	2	2	2	0.00142%	2.68%
Tumor tissue	ATP-dependent RNA helicase A GN=DHX9 PE=1 SV=4	sp|Q08211|DHX9_HUMAN	140,961.50	100.00%	26	38	54	0.04130%	25.80%
Tumor tissue	ATP-dependent RNA helicase DDX1 GN=DDX1 PE=1 SV=2	sp|Q92499|DDX1_HUMAN	82,433.20	100.00%	15	25	29	0.02220%	24.90%
Swab	ATP-dependent RNA helicase DDX18 GN=DDX18 PE=1 SV=2	DDX18_HUMAN	75,409.70	99.70%	1	1	1	0.00071%	2.09%

Biological sample	Protein name	Protein accession numbers	Protein molecular weight (Da)	Protein identification probability	Exclusive unique peptide count	Exclusive unique spectrum count	Total spectrum count	Percentage of total spectra	Percentage sequence coverage
Tumor tissue	ATP-dependent RNA helicase DDX18 GN=DDX18 PE=1 SV=2	DDX18_HUMAN	75,409.70	100.00%	2	2	5	0.00383%	8.66%
Pap test	ATP-dependent RNA helicase DDX3X GN=DDX3X PE=1 SV=1	A0A0D9SG12_HUMAN	71,542.90	99.20%	1	1	1	0.00156%	1.85%
Tumor tissue	ATP-dependent RNA helicase DDX3X GN=DDX3X PE=1 SV=1	A0A0D9SG12_HUMAN	71,542.90	100.00%	4	5	32	0.02450%	28.60%
Tumor tissue	ATP-dependent RNA helicase DDX42 GN=DDX42 PE=1 SV=1	sp|Q86XP3|DDX42_HUMAN	102,976.90	100.00%	1	1	7	0.00536%	10.60%
Tumor tissue	ATP-dependent RNA helicase DDX50 GN=DDX50 PE=1 SV=1	DDX50_HUMAN	82,566.70	100.00%	5	5	7	0.00536%	12.50%
Tumor tissue	ATP-dependent RNA helicase DHX29 GN=DHX29 PE=1 SV=1	DHX29_HUMAN	155,296.30	100.00%	3	3	3	0.00230%	2.99%
Tumor tissue	ATP-dependent RNA helicase DHX36 GN=DHX36 PE=1 SV=2	sp|Q9H2U1|DHX36_HUMAN	114,763.80	100.00%	6	6	6	0.00459%	10.70%
Swab	AT-rich interactive domain-containing protein 1A GN=ARID1A PE=1 SV=3	sp|O14497|ARI1A_HUMAN	242,043.80	100.00%	1	1	1	0.00071%	1.27%
Tumor tissue	AT-rich interactive domain-containing protein 1A GN=ARID1A PE=1 SV=3	sp|O14497|ARI1A_HUMAN	242,043.80	100.00%	3	3	3	0.00230%	1.49%
Tumor tissue	Autophagy protein 5 GN=ATG5 PE=1 SV=2	sp|Q9H1Y0|ATG5_HUMAN	32,448.40	100.00%	2	3	3	0.00230%	10.90%
Tumor tissue	Autophagy-related protein 101 GN=ATG101 PE=1 SV=1	ATGA1_HUMAN	21,925.40	100.00%	2	2	2	0.00153%	12.40%
Tumor tissue	Axin interactor, dorsalization-associated protein GN=AIDA PE=1 SV=1	sp|Q96BJ3|AIDA_HUMAN	35,024.60	100.00%	4	5	6	0.00459%	19.00%
Pap test	Azurocidin GN=AZU1 PE=1 SV=3	CAP7_HUMAN	26,885.60	100.00%	3	7	25	0.03900%	25.90%
Swab	Azurocidin GN=AZU1 PE=1 SV=3	CAP7_HUMAN	26,885.60	100.00%	3	5	21	0.01490%	37.50%
Pap test	Bactericidal permeability-increasing protein GN=BPI PE=1 SV=4	BPI_HUMAN	53,900.80	100.00%	2	2	3	0.00468%	4.11%
Swab	Bactericidal permeability-increasing protein GN=BPI PE=1 SV=4	BPI_HUMAN	53,900.80	99.90%	1	1	2	0.00142%	1.85%

Biological sample	Protein name	Protein accession numbers	Protein molecular weight (Da)	Protein identification probability	Exclusive unique peptide count	Exclusive unique spectrum count	Total spectrum count	Percentage of total spectra	Percentage sequence coverage
Tumor tissue	Baculoviral IAP repeat-containing protein 6 GN=BIRC6 PE=1 SV=2	BIRC6_HUMAN	530,254.90	100.00%	5	5	5	0.00383%	1.48%
Tumor tissue	BAG family molecular chaperone regulator 2 GN=BAG2 PE=1 SV=1	sp|O95816|BAG2_HUMAN	23,772.50	100.00%	3	5	5	0.00383%	20.90%
Pap test	BAG family molecular chaperone regulator 3 GN=BAG3 PE=1 SV=3	BAG3_HUMAN	61,593.20	100.00%	2	2	2	0.00312%	3.65%
Swab	BAG family molecular chaperone regulator 3 GN=BAG3 PE=1 SV=3	BAG3_HUMAN	61,593.20	100.00%	1	1	2	0.00142%	2.26%
Tumor tissue	BAG family molecular chaperone regulator 3 GN=BAG3 PE=1 SV=3	BAG3_HUMAN	61,593.20	100.00%	10	10	10	0.00766%	26.40%
Pap test	Band 3 anion transport protein GN=SLC4A1 PE=1 SV=3	sp|P02730|B3AT_HUMAN	101,796.00	100.00%	2	3	4	0.00624%	3.29%
Tumor tissue	Band 3 anion transport protein GN=SLC4A1 PE=1 SV=3	sp|P02730|B3AT_HUMAN	101,796.00	100.00%	10	13	20	0.01530%	13.90%
Swab	Barrier-to-autointegration factor GN=BANF1 PE=1 SV=1	BAF_HUMAN	10,058.70	100.00%	1	1	2	0.00142%	27.00%
Tumor tissue	Barrier-to-autointegration factor GN=BANF1 PE=1 SV=1	BAF_HUMAN	10,058.70	100.00%	4	7	24	0.01840%	53.90%
Tumor tissue	Basal cell adhesion molecule GN=BCAM PE=1 SV=2	BCAM_HUMAN	67,403.90	100.00%	13	13	21	0.01610%	32.20%
Pap test	Basement membrane-specific heparan sulfate proteoglycan core protein GN=HSPG2 PE=1 SV=4	PGBM_HUMAN	468,810.60	100.00%	2	2	2	0.00312%	0.64%
Swab	Basement membrane-specific heparan sulfate proteoglycan core protein GN=HSPG2 PE=1 SV=4	PGBM_HUMAN	468,810.60	100.00%	1	1	1	0.00071%	0.34%
Tumor tissue	Basement membrane-specific heparan sulfate proteoglycan core protein GN=HSPG2 PE=1 SV=4	PGBM_HUMAN	468,810.60	100.00%	59	92	150	0.11500%	19.00%
Tumor tissue	Basic leucine zipper and W2 domain-containing protein 2 (Fragment) GN=BZW2 PE=1 SV=1	E7ETZ4_HUMAN	46,915.00	100.00%	2	2	7	0.00536%	12.30%
Tumor tissue	Bcl-2-associated transcription factor 1 GN=BCLAF1 PE=1 SV=1	sp|Q9NYF8-3|BCLF1_HUMAN	100,408.60	100.00%	2	2	14	0.01070%	13.10%
Tumor tissue	Bcl-2-like protein 13 GN=BCL2L13 PE=1 SV=1	sp|Q9BXK5|B2L13_HUMAN	54,389.40	100.00%	3	3	3	0.00230%	7.86%

Biological sample	Protein name	Protein accession numbers	Protein molecular weight (Da)	Protein identification probability	Exclusive unique peptide count	Exclusive unique spectrum count	Total spectrum count	Percentage of total spectra	Percentage sequence coverage
Tumor tissue	Beta-1,3-glucosyltransferase GN=B3GLCT PE=1 SV=2	B3GLT_HUMAN	56,566.00	100.00%	2	3	4	0.00306%	8.63%
Pap test	Beta-2-glycoprotein 1 GN=APOH PE=1 SV=3	APOH_HUMAN	38,298.50	100.00%	13	25	76	0.11900%	49.30%
Swab	Beta-2-glycoprotein 1 GN=APOH PE=1 SV=3	APOH_HUMAN	38,298.50	100.00%	13	22	94	0.06660%	38.00%
Tumor tissue	Beta-2-glycoprotein 1 GN=APOH PE=1 SV=3	APOH_HUMAN	38,298.50	100.00%	2	2	2	0.00153%	4.93%
Pap test	Beta-2-microglobulin GN=B2M PE=1 SV=1	B2MG_HUMAN	13,714.90	100.00%	2	2	4	0.00624%	16.80%
Swab	Beta-2-microglobulin GN=B2M PE=1 SV=1	B2MG_HUMAN	13,714.90	100.00%	2	3	7	0.00496%	16.80%
Tumor tissue	Beta-2-microglobulin GN=B2M PE=1 SV=1	B2MG_HUMAN	13,714.90	100.00%	3	5	8	0.00612%	19.30%
Tumor tissue	Beta-2-syntrophin GN=SNTB2 PE=1 SV=1	sp|Q13425|SNTB2_HUMAN	57,951.00	100.00%	10	10	12	0.00919%	23.90%
Tumor tissue	Beta-actin-like protein 2 GN=ACTBL2 PE=1 SV=2	ACTBL_HUMAN	42,004.20	100.00%	3	4	101	0.07730%	24.50%
Tumor tissue	Beta-catenin-like protein 1 GN=CTNNBL1 PE=1 SV=1	sp|Q8WYA6|CTBL1_HUMAN	65,704.90	100.00%	5	5	6	0.00459%	12.00%
Tumor tissue	Beta-centractin GN=ACTR1B PE=1 SV=1	ACTY_HUMAN	42,294.40	100.00%	3	3	6	0.00459%	23.70%
Pap test	Beta-hexosaminidase GN=HEXA PE=1 SV=1	H3BP20_HUMAN	58,441.70	99.90%	1	1	1	0.00156%	2.04%
Tumor tissue	Beta-hexosaminidase GN=HEXA PE=1 SV=1	sp|P06865|HEXA_HUMAN	58,441.70	100.00%	5	5	6	0.00459%	13.30%
Pap test	Beta-hexosaminidase subunit beta GN=HEXB PE=1 SV=3	HEXB_HUMAN	63,113.30	100.00%	5	5	5	0.00780%	10.80%
Swab	Beta-hexosaminidase subunit beta GN=HEXB PE=1 SV=3	HEXB_HUMAN	63,113.30	100.00%	6	6	9	0.00638%	12.60%
Tumor tissue	Beta-hexosaminidase subunit beta GN=HEXB PE=1 SV=3	HEXB_HUMAN	63,113.30	100.00%	4	4	4	0.00306%	8.99%
Pap test	BICD family-like cargo adapter 2 GN=BICDL2 PE=1 SV=2	sp|A1A5D9|BICL2_HUMAN	56,835.20	100.00%	2	2	2	0.00312%	4.33%

Biological sample	Protein name	Protein accession numbers	Protein molecular weight (Da)	Protein identification probability	Exclusive unique peptide count	Exclusive unique spectrum count	Total spectrum count	Percentage of total spectra	Percentage sequence coverage
Tumor tissue	Bifunctional 3'-phosphoadenosine 5'-phosphosulfate synthase 1 GN=PAPSS1 PE=1 SV=2	PAPS1_HUMAN	70,833.30	100.00%	5	5	5	0.00383%	9.46%
Swab	Bifunctional epoxide hydrolase 2 GN=EPHX2 PE=1 SV=1	sp|P34913|HYES_HUMAN	58,856.90	100.00%	2	2	2	0.00142%	7.07%
Tumor tissue	Bifunctional epoxide hydrolase 2 GN=EPHX2 PE=1 SV=1	sp|P34913|HYES_HUMAN	58,856.90	99.90%	1	1	1	0.00077%	3.82%
Pap test	Bifunctional glutamate/proline--tRNA ligase GN=EPRS PE=1 SV=5	SYEP_HUMAN	170,593.20	100.00%	2	2	2	0.00312%	1.72%
Swab	Bifunctional glutamate/proline--tRNA ligase GN=EPRS PE=1 SV=5	SYEP_HUMAN	170,593.20	100.00%	2	2	2	0.00142%	1.85%
Tumor tissue	Bifunctional glutamate/proline--tRNA ligase GN=EPRS PE=1 SV=5	SYEP_HUMAN	170,593.20	100.00%	17	22	25	0.01910%	15.80%
Tumor tissue	Bifunctional lysine-specific demethylase and histidyl-hydroxylase NO66 GN=NO66 PE=1 SV=2	sp|Q9H6W3|NO66_HUMAN	71,086.50	100.00%	2	2	2	0.00153%	3.59%
Tumor tissue	Bifunctional polynucleotide phosphatase/kinase GN=PNKP PE=1 SV=1	M0R3C8_HUMAN	49,296.80	100.00%	1	1	2	0.00153%	5.71%
Tumor tissue	Biglycan GN=BGN PE=1 SV=2	PGS1_HUMAN	41,655.90	100.00%	6	14	77	0.05890%	29.60%
Pap test	Biliverdin reductase A GN=BLVRA PE=1 SV=2	BIEA_HUMAN	33,429.00	100.00%	4	4	4	0.00624%	16.60%
Swab	Biliverdin reductase A GN=BLVRA PE=1 SV=2	BIEA_HUMAN	33,429.00	100.00%	4	4	6	0.00425%	15.90%
Tumor tissue	Biliverdin reductase A GN=BLVRA PE=1 SV=2	BIEA_HUMAN	33,429.00	100.00%	5	5	6	0.00459%	22.30%
Pap test	Bisphosphoglycerate mutase GN=BPGM PE=1 SV=2	PMGE_HUMAN	30,005.90	99.20%	1	1	1	0.00156%	7.34%
Swab	Bisphosphoglycerate mutase GN=BPGM PE=1 SV=2	PMGE_HUMAN	30,005.90	100.00%	2	2	2	0.00142%	8.49%
Tumor tissue	Bisphosphoglycerate mutase GN=BPGM PE=1 SV=2	PMGE_HUMAN	30,005.90	100.00%	2	2	2	0.00153%	12.00%
Pap test	Bleomycin hydrolase GN=BLMH PE=1 SV=1	BLMH_HUMAN	52,562.60	100.00%	2	2	2	0.00312%	5.49%

Biological sample	Protein name	Protein accession numbers	Protein molecular weight (Da)	Protein identification probability	Exclusive unique peptide count	Exclusive unique spectrum count	Total spectrum count	Percentage of total spectra	Percentage sequence coverage
Swab	Bleomycin hydrolase GN=BLMH PE=1 SV=1	BLMH_HUMAN	52,562.60	100.00%	3	3	4	0.00283%	8.79%
Tumor tissue	Bleomycin hydrolase GN=BLMH PE=1 SV=1	BLMH_HUMAN	52,562.60	100.00%	4	6	7	0.00536%	11.90%
Pap test	BPI fold-containing family B member 1 GN=BPIFB1 PE=1 SV=1	sp|Q8TDL5|BPIB1_HUMAN	52,443.80	100.00%	16	30	86	0.13400%	46.90%
Swab	BPI fold-containing family B member 1 GN=BPIFB1 PE=1 SV=1	sp|Q8TDL5|BPIB1_HUMAN	52,443.80	100.00%	11	15	36	0.02550%	30.60%
Pap test	Brain acid soluble protein 1 GN=BASP1 PE=1 SV=2	sp|P80723|BASP1_HUMAN	22,693.30	100.00%	4	5	5	0.00780%	29.50%
Swab	Brain acid soluble protein 1 GN=BASP1 PE=1 SV=2	sp|P80723|BASP1_HUMAN	22,693.30	100.00%	5	6	8	0.00567%	48.00%
Tumor tissue	Brain acid soluble protein 1 GN=BASP1 PE=1 SV=2	sp|P80723|BASP1_HUMAN	22,693.30	100.00%	7	16	17	0.01300%	57.30%
Tumor tissue	Brain-specific angiogenesis inhibitor 1-associated protein 2-like protein 1 GN=BAIAP2L1 PE=1 SV=2	BI2L1_HUMAN	56,883.90	100.00%	2	3	4	0.00306%	7.24%
Tumor tissue	Branched-chain-amino-acid aminotransferase GN=BCAT2 PE=1 SV=1	sp|O15382|BCAT2_HUMAN	39,915.80	100.00%	2	2	2	0.00153%	9.66%
Tumor tissue	Breakpoint cluster region protein GN=BCR PE=1 SV=2	sp|P11274|BCR_HUMAN	142,822.60	100.00%	2	2	3	0.00230%	2.36%
Swab	Breast carcinoma-amplified sequence 1 GN=BCAS1 PE=1 SV=2	sp|O75363|BCAS1_HUMAN	61,708.70	99.80%	1	1	2	0.00142%	5.99%
Tumor tissue	Brefeldin A-inhibited guanine nucleotide-exchange protein 1 GN=ARFGEF1 PE=1 SV=2	BIG1_HUMAN	208,771.30	100.00%	5	5	6	0.00459%	5.46%
Tumor tissue	Bromodomain-containing protein 4 GN=BRD4 PE=1 SV=2	sp|O60885|BRD4_HUMAN	152,216.90	100.00%	1	1	4	0.00306%	4.41%
Pap test	BTB/POZ domain-containing protein KCTD12 GN=KCTD12 PE=1 SV=1	KCD12_HUMAN	35,701.80	100.00%	3	3	5	0.00780%	15.10%
Swab	BTB/POZ domain-containing protein KCTD12 GN=KCTD12 PE=1 SV=1	KCD12_HUMAN	35,701.80	100.00%	2	2	2	0.00142%	7.69%

Biological sample	Protein name	Protein accession numbers	Protein molecular weight (Da)	Protein identification probability	Exclusive unique peptide count	Exclusive unique spectrum count	Total spectrum count	Percentage of total spectra	Percentage sequence coverage
Tumor tissue	BTB/POZ domain-containing protein KCTD12 GN=KCTD12 PE=1 SV=1	KCD12_HUMAN	35,701.80	100.00%	7	10	18	0.01380%	31.10%
Tumor tissue	BUB3-interacting and GLEBS motif-containing protein ZNF207 GN=ZNF207 PE=1 SV=1	sp|O43670|ZN207_HUMAN	52,542.10	100.00%	1	2	4	0.00306%	5.27%
Tumor tissue	Bystin GN=BYSL PE=1 SV=3	BYST_HUMAN	49,602.50	100.00%	2	2	2	0.00153%	7.78%
Swab	C-1-tetrahydrofolate synthase, cytoplasmic GN=MTHFD1 PE=1 SV=3	C1TC_HUMAN	101,561.50	100.00%	6	6	9	0.00638%	7.81%
Tumor tissue	C-1-tetrahydrofolate synthase, cytoplasmic GN=MTHFD1 PE=1 SV=3	C1TC_HUMAN	101,561.50	100.00%	22	27	39	0.02990%	30.10%
Pap test	C4b-binding protein alpha chain GN=C4BPA PE=1 SV=2	C4BPA_HUMAN	67,033.00	100.00%	2	2	2	0.00312%	4.19%
Swab	C4b-binding protein alpha chain GN=C4BPA PE=1 SV=2	C4BPA_HUMAN	67,033.00	100.00%	3	3	4	0.00283%	7.04%
Tumor tissue	C4b-binding protein alpha chain GN=C4BPA PE=1 SV=2	C4BPA_HUMAN	67,033.00	100.00%	4	8	8	0.00612%	8.88%
Swab	CAD protein GN=CAD PE=1 SV=1	PYR1_HUMAN	236,022.10	99.60%	1	1	1	0.00071%	0.42%
Tumor tissue	CAD protein GN=CAD PE=1 SV=1	PYR1_HUMAN	236,022.10	100.00%	24	25	30	0.02300%	18.70%
Swab	Cadherin-1 GN=CDH1 PE=1 SV=3	sp|P12830|CADH1_HUMAN	97,457.60	100.00%	3	3	8	0.00567%	7.82%
Tumor tissue	Cadherin-1 GN=CDH1 PE=1 SV=3	sp|P12830|CADH1_HUMAN	97,457.60	100.00%	4	4	7	0.00536%	7.37%
Tumor tissue	Cadherin-11 GN=CDH11 PE=1 SV=1	sp|P55287|CAD11_HUMAN	76,457.60	100.00%	3	3	3	0.00230%	7.46%
Swab	Cadherin-5 GN=CDH5 PE=1 SV=5	sp|P33151|CADH5_HUMAN	87,529.50	100.00%	2	2	2	0.00142%	3.70%
Tumor tissue	Cadherin-6 GN=CDH6 PE=1 SV=1	sp|P55285|CADH6_HUMAN	88,311.20	100.00%	3	3	4	0.00306%	4.18%
Tumor tissue	Calcineurin B homologous protein 1 GN=CHP1 PE=1 SV=3	CHP1_HUMAN	22,457.20	99.90%	1	1	3	0.00230%	16.40%
Tumor tissue	Calcium homeostasis endoplasmic reticulum protein GN=CHERP PE=1 SV=3	CHERP_HUMAN	104,934.20	100.00%	2	3	4	0.00306%	3.17%

Biological sample	Protein name	Protein accession numbers	Protein molecular weight (Da)	Protein identification probability	Exclusive unique peptide count	Exclusive unique spectrum count	Total spectrum count	Percentage of total spectra	Percentage sequence coverage
Tumor tissue	Calcium/calmodulin-dependent protein kinase type 1 (Fragment) GN=CAMK1 PE=1 SV=1	C9JES6_HUMAN	14,021.90	100.00%	3	3	3	0.00230%	30.30%
Pap test	Calcium-activated chloride channel regulator 4 GN=CLCA4 PE=1 SV=2	sp|Q14CN2|CLCA4_HUMAN	101,285.00	100.00%	4	4	6	0.00936%	6.53%
Tumor tissue	Calcium-binding mitochondrial carrier protein Aralar1 GN=SLC25A12 PE=1 SV=2	sp|O75746|CMC1_HUMAN	74,764.00	100.00%	4	5	12	0.00919%	17.10%
Tumor tissue	Calcium-binding mitochondrial carrier protein SCaMC-1 GN=SLC25A24 PE=1 SV=2	sp|Q6NUK1|SCMC1_HUMAN	53,356.60	100.00%	5	7	10	0.00766%	16.80%
Pap test	Calcium-binding protein 39 GN=CAB39 PE=1 SV=1	CAB39_HUMAN	39,871.20	100.00%	1	1	1	0.00156%	3.23%
Tumor tissue	Calcium-binding protein 39 GN=CAB39 PE=1 SV=1	CAB39_HUMAN	39,871.20	100.00%	4	6	9	0.00689%	15.80%
Pap test	Calcium-regulated heat-stable protein 1 GN=CARHSP1 PE=1 SV=2	CHSP1_HUMAN	15,891.60	99.90%	1	1	1	0.00156%	10.90%
Swab	Calcium-regulated heat-stable protein 1 GN=CARHSP1 PE=1 SV=2	CHSP1_HUMAN	15,891.60	100.00%	1	1	1	0.00071%	24.50%
Tumor tissue	Calcium-regulated heat-stable protein 1 GN=CARHSP1 PE=1 SV=2	CHSP1_HUMAN	15,891.60	100.00%	3	4	8	0.00612%	25.90%
Swab	Calcyphosin GN=CAPS PE=1 SV=1	sp|Q13938|CAYP1_HUMAN	20,967.70	100.00%	1	2	35	0.02480%	53.40%
Tumor tissue	Caldesmon GN=CALD1 PE=1 SV=3	sp|Q05682|CALD1_HUMAN	93,231.70	100.00%	3	5	78	0.05970%	32.30%
Tumor tissue	Calmin GN=CLMN PE=1 SV=1	CLMN_HUMAN	111,652.50	100.00%	2	3	4	0.00306%	2.89%
Pap test	Calmodulin GN=CALM1 PE=1 SV=2	CALM_HUMAN	16,838.00	100.00%	1	1	6	0.00936%	22.10%
Swab	Calmodulin GN=CALM1 PE=1 SV=2	CALM_HUMAN	16,838.00	100.00%	5	11	27	0.01910%	53.00%
Tumor tissue	Calmodulin GN=CALM1 PE=1 SV=2	CALM_HUMAN	16,966.10	100.00%	3	4	13	0.00995%	30.90%
Pap test	Calmodulin-like protein 3 GN=CALML3 PE=1 SV=2	CALL3_HUMAN	16,890.80	100.00%	5	6	13	0.02030%	43.00%
Swab	Calmodulin-like protein 3 GN=CALML3 PE=1 SV=2	CALL3_HUMAN	16,890.80	100.00%	6	8	17	0.01200%	49.00%
Pap test	Calmodulin-like protein 5 GN=CALML5 PE=1 SV=2	CALL5_HUMAN	15,892.90	100.00%	2	2	2	0.00312%	13.00%

Biological sample	Protein name	Protein accession numbers	Protein molecular weight (Da)	Protein identification probability	Exclusive unique peptide count	Exclusive unique spectrum count	Total spectrum count	Percentage of total spectra	Percentage sequence coverage
Swab	Calmodulin-like protein 5 GN=CALML5 PE=1 SV=2	CALL5_HUMAN	15,892.90	100.00%	2	2	2	0.00142%	16.40%
Tumor tissue	Calmodulin-like protein 5 GN=CALML5 PE=1 SV=2	CALL5_HUMAN	15,892.90	100.00%	6	12	17	0.01300%	59.60%
Tumor tissue	Calnexin (Fragment) GN=CANX PE=1 SV=1	D6RB85_HUMAN	16,008.60	98.60%	1	1	6	0.00459%	32.60%
Pap test	Calpain-1 catalytic subunit GN=CAPN1 PE=1 SV=1	CAN1_HUMAN	81,892.60	100.00%	20	25	48	0.07490%	29.10%
Swab	Calpain-1 catalytic subunit GN=CAPN1 PE=1 SV=1	CAN1_HUMAN	81,892.60	100.00%	17	20	38	0.02690%	30.10%
Tumor tissue	Calpain-1 catalytic subunit GN=CAPN1 PE=1 SV=1	CAN1_HUMAN	81,892.60	100.00%	13	17	23	0.01760%	20.30%
Pap test	Calpain-2 catalytic subunit GN=CAPN2 PE=1 SV=6	sp|P17655|CAN2_HUMAN	79,999.10	100.00%	5	5	5	0.00780%	11.60%
Swab	Calpain-2 catalytic subunit GN=CAPN2 PE=1 SV=6	sp|P17655|CAN2_HUMAN	79,999.10	100.00%	5	5	7	0.00496%	12.40%
Tumor tissue	Calpain-2 catalytic subunit GN=CAPN2 PE=1 SV=6	sp|P17655|CAN2_HUMAN	79,999.10	100.00%	4	8	10	0.00766%	7.29%
Pap test	Calpain-5 GN=CAPN5 PE=1 SV=2	CAN5_HUMAN	77,470.00	99.90%	1	1	1	0.00156%	2.97%
Tumor tissue	Calpain-5 GN=CAPN5 PE=1 SV=2	CAN5_HUMAN	77,470.00	100.00%	3	3	3	0.00230%	6.88%
Tumor tissue	Calponin-1 GN=CNN1 PE=1 SV=2	sp|P51911|CNN1_HUMAN	33,171.00	100.00%	1	1	48	0.03670%	52.90%
Tumor tissue	Calponin-3 GN=CNN3 PE=1 SV=1	sp|Q15417|CNN3_HUMAN	36,414.60	100.00%	3	5	29	0.02220%	47.40%
Pap test	Calreticulin GN=CALR PE=1 SV=1	CALR_HUMAN	48,142.90	100.00%	2	3	26	0.04060%	37.20%
Swab	Calreticulin GN=CALR PE=1 SV=1	CALR_HUMAN	48,142.90	100.00%	2	2	31	0.02200%	42.00%
Tumor tissue	Calreticulin GN=CALR PE=1 SV=1	CALR_HUMAN	48,142.90	100.00%	3	4	55	0.04210%	36.20%
Tumor tissue	Calretinin GN=CALB2 PE=2 SV=2	CALB2_HUMAN	31,541.80	100.00%	4	4	5	0.00383%	21.80%
Tumor tissue	cAMP-dependent protein kinase catalytic subunit alpha GN=PRKACA PE=1 SV=2	sp|P17612|KAPCA_HUMAN	40,591.40	100.00%	4	5	9	0.00689%	18.20%

Biological sample	Protein name	Protein accession numbers	Protein molecular weight (Da)	Protein identification probability	Exclusive unique peptide count	Exclusive unique spectrum count	Total spectrum count	Percentage of total spectra	Percentage sequence coverage
Swab	cAMP-dependent protein kinase type I-alpha regulatory subunit GN=PRKAR1A PE=1 SV=1	sp|P10644|KAP0_HUMAN	42,982.40	100.00%	3	4	5	0.00354%	10.80%
Tumor tissue	cAMP-dependent protein kinase type I-alpha regulatory subunit GN=PRKAR1A PE=1 SV=1	sp|P10644|KAP0_HUMAN	42,982.40	100.00%	8	13	19	0.01450%	28.30%
Swab	cAMP-dependent protein kinase type II-alpha regulatory subunit GN=PRKAR2A PE=1 SV=2	sp|P13861|KAP2_HUMAN	45,518.80	99.60%	1	2	2	0.00142%	4.95%
Tumor tissue	cAMP-dependent protein kinase type II-alpha regulatory subunit GN=PRKAR2A PE=1 SV=2	sp|P13861|KAP2_HUMAN	45,518.80	100.00%	8	12	18	0.01380%	29.70%
Tumor tissue	cAMP-dependent protein kinase type II-beta regulatory subunit GN=PRKAR2B PE=1 SV=3	KAP3_HUMAN	46,302.70	100.00%	2	3	8	0.00612%	15.60%
Tumor tissue	cAMP-regulated phosphoprotein 19 GN=ARPP19 PE=1 SV=1	sp|P56211|ARP19_HUMAN	14,484.80	99.60%	1	1	4	0.00306%	39.70%
Tumor tissue	Cancer-related nucleoside-triphosphatase GN=NTPCR PE=1 SV=1	NTPCR_HUMAN	20,712.80	100.00%	3	3	3	0.00230%	23.70%
Pap test	Carbonic anhydrase 1 GN=CA1 PE=1 SV=2	CAH1_HUMAN	28,870.40	100.00%	6	15	45	0.07020%	51.70%
Swab	Carbonic anhydrase 1 GN=CA1 PE=1 SV=2	CAH1_HUMAN	28,870.40	100.00%	1	1	3	0.00213%	11.50%
Tumor tissue	Carbonic anhydrase 1 GN=CA1 PE=1 SV=2	CAH1_HUMAN	28,870.40	100.00%	9	23	40	0.03060%	70.10%
Pap test	Carbonic anhydrase 2 GN=CA2 PE=1 SV=2	CAH2_HUMAN	29,246.60	100.00%	3	4	4	0.00624%	14.20%
Swab	Carbonic anhydrase 2 GN=CA2 PE=1 SV=2	CAH2_HUMAN	29,246.60	100.00%	4	4	4	0.00283%	19.20%
Tumor tissue	Carbonic anhydrase 2 GN=CA2 PE=1 SV=2	CAH2_HUMAN	29,246.60	100.00%	10	13	23	0.01760%	52.70%
Tumor tissue	Carbonic anhydrase 3 GN=CA3 PE=1 SV=3	CAH3_HUMAN	29,557.80	100.00%	3	3	3	0.00230%	17.70%
Pap test	Carbonyl reductase [NADPH] 1 GN=CBR1 PE=1 SV=3	sp|P16152|CBR1_HUMAN	30,374.80	100.00%	6	6	14	0.02180%	42.60%

Biological sample	Protein name	Protein accession numbers	Protein molecular weight (Da)	Protein identification probability	Exclusive unique peptide count	Exclusive unique spectrum count	Total spectrum count	Percentage of total spectra	Percentage sequence coverage
Swab	Carbonyl reductase [NADPH] 1 GN=CBR1 PE=1 SV=3	sp|P16152|CBR1_HUMAN	30,374.80	100.00%	6	9	29	0.02060%	51.60%
Tumor tissue	Carbonyl reductase [NADPH] 1 GN=CBR1 PE=1 SV=3	sp|P16152|CBR1_HUMAN	30,374.80	100.00%	4	4	13	0.00995%	37.50%
Pap test	Carbonyl reductase [NADPH] 3 GN=CBR3 PE=1 SV=3	CBR3_HUMAN	30,850.30	99.90%	1	1	3	0.00468%	10.10%
Swab	Carbonyl reductase [NADPH] 3 GN=CBR3 PE=1 SV=3	CBR3_HUMAN	30,850.30	100.00%	2	2	10	0.00709%	22.00%
Tumor tissue	Carbonyl reductase [NADPH] 3 GN=CBR3 PE=1 SV=3	CBR3_HUMAN	30,850.30	100.00%	1	1	6	0.00459%	19.50%
Pap test	Carboxypeptidase GN=CTSA PE=1 SV=1	X6R5C5_HUMAN	54,257.40	99.70%	1	1	1	0.00156%	2.70%
Swab	Carboxypeptidase GN=CTSA PE=1 SV=1	X6R5C5_HUMAN	56,234.50	100.00%	1	1	2	0.00142%	2.70%
Tumor tissue	Carboxypeptidase GN=CTSA PE=1 SV=1	X6R5C5_HUMAN	54,257.40	100.00%	3	3	3	0.00230%	11.90%
Tumor tissue	Carboxypeptidase B2 GN=CPB2 PE=1 SV=1	sp|Q96IY4|CBPB2_HUMAN	44,022.50	100.00%	2	2	2	0.00153%	7.25%
Tumor tissue	Carboxypeptidase Q GN=CPQ PE=1 SV=1	CBPQ_HUMAN	51,888.10	100.00%	1	1	4	0.00306%	9.75%
Pap test	Carcinoembryonic antigen-related cell adhesion molecule 5 GN=CEACAM5 PE=1 SV=1	sp|P06731|CEAM5_HUMAN	76,795.90	100.00%	5	7	20	0.03120%	15.40%
Swab	Carcinoembryonic antigen-related cell adhesion molecule 5 GN=CEACAM5 PE=1 SV=1	sp|P06731|CEAM5_HUMAN	76,795.90	100.00%	2	2	7	0.00496%	4.56%
Pap test	Carcinoembryonic antigen-related cell adhesion molecule 6 GN=CEACAM6 PE=1 SV=3	CEAM6_HUMAN	37,194.80	100.00%	3	5	21	0.03280%	21.50%
Swab	Carcinoembryonic antigen-related cell adhesion molecule 6 GN=CEACAM6 PE=1 SV=3	CEAM6_HUMAN	37,194.80	100.00%	2	4	8	0.00567%	9.88%
Pap test	Carcinoembryonic antigen-related cell adhesion molecule 7 GN=CEACAM7 PE=1 SV=1	CEAM7_HUMAN	29,379.00	100.00%	2	4	10	0.01560%	19.20%

Biological sample	Protein name	Protein accession numbers	Protein molecular weight (Da)	Protein identification probability	Exclusive unique peptide count	Exclusive unique spectrum count	Total spectrum count	Percentage of total spectra	Percentage sequence coverage
Swab	Carcinoembryonic antigen-related cell adhesion molecule 7 GN=CEACAM7 PE=1 SV=1	sp|Q14002|CEAM7_HUMAN	29,379.00	99.80%	1	1	4	0.00283%	10.60%
Pap test	Carcinoembryonic antigen-related cell adhesion molecule 8 GN=CEACAM8 PE=1 SV=2	CEAM8_HUMAN	38,153.90	100.00%	2	2	9	0.01400%	11.20%
Swab	Carcinoembryonic antigen-related cell adhesion molecule 8 GN=CEACAM8 PE=1 SV=2	CEAM8_HUMAN	38,153.90	100.00%	1	1	6	0.00425%	6.59%
Tumor tissue	Carnitine O-palmitoyltransferase 1, liver isoform GN=CPT1A PE=1 SV=2	sp|P50416|CPT1A_HUMAN	88,370.10	100.00%	7	7	8	0.00612%	13.50%
Tumor tissue	Carnitine O-palmitoyltransferase 2, mitochondrial GN=CPT2 PE=1 SV=2	CPT2_HUMAN	73,779.20	100.00%	4	4	7	0.00536%	14.60%
Tumor tissue	Cartilage intermediate layer protein 1 GN=CILP PE=1 SV=4	CILP1_HUMAN	132,565.10	100.00%	15	17	20	0.01530%	15.70%
Tumor tissue	Cartilage-associated protein GN=CRTAP PE=1 SV=1	C9JP16_HUMAN	46,563.50	100.00%	4	5	5	0.00383%	14.00%
Tumor tissue	Casein kinase II subunit alpha GN=CSNK2A1 PE=1 SV=1	E7EU96_HUMAN	45,311.80	100.00%	8	10	13	0.00995%	30.90%
Tumor tissue	Casein kinase II subunit alpha' GN=CSNK2A2 PE=1 SV=1	CSK22_HUMAN	41,214.50	100.00%	4	6	6	0.00459%	13.70%
Tumor tissue	Casein kinase II subunit beta GN=CSNK2B PE=1 SV=1	CSK2B_HUMAN	26,926.20	100.00%	2	3	4	0.00306%	14.40%
Tumor tissue	Caseinolytic peptidase B protein homolog GN=CLPB PE=1 SV=1	F5GX99_HUMAN	54,823.70	100.00%	1	1	3	0.00230%	8.78%
Swab	Caspase GN=CASP1 PE=1 SV=1	sp|P29466|CASP1_HUMAN	40,944.50	100.00%	2	2	2	0.00142%	6.54%
Tumor tissue	Caspase GN=CASP1 PE=1 SV=1	sp|P29466|CASP1_HUMAN	40,944.50	100.00%	5	7	7	0.00536%	21.80%
Pap test	Caspase-14 GN=CASP14 PE=1 SV=2	CASPE_HUMAN	27,679.80	100.00%	8	10	14	0.02180%	36.80%
Swab	Caspase-14 GN=CASP14 PE=1 SV=2	CASPE_HUMAN	27,679.80	100.00%	8	12	19	0.01350%	40.10%
Tumor tissue	Caspase-6 (Fragment) GN=CASP6 PE=1 SV=1	sp|P55212|CASP6_HUMAN	16,716.80	100.00%	2	2	2	0.00153%	14.70%
Pap test	Catalase GN=CAT PE=1 SV=3	CATA_HUMAN	59,756.50	100.00%	18	31	57	0.08890%	44.60%
Swab	Catalase GN=CAT PE=1 SV=3	CATA_HUMAN	59,756.50	100.00%	17	22	38	0.02690%	39.30%
Tumor tissue	Catalase GN=CAT PE=1 SV=3	CATA_HUMAN	59,756.50	100.00%	17	22	23	0.01760%	36.10%

Biological sample	Protein name	Protein accession numbers	Protein molecular weight (Da)	Protein identification probability	Exclusive unique peptide count	Exclusive unique spectrum count	Total spectrum count	Percentage of total spectra	Percentage sequence coverage
Pap test	Catenin beta-1 GN=CTNNB1 PE=1 SV=1	B4DGU4_HUMAN	84,766.10	100.00%	5	5	10	0.01560%	12.80%
Tumor tissue	Catenin beta-1 GN=CTNNB1 PE=1 SV=1	B4DGU4_HUMAN	84,766.10	100.00%	10	13	22	0.01680%	23.80%
Pap test	Catenin delta-1 GN=CTNND1 PE=1 SV=2	sp|O60716|CTND1_HUMAN	104,850.60	100.00%	4	4	4	0.00624%	5.97%
Swab	Catenin delta-1 GN=CTNND1 PE=1 SV=2	sp|O60716|CTND1_HUMAN	104,850.60	100.00%	1	1	1	0.00071%	1.92%
Tumor tissue	Catenin delta-1 GN=CTNND1 PE=1 SV=2	sp|O60716|CTND1_HUMAN	104,850.60	100.00%	20	27	40	0.03060%	27.00%
Pap test	Cathelicidin antimicrobial peptide GN=CAMP PE=1 SV=1	CAMP_HUMAN	19,591.60	100.00%	3	5	8	0.01250%	18.20%
Swab	Cathelicidin antimicrobial peptide GN=CAMP PE=1 SV=1	CAMP_HUMAN	19,591.60	100.00%	4	6	12	0.00850%	25.90%
Pap test	Cathepsin B GN=CTSB PE=1 SV=3	CATB_HUMAN	37,821.20	100.00%	5	8	9	0.01400%	21.20%
Swab	Cathepsin B GN=CTSB PE=1 SV=3	CATB_HUMAN	37,821.20	100.00%	7	16	41	0.02910%	35.40%
Tumor tissue	Cathepsin B GN=CTSB PE=1 SV=3	CATB_HUMAN	37,821.20	100.00%	5	11	14	0.01070%	15.00%
Pap test	Cathepsin D GN=CTSD PE=1 SV=1	A0A1B0GVP3_HUMAN	43,687.90	100.00%	1	1	53	0.08270%	35.30%
Swab	Cathepsin D GN=CTSD PE=1 SV=1	A0A1B0GVP3_HUMAN	43,687.90	100.00%	1	1	51	0.03610%	21.50%
Tumor tissue	Cathepsin D GN=CTSD PE=1 SV=1	A0A1B0GVP3_HUMAN	43,687.90	100.00%	1	1	35	0.02680%	25.70%
Pap test	Cathepsin G GN=CTSG PE=1 SV=2	CATG_HUMAN	28,837.50	100.00%	7	9	13	0.02030%	31.40%
Swab	Cathepsin G GN=CTSG PE=1 SV=2	CATG_HUMAN	28,837.50	100.00%	3	3	4	0.00283%	16.90%
Tumor tissue	Cathepsin G GN=CTSG PE=1 SV=2	CATG_HUMAN	28,837.50	100.00%	3	4	5	0.00383%	12.20%
Pap test	Cathepsin S GN=CTSS PE=1 SV=3	sp|P25774|CATS_HUMAN	37,495.60	100.00%	4	4	5	0.00780%	15.40%
Swab	Cathepsin S GN=CTSS PE=1 SV=3	sp|P25774|CATS_HUMAN	37,495.60	100.00%	6	6	12	0.00850%	22.40%
Tumor tissue	Cathepsin S GN=CTSS PE=1 SV=3	sp|P25774|CATS_HUMAN	37,495.60	100.00%	2	2	2	0.00153%	5.44%
Pap test	Cathepsin Z GN=CTSZ PE=1 SV=1	CATZ_HUMAN	33,868.20	100.00%	4	4	4	0.00624%	17.50%
Swab	Cathepsin Z GN=CTSZ PE=1 SV=1	CATZ_HUMAN	33,868.20	100.00%	3	3	3	0.00213%	12.20%
Tumor tissue	Cathepsin Z GN=CTSZ PE=1 SV=1	CATZ_HUMAN	33,868.20	100.00%	4	4	4	0.00306%	13.90%

Biological sample	Protein name	Protein accession numbers	Protein molecular weight (Da)	Protein identification probability	Exclusive unique peptide count	Exclusive unique spectrum count	Total spectrum count	Percentage of total spectra	Percentage sequence coverage
Tumor tissue	Cation-independent mannose-6-phosphate receptor GN=IGF2R PE=1 SV=3	MPRI_HUMAN	274,372.70	100.00%	5	5	7	0.00536%	3.93%
Tumor tissue	Caveolin-1 GN=CAV1 PE=1 SV=4	sp|Q03135|CAV1_HUMAN	20,472.20	100.00%	4	5	5	0.00383%	29.20%
Tumor tissue	CCAAT/enhancer-binding protein zeta GN=CEBPZ PE=1 SV=3	CEBPZ_HUMAN	120,978.00	100.00%	2	2	2	0.00153%	2.66%
Tumor tissue	CD109 antigen GN=CD109 PE=1 SV=2	sp|Q6YHK3|CD109_HUMAN	161,692.20	100.00%	15	20	23	0.01760%	14.20%
Swab	CD177 antigen GN=CD177 PE=1 SV=2	sp|Q8N6Q3|CD177_HUMAN	46,362.10	99.30%	1	1	3	0.00213%	5.95%
Tumor tissue	CD2 antigen cytoplasmic tail-binding protein 2 GN=CD2BP2 PE=1 SV=1	CD2B2_HUMAN	37,647.20	100.00%	2	2	2	0.00153%	9.97%
Tumor tissue	CD2-associated protein GN=CD2AP PE=1 SV=1	CD2AP_HUMAN	71,454.00	100.00%	6	7	7	0.00536%	17.40%
Pap test	CD5 antigen-like GN=CD5L PE=1 SV=1	CD5L_HUMAN	38,085.90	100.00%	4	4	4	0.00624%	19.60%
Swab	CD5 antigen-like GN=CD5L PE=1 SV=1	CD5L_HUMAN	38,085.90	100.00%	5	7	9	0.00638%	23.30%
Tumor tissue	CDGSH iron-sulfur domain-containing protein 1 GN=CISD1 PE=1 SV=1	CISD1_HUMAN	12,199.30	100.00%	2	4	4	0.00306%	25.90%
Tumor tissue	CDK5 regulatory subunit-associated protein 3 GN=CDK5RAP3 PE=1 SV=2	sp|Q96JB5|CK5P3_HUMAN	56,921.60	100.00%	3	3	10	0.00766%	14.80%
Tumor tissue	CDKN2A-interacting protein GN=CDKN2AIP PE=1 SV=3	CARF_HUMAN	61,125.10	100.00%	2	2	2	0.00153%	7.07%
Tumor tissue	CDP-diacylglycerol--inositol 3-phosphatidyltransferase GN=CDIPT PE=1 SV=1	sp|O14735|CDIPT_HUMAN	23,540.30	100.00%	2	3	3	0.00230%	11.30%
Tumor tissue	Cell adhesion molecule 1 GN=CADM1 PE=1 SV=1	sp|Q9BY67|CADM1_HUMAN	45,532.60	100.00%	2	2	2	0.00153%	9.07%
Pap test	Cell cycle and apoptosis regulator protein 2 GN=CCAR2 PE=1 SV=2	sp|Q8N163|CCAR2_HUMAN	102,903.10	99.20%	1	1	1	0.00156%	1.52%

Biological sample	Protein name	Protein accession numbers	Protein molecular weight (Da)	Protein identification probability	Exclusive unique peptide count	Exclusive unique spectrum count	Total spectrum count	Percentage of total spectra	Percentage sequence coverage
Tumor tissue	Cell cycle and apoptosis regulator protein 2 GN=CCAR2 PE=1 SV=2	sp|Q8N163|CCAR2_HUMAN	102,903.10	100.00%	10	12	23	0.01760%	26.50%
Pap test	Cell division control protein 42 homolog GN=CDC42 PE=1 SV=2	sp|P60953|CDC42_HUMAN	21,258.80	100.00%	3	3	4	0.00624%	25.70%
Swab	Cell division control protein 42 homolog GN=CDC42 PE=1 SV=2	sp|P60953|CDC42_HUMAN	21,258.80	100.00%	4	6	12	0.00850%	33.00%
Tumor tissue	Cell division control protein 42 homolog GN=CDC42 PE=1 SV=2	sp|P60953|CDC42_HUMAN	21,258.80	100.00%	3	6	8	0.00612%	16.80%
Tumor tissue	Cell division cycle 5-like protein GN=CDC5L PE=1 SV=2	CDC5L_HUMAN	92,254.20	100.00%	10	10	10	0.00766%	19.50%
Tumor tissue	Cell division cycle protein 16 homolog GN=CDC16 PE=1 SV=1	sp|Q13042|CDC16_HUMAN	65,862.20	100.00%	2	2	2	0.00153%	4.84%
Tumor tissue	Cell growth-regulating nucleolar protein GN=LYAR PE=1 SV=2	LYAR_HUMAN	43,615.10	100.00%	4	5	5	0.00383%	15.60%
Tumor tissue	Cell surface glycoprotein MUC18 GN=MCAM PE=1 SV=2	sp|P43121|MUC18_HUMAN	71,606.50	100.00%	3	3	3	0.00230%	10.80%
Tumor tissue	Cellular retinoic acid-binding protein 1 GN=CRABP1 PE=1 SV=2	RABP1_HUMAN	15,565.60	100.00%	7	11	25	0.01910%	42.30%
Pap test	Cellular retinoic acid-binding protein 2 GN=CRABP2 PE=1 SV=2	RABP2_HUMAN	15,692.90	100.00%	4	4	5	0.00780%	39.90%
Swab	Cellular retinoic acid-binding protein 2 GN=CRABP2 PE=1 SV=2	RABP2_HUMAN	15,692.90	100.00%	4	8	26	0.01840%	42.80%
Tumor tissue	Cellular retinoic acid-binding protein 2 GN=CRABP2 PE=1 SV=2	RABP2_HUMAN	15,692.90	100.00%	5	7	8	0.00612%	29.70%
Tumor tissue	Centrin-2 GN=CETN2 PE=1 SV=1	CETN2_HUMAN	19,739.60	99.90%	1	1	2	0.00153%	12.80%
Tumor tissue	Centromere/kinetochore protein zw10 homolog GN=ZW10 PE=1 SV=3	sp|O43264|ZW10_HUMAN	88,832.50	100.00%	4	4	4	0.00306%	6.93%
Tumor tissue	Centrosomal protein of 41 kDa GN=CEP41 PE=1 SV=1	sp|Q9BYV8|CEP41_HUMAN	41,370.10	100.00%	3	3	3	0.00230%	13.70%
Tumor tissue	Ceroid-lipofuscinosis neuronal 6 late infantile variant isoform 2 GN=CLN6 PE=1 SV=1	sp|Q9NWW5|CLN6_HUMAN	19,300.30	100.00%	2	2	2	0.00153%	14.00%
Pap test	Ceruloplasmin GN=CP PE=1 SV=1	CERU_HUMAN	122,207.90	100.00%	2	4	211	0.32900%	40.40%
Swab	Ceruloplasmin GN=CP PE=1 SV=1	CERU_HUMAN	122,207.90	100.00%	1	2	230	0.16300%	30.90%

Biological sample	Protein name	Protein accession numbers	Protein molecular weight (Da)	Protein identification probability	Exclusive unique peptide count	Exclusive unique spectrum count	Total spectrum count	Percentage of total spectra	Percentage sequence coverage
Tumor tissue	cGMP-dependent protein kinase 1 GN=PRKG1 PE=1 SV=3	sp|Q13976|KGP1_HUMAN	76,367.90	100.00%	2	3	4	0.00306%	4.47%
Tumor tissue	Charged multivesicular body protein 2a GN=CHMP2A PE=1 SV=1	CHM2A_HUMAN	25,105.00	100.00%	3	4	4	0.00306%	13.10%
Tumor tissue	Charged multivesicular body protein 4b GN=CHMP4B PE=1 SV=1	CHM4B_HUMAN	24,951.20	100.00%	3	4	4	0.00306%	15.60%
Tumor tissue	Charged multivesicular body protein 6 GN=CHMP6 PE=1 SV=3	CHMP6_HUMAN	23,485.30	100.00%	2	2	2	0.00153%	10.40%
Pap test	Chitinase-3-like protein 1 GN=CHI3L1 PE=1 SV=2	CH3L1_HUMAN	42,627.00	100.00%	9	12	20	0.03120%	33.20%
Swab	Chitinase-3-like protein 1 GN=CHI3L1 PE=1 SV=2	CH3L1_HUMAN	42,627.00	100.00%	8	9	19	0.01350%	28.70%
Tumor tissue	Chitobiosyldiphosphodolichol beta-mannosyltransferase GN=ALG1 PE=1 SV=2	sp|Q9BT22|ALG1_HUMAN	52,518.70	100.00%	2	3	3	0.00230%	6.47%
Pap test	Chitotriosidase-1 GN=CHIT1 PE=1 SV=1	D6REY1_HUMAN	40,239.10	100.00%	2	2	12	0.01870%	29.40%
Swab	Chitotriosidase-1 GN=CHIT1 PE=1 SV=1	D6REY1_HUMAN	40,239.10	100.00%	1	2	13	0.00921%	19.70%
Pap test	Chloride intracellular channel protein 1 GN=CLIC1 PE=1 SV=4	CLIC1_HUMAN	26,923.30	100.00%	8	9	10	0.01560%	44.80%
Swab	Chloride intracellular channel protein 1 GN=CLIC1 PE=1 SV=4	CLIC1_HUMAN	26,923.30	100.00%	10	13	36	0.02550%	61.80%
Tumor tissue	Chloride intracellular channel protein 1 GN=CLIC1 PE=1 SV=4	CLIC1_HUMAN	26,923.30	100.00%	6	9	18	0.01380%	32.80%
Pap test	Chloride intracellular channel protein 3 GN=CLIC3 PE=1 SV=2	CLIC3_HUMAN	26,649.40	100.00%	6	8	12	0.01870%	34.30%
Swab	Chloride intracellular channel protein 3 GN=CLIC3 PE=1 SV=2	CLIC3_HUMAN	26,649.40	100.00%	5	6	8	0.00567%	29.20%
Tumor tissue	Chloride intracellular channel protein 4 GN=CLIC4 PE=1 SV=4	CLIC4_HUMAN	28,773.60	100.00%	6	9	17	0.01300%	28.10%
Pap test	Chloride intracellular channel protein 6 GN=CLIC6 PE=2 SV=3	sp|Q96NY7|CLIC6_HUMAN	73,011.00	100.00%	2	3	4	0.00624%	6.53%
Swab	Chloride intracellular channel protein 6 GN=CLIC6 PE=2 SV=3	sp|Q96NY7|CLIC6_HUMAN	73,011.00	100.00%	2	3	4	0.00283%	5.82%

Biological sample	Protein name	Protein accession numbers	Protein molecular weight (Da)	Protein identification probability	Exclusive unique peptide count	Exclusive unique spectrum count	Total spectrum count	Percentage of total spectra	Percentage sequence coverage
Tumor tissue	Chondroitin sulfate synthase 2 GN=CHPF PE=1 SV=2	sp|Q8IZ52|CHSS2_HUMAN	85,468.10	100.00%	3	3	3	0.00230%	5.42%
Tumor tissue	Chromatin complexes subunit BAP18 GN=C17orf49 PE=1 SV=1	sp|Q8IXM2|BAP18_HUMAN	16,290.30	100.00%	2	3	6	0.00459%	56.70%
Tumor tissue	Chromatin target of PRMT1 protein GN=CHTOP PE=1 SV=1	sp|Q9Y3Y2|CHTOP_HUMAN	23,662.60	100.00%	4	4	4	0.00306%	28.70%
Tumor tissue	Chromobox protein homolog 1 (Fragment) GN=CBX1 PE=1 SV=1	CBX1_HUMAN	20,030.60	99.50%	1	1	3	0.00230%	16.10%
Tumor tissue	Chromobox protein homolog 3 GN=CBX3 PE=1 SV=4	CBX3_HUMAN	20,812.00	100.00%	2	3	11	0.00842%	23.50%
Tumor tissue	Chromobox protein homolog 5 GN=CBX5 PE=1 SV=1	CBX5_HUMAN	22,225.60	100.00%	3	4	4	0.00306%	19.40%
Tumor tissue	Chromodomain-helicase-DNA-binding protein 4 GN=CHD4 PE=1 SV=1	sp|Q14839|CHD4_HUMAN	217,107.40	100.00%	12	13	21	0.01610%	12.20%
Tumor tissue	Chromosome 6 open reading frame 55, isoform CRA_b GN=VTA1 PE=1 SV=1	sp|Q9NP79|VTA1_HUMAN	33,880.00	100.00%	5	6	7	0.00536%	20.40%
Tumor tissue	Chromosome alignment-maintaining phosphoprotein 1 GN=CHAMP1 PE=1 SV=2	CHAP1_HUMAN	89,100.90	100.00%	3	3	4	0.00306%	4.68%
Tumor tissue	Chromosome transmission fidelity protein 18 homolog GN=CHTF18 PE=1 SV=1	A0A0D9SF58_HUMAN	129,403.20	100.00%	2	2	2	0.00153%	2.45%
Tumor tissue	Cilia- and flagella-associated protein 20 GN=CFAP20 PE=1 SV=1	CFA20_HUMAN	22,775.60	100.00%	2	2	3	0.00230%	9.33%
Tumor tissue	Cilia- and flagella-associated protein 74 GN=CFAP74 PE=2 SV=3	sp|Q9C0B2|CFA74_HUMAN	178,590.70	100.00%	2	2	2	0.00153%	2.90%
Tumor tissue	Cingulin GN=CGN PE=1 SV=2	sp|Q9P2M7|CING_HUMAN	136,387.40	100.00%	12	14	14	0.01070%	16.40%
Tumor tissue	Cisplatin resistance-associated overexpressed protein, isoform CRA_b GN=LUC7L3 PE=1 SV=1	LC7L3_HUMAN	58,221.40	100.00%	3	3	6	0.00459%	9.61%
Tumor tissue	Citrate lyase subunit beta-like protein, mitochondrial GN=CLYBL PE=1 SV=2	sp|Q8N0X4|CLYBL_HUMAN	37,360.70	100.00%	2	2	2	0.00153%	7.35%

Biological sample	Protein name	Protein accession numbers	Protein molecular weight (Da)	Protein identification probability	Exclusive unique peptide count	Exclusive unique spectrum count	Total spectrum count	Percentage of total spectra	Percentage sequence coverage
Pap test	Citrate synthase GN=CS PE=1 SV=1	CISY_HUMAN	50,433.00	100.00%	1	1	2	0.00312%	5.96%
Swab	Citrate synthase GN=CS PE=1 SV=1	CISY_HUMAN	50,433.00	100.00%	2	2	5	0.00354%	9.93%
Tumor tissue	Citrate synthase GN=CS PE=1 SV=1	CISY_HUMAN	50,433.00	100.00%	4	5	12	0.00919%	13.00%
Tumor tissue	Claudin-3 GN=CLDN3 PE=1 SV=1	CLD3_HUMAN	23,318.70	100.00%	2	2	3	0.00230%	19.10%
Tumor tissue	Claudin-6 GN=CLDN6 PE=1 SV=2	CLD6_HUMAN	23,291.50	100.00%	1	1	2	0.00153%	12.70%
Tumor tissue	Cleavage and polyadenylation specificity factor subunit 1 GN=CPSF1 PE=1 SV=2	CPSF1_HUMAN	160,885.90	100.00%	4	4	4	0.00306%	4.57%
Tumor tissue	Cleavage and polyadenylation specificity factor subunit 2 GN=CPSF2 PE=1 SV=2	CPSF2_HUMAN	88,489.50	100.00%	2	2	3	0.00230%	3.20%
Tumor tissue	Cleavage and polyadenylation specificity factor subunit 3 GN=CPSF3 PE=1 SV=1	CPSF3_HUMAN	73,478.90	100.00%	3	3	3	0.00230%	6.73%
Pap test	Cleavage and polyadenylation specificity factor subunit 5 GN=NUDT21 PE=1 SV=1	CPSF5_HUMAN	26,228.10	99.20%	1	1	1	0.00156%	7.93%
Tumor tissue	Cleavage and polyadenylation specificity factor subunit 5 GN=NUDT21 PE=1 SV=1	CPSF5_HUMAN	26,228.10	100.00%	7	10	14	0.01070%	36.10%
Tumor tissue	Cleavage stimulation factor subunit 1 GN=CSTF1 PE=1 SV=1	CSTF1_HUMAN	48,358.50	100.00%	5	7	7	0.00536%	17.90%
Tumor tissue	Cleavage stimulation factor subunit 3 GN=CSTF3 PE=1 SV=1	sp|Q12996|CSTF3_HUMAN	82,924.20	100.00%	2	3	4	0.00306%	7.67%
Tumor tissue	CLIP-associating protein 1 GN=CLASP1 PE=1 SV=1	sp|Q7Z460|CLAP1_HUMAN	169,455.80	100.00%	2	3	4	0.00306%	2.80%
Tumor tissue	CLIP-associating protein 2 GN=CLASP2 PE=1 SV=1	sp|O75122-3|CLAP2_HUMAN	165,703.10	100.00%	3	3	4	0.00306%	3.50%
Swab	Coactosin-like protein GN=COTL1 PE=1 SV=3	COTL1_HUMAN	15,945.20	100.00%	3	3	4	0.00283%	31.70%
Tumor tissue	Coactosin-like protein GN=COTL1 PE=1 SV=3	COTL1_HUMAN	15,945.20	100.00%	3	5	10	0.00766%	23.90%

Biological sample	Protein name	Protein accession numbers	Protein molecular weight (Da)	Protein identification probability	Exclusive unique peptide count	Exclusive unique spectrum count	Total spectrum count	Percentage of total spectra	Percentage sequence coverage
Pap test	Coagulation factor XII GN=F12 PE=1 SV=3	FA12_HUMAN	67,790.60	100.00%	6	7	8	0.01250%	12.80%
Swab	Coagulation factor XII GN=F12 PE=1 SV=3	FA12_HUMAN	67,790.60	100.00%	4	4	4	0.00283%	8.29%
Tumor tissue	Coagulation factor XII GN=F12 PE=1 SV=3	FA12_HUMAN	67,790.60	100.00%	3	4	4	0.00306%	5.69%
Tumor tissue	Coagulation factor XIII A chain GN=F13A1 PE=1 SV=4	F13A_HUMAN	83,267.30	100.00%	17	25	34	0.02600%	25.10%
Tumor tissue	Coatomer protein complex, subunit epsilon, isoform CRA_g GN=COPE PE=1 SV=1	sp|O14579|COPE_HUMAN	36,924.60	100.00%	7	10	11	0.00842%	30.50%
Pap test	Coatomer subunit beta GN=COPB1 PE=1 SV=3	COPB_HUMAN	107,146.00	100.00%	1	1	1	0.00156%	1.36%
Swab	Coatomer subunit beta GN=COPB1 PE=1 SV=3	COPB_HUMAN	107,146.00	100.00%	1	1	1	0.00071%	1.05%
Tumor tissue	Coatomer subunit beta GN=COPB1 PE=1 SV=3	COPB_HUMAN	107,146.00	100.00%	21	32	37	0.02830%	27.30%
Pap test	Coatomer subunit gamma-1 GN=COPG1 PE=1 SV=1	COPG1_HUMAN	97,719.40	99.70%	1	1	1	0.00156%	1.95%
Swab	Coatomer subunit gamma-1 GN=COPG1 PE=1 SV=1	COPG1_HUMAN	97,719.40	100.00%	1	2	3	0.00213%	2.06%
Tumor tissue	Coatomer subunit gamma-1 GN=COPG1 PE=1 SV=1	COPG1_HUMAN	97,719.40	100.00%	15	23	30	0.02300%	25.50%
Swab	Coatomer subunit zeta-1 GN=COPZ1 PE=1 SV=1	sp|P61923|COPZ1_HUMAN	22,341.00	99.50%	1	1	1	0.00071%	5.56%
Tumor tissue	Coatomer subunit zeta-1 GN=COPZ1 PE=1 SV=1	sp|P61923|COPZ1_HUMAN	20,198.90	100.00%	3	3	8	0.00612%	26.30%
Pap test	Cofilin-1 GN=CFL1 PE=1 SV=1	E9PK25_HUMAN	22,728.80	100.00%	4	5	11	0.01720%	42.60%
Swab	Cofilin-1 GN=CFL1 PE=1 SV=1	E9PK25_HUMAN	22,728.80	100.00%	7	13	63	0.04460%	46.60%
Tumor tissue	Cofilin-1 GN=CFL1 PE=1 SV=1	E9PK25_HUMAN	22,728.80	100.00%	3	5	27	0.02070%	34.30%
Tumor tissue	Cofilin-2 GN=CFL2 PE=1 SV=1	sp|Q9Y281|COF2_HUMAN	18,737.30	100.00%	2	5	18	0.01380%	37.30%
Tumor tissue	Coiled-coil domain-containing protein 124 GN=CCDC124 PE=1 SV=1	CC124_HUMAN	25,835.70	100.00%	2	4	4	0.00306%	15.70%

Biological sample	Protein name	Protein accession numbers	Protein molecular weight (Da)	Protein identification probability	Exclusive unique peptide count	Exclusive unique spectrum count	Total spectrum count	Percentage of total spectra	Percentage sequence coverage
Tumor tissue	Coiled-coil domain-containing protein 22 GN=CCDC22 PE=1 SV=1	CCD22_HUMAN	70,756.70	100.00%	7	8	8	0.00612%	16.40%
Tumor tissue	Coiled-coil domain-containing protein 47 GN=CCDC47 PE=1 SV=1	sp|Q96A33|CCD47_HUMAN	55,874.90	100.00%	3	3	9	0.00689%	13.70%
Tumor tissue	Coiled-coil domain-containing protein 58 GN=CCDC58 PE=1 SV=1	CCD58_HUMAN	15,296.70	100.00%	2	2	3	0.00230%	34.60%
Pap test	Coiled-coil domain-containing protein 6 GN=CCDC6 PE=1 SV=2	CCDC6_HUMAN	53,292.00	99.70%	1	1	1	0.00156%	2.95%
Swab	Coiled-coil domain-containing protein 6 GN=CCDC6 PE=1 SV=2	CCDC6_HUMAN	53,292.00	100.00%	1	1	2	0.00142%	4.01%
Tumor tissue	Coiled-coil domain-containing protein 6 GN=CCDC6 PE=1 SV=2	CCDC6_HUMAN	53,292.00	100.00%	10	13	14	0.01070%	25.10%
Tumor tissue	Coiled-coil domain-containing protein 93 GN=CCDC93 PE=1 SV=2	CCD93_HUMAN	73,043.90	100.00%	4	4	4	0.00306%	8.56%
Tumor tissue	Cold shock domain-containing protein E1 GN=CSDE1 PE=1 SV=2	sp|O75534|CSDE1_HUMAN	88,885.00	100.00%	7	9	9	0.00689%	13.20%
Tumor tissue	Cold-inducible RNA-binding protein GN=CIRBP PE=1 SV=1	sp|Q14011|CIRBP_HUMAN	18,648.30	99.90%	1	1	2	0.00153%	14.50%
Swab	Collagen alpha-1(I) chain GN=COL1A1 PE=1 SV=5	CO1A1_HUMAN	138,941.40	99.90%	1	1	1	0.00071%	1.84%
Tumor tissue	Collagen alpha-1(I) chain GN=COL1A1 PE=1 SV=5	CO1A1_HUMAN	138,941.40	100.00%	19	30	60	0.04590%	19.30%
Tumor tissue	Collagen alpha-1(III) chain GN=COL3A1 PE=1 SV=4	sp|P02461|CO3A1_HUMAN	138,565.00	100.00%	8	12	31	0.02370%	8.25%
Tumor tissue	Collagen alpha-1(IV) chain GN=COL4A1 PE=1 SV=3	sp|P02462|CO4A1_HUMAN	160,617.50	100.00%	1	3	8	0.00612%	2.34%
Swab	Collagen alpha-1(V) chain GN=COL5A1 PE=1 SV=3	CO5A1_HUMAN	183,562.70	99.70%	1	1	1	0.00071%	0.82%
Tumor tissue	Collagen alpha-1(V) chain GN=COL5A1 PE=1 SV=3	CO5A1_HUMAN	183,562.70	100.00%	5	5	5	0.00383%	4.79%
Tumor tissue	Collagen alpha-1(VI) chain GN=COL6A1 PE=1 SV=1	CO6A1_HUMAN	108,340.80	100.00%	20	37	94	0.07200%	20.60%
Tumor tissue	Collagen alpha-1(VIII) chain GN=COL8A1 PE=1 SV=2	CO8A1_HUMAN	73,366.60	100.00%	3	3	5	0.00383%	6.72%
Swab	Collagen alpha-1(XII) chain GN=COL12A1 PE=1 SV=1	sp|Q99715|COCA1_HUMAN	333,200.50	100.00%	1	1	1	0.00071%	0.95%

Biological sample	Protein name	Protein accession numbers	Protein molecular weight (Da)	Protein identification probability	Exclusive unique peptide count	Exclusive unique spectrum count	Total spectrum count	Percentage of total spectra	Percentage sequence coverage
Tumor tissue	Collagen alpha-1(XII) chain GN=COL12A1 PE=1 SV=1	sp|Q99715|COCA1_HUMAN	333,200.50	100.00%	78	118	167	0.12800%	33.70%
Tumor tissue	Collagen alpha-1(XV) chain GN=COL15A1 PE=1 SV=1	COFA1_HUMAN	140,061.70	100.00%	4	4	5	0.00383%	3.93%
Swab	Collagen alpha-2(I) chain GN=COL1A2 PE=1 SV=1	CO1A2_HUMAN	129,151.60	99.90%	1	1	1	0.00071%	1.98%
Tumor tissue	Collagen alpha-2(I) chain GN=COL1A2 PE=1 SV=1	CO1A2_HUMAN	129,151.60	100.00%	24	41	90	0.06890%	20.60%
Tumor tissue	Collagen alpha-2(IV) chain GN=COL4A2 PE=1 SV=4	CO4A2_HUMAN	167,557.60	100.00%	5	6	8	0.00612%	4.21%
Tumor tissue	Collagen alpha-2(V) chain GN=COL5A2 PE=1 SV=3	CO5A2_HUMAN	144,909.70	100.00%	1	2	8	0.00612%	6.14%
Tumor tissue	Collagen alpha-2(VI) chain GN=COL6A2 PE=1 SV=4	sp|P12110|CO6A2_HUMAN	108,580.80	100.00%	3	6	63	0.04820%	18.70%
Tumor tissue	Collagen alpha-3(VI) chain GN=COL6A3 PE=1 SV=2	sp|P12111-4|CO6A3_HUMAN	278,118.10	100.00%	2	2	397	0.30400%	37.90%
Tumor tissue	Collagen alpha-3(VI) chain GN=COL6A3 PE=1 SV=5	sp|P12111|CO6A3_HUMAN	343,667.40	100.00%	14	17	417	0.31900%	36.90%
Tumor tissue	Collectin-12 GN=COLEC12 PE=1 SV=3	COL12_HUMAN	81,516.90	100.00%	3	3	3	0.00230%	6.60%
Tumor tissue	COMM domain-containing protein 4 GN=COMMD4 PE=1 SV=1	sp|Q9H0A8|COMD4_HUMAN	14,583.60	100.00%	2	2	4	0.00306%	19.20%
Tumor tissue	Complement C1q subcomponent subunit B (Fragment) GN=C1QB PE=1 SV=6	C1QB_HUMAN	26,460.40	100.00%	3	7	10	0.00766%	18.90%
Pap test	Complement C1q subcomponent subunit C GN=C1QC PE=1 SV=3	C1QC_HUMAN	25,774.00	100.00%	1	1	1	0.00156%	3.67%
Swab	Complement C1q subcomponent subunit C GN=C1QC PE=1 SV=3	C1QC_HUMAN	25,774.00	100.00%	1	1	1	0.00071%	3.67%
Tumor tissue	Complement C1q subcomponent subunit C GN=C1QC PE=1 SV=3	C1QC_HUMAN	25,774.00	100.00%	4	5	6	0.00459%	21.20%
Swab	Complement C1r subcomponent GN=C1R PE=1 SV=1	C1R_HUMAN	81,890.50	100.00%	3	3	3	0.00213%	7.65%
Tumor tissue	Complement C1r subcomponent GN=C1R PE=1 SV=1	C1R_HUMAN	81,890.50	100.00%	3	3	6	0.00459%	9.32%

Biological sample	Protein name	Protein accession numbers	Protein molecular weight (Da)	Protein identification probability	Exclusive unique peptide count	Exclusive unique spectrum count	Total spectrum count	Percentage of total spectra	Percentage sequence coverage
Pap test	Complement C1s subcomponent GN=C1S PE=1 SV=1	C1S_HUMAN	76,684.20	100.00%	3	3	3	0.00468%	5.38%
Swab	Complement C1s subcomponent GN=C1S PE=1 SV=1	C1S_HUMAN	76,684.20	100.00%	5	6	7	0.00496%	14.80%
Tumor tissue	Complement C1s subcomponent GN=C1S PE=1 SV=1	C1S_HUMAN	76,684.20	100.00%	8	11	13	0.00995%	15.80%
Pap test	Complement C2 GN=C2 PE=1 SV=2	sp|P06681|CO2_HUMAN	83,268.80	100.00%	1	1	8	0.01250%	8.11%
Swab	Complement C2 GN=C2 PE=1 SV=2	sp|P06681|CO2_HUMAN	83,268.80	100.00%	3	3	18	0.01280%	23.70%
Tumor tissue	Complement C2 GN=C2 PE=1 SV=2	sp|P06681|CO2_HUMAN	83,268.80	100.00%	2	2	9	0.00689%	8.64%
Pap test	Complement C3 GN=C3 PE=1 SV=2	CO3_HUMAN	187,149.10	100.00%	62	108	319	0.49800%	44.70%
Swab	Complement C3 GN=C3 PE=1 SV=2	CO3_HUMAN	187,149.10	100.00%	76	156	717	0.50800%	52.20%
Tumor tissue	Complement C3 GN=C3 PE=1 SV=2	CO3_HUMAN	187,149.10	100.00%	52	87	150	0.11500%	39.90%
Pap test	Complement C4-A GN=C4A PE=1 SV=2	sp|P0C0L4|CO4A_HUMAN	192,786.80	100.00%	1	1	125	0.19500%	31.90%
Swab	Complement C4-A GN=C4A PE=1 SV=2	sp|P0C0L4|CO4A_HUMAN	192,786.80	100.00%	1	1	187	0.13300%	31.10%
Tumor tissue	Complement C4-A GN=C4A PE=1 SV=2	sp|P0C0L4|CO4A_HUMAN	192,786.80	100.00%	1	1	95	0.07270%	26.20%
Pap test	Complement C4-B GN=C4B PE=1 SV=2	CO4B_HUMAN	192,752.80	100.00%	2	4	128	0.20000%	32.60%
Swab	Complement C4-B GN=C4B PE=1 SV=2	CO4B_HUMAN	192,752.80	100.00%	3	5	197	0.14000%	32.30%
Tumor tissue	Complement C4-B GN=C4B PE=1 SV=2	CO4B_HUMAN	192,752.80	100.00%	2	4	98	0.07500%	26.90%
Pap test	Complement C5 GN=C5 PE=1 SV=4	CO5_HUMAN	188,309.20	100.00%	10	11	12	0.01870%	6.80%
Swab	Complement C5 GN=C5 PE=1 SV=4	CO5_HUMAN	188,309.20	100.00%	8	10	12	0.00850%	7.28%
Tumor tissue	Complement C5 GN=C5 PE=1 SV=4	CO5_HUMAN	188,309.20	100.00%	10	10	11	0.00842%	8.05%
Pap test	Complement component 1 Q subcomponent-binding protein, mitochondrial GN=C1QBP PE=1 SV=1	C1QBP_HUMAN	31,362.60	99.90%	1	1	1	0.00156%	7.09%

Biological sample	Protein name	Protein accession numbers	Protein molecular weight (Da)	Protein identification probability	Exclusive unique peptide count	Exclusive unique spectrum count	Total spectrum count	Percentage of total spectra	Percentage sequence coverage
Swab	Complement component 1 Q subcomponent-binding protein, mitochondrial GN=C1QBP PE=1 SV=1	C1QBP_HUMAN	31,362.60	100.00%	3	3	5	0.00354%	19.10%
Tumor tissue	Complement component 1 Q subcomponent-binding protein, mitochondrial GN=C1QBP PE=1 SV=1	C1QBP_HUMAN	31,362.60	100.00%	5	9	13	0.00995%	22.00%
Pap test	Complement component C6 GN=C6 PE=1 SV=3	CO6_HUMAN	104,786.20	100.00%	6	7	9	0.01400%	8.46%
Swab	Complement component C6 GN=C6 PE=1 SV=3	CO6_HUMAN	104,786.20	100.00%	13	13	13	0.00921%	19.60%
Tumor tissue	Complement component C6 GN=C6 PE=1 SV=3	CO6_HUMAN	104,786.20	100.00%	2	2	2	0.00153%	2.14%
Pap test	Complement component C7 GN=C7 PE=1 SV=2	CO7_HUMAN	93,516.60	100.00%	9	9	15	0.02340%	14.40%
Swab	Complement component C7 GN=C7 PE=1 SV=2	CO7_HUMAN	93,516.60	100.00%	12	15	22	0.01560%	20.60%
Tumor tissue	Complement component C7 GN=C7 PE=1 SV=2	CO7_HUMAN	93,516.60	100.00%	2	2	2	0.00153%	4.15%
Pap test	Complement component C8 alpha chain GN=C8A PE=1 SV=2	CO8A_HUMAN	65,163.80	100.00%	5	7	9	0.01400%	12.80%
Swab	Complement component C8 alpha chain GN=C8A PE=1 SV=2	CO8A_HUMAN	65,163.80	100.00%	10	12	17	0.01200%	25.30%
Tumor tissue	Complement component C8 alpha chain GN=C8A PE=1 SV=2	CO8A_HUMAN	65,163.80	100.00%	1	1	1	0.00077%	3.08%
Pap test	Complement component C8 beta chain GN=C8B PE=1 SV=3	CO8B_HUMAN	67,046.90	100.00%	7	7	8	0.01250%	17.90%
Swab	Complement component C8 beta chain GN=C8B PE=1 SV=3	CO8B_HUMAN	67,046.90	100.00%	8	10	13	0.00921%	19.50%
Tumor tissue	Complement component C8 beta chain GN=C8B PE=1 SV=3	CO8B_HUMAN	67,046.90	100.00%	7	8	10	0.00766%	14.60%
Pap test	Complement component C8 gamma chain GN=C8G PE=1 SV=3	CO8G_HUMAN	22,277.30	100.00%	2	2	18	0.02810%	46.50%
Swab	Complement component C8 gamma chain GN=C8G PE=1 SV=3	CO8G_HUMAN	22,277.30	100.00%	4	7	15	0.01060%	40.60%

Biological sample	Protein name	Protein accession numbers	Protein molecular weight (Da)	Protein identification probability	Exclusive unique peptide count	Exclusive unique spectrum count	Total spectrum count	Percentage of total spectra	Percentage sequence coverage
Tumor tissue	Complement component C8 gamma chain GN=C8G PE=1 SV=3	CO8G_HUMAN	22,277.30	100.00%	2	3	5	0.00383%	19.30%
Pap test	Complement component C9 GN=C9 PE=1 SV=2	CO9_HUMAN	63,174.90	100.00%	13	16	29	0.04530%	21.60%
Swab	Complement component C9 GN=C9 PE=1 SV=2	CO9_HUMAN	63,174.90	100.00%	10	14	26	0.01840%	19.10%
Tumor tissue	Complement component C9 GN=C9 PE=1 SV=2	CO9_HUMAN	63,174.90	100.00%	9	11	11	0.00842%	15.00%
Pap test	Complement factor D GN=CFD PE=1 SV=5	CFAD_HUMAN	27,862.20	100.00%	4	4	6	0.00936%	26.50%
Swab	Complement factor D GN=CFD PE=1 SV=5	CFAD_HUMAN	27,862.20	100.00%	5	6	7	0.00496%	24.50%
Tumor tissue	Complement factor D GN=CFD PE=1 SV=5	CFAD_HUMAN	27,862.20	99.80%	1	1	1	0.00077%	5.93%
Pap test	Complement factor H GN=CFH PE=1 SV=4	sp|P08603|CFAH_HUMAN	139,096.20	100.00%	40	58	103	0.16100%	46.50%
Swab	Complement factor H GN=CFH PE=1 SV=4	sp|P08603|CFAH_HUMAN	139,096.20	100.00%	36	58	112	0.07940%	41.30%
Tumor tissue	Complement factor H GN=CFH PE=1 SV=4	sp|P08603|CFAH_HUMAN	139,096.20	100.00%	12	14	16	0.01220%	12.90%
Pap test	Complement factor H-related protein 2 GN=CFHR2 PE=1 SV=1	V9GYE7_HUMAN	28,911.80	99.30%	1	1	4	0.00624%	8.66%
Swab	Complement factor H-related protein 2 GN=CFHR2 PE=1 SV=1	V9GYE7_HUMAN	28,911.80	99.80%	1	1	3	0.00213%	8.66%
Pap test	Complement factor I GN=CFI PE=1 SV=2	CFAI_HUMAN	65,059.10	100.00%	8	8	11	0.01720%	14.60%
Swab	Complement factor I GN=CFI PE=1 SV=2	CFAI_HUMAN	65,059.10	100.00%	11	17	37	0.02620%	25.40%
Tumor tissue	Complement factor I GN=CFI PE=1 SV=2	CFAI_HUMAN	65,059.10	100.00%	1	1	1	0.00077%	2.23%
Tumor tissue	Condensin complex subunit 1 GN=NCAPD2 PE=1 SV=3	CND1_HUMAN	157,186.30	100.00%	5	5	5	0.00383%	6.50%
Tumor tissue	Condensin complex subunit 3 GN=NCAPG PE=1 SV=1	CND3_HUMAN	114,338.40	100.00%	3	3	3	0.00230%	3.74%

Biological sample	Protein name	Protein accession numbers	Protein molecular weight (Da)	Protein identification probability	Exclusive unique peptide count	Exclusive unique spectrum count	Total spectrum count	Percentage of total spectra	Percentage sequence coverage
Tumor tissue	Conserved oligomeric Golgi complex subunit 1 GN=COG1 PE=1 SV=1	COG1_HUMAN	107,272.00	100.00%	2	2	2	0.00153%	4.15%
Tumor tissue	Conserved oligomeric Golgi complex subunit 3 GN=COG3 PE=1 SV=3	sp|Q96JB2|COG3_HUMAN	94,099.60	100.00%	2	2	2	0.00153%	3.38%
Tumor tissue	Conserved oligomeric Golgi complex subunit 4 GN=COG4 PE=1 SV=1	sp|Q9H9E3|COG4_HUMAN	89,503.30	100.00%	4	4	4	0.00306%	6.51%
Tumor tissue	Conserved oligomeric Golgi complex subunit 7 GN=COG7 PE=1 SV=1	COG7_HUMAN	86,346.50	100.00%	3	3	3	0.00230%	5.97%
Tumor tissue	Constitutive coactivator of PPAR-gamma-like protein 2 GN=FAM120C PE=2 SV=3	sp|Q9NX05|F120C_HUMAN	120,589.00	100.00%	2	2	2	0.00153%	2.55%
Tumor tissue	Contactin-1 GN=CNTN1 PE=1 SV=1	sp|Q12860|CNTN1_HUMAN	113,322.80	100.00%	2	2	2	0.00153%	4.22%
Tumor tissue	COP9 signalosome complex subunit 1 GN=GPS1 PE=1 SV=1	A8K070_HUMAN	58,924.00	100.00%	1	1	7	0.00536%	18.10%
Tumor tissue	COP9 signalosome complex subunit 1 GN=GPS1 PE=1 SV=2	sp|Q13098|CSN1_HUMAN	53,373.80	100.00%	1	1	7	0.00536%	18.90%
Tumor tissue	COP9 signalosome complex subunit 3 GN=COPS3 PE=1 SV=3	sp|Q9UNS2|CSN3_HUMAN	47,874.50	100.00%	3	4	5	0.00383%	14.40%
Swab	COP9 signalosome complex subunit 4 GN=COPS4 PE=1 SV=1	sp|Q9BT78|CSN4_HUMAN	46,270.20	99.90%	1	1	1	0.00071%	4.19%
Tumor tissue	COP9 signalosome complex subunit 4 GN=COPS4 PE=1 SV=1	sp|Q9BT78|CSN4_HUMAN	46,270.20	100.00%	8	10	12	0.00919%	26.80%
Tumor tissue	COP9 signalosome complex subunit 5 GN=COPS5 PE=1 SV=4	CSN5_HUMAN	37,579.90	100.00%	5	6	6	0.00459%	17.10%
Tumor tissue	COP9 signalosome complex subunit 6 GN=COPS6 PE=1 SV=1	CSN6_HUMAN	36,064.40	100.00%	3	4	4	0.00306%	17.40%
Tumor tissue	COP9 signalosome complex subunit 7a GN=COPS7A PE=1 SV=1	CSN7A_HUMAN	30,276.80	100.00%	3	3	3	0.00230%	13.10%
Tumor tissue	COP9 signalosome complex subunit 7b GN=COPS7B PE=1 SV=1	sp|Q9H9Q2|CSN7B_HUMAN	26,425.00	100.00%	2	2	4	0.00306%	23.50%

Biological sample	Protein name	Protein accession numbers	Protein molecular weight (Da)	Protein identification probability	Exclusive unique peptide count	Exclusive unique spectrum count	Total spectrum count	Percentage of total spectra	Percentage sequence coverage
Tumor tissue	COP9 signalosome complex subunit 8 GN=COPS8 PE=1 SV=1	sp|Q99627|CSN8_HUMAN	19,344.70	100.00%	3	3	3	0.00230%	28.90%
Tumor tissue	Copine-1 GN=CPNE1 PE=1 SV=1	CPNE1_HUMAN	59,717.60	100.00%	5	5	8	0.00612%	15.30%
Tumor tissue	Copine-2 GN=CPNE2 PE=1 SV=3	sp|Q96FN4|CPNE2_HUMAN	61,191.60	100.00%	3	4	6	0.00459%	8.94%
Pap test	Copine-3 GN=CPNE3 PE=1 SV=1	CPNE3_HUMAN	60,131.60	100.00%	5	5	8	0.01250%	14.30%
Swab	Copine-3 GN=CPNE3 PE=1 SV=1	CPNE3_HUMAN	60,131.60	99.80%	1	1	3	0.00213%	6.15%
Tumor tissue	Copine-3 GN=CPNE3 PE=1 SV=1	CPNE3_HUMAN	60,131.60	100.00%	5	6	12	0.00919%	11.50%
Pap test	Copper chaperone for superoxide dismutase GN=CCS PE=1 SV=1	CCS_HUMAN	29,040.10	99.20%	1	1	1	0.00156%	4.74%
Swab	Copper chaperone for superoxide dismutase GN=CCS PE=1 SV=1	CCS_HUMAN	29,040.10	100.00%	2	2	3	0.00213%	7.66%
Tumor tissue	Copper chaperone for superoxide dismutase GN=CCS PE=1 SV=1	CCS_HUMAN	29,040.10	100.00%	2	2	2	0.00153%	7.66%
Pap test	Cordon-bleu protein-like 1 (Fragment) GN=COBLL1 PE=1 SV=1	sp|Q53SF7-2|COBL1_HUMAN	127,945.60	99.80%	1	1	1	0.00156%	1.37%
Tumor tissue	Cordon-bleu protein-like 1 (Fragment) GN=COBLL1 PE=1 SV=1	sp|Q53SF7-2|COBL1_HUMAN	127,945.60	100.00%	2	2	2	0.00153%	2.40%
Tumor tissue	Core histone macro-H2A.2 GN=H2AFY2 PE=1 SV=3	H2AW_HUMAN	40,060.10	100.00%	8	11	17	0.01300%	34.10%
Pap test	Cornifin-A GN=SPRR1A PE=1 SV=2	SPR1A_HUMAN	9,876.50	99.90%	1	2	11	0.01720%	43.80%
Pap test	Cornifin-B GN=SPRR1B PE=1 SV=2	SPR1B_HUMAN	9,886.70	99.90%	1	2	10	0.01560%	43.80%
Swab	Cornifin-B GN=SPRR1B PE=1 SV=2	SPR1B_HUMAN	9,886.70	99.90%	1	1	4	0.00283%	43.80%
Pap test	Cornulin GN=CRNN PE=1 SV=1	CRNN_HUMAN	53,530.10	100.00%	13	21	40	0.06240%	45.70%
Swab	Cornulin GN=CRNN PE=1 SV=1	CRNN_HUMAN	53,530.10	100.00%	10	12	16	0.01130%	40.80%
Pap test	Coronin-1A GN=CORO1A PE=1 SV=4	COR1A_HUMAN	51,026.10	100.00%	2	3	6	0.00936%	11.10%
Swab	Coronin-1A GN=CORO1A PE=1 SV=4	COR1A_HUMAN	51,026.10	100.00%	9	9	29	0.02060%	32.30%
Tumor tissue	Coronin-1A GN=CORO1A PE=1 SV=4	COR1A_HUMAN	51,026.10	100.00%	6	7	15	0.01150%	14.50%
Pap test	Coronin-1B GN=CORO1B PE=1 SV=1	COR1B_HUMAN	54,234.90	100.00%	3	3	3	0.00468%	6.95%
Swab	Coronin-1B GN=CORO1B PE=1 SV=1	COR1B_HUMAN	54,234.90	100.00%	3	4	8	0.00567%	11.00%
Tumor tissue	Coronin-1B GN=CORO1B PE=1 SV=1	COR1B_HUMAN	54,234.90	100.00%	6	9	13	0.00995%	16.60%

Biological sample	Protein name	Protein accession numbers	Protein molecular weight (Da)	Protein identification probability	Exclusive unique peptide count	Exclusive unique spectrum count	Total spectrum count	Percentage of total spectra	Percentage sequence coverage
Pap test	Corticosteroid-binding globulin GN=SERPINA6 PE=1 SV=1	CBG_HUMAN	45,142.10	100.00%	4	5	12	0.01870%	10.90%
Swab	Corticosteroid-binding globulin GN=SERPINA6 PE=1 SV=1	CBG_HUMAN	45,142.10	100.00%	4	5	16	0.01130%	10.90%
Tumor tissue	Corticosteroid-binding globulin GN=SERPINA6 PE=1 SV=1	CBG_HUMAN	45,142.10	100.00%	3	3	3	0.00230%	10.90%
Pap test	Costars family protein ABRACL GN=ABRACL PE=1 SV=1	ABRAL_HUMAN	9,056.70	100.00%	2	2	2	0.00312%	35.80%
Swab	Costars family protein ABRACL GN=ABRACL PE=1 SV=1	ABRAL_HUMAN	9,056.70	99.60%	1	1	2	0.00142%	19.80%
Pap test	C-reactive protein GN=CRP PE=1 SV=1	sp|P02741|CRP_HUMAN	25,039.00	100.00%	1	1	2	0.00312%	4.46%
Swab	C-reactive protein GN=CRP PE=1 SV=1	sp|P02741|CRP_HUMAN	25,039.00	100.00%	4	5	9	0.00638%	17.90%
Pap test	Creatine kinase B-type GN=CKB PE=1 SV=1	KCRB_HUMAN	42,645.10	99.20%	1	1	2	0.00312%	2.62%
Swab	Creatine kinase B-type GN=CKB PE=1 SV=1	KCRB_HUMAN	42,645.10	100.00%	5	5	7	0.00496%	18.60%
Tumor tissue	Creatine kinase B-type GN=CKB PE=1 SV=1	KCRB_HUMAN	42,645.10	100.00%	10	20	27	0.02070%	34.60%
Pap test	Creatine kinase U-type, mitochondrial GN=CKMT1A PE=1 SV=1	sp|P12532|KCRU_HUMAN	47,038.00	100.00%	3	4	5	0.00780%	14.10%
Pap test	Crk-like protein GN=CRKL PE=1 SV=1	CRKL_HUMAN	33,777.30	100.00%	3	3	3	0.00468%	14.90%
Tumor tissue	Crk-like protein GN=CRKL PE=1 SV=1	CRKL_HUMAN	33,777.30	100.00%	7	7	7	0.00536%	26.70%
Tumor tissue	Crooked neck-like protein 1 GN=CRNKL1 PE=1 SV=4	sp|Q9BZJ0|CRNL1_HUMAN	100,454.20	100.00%	2	2	2	0.00153%	2.95%
Tumor tissue	CTP synthase 1 GN=CTPS1 PE=1 SV=2	sp|P17812|PYRG1_HUMAN	66,690.70	100.00%	4	5	9	0.00689%	13.90%
Tumor tissue	CTP synthase 2 GN=CTPS2 PE=1 SV=1	PYRG2_HUMAN	65,679.40	100.00%	5	5	9	0.00689%	16.00%
Tumor tissue	C-type mannose receptor 2 GN=MRC2 PE=1 SV=2	MRC2_HUMAN	166,674.50	100.00%	6	7	12	0.00919%	6.56%
Swab	Cullin-1 GN=CUL1 PE=1 SV=2	CUL1_HUMAN	89,681.40	99.90%	1	1	1	0.00071%	1.68%

Biological sample	Protein name	Protein accession numbers	Protein molecular weight (Da)	Protein identification probability	Exclusive unique peptide count	Exclusive unique spectrum count	Total spectrum count	Percentage of total spectra	Percentage sequence coverage
Tumor tissue	Cullin-1 GN=CUL1 PE=1 SV=2	CUL1_HUMAN	89,681.40	100.00%	4	4	5	0.00383%	6.44%
Tumor tissue	Cullin-2 GN=CUL2 PE=1 SV=1	sp|Q13617|CUL2_HUMAN	88,508.10	100.00%	9	12	13	0.00995%	15.80%
Tumor tissue	Cullin-4A GN=CUL4A PE=1 SV=3	sp|Q13619|CUL4A_HUMAN	87,683.70	100.00%	3	3	9	0.00689%	9.75%
Tumor tissue	Cullin-4B GN=CUL4B PE=1 SV=4	sp|Q13620|CUL4B_HUMAN	103,986.60	100.00%	1	1	7	0.00536%	4.93%
Tumor tissue	Cullin-5 GN=CUL5 PE=1 SV=4	CUL5_HUMAN	90,959.20	100.00%	5	5	5	0.00383%	6.41%
Swab	Cullin-associated NEDD8-dissociated protein 1 GN=CAND1 PE=1 SV=2	sp|Q86VP6|CAND1_HUMAN	136,381.10	100.00%	5	5	24	0.01700%	13.90%
Tumor tissue	Cullin-associated NEDD8-dissociated protein 1 GN=CAND1 PE=1 SV=2	sp|Q86VP6|CAND1_HUMAN	136,381.10	100.00%	3	4	37	0.02830%	17.30%
Tumor tissue	Cullin-associated NEDD8-dissociated protein 2 GN=CAND2 PE=1 SV=3	sp|O75155|CAND2_HUMAN	135,260.30	100.00%	2	2	4	0.00306%	4.13%
Tumor tissue	Cyclin-dependent kinase 1 GN=CDK1 PE=1 SV=3	sp|P06493|CDK1_HUMAN	34,097.30	100.00%	1	1	6	0.00459%	16.20%
Tumor tissue	Cyclin-dependent kinase 2 GN=CDK2 PE=1 SV=1	G3V5T9_HUMAN	39,179.30	100.00%	2	3	7	0.00536%	15.90%
Tumor tissue	Cyclin-dependent kinase 6 GN=CDK6 PE=1 SV=1	CDK6_HUMAN	36,938.80	100.00%	2	2	5	0.00383%	12.00%
Tumor tissue	Cyclin-dependent-like kinase 5 GN=CDK5 PE=1 SV=3	sp|Q00535|CDK5_HUMAN	33,305.80	100.00%	2	2	5	0.00383%	11.60%
Tumor tissue	Cyclin-G-associated kinase GN=GAK PE=1 SV=2	sp|O14976|GAK_HUMAN	143,192.40	100.00%	7	9	10	0.00766%	7.48%
Tumor tissue	Cyclin-Y GN=CCNY PE=1 SV=2	sp|Q8ND76|CCNY_HUMAN	39,338.40	100.00%	2	2	3	0.00230%	12.60%
Pap test	Cystatin-A GN=CSTA PE=1 SV=1	CYTA_HUMAN	11,006.60	100.00%	1	2	3	0.00468%	18.40%
Swab	Cystatin-A GN=CSTA PE=1 SV=1	CYTA_HUMAN	11,006.60	100.00%	2	2	3	0.00213%	40.80%
Pap test	Cystatin-B GN=CSTB PE=1 SV=2	CYTB_HUMAN	11,139.30	100.00%	4	12	38	0.05930%	70.40%
Swab	Cystatin-B GN=CSTB PE=1 SV=2	CYTB_HUMAN	11,139.30	100.00%	5	13	46	0.03260%	79.60%

Biological sample	Protein name	Protein accession numbers	Protein molecular weight (Da)	Protein identification probability	Exclusive unique peptide count	Exclusive unique spectrum count	Total spectrum count	Percentage of total spectra	Percentage sequence coverage
Tumor tissue	Cystatin-B GN=CSTB PE=1 SV=2	CYTB_HUMAN	11,139.30	100.00%	3	8	16	0.01220%	45.90%
Pap test	Cystatin-C GN=CST3 PE=1 SV=1	CYTC_HUMAN	15,799.20	100.00%	2	2	2	0.00312%	18.50%
Swab	Cystatin-C GN=CST3 PE=1 SV=1	CYTC_HUMAN	15,799.20	100.00%	4	7	12	0.00850%	30.80%
Tumor tissue	Cystatin-C GN=CST3 PE=1 SV=1	CYTC_HUMAN	15,799.20	99.90%	1	1	1	0.00077%	11.00%
Tumor tissue	Cysteine and glycine-rich protein 1 GN=CSRP1 PE=1 SV=3	CSRP1_HUMAN	20,567.10	100.00%	3	8	13	0.00995%	20.20%
Tumor tissue	Cysteine-rich protein 2 (Fragment) GN=CRIP2 PE=1 SV=1	H0YHD8_HUMAN	11,159.40	100.00%	2	3	4	0.00306%	18.30%
Pap test	Cysteine-rich secretory protein 3 GN=CRISP3 PE=1 SV=1	sp|P54108|CRIS3_HUMAN	30,974.80	100.00%	3	4	5	0.00780%	16.30%
Swab	Cysteine-rich secretory protein 3 GN=CRISP3 PE=1 SV=1	sp|P54108|CRIS3_HUMAN	30,974.80	100.00%	4	4	6	0.00425%	19.20%
Tumor tissue	Cysteine-rich secretory protein LCCL domain-containing 2 GN=CRISPLD2 PE=1 SV=1	sp|Q9H0B8|CRLD2_HUMAN	55,789.60	100.00%	2	2	2	0.00153%	6.25%
Pap test	Cytidine deaminase GN=CDA PE=1 SV=2	CDD_HUMAN	16,184.70	100.00%	2	2	2	0.00312%	28.10%
Swab	Cytidine deaminase GN=CDA PE=1 SV=2	CDD_HUMAN	16,184.70	100.00%	4	7	8	0.00567%	47.30%
Pap test	Cytochrome b-245 heavy chain GN=CYBB PE=1 SV=2	CY24B_HUMAN	65,337.20	99.20%	1	1	1	0.00156%	2.98%
Swab	Cytochrome b-245 heavy chain GN=CYBB PE=1 SV=2	CY24B_HUMAN	65,337.20	99.20%	1	1	1	0.00071%	1.75%
Tumor tissue	Cytochrome b-245 heavy chain GN=CYBB PE=1 SV=2	CY24B_HUMAN	65,337.20	100.00%	3	3	3	0.00230%	5.44%
Tumor tissue	Cytochrome b-245 light chain GN=CYBA PE=1 SV=3	CY24A_HUMAN	21,012.80	100.00%	2	3	3	0.00230%	21.00%
Tumor tissue	Cytochrome b5 type B GN=CYB5B PE=1 SV=2	CYB5B_HUMAN	16,694.80	100.00%	4	4	4	0.00306%	50.70%
Pap test	Cytochrome b-c1 complex subunit 1, mitochondrial GN=UQCRC1 PE=1 SV=3	QCR1_HUMAN	52,645.90	100.00%	3	3	3	0.00468%	10.60%

Biological sample	Protein name	Protein accession numbers	Protein molecular weight (Da)	Protein identification probability	Exclusive unique peptide count	Exclusive unique spectrum count	Total spectrum count	Percentage of total spectra	Percentage sequence coverage
Tumor tissue	Cytochrome b-c1 complex subunit 1, mitochondrial GN=UQCRC1 PE=1 SV=3	QCR1_HUMAN	52,645.90	100.00%	6	9	10	0.00766%	16.90%
Tumor tissue	Cytochrome b-c1 complex subunit 2, mitochondrial GN=UQCRC2 PE=1 SV=1	H3BSJ9_HUMAN	36,389.60	99.80%	1	1	31	0.02370%	50.30%
Pap test	Cytochrome b-c1 complex subunit 2, mitochondrial GN=UQCRC2 PE=1 SV=3	QCR2_HUMAN	48,443.40	100.00%	1	1	5	0.00780%	13.00%
Tumor tissue	Cytochrome b-c1 complex subunit 2, mitochondrial GN=UQCRC2 PE=1 SV=3	QCR2_HUMAN	48,443.40	100.00%	1	1	31	0.02370%	36.20%
Pap test	Cytochrome b-c1 complex subunit 7 GN=UQCRB PE=1 SV=2	sp|P14927|QCR7_HUMAN	13,531.30	99.90%	1	1	1	0.00156%	11.70%
Tumor tissue	Cytochrome b-c1 complex subunit 7 GN=UQCRB PE=1 SV=2	sp|P14927|QCR7_HUMAN	13,531.30	100.00%	2	3	3	0.00230%	25.20%
Pap test	Cytochrome b-c1 complex subunit Rieske, mitochondrial GN=UQCRFS1 PE=1 SV=2	UCRI_HUMAN	29,667.80	99.20%	1	1	1	0.00156%	5.11%
Tumor tissue	Cytochrome b-c1 complex subunit Rieske, mitochondrial GN=UQCRFS1 PE=1 SV=2	UCRI_HUMAN	29,667.80	100.00%	7	11	17	0.01300%	40.50%
Pap test	Cytochrome c (Fragment) GN=CYCS PE=1 SV=1	CYC_HUMAN	11,334.00	100.00%	2	3	7	0.01090%	42.60%
Swab	Cytochrome c (Fragment) GN=CYCS PE=1 SV=1	CYC_HUMAN	11,334.00	100.00%	1	1	6	0.00425%	28.70%
Tumor tissue	Cytochrome c (Fragment) GN=CYCS PE=1 SV=1	CYC_HUMAN	11,334.00	100.00%	3	3	7	0.00536%	43.60%
Pap test	Cytochrome c oxidase subunit 2 GN=MT-CO2 PE=1 SV=1	COX2_HUMAN	25,566.40	99.90%	1	1	2	0.00312%	4.41%
Tumor tissue	Cytochrome c oxidase subunit 2 GN=MT-CO2 PE=1 SV=1	COX2_HUMAN	25,566.40	100.00%	3	5	10	0.00766%	20.30%
Pap test	Cytochrome c oxidase subunit 4 isoform 1, mitochondrial GN=COX4I1 PE=1 SV=1	COX41_HUMAN	19,576.80	98.70%	1	1	1	0.00156%	7.10%

Biological sample	Protein name	Protein accession numbers	Protein molecular weight (Da)	Protein identification probability	Exclusive unique peptide count	Exclusive unique spectrum count	Total spectrum count	Percentage of total spectra	Percentage sequence coverage
Tumor tissue	Cytochrome c oxidase subunit 4 isoform 1, mitochondrial GN=COX4I1 PE=1 SV=1	COX41_HUMAN	19,576.80	100.00%	6	7	8	0.00612%	38.50%
Tumor tissue	Cytochrome c oxidase subunit 5A, mitochondrial GN=COX5A PE=1 SV=2	COX5A_HUMAN	16,762.50	100.00%	2	3	4	0.00306%	16.00%
Pap test	Cytochrome c oxidase subunit 5B, mitochondrial GN=COX5B PE=1 SV=2	COX5B_HUMAN	13,695.70	99.30%	1	1	2	0.00312%	6.20%
Tumor tissue	Cytochrome c oxidase subunit 5B, mitochondrial GN=COX5B PE=1 SV=2	COX5B_HUMAN	13,695.70	100.00%	4	6	7	0.00536%	34.90%
Tumor tissue	Cytochrome c oxidase subunit 6C GN=COX6C PE=1 SV=2	COX6C_HUMAN	8,781.80	100.00%	2	3	3	0.00230%	28.00%
Pap test	Cytochrome c1, heme protein, mitochondrial GN=CYC1 PE=1 SV=3	CY1_HUMAN	35,422.80	99.20%	1	1	1	0.00156%	3.69%
Tumor tissue	Cytochrome c1, heme protein, mitochondrial GN=CYC1 PE=1 SV=3	CY1_HUMAN	35,422.80	100.00%	4	8	15	0.01150%	20.90%
Pap test	Cytoplasmic aconitate hydratase GN=ACO1 PE=1 SV=3	ACOC_HUMAN	98,401.00	99.90%	1	1	1	0.00156%	1.57%
Swab	Cytoplasmic aconitate hydratase GN=ACO1 PE=1 SV=3	ACOC_HUMAN	98,401.00	100.00%	3	5	6	0.00425%	5.06%
Tumor tissue	Cytoplasmic aconitate hydratase GN=ACO1 PE=1 SV=3	ACOC_HUMAN	98,401.00	100.00%	13	16	21	0.01610%	21.00%
Swab	Cytoplasmic dynein 1 heavy chain 1 GN=DYNC1H1 PE=1 SV=5	DYHC1_HUMAN	532,405.60	100.00%	3	3	5	0.00354%	0.86%
Tumor tissue	Cytoplasmic dynein 1 heavy chain 1 GN=DYNC1H1 PE=1 SV=5	DYHC1_HUMAN	532,405.60	100.00%	96	127	154	0.11800%	26.10%
Tumor tissue	Cytoplasmic dynein 1 light intermediate chain 1 GN=DYNC1LI1 PE=1 SV=3	DC1L1_HUMAN	56,580.20	100.00%	3	3	3	0.00230%	8.03%
Pap test	Cytoplasmic FMR1-interacting protein 1 GN=CYFIP1 PE=1 SV=1	sp|Q7L576|CYFP1_HUMAN	145,186.70	99.50%	1	1	1	0.00156%	0.72%
Tumor tissue	Cytoplasmic FMR1-interacting protein 1 GN=CYFIP1 PE=1 SV=1	sp|Q7L576|CYFP1_HUMAN	145,186.70	100.00%	7	8	13	0.00995%	12.50%

Biological sample	Protein name	Protein accession numbers	Protein molecular weight (Da)	Protein identification probability	Exclusive unique peptide count	Exclusive unique spectrum count	Total spectrum count	Percentage of total spectra	Percentage sequence coverage
Tumor tissue	Cytoplasmic FMR1-interacting protein 1 (Fragment) GN=CYFIP1 PE=1 SV=4	A0A0G2JR96_HUMAN	100,300.30	99.90%	1	1	4	0.00306%	6.61%
Tumor tissue	Cytoplasmic protein NCK1 GN=NCK1 PE=1 SV=1	sp|P16333|NCK1_HUMAN	42,864.40	100.00%	4	4	4	0.00306%	15.90%
Pap test	Cytoskeleton-associated protein 4 GN=CKAP4 PE=1 SV=2	CKAP4_HUMAN	66,022.20	99.70%	1	1	1	0.00156%	1.99%
Tumor tissue	Cytoskeleton-associated protein 4 GN=CKAP4 PE=1 SV=2	CKAP4_HUMAN	66,022.20	100.00%	25	44	76	0.05820%	51.20%
Pap test	Cytosol aminopeptidase GN=LAP3 PE=1 SV=3	sp|P28838|AMPL_HUMAN	56,167.80	99.50%	1	1	1	0.00156%	2.31%
Swab	Cytosol aminopeptidase GN=LAP3 PE=1 SV=3	sp|P28838|AMPL_HUMAN	56,167.80	100.00%	11	13	15	0.01060%	22.70%
Tumor tissue	Cytosol aminopeptidase GN=LAP3 PE=1 SV=3	sp|P28838|AMPL_HUMAN	56,167.80	100.00%	19	29	45	0.03440%	44.30%
Tumor tissue	Cytosolic Fe-S cluster assembly factor NUBP2 GN=NUBP2 PE=1 SV=1	NUBP2_HUMAN	26,381.00	100.00%	1	1	3	0.00230%	12.70%
Pap test	Cytosolic non-specific dipeptidase GN=CNDP2 PE=1 SV=2	sp|Q96KP4|CNDP2_HUMAN	52,879.70	100.00%	6	7	10	0.01560%	17.10%
Swab	Cytosolic non-specific dipeptidase GN=CNDP2 PE=1 SV=2	sp|Q96KP4|CNDP2_HUMAN	52,879.70	100.00%	11	15	34	0.02410%	37.30%
Tumor tissue	Cytosolic non-specific dipeptidase GN=CNDP2 PE=1 SV=2	sp|Q96KP4|CNDP2_HUMAN	52,879.70	100.00%	15	22	42	0.03220%	43.80%
Pap test	Cytosolic purine 5'-nucleotidase GN=NT5C2 PE=1 SV=1	sp|P49902|5NTC_HUMAN	64,972.50	100.00%	1	1	2	0.00312%	4.10%
Swab	Cytosolic purine 5'-nucleotidase GN=NT5C2 PE=1 SV=1	sp|P49902|5NTC_HUMAN	64,972.50	99.50%	1	1	1	0.00071%	1.78%
Tumor tissue	Cytosolic purine 5'-nucleotidase GN=NT5C2 PE=1 SV=1	sp|P49902|5NTC_HUMAN	64,972.50	100.00%	4	4	5	0.00383%	11.20%
Tumor tissue	Cytospin-B GN=SPECC1 PE=1 SV=1	sp|Q5M775|CYTSB_HUMAN	118,587.60	100.00%	2	2	2	0.00153%	1.78%
Pap test	D-3-phosphoglycerate dehydrogenase GN=PHGDH PE=1 SV=4	SERA_HUMAN	56,650.60	100.00%	5	5	6	0.00936%	11.10%

Biological sample	Protein name	Protein accession numbers	Protein molecular weight (Da)	Protein identification probability	Exclusive unique peptide count	Exclusive unique spectrum count	Total spectrum count	Percentage of total spectra	Percentage sequence coverage
Swab	D-3-phosphoglycerate dehydrogenase GN=PHGDH PE=1 SV=4	SERA_HUMAN	56,650.60	100.00%	8	9	11	0.00780%	19.30%
Tumor tissue	D-3-phosphoglycerate dehydrogenase GN=PHGDH PE=1 SV=4	SERA_HUMAN	56,650.60	100.00%	7	8	12	0.00919%	14.10%
Tumor tissue	DBIRD complex subunit ZNF326 GN=ZNF326 PE=1 SV=2	sp|Q5BKZ1|ZN326_HUMAN	65,653.50	100.00%	2	2	2	0.00153%	4.81%
Tumor tissue	DCC-interacting protein 13-alpha GN=APPL1 PE=1 SV=1	DP13A_HUMAN	79,665.10	100.00%	6	8	9	0.00689%	12.00%
Tumor tissue	DDB1- and CUL4-associated factor 7 (Fragment) GN=DCAF7 PE=1 SV=6	DCAF7_HUMAN	38,926.30	100.00%	2	3	3	0.00230%	13.60%
Pap test	D-dopachrome decarboxylase GN=DDT PE=1 SV=1	sp|P30046|DOPD_HUMAN	14,193.20	100.00%	1	1	5	0.00780%	17.40%
Tumor tissue	D-dopachrome decarboxylase GN=DDT PE=1 SV=1	sp|P30046|DOPD_HUMAN	14,193.20	99.90%	1	1	9	0.00689%	17.40%
Tumor tissue	DDRGK domain-containing protein 1 GN=DDRGK1 PE=1 SV=2	sp|Q96HY6|DDRGK_HUMAN	35,611.00	100.00%	5	7	7	0.00536%	25.80%
Tumor tissue	Death-associated protein kinase 3 GN=DAPK3 PE=1 SV=1	sp|O43293|DAPK3_HUMAN	52,537.90	100.00%	2	2	2	0.00153%	5.29%
Pap test	Death-inducer obliterator 1 GN=DIDO1 PE=1 SV=5	sp|Q9BTC0|DIDO1_HUMAN	243,873.90	99.50%	1	1	3	0.00468%	0.80%
Swab	Death-inducer obliterator 1 GN=DIDO1 PE=1 SV=5	sp|Q9BTC0|DIDO1_HUMAN	243,873.90	100.00%	1	1	2	0.00142%	0.71%
Tumor tissue	Death-inducer obliterator 1 GN=DIDO1 PE=1 SV=5	sp|Q9BTC0|DIDO1_HUMAN	243,873.90	100.00%	2	2	2	0.00153%	1.34%
Tumor tissue	Decorin GN=DCN PE=1 SV=1	sp|P07585|PGS2_HUMAN	39,748.20	100.00%	2	4	39	0.02990%	24.80%
Tumor tissue	Dedicator of cytokinesis protein 1 GN=DOCK1 PE=1 SV=1	DOCK1_HUMAN	217,760.10	100.00%	2	2	2	0.00153%	1.80%
Tumor tissue	Dedicator of cytokinesis protein 11 GN=DOCK11 PE=1 SV=2	DOC11_HUMAN	238,105.60	100.00%	5	5	5	0.00383%	3.90%
Tumor tissue	Dedicator of cytokinesis protein 2 GN=DOCK2 PE=1 SV=1	E5RFJ0_HUMAN	119,225.10	100.00%	4	4	5	0.00383%	6.98%
Tumor tissue	Dedicator of cytokinesis protein 2 GN=DOCK2 PE=1 SV=1	E7ERW7_HUMAN	153,374.10	99.50%	1	1	2	0.00153%	1.89%

Biological sample	Protein name	Protein accession numbers	Protein molecular weight (Da)	Protein identification probability	Exclusive unique peptide count	Exclusive unique spectrum count	Total spectrum count	Percentage of total spectra	Percentage sequence coverage
Tumor tissue	Dedicator of cytokinesis protein 8 GN=DOCK8 PE=1 SV=3	sp|Q8NF50|DOCK8_HUMAN	238,533.80	100.00%	3	3	4	0.00306%	3.29%
Tumor tissue	Dehydrogenase/reductase SDR family member 7B GN=DHRS7B PE=1 SV=1	DRS7B_HUMAN	33,524.90	100.00%	2	2	2	0.00153%	9.35%
Tumor tissue	Delta(24)-sterol reductase GN=Nbla03646 PE=1 SV=1	sp|Q15392|DHC24_HUMAN	49,437.40	100.00%	4	4	4	0.00306%	7.96%
Pap test	Delta(3,5)-Delta(2,4)-dienoyl-CoA isomerase, mitochondrial GN=ECH1 PE=1 SV=2	ECH1_HUMAN	35,816.20	100.00%	4	5	5	0.00780%	14.00%
Tumor tissue	Delta(3,5)-Delta(2,4)-dienoyl-CoA isomerase, mitochondrial GN=ECH1 PE=1 SV=2	ECH1_HUMAN	35,816.20	100.00%	7	10	14	0.01070%	30.50%
Tumor tissue	Delta-1-pyrroline-5-carboxylate synthase GN=ALDH18A1 PE=1 SV=2	sp|P54886|P5CS_HUMAN	87,302.90	100.00%	15	22	27	0.02070%	27.50%
Pap test	Delta-aminolevulinic acid dehydratase GN=ALAD PE=1 SV=1	sp|P13716|HEM2_HUMAN	36,295.30	100.00%	5	6	8	0.01250%	19.70%
Swab	Delta-aminolevulinic acid dehydratase GN=ALAD PE=1 SV=1	sp|P13716|HEM2_HUMAN	36,295.30	100.00%	4	4	6	0.00425%	15.20%
Tumor tissue	Delta-aminolevulinic acid dehydratase GN=ALAD PE=1 SV=1	sp|P13716|HEM2_HUMAN	36,295.30	100.00%	6	6	7	0.00536%	21.50%
Tumor tissue	Deoxyhypusine hydroxylase GN=DOHH PE=1 SV=1	DOHH_HUMAN	24,385.70	100.00%	2	2	3	0.00230%	10.90%
Swab	Deoxynucleoside triphosphate triphosphohydrolase SAMHD1 GN=SAMHD1 PE=1 SV=2	sp|Q9Y3Z3|SAMH1_HUMAN	72,202.70	100.00%	4	4	4	0.00283%	7.35%
Tumor tissue	Deoxynucleoside triphosphate triphosphohydrolase SAMHD1 GN=SAMHD1 PE=1 SV=2	sp|Q9Y3Z3|SAMH1_HUMAN	72,202.70	100.00%	20	31	37	0.02830%	38.00%
Tumor tissue	Deoxynucleotidyltransferase terminal-interacting protein 2 GN=DNTTIP2 PE=1 SV=2	TDIF2_HUMAN	84,471.10	100.00%	2	2	2	0.00153%	4.23%
Tumor tissue	Deoxyribose-phosphate aldolase GN=DERA PE=1 SV=2	DEOC_HUMAN	35,231.80	100.00%	2	2	6	0.00459%	24.50%

Biological sample	Protein name	Protein accession numbers	Protein molecular weight (Da)	Protein identification probability	Exclusive unique peptide count	Exclusive unique spectrum count	Total spectrum count	Percentage of total spectra	Percentage sequence coverage
Tumor tissue	Deoxyuridine 5'-triphosphate nucleotidohydrolase, mitochondrial GN=DUT PE=1 SV=1	H0YKI0_HUMAN	23,712.50	100.00%	1	1	5	0.00383%	24.30%
Tumor tissue	Deoxyuridine 5'-triphosphate nucleotidohydrolase, mitochondrial (Fragment) GN=DUT PE=1 SV=1	H0YKC5_HUMAN	23,739.80	100.00%	1	2	6	0.00459%	24.40%
Tumor tissue	Derlin GN=DERL1 PE=1 SV=1	sp|Q9BUN8|DERL1_HUMAN	17,033.70	100.00%	2	2	3	0.00230%	16.60%
Swab	Dermcidin GN=DCD PE=1 SV=2	sp|P81605|DCD_HUMAN	11,284.10	100.00%	3	3	3	0.00213%	27.30%
Tumor tissue	Dermcidin GN=DCD PE=1 SV=2	sp|P81605|DCD_HUMAN	11,284.10	100.00%	2	3	3	0.00230%	25.50%
Tumor tissue	Desmin GN=DES PE=1 SV=3	DESM_HUMAN	53,536.60	100.00%	22	42	138	0.10600%	57.90%
Pap test	Desmocollin-2 GN=DSC2 PE=1 SV=1	sp|Q02487|DSC2_HUMAN	99,962.60	100.00%	2	2	2	0.00312%	3.77%
Swab	Desmocollin-2 GN=DSC2 PE=1 SV=1	sp|Q02487|DSC2_HUMAN	99,962.60	100.00%	1	1	1	0.00071%	2.00%
Pap test	Desmoglein-2 GN=DSG2 PE=1 SV=2	DSG2_HUMAN	122,294.40	99.10%	1	1	1	0.00156%	1.25%
Tumor tissue	Desmoglein-2 GN=DSG2 PE=1 SV=2	DSG2_HUMAN	122,294.40	100.00%	11	12	17	0.01300%	18.10%
Pap test	Desmoglein-3 GN=DSG3 PE=1 SV=2	DSG3_HUMAN	107,534.10	100.00%	4	5	5	0.00780%	6.21%
Pap test	Desmoplakin GN=DSP PE=1 SV=3	sp|P15924|DESP_HUMAN	331,781.40	100.00%	43	48	71	0.11100%	19.20%
Swab	Desmoplakin GN=DSP PE=1 SV=3	sp|P15924|DESP_HUMAN	331,781.40	100.00%	1	1	1	0.00071%	0.31%
Tumor tissue	Desmoplakin GN=DSP PE=1 SV=3	sp|P15924|DESP_HUMAN	331,781.40	100.00%	59	75	94	0.07200%	24.50%
Tumor tissue	Desmuslin, isoform CRA_a GN=SYNM PE=1 SV=1	sp|O15061|SYNEM_HUMAN	140,162.00	100.00%	7	7	12	0.00919%	12.80%
Pap test	Destrin GN=DSTN PE=1 SV=3	sp|P60981|DEST_HUMAN	18,506.20	100.00%	2	3	3	0.00468%	12.10%
Swab	Destrin GN=DSTN PE=1 SV=3	sp|P60981|DEST_HUMAN	18,506.20	100.00%	5	5	7	0.00496%	34.50%
Tumor tissue	Destrin GN=DSTN PE=1 SV=3	sp|P60981|DEST_HUMAN	18,506.20	100.00%	2	2	3	0.00230%	15.80%
Tumor tissue	DET1- and DDB1-associated protein 1 GN=DDA1 PE=1 SV=1	DDA1_HUMAN	11,835.50	100.00%	2	2	2	0.00153%	27.50%
Tumor tissue	Deubiquitinating protein VCIP135 GN=VCPIP1 PE=1 SV=2	VCIP1_HUMAN	134,322.00	100.00%	4	4	4	0.00306%	4.99%

Biological sample	Protein name	Protein accession numbers	Protein molecular weight (Da)	Protein identification probability	Exclusive unique peptide count	Exclusive unique spectrum count	Total spectrum count	Percentage of total spectra	Percentage sequence coverage
Tumor tissue	Developmentally-regulated GTP-binding protein 1 GN=DRG1 PE=1 SV=1	DRG1_HUMAN	40,543.80	100.00%	7	10	11	0.00842%	25.90%
Tumor tissue	Developmentally-regulated GTP-binding protein 2 GN=DRG2 PE=1 SV=1	DRG2_HUMAN	40,747.90	100.00%	2	3	3	0.00230%	8.52%
Tumor tissue	Differentially expressed in FDCP 6 homolog GN=DEF6 PE=1 SV=1	DEFI6_HUMAN	73,910.80	100.00%	2	2	3	0.00230%	5.86%
Swab	Dihydrolipoyl dehydrogenase GN=DLD PE=1 SV=1	sp|P09622|DLDH_HUMAN	54,177.50	100.00%	1	1	1	0.00071%	2.67%
Tumor tissue	Dihydrolipoyl dehydrogenase GN=DLD PE=1 SV=1	sp|P09622|DLDH_HUMAN	51,815.80	100.00%	7	10	17	0.01300%	19.50%
Tumor tissue	Dihydrolipoyllysine-residue acetyltransferase component of pyruvate dehydrogenase complex, mitochondrial GN=DLAT PE=1 SV=3	ODP2_HUMAN	68,997.20	100.00%	8	12	14	0.01070%	16.70%
Pap test	Dihydrolipoyllysine-residue succinyltransferase component of 2-oxoglutarate dehydrogenase complex, mitochondrial GN=DLST PE=1 SV=4	sp|P36957|ODO2_HUMAN	48,755.20	100.00%	2	2	3	0.00468%	10.40%
Tumor tissue	Dihydrolipoyllysine-residue succinyltransferase component of 2-oxoglutarate dehydrogenase complex, mitochondrial GN=DLST PE=1 SV=4	sp|P36957|ODO2_HUMAN	48,755.20	100.00%	6	10	17	0.01300%	20.30%
Swab	Dihydropteridine reductase GN=QDPR PE=1 SV=2	sp|P09417|DHPR_HUMAN	25,789.50	100.00%	3	3	3	0.00213%	18.90%
Tumor tissue	Dihydropteridine reductase GN=QDPR PE=1 SV=2	sp|P09417|DHPR_HUMAN	25,789.50	100.00%	2	4	4	0.00306%	13.90%
Swab	Dihydropyrimidinase-related protein 2 GN=DPYSL2 PE=1 SV=1	A0A1C7CYX9_HUMAN	73,503.00	100.00%	4	6	8	0.00567%	12.00%
Tumor tissue	Dihydropyrimidinase-related protein 2 GN=DPYSL2 PE=1 SV=1	A0A1C7CYX9_HUMAN	73,503.00	100.00%	10	17	43	0.03290%	36.00%
Pap test	Dihydropyrimidine dehydrogenase [NADP(+)] GN=DPYD PE=1 SV=2	sp|Q12882|DPYD_HUMAN	111,404.30	100.00%	12	12	13	0.02030%	15.00%

Biological sample	Protein name	Protein accession numbers	Protein molecular weight (Da)	Protein identification probability	Exclusive unique peptide count	Exclusive unique spectrum count	Total spectrum count	Percentage of total spectra	Percentage sequence coverage
Swab	Dihydropyrimidine dehydrogenase [NADP(+)] GN=DPYD PE=1 SV=2	sp|Q12882|DPYD_HUMAN	111,404.30	100.00%	8	8	8	0.00567%	10.60%
Tumor tissue	Dihydropyrimidine dehydrogenase [NADP(+)] GN=DPYD PE=1 SV=2	sp|Q12882|DPYD_HUMAN	111,404.30	100.00%	10	12	12	0.00919%	15.70%
Swab	Dipeptidyl peptidase 1 GN=CTSC PE=1 SV=2	sp|P53634|CATC_HUMAN	51,854.40	100.00%	3	4	6	0.00425%	11.90%
Tumor tissue	Dipeptidyl peptidase 1 GN=CTSC PE=1 SV=2	sp|P53634|CATC_HUMAN	51,854.40	100.00%	2	2	2	0.00153%	4.97%
Pap test	Dipeptidyl peptidase 2 GN=DPP7 PE=1 SV=3	DPP2_HUMAN	54,342.90	99.20%	1	1	1	0.00156%	2.24%
Swab	Dipeptidyl peptidase 2 GN=DPP7 PE=1 SV=3	DPP2_HUMAN	54,342.90	100.00%	2	2	3	0.00213%	4.27%
Tumor tissue	Dipeptidyl peptidase 2 GN=DPP7 PE=1 SV=3	DPP2_HUMAN	54,342.90	100.00%	3	4	4	0.00306%	7.72%
Pap test	Dipeptidyl peptidase 3 GN=DPP3 PE=1 SV=1	sp|Q9NY33|DPP3_HUMAN	84,378.50	99.10%	1	1	1	0.00156%	1.06%
Swab	Dipeptidyl peptidase 3 GN=DPP3 PE=1 SV=1	sp|Q9NY33|DPP3_HUMAN	84,307.50	100.00%	8	9	11	0.00780%	15.10%
Tumor tissue	Dipeptidyl peptidase 3 GN=DPP3 PE=1 SV=1	sp|Q9NY33|DPP3_HUMAN	84,307.50	100.00%	10	13	14	0.01070%	24.70%
Swab	Dipeptidyl peptidase 4 GN=DPP4 PE=1 SV=2	DPP4_HUMAN	88,281.00	99.90%	1	1	1	0.00071%	1.83%
Tumor tissue	Dipeptidyl peptidase 4 GN=DPP4 PE=1 SV=2	DPP4_HUMAN	88,281.00	100.00%	2	2	2	0.00153%	3.13%
Tumor tissue	Diphosphoinositol polyphosphate phosphohydrolase 1 GN=NUDT3 PE=1 SV=1	NUDT3_HUMAN	19,470.50	100.00%	3	4	4	0.00306%	23.80%
Tumor tissue	Diphosphomevalonate decarboxylase GN=MVD PE=1 SV=1	MVD1_HUMAN	43,404.70	100.00%	2	2	2	0.00153%	5.75%
Tumor tissue	Disabled homolog 2 GN=DAB2 PE=1 SV=3	sp|P98082|DAB2_HUMAN	82,449.10	100.00%	6	6	6	0.00459%	9.35%
Tumor tissue	Disks large homolog 1 GN=DLG1 PE=1 SV=1	sp|Q12959-4|DLG1_HUMAN	99,815.70	100.00%	1	1	11	0.00842%	18.40%
Pap test	DNA damage-binding protein 1 GN=DDB1 PE=1 SV=1	sp|Q16531|DDB1_HUMAN	126,970.20	100.00%	1	1	2	0.00312%	0.88%

Biological sample	Protein name	Protein accession numbers	Protein molecular weight (Da)	Protein identification probability	Exclusive unique peptide count	Exclusive unique spectrum count	Total spectrum count	Percentage of total spectra	Percentage sequence coverage
Swab	DNA damage-binding protein 1 GN=DDB1 PE=1 SV=1	sp|Q16531|DDB1_HUMAN	126,970.20	100.00%	2	2	2	0.00142%	1.75%
Tumor tissue	DNA damage-binding protein 1 GN=DDB1 PE=1 SV=1	sp|Q16531|DDB1_HUMAN	126,970.20	100.00%	19	22	32	0.02450%	21.10%
Tumor tissue	DNA fragmentation factor subunit alpha GN=DFFA PE=1 SV=1	sp|O00273|DFFA_HUMAN	36,522.50	100.00%	1	1	7	0.00536%	27.20%
Tumor tissue	DNA mismatch repair protein Msh2 GN=MSH2 PE=1 SV=1	sp|P43246|MSH2_HUMAN	104,745.80	100.00%	9	10	13	0.00995%	15.00%
Tumor tissue	DNA mismatch repair protein Msh6 GN=MSH6 PE=1 SV=2	sp|P52701|MSH6_HUMAN	152,789.90	100.00%	8	8	8	0.00612%	8.24%
Tumor tissue	DNA polymerase delta catalytic subunit GN=POLD1 PE=1 SV=2	DPOD1_HUMAN	126,390.70	100.00%	3	3	3	0.00230%	3.52%
Tumor tissue	DNA repair protein complementing XP-C cells GN=XPC PE=1 SV=4	sp|Q01831|XPC_HUMAN	105,955.00	100.00%	1	1	3	0.00230%	4.57%
Tumor tissue	DNA replication licensing factor MCM2 GN=MCM2 PE=1 SV=4	MCM2_HUMAN	101,898.10	100.00%	10	11	14	0.01070%	14.90%
Tumor tissue	DNA replication licensing factor MCM4 GN=MCM4 PE=1 SV=5	MCM4_HUMAN	96,560.60	100.00%	13	15	15	0.01150%	19.60%
Tumor tissue	DNA replication licensing factor MCM5 GN=MCM5 PE=1 SV=5	MCM5_HUMAN	82,288.30	100.00%	13	15	16	0.01220%	26.60%
Tumor tissue	DNA replication licensing factor MCM6 GN=MCM6 PE=1 SV=1	MCM6_HUMAN	92,890.60	100.00%	12	14	14	0.01070%	20.60%
Tumor tissue	DNA replication licensing factor MCM7 GN=MCM7 PE=1 SV=4	sp|P33993|MCM7_HUMAN	81,309.00	100.00%	17	21	25	0.01910%	35.30%
Tumor tissue	DNA topoisomerase 1 GN=TOP1 PE=1 SV=2	TOP1_HUMAN	90,729.70	100.00%	6	6	11	0.00842%	13.20%
Pap test	DNA-(apurinic or apyrimidinic site) lyase GN=APEX1 PE=1 SV=2	APEX1_HUMAN	35,555.70	100.00%	3	3	3	0.00468%	15.10%
Tumor tissue	DNA-(apurinic or apyrimidinic site) lyase GN=APEX1 PE=1 SV=2	APEX1_HUMAN	35,555.70	100.00%	7	10	19	0.01450%	28.30%
Pap test	DNA-dependent protein kinase catalytic subunit GN=PRKDC PE=1 SV=3	sp|P78527|PRKDC_HUMAN	469,095.50	99.50%	1	1	1	0.00156%	0.27%
Tumor tissue	DNA-dependent protein kinase catalytic subunit GN=PRKDC PE=1 SV=3	sp|P78527|PRKDC_HUMAN	469,095.50	100.00%	65	85	108	0.08270%	21.30%

Biological sample	Protein name	Protein accession numbers	Protein molecular weight (Da)	Protein identification probability	Exclusive unique peptide count	Exclusive unique spectrum count	Total spectrum count	Percentage of total spectra	Percentage sequence coverage
Tumor tissue	DNA-directed RNA polymerase II subunit RPB1 GN=POLR2A PE=1 SV=2	sp|P24928|RPB1_HUMAN	217,181.10	100.00%	3	3	3	0.00230%	2.54%
Tumor tissue	DNA-directed RNA polymerase II subunit RPB3 GN=POLR2C PE=1 SV=2	RPB3_HUMAN	31,441.70	100.00%	4	4	7	0.00536%	20.70%
Tumor tissue	DNA-directed RNA polymerase II subunit RPB7 GN=POLR2G PE=1 SV=1	RPB7_HUMAN	19,294.60	100.00%	2	2	2	0.00153%	11.00%
Tumor tissue	DNA-directed RNA polymerase subunit beta GN=POLR2B PE=1 SV=2	RPB2_HUMAN	133,061.10	100.00%	9	11	12	0.00919%	11.80%
Tumor tissue	DNA-directed RNA polymerases I and III subunit RPAC1 GN=POLR1C PE=1 SV=1	sp|O15160|RPAC1_HUMAN	33,839.60	100.00%	2	2	2	0.00153%	10.50%
Tumor tissue	DNA-directed RNA polymerases I, II, and III subunit RPABC1 GN=POLR2E PE=1 SV=1	RPAB1_HUMAN	23,566.00	100.00%	2	2	2	0.00153%	12.50%
Tumor tissue	DnaJ (Hsp40) homolog, subfamily B, member 12, isoform CRA_c GN=DNAJB12 PE=1 SV=1	sp|Q9NXW2|DJB12_HUMAN	45,490.60	100.00%	2	2	2	0.00153%	10.30%
Tumor tissue	DnaJ homolog subfamily A member 1 GN=DNAJA1 PE=1 SV=2	sp|P31689|DNJA1_HUMAN	44,868.60	100.00%	5	7	8	0.00612%	20.70%
Tumor tissue	DnaJ homolog subfamily A member 2 GN=DNAJA2 PE=1 SV=1	DNJA2_HUMAN	45,745.70	100.00%	3	3	5	0.00383%	9.71%
Pap test	DnaJ homolog subfamily B member 1 GN=DNAJB1 PE=1 SV=4	sp|P25685|DNJB1_HUMAN	38,045.60	100.00%	2	2	3	0.00468%	6.76%
Swab	DnaJ homolog subfamily B member 1 GN=DNAJB1 PE=1 SV=4	sp|P25685|DNJB1_HUMAN	38,045.60	100.00%	1	1	1	0.00071%	4.41%
Tumor tissue	DnaJ homolog subfamily B member 1 GN=DNAJB1 PE=1 SV=4	sp|P25685|DNJB1_HUMAN	38,045.60	100.00%	3	3	4	0.00306%	9.41%
Tumor tissue	DnaJ homolog subfamily B member 11 GN=DNAJB11 PE=1 SV=1	DJB11_HUMAN	40,514.60	100.00%	6	7	7	0.00536%	23.50%
Tumor tissue	DnaJ homolog subfamily C member 1 GN=DNAJC1 PE=1 SV=1	DNJC1_HUMAN	63,884.90	100.00%	3	3	3	0.00230%	6.86%

Biological sample	Protein name	Protein accession numbers	Protein molecular weight (Da)	Protein identification probability	Exclusive unique peptide count	Exclusive unique spectrum count	Total spectrum count	Percentage of total spectra	Percentage sequence coverage
Tumor tissue	DnaJ homolog subfamily C member 10 GN=DNAJC10 PE=1 SV=2	sp|Q8IXB1|DJC10_HUMAN	91,082.40	100.00%	4	4	4	0.00306%	5.55%
Tumor tissue	DnaJ homolog subfamily C member 13 GN=DNAJC13 PE=1 SV=5	DJC13_HUMAN	254,422.90	100.00%	17	19	21	0.01610%	10.70%
Tumor tissue	DnaJ homolog subfamily C member 2 GN=DNAJC2 PE=1 SV=4	sp|Q99543|DNJC2_HUMAN	71,997.90	100.00%	2	2	2	0.00153%	4.03%
Tumor tissue	DnaJ homolog subfamily C member 3 GN=DNAJC3 PE=1 SV=1	DNJC3_HUMAN	57,582.20	100.00%	3	3	4	0.00306%	7.54%
Tumor tissue	DnaJ homolog subfamily C member 8 GN=DNAJC8 PE=1 SV=2	DNJC8_HUMAN	29,842.20	100.00%	2	2	2	0.00153%	13.40%
Tumor tissue	DnaJ homolog subfamily C member 9 GN=DNAJC9 PE=1 SV=1	DNJC9_HUMAN	29,910.30	100.00%	4	4	4	0.00306%	16.90%
Tumor tissue	Docking protein 2 GN=DOK2 PE=1 SV=2	DOK2_HUMAN	45,379.60	100.00%	2	2	2	0.00153%	6.31%
Tumor tissue	Dolichol-phosphate mannosyltransferase subunit 1 GN=DPM1 PE=1 SV=1	DPM1_HUMAN	32,428.20	100.00%	6	6	7	0.00536%	30.80%
Pap test	Dolichyl-diphosphooligosaccharide--protein glycosyltransferase subunit 1 GN=RPN1 PE=1 SV=1	RPN1_HUMAN	68,571.00	100.00%	5	5	6	0.00936%	10.70%
Tumor tissue	Dolichyl-diphosphooligosaccharide--protein glycosyltransferase subunit 1 GN=RPN1 PE=1 SV=1	RPN1_HUMAN	68,571.00	100.00%	19	29	57	0.04360%	43.50%
Tumor tissue	Dolichyl-diphosphooligosaccharide--protein glycosyltransferase subunit DAD1 GN=DAD1 PE=1 SV=3	DAD1_HUMAN	9,554.70	100.00%	2	2	3	0.00230%	19.50%
Tumor tissue	Dolichyl-diphosphooligosaccharide--protein glycosyltransferase subunit STT3A GN=STT3A PE=1 SV=2	sp|P46977|STT3A_HUMAN	80,532.80	100.00%	6	7	12	0.00919%	8.37%

Biological sample	Protein name	Protein accession numbers	Protein molecular weight (Da)	Protein identification probability	Exclusive unique peptide count	Exclusive unique spectrum count	Total spectrum count	Percentage of total spectra	Percentage sequence coverage
Tumor tissue	Dolichyl-diphosphooligosaccharide--protein glycosyltransferase subunit STT3B GN=STT3B PE=1 SV=1	STT3B_HUMAN	93,677.70	100.00%	4	4	5	0.00383%	5.57%
Tumor tissue	Dolichyl-phosphate beta-glucosyltransferase GN=ALG5 PE=1 SV=1	sp|Q9Y673|ALG5_HUMAN	36,947.50	100.00%	3	3	4	0.00306%	14.80%
Tumor tissue	Double-strand break repair protein MRE11A GN=MRE11 PE=1 SV=1	sp|P49959|MRE11_HUMAN	80,524.00	100.00%	5	5	5	0.00383%	8.63%
Tumor tissue	Double-strand-break repair protein rad21 homolog GN=RAD21 PE=1 SV=2	RAD21_HUMAN	71,691.60	100.00%	5	5	6	0.00459%	9.19%
Tumor tissue	Double-stranded RNA-binding protein Staufen homolog 1 GN=STAU1 PE=1 SV=1	sp|O95793|STAU1_HUMAN	54,709.50	100.00%	2	3	17	0.01300%	41.10%
Tumor tissue	Double-stranded RNA-binding protein Staufen homolog 2 GN=STAU2 PE=1 SV=1	sp|Q9NUL3|STAU2_HUMAN	58,971.60	100.00%	1	1	2	0.00153%	4.91%
Pap test	Drebrin-like protein GN=DBNL PE=1 SV=1	sp|Q9UJU6|DBNL_HUMAN	48,206.30	100.00%	3	4	4	0.00624%	10.70%
Swab	Drebrin-like protein GN=DBNL PE=1 SV=1	sp|Q9UJU6|DBNL_HUMAN	48,206.30	100.00%	7	9	14	0.00992%	23.50%
Tumor tissue	Drebrin-like protein GN=DBNL PE=1 SV=1	sp|Q9UJU6|DBNL_HUMAN	48,206.30	100.00%	12	20	23	0.01760%	34.40%
Tumor tissue	D-tyrosyl-tRNA(Tyr) deacylase 1 GN=DTD1 PE=1 SV=2	DTD1_HUMAN	23,423.60	99.70%	2	2	2	0.00153%	12.90%
Pap test	Dual oxidase 2 GN=DUOX2 PE=1 SV=2	DUOX2_HUMAN	175,341.70	100.00%	2	2	8	0.01250%	4.46%
Tumor tissue	Dual specificity mitogen-activated protein kinase kinase 2 GN=MAP2K2 PE=1 SV=1	MP2K2_HUMAN	44,425.30	100.00%	2	2	6	0.00459%	13.80%
Swab	Dual specificity protein phosphatase 23 GN=DUSP23 PE=1 SV=1	DUS23_HUMAN	16,588.40	99.60%	1	1	1	0.00071%	8.00%

Biological sample	Protein name	Protein accession numbers	Protein molecular weight (Da)	Protein identification probability	Exclusive unique peptide count	Exclusive unique spectrum count	Total spectrum count	Percentage of total spectra	Percentage sequence coverage
Tumor tissue	Dual specificity protein phosphatase 23 GN=DUSP23 PE=1 SV=1	DUS23_HUMAN	16,588.40	100.00%	4	5	5	0.00383%	33.30%
Swab	Dual specificity protein phosphatase 3 GN=DUSP3 PE=1 SV=1	sp|P51452|DUS3_HUMAN	20,478.90	99.60%	1	1	1	0.00071%	7.03%
Tumor tissue	Dual specificity protein phosphatase 3 GN=DUSP3 PE=1 SV=1	sp|P51452|DUS3_HUMAN	20,478.90	100.00%	3	4	7	0.00536%	27.00%
Pap test	Dynactin subunit 1 GN=DCTN1 PE=1 SV=1	sp|Q14203|DCTN1_HUMAN	139,096.90	100.00%	2	2	2	0.00312%	1.91%
Swab	Dynactin subunit 1 GN=DCTN1 PE=1 SV=1	sp|Q14203|DCTN1_HUMAN	139,096.90	100.00%	4	4	4	0.00283%	4.94%
Tumor tissue	Dynactin subunit 1 GN=DCTN1 PE=1 SV=1	sp|Q14203|DCTN1_HUMAN	139,096.90	100.00%	19	24	29	0.02220%	23.60%
Tumor tissue	Dynactin subunit 3 GN=DCTN3 PE=1 SV=1	X6RA56_HUMAN	16,091.10	100.00%	3	3	3	0.00230%	20.40%
Tumor tissue	Dynamin-1-like protein (Fragment) GN=DNM1L PE=1 SV=8	F8W1W3_HUMAN	18,330.40	99.50%	1	1	4	0.00306%	26.20%
Tumor tissue	Dynein assembly factor 5, axonemal GN=DNAAF5 PE=1 SV=4	sp|Q86Y56|DAAF5_HUMAN	93,522.40	100.00%	2	2	2	0.00153%	3.27%
Tumor tissue	Dynein light chain 1, cytoplasmic GN=DYNLL1 PE=1 SV=1	DYL1_HUMAN	10,366.10	100.00%	1	1	2	0.00153%	25.80%
Swab	Dynein light chain roadblock-type 1 GN=DYNLRB1 PE=1 SV=1	DLRB1_HUMAN	16,253.00	99.60%	1	1	2	0.00142%	8.11%
Tumor tissue	Dynein light chain roadblock-type 1 GN=DYNLRB1 PE=1 SV=1	DLRB1_HUMAN	16,253.00	100.00%	2	2	4	0.00306%	22.30%
Tumor tissue	Dystroglycan GN=DAG1 PE=1 SV=2	DAG1_HUMAN	97,442.20	100.00%	4	5	5	0.00383%	5.36%
Tumor tissue	E2 ubiquitin-conjugating enzyme GN=UBE2O PE=1 SV=3	sp|Q9C0C9|UBE2O_HUMAN	141,293.50	100.00%	5	5	5	0.00383%	5.96%
Tumor tissue	E3 SUMO-protein ligase RanBP2 GN=RANBP2 PE=1 SV=2	RBP2_HUMAN	358,198.20	100.00%	14	15	23	0.01760%	8.65%
Tumor tissue	E3 ubiquitin/ISG15 ligase TRIM25 GN=TRIM25 PE=1 SV=2	TRI25_HUMAN	70,973.20	100.00%	12	14	15	0.01150%	23.80%

Biological sample	Protein name	Protein accession numbers	Protein molecular weight (Da)	Protein identification probability	Exclusive unique peptide count	Exclusive unique spectrum count	Total spectrum count	Percentage of total spectra	Percentage sequence coverage
Tumor tissue	E3 ubiquitin-protein ligase ARIH1 GN=ARIH1 PE=1 SV=2	ARI1_HUMAN	64,117.20	100.00%	2	2	2	0.00153%	4.67%
Tumor tissue	E3 ubiquitin-protein ligase BRE1A GN=RNF20 PE=1 SV=2	BRE1A_HUMAN	113,666.00	100.00%	2	3	5	0.00383%	8.21%
Tumor tissue	E3 ubiquitin-protein ligase BRE1B GN=RNF40 PE=1 SV=2	sp|O75150|BRE1B_HUMAN	109,051.60	100.00%	2	2	3	0.00230%	5.20%
Tumor tissue	E3 ubiquitin-protein ligase CHIP GN=STUB1 PE=1 SV=2	sp|Q9UNE7|CHIP_HUMAN	34,857.00	100.00%	1	1	2	0.00153%	8.91%
Tumor tissue	E3 ubiquitin-protein ligase DTX3L GN=DTX3L PE=1 SV=1	sp|Q8TDB6|DTX3L_HUMAN	83,556.20	100.00%	10	12	13	0.00995%	20.70%
Tumor tissue	E3 ubiquitin-protein ligase HECTD1 GN=HECTD1 PE=1 SV=1	A0A087X2H1_HUMAN	289,635.80	100.00%	2	2	2	0.00153%	1.15%
Tumor tissue	E3 ubiquitin-protein ligase RNF170 GN=RNF170 PE=1 SV=2	sp|Q96K19|RN170_HUMAN	29,815.40	100.00%	2	2	2	0.00153%	8.91%
Tumor tissue	E3 ubiquitin-protein ligase RNF213 GN=RNF213 PE=1 SV=1	sp|Q63HN8|RN213_HUMAN	596,486.10	100.00%	29	32	39	0.02990%	8.73%
Tumor tissue	E3 ubiquitin-protein ligase TRIM21 GN=TRIM21 PE=1 SV=1	sp|P19474|RO52_HUMAN	54,169.50	100.00%	2	3	3	0.00230%	5.47%
Tumor tissue	E3 ubiquitin-protein ligase TRIM56 GN=TRIM56 PE=1 SV=3	sp|Q9BRZ2|TRI56_HUMAN	81,487.20	100.00%	3	3	4	0.00306%	5.96%
Tumor tissue	E3 ubiquitin-protein ligase TRIP12 GN=TRIP12 PE=1 SV=1	sp|Q14669|TRIPC_HUMAN	220,438.10	100.00%	8	8	10	0.00766%	5.82%
Tumor tissue	E3 ubiquitin-protein ligase UBR5 GN=UBR5 PE=1 SV=1	sp|O95071|UBR5_HUMAN	308,576.60	100.00%	4	4	4	0.00306%	3.44%
Tumor tissue	E3 UFM1-protein ligase 1 GN=UFL1 PE=1 SV=2	sp|O94874|UFL1_HUMAN	89,598.00	100.00%	14	16	17	0.01300%	24.30%
Pap test	Early endosome antigen 1 GN=EEA1 PE=1 SV=2	EEA1_HUMAN	162,468.10	100.00%	3	3	3	0.00468%	2.41%
Tumor tissue	Early endosome antigen 1 GN=EEA1 PE=1 SV=2	EEA1_HUMAN	162,468.10	100.00%	19	20	20	0.01530%	19.70%
Tumor tissue	Echinoderm microtubule associated protein like 3, isoform CRA_e GN=EML3 PE=1 SV=1	sp|Q32P44|EMAL3_HUMAN	95,284.20	100.00%	2	2	5	0.00383%	7.58%
Tumor tissue	Echinoderm microtubule-associated protein-like 1 GN=EML1 PE=1 SV=1	sp|O00423|EMAL1_HUMAN	88,716.90	100.00%	1	1	3	0.00230%	6.35%

Biological sample	Protein name	Protein accession numbers	Protein molecular weight (Da)	Protein identification probability	Exclusive unique peptide count	Exclusive unique spectrum count	Total spectrum count	Percentage of total spectra	Percentage sequence coverage
Tumor tissue	Echinoderm microtubule-associated protein-like 4 GN=EML4 PE=1 SV=3	sp|Q9HC35|EMAL4_HUMAN	108,917.50	100.00%	1	1	16	0.01220%	16.80%
Tumor tissue	Ectonucleotide pyrophosphatase/phosphodiesterase family member 1 GN=ENPP1 PE=1 SV=2	ENPP1_HUMAN	104,927.20	100.00%	6	8	9	0.00689%	12.00%
Tumor tissue	EF-hand domain-containing protein D1 GN=EFHD1 PE=1 SV=1	sp|Q9BUP0|EFHD1_HUMAN	26,929.20	100.00%	4	4	7	0.00536%	40.20%
Pap test	EF-hand domain-containing protein D2 GN=EFHD2 PE=1 SV=1	EFHD2_HUMAN	26,698.40	100.00%	1	1	7	0.01090%	27.10%
Swab	EF-hand domain-containing protein D2 GN=EFHD2 PE=1 SV=1	EFHD2_HUMAN	26,698.40	100.00%	2	3	12	0.00850%	23.30%
Tumor tissue	EF-hand domain-containing protein D2 GN=EFHD2 PE=1 SV=1	EFHD2_HUMAN	26,698.40	100.00%	4	5	11	0.00842%	30.40%
Tumor tissue	EH domain-binding protein 1-like protein 1 GN=EHBP1L1 PE=1 SV=2	EH1L1_HUMAN	161,851.80	100.00%	2	2	3	0.00230%	2.63%
Pap test	EH domain-containing protein 1 GN=EHD1 PE=1 SV=2	EHD1_HUMAN	60,629.10	100.00%	2	2	2	0.00312%	6.37%
Swab	EH domain-containing protein 1 GN=EHD1 PE=1 SV=2	EHD1_HUMAN	60,629.10	100.00%	2	3	6	0.00425%	7.68%
Tumor tissue	EH domain-containing protein 1 GN=EHD1 PE=1 SV=2	EHD1_HUMAN	60,629.10	100.00%	13	14	19	0.01450%	33.50%
Tumor tissue	EH domain-containing protein 2 GN=EHD2 PE=1 SV=2	sp|Q9NZN4|EHD2_HUMAN	61,163.20	100.00%	18	26	42	0.03220%	46.60%
Tumor tissue	EH domain-containing protein 4 GN=EHD4 PE=1 SV=1	EHD4_HUMAN	61,177.60	100.00%	14	14	23	0.01760%	38.10%
Tumor tissue	eIF-2-alpha kinase activator GCN1 GN=GCN1 PE=1 SV=6	GCN1_HUMAN	292,764.40	100.00%	35	47	61	0.04670%	18.20%
Swab	Electron transfer flavoprotein subunit alpha, mitochondrial GN=ETFA PE=1 SV=1	sp|P13804|ETFA_HUMAN	35,080.10	99.90%	1	1	3	0.00213%	7.81%
Tumor tissue	Electron transfer flavoprotein-ubiquinone oxidoreductase, mitochondrial GN=ETFDH PE=1 SV=2	sp|Q16134|ETFD_HUMAN	68,496.60	100.00%	2	2	2	0.00153%	5.35%

Biological sample	Protein name	Protein accession numbers	Protein molecular weight (Da)	Protein identification probability	Exclusive unique peptide count	Exclusive unique spectrum count	Total spectrum count	Percentage of total spectra	Percentage sequence coverage
Tumor tissue	ELKS/Rab6-interacting/CAST family member 1 GN=ERC1 PE=1 SV=1	sp|Q8IUD2|RB6I2_HUMAN	124,824.50	100.00%	1	1	5	0.00383%	6.35%
Tumor tissue	ELMO domain-containing protein 2 (Fragment) GN=ELMOD2 PE=1 SV=8	ELMD2_HUMAN	20,148.30	100.00%	3	4	4	0.00306%	22.20%
Swab	Elongation factor 1-beta GN=EEF1B2 PE=1 SV=3	EF1B_HUMAN	24,764.20	100.00%	3	3	4	0.00283%	16.40%
Tumor tissue	Elongation factor 1-beta GN=EEF1B2 PE=1 SV=3	EF1B_HUMAN	24,764.20	100.00%	4	7	13	0.00995%	24.40%
Pap test	Elongation factor 2 GN=EEF2 PE=1 SV=4	EF2_HUMAN	95,340.10	100.00%	12	13	20	0.03120%	16.70%
Swab	Elongation factor 2 GN=EEF2 PE=1 SV=4	EF2_HUMAN	95,340.10	100.00%	13	15	31	0.02200%	19.00%
Tumor tissue	Elongation factor 2 GN=EEF2 PE=1 SV=4	EF2_HUMAN	95,340.10	100.00%	27	44	78	0.05970%	30.70%
Tumor tissue	Elongation factor G, mitochondrial GN=GFM1 PE=1 SV=2	sp|Q96RP9|EFGM_HUMAN	83,473.50	100.00%	2	2	9	0.00689%	16.10%
Tumor tissue	Elongation factor Ts, mitochondrial GN=TSFM PE=1 SV=2	sp|P43897|EFTS_HUMAN	35,391.00	100.00%	2	2	3	0.00230%	20.30%
Pap test	Elongation factor Tu, mitochondrial GN=TUFM PE=1 SV=2	EFTU_HUMAN	49,542.40	100.00%	2	2	2	0.00312%	7.30%
Swab	Elongation factor Tu, mitochondrial GN=TUFM PE=1 SV=2	EFTU_HUMAN	49,542.40	100.00%	2	2	2	0.00142%	8.19%
Tumor tissue	Elongation factor Tu, mitochondrial GN=TUFM PE=1 SV=2	EFTU_HUMAN	49,542.40	100.00%	12	20	29	0.02220%	35.20%
Tumor tissue	Elongator complex protein 1 GN=IKBKAP PE=1 SV=3	ELP1_HUMAN	150,255.40	100.00%	2	2	2	0.00153%	3.08%
Tumor tissue	Embryonic stem cell-specific 5-hydroxymethylcytosine-binding protein GN=HMCES PE=1 SV=1	HMCES_HUMAN	40,575.20	100.00%	2	2	3	0.00230%	13.60%
Tumor tissue	Emerin GN=EMD PE=1 SV=1	EMD_HUMAN	28,994.90	100.00%	6	7	9	0.00689%	27.20%
Tumor tissue	EMILIN-1 GN=EMILIN1 PE=1 SV=1	A0A0C4DFX3_HUMAN	106,694.70	100.00%	18	31	44	0.03370%	24.00%
Tumor tissue	EMILIN-2 GN=EMILIN2 PE=1 SV=3	EMIL2_HUMAN	115,686.90	100.00%	4	4	4	0.00306%	4.46%

Biological sample	Protein name	Protein accession numbers	Protein molecular weight (Da)	Protein identification probability	Exclusive unique peptide count	Exclusive unique spectrum count	Total spectrum count	Percentage of total spectra	Percentage sequence coverage
Pap test	Endonuclease domain-containing 1 protein GN=ENDOD1 PE=1 SV=2	ENDD1_HUMAN	55,017.60	99.20%	1	1	1	0.00156%	2.80%
Tumor tissue	Endonuclease domain-containing 1 protein GN=ENDOD1 PE=1 SV=2	ENDD1_HUMAN	55,017.60	100.00%	3	3	3	0.00230%	7.00%
Pap test	Endophilin-A2 GN=SH3GL1 PE=1 SV=1	sp|Q99961|SH3G1_HUMAN	41,491.20	99.80%	1	1	1	0.00156%	3.26%
Tumor tissue	Endophilin-A2 GN=SH3GL1 PE=1 SV=1	sp|Q99961|SH3G1_HUMAN	41,491.20	100.00%	3	3	4	0.00306%	14.40%
Tumor tissue	Endophilin-B1 GN=SH3GLB1 PE=1 SV=1	sp|Q9Y371|SHLB1_HUMAN	44,262.20	100.00%	7	9	10	0.00766%	20.80%
Tumor tissue	Endophilin-B2 GN=SH3GLB2 PE=1 SV=1	sp|Q9NR46|SHLB2_HUMAN	41,669.60	100.00%	5	5	6	0.00459%	16.00%
Swab	Endoplasmic reticulum aminopeptidase 1 GN=ERAP1 PE=1 SV=3	sp|Q9NZ08|ERAP1_HUMAN	107,237.90	100.00%	1	1	1	0.00071%	1.38%
Tumor tissue	Endoplasmic reticulum aminopeptidase 1 GN=ERAP1 PE=1 SV=3	sp|Q9NZ08|ERAP1_HUMAN	107,237.90	100.00%	17	21	23	0.01760%	23.10%
Pap test	Endoplasmic reticulum metallopeptidase 1 GN=ERMP1 PE=1 SV=1	sp|Q7Z2K6|ERMP1_HUMAN	93,162.30	100.00%	6	6	8	0.01250%	8.92%
Tumor tissue	Endoplasmic reticulum metallopeptidase 1 GN=ERMP1 PE=1 SV=1	sp|Q7Z2K6|ERMP1_HUMAN	93,162.30	100.00%	4	4	4	0.00306%	6.54%
Swab	Endoplasmic reticulum resident protein 29 GN=ERP29 PE=1 SV=4	sp|P30040|ERP29_HUMAN	28,994.90	100.00%	2	2	4	0.00283%	8.43%
Tumor tissue	Endoplasmic reticulum resident protein 29 GN=ERP29 PE=1 SV=4	sp|P30040|ERP29_HUMAN	28,994.90	100.00%	7	9	22	0.01680%	28.70%
Pap test	Endoplasmic reticulum resident protein 44 GN=ERP44 PE=1 SV=1	ERP44_HUMAN	46,972.50	100.00%	4	4	4	0.00624%	11.80%
Swab	Endoplasmic reticulum resident protein 44 GN=ERP44 PE=1 SV=1	ERP44_HUMAN	46,972.50	100.00%	4	5	8	0.00567%	15.30%
Tumor tissue	Endoplasmic reticulum resident protein 44 GN=ERP44 PE=1 SV=1	ERP44_HUMAN	46,972.50	100.00%	5	9	11	0.00842%	19.70%

Biological sample	Protein name	Protein accession numbers	Protein molecular weight (Da)	Protein identification probability	Exclusive unique peptide count	Exclusive unique spectrum count	Total spectrum count	Percentage of total spectra	Percentage sequence coverage
Tumor tissue	Endoplasmic reticulum-Golgi intermediate compartment protein 2 GN=ERGIC2 PE=1 SV=1	ERGI2_HUMAN	43,432.60	100.00%	2	2	2	0.00153%	9.07%
Tumor tissue	Endoplasmic reticulum-Golgi intermediate compartment protein 3 (Fragment) GN=ERGIC3 PE=1 SV=1	sp|Q9Y282|ERGI3_HUMAN	44,679.10	100.00%	3	3	3	0.00230%	8.56%
Pap test	Endoplasmin GN=HSP90B1 PE=1 SV=1	ENPL_HUMAN	92,471.70	100.00%	6	6	8	0.01250%	11.80%
Swab	Endoplasmin GN=HSP90B1 PE=1 SV=1	ENPL_HUMAN	92,471.70	100.00%	6	7	13	0.00921%	12.50%
Tumor tissue	Endoplasmin GN=HSP90B1 PE=1 SV=1	ENPL_HUMAN	92,471.70	100.00%	27	46	98	0.07500%	43.30%
Tumor tissue	Endoribonuclease LACTB2 GN=LACTB2 PE=1 SV=2	LACB2_HUMAN	32,806.70	100.00%	5	7	7	0.00536%	24.00%
Tumor tissue	Endothelial protein C receptor (Fragment) GN=PROCR PE=1 SV=1	EPCR_HUMAN	24,395.20	100.00%	2	2	2	0.00153%	12.20%
Tumor tissue	Engulfment and cell motility protein 1 GN=ELMO1 PE=1 SV=2	sp|Q92556|ELMO1_HUMAN	83,833.20	100.00%	5	5	6	0.00459%	10.30%
Tumor tissue	Enhancer of mRNA-decapping protein 3 GN=EDC3 PE=1 SV=1	EDC3_HUMAN	56,077.40	100.00%	2	2	2	0.00153%	6.30%
Tumor tissue	Enhancer of mRNA-decapping protein 4 GN=EDC4 PE=1 SV=1	sp|Q6P2E9|EDC4_HUMAN	151,662.20	100.00%	12	12	21	0.01610%	19.20%
Swab	Enhancer of rudimentary homolog GN=ERH PE=1 SV=1	ERH_HUMAN	12,259.30	98.00%	1	1	1	0.00071%	16.30%
Tumor tissue	Enhancer of rudimentary homolog GN=ERH PE=1 SV=1	ERH_HUMAN	12,259.30	100.00%	2	2	2	0.00153%	18.30%
Swab	Enolase-phosphatase E1 GN=ENOPH1 PE=1 SV=1	sp|Q9UHY7|ENOPH_HUMAN	28,932.40	100.00%	1	1	1	0.00071%	7.66%
Pap test	Enoyl-CoA hydratase, mitochondrial GN=ECHS1 PE=1 SV=4	ECHM_HUMAN	31,388.20	100.00%	2	2	2	0.00312%	13.40%
Swab	Enoyl-CoA hydratase, mitochondrial GN=ECHS1 PE=1 SV=4	ECHM_HUMAN	31,388.20	100.00%	2	2	3	0.00213%	13.40%

Biological sample	Protein name	Protein accession numbers	Protein molecular weight (Da)	Protein identification probability	Exclusive unique peptide count	Exclusive unique spectrum count	Total spectrum count	Percentage of total spectra	Percentage sequence coverage
Tumor tissue	Enoyl-CoA hydratase, mitochondrial GN=ECHS1 PE=1 SV=4	ECHM_HUMAN	31,388.20	100.00%	7	10	11	0.00842%	31.40%
Pap test	Envoplakin GN=EVPL PE=1 SV=3	EVPL_HUMAN	233,830.90	100.00%	30	33	38	0.05930%	20.60%
Tumor tissue	Envoplakin GN=EVPL PE=1 SV=3	EVPL_HUMAN	233,830.90	100.00%	6	6	6	0.00459%	4.62%
Pap test	Eosinophil cationic protein GN=RNASE3 PE=1 SV=2	ECP_HUMAN	18,385.30	100.00%	2	2	9	0.01400%	13.10%
Swab	Eosinophil cationic protein GN=RNASE3 PE=1 SV=2	ECP_HUMAN	18,385.30	100.00%	2	2	3	0.00213%	13.10%
Tumor tissue	Eosinophil cationic protein GN=RNASE3 PE=1 SV=2	ECP_HUMAN	18,385.30	100.00%	2	4	7	0.00536%	16.90%
Tumor tissue	Eosinophil peroxidase GN=EPX PE=1 SV=2	PERE_HUMAN	81,043.90	100.00%	9	12	17	0.01300%	13.30%
Pap test	Epidermal growth factor receptor kinase substrate 8-like protein 1 GN=EPS8L1 PE=1 SV=3	sp|Q8TE68|ES8L1_HUMAN	80,250.80	100.00%	1	1	10	0.01560%	16.00%
Pap test	Epidermal growth factor receptor kinase substrate 8-like protein 2 GN=EPS8L2 PE=1 SV=2	sp|Q9H6S3|ES8L2_HUMAN	80,621.10	100.00%	1	1	2	0.00312%	2.80%
Swab	Epidermal growth factor receptor kinase substrate 8-like protein 2 GN=EPS8L2 PE=1 SV=2	sp|Q9H6S3|ES8L2_HUMAN	80,621.10	100.00%	1	1	1	0.00071%	2.80%
Tumor tissue	Epidermal growth factor receptor kinase substrate 8-like protein 2 GN=EPS8L2 PE=1 SV=2	sp|Q9H6S3|ES8L2_HUMAN	80,621.10	100.00%	2	2	3	0.00230%	5.45%
Swab	Epidermal growth factor receptor substrate 15 GN=EPS15 PE=1 SV=2	sp|P42566|EPS15_HUMAN	98,658.00	99.80%	1	1	1	0.00071%	2.12%
Tumor tissue	Epidermal growth factor receptor substrate 15 GN=EPS15 PE=1 SV=2	sp|P42566|EPS15_HUMAN	98,658.00	100.00%	8	10	10	0.00766%	12.40%
Pap test	Epididymal secretory protein E1 GN=NPC2 PE=1 SV=1	sp|P61916|NPC2_HUMAN	19,229.80	100.00%	3	4	7	0.01090%	24.40%
Swab	Epididymal secretory protein E1 GN=NPC2 PE=1 SV=1	sp|P61916|NPC2_HUMAN	19,229.80	100.00%	2	2	6	0.00425%	12.90%
Swab	Epiplakin GN=EPPK1 PE=1 SV=2	A0A075B730_HUMAN	555,652.20	100.00%	1	1	1	0.00071%	0.55%

Biological sample	Protein name	Protein accession numbers	Protein molecular weight (Da)	Protein identification probability	Exclusive unique peptide count	Exclusive unique spectrum count	Total spectrum count	Percentage of total spectra	Percentage sequence coverage
Tumor tissue	Epiplakin GN=EPPK1 PE=1 SV=2	A0A075B730_HUMAN	555,652.20	100.00%	1	1	87	0.06660%	13.70%
Tumor tissue	Epiplakin GN=EPPK1 PE=1 SV=2	EPIPL_HUMAN	555,615.40	100.00%	2	4	91	0.06970%	14.00%
Tumor tissue	EPM2A-interacting protein 1 GN=EPM2AIP1 PE=1 SV=1	EPMIP_HUMAN	70,370.80	100.00%	6	7	7	0.00536%	16.10%
Pap test	Epoxide hydrolase 1 GN=EPHX1 PE=1 SV=1	HYEP_HUMAN	52,951.10	100.00%	2	2	2	0.00312%	5.93%
Tumor tissue	Epoxide hydrolase 1 GN=EPHX1 PE=1 SV=1	HYEP_HUMAN	52,951.10	100.00%	8	13	14	0.01070%	19.80%
Tumor tissue	ER membrane protein complex subunit 2 GN=EMC2 PE=1 SV=1	EMC2_HUMAN	34,834.80	100.00%	3	3	4	0.00306%	19.20%
Pap test	ERO1-like protein alpha GN=ERO1A PE=1 SV=2	ERO1A_HUMAN	54,394.70	100.00%	12	18	36	0.05620%	36.80%
Swab	ERO1-like protein alpha GN=ERO1A PE=1 SV=2	ERO1A_HUMAN	54,394.70	100.00%	6	8	14	0.00992%	24.40%
Tumor tissue	ERO1-like protein alpha GN=ERO1A PE=1 SV=2	ERO1A_HUMAN	54,394.70	100.00%	6	8	13	0.00995%	20.30%
Pap test	Erythrocyte band 7 integral membrane protein GN=STOM PE=1 SV=3	sp|P27105|STOM_HUMAN	31,732.10	100.00%	3	3	6	0.00936%	11.80%
Swab	Erythrocyte band 7 integral membrane protein GN=STOM PE=1 SV=3	sp|P27105|STOM_HUMAN	31,732.10	100.00%	3	3	3	0.00213%	13.50%
Tumor tissue	Erythrocyte band 7 integral membrane protein GN=STOM PE=1 SV=3	sp|P27105|STOM_HUMAN	31,732.10	100.00%	7	9	9	0.00689%	31.60%
Pap test	Ester hydrolase C11orf54 GN=C11orf54 PE=1 SV=1	sp|Q9H0W9|CK054_HUMAN	28,683.50	100.00%	4	5	7	0.01090%	18.80%
Swab	Ester hydrolase C11orf54 GN=C11orf54 PE=1 SV=1	sp|Q9H0W9|CK054_HUMAN	28,683.50	99.30%	1	1	2	0.00142%	4.30%
Tumor tissue	Ester hydrolase C11orf54 GN=C11orf54 PE=1 SV=1	sp|Q9H0W9|CK054_HUMAN	28,683.50	100.00%	2	3	5	0.00383%	10.20%
Tumor tissue	Estradiol 17-beta-dehydrogenase 11 GN=HSD17B11 PE=1 SV=2	DHB11_HUMAN	28,103.50	100.00%	2	3	3	0.00230%	12.90%

Biological sample	Protein name	Protein accession numbers	Protein molecular weight (Da)	Protein identification probability	Exclusive unique peptide count	Exclusive unique spectrum count	Total spectrum count	Percentage of total spectra	Percentage sequence coverage
Pap test	Eukaryotic initiation factor 4A-I GN=EIF4A1 PE=1 SV=1	sp|P60842|IF4A1_HUMAN	46,155.30	100.00%	2	2	5	0.00780%	12.10%
Swab	Eukaryotic initiation factor 4A-I GN=EIF4A1 PE=1 SV=1	sp|P60842|IF4A1_HUMAN	46,155.30	100.00%	3	5	17	0.01200%	15.30%
Tumor tissue	Eukaryotic initiation factor 4A-I GN=EIF4A1 PE=1 SV=1	sp|P60842|IF4A1_HUMAN	46,155.30	100.00%	9	16	37	0.02830%	39.90%
Swab	Eukaryotic initiation factor 4A-III GN=EIF4A3 PE=1 SV=4	IF4A3_HUMAN	46,872.40	99.80%	1	1	1	0.00071%	2.43%
Tumor tissue	Eukaryotic initiation factor 4A-III GN=EIF4A3 PE=1 SV=4	IF4A3_HUMAN	46,872.40	100.00%	8	11	18	0.01380%	25.30%
Tumor tissue	Eukaryotic peptide chain release factor subunit 1 GN=ETF1 PE=1 SV=3	sp|P62495|ERF1_HUMAN	49,032.60	100.00%	1	1	13	0.00995%	25.60%
Tumor tissue	Eukaryotic translation initiation factor 1A, X-chromosomal GN=EIF1AX PE=1 SV=2	IF1AX_HUMAN	16,461.30	100.00%	3	3	3	0.00230%	29.20%
Tumor tissue	Eukaryotic translation initiation factor 1b GN=EIF1B PE=1 SV=2	EIF1B_HUMAN	13,608.10	100.00%	2	2	3	0.00230%	26.50%
Tumor tissue	Eukaryotic translation initiation factor 2 subunit 1 GN=EIF2S1 PE=1 SV=3	IF2A_HUMAN	36,112.70	100.00%	1	1	11	0.00842%	24.10%
Pap test	Eukaryotic translation initiation factor 2 subunit 2 GN=EIF2S2 PE=1 SV=2	IF2B_HUMAN	38,389.70	99.60%	1	1	1	0.00156%	3.60%
Tumor tissue	Eukaryotic translation initiation factor 2 subunit 2 GN=EIF2S2 PE=1 SV=2	IF2B_HUMAN	38,389.70	100.00%	8	11	12	0.00919%	22.50%
Tumor tissue	Eukaryotic translation initiation factor 2 subunit 3 GN=EIF2S3 PE=1 SV=3	IF2G_HUMAN	51,110.20	100.00%	9	12	16	0.01220%	25.20%
Tumor tissue	Eukaryotic translation initiation factor 2A GN=EIF2A PE=1 SV=3	sp|Q9BY44|EIF2A_HUMAN	64,991.20	100.00%	1	1	8	0.00612%	18.60%
Tumor tissue	Eukaryotic translation initiation factor 2D GN=EIF2D PE=1 SV=3	sp|P41214|EIF2D_HUMAN	64,707.50	100.00%	2	2	2	0.00153%	5.65%

Biological sample	Protein name	Protein accession numbers	Protein molecular weight (Da)	Protein identification probability	Exclusive unique peptide count	Exclusive unique spectrum count	Total spectrum count	Percentage of total spectra	Percentage sequence coverage
Pap test	Eukaryotic translation initiation factor 3 subunit A GN=EIF3A PE=1 SV=1	sp|Q14152|EIF3A_HUMAN	166,574.90	99.20%	1	1	1	0.00156%	1.09%
Tumor tissue	Eukaryotic translation initiation factor 3 subunit A GN=EIF3A PE=1 SV=1	sp|Q14152|EIF3A_HUMAN	166,574.90	100.00%	27	36	49	0.03750%	24.70%
Tumor tissue	Eukaryotic translation initiation factor 3 subunit E GN=EIF3E PE=1 SV=1	EIF3E_HUMAN	52,222.60	100.00%	10	14	19	0.01450%	29.40%
Tumor tissue	Eukaryotic translation initiation factor 3 subunit F GN=EIF3F PE=1 SV=1	EIF3F_HUMAN	37,563.50	100.00%	7	9	13	0.00995%	29.40%
Tumor tissue	Eukaryotic translation initiation factor 3 subunit G GN=EIF3G PE=1 SV=2	EIF3G_HUMAN	35,612.00	100.00%	1	1	7	0.00536%	24.70%
Tumor tissue	Eukaryotic translation initiation factor 3 subunit H GN=EIF3H PE=1 SV=1	EIF3H_HUMAN	41,581.70	100.00%	3	3	16	0.01220%	34.70%
Pap test	Eukaryotic translation initiation factor 3 subunit I GN=EIF3I PE=1 SV=1	EIF3I_HUMAN	36,502.00	99.20%	1	1	1	0.00156%	3.69%
Tumor tissue	Eukaryotic translation initiation factor 3 subunit I GN=EIF3I PE=1 SV=1	EIF3I_HUMAN	36,502.00	100.00%	7	9	11	0.00842%	32.60%
Pap test	Eukaryotic translation initiation factor 3 subunit K GN=EIF3K PE=1 SV=1	sp|Q9UBQ5|EIF3K_HUMAN	22,096.00	98.80%	1	1	1	0.00156%	5.73%
Tumor tissue	Eukaryotic translation initiation factor 3 subunit K GN=EIF3K PE=1 SV=1	sp|Q9UBQ5|EIF3K_HUMAN	22,096.00	100.00%	2	2	3	0.00230%	13.00%
Tumor tissue	Eukaryotic translation initiation factor 3 subunit L GN=EIF3L PE=1 SV=1	sp|Q9Y262|EIF3L_HUMAN	70,904.50	100.00%	9	12	18	0.01380%	24.10%
Tumor tissue	Eukaryotic translation initiation factor 3 subunit M GN=EIF3M PE=1 SV=1	sp|Q7L2H7|EIF3M_HUMAN	42,503.90	100.00%	8	11	12	0.00919%	26.50%

Biological sample	Protein name	Protein accession numbers	Protein molecular weight (Da)	Protein identification probability	Exclusive unique peptide count	Exclusive unique spectrum count	Total spectrum count	Percentage of total spectra	Percentage sequence coverage
Tumor tissue	Eukaryotic translation initiation factor 4 gamma 2 GN=EIF4G2 PE=1 SV=1	sp|P78344|IF4G2_HUMAN	102,366.80	100.00%	13	13	15	0.01150%	20.00%
Tumor tissue	Eukaryotic translation initiation factor 4B GN=EIF4B PE=1 SV=1	sp|P23588|IF4B_HUMAN	69,700.40	100.00%	2	3	3	0.00230%	4.71%
Pap test	Eukaryotic translation initiation factor 4E GN=EIF4E PE=1 SV=1	sp|P06730|IF4E_HUMAN	28,512.80	99.20%	1	1	1	0.00156%	4.49%
Tumor tissue	Eukaryotic translation initiation factor 4E GN=EIF4E PE=1 SV=1	sp|P06730|IF4E_HUMAN	28,512.80	100.00%	3	5	5	0.00383%	11.00%
Pap test	Eukaryotic translation initiation factor 4H GN=EIF4H PE=1 SV=5	sp|Q15056|IF4H_HUMAN	27,386.00	99.50%	1	1	1	0.00156%	5.24%
Swab	Eukaryotic translation initiation factor 4H GN=EIF4H PE=1 SV=5	sp|Q15056|IF4H_HUMAN	27,386.00	99.90%	1	1	2	0.00142%	5.24%
Tumor tissue	Eukaryotic translation initiation factor 4H GN=EIF4H PE=1 SV=5	sp|Q15056|IF4H_HUMAN	27,386.00	100.00%	2	3	4	0.00306%	10.90%
Tumor tissue	Eukaryotic translation initiation factor 5 GN=EIF5 PE=1 SV=2	IF5_HUMAN	49,223.40	100.00%	4	5	8	0.00612%	12.10%
Pap test	Eukaryotic translation initiation factor 5A (Fragment) GN=EIF5A PE=1 SV=8	sp|P63241|IF5A1_HUMAN	16,019.60	100.00%	1	2	2	0.00312%	8.22%
Swab	Eukaryotic translation initiation factor 5A (Fragment) GN=EIF5A PE=1 SV=8	sp|P63241|IF5A1_HUMAN	20,170.90	100.00%	3	5	7	0.00496%	28.80%
Tumor tissue	Eukaryotic translation initiation factor 5A (Fragment) GN=EIF5A PE=1 SV=8	sp|P63241|IF5A1_HUMAN	16,019.60	100.00%	4	6	11	0.00842%	20.50%
Tumor tissue	Eukaryotic translation initiation factor 5B GN=EIF5B PE=1 SV=1	IF2P_HUMAN	138,686.50	100.00%	13	15	17	0.01300%	16.60%
Pap test	Eukaryotic translation initiation factor 6 GN=EIF6 PE=1 SV=1	sp|P56537|IF6_HUMAN	26,598.20	100.00%	4	4	6	0.00936%	33.90%
Swab	Eukaryotic translation initiation factor 6 GN=EIF6 PE=1 SV=1	sp|P56537|IF6_HUMAN	26,598.20	100.00%	4	5	6	0.00425%	25.70%
Tumor tissue	Eukaryotic translation initiation factor 6 GN=EIF6 PE=1 SV=1	sp|P56537|IF6_HUMAN	26,598.20	100.00%	2	3	3	0.00230%	13.10%
Tumor tissue	Exocyst complex component 2 GN=EXOC2 PE=1 SV=1	EXOC2_HUMAN	104,069.50	100.00%	5	5	6	0.00459%	8.23%

Biological sample	Protein name	Protein accession numbers	Protein molecular weight (Da)	Protein identification probability	Exclusive unique peptide count	Exclusive unique spectrum count	Total spectrum count	Percentage of total spectra	Percentage sequence coverage
Tumor tissue	Exocyst complex component 4 GN=EXOC4 PE=1 SV=1	sp|Q96A65|EXOC4_HUMAN	110,502.50	100.00%	7	8	8	0.00612%	10.60%
Tumor tissue	Exocyst complex component 7 GN=EXOC7 PE=1 SV=1	sp|Q9UPT5|EXOC7_HUMAN	78,811.40	100.00%	3	4	5	0.00383%	8.66%
Tumor tissue	Exosome complex component MTR3 GN=EXOSC6 PE=1 SV=1	EXOS6_HUMAN	28,235.60	100.00%	4	4	6	0.00459%	24.30%
Tumor tissue	Exosome complex exonuclease RRP44 GN=DIS3 PE=1 SV=2	sp|Q9Y2L1|RRP44_HUMAN	109,005.70	100.00%	3	3	3	0.00230%	4.18%
Tumor tissue	Exosome component 10 GN=EXOSC10 PE=1 SV=2	sp|Q01780|EXOSX_HUMAN	100,833.90	100.00%	3	3	3	0.00230%	5.08%
Pap test	Exportin-1 GN=XPO1 PE=1 SV=1	XPO1_HUMAN	123,388.90	99.20%	1	1	1	0.00156%	1.12%
Swab	Exportin-1 GN=XPO1 PE=1 SV=1	XPO1_HUMAN	123,388.90	100.00%	4	4	5	0.00354%	5.60%
Tumor tissue	Exportin-1 GN=XPO1 PE=1 SV=1	XPO1_HUMAN	123,388.90	100.00%	13	16	17	0.01300%	14.60%
Tumor tissue	Exportin-5 GN=XPO5 PE=1 SV=1	XPO5_HUMAN	136,314.80	100.00%	6	7	11	0.00842%	6.73%
Tumor tissue	Exportin-7 GN=XPO7 PE=1 SV=3	XPO7_HUMAN	123,912.80	100.00%	2	2	3	0.00230%	3.40%
Swab	Exportin-T GN=XPOT PE=1 SV=2	XPOT_HUMAN	109,966.60	99.20%	1	1	1	0.00071%	1.46%
Tumor tissue	Exportin-T GN=XPOT PE=1 SV=2	XPOT_HUMAN	109,966.60	100.00%	5	6	6	0.00459%	7.90%
Tumor tissue	Extracellular sulfatase Sulf-1 GN=SULF1 PE=1 SV=1	SULF1_HUMAN	77,434.40	100.00%	3	3	3	0.00230%	5.53%
Pap test	Extracellular superoxide dismutase [Cu-Zn] GN=SOD3 PE=1 SV=2	SODE_HUMAN	25,850.30	100.00%	4	4	5	0.00780%	23.30%
Swab	Extracellular superoxide dismutase [Cu-Zn] GN=SOD3 PE=1 SV=2	SODE_HUMAN	25,850.30	100.00%	1	2	2	0.00142%	6.67%
Tumor tissue	Extracellular superoxide dismutase [Cu-Zn] GN=SOD3 PE=1 SV=2	SODE_HUMAN	25,850.30	100.00%	2	2	2	0.00153%	10.00%
Pap test	Ezrin GN=EZR PE=1 SV=3	EZRI_HUMAN	69,373.50	100.00%	7	10	27	0.04210%	31.10%
Swab	Ezrin GN=EZR PE=1 SV=3	EZRI_HUMAN	69,373.50	100.00%	5	6	19	0.01350%	17.90%
Tumor tissue	Ezrin GN=EZR PE=1 SV=3	EZRI_HUMAN	69,373.50	100.00%	8	12	41	0.03140%	29.00%
Tumor tissue	FACT complex subunit SPT16 GN=SUPT16H PE=1 SV=1	SP16H_HUMAN	119,917.40	100.00%	10	11	13	0.00995%	13.60%

Biological sample	Protein name	Protein accession numbers	Protein molecular weight (Da)	Protein identification probability	Exclusive unique peptide count	Exclusive unique spectrum count	Total spectrum count	Percentage of total spectra	Percentage sequence coverage
Tumor tissue	FACT complex subunit SSRP1 GN=SSRP1 PE=1 SV=1	SSRP1_HUMAN	81,077.60	100.00%	7	8	9	0.00689%	12.70%
Pap test	F-actin-capping protein subunit alpha-1 GN=CAPZA1 PE=1 SV=3	CAZA1_HUMAN	32,923.20	100.00%	2	3	9	0.01400%	15.70%
Swab	F-actin-capping protein subunit alpha-1 GN=CAPZA1 PE=1 SV=3	CAZA1_HUMAN	32,923.20	100.00%	6	7	19	0.01350%	43.40%
Tumor tissue	F-actin-capping protein subunit alpha-1 GN=CAPZA1 PE=1 SV=3	CAZA1_HUMAN	32,923.20	100.00%	6	12	33	0.02530%	40.20%
Pap test	F-actin-capping protein subunit alpha-2 GN=CAPZA2 PE=1 SV=3	sp|P47755|CAZA2_HUMAN	32,949.20	100.00%	5	5	7	0.01090%	36.40%
Swab	F-actin-capping protein subunit alpha-2 GN=CAPZA2 PE=1 SV=3	sp|P47755|CAZA2_HUMAN	32,949.20	100.00%	4	5	15	0.01060%	29.00%
Tumor tissue	F-actin-capping protein subunit alpha-2 GN=CAPZA2 PE=1 SV=3	sp|P47755|CAZA2_HUMAN	32,949.20	100.00%	5	9	20	0.01530%	40.20%
Tumor tissue	FAD synthase GN=FLAD1 PE=1 SV=1	sp|Q8NFF5|FAD1_HUMAN	50,142.20	100.00%	2	2	3	0.00230%	6.14%
Tumor tissue	Far upstream element-binding protein 1 GN=FUBP1 PE=1 SV=3	sp|Q96AE4|FUBP1_HUMAN	67,560.60	100.00%	1	1	19	0.01450%	25.90%
Pap test	Far upstream element-binding protein 2 GN=KHSRP PE=1 SV=4	FUBP2_HUMAN	73,115.60	100.00%	3	4	5	0.00780%	4.92%
Tumor tissue	Far upstream element-binding protein 2 GN=KHSRP PE=1 SV=4	FUBP2_HUMAN	73,115.60	100.00%	19	23	29	0.02220%	33.30%
Tumor tissue	Far upstream element-binding protein 3 GN=FUBP3 PE=1 SV=2	sp|Q96I24|FUBP3_HUMAN	61,639.90	100.00%	8	9	9	0.00689%	24.50%
Tumor tissue	FAS-associated factor 2 GN=FAF2 PE=1 SV=2	FAF2_HUMAN	52,624.40	100.00%	4	4	5	0.00383%	16.60%
Swab	Fascin GN=FSCN1 PE=1 SV=3	FSCN1_HUMAN	54,530.30	100.00%	4	4	4	0.00283%	12.80%
Tumor tissue	Fascin GN=FSCN1 PE=1 SV=3	FSCN1_HUMAN	54,530.30	100.00%	13	15	24	0.01840%	30.40%
Pap test	Fatty acid synthase GN=FASN PE=1 SV=3	FAS_HUMAN	273,427.10	100.00%	2	2	2	0.00312%	1.15%
Swab	Fatty acid synthase GN=FASN PE=1 SV=3	FAS_HUMAN	273,427.10	100.00%	4	4	5	0.00354%	2.15%
Tumor tissue	Fatty acid synthase GN=FASN PE=1 SV=3	FAS_HUMAN	273,427.10	100.00%	48	57	72	0.05510%	26.40%

Biological sample	Protein name	Protein accession numbers	Protein molecular weight (Da)	Protein identification probability	Exclusive unique peptide count	Exclusive unique spectrum count	Total spectrum count	Percentage of total spectra	Percentage sequence coverage
Pap test	Fatty acid-binding protein, epidermal GN=FABP5 PE=1 SV=3	FABP5_HUMAN	15,164.40	100.00%	6	12	37	0.05770%	53.30%
Swab	Fatty acid-binding protein, epidermal GN=FABP5 PE=1 SV=3	FABP5_HUMAN	15,164.40	100.00%	8	19	49	0.03470%	64.40%
Tumor tissue	Fatty acid-binding protein, epidermal GN=FABP5 PE=1 SV=3	FABP5_HUMAN	15,164.40	99.80%	1	1	1	0.00077%	7.41%
Swab	Fatty acid-binding protein, heart GN=FABP3 PE=1 SV=4	FABPH_HUMAN	14,787.40	100.00%	2	2	2	0.00142%	17.30%
Tumor tissue	Fatty acid-binding protein, heart GN=FABP3 PE=1 SV=4	FABPH_HUMAN	14,787.40	100.00%	4	5	6	0.00459%	35.30%
Tumor tissue	F-BAR domain only protein 2 GN=FCHO2 PE=1 SV=2	sp|Q0JRZ9|FCHO2_HUMAN	88,926.20	100.00%	2	2	2	0.00153%	4.32%
Tumor tissue	F-box only protein 22 GN=FBXO22 PE=1 SV=1	sp|Q8NEZ5|FBX22_HUMAN	44,508.30	100.00%	3	3	3	0.00230%	8.19%
Pap test	F-box only protein 50 GN=NCCRP1 PE=1 SV=1	FBX50_HUMAN	30,847.20	100.00%	7	11	24	0.03750%	33.10%
Swab	F-box only protein 50 GN=NCCRP1 PE=1 SV=1	FBX50_HUMAN	30,847.20	100.00%	5	8	16	0.01130%	26.20%
Pap test	F-box only protein 7 GN=FBXO7 PE=1 SV=1	sp|Q9Y3I1|FBX7_HUMAN	58,505.20	99.20%	1	1	1	0.00156%	2.49%
Tumor tissue	F-box only protein 7 GN=FBXO7 PE=1 SV=1	sp|Q9Y3I1|FBX7_HUMAN	58,505.20	100.00%	2	2	3	0.00230%	5.56%
Tumor tissue	F-box-like/WD repeat-containing protein TBL1XR1 GN=TBL1XR1 PE=1 SV=1	TBL1R_HUMAN	51,556.50	100.00%	3	5	8	0.00612%	14.80%
Pap test	Fc of IgG low affinity IIIa receptor isoform 1 GN=FCGR3A PE=1 SV=1	M9MML0_HUMAN	32,691.00	99.90%	1	1	4	0.00624%	7.93%
Swab	Fc of IgG low affinity IIIa receptor isoform 1 GN=FCGR3A PE=1 SV=1	M9MML0_HUMAN	32,691.00	100.00%	1	1	9	0.00638%	10.70%
Tumor tissue	Fc of IgG low affinity IIIa receptor isoform 1 GN=FCGR3A PE=1 SV=1	M9MML0_HUMAN	32,691.00	99.50%	1	1	3	0.00230%	7.93%
Tumor tissue	FERM, RhoGEF and pleckstrin domain-containing protein 1 GN=FARP1 PE=1 SV=1	sp|Q9Y4F1|FARP1_HUMAN	118,635.90	100.00%	1	1	11	0.00842%	9.67%
Pap test	Ferritin heavy chain GN=FTH1 PE=1 SV=2	FRIH_HUMAN	21,226.20	100.00%	6	10	17	0.02650%	57.40%

Biological sample	Protein name	Protein accession numbers	Protein molecular weight (Da)	Protein identification probability	Exclusive unique peptide count	Exclusive unique spectrum count	Total spectrum count	Percentage of total spectra	Percentage sequence coverage
Swab	Ferritin heavy chain GN=FTH1 PE=1 SV=2	FRIH_HUMAN	21,226.20	100.00%	5	9	23	0.01630%	51.90%
Tumor tissue	Ferritin heavy chain GN=FTH1 PE=1 SV=2	FRIH_HUMAN	21,226.20	100.00%	7	13	16	0.01220%	37.20%
Pap test	Ferritin light chain GN=FTL PE=1 SV=2	FRIL_HUMAN	20,020.60	100.00%	5	7	11	0.01720%	36.00%
Swab	Ferritin light chain GN=FTL PE=1 SV=2	FRIL_HUMAN	20,020.60	100.00%	7	12	24	0.01700%	40.00%
Tumor tissue	Ferritin light chain GN=FTL PE=1 SV=2	FRIL_HUMAN	20,020.60	100.00%	7	15	20	0.01530%	40.00%
Tumor tissue	Fibrillin-1 GN=FBN1 PE=1 SV=3	FBN1_HUMAN	312,226.00	100.00%	5	5	8	0.00612%	1.85%
Pap test	Fibrinogen beta chain GN=FGB PE=1 SV=2	FIBB_HUMAN	55,928.60	100.00%	19	46	118	0.18400%	55.60%
Swab	Fibrinogen beta chain GN=FGB PE=1 SV=2	FIBB_HUMAN	55,928.60	100.00%	24	53	133	0.09430%	57.80%
Tumor tissue	Fibrinogen beta chain GN=FGB PE=1 SV=2	FIBB_HUMAN	55,928.60	100.00%	17	50	95	0.07270%	45.00%
Pap test	Fibrinogen gamma chain GN=FGG PE=1 SV=1	sp|P02679|FIBG_HUMAN	52,339.20	100.00%	11	22	108	0.16900%	48.80%
Swab	Fibrinogen gamma chain GN=FGG PE=1 SV=1	sp|P02679|FIBG_HUMAN	52,339.20	100.00%	12	22	120	0.08500%	50.10%
Tumor tissue	Fibrinogen gamma chain GN=FGG PE=1 SV=1	sp|P02679|FIBG_HUMAN	52,339.20	100.00%	11	23	95	0.07270%	41.60%
Tumor tissue	Fibromodulin GN=FMOD PE=1 SV=2	FMOD_HUMAN	43,180.40	100.00%	3	5	8	0.00612%	10.90%
Tumor tissue	Fibronectin type III domain-containing protein 3B GN=FNDC3B PE=1 SV=2	sp|Q53EP0|FND3B_HUMAN	132,889.60	100.00%	5	6	6	0.00459%	6.56%
Pap test	Fibulin-1 GN=FBLN1 PE=1 SV=4	sp|P23142|FBLN1_HUMAN	77,211.60	100.00%	2	3	30	0.04680%	31.00%
Swab	Fibulin-1 GN=FBLN1 PE=1 SV=4	sp|P23142|FBLN1_HUMAN	77,211.60	100.00%	3	5	47	0.03330%	34.40%
Tumor tissue	Fibulin-1 GN=FBLN1 PE=1 SV=4	sp|P23142|FBLN1_HUMAN	77,211.60	100.00%	5	11	35	0.02680%	17.20%
Tumor tissue	Fibulin-5 GN=FBLN5 PE=1 SV=1	FBLN5_HUMAN	50,880.80	100.00%	3	3	5	0.00383%	7.59%
Pap test	Filaggrin GN=FLG PE=1 SV=3	FILA_HUMAN	435,145.70	99.90%	1	1	1	0.00156%	0.59%

Biological sample	Protein name	Protein accession numbers	Protein molecular weight (Da)	Protein identification probability	Exclusive unique peptide count	Exclusive unique spectrum count	Total spectrum count	Percentage of total spectra	Percentage sequence coverage
Tumor tissue	Filaggrin GN=FLG PE=1 SV=3	FILA_HUMAN	435,145.70	100.00%	6	7	8	0.00612%	2.83%
Swab	Filamin A-interacting protein 1-like GN=FILIP1L PE=1 SV=2	sp|Q4L180|FIL1L_HUMAN	130,385.60	99.90%	1	1	1	0.00071%	1.67%
Tumor tissue	Filamin A-interacting protein 1-like GN=FILIP1L PE=1 SV=2	sp|Q4L180|FIL1L_HUMAN	130,385.60	100.00%	3	3	13	0.00995%	14.70%
Tumor tissue	Filamin-binding LIM protein 1 (Fragment) GN=FBLIM1 PE=1 SV=1	E7EWE8_HUMAN	15,933.90	100.00%	2	3	3	0.00230%	15.30%
Tumor tissue	FK506-binding protein 15 (Fragment) GN=FKBP15 PE=1 SV=1	sp|Q5T1M5|FKB15_HUMAN	136,274.10	100.00%	9	9	9	0.00689%	10.20%
Swab	Flap endonuclease 1 GN=FEN1 PE=1 SV=1	sp|P39748|FEN1_HUMAN	42,594.20	99.90%	1	1	1	0.00071%	5.26%
Tumor tissue	Flap endonuclease 1 GN=FEN1 PE=1 SV=1	sp|P39748|FEN1_HUMAN	42,594.20	100.00%	5	7	7	0.00536%	20.80%
Pap test	Flavin reductase (NADPH) GN=BLVRB PE=1 SV=3	BLVRB_HUMAN	22,118.70	100.00%	3	4	4	0.00624%	18.90%
Swab	Flavin reductase (NADPH) GN=BLVRB PE=1 SV=3	BLVRB_HUMAN	22,118.70	100.00%	3	4	11	0.00780%	18.90%
Tumor tissue	Flavin reductase (NADPH) GN=BLVRB PE=1 SV=3	BLVRB_HUMAN	22,118.70	100.00%	6	10	14	0.01070%	47.10%
Tumor tissue	Flotillin-1 GN=FLOT1 PE=1 SV=3	sp|O75955|FLOT1_HUMAN	47,354.80	100.00%	12	15	17	0.01300%	39.30%
Pap test	Flotillin-2 GN=FLOT2 PE=1 SV=1	FLOT2_HUMAN	47,142.10	100.00%	1	1	1	0.00156%	3.93%
Tumor tissue	Flotillin-2 GN=FLOT2 PE=1 SV=1	FLOT2_HUMAN	47,142.10	100.00%	12	17	20	0.01530%	32.10%
Pap test	Folate receptor alpha GN=FOLR1 PE=1 SV=3	FOLR1_HUMAN	29,818.50	100.00%	1	1	2	0.00312%	4.28%
Swab	Folate receptor alpha GN=FOLR1 PE=1 SV=3	FOLR1_HUMAN	29,818.50	100.00%	2	2	4	0.00283%	7.78%
Tumor tissue	Forkhead box protein K1 GN=FOXK1 PE=1 SV=1	sp|P85037|FOXK1_HUMAN	75,456.60	100.00%	3	3	3	0.00230%	4.77%
Tumor tissue	Formin-binding protein 1-like GN=FNBP1L PE=1 SV=1	sp|Q5T0N5|FBP1L_HUMAN	70,527.60	100.00%	2	2	2	0.00153%	3.12%
Tumor tissue	Fragile X mental retardation syndrome-related protein 1 GN=FXR1 PE=1 SV=1	sp|P51114|FXR1_HUMAN	68,327.30	100.00%	7	8	11	0.00842%	24.50%

Biological sample	Protein name	Protein accession numbers	Protein molecular weight (Da)	Protein identification probability	Exclusive unique peptide count	Exclusive unique spectrum count	Total spectrum count	Percentage of total spectra	Percentage sequence coverage
Tumor tissue	Fragile X mental retardation syndrome-related protein 2 GN=FXR2 PE=1 SV=2	FXR2_HUMAN	74,224.00	99.80%	1	1	2	0.00153%	4.75%
Pap test	Fructose-1,6-bisphosphatase 1 GN=FBP1 PE=1 SV=5	F16P1_HUMAN	36,843.20	100.00%	2	2	3	0.00468%	8.28%
Swab	Fructose-1,6-bisphosphatase 1 GN=FBP1 PE=1 SV=5	F16P1_HUMAN	36,843.20	100.00%	3	6	14	0.00992%	15.40%
Tumor tissue	Fructose-1,6-bisphosphatase 1 GN=FBP1 PE=1 SV=5	F16P1_HUMAN	36,843.20	100.00%	9	12	17	0.01300%	35.50%
Swab	Fructose-1,6-bisphosphatase isozyme 2 GN=FBP2 PE=1 SV=2	F16P2_HUMAN	36,743.90	99.60%	1	1	2	0.00142%	9.44%
Tumor tissue	Fructose-2,6-bisphosphatase TIGAR GN=TIGAR PE=1 SV=1	TIGAR_HUMAN	30,063.10	100.00%	2	2	2	0.00153%	15.20%
Pap test	Fructose-bisphosphate aldolase C GN=ALDOC PE=1 SV=2	ALDOC_HUMAN	39,456.20	100.00%	4	6	24	0.03750%	27.50%
Swab	Fructose-bisphosphate aldolase C GN=ALDOC PE=1 SV=2	ALDOC_HUMAN	39,456.20	100.00%	3	5	38	0.02690%	22.80%
Tumor tissue	Fructose-bisphosphate aldolase C GN=ALDOC PE=1 SV=2	ALDOC_HUMAN	39,456.20	100.00%	6	8	18	0.01380%	22.30%
Pap test	Fumarylacetoacetase GN=FAH PE=1 SV=2	sp|P16930|FAAA_HUMAN	46,375.60	100.00%	4	4	4	0.00624%	12.20%
Tumor tissue	Fumarylacetoacetase GN=FAH PE=1 SV=2	sp|P16930|FAAA_HUMAN	46,375.60	100.00%	4	4	5	0.00383%	13.10%
Tumor tissue	Fumarylacetoacetate hydrolase domain-containing protein 2A GN=FAHD2A PE=1 SV=1	FAH2A_HUMAN	34,595.20	100.00%	3	3	3	0.00230%	15.30%
Tumor tissue	G patch domain and KOW motifs-containing protein GN=GPKOW PE=1 SV=2	GPKOW_HUMAN	52,228.60	100.00%	3	3	3	0.00230%	8.61%
Swab	Galactokinase GN=GALK1 PE=1 SV=1	sp|P51570|GALK1_HUMAN	42,272.10	100.00%	3	3	5	0.00354%	12.80%
Tumor tissue	Galactokinase GN=GALK1 PE=1 SV=1	sp|P51570|GALK1_HUMAN	42,272.10	100.00%	4	5	10	0.00766%	21.40%
Tumor tissue	Galactose-1-phosphate uridylyltransferase GN=GALT PE=1 SV=3	sp|P07902|GALT_HUMAN	43,362.90	100.00%	2	2	2	0.00153%	6.60%

Biological sample	Protein name	Protein accession numbers	Protein molecular weight (Da)	Protein identification probability	Exclusive unique peptide count	Exclusive unique spectrum count	Total spectrum count	Percentage of total spectra	Percentage sequence coverage
Pap test	Galectin-1 GN=LGALS1 PE=1 SV=2	LEG1_HUMAN	14,715.80	100.00%	4	4	5	0.00780%	34.10%
Swab	Galectin-1 GN=LGALS1 PE=1 SV=2	LEG1_HUMAN	14,715.80	100.00%	4	7	12	0.00850%	39.30%
Tumor tissue	Galectin-1 GN=LGALS1 PE=1 SV=2	LEG1_HUMAN	14,715.80	100.00%	5	10	29	0.02220%	35.60%
Pap test	Galectin-3 GN=LGALS3 PE=1 SV=5	LEG3_HUMAN	26,152.80	100.00%	4	5	6	0.00936%	18.40%
Swab	Galectin-3 GN=LGALS3 PE=1 SV=5	LEG3_HUMAN	26,152.80	100.00%	4	6	10	0.00709%	19.60%
Tumor tissue	Galectin-3 GN=LGALS3 PE=1 SV=5	LEG3_HUMAN	26,152.80	100.00%	4	11	15	0.01150%	21.20%
Pap test	Galectin-3-binding protein GN=LGALS3BP PE=1 SV=1	LG3BP_HUMAN	65,332.10	100.00%	9	12	23	0.03590%	23.10%
Swab	Galectin-3-binding protein GN=LGALS3BP PE=1 SV=1	LG3BP_HUMAN	65,332.10	100.00%	6	10	19	0.01350%	16.90%
Tumor tissue	Galectin-3-binding protein GN=LGALS3BP PE=1 SV=1	LG3BP_HUMAN	65,332.10	100.00%	9	12	19	0.01450%	19.10%
Pap test	Galectin-7 GN=LGALS7 PE=1 SV=2	LEG7_HUMAN	15,074.70	100.00%	4	4	4	0.00624%	36.80%
Tumor tissue	Gamma-aminobutyric acid receptor-associated protein-like 2 GN=GABARAPL2 PE=1 SV=1	GBRL2_HUMAN	10,401.40	100.00%	2	2	2	0.00153%	16.20%
Swab	Gamma-enolase GN=ENO2 PE=1 SV=3	sp|P09104|ENOG_HUMAN	47,269.70	100.00%	1	1	12	0.00850%	12.70%
Tumor tissue	Gamma-enolase GN=ENO2 PE=1 SV=3	sp|P09104|ENOG_HUMAN	47,269.70	100.00%	5	7	19	0.01450%	24.90%
Pap test	Gamma-glutamyl hydrolase GN=GGH PE=1 SV=2	GGH_HUMAN	35,965.90	99.90%	1	1	1	0.00156%	3.77%
Tumor tissue	Gamma-glutamyl hydrolase GN=GGH PE=1 SV=2	GGH_HUMAN	35,965.90	100.00%	5	5	6	0.00459%	21.10%
Pap test	Gamma-glutamylcyclotransferase GN=GGCT PE=1 SV=1	sp|O75223|GGCT_HUMAN	21,008.00	100.00%	5	7	10	0.01560%	29.30%
Swab	Gamma-glutamylcyclotransferase GN=GGCT PE=1 SV=1	sp|O75223|GGCT_HUMAN	21,008.00	100.00%	3	3	7	0.00496%	21.80%
Tumor tissue	Gamma-glutamylcyclotransferase GN=GGCT PE=1 SV=1	sp|O75223|GGCT_HUMAN	21,008.00	100.00%	3	4	5	0.00383%	17.00%
Pap test	Gamma-synuclein GN=SNCG PE=1 SV=2	SYUG_HUMAN	13,329.60	100.00%	2	2	2	0.00312%	23.60%
Swab	Gamma-synuclein GN=SNCG PE=1 SV=2	SYUG_HUMAN	13,329.60	100.00%	4	5	6	0.00425%	42.50%

Biological sample	Protein name	Protein accession numbers	Protein molecular weight (Da)	Protein identification probability	Exclusive unique peptide count	Exclusive unique spectrum count	Total spectrum count	Percentage of total spectra	Percentage sequence coverage
Tumor tissue	Gamma-synuclein GN=SNCG PE=1 SV=2	SYUG_HUMAN	13,329.60	100.00%	2	2	2	0.00153%	24.40%
Tumor tissue	Gamma-tubulin complex component 3 GN=TUBGCP3 PE=1 SV=2	sp|Q96CW5|GCP3_HUMAN	103,574.60	100.00%	1	1	2	0.00153%	3.42%
Pap test	Ganglioside GM2 activator GN=GM2A PE=1 SV=4	SAP3_HUMAN	20,839.20	100.00%	2	3	3	0.00468%	10.90%
Swab	Ganglioside GM2 activator GN=GM2A PE=1 SV=4	SAP3_HUMAN	20,839.20	100.00%	3	3	4	0.00283%	15.00%
Tumor tissue	Ganglioside GM2 activator GN=GM2A PE=1 SV=4	SAP3_HUMAN	20,839.20	100.00%	1	1	2	0.00153%	9.33%
Tumor tissue	Gap junction alpha-1 protein GN=GJA1 PE=1 SV=2	CXA1_HUMAN	43,009.40	100.00%	3	4	4	0.00306%	16.00%
Swab	Gasdermin domain containing 1, isoform CRA_d GN=GSDMD PE=1 SV=1	GSDMD_HUMAN	52,800.90	99.80%	1	1	1	0.00071%	2.63%
Tumor tissue	Gasdermin domain containing 1, isoform CRA_d GN=GSDMD PE=1 SV=1	GSDMD_HUMAN	57,734.80	100.00%	3	3	3	0.00230%	7.89%
Tumor tissue	GDH/6PGL endoplasmic bifunctional protein GN=H6PD PE=1 SV=2	G6PE_HUMAN	90,174.10	100.00%	17	21	23	0.01760%	29.10%
Tumor tissue	GDP-fucose protein O-fucosyltransferase 1 GN=POFUT1 PE=1 SV=1	sp|Q9H488|OFUT1_HUMAN	43,956.20	100.00%	3	6	7	0.00536%	13.10%
Pap test	GDP-L-fucose synthase GN=TSTA3 PE=1 SV=1	FCL_HUMAN	35,892.60	98.20%	1	1	1	0.00156%	3.74%
Swab	GDP-L-fucose synthase GN=TSTA3 PE=1 SV=1	FCL_HUMAN	35,892.60	100.00%	4	4	5	0.00354%	19.30%
Tumor tissue	GDP-L-fucose synthase GN=TSTA3 PE=1 SV=1	FCL_HUMAN	35,892.60	100.00%	5	7	8	0.00612%	23.40%
Pap test	GDP-mannose 4,6 dehydratase GN=GMDS PE=1 SV=1	sp|O60547|GMDS_HUMAN	41,950.00	100.00%	2	2	2	0.00312%	8.33%
Swab	GDP-mannose 4,6 dehydratase GN=GMDS PE=1 SV=1	sp|O60547|GMDS_HUMAN	41,950.00	100.00%	4	4	4	0.00283%	15.90%

Biological sample	Protein name	Protein accession numbers	Protein molecular weight (Da)	Protein identification probability	Exclusive unique peptide count	Exclusive unique spectrum count	Total spectrum count	Percentage of total spectra	Percentage sequence coverage
Tumor tissue	GDP-mannose 4,6 dehydratase GN=GMDS PE=1 SV=1	sp|O60547|GMDS_HUMAN	41,950.00	100.00%	2	2	2	0.00153%	8.60%
Pap test	Gelsolin GN=GSN PE=1 SV=1	sp|P06396|GELS_HUMAN	85,697.80	100.00%	12	19	54	0.08430%	30.60%
Swab	Gelsolin GN=GSN PE=1 SV=1	sp|P06396|GELS_HUMAN	85,697.80	100.00%	16	31	97	0.06870%	37.20%
Tumor tissue	Gelsolin GN=GSN PE=1 SV=1	sp|P06396|GELS_HUMAN	85,697.80	100.00%	13	25	70	0.05360%	41.60%
Tumor tissue	Gem-associated protein 5 GN=GEMIN5 PE=1 SV=3	GEMI5_HUMAN	168,590.80	100.00%	2	2	2	0.00153%	1.66%
Tumor tissue	General transcription factor IIF subunit 1 GN=GTF2F1 PE=1 SV=2	T2FA_HUMAN	58,241.90	100.00%	2	3	4	0.00306%	9.86%
Swab	Glia maturation factor beta GN=GMFB PE=1 SV=2	GMFB_HUMAN	16,713.60	100.00%	3	3	5	0.00354%	31.00%
Tumor tissue	Glia maturation factor beta GN=GMFB PE=1 SV=2	GMFB_HUMAN	16,713.60	100.00%	1	1	3	0.00230%	14.10%
Pap test	Glia maturation factor gamma GN=GMFG PE=1 SV=1	GMFG_HUMAN	12,964.90	99.40%	1	1	1	0.00156%	9.15%
Swab	Glia maturation factor gamma GN=GMFG PE=1 SV=1	GMFG_HUMAN	12,964.90	100.00%	2	2	4	0.00283%	21.80%
Tumor tissue	Glia maturation factor gamma GN=GMFG PE=1 SV=1	GMFG_HUMAN	12,964.90	100.00%	1	1	3	0.00230%	15.50%
Tumor tissue	Glucosamine 6-phosphate N-acetyltransferase (Fragment) GN=GNPNAT1 PE=1 SV=1	GNA1_HUMAN	18,057.00	100.00%	2	2	3	0.00230%	17.80%
Tumor tissue	Glucosamine-6-phosphate isomerase 1 GN=GNPDA1 PE=1 SV=1	sp|P46926|GNPI1_HUMAN	32,669.40	100.00%	1	1	11	0.00842%	33.90%
Swab	Glucose 1,6-bisphosphate synthase GN=PGM2L1 PE=1 SV=3	PGM2L_HUMAN	70,443.20	99.80%	1	1	1	0.00071%	1.61%
Tumor tissue	Glucose 1,6-bisphosphate synthase GN=PGM2L1 PE=1 SV=3	PGM2L_HUMAN	70,443.20	100.00%	4	4	4	0.00306%	8.84%
Pap test	Glucose-6-phosphate isomerase (Fragment) GN=GPI PE=1 SV=1	sp|P06744|G6PI_HUMAN	64,826.50	100.00%	1	1	34	0.05310%	22.90%
Swab	Glucose-6-phosphate isomerase (Fragment) GN=GPI PE=1 SV=1	sp|P06744|G6PI_HUMAN	64,826.50	100.00%	2	4	53	0.03760%	29.00%
Tumor tissue	Glucose-6-phosphate isomerase (Fragment) GN=GPI PE=1 SV=1	sp|P06744|G6PI_HUMAN	64,826.50	100.00%	3	11	75	0.05740%	41.70%

Biological sample	Protein name	Protein accession numbers	Protein molecular weight (Da)	Protein identification probability	Exclusive unique peptide count	Exclusive unique spectrum count	Total spectrum count	Percentage of total spectra	Percentage sequence coverage
Tumor tissue	Glucose-induced degradation protein 8 homolog GN=GID8 PE=1 SV=1	GID8_HUMAN	26,749.60	100.00%	3	3	4	0.00306%	22.40%
Pap test	Glucosidase 2 subunit beta GN=PRKCSH PE=1 SV=1	K7ELL7_HUMAN	60,192.50	100.00%	7	7	7	0.01090%	16.80%
Swab	Glucosidase 2 subunit beta GN=PRKCSH PE=1 SV=1	K7ELL7_HUMAN	60,192.50	100.00%	5	5	6	0.00425%	12.70%
Tumor tissue	Glucosidase 2 subunit beta GN=PRKCSH PE=1 SV=1	K7ELL7_HUMAN	60,192.50	100.00%	10	15	18	0.01380%	22.80%
Tumor tissue	Glucosylceramidase GN=GBA PE=1 SV=1	sp|P04062|GLCM_HUMAN	59,664.60	100.00%	2	2	2	0.00153%	3.92%
Tumor tissue	Glutamate dehydrogenase 1, mitochondrial GN=GLUD1 PE=1 SV=2	sp|P00367|DHE3_HUMAN	61,399.70	100.00%	3	4	33	0.02530%	34.20%
Swab	Glutamate--cysteine ligase catalytic subunit GN=GCLC PE=1 SV=1	GSH1_HUMAN	68,631.90	99.90%	1	1	4	0.00283%	4.84%
Swab	Glutamate--cysteine ligase regulatory subunit GN=GCLM PE=1 SV=1	sp|P48507|GSH0_HUMAN	30,727.30	99.60%	1	1	1	0.00071%	4.74%
Tumor tissue	Glutamate--cysteine ligase regulatory subunit GN=GCLM PE=1 SV=1	sp|P48507|GSH0_HUMAN	30,727.30	100.00%	3	3	3	0.00230%	15.30%
Tumor tissue	Glutamate-rich WD repeat-containing protein 1 (Fragment) GN=GRWD1 PE=1 SV=1	M0QX71_HUMAN	25,477.00	100.00%	2	3	3	0.00230%	14.00%
Pap test	Glutamine synthetase GN=GLUL PE=1 SV=4	GLNA_HUMAN	42,064.90	100.00%	9	15	31	0.04840%	27.90%
Swab	Glutamine synthetase GN=GLUL PE=1 SV=4	GLNA_HUMAN	42,064.90	100.00%	8	13	25	0.01770%	25.70%
Tumor tissue	Glutamine synthetase GN=GLUL PE=1 SV=4	GLNA_HUMAN	42,064.90	99.30%	1	1	1	0.00077%	2.95%
Swab	Glutamine-dependent NAD(+) synthetase GN=NADSYN1 PE=1 SV=1	sp|Q6IA69|NADE_HUMAN	16,147.30	100.00%	2	2	3	0.00213%	6.87%

Biological sample	Protein name	Protein accession numbers	Protein molecular weight (Da)	Protein identification probability	Exclusive unique peptide count	Exclusive unique spectrum count	Total spectrum count	Percentage of total spectra	Percentage sequence coverage
Swab	Glutamine--fructose-6-phosphate aminotransferase [isomerizing] 1 GN=GFPT1 PE=1 SV=3	sp|Q06210|GFPT1_HUMAN	78,807.70	100.00%	2	2	3	0.00213%	4.15%
Tumor tissue	Glutamine--fructose-6-phosphate aminotransferase [isomerizing] 1 GN=GFPT1 PE=1 SV=3	sp|Q06210|GFPT1_HUMAN	78,807.70	100.00%	9	10	12	0.00919%	21.20%
Tumor tissue	Glutamine-rich protein 1 GN=QRICH1 PE=1 SV=1	QRIC1_HUMAN	86,433.20	100.00%	3	3	3	0.00230%	6.19%
Tumor tissue	Glutamine--tRNA ligase GN=QARS PE=1 SV=1	sp|P47897|SYQ_HUMAN	87,799.90	100.00%	1	1	26	0.01990%	31.70%
Tumor tissue	Glutamyl aminopeptidase GN=ENPEP PE=1 SV=3	AMPE_HUMAN	109,247.20	100.00%	2	2	2	0.00153%	2.61%
Swab	Glutaredoxin-1 GN=GLRX PE=1 SV=2	GLRX1_HUMAN	11,775.80	100.00%	3	4	7	0.00496%	31.10%
Tumor tissue	Glutaredoxin-1 GN=GLRX PE=1 SV=2	GLRX1_HUMAN	11,775.80	99.90%	2	2	2	0.00153%	11.30%
Swab	Glutaredoxin-3 GN=GLRX3 PE=1 SV=2	GLRX3_HUMAN	37,432.80	100.00%	1	1	1	0.00071%	3.58%
Tumor tissue	Glutaredoxin-3 GN=GLRX3 PE=1 SV=2	GLRX3_HUMAN	37,432.80	100.00%	7	7	7	0.00536%	25.40%
Tumor tissue	Glutaryl-CoA dehydrogenase, mitochondrial GN=GCDH PE=1 SV=1	sp|Q92947|GCDH_HUMAN	48,128.50	100.00%	3	3	4	0.00306%	15.80%
Pap test	Glutathione peroxidase GN=GPX1 PE=1 SV=1	A0A087WUQ6_HUMAN	21,938.60	100.00%	9	11	16	0.02500%	54.50%
Swab	Glutathione peroxidase GN=GPX1 PE=1 SV=1	A0A087WUQ6_HUMAN	21,938.60	100.00%	4	4	7	0.00496%	30.20%
Tumor tissue	Glutathione peroxidase GN=GPX1 PE=1 SV=1	A0A087WUQ6_HUMAN	21,938.60	100.00%	7	9	14	0.01070%	37.10%
Pap test	Glutathione peroxidase GN=GPX3 PE=1 SV=1	GPX3_HUMAN	25,403.10	100.00%	2	2	2	0.00312%	12.00%
Swab	Glutathione peroxidase GN=GPX3 PE=1 SV=1	GPX3_HUMAN	25,403.10	100.00%	2	2	6	0.00425%	12.00%
Tumor tissue	Glutathione peroxidase GN=GPX3 PE=1 SV=1	GPX3_HUMAN	25,403.10	100.00%	1	1	3	0.00230%	11.60%
Tumor tissue	Glutathione peroxidase GN=GPX4 PE=1 SV=1	A0A087WT12_HUMAN	26,948.60	100.00%	3	4	4	0.00306%	19.30%

Biological sample	Protein name	Protein accession numbers	Protein molecular weight (Da)	Protein identification probability	Exclusive unique peptide count	Exclusive unique spectrum count	Total spectrum count	Percentage of total spectra	Percentage sequence coverage
Tumor tissue	Glutathione peroxidase 7 GN=GPX7 PE=1 SV=1	GPX7_HUMAN	20,995.80	100.00%	2	3	4	0.00306%	11.20%
Pap test	Glutathione reductase, mitochondrial GN=GSR PE=1 SV=2	sp|P00390|GSHR_HUMAN	56,257.40	100.00%	14	23	38	0.05930%	45.60%
Swab	Glutathione reductase, mitochondrial GN=GSR PE=1 SV=2	sp|P00390|GSHR_HUMAN	56,257.40	100.00%	6	7	14	0.00992%	15.50%
Tumor tissue	Glutathione reductase, mitochondrial GN=GSR PE=1 SV=2	sp|P00390|GSHR_HUMAN	56,257.40	100.00%	7	9	10	0.00766%	21.60%
Tumor tissue	Glutathione S-transferase GN=GSTM2 PE=1 SV=1	sp|P28161|GSTM2_HUMAN	21,364.40	100.00%	3	5	9	0.00689%	30.60%
Pap test	Glutathione S-transferase kappa 1 GN=GSTK1 PE=1 SV=3	sp|Q9Y2Q3|GSTK1_HUMAN	25,498.10	99.20%	1	1	1	0.00156%	7.08%
Swab	Glutathione S-transferase kappa 1 GN=GSTK1 PE=1 SV=3	sp|Q9Y2Q3|GSTK1_HUMAN	25,498.10	100.00%	2	2	3	0.00213%	13.30%
Tumor tissue	Glutathione S-transferase kappa 1 GN=GSTK1 PE=1 SV=3	sp|Q9Y2Q3|GSTK1_HUMAN	25,498.10	100.00%	10	17	24	0.01840%	59.70%
Swab	Glutathione S-transferase Mu 3 GN=GSTM3 PE=1 SV=3	GSTM3_HUMAN	26,561.10	99.80%	1	1	2	0.00142%	9.33%
Tumor tissue	Glutathione S-transferase Mu 3 GN=GSTM3 PE=1 SV=3	GSTM3_HUMAN	26,561.10	100.00%	2	2	3	0.00230%	16.40%
Pap test	Glutathione S-transferase omega-1 GN=GSTO1 PE=1 SV=2	sp|P78417|GSTO1_HUMAN	27,567.20	100.00%	5	5	7	0.01090%	22.40%
Swab	Glutathione S-transferase omega-1 GN=GSTO1 PE=1 SV=2	sp|P78417|GSTO1_HUMAN	27,567.20	100.00%	3	3	6	0.00425%	13.30%
Tumor tissue	Glutathione S-transferase omega-1 GN=GSTO1 PE=1 SV=2	sp|P78417|GSTO1_HUMAN	27,567.20	100.00%	7	8	12	0.00919%	31.50%
Pap test	Glutathione S-transferase P GN=GSTP1 PE=1 SV=2	GSTP1_HUMAN	23,356.70	100.00%	9	17	49	0.07650%	60.50%
Swab	Glutathione S-transferase P GN=GSTP1 PE=1 SV=2	GSTP1_HUMAN	23,356.70	100.00%	10	27	102	0.07230%	61.00%
Tumor tissue	Glutathione S-transferase P GN=GSTP1 PE=1 SV=2	GSTP1_HUMAN	23,356.70	100.00%	11	29	78	0.05970%	61.90%
Tumor tissue	Glutathione S-transferase theta-2 GN=GSTT2 PE=3 SV=1	GSTT2_HUMAN	27,384.10	100.00%	4	5	6	0.00459%	23.00%
Pap test	Glutathione synthetase GN=GSS PE=1 SV=1	sp|P48637|GSHB_HUMAN	52,385.90	100.00%	7	7	9	0.01400%	18.10%

Biological sample	Protein name	Protein accession numbers	Protein molecular weight (Da)	Protein identification probability	Exclusive unique peptide count	Exclusive unique spectrum count	Total spectrum count	Percentage of total spectra	Percentage sequence coverage
Swab	Glutathione synthetase GN=GSS PE=1 SV=1	sp|P48637|GSHB_HUMAN	52,385.90	100.00%	8	8	9	0.00638%	24.10%
Tumor tissue	Glutathione synthetase GN=GSS PE=1 SV=1	sp|P48637|GSHB_HUMAN	52,385.90	100.00%	7	8	9	0.00689%	23.00%
Pap test	Glyceraldehyde-3-phosphate dehydrogenase GN=GAPDH PE=1 SV=3	sp|P04406|G3P_HUMAN	36,053.40	100.00%	11	27	64	0.09990%	49.90%
Swab	Glyceraldehyde-3-phosphate dehydrogenase GN=GAPDH PE=1 SV=3	sp|P04406|G3P_HUMAN	36,053.40	100.00%	15	34	180	0.12800%	51.00%
Tumor tissue	Glyceraldehyde-3-phosphate dehydrogenase GN=GAPDH PE=1 SV=3	sp|P04406|G3P_HUMAN	36,053.40	100.00%	18	73	400	0.30600%	61.50%
Swab	Glycerol-3-phosphate dehydrogenase 1-like protein GN=GPD1L PE=1 SV=1	GPD1L_HUMAN	38,419.30	100.00%	2	2	3	0.00213%	7.69%
Tumor tissue	Glycerol-3-phosphate dehydrogenase 1-like protein GN=GPD1L PE=1 SV=1	GPD1L_HUMAN	38,419.30	100.00%	3	3	4	0.00306%	10.30%
Pap test	Glycerol-3-phosphate dehydrogenase, mitochondrial GN=GPD2 PE=1 SV=3	sp|P43304|GPDM_HUMAN	80,854.60	100.00%	4	5	5	0.00780%	8.12%
Tumor tissue	Glycerol-3-phosphate dehydrogenase, mitochondrial GN=GPD2 PE=1 SV=3	sp|P43304|GPDM_HUMAN	80,854.60	100.00%	9	11	11	0.00842%	19.00%
Tumor tissue	Glycerol-3-phosphate phosphatase GN=PGP PE=1 SV=1	PGP_HUMAN	34,006.70	100.00%	2	2	2	0.00153%	7.79%
Tumor tissue	Glycine--tRNA ligase GN=GARS PE=1 SV=3	SYG_HUMAN	83,167.60	100.00%	12	19	25	0.01910%	22.70%
Pap test	Glycogen debranching enzyme GN=AGL PE=1 SV=3	sp|P35573|GDE_HUMAN	174,767.60	100.00%	3	3	3	0.00468%	2.74%
Swab	Glycogen debranching enzyme GN=AGL PE=1 SV=3	sp|P35573|GDE_HUMAN	174,767.60	100.00%	2	2	3	0.00213%	1.37%
Tumor tissue	Glycogen debranching enzyme GN=AGL PE=1 SV=3	sp|P35573|GDE_HUMAN	174,767.60	100.00%	8	8	8	0.00612%	6.85%

Biological sample	Protein name	Protein accession numbers	Protein molecular weight (Da)	Protein identification probability	Exclusive unique peptide count	Exclusive unique spectrum count	Total spectrum count	Percentage of total spectra	Percentage sequence coverage
Pap test	Glycogen phosphorylase, brain form GN=PYGB PE=1 SV=5	PYGB_HUMAN	96,698.30	100.00%	6	6	9	0.01400%	9.61%
Swab	Glycogen phosphorylase, brain form GN=PYGB PE=1 SV=5	PYGB_HUMAN	96,698.30	100.00%	7	7	7	0.00496%	11.30%
Tumor tissue	Glycogen phosphorylase, brain form GN=PYGB PE=1 SV=5	PYGB_HUMAN	96,698.30	100.00%	15	17	26	0.01990%	24.90%
Tumor tissue	Glycogen synthase kinase-3 alpha GN=GSK3A PE=1 SV=2	GSK3A_HUMAN	44,920.70	100.00%	2	2	3	0.00230%	9.98%
Tumor tissue	Glycosylphosphatidylinositol anchor attachment 1 protein GN=GPAA1 PE=1 SV=3	sp|O43292|GPAA1_HUMAN	67,625.20	100.00%	2	2	2	0.00153%	3.86%
Pap test	Glyoxalase domain-containing protein 4 GN=GLOD4 PE=1 SV=1	F6TLX2_HUMAN	54,721.00	100.00%	3	3	11	0.01720%	12.70%
Swab	Glyoxalase domain-containing protein 4 GN=GLOD4 PE=1 SV=1	F6TLX2_HUMAN	54,721.00	100.00%	1	1	11	0.00780%	12.50%
Tumor tissue	Glyoxalase domain-containing protein 4 GN=GLOD4 PE=1 SV=1	F6TLX2_HUMAN	54,721.00	100.00%	2	2	5	0.00383%	8.57%
Swab	Glyoxylate reductase/hydroxypyruvate reductase GN=GRHPR PE=1 SV=1	U3KQ56_HUMAN	38,696.10	100.00%	1	1	7	0.00496%	23.20%
Tumor tissue	Glyoxylate reductase/hydroxypyruvate reductase GN=GRHPR PE=1 SV=1	GRHPR_HUMAN	35,669.10	100.00%	1	1	7	0.00536%	26.20%
Tumor tissue	Glypican-1 GN=GPC1 PE=1 SV=2	sp|P35052|GPC1_HUMAN	61,681.30	100.00%	1	1	3	0.00230%	7.71%
Tumor tissue	Glypican-6 GN=GPC6 PE=1 SV=1	GPC6_HUMAN	62,736.40	100.00%	2	3	3	0.00230%	5.95%
Tumor tissue	GMP reductase 1 GN=GMPR PE=1 SV=1	GMPR1_HUMAN	37,418.70	100.00%	1	1	2	0.00153%	6.96%
Tumor tissue	Golgi autoantigen, golgin subfamily a, 2, isoform CRA_a GN=GOLGA2 PE=1 SV=1	sp|Q08379|GOGA2_HUMAN	111,658.30	100.00%	4	4	8	0.00612%	10.60%
Tumor tissue	Golgi pH regulator B GN=GPR89B PE=1 SV=1	sp|B7ZAQ6-3|GPHRA_HUMAN	49,989.30	100.00%	2	2	2	0.00153%	5.49%
Tumor tissue	Golgi resident protein GCP60 GN=ACBD3 PE=1 SV=4	GCP60_HUMAN	60,592.70	100.00%	6	8	9	0.00689%	19.10%

Biological sample	Protein name	Protein accession numbers	Protein molecular weight (Da)	Protein identification probability	Exclusive unique peptide count	Exclusive unique spectrum count	Total spectrum count	Percentage of total spectra	Percentage sequence coverage
Tumor tissue	Golgi-associated PDZ and coiled-coil motif containing protein transcript variant 3 GN=GOPC PE=1 SV=2	sp|Q9HD26|GOPC_HUMAN	44,790.90	100.00%	5	5	5	0.00383%	18.90%
Tumor tissue	Golgi-associated plant pathogenesis-related protein 1 GN=GLIPR2 PE=1 SV=3	GAPR1_HUMAN	17,218.20	100.00%	2	2	2	0.00153%	16.90%
Tumor tissue	Golgin subfamily A member 3 GN=GOLGA3 PE=1 SV=2	sp|Q08378|GOGA3_HUMAN	167,356.00	100.00%	10	11	11	0.00842%	10.30%
Tumor tissue	Golgin subfamily B member 1 GN=GOLGB1 PE=1 SV=2	sp|Q14789|GOGB1_HUMAN	376,017.80	100.00%	1	1	29	0.02220%	12.70%
Tumor tissue	GPI ethanolamine phosphate transferase 3 GN=PIGO PE=1 SV=3	sp|Q8TEQ8|PIGO_HUMAN	118,702.40	100.00%	3	3	3	0.00230%	3.67%
Tumor tissue	GPI-anchor transamidase GN=PIGK PE=1 SV=2	sp|Q92643|GPI8_HUMAN	45,252.80	100.00%	2	2	4	0.00306%	14.90%
Tumor tissue	GRAM domain-containing protein 1A GN=GRAMD1A PE=1 SV=1	M0QZ12_HUMAN	89,737.80	100.00%	2	2	2	0.00153%	3.47%
Pap test	Grancalcin GN=GCA PE=1 SV=2	GRAN_HUMAN	22,109.50	100.00%	3	3	5	0.00780%	14.70%
Swab	Grancalcin GN=GCA PE=1 SV=2	GRAN_HUMAN	22,109.50	100.00%	2	4	4	0.00283%	13.40%
Tumor tissue	Grancalcin GN=GCA PE=1 SV=2	GRAN_HUMAN	22,109.50	100.00%	3	4	4	0.00306%	18.90%
Pap test	Granulins GN=GRN PE=1 SV=2	sp|P28799|GRN_HUMAN	63,540.00	100.00%	1	1	7	0.01090%	12.00%
Tumor tissue	G-rich sequence factor 1 GN=GRSF1 PE=1 SV=3	sp|Q12849|GRSF1_HUMAN	53,127.00	100.00%	3	3	3	0.00230%	9.58%
Tumor tissue	GRIP1-associated protein 1 GN=GRIPAP1 PE=1 SV=2	sp|Q4V328|GRAP1_HUMAN	92,755.20	100.00%	5	5	6	0.00459%	12.20%
Pap test	Growth factor receptor-bound protein 2 GN=GRB2 PE=1 SV=1	sp|P62993|GRB2_HUMAN	25,206.80	100.00%	4	5	6	0.00936%	22.10%
Swab	Growth factor receptor-bound protein 2 GN=GRB2 PE=1 SV=1	sp|P62993|GRB2_HUMAN	25,206.80	100.00%	4	4	4	0.00283%	22.10%
Tumor tissue	Growth factor receptor-bound protein 2 GN=GRB2 PE=1 SV=1	sp|P62993|GRB2_HUMAN	25,206.80	100.00%	5	6	7	0.00536%	24.40%
Tumor tissue	GrpE protein homolog 1, mitochondrial GN=GRPEL1 PE=1 SV=2	GRPE1_HUMAN	24,279.20	100.00%	2	2	2	0.00153%	16.10%

Biological sample	Protein name	Protein accession numbers	Protein molecular weight (Da)	Protein identification probability	Exclusive unique peptide count	Exclusive unique spectrum count	Total spectrum count	Percentage of total spectra	Percentage sequence coverage
Tumor tissue	GTP:AMP phosphotransferase AK3, mitochondrial GN=AK3 PE=1 SV=4	sp|Q9UIJ7|KAD3_HUMAN	25,566.30	100.00%	6	6	6	0.00459%	29.50%
Tumor tissue	GTPase IMAP family member 1 GN=GIMAP1 PE=1 SV=1	GIMA1_HUMAN	34,368.50	100.00%	2	3	6	0.00459%	20.60%
Tumor tissue	GTPase IMAP family member 4 GN=GIMAP4 PE=1 SV=1	G5E9W9_HUMAN	39,034.50	100.00%	3	4	4	0.00306%	10.20%
Tumor tissue	GTPase NRas GN=NRAS PE=1 SV=1	RASN_HUMAN	21,229.30	100.00%	1	2	5	0.00383%	18.50%
Pap test	GTP-binding nuclear protein Ran (Fragment) GN=RAN PE=1 SV=1	J3KQE5_HUMAN	26,816.40	98.60%	1	1	2	0.00312%	8.12%
Swab	GTP-binding nuclear protein Ran (Fragment) GN=RAN PE=1 SV=1	J3KQE5_HUMAN	26,816.40	100.00%	1	1	5	0.00354%	19.20%
Tumor tissue	GTP-binding nuclear protein Ran (Fragment) GN=RAN PE=1 SV=1	J3KQE5_HUMAN	26,816.40	100.00%	1	1	25	0.01910%	31.20%
Tumor tissue	GTP-binding protein 1 GN=GTPBP1 PE=1 SV=3	GTPB1_HUMAN	72,454.50	100.00%	4	4	4	0.00306%	7.62%
Tumor tissue	GTP-binding protein SAR1a GN=SAR1A PE=1 SV=1	sp|Q9NR31|SAR1A_HUMAN	22,367.80	100.00%	3	3	11	0.00842%	33.80%
Tumor tissue	GTP-binding protein SAR1b GN=SAR1B PE=1 SV=1	SAR1B_HUMAN	22,411.00	100.00%	1	2	10	0.00766%	20.70%
Pap test	Guanine nucleotide-binding protein G(i) subunit alpha-2 GN=GNAI2 PE=1 SV=3	sp|P04899|GNAI2_HUMAN	40,452.00	100.00%	2	2	4	0.00624%	10.40%
Swab	Guanine nucleotide-binding protein G(i) subunit alpha-2 GN=GNAI2 PE=1 SV=3	sp|P04899|GNAI2_HUMAN	40,452.00	100.00%	3	3	8	0.00567%	18.90%
Tumor tissue	Guanine nucleotide-binding protein G(i) subunit alpha-2 GN=GNAI2 PE=1 SV=3	sp|P04899|GNAI2_HUMAN	40,452.00	100.00%	5	9	21	0.01610%	24.50%
Pap test	Guanine nucleotide-binding protein G(I)/G(S)/G(O) subunit gamma-12 GN=GNG12 PE=1 SV=3	GBG12_HUMAN	8,006.60	99.20%	1	1	2	0.00312%	22.20%
Swab	Guanine nucleotide-binding protein G(I)/G(S)/G(O) subunit gamma-12 GN=GNG12 PE=1 SV=3	GBG12_HUMAN	8,006.60	99.60%	1	1	1	0.00071%	22.20%

Biological sample	Protein name	Protein accession numbers	Protein molecular weight (Da)	Protein identification probability	Exclusive unique peptide count	Exclusive unique spectrum count	Total spectrum count	Percentage of total spectra	Percentage sequence coverage
Tumor tissue	Guanine nucleotide-binding protein G(I)/G(S)/G(O) subunit gamma-12 GN=GNG12 PE=1 SV=3	GBG12_HUMAN	8,006.60	100.00%	2	2	6	0.00459%	37.50%
Pap test	Guanine nucleotide-binding protein G(I)/G(S)/G(T) subunit beta-2 GN=GNB2 PE=1 SV=3	sp|P62879|GBB2_HUMAN	37,331.80	100.00%	1	1	5	0.00780%	12.60%
Swab	Guanine nucleotide-binding protein G(k) subunit alpha GN=GNAI3 PE=1 SV=3	GNAI3_HUMAN	40,533.50	99.70%	1	1	5	0.00354%	7.91%
Tumor tissue	Guanine nucleotide-binding protein G(k) subunit alpha GN=GNAI3 PE=1 SV=3	GNAI3_HUMAN	40,533.50	100.00%	3	3	9	0.00689%	16.10%
Tumor tissue	Guanine nucleotide-binding protein G(q) subunit alpha GN=GNAQ PE=1 SV=4	GNAQ_HUMAN	42,143.60	100.00%	2	2	5	0.00383%	11.70%
Tumor tissue	Guanine nucleotide-binding protein G(s) subunit alpha isoforms XLas GN=GNAS PE=1 SV=2	sp|Q5JWF2|GNAS1_HUMAN	111,026.30	100.00%	1	1	17	0.01300%	10.90%
Tumor tissue	Guanine nucleotide-binding protein subunit alpha-11 GN=GNA11 PE=1 SV=2	GNA11_HUMAN	42,125.10	100.00%	2	2	5	0.00383%	13.10%
Tumor tissue	Guanine nucleotide-binding protein subunit alpha-13 GN=GNA13 PE=1 SV=2	sp|Q14344|GNA13_HUMAN	44,051.00	100.00%	6	7	13	0.00995%	28.40%
Tumor tissue	Guanine nucleotide-binding protein subunit beta-4 GN=GNB4 PE=1 SV=3	GBB4_HUMAN	37,567.80	99.80%	1	1	6	0.00459%	12.40%
Tumor tissue	Guanine nucleotide-binding protein-like 1 GN=GNL1 PE=1 SV=2	sp|P36915|GNL1_HUMAN	68,660.80	100.00%	2	3	7	0.00536%	7.91%
Tumor tissue	Guanylate kinase GN=GUK1 PE=1 SV=2	sp|Q16774|KGUA_HUMAN	25,336.10	100.00%	1	2	5	0.00383%	21.70%
Pap test	Guanylate-binding protein 1 GN=GBP1 PE=1 SV=2	GBP1_HUMAN	67,932.50	99.90%	1	1	5	0.00780%	4.90%
Tumor tissue	Guanylate-binding protein 1 GN=GBP1 PE=1 SV=2	GBP1_HUMAN	67,932.50	100.00%	11	18	31	0.02370%	23.80%
Pap test	Guanylate-binding protein 2 GN=GBP2 PE=1 SV=3	GBP2_HUMAN	67,211.10	99.80%	1	1	5	0.00780%	5.58%

Biological sample	Protein name	Protein accession numbers	Protein molecular weight (Da)	Protein identification probability	Exclusive unique peptide count	Exclusive unique spectrum count	Total spectrum count	Percentage of total spectra	Percentage sequence coverage
Swab	Guanylate-binding protein 2 GN=GBP2 PE=1 SV=3	GBP2_HUMAN	67,211.10	99.90%	1	1	3	0.00213%	4.91%
Tumor tissue	Guanylate-binding protein 2 GN=GBP2 PE=1 SV=3	GBP2_HUMAN	67,211.10	100.00%	6	7	18	0.01380%	16.10%
Tumor tissue	Guanylate-binding protein 5 GN=GBP5 PE=1 SV=1	sp|Q96PP8|GBP5_HUMAN	66,617.90	100.00%	3	3	5	0.00383%	6.66%
Pap test	Guanylate-binding protein 6 GN=GBP6 PE=2 SV=1	sp|Q6ZN66|GBP6_HUMAN	72,427.70	100.00%	2	2	2	0.00312%	5.37%
Tumor tissue	H/ACA ribonucleoprotein complex subunit 2 (Fragment) GN=NHP2 PE=1 SV=1	NHP2_HUMAN	15,017.60	100.00%	2	3	3	0.00230%	22.00%
Tumor tissue	H/ACA ribonucleoprotein complex subunit 4 GN=DKC1 PE=1 SV=3	sp|O60832|DKC1_HUMAN	57,675.20	100.00%	2	2	11	0.00842%	19.50%
Swab	Haloacid dehalogenase-like hydrolase domain-containing protein 2 (Fragment) GN=HDHD2 PE=1 SV=1	sp|Q9H0R4|HDHD2_HUMAN	22,491.00	100.00%	3	3	4	0.00283%	14.70%
Tumor tissue	Haloacid dehalogenase-like hydrolase domain-containing protein 2 (Fragment) GN=HDHD2 PE=1 SV=1	sp|Q9H0R4|HDHD2_HUMAN	22,491.00	100.00%	3	4	4	0.00306%	21.10%
Tumor tissue	Haloacid dehalogenase-like hydrolase domain-containing protein 3 GN=HDHD3 PE=1 SV=1	HDHD3_HUMAN	28,000.10	100.00%	3	4	4	0.00306%	19.10%
Pap test	Haptoglobin GN=HP PE=1 SV=1	sp|P00738|HPT_HUMAN	45,204.90	100.00%	2	4	213	0.33200%	56.40%
Swab	Haptoglobin GN=HP PE=1 SV=1	sp|P00738|HPT_HUMAN	45,204.90	100.00%	2	7	190	0.13500%	47.80%
Tumor tissue	HCG2002594, isoform CRA_c GN=SEPT5 PE=1 SV=1	G3XAH0_HUMAN	43,845.70	100.00%	2	2	2	0.00153%	5.82%
Swab	HCG2041210 (Fragment) GN=IGLV3-12 PE=1 SV=1	A0A075B6K2_HUMAN	12,584.80	99.80%	1	1	8	0.00567%	23.10%
Pap test	HCG2043238 (Fragment) GN=IGLV3-10 PE=1 SV=1	A0A075B6K4_HUMAN	12,629.20	100.00%	2	3	8	0.01250%	27.40%
Swab	HCG2043238 (Fragment) GN=IGLV3-10 PE=1 SV=1	A0A075B6K4_HUMAN	12,629.20	100.00%	1	2	12	0.00850%	18.80%
Pap test	HCG2043239 (Fragment) GN=IGLV3-9 PE=1 SV=1	A0A075B6K5_HUMAN	12,331.70	99.90%	1	2	38	0.05930%	33.00%

Biological sample	Protein name	Protein accession numbers	Protein molecular weight (Da)	Protein identification probability	Exclusive unique peptide count	Exclusive unique spectrum count	Total spectrum count	Percentage of total spectra	Percentage sequence coverage
Swab	HCG2043239 (Fragment) GN=IGLV3-9 PE=1 SV=1	A0A075B6K5_HUMAN	12,331.70	99.90%	1	1	34	0.02410%	33.00%
Tumor tissue	HCG2043275 GN=EEF1E1-BLOC1S5 PE=4 SV=2	sp|O43324|MCA3_HUMAN	17,018.60	100.00%	3	4	5	0.00383%	30.50%
Pap test	HCG2044781 GN=TMEM189-UBE2V1 PE=4 SV=1	sp|Q13404|UB2V1_HUMAN	42,209.00	100.00%	2	2	8	0.01250%	17.80%
Swab	HCG2044781 GN=TMEM189-UBE2V1 PE=4 SV=1	sp|Q13404|UB2V1_HUMAN	42,209.00	99.90%	1	1	6	0.00425%	10.80%
Tumor tissue	HCG2044781 GN=TMEM189-UBE2V1 PE=4 SV=1	sp|Q13404|UB2V1_HUMAN	42,209.00	100.00%	2	2	9	0.00689%	14.30%
Tumor tissue	HCLS1-binding protein 3 GN=HS1BP3 PE=1 SV=1	H1BP3_HUMAN	42,780.90	100.00%	3	3	3	0.00230%	20.20%
Tumor tissue	HEAT repeat-containing protein 1 GN=HEATR1 PE=1 SV=3	HEAT1_HUMAN	233,284.70	100.00%	5	6	6	0.00459%	3.36%
Tumor tissue	HEAT repeat-containing protein 3 GN=HEATR3 PE=1 SV=2	sp|Q7Z4Q2|HEAT3_HUMAN	74,584.90	100.00%	1	1	2	0.00153%	3.82%
Tumor tissue	HEAT repeat-containing protein 5A GN=HEATR5A PE=1 SV=1	sp|Q86XA9|HTR5A_HUMAN	222,709.00	100.00%	2	2	2	0.00153%	1.56%
Tumor tissue	Heat shock 70 kDa protein 12A GN=HSPA12A PE=1 SV=1	HS12A_HUMAN	77,020.80	100.00%	9	9	11	0.00842%	17.60%
Tumor tissue	Heat shock 70 kDa protein 13 GN=HSPA13 PE=1 SV=1	HSP13_HUMAN	51,928.50	100.00%	4	4	4	0.00306%	10.60%
Tumor tissue	Heat shock 70 kDa protein 14 GN=HSPA14 PE=1 SV=1	HSP7E_HUMAN	54,795.50	100.00%	5	5	5	0.00383%	14.10%
Pap test	Heat shock 70 kDa protein 1B GN=HSPA1B PE=1 SV=1	HS71B_HUMAN	70,111.10	100.00%	8	11	43	0.06710%	32.70%
Swab	Heat shock 70 kDa protein 1B GN=HSPA1B PE=1 SV=1	HS71B_HUMAN	70,111.10	100.00%	9	13	63	0.04460%	34.60%
Tumor tissue	Heat shock 70 kDa protein 1B GN=HSPA1B PE=1 SV=1	HS71B_HUMAN	70,111.10	100.00%	11	20	66	0.05050%	35.20%
Pap test	Heat shock 70 kDa protein 4 GN=HSPA4 PE=1 SV=4	sp|P34932|HSP74_HUMAN	94,331.90	100.00%	8	9	9	0.01400%	12.40%
Swab	Heat shock 70 kDa protein 4 GN=HSPA4 PE=1 SV=4	sp|P34932|HSP74_HUMAN	94,331.90	100.00%	12	12	19	0.01350%	24.90%
Tumor tissue	Heat shock 70 kDa protein 4 GN=HSPA4 PE=1 SV=4	sp|P34932|HSP74_HUMAN	94,331.90	100.00%	17	24	31	0.02370%	31.90%

Biological sample	Protein name	Protein accession numbers	Protein molecular weight (Da)	Protein identification probability	Exclusive unique peptide count	Exclusive unique spectrum count	Total spectrum count	Percentage of total spectra	Percentage sequence coverage
Swab	Heat shock 70 kDa protein 4L GN=HSPA4L PE=1 SV=1	HS74L_HUMAN	91,955.90	99.60%	1	1	4	0.00283%	4.43%
Pap test	Heat shock cognate 71 kDa protein GN=HSPA8 PE=1 SV=1	sp|P11142|HSP7C_HUMAN	70,899.80	100.00%	2	3	50	0.07800%	33.70%
Swab	Heat shock cognate 71 kDa protein GN=HSPA8 PE=1 SV=1	sp|P11142|HSP7C_HUMAN	70,899.80	100.00%	2	4	80	0.05670%	37.60%
Tumor tissue	Heat shock cognate 71 kDa protein GN=HSPA8 PE=1 SV=1	sp|P11142|HSP7C_HUMAN	70,899.80	100.00%	2	5	133	0.10200%	46.00%
Tumor tissue	Heat shock factor-binding protein 1 GN=HSBP1 PE=1 SV=1	HSBP1_HUMAN	8,543.70	100.00%	2	2	2	0.00153%	47.40%
Pap test	Heat shock protein beta-1 GN=HSPB1 PE=1 SV=2	HSPB1_HUMAN	22,782.60	100.00%	8	15	88	0.13700%	63.40%
Swab	Heat shock protein beta-1 GN=HSPB1 PE=1 SV=2	HSPB1_HUMAN	22,782.60	100.00%	5	7	36	0.02550%	59.50%
Tumor tissue	Heat shock protein beta-1 GN=HSPB1 PE=1 SV=2	HSPB1_HUMAN	22,782.60	100.00%	6	12	50	0.03830%	49.80%
Tumor tissue	Heat shock protein beta-6 GN=HSPB6 PE=1 SV=2	HSPB6_HUMAN	17,135.20	100.00%	4	4	4	0.00306%	29.40%
Pap test	Heat shock protein HSP 90-beta GN=HSP90AB1 PE=1 SV=4	HS90B_HUMAN	83,267.30	100.00%	1	1	17	0.02650%	16.60%
Swab	Heat shock protein HSP 90-beta GN=HSP90AB1 PE=1 SV=4	HS90B_HUMAN	83,267.30	100.00%	5	5	49	0.03470%	35.60%
Tumor tissue	Heat shock protein HSP 90-beta GN=HSP90AB1 PE=1 SV=4	HS90B_HUMAN	83,267.30	100.00%	7	14	109	0.08340%	37.30%
Tumor tissue	Heat shock-related 70 kDa protein 2 GN=HSPA2 PE=1 SV=1	HSP72_HUMAN	70,023.00	100.00%	8	10	56	0.04290%	31.30%
Tumor tissue	Helicase SKI2W GN=SKIV2L PE=1 SV=3	SKIV2_HUMAN	137,757.50	100.00%	6	6	7	0.00536%	6.90%
Tumor tissue	Hematological and neurological-expressed 1 protein GN=HN1 PE=1 SV=1	sp|Q9UK76|HN1_HUMAN	11,012.20	100.00%	3	4	4	0.00306%	37.50%
Tumor tissue	Hematopoietic lineage cell-specific protein GN=HCLS1 PE=1 SV=3	sp|P14317|HCLS1_HUMAN	54,013.20	100.00%	6	7	13	0.00995%	23.90%
Tumor tissue	Heme oxygenase 2 GN=HMOX2 PE=1 SV=1	sp|P30519|HMOX2_HUMAN	41,669.80	100.00%	2	3	4	0.00306%	13.20%

Biological sample	Protein name	Protein accession numbers	Protein molecular weight (Da)	Protein identification probability	Exclusive unique peptide count	Exclusive unique spectrum count	Total spectrum count	Percentage of total spectra	Percentage sequence coverage
Swab	Heme-binding protein 1 GN=HEBP1 PE=1 SV=1	HEBP1_HUMAN	21,097.40	100.00%	1	1	1	0.00071%	8.47%
Tumor tissue	Heme-binding protein 1 GN=HEBP1 PE=1 SV=1	HEBP1_HUMAN	21,097.40	100.00%	3	4	5	0.00383%	28.60%
Pap test	Heme-binding protein 2 GN=HEBP2 PE=1 SV=1	sp|Q9Y5Z4|HEBP2_HUMAN	22,875.70	100.00%	6	10	35	0.05460%	39.00%
Swab	Heme-binding protein 2 GN=HEBP2 PE=1 SV=1	sp|Q9Y5Z4|HEBP2_HUMAN	22,875.70	100.00%	6	7	16	0.01130%	32.70%
Tumor tissue	Heme-binding protein 2 GN=HEBP2 PE=1 SV=1	sp|Q9Y5Z4|HEBP2_HUMAN	22,875.70	100.00%	3	4	5	0.00383%	19.50%
Pap test	Hemoglobin subunit alpha GN=HBA1 PE=1 SV=2	HBA_HUMAN	15,257.60	100.00%	7	24	176	0.27500%	64.10%
Swab	Hemoglobin subunit alpha GN=HBA1 PE=1 SV=2	HBA_HUMAN	15,257.60	100.00%	5	11	88	0.06240%	54.90%
Tumor tissue	Hemoglobin subunit alpha GN=HBA1 PE=1 SV=2	HBA_HUMAN	15,257.60	100.00%	9	58	636	0.48700%	64.10%
Pap test	Hemoglobin subunit beta GN=HBB PE=1 SV=2	HBB_HUMAN	15,998.00	100.00%	5	11	212	0.33100%	77.60%
Swab	Hemoglobin subunit beta GN=HBB PE=1 SV=2	HBB_HUMAN	15,998.00	100.00%	6	13	143	0.10100%	77.60%
Tumor tissue	Hemoglobin subunit beta GN=HBB PE=1 SV=2	HBB_HUMAN	15,998.00	100.00%	4	11	363	0.27800%	70.10%
Pap test	Hemoglobin subunit delta GN=HBD PE=1 SV=2	HBD_HUMAN	16,055.20	100.00%	6	11	110	0.17200%	85.70%
Swab	Hemoglobin subunit delta GN=HBD PE=1 SV=2	HBD_HUMAN	16,055.20	100.00%	3	4	68	0.04820%	56.50%
Tumor tissue	Hemoglobin subunit delta GN=HBD PE=1 SV=2	HBD_HUMAN	16,055.20	100.00%	4	9	221	0.16900%	70.10%
Tumor tissue	Hemoglobin subunit epsilon GN=HBE1 PE=1 SV=2	HBE_HUMAN	16,203.00	99.90%	1	1	31	0.02370%	17.70%
Tumor tissue	Hemoglobin subunit gamma-1 GN=HBG1 PE=1 SV=2	HBG1_HUMAN	16,140.50	100.00%	2	2	32	0.02450%	26.50%
Pap test	Hemopexin GN=HPX PE=1 SV=2	HEMO_HUMAN	51,676.50	100.00%	18	40	231	0.36000%	51.30%
Swab	Hemopexin GN=HPX PE=1 SV=2	HEMO_HUMAN	51,676.50	100.00%	15	27	242	0.17100%	43.90%
Tumor tissue	Hemopexin GN=HPX PE=1 SV=2	HEMO_HUMAN	51,676.50	100.00%	6	6	8	0.00612%	14.90%

Biological sample	Protein name	Protein accession numbers	Protein molecular weight (Da)	Protein identification probability	Exclusive unique peptide count	Exclusive unique spectrum count	Total spectrum count	Percentage of total spectra	Percentage sequence coverage
Pap test	Heparin cofactor 2 GN=SERPIND1 PE=1 SV=3	HEP2_HUMAN	57,072.90	100.00%	2	3	4	0.00624%	4.01%
Swab	Heparin cofactor 2 GN=SERPIND1 PE=1 SV=3	HEP2_HUMAN	57,072.90	100.00%	3	3	3	0.00213%	5.41%
Tumor tissue	Heparin cofactor 2 GN=SERPIND1 PE=1 SV=3	HEP2_HUMAN	57,072.90	100.00%	3	4	5	0.00383%	6.41%
Swab	Hepatocyte growth factor-regulated tyrosine kinase substrate GN=HGS PE=1 SV=1	sp|O14964|HGS_HUMAN	86,191.60	99.90%	1	1	1	0.00071%	1.54%
Tumor tissue	Hepatocyte growth factor-regulated tyrosine kinase substrate GN=HGS PE=1 SV=1	sp|O14964|HGS_HUMAN	86,191.60	100.00%	3	4	4	0.00306%	4.12%
Pap test	Hepatoma-derived growth factor GN=HDGF PE=1 SV=1	sp|P51858|HDGF_HUMAN	26,788.60	100.00%	2	2	11	0.01720%	25.00%
Tumor tissue	Hepatoma-derived growth factor GN=HDGF PE=1 SV=1	sp|P51858|HDGF_HUMAN	26,788.60	100.00%	1	1	25	0.01910%	47.10%
Tumor tissue	Hepatoma-derived growth factor, related protein 3, isoform CRA_a GN=HDGFRP3 PE=1 SV=1	A0A024R216_HUMAN	22,620.50	100.00%	1	1	2	0.00153%	16.30%
Tumor tissue	Hephaestin GN=HEPH PE=1 SV=1	sp|Q9BQS7|HEPH_HUMAN	130,767.70	100.00%	2	2	2	0.00153%	2.24%
Tumor tissue	Hermansky-Pudlak syndrome 5 protein GN=HPS5 PE=1 SV=2	sp|Q9UPZ3|HPS5_HUMAN	127,453.30	100.00%	2	2	2	0.00153%	2.39%
Tumor tissue	Heterochromatin protein 1-binding protein 3 GN=HP1BP3 PE=1 SV=1	sp|Q5SSJ5|HP1B3_HUMAN	61,208.70	100.00%	14	16	19	0.01450%	25.50%
Tumor tissue	Heterogeneous nuclear ribonucleoprotein A0 GN=HNRNPA0 PE=1 SV=1	ROA0_HUMAN	30,840.50	100.00%	5	6	11	0.00842%	26.20%
Swab	Heterogeneous nuclear ribonucleoprotein A1 GN=HNRNPA1 PE=1 SV=2	sp|P09651|ROA1_HUMAN	33,155.40	100.00%	1	2	9	0.00638%	23.80%
Tumor tissue	Heterogeneous nuclear ribonucleoprotein A1 GN=HNRNPA1 PE=1 SV=2	sp|P09651|ROA1_HUMAN	33,155.40	100.00%	4	7	41	0.03140%	46.60%

Biological sample	Protein name	Protein accession numbers	Protein molecular weight (Da)	Protein identification probability	Exclusive unique peptide count	Exclusive unique spectrum count	Total spectrum count	Percentage of total spectra	Percentage sequence coverage
Tumor tissue	Heterogeneous nuclear ribonucleoprotein A3 GN=HNRNPA3 PE=1 SV=2	sp|P51991|ROA3_HUMAN	39,595.10	100.00%	2	2	30	0.02300%	35.20%
Swab	Heterogeneous nuclear ribonucleoprotein D0 (Fragment) GN=HNRNPD PE=1 SV=8	H0Y8G5_HUMAN	29,667.70	100.00%	5	5	8	0.00567%	24.60%
Tumor tissue	Heterogeneous nuclear ribonucleoprotein D0 (Fragment) GN=HNRNPD PE=1 SV=8	H0Y8G5_HUMAN	29,667.70	100.00%	8	13	25	0.01910%	29.60%
Tumor tissue	Heterogeneous nuclear ribonucleoprotein D-like GN=HNRNPDL PE=1 SV=1	sp|O14979|HNRDL_HUMAN	40,041.00	100.00%	4	6	12	0.00919%	12.40%
Pap test	Heterogeneous nuclear ribonucleoprotein F GN=HNRNPF PE=1 SV=3	HNRPF_HUMAN	45,671.90	100.00%	1	1	6	0.00936%	10.60%
Swab	Heterogeneous nuclear ribonucleoprotein F GN=HNRNPF PE=1 SV=3	HNRPF_HUMAN	45,671.90	100.00%	2	2	4	0.00283%	10.40%
Tumor tissue	Heterogeneous nuclear ribonucleoprotein F GN=HNRNPF PE=1 SV=3	HNRPF_HUMAN	45,671.90	100.00%	4	7	32	0.02450%	23.40%
Tumor tissue	Heterogeneous nuclear ribonucleoprotein H GN=HNRNPH1 PE=1 SV=1	G8JLB6_HUMAN	51,230.10	100.00%	1	2	42	0.03220%	30.70%
Swab	Heterogeneous nuclear ribonucleoprotein H2 GN=HNRNPH2 PE=1 SV=1	HNRH2_HUMAN	49,264.10	100.00%	1	1	3	0.00213%	10.00%
Tumor tissue	Heterogeneous nuclear ribonucleoprotein H2 GN=HNRNPH2 PE=1 SV=1	HNRH2_HUMAN	49,264.10	100.00%	5	9	20	0.01530%	26.50%
Tumor tissue	Heterogeneous nuclear ribonucleoprotein L GN=HNRNPL PE=1 SV=2	sp|P14866|HNRPL_HUMAN	64,132.80	100.00%	12	28	45	0.03440%	38.50%
Pap test	Heterogeneous nuclear ribonucleoprotein M GN=HNRNPM PE=1 SV=1	sp|P52272|HNRPM_HUMAN	77,571.30	100.00%	1	1	4	0.00624%	5.34%

Biological sample	Protein name	Protein accession numbers	Protein molecular weight (Da)	Protein identification probability	Exclusive unique peptide count	Exclusive unique spectrum count	Total spectrum count	Percentage of total spectra	Percentage sequence coverage
Tumor tissue	Heterogeneous nuclear ribonucleoprotein M GN=HNRNPM PE=1 SV=1	sp|P52272|HNRPM_HUMAN	77,571.30	100.00%	4	5	59	0.04520%	42.90%
Tumor tissue	Heterogeneous nuclear ribonucleoprotein M (Fragment) GN=HNRNPM PE=1 SV=1	M0QZM1_HUMAN	40,045.50	100.00%	1	1	35	0.02680%	45.40%
Tumor tissue	Heterogeneous nuclear ribonucleoprotein R GN=HNRNPR PE=1 SV=1	sp|O43390|HNRPR_HUMAN	70,943.90	100.00%	1	2	26	0.01990%	23.70%
Tumor tissue	Heterogeneous nuclear ribonucleoprotein U GN=HNRNPU PE=1 SV=6	sp|Q00839|HNRPU_HUMAN	90,585.20	100.00%	2	4	39	0.02990%	18.40%
Pap test	Heterogeneous nuclear ribonucleoprotein U-like protein 1 GN=HNRNPUL1 PE=1 SV=1	B7Z4B8_HUMAN	86,123.50	100.00%	4	4	4	0.00624%	6.26%
Tumor tissue	Heterogeneous nuclear ribonucleoprotein U-like protein 1 GN=HNRNPUL1 PE=1 SV=1	B7Z4B8_HUMAN	86,123.50	100.00%	9	12	15	0.01150%	17.90%
Tumor tissue	Heterogeneous nuclear ribonucleoprotein U-like protein 2 GN=HNRNPUL2 PE=1 SV=1	HNRL2_HUMAN	85,105.20	100.00%	8	9	10	0.00766%	11.10%
Pap test	Heterogeneous nuclear ribonucleoproteins A2/B1 GN=HNRNPA2B1 PE=1 SV=2	sp|P22626|ROA2_HUMAN	37,430.30	100.00%	3	3	4	0.00624%	11.90%
Swab	Heterogeneous nuclear ribonucleoproteins A2/B1 GN=HNRNPA2B1 PE=1 SV=2	sp|P22626|ROA2_HUMAN	37,430.30	100.00%	6	6	7	0.00496%	20.40%
Tumor tissue	Heterogeneous nuclear ribonucleoproteins A2/B1 GN=HNRNPA2B1 PE=1 SV=2	sp|P22626|ROA2_HUMAN	37,430.30	100.00%	13	25	58	0.04440%	45.60%
Pap test	Heterogeneous nuclear ribonucleoproteins C1/C2 GN=HNRNPC PE=1 SV=1	sp|P07910-2|HNRPC_HUMAN	32,338.00	100.00%	1	1	4	0.00624%	11.30%
Tumor tissue	Heterogeneous nuclear ribonucleoproteins C1/C2 GN=HNRNPC PE=1 SV=1	sp|P07910-2|HNRPC_HUMAN	32,239.00	100.00%	2	5	31	0.02370%	24.00%

Biological sample	Protein name	Protein accession numbers	Protein molecular weight (Da)	Protein identification probability	Exclusive unique peptide count	Exclusive unique spectrum count	Total spectrum count	Percentage of total spectra	Percentage sequence coverage
Swab	Hexokinase-2 GN=HK2 PE=1 SV=2	HXK2_HUMAN	102,381.30	100.00%	5	6	8	0.00567%	10.50%
Tumor tissue	Hexokinase-2 GN=HK2 PE=1 SV=2	HXK2_HUMAN	102,381.30	100.00%	5	5	12	0.00919%	12.40%
Pap test	Hexokinase-3 GN=HK3 PE=1 SV=2	HXK3_HUMAN	99,023.80	99.20%	1	1	1	0.00156%	1.08%
Swab	Hexokinase-3 GN=HK3 PE=1 SV=2	HXK3_HUMAN	99,023.80	100.00%	14	16	34	0.02410%	20.30%
Tumor tissue	Hexokinase-3 GN=HK3 PE=1 SV=2	HXK3_HUMAN	99,023.80	100.00%	3	3	8	0.00612%	9.21%
Tumor tissue	High affinity immunoglobulin epsilon receptor subunit gamma GN=FCER1G PE=1 SV=1	FCERG_HUMAN	9,668.00	100.00%	2	2	2	0.00153%	23.30%
Tumor tissue	High density lipoprotein binding protein (Vigilin), isoform CRA_a GN=HDLBP PE=1 SV=1	A0A024R4E5_HUMAN	141,442.20	100.00%	2	2	59	0.04520%	33.00%
Pap test	High mobility group protein B1 GN=HMGB1 PE=1 SV=3	HMGB1_HUMAN	24,894.70	99.90%	1	1	4	0.00624%	14.40%
Tumor tissue	High mobility group protein B1 GN=HMGB1 PE=1 SV=3	HMGB1_HUMAN	24,894.70	100.00%	3	8	20	0.01530%	31.20%
Pap test	High mobility group protein B2 GN=HMGB2 PE=1 SV=2	HMGB2_HUMAN	24,034.60	100.00%	2	2	2	0.00312%	11.50%
Swab	High mobility group protein B2 GN=HMGB2 PE=1 SV=2	HMGB2_HUMAN	24,034.60	100.00%	2	3	3	0.00213%	11.50%
Tumor tissue	High mobility group protein B2 GN=HMGB2 PE=1 SV=2	HMGB2_HUMAN	24,034.60	100.00%	4	9	10	0.00766%	23.90%
Tumor tissue	High mobility group protein B3 (Fragment) GN=HMGB3 PE=1 SV=1	HMGB3_HUMAN	22,091.00	100.00%	5	6	7	0.00536%	21.20%
Tumor tissue	Histidine triad nucleotide-binding protein 1 GN=HINT1 PE=1 SV=2	HINT1_HUMAN	13,802.10	100.00%	3	8	12	0.00919%	50.00%
Swab	Histidine triad nucleotide-binding protein 2, mitochondrial GN=HINT2 PE=1 SV=1	HINT2_HUMAN	17,161.70	100.00%	1	1	1	0.00071%	10.40%
Tumor tissue	Histidine triad nucleotide-binding protein 2, mitochondrial GN=HINT2 PE=1 SV=1	HINT2_HUMAN	17,161.70	100.00%	2	2	2	0.00153%	17.80%
Pap test	Histidine-rich glycoprotein GN=HRG PE=1 SV=1	HRG_HUMAN	59,576.60	100.00%	12	16	41	0.06400%	25.50%

Biological sample	Protein name	Protein accession numbers	Protein molecular weight (Da)	Protein identification probability	Exclusive unique peptide count	Exclusive unique spectrum count	Total spectrum count	Percentage of total spectra	Percentage sequence coverage
Swab	Histidine-rich glycoprotein GN=HRG PE=1 SV=1	HRG_HUMAN	59,576.60	100.00%	9	12	28	0.01980%	22.10%
Tumor tissue	Histidine-rich glycoprotein GN=HRG PE=1 SV=1	HRG_HUMAN	59,576.60	100.00%	9	15	20	0.01530%	23.20%
Tumor tissue	Histone deacetylase 1 GN=HDAC1 PE=1 SV=1	HDAC1_HUMAN	55,104.10	100.00%	1	1	6	0.00459%	11.20%
Tumor tissue	Histone deacetylase complex subunit SAP18 GN=SAP18 PE=1 SV=1	X6RAL5_HUMAN	19,526.90	100.00%	4	5	5	0.00383%	27.90%
Tumor tissue	Histone H1.1 GN=HIST1H1A PE=1 SV=3	H11_HUMAN	21,843.20	100.00%	3	5	19	0.01450%	26.50%
Tumor tissue	Histone H1.2 GN=HIST1H1C PE=1 SV=2	H12_HUMAN	21,365.80	100.00%	1	1	39	0.02990%	26.30%
Tumor tissue	Histone H1.3 GN=HIST1H1D PE=1 SV=2	H13_HUMAN	22,351.30	100.00%	1	1	21	0.01610%	25.30%
Pap test	Histone H1.5 GN=HIST1H1B PE=1 SV=3	H15_HUMAN	22,581.70	100.00%	3	3	5	0.00780%	18.10%
Tumor tissue	Histone H1.5 GN=HIST1H1B PE=1 SV=3	H15_HUMAN	22,581.70	100.00%	7	11	20	0.01530%	21.70%
Tumor tissue	Histone H1x GN=H1FX PE=1 SV=1	H1X_HUMAN	22,487.90	100.00%	6	7	9	0.00689%	26.30%
Tumor tissue	Histone H2A type 1-B/E GN=HIST1H2AB PE=1 SV=2	H2A1B_HUMAN	14,106.00	99.90%	1	2	52	0.03980%	57.70%
Tumor tissue	Histone H2A type 2-A GN=HIST2H2AA3 PE=1 SV=3	H2A2A_HUMAN	14,096.00	100.00%	3	6	50	0.03830%	60.00%
Tumor tissue	Histone H2AX GN=H2AFX PE=1 SV=2	H2AX_HUMAN	15,145.10	99.80%	1	1	25	0.01910%	58.00%
Pap test	Histone H3 GN=HIST2H3PS2 PE=1 SV=1	Q5TEC6_HUMAN	15,430.70	98.90%	1	1	4	0.00624%	14.70%
Tumor tissue	Histone H3.2 GN=HIST2H3A PE=1 SV=3	H32_HUMAN	15,388.70	99.90%	1	1	13	0.00995%	33.80%
Pap test	Histone H4 GN=HIST1H4A PE=1 SV=2	H4_HUMAN	11,367.70	100.00%	6	10	40	0.06240%	42.70%
Swab	Histone H4 GN=HIST1H4A PE=1 SV=2	H4_HUMAN	11,367.70	100.00%	8	13	51	0.03610%	53.40%

Biological sample	Protein name	Protein accession numbers	Protein molecular weight (Da)	Protein identification probability	Exclusive unique peptide count	Exclusive unique spectrum count	Total spectrum count	Percentage of total spectra	Percentage sequence coverage
Tumor tissue	Histone H4 GN=HIST1H4A PE=1 SV=2	H4_HUMAN	11,367.70	100.00%	10	23	131	0.10000%	57.30%
Tumor tissue	Histone PARylation factor 1 GN=HPF1 PE=1 SV=2	HPF1_HUMAN	39,437.50	100.00%	2	3	3	0.00230%	8.09%
Tumor tissue	Histone-binding protein RBBP7 GN=RBBP7 PE=1 SV=1	sp|Q16576|RBBP7_HUMAN	46,938.10	100.00%	2	3	7	0.00536%	19.70%
Tumor tissue	Histone-lysine N-methyltransferase setd3 GN=SETD3 PE=1 SV=1	sp|Q86TU7|SETD3_HUMAN	67,259.00	100.00%	2	2	2	0.00153%	4.21%
Tumor tissue	Histone-lysine N-methyltransferase SETD7 GN=SETD7 PE=1 SV=1	SETD7_HUMAN	40,720.70	100.00%	2	2	2	0.00153%	10.70%
Tumor tissue	HIV Tat-specific factor 1 GN=HTATSF1 PE=1 SV=1	HTSF1_HUMAN	85,854.60	100.00%	6	8	8	0.00612%	11.50%
Tumor tissue	HLA class I histocompatibility antigen, A-2 alpha chain GN=HLA-A PE=1 SV=1	1A02_HUMAN	34,287.70	100.00%	4	6	38	0.02910%	35.90%
Tumor tissue	HLA class I histocompatibility antigen, alpha chain E GN=HLA-E PE=1 SV=3	HLAE_HUMAN	40,156.30	99.70%	1	1	2	0.00153%	12.30%
Tumor tissue	HLA class I histocompatibility antigen, alpha chain F GN=HLA-F PE=1 SV=1	sp|P30511|HLAF_HUMAN	50,427.70	100.00%	2	2	8	0.00612%	14.50%
Tumor tissue	HLA class I histocompatibility antigen, B-46 alpha chain (Fragment) GN=HLA-B PE=1 SV=1	A0A140T997_HUMAN	27,767.10	100.00%	2	5	22	0.01680%	30.50%
Tumor tissue	HLA class I histocompatibility antigen, Cw-6 alpha chain GN=HLA-C PE=1 SV=1	A0A140T912_HUMAN	44,432.60	100.00%	1	3	27	0.02070%	37.40%
Tumor tissue	HLA class II histocompatibility antigen, DP beta 1 chain GN=HLA-DPB1 PE=1 SV=1	A0A140T9T6_HUMAN	25,992.00	100.00%	2	3	5	0.00383%	14.50%
Tumor tissue	HLA class II histocompatibility antigen, DQ beta 1 chain GN=HLA-DQB1 PE=1 SV=1	A0A140T9S8_HUMAN	30,376.90	100.00%	2	4	7	0.00536%	15.60%
Tumor tissue	HLA class II histocompatibility antigen, DR beta 4 chain GN=HLA-DRB4 PE=1 SV=1	DRB4_HUMAN	29,940.60	100.00%	2	2	2	0.00153%	8.65%

Biological sample	Protein name	Protein accession numbers	Protein molecular weight (Da)	Protein identification probability	Exclusive unique peptide count	Exclusive unique spectrum count	Total spectrum count	Percentage of total spectra	Percentage sequence coverage
Tumor tissue	HLA class II histocompatibility antigen, DRB1-7 beta chain GN=HLA-DRB1 PE=1 SV=1	2B17_HUMAN	29,048.10	100.00%	2	3	8	0.00612%	22.90%
Tumor tissue	HLA-B associated transcript 3, isoform CRA_a GN=BAT3 PE=1 SV=1	sp|P46379|BAG6_HUMAN	118,691.40	100.00%	5	5	6	0.00459%	7.02%
Tumor tissue	HLA-B associated transcript 5, isoform CRA_b GN=ABHD16A PE=1 SV=1	A0A0G2JJD3_HUMAN	67,520.20	100.00%	5	5	5	0.00383%	9.82%
Tumor tissue	Homeobox protein Meis1 GN=MEIS1 PE=1 SV=1	F5GYS8_HUMAN	36,014.00	100.00%	1	1	3	0.00230%	12.00%
Pap test	Hornerin GN=HRNR PE=1 SV=2	HORN_HUMAN	282,372.70	100.00%	3	5	5	0.00780%	1.75%
Swab	Hornerin GN=HRNR PE=1 SV=2	HORN_HUMAN	282,372.70	100.00%	4	6	6	0.00425%	2.14%
Tumor tissue	Host cell factor 1 GN=HCFC1 PE=1 SV=2	sp|P51610|HCFC1_HUMAN	213,468.10	100.00%	12	14	14	0.01070%	7.74%
Pap test	Hsc70-interacting protein GN=ST13 PE=1 SV=2	F10A1_HUMAN	41,332.40	100.00%	3	4	6	0.00936%	10.60%
Swab	Hsc70-interacting protein GN=ST13 PE=1 SV=2	F10A1_HUMAN	41,332.40	100.00%	6	6	9	0.00638%	20.90%
Tumor tissue	Hsc70-interacting protein GN=ST13 PE=1 SV=2	F10A1_HUMAN	41,332.40	100.00%	4	7	10	0.00766%	13.80%
Pap test	Hsp90 co-chaperone Cdc37 GN=CDC37 PE=1 SV=1	CDC37_HUMAN	44,468.20	99.50%	1	1	1	0.00156%	5.56%
Swab	Hsp90 co-chaperone Cdc37 GN=CDC37 PE=1 SV=1	CDC37_HUMAN	44,468.20	100.00%	3	4	5	0.00354%	9.79%
Tumor tissue	Hsp90 co-chaperone Cdc37 GN=CDC37 PE=1 SV=1	CDC37_HUMAN	44,468.20	100.00%	9	9	9	0.00689%	31.20%
Tumor tissue	Huntingtin GN=HTT PE=1 SV=2	HD_HUMAN	347,603.10	100.00%	2	2	2	0.00153%	0.80%
Tumor tissue	Huntingtin-interacting protein K GN=HYPK PE=1 SV=2	HYPK_HUMAN	14,665.70	100.00%	2	3	4	0.00306%	23.30%
Tumor tissue	Hyaluronan and proteoglycan link protein 3 GN=HAPLN3 PE=1 SV=1	HPLN3_HUMAN	47,756.60	100.00%	2	3	3	0.00230%	7.82%
Tumor tissue	Hydroxyacylglutathione hydrolase, mitochondrial (Fragment) GN=HAGH PE=1 SV=1	sp|Q16775|GLO2_HUMAN	33,805.50	100.00%	2	3	3	0.00230%	9.62%

Biological sample	Protein name	Protein accession numbers	Protein molecular weight (Da)	Protein identification probability	Exclusive unique peptide count	Exclusive unique spectrum count	Total spectrum count	Percentage of total spectra	Percentage sequence coverage
Tumor tissue	Hydroxymethylglutaryl-CoA lyase, mitochondrial GN=HMGCL PE=1 SV=2	sp|P35914|HMGCL_HUMAN	34,360.40	100.00%	4	5	8	0.00612%	15.70%
Tumor tissue	Hydroxysteroid dehydrogenase-like protein 2 GN=HSDL2 PE=1 SV=1	sp|Q6YN16|HSDL2_HUMAN	45,396.60	100.00%	3	3	3	0.00230%	12.90%
Pap test	Hypoxanthine-guanine phosphoribosyltransferase GN=HPRT1 PE=1 SV=2	HPRT_HUMAN	24,580.20	100.00%	3	3	5	0.00780%	17.00%
Swab	Hypoxanthine-guanine phosphoribosyltransferase GN=HPRT1 PE=1 SV=2	HPRT_HUMAN	24,580.20	100.00%	3	4	6	0.00425%	25.70%
Tumor tissue	Hypoxanthine-guanine phosphoribosyltransferase GN=HPRT1 PE=1 SV=2	HPRT_HUMAN	24,580.20	100.00%	5	7	9	0.00689%	24.80%
Pap test	Hypoxia up-regulated protein 1 GN=HYOU1 PE=1 SV=1	sp|Q9Y4L1|HYOU1_HUMAN	104,780.30	99.70%	1	1	1	0.00156%	2.24%
Swab	Hypoxia up-regulated protein 1 GN=HYOU1 PE=1 SV=1	sp|Q9Y4L1|HYOU1_HUMAN	104,780.30	100.00%	1	1	1	0.00071%	2.24%
Tumor tissue	Hypoxia up-regulated protein 1 GN=HYOU1 PE=1 SV=1	sp|Q9Y4L1|HYOU1_HUMAN	104,780.30	100.00%	18	27	53	0.04060%	31.20%
Pap test	Ig alpha-1 chain C region GN=IGHA1 PE=1 SV=2	IGHA1_HUMAN	37,653.80	100.00%	6	12	480	0.74900%	59.50%
Swab	Ig alpha-1 chain C region GN=IGHA1 PE=1 SV=2	IGHA1_HUMAN	37,653.80	100.00%	7	13	744	0.52700%	62.00%
Tumor tissue	Ig alpha-1 chain C region GN=IGHA1 PE=1 SV=2	IGHA1_HUMAN	37,653.80	100.00%	2	2	14	0.01070%	17.00%
Pap test	Ig alpha-2 chain C region (Fragment) GN=IGHA2 PE=1 SV=1	A0A0G2JMB2_HUMAN	36,507.30	100.00%	1	2	383	0.59800%	66.50%
Swab	Ig alpha-2 chain C region (Fragment) GN=IGHA2 PE=1 SV=1	A0A0G2JMB2_HUMAN	36,507.30	100.00%	1	6	663	0.47000%	69.10%
Pap test	Ig delta chain C region GN=IGHD PE=1 SV=1	sp|P01880|IGHD_HUMAN	47,539.60	100.00%	4	4	5	0.00780%	15.10%
Swab	Ig delta chain C region GN=IGHD PE=1 SV=1	sp|P01880|IGHD_HUMAN	47,539.60	100.00%	4	4	7	0.00496%	14.30%
Pap test	Ig gamma-1 chain C region GN=IGHG1 PE=1 SV=1	IGHG1_HUMAN	36,105.00	100.00%	9	18	732	1.14000%	65.20%

Biological sample	Protein name	Protein accession numbers	Protein molecular weight (Da)	Protein identification probability	Exclusive unique peptide count	Exclusive unique spectrum count	Total spectrum count	Percentage of total spectra	Percentage sequence coverage
Swab	Ig gamma-1 chain C region GN=IGHG1 PE=1 SV=1	IGHG1_HUMAN	36,105.00	100.00%	7	16	984	0.69700%	57.90%
Tumor tissue	Ig gamma-1 chain C region GN=IGHG1 PE=1 SV=1	IGHG1_HUMAN	36,105.00	100.00%	4	8	180	0.13800%	32.70%
Pap test	Ig gamma-2 chain C region GN=IGHG2 PE=1 SV=2	IGHG2_HUMAN	35,899.20	100.00%	7	14	414	0.64600%	54.00%
Swab	Ig gamma-2 chain C region GN=IGHG2 PE=1 SV=2	IGHG2_HUMAN	35,899.20	100.00%	5	10	603	0.42700%	50.60%
Tumor tissue	Ig gamma-2 chain C region GN=IGHG2 PE=1 SV=2	IGHG2_HUMAN	35,899.20	100.00%	3	10	81	0.06200%	18.40%
Pap test	Ig gamma-3 chain C region (Fragment) GN=IGHG3 PE=1 SV=1	IGHG3_HUMAN	41,325.80	100.00%	6	14	376	0.58700%	48.00%
Swab	Ig gamma-3 chain C region (Fragment) GN=IGHG3 PE=1 SV=1	IGHG3_HUMAN	41,325.80	100.00%	6	15	661	0.46800%	46.20%
Tumor tissue	Ig gamma-3 chain C region (Fragment) GN=IGHG3 PE=1 SV=1	IGHG3_HUMAN	41,325.80	99.80%	1	1	118	0.09030%	14.60%
Pap test	Ig gamma-4 chain C region GN=IGHG4 PE=1 SV=1	IGHG4_HUMAN	35,939.50	100.00%	3	9	252	0.39300%	45.00%
Swab	Ig gamma-4 chain C region GN=IGHG4 PE=1 SV=1	IGHG4_HUMAN	35,939.50	100.00%	4	8	263	0.18600%	54.10%
Tumor tissue	Ig gamma-4 chain C region GN=IGHG4 PE=1 SV=1	IGHG4_HUMAN	35,939.50	100.00%	1	2	78	0.05970%	17.70%
Pap test	Ig kappa chain C region GN=IGKC PE=1 SV=1	IGKC_HUMAN	11,608.60	100.00%	9	21	608	0.94900%	89.60%
Swab	Ig kappa chain C region GN=IGKC PE=1 SV=1	IGKC_HUMAN	11,608.60	100.00%	9	27	711	0.50400%	89.60%
Tumor tissue	Ig kappa chain C region GN=IGKC PE=1 SV=1	IGKC_HUMAN	11,608.60	100.00%	4	12	83	0.06350%	49.10%
Pap test	Ig lambda-2 chain C regions (Fragment) GN=IGLC2 PE=4 SV=1	LAC2_HUMAN	11,319.10	100.00%	3	5	196	0.30600%	67.00%
Swab	Ig lambda-2 chain C regions (Fragment) GN=IGLC2 PE=4 SV=1	LAC2_HUMAN	11,319.10	100.00%	3	6	150	0.10600%	74.50%
Tumor tissue	Ig lambda-2 chain C regions (Fragment) GN=IGLC2 PE=4 SV=1	LAC2_HUMAN	11,319.10	99.50%	1	1	24	0.01840%	41.50%
Pap test	Ig lambda-7 chain C region (Fragment) GN=IGLC7 PE=1 SV=2	LAC7_HUMAN	11,307.00	100.00%	2	2	150	0.23400%	60.40%

Biological sample	Protein name	Protein accession numbers	Protein molecular weight (Da)	Protein identification probability	Exclusive unique peptide count	Exclusive unique spectrum count	Total spectrum count	Percentage of total spectra	Percentage sequence coverage
Swab	Ig lambda-7 chain C region (Fragment) GN=IGLC7 PE=1 SV=2	LAC7_HUMAN	11,307.00	98.30%	1	1	55	0.03900%	41.50%
Pap test	Ig mu chain C region (Fragment) GN=IGHM PE=1 SV=2	A0A075B6N9_HUMAN	49,438.80	100.00%	11	18	53	0.08270%	29.80%
Swab	Ig mu chain C region (Fragment) GN=IGHM PE=1 SV=2	A0A075B6N9_HUMAN	49,438.80	100.00%	14	22	88	0.06240%	41.70%
Tumor tissue	Ig mu chain C region (Fragment) GN=IGHM PE=1 SV=2	A0A075B6N9_HUMAN	49,438.80	100.00%	7	12	47	0.03600%	20.10%
Pap test	IgGFc-binding protein GN=FCGBP PE=1 SV=3	FCGBP_HUMAN	571,982.80	100.00%	66	91	163	0.25400%	19.20%
Swab	IgGFc-binding protein GN=FCGBP PE=1 SV=3	FCGBP_HUMAN	571,982.80	100.00%	68	119	304	0.21500%	19.70%
Tumor tissue	Immunity-related GTPase family Q protein GN=IRGQ PE=1 SV=1	IRGQ_HUMAN	62,718.50	100.00%	2	3	4	0.00306%	5.94%
Pap test	Immunoglobulin heavy variable 1-18 GN=IGHV1-18 PE=3 SV=1	HV118_HUMAN	12,820.30	99.70%	1	3	8	0.01250%	23.90%
Swab	Immunoglobulin heavy variable 1-18 GN=IGHV1-18 PE=3 SV=1	HV118_HUMAN	12,820.30	99.80%	1	2	10	0.00709%	23.90%
Tumor tissue	Immunoglobulin heavy variable 1-18 GN=IGHV1-18 PE=3 SV=1	HV118_HUMAN	12,820.30	98.40%	1	1	1	0.00077%	14.50%
Pap test	Immunoglobulin heavy variable 1-2 GN=IGHV1-2 PE=1 SV=2	HV102_HUMAN	13,084.60	99.90%	1	2	5	0.00780%	20.50%
Swab	Immunoglobulin heavy variable 1-2 GN=IGHV1-2 PE=1 SV=2	HV102_HUMAN	13,084.60	100.00%	2	2	9	0.00638%	22.20%
Pap test	Immunoglobulin heavy variable 1-24 GN=IGHV1-24 PE=3 SV=1	HV124_HUMAN	12,824.20	100.00%	2	7	14	0.02180%	34.20%
Swab	Immunoglobulin heavy variable 1-24 GN=IGHV1-24 PE=3 SV=1	HV124_HUMAN	12,824.20	100.00%	2	6	13	0.00921%	34.20%
Pap test	Immunoglobulin heavy variable 1-3 GN=IGHV1-3 PE=3 SV=1	HV103_HUMAN	13,007.50	100.00%	2	5	13	0.02030%	35.00%
Swab	Immunoglobulin heavy variable 1-3 GN=IGHV1-3 PE=3 SV=1	HV103_HUMAN	13,007.50	100.00%	3	5	22	0.01560%	39.30%
Pap test	Immunoglobulin heavy variable 1-46 GN=IGHV1-46 PE=1 SV=2	HV146_HUMAN	12,932.30	100.00%	2	3	8	0.01250%	43.60%
Swab	Immunoglobulin heavy variable 1-46 GN=IGHV1-46 PE=1 SV=2	HV146_HUMAN	12,932.30	99.70%	1	3	16	0.01130%	22.20%

Biological sample	Protein name	Protein accession numbers	Protein molecular weight (Da)	Protein identification probability	Exclusive unique peptide count	Exclusive unique spectrum count	Total spectrum count	Percentage of total spectra	Percentage sequence coverage
Pap test	Immunoglobulin heavy variable 1-69 GN=IGHV1-69 PE=1 SV=2	HV169_HUMAN	12,659.20	100.00%	1	2	13	0.02030%	54.70%
Swab	Immunoglobulin heavy variable 1-69 GN=IGHV1-69 PE=1 SV=2	HV169_HUMAN	12,659.20	100.00%	1	1	12	0.00850%	20.50%
Tumor tissue	Immunoglobulin heavy variable 1-69 GN=IGHV1-69 PE=1 SV=2	HV169_HUMAN	12,659.20	99.80%	1	1	2	0.00153%	23.90%
Pap test	Immunoglobulin heavy variable 2-26 GN=IGHV2-26 PE=3 SV=1	HV226_HUMAN	13,182.60	100.00%	2	3	4	0.00624%	35.30%
Swab	Immunoglobulin heavy variable 2-26 GN=IGHV2-26 PE=3 SV=1	HV226_HUMAN	13,182.60	99.90%	1	1	1	0.00071%	18.50%
Tumor tissue	Immunoglobulin heavy variable 2-26 GN=IGHV2-26 PE=3 SV=1	HV226_HUMAN	13,182.60	99.80%	1	2	3	0.00230%	19.30%
Pap test	Immunoglobulin heavy variable 3-13 GN=IGHV3-13 PE=1 SV=2	HV313_HUMAN	12,506.20	99.90%	1	1	26	0.04060%	19.00%
Pap test	Immunoglobulin heavy variable 3-15 GN=IGHV3-15 PE=3 SV=1	HV315_HUMAN	12,926.00	100.00%	2	2	4	0.00624%	30.30%
Swab	Immunoglobulin heavy variable 3-15 GN=IGHV3-15 PE=3 SV=1	HV315_HUMAN	12,926.00	100.00%	2	2	5	0.00354%	22.70%
Tumor tissue	Immunoglobulin heavy variable 3-15 GN=IGHV3-15 PE=3 SV=1	HV315_HUMAN	12,926.00	100.00%	1	1	1	0.00077%	10.90%
Pap test	Immunoglobulin heavy variable 3-23 GN=IGHV3-23 PE=1 SV=2	HV323_HUMAN	12,582.50	99.80%	1	2	38	0.05930%	37.60%
Swab	Immunoglobulin heavy variable 3-23 GN=IGHV3-23 PE=1 SV=2	HV323_HUMAN	12,582.50	99.80%	1	1	38	0.02690%	41.00%
Pap test	Immunoglobulin heavy variable 3-49 GN=IGHV3-49 PE=1 SV=1	HV349_HUMAN	13,056.00	100.00%	4	7	23	0.03590%	37.00%
Swab	Immunoglobulin heavy variable 3-49 GN=IGHV3-49 PE=1 SV=1	HV349_HUMAN	13,056.00	100.00%	4	6	22	0.01560%	37.00%
Tumor tissue	Immunoglobulin heavy variable 3-49 GN=IGHV3-49 PE=1 SV=1	HV349_HUMAN	13,056.00	100.00%	2	3	8	0.00612%	16.80%
Pap test	Immunoglobulin heavy variable 3-7 GN=IGHV3-7 PE=1 SV=2	HV307_HUMAN	12,942.70	99.90%	1	1	29	0.04530%	26.50%
Swab	Immunoglobulin heavy variable 3-7 GN=IGHV3-7 PE=1 SV=2	HV307_HUMAN	12,942.70	100.00%	1	2	36	0.02550%	35.00%
Tumor tissue	Immunoglobulin heavy variable 3-7 GN=IGHV3-7 PE=1 SV=2	HV307_HUMAN	12,942.70	99.60%	1	1	6	0.00459%	17.10%

Biological sample	Protein name	Protein accession numbers	Protein molecular weight (Da)	Protein identification probability	Exclusive unique peptide count	Exclusive unique spectrum count	Total spectrum count	Percentage of total spectra	Percentage sequence coverage
Pap test	Immunoglobulin heavy variable 3-9 GN=IGHV3-9 PE=1 SV=2	HV309_HUMAN	12,945.10	100.00%	2	2	32	0.04990%	34.70%
Swab	Immunoglobulin heavy variable 3-9 GN=IGHV3-9 PE=1 SV=2	HV309_HUMAN	12,945.10	100.00%	1	1	35	0.02480%	22.00%
Pap test	Immunoglobulin heavy variable 4-34 GN=IGHV4-34 PE=1 SV=2	HV434_HUMAN	13,815.20	100.00%	2	2	12	0.01870%	37.40%
Swab	Immunoglobulin heavy variable 4-34 GN=IGHV4-34 PE=1 SV=2	HV434_HUMAN	13,815.20	100.00%	2	2	33	0.02340%	39.00%
Pap test	Immunoglobulin J chain GN=JCHAIN PE=1 SV=4	IGJ_HUMAN	18,098.50	100.00%	7	13	65	0.10100%	59.10%
Swab	Immunoglobulin J chain GN=JCHAIN PE=1 SV=4	IGJ_HUMAN	18,098.50	100.00%	6	13	171	0.12100%	52.20%
Pap test	Immunoglobulin kappa variable 1-17 GN=IGKV1-17 PE=1 SV=2	KV117_HUMAN	12,779.00	100.00%	2	2	5	0.00780%	23.10%
Swab	Immunoglobulin kappa variable 1-17 GN=IGKV1-17 PE=1 SV=2	KV117_HUMAN	12,779.00	100.00%	2	3	9	0.00638%	23.10%
Tumor tissue	Immunoglobulin kappa variable 1-17 GN=IGKV1-17 PE=1 SV=2	KV117_HUMAN	12,779.00	99.80%	1	1	1	0.00077%	12.80%
Pap test	Immunoglobulin kappa variable 1-39 GN=IGKV1-39 PE=1 SV=2	KV139_HUMAN	12,738.00	99.90%	1	1	10	0.01560%	29.10%
Tumor tissue	Immunoglobulin kappa variable 1-39 GN=IGKV1-39 PE=1 SV=2	KV139_HUMAN	12,738.00	100.00%	1	1	8	0.00612%	29.10%
Pap test	Immunoglobulin kappa variable 1-5 GN=IGKV1-5 PE=1 SV=2	KV105_HUMAN	12,782.10	99.80%	1	1	1	0.00156%	9.40%
Swab	Immunoglobulin kappa variable 1-5 GN=IGKV1-5 PE=1 SV=2	KV105_HUMAN	12,782.10	100.00%	2	2	6	0.00425%	13.70%
Tumor tissue	Immunoglobulin kappa variable 1-5 GN=IGKV1-5 PE=1 SV=2	KV105_HUMAN	12,782.10	99.90%	1	1	2	0.00153%	15.40%
Pap test	Immunoglobulin kappa variable 1D-33 GN=IGKV1D-33 PE=1 SV=2	KVD33_HUMAN	12,849.00	99.20%	1	2	9	0.01400%	13.70%
Swab	Immunoglobulin kappa variable 1D-33 GN=IGKV1D-33 PE=1 SV=2	KVD33_HUMAN	12,849.00	100.00%	2	4	14	0.00992%	16.20%
Swab	Immunoglobulin kappa variable 2-30 GN=IGKV2-30 PE=3 SV=2	KV230_HUMAN	13,185.00	99.80%	1	2	13	0.00921%	22.50%
Pap test	Immunoglobulin kappa variable 2D-40 GN=IGKV2-40 PE=1 SV=1	A0A087X0Q4_HUMAN	11,434.00	99.90%	1	2	15	0.02340%	28.80%

Biological sample	Protein name	Protein accession numbers	Protein molecular weight (Da)	Protein identification probability	Exclusive unique peptide count	Exclusive unique spectrum count	Total spectrum count	Percentage of total spectra	Percentage sequence coverage
Swab	Immunoglobulin kappa variable 2D-40 GN=IGKV2-40 PE=1 SV=1	A0A087X0Q4_HUMAN	11,434.00	99.90%	1	2	14	0.00992%	28.80%
Pap test	Immunoglobulin kappa variable 3-20 GN=IGKV3-20 PE=1 SV=2	KV320_HUMAN	12,557.70	100.00%	2	2	17	0.02650%	40.50%
Swab	Immunoglobulin kappa variable 3-20 GN=IGKV3-20 PE=1 SV=2	KV320_HUMAN	12,557.70	100.00%	2	2	30	0.02130%	40.50%
Tumor tissue	Immunoglobulin kappa variable 3-20 GN=IGKV3-20 PE=1 SV=2	KV320_HUMAN	12,557.70	98.10%	1	1	7	0.00536%	21.60%
Pap test	Immunoglobulin kappa variable 3-20 GN=IGKV3D-20 PE=3 SV=1	KVD20_HUMAN	12,515.70	99.80%	1	1	19	0.02960%	21.60%
Swab	Immunoglobulin kappa variable 3-20 GN=IGKV3D-20 PE=3 SV=1	KVD20_HUMAN	12,515.70	98.00%	1	1	27	0.01910%	21.60%
Pap test	Immunoglobulin kappa variable 4-1 GN=IGKV4-1 PE=1 SV=1	KV401_HUMAN	13,380.30	100.00%	3	5	8	0.01250%	29.80%
Swab	Immunoglobulin kappa variable 4-1 GN=IGKV4-1 PE=1 SV=1	KV401_HUMAN	13,380.30	100.00%	2	3	4	0.00283%	22.30%
Tumor tissue	Immunoglobulin kappa variable 4-1 GN=IGKV4-1 PE=1 SV=1	KV401_HUMAN	13,380.30	100.00%	2	2	2	0.00153%	22.30%
Pap test	Immunoglobulin lambda variable 1-44 GN=IGLV1-44 PE=1 SV=2	LV144_HUMAN	12,201.70	99.80%	1	1	15	0.02340%	38.50%
Pap test	Immunoglobulin lambda variable 1-47 GN=IGLV1-47 PE=1 SV=2	LV147_HUMAN	12,283.90	99.80%	1	1	40	0.06240%	24.80%
Swab	Immunoglobulin lambda variable 1-47 GN=IGLV1-47 PE=1 SV=2	LV147_HUMAN	12,283.90	99.80%	1	1	19	0.01350%	24.80%
Tumor tissue	Immunoglobulin lambda variable 1-47 GN=IGLV1-47 PE=1 SV=2	LV147_HUMAN	12,283.90	99.50%	1	1	4	0.00306%	24.80%
Pap test	Immunoglobulin lambda variable 1-51 GN=IGLV1-51 PE=1 SV=2	LV151_HUMAN	12,249.00	100.00%	2	3	5	0.00780%	31.60%
Swab	Immunoglobulin lambda variable 1-51 GN=IGLV1-51 PE=1 SV=2	LV151_HUMAN	12,249.00	100.00%	3	5	12	0.00850%	13.70%
Tumor tissue	Immunoglobulin lambda variable 1-51 GN=IGLV1-51 PE=1 SV=2	LV151_HUMAN	12,249.00	99.90%	1	1	1	0.00077%	13.70%
Swab	Immunoglobulin lambda variable 2-23 GN=IGLV2-23 PE=1 SV=2	LV223_HUMAN	11,893.40	100.00%	2	3	8	0.00567%	14.20%
Pap test	Immunoglobulin lambda variable 2-8 GN=IGLV2-8 PE=1 SV=2	LV208_HUMAN	12,381.70	99.60%	1	1	6	0.00936%	13.60%

Biological sample	Protein name	Protein accession numbers	Protein molecular weight (Da)	Protein identification probability	Exclusive unique peptide count	Exclusive unique spectrum count	Total spectrum count	Percentage of total spectra	Percentage sequence coverage
Pap test	Immunoglobulin lambda variable 3-19 GN=IGLV3-19 PE=1 SV=2	LV319_HUMAN	12,042.50	100.00%	2	4	7	0.01090%	35.70%
Swab	Immunoglobulin lambda variable 3-19 GN=IGLV3-19 PE=1 SV=2	LV319_HUMAN	12,042.50	100.00%	3	6	12	0.00850%	43.80%
Swab	Immunoglobulin lambda variable 3-21 GN=IGLV3-21 PE=1 SV=2	LV321_HUMAN	12,445.30	99.90%	1	2	37	0.02620%	48.70%
Pap test	Immunoglobulin lambda variable 3-27 GN=IGLV3-27 PE=1 SV=2	LV327_HUMAN	12,164.90	99.90%	1	1	2	0.00312%	18.60%
Swab	Immunoglobulin lambda variable 3-27 GN=IGLV3-27 PE=1 SV=2	LV327_HUMAN	12,164.90	99.20%	1	1	5	0.00354%	18.60%
Pap test	Immunoglobulin lambda variable 7-43 GN=IGLV7-43 PE=3 SV=2	LV743_HUMAN	12,451.10	99.50%	1	1	10	0.01560%	15.40%
Swab	Immunoglobulin lambda variable 7-43 GN=IGLV7-43 PE=3 SV=2	LV743_HUMAN	12,451.10	99.80%	1	1	4	0.00283%	15.40%
Pap test	Immunoglobulin lambda-like polypeptide 5 GN=IGLL5 PE=1 SV=1	A0A0B4J231_HUMAN	23,150.00	100.00%	4	6	173	0.27000%	36.70%
Swab	Immunoglobulin lambda-like polypeptide 5 GN=IGLL5 PE=1 SV=1	A0A0B4J231_HUMAN	23,150.00	100.00%	4	5	83	0.05880%	37.20%
Tumor tissue	Immunoglobulin lambda-like polypeptide 5 GN=IGLL5 PE=1 SV=1	A0A0B4J231_HUMAN	23,150.00	100.00%	2	3	23	0.01760%	19.50%
Tumor tissue	Immunoglobulin superfamily containing leucine-rich repeat protein GN=ISLR PE=2 SV=1	ISLR_HUMAN	45,998.30	100.00%	4	5	5	0.00383%	12.40%
Tumor tissue	Immunoglobulin-binding protein 1 GN=IGBP1 PE=1 SV=1	IGBP1_HUMAN	39,222.50	100.00%	3	3	3	0.00230%	12.40%
Tumor tissue	Importin subunit alpha-1 GN=KPNA2 PE=1 SV=1	IMA1_HUMAN	57,862.50	100.00%	5	7	10	0.00766%	12.70%
Swab	Importin subunit alpha-3 GN=KPNA4 PE=1 SV=1	IMA3_HUMAN	57,886.90	99.90%	2	2	2	0.00142%	7.49%
Tumor tissue	Importin subunit alpha-3 GN=KPNA4 PE=1 SV=1	IMA3_HUMAN	57,886.90	100.00%	2	2	4	0.00306%	8.64%
Tumor tissue	Importin subunit alpha-4 GN=KPNA3 PE=1 SV=2	IMA4_HUMAN	57,811.10	100.00%	1	1	3	0.00230%	7.87%
Tumor tissue	Importin subunit alpha-7 GN=KPNA6 PE=1 SV=1	S4R3E5_HUMAN	21,700.00	100.00%	2	2	2	0.00153%	12.90%

Biological sample	Protein name	Protein accession numbers	Protein molecular weight (Da)	Protein identification probability	Exclusive unique peptide count	Exclusive unique spectrum count	Total spectrum count	Percentage of total spectra	Percentage sequence coverage
Pap test	Importin subunit beta-1 GN=KPNB1 PE=1 SV=2	sp|Q14974|IMB1_HUMAN	97,171.30	100.00%	1	1	7	0.01090%	5.59%
Swab	Importin subunit beta-1 GN=KPNB1 PE=1 SV=2	sp|Q14974|IMB1_HUMAN	97,171.30	100.00%	4	5	11	0.00780%	12.60%
Tumor tissue	Importin subunit beta-1 GN=KPNB1 PE=1 SV=2	sp|Q14974|IMB1_HUMAN	97,171.30	100.00%	6	8	45	0.03440%	22.80%
Tumor tissue	Importin-7 GN=IPO7 PE=1 SV=1	IPO7_HUMAN	119,519.50	100.00%	4	4	17	0.01300%	8.67%
Swab	Importin-9 GN=IPO9 PE=1 SV=3	IPO9_HUMAN	115,964.00	100.00%	1	1	1	0.00071%	1.73%
Tumor tissue	Importin-9 GN=IPO9 PE=1 SV=3	IPO9_HUMAN	115,964.00	100.00%	7	9	13	0.00995%	9.03%
Tumor tissue	Inhibitor of nuclear factor kappa-B kinase-interacting protein GN=IKBIP PE=1 SV=1	sp|Q70UQ0|IKIP_HUMAN	39,310.80	100.00%	3	3	4	0.00306%	11.70%
Tumor tissue	Inner nuclear membrane protein Man1 GN=LEMD3 PE=1 SV=2	MAN1_HUMAN	99,999.40	100.00%	3	3	3	0.00230%	4.28%
Pap test	Inorganic pyrophosphatase GN=PPA1 PE=1 SV=2	IPYR_HUMAN	32,660.60	100.00%	11	15	21	0.03280%	54.70%
Swab	Inorganic pyrophosphatase GN=PPA1 PE=1 SV=2	IPYR_HUMAN	32,660.60	100.00%	9	13	25	0.01770%	41.20%
Tumor tissue	Inorganic pyrophosphatase GN=PPA1 PE=1 SV=2	IPYR_HUMAN	32,660.60	100.00%	10	16	25	0.01910%	42.60%
Swab	Inosine triphosphate pyrophosphatase GN=ITPA PE=1 SV=2	sp|Q9BY32|ITPA_HUMAN	21,445.90	98.30%	1	1	2	0.00142%	4.64%
Tumor tissue	Inosine triphosphate pyrophosphatase GN=ITPA PE=1 SV=2	sp|Q9BY32|ITPA_HUMAN	21,445.90	100.00%	3	3	3	0.00230%	21.60%
Pap test	Inosine-5'-monophosphate dehydrogenase 2 (Fragment) GN=IMPDH2 PE=1 SV=1	IMDH2_HUMAN	51,069.60	99.80%	1	1	1	0.00156%	4.04%
Tumor tissue	Inosine-5'-monophosphate dehydrogenase 2 (Fragment) GN=IMPDH2 PE=1 SV=1	IMDH2_HUMAN	51,069.60	100.00%	13	16	20	0.01530%	31.30%

Biological sample	Protein name	Protein accession numbers	Protein molecular weight (Da)	Protein identification probability	Exclusive unique peptide count	Exclusive unique spectrum count	Total spectrum count	Percentage of total spectra	Percentage sequence coverage
Tumor tissue	Inositol 1,4,5-trisphosphate receptor type 2 GN=ITPR2 PE=1 SV=2	sp|Q14571|ITPR2_HUMAN	308,067.80	100.00%	2	2	3	0.00230%	1.55%
Tumor tissue	Inositol monophosphatase 3 GN=IMPAD1 PE=1 SV=1	IMPA3_HUMAN	38,681.70	99.90%	2	2	2	0.00153%	5.57%
Pap test	Inositol polyphosphate 1-phosphatase GN=INPP1 PE=1 SV=1	INPP_HUMAN	43,998.70	100.00%	2	3	4	0.00624%	4.51%
Swab	Inositol polyphosphate 1-phosphatase GN=INPP1 PE=1 SV=1	INPP_HUMAN	43,998.70	100.00%	1	1	1	0.00071%	6.27%
Tumor tissue	Inositol polyphosphate 1-phosphatase GN=INPP1 PE=1 SV=1	INPP_HUMAN	43,998.70	100.00%	2	2	2	0.00153%	5.51%
Swab	Insulin-degrading enzyme GN=IDE PE=1 SV=4	sp|P14735|IDE_HUMAN	117,974.00	100.00%	1	1	1	0.00071%	1.47%
Tumor tissue	Insulin-degrading enzyme GN=IDE PE=1 SV=4	sp|P14735|IDE_HUMAN	117,974.00	100.00%	12	13	13	0.00995%	15.00%
Tumor tissue	Insulin-like growth factor 2 mRNA-binding protein 2 GN=IGF2BP2 PE=1 SV=1	sp|Q9Y6M1|IF2B2_HUMAN	66,787.20	100.00%	7	9	13	0.00995%	20.80%
Pap test	Insulin-like growth factor binding protein 3 GN=IGFBP3 PE=1 SV=1	sp|P17936|IBP3_HUMAN	29,746.90	99.20%	1	1	1	0.00156%	8.89%
Swab	Insulin-like growth factor binding protein 3 GN=IGFBP3 PE=1 SV=1	sp|P17936|IBP3_HUMAN	29,746.90	100.00%	2	3	5	0.00354%	20.00%
Swab	Insulin-like growth factor-binding protein complex acid labile subunit GN=IGFALS PE=1 SV=1	sp|P35858|ALS_HUMAN	66,038.60	100.00%	3	3	4	0.00283%	7.27%
Tumor tissue	Insulin-like growth factor-binding protein complex acid labile subunit GN=IGFALS PE=1 SV=1	sp|P35858|ALS_HUMAN	66,038.60	100.00%	3	3	3	0.00230%	7.44%
Tumor tissue	Integrin alpha-1 GN=ITGA1 PE=1 SV=2	ITA1_HUMAN	130,850.40	100.00%	6	7	8	0.00612%	6.45%
Tumor tissue	Integrin alpha-5 GN=ITGA5 PE=1 SV=2	ITA5_HUMAN	114,538.50	100.00%	4	4	4	0.00306%	5.43%
Tumor tissue	Integrin alpha-X GN=ITGAX PE=1 SV=3	ITAX_HUMAN	127,830.30	100.00%	4	4	4	0.00306%	4.90%
Pap test	Integrin beta GN=ITGB2 PE=1 SV=1	D3DSM0_HUMAN	78,353.90	99.90%	1	1	3	0.00468%	5.90%
Swab	Integrin beta GN=ITGB2 PE=1 SV=1	D3DSM0_HUMAN	78,353.90	100.00%	1	1	6	0.00425%	11.50%

Biological sample	Protein name	Protein accession numbers	Protein molecular weight (Da)	Protein identification probability	Exclusive unique peptide count	Exclusive unique spectrum count	Total spectrum count	Percentage of total spectra	Percentage sequence coverage
Tumor tissue	Integrin beta GN=ITGB2 PE=1 SV=1	D3DSM0_HUMAN	78,353.90	100.00%	1	2	11	0.00842%	15.60%
Tumor tissue	Integrin beta-1 GN=ITGB1 PE=1 SV=2	sp|P05556|ITB1_HUMAN	88,415.10	100.00%	1	1	16	0.01220%	14.40%
Tumor tissue	Integrin beta-2 GN=ITGB2 PE=1 SV=2	ITB2_HUMAN	84,782.00	100.00%	1	1	12	0.00919%	14.60%
Tumor tissue	Integrin beta-4 GN=ITGB4 PE=1 SV=5	sp|P16144|ITB4_HUMAN	202,166.70	100.00%	6	6	6	0.00459%	4.06%
Tumor tissue	Integrin beta-5 GN=ITGB5 PE=1 SV=1	ITB5_HUMAN	88,054.80	100.00%	3	3	6	0.00459%	6.01%
Tumor tissue	Integrin-linked kinase-associated serine/threonine phosphatase 2C GN=ILKAP PE=1 SV=1	ILKAP_HUMAN	42,907.20	100.00%	3	3	3	0.00230%	7.40%
Tumor tissue	Integrin-linked protein kinase GN=ILK PE=1 SV=1	sp|Q13418|ILK_HUMAN	54,612.60	100.00%	1	1	21	0.01610%	22.60%
Pap test	Inter-alpha-trypsin inhibitor heavy chain H1 GN=ITIH1 PE=1 SV=3	sp|P19827|ITIH1_HUMAN	101,389.80	100.00%	5	5	7	0.01090%	6.92%
Swab	Inter-alpha-trypsin inhibitor heavy chain H1 GN=ITIH1 PE=1 SV=3	sp|P19827|ITIH1_HUMAN	101,389.80	100.00%	8	12	16	0.01130%	12.50%
Tumor tissue	Inter-alpha-trypsin inhibitor heavy chain H1 GN=ITIH1 PE=1 SV=3	sp|P19827|ITIH1_HUMAN	101,389.80	100.00%	9	16	26	0.01990%	15.30%
Pap test	Inter-alpha-trypsin inhibitor heavy chain H2 GN=ITIH2 PE=1 SV=2	ITIH2_HUMAN	105,218.00	100.00%	7	8	14	0.02180%	9.94%
Swab	Inter-alpha-trypsin inhibitor heavy chain H2 GN=ITIH2 PE=1 SV=2	ITIH2_HUMAN	105,218.00	100.00%	6	6	9	0.00638%	8.35%
Tumor tissue	Inter-alpha-trypsin inhibitor heavy chain H2 GN=ITIH2 PE=1 SV=2	ITIH2_HUMAN	105,218.00	100.00%	14	17	22	0.01680%	18.10%
Tumor tissue	Inter-alpha-trypsin inhibitor heavy chain H4 GN=ITIH4 PE=1 SV=4	sp|Q14624|ITIH4_HUMAN	103,358.40	100.00%	1	1	16	0.01220%	16.10%
Tumor tissue	Inter-alpha-trypsin inhibitor heavy chain H5 GN=ITIH5 PE=1 SV=1	sp|Q86UX2|ITIH5_HUMAN	77,402.80	100.00%	1	1	4	0.00306%	7.14%
Pap test	Intercellular adhesion molecule 1 GN=ICAM1 PE=1 SV=2	ICAM1_HUMAN	57,824.50	98.30%	1	1	1	0.00156%	1.88%
Tumor tissue	Intercellular adhesion molecule 1 GN=ICAM1 PE=1 SV=2	ICAM1_HUMAN	57,824.50	100.00%	7	8	11	0.00842%	18.00%

Biological sample	Protein name	Protein accession numbers	Protein molecular weight (Da)	Protein identification probability	Exclusive unique peptide count	Exclusive unique spectrum count	Total spectrum count	Percentage of total spectra	Percentage sequence coverage
Tumor tissue	Interferon regulatory factor 2-binding protein 2 GN=IRF2BP2 PE=1 SV=2	sp|Q7Z5L9|I2BP2_HUMAN	61,024.20	100.00%	4	4	4	0.00306%	13.10%
Tumor tissue	Interferon regulatory factor 2-binding protein-like GN=IRF2BPL PE=1 SV=1	I2BPL_HUMAN	82,658.10	100.00%	4	4	4	0.00306%	7.79%
Tumor tissue	Interferon regulatory factor 9 (Fragment) GN=IRF9 PE=1 SV=1	IRF9_HUMAN	43,696.10	100.00%	2	2	3	0.00230%	13.40%
Swab	Interferon-induced GTP-binding protein Mx1 GN=MX1 PE=1 SV=4	sp|P20591|MX1_HUMAN	75,522.40	100.00%	2	2	3	0.00213%	4.08%
Tumor tissue	Interferon-induced GTP-binding protein Mx1 GN=MX1 PE=1 SV=4	sp|P20591|MX1_HUMAN	75,522.40	100.00%	11	14	24	0.01840%	29.80%
Tumor tissue	Interferon-induced GTP-binding protein Mx2 GN=MX2 PE=1 SV=1	sp|P20592|MX2_HUMAN	82,090.90	100.00%	2	2	7	0.00536%	8.81%
Tumor tissue	Interferon-induced protein with tetratricopeptide repeats 3 GN=IFIT3 PE=1 SV=1	IFIT3_HUMAN	55,986.00	100.00%	4	4	4	0.00306%	12.00%
Swab	Interferon-induced protein with tetratricopeptide repeats 5 GN=IFIT5 PE=1 SV=1	sp|Q13325|IFIT5_HUMAN	55,849.30	99.80%	1	1	1	0.00071%	4.98%
Tumor tissue	Interferon-induced protein with tetratricopeptide repeats 5 GN=IFIT5 PE=1 SV=1	sp|Q13325|IFIT5_HUMAN	55,849.30	100.00%	3	3	3	0.00230%	8.51%
Tumor tissue	Interferon-induced transmembrane protein 1 GN=IFITM1 PE=1 SV=3	IFM1_HUMAN	13,964.70	100.00%	3	5	5	0.00383%	30.40%
Tumor tissue	Interferon-induced, double-stranded RNA-activated protein kinase GN=EIF2AK2 PE=1 SV=2	sp|P19525|E2AK2_HUMAN	62,097.10	100.00%	9	11	13	0.00995%	18.70%
Tumor tissue	Interferon-stimulated gene 20 kDa protein GN=ISG20 PE=1 SV=2	sp|Q96AZ6|ISG20_HUMAN	20,363.60	100.00%	3	3	3	0.00230%	21.50%
Pap test	Interleukin enhancer-binding factor 2 GN=ILF2 PE=1 SV=1	ILF2_HUMAN	38,910.90	100.00%	2	2	2	0.00312%	6.82%
Swab	Interleukin enhancer-binding factor 2 GN=ILF2 PE=1 SV=1	ILF2_HUMAN	38,910.90	100.00%	2	2	2	0.00142%	6.82%
Tumor tissue	Interleukin enhancer-binding factor 2 GN=ILF2 PE=1 SV=1	ILF2_HUMAN	38,910.90	100.00%	12	16	42	0.03220%	34.90%

Biological sample	Protein name	Protein accession numbers	Protein molecular weight (Da)	Protein identification probability	Exclusive unique peptide count	Exclusive unique spectrum count	Total spectrum count	Percentage of total spectra	Percentage sequence coverage
Tumor tissue	Intraflagellar transport protein 25 homolog GN=HSPB11 PE=1 SV=1	IFT25_HUMAN	16,297.20	100.00%	3	4	4	0.00306%	36.10%
Swab	Intron-binding protein aquarius GN=AQR PE=1 SV=4	AQR_HUMAN	171,299.70	99.40%	1	1	1	0.00071%	2.02%
Tumor tissue	Intron-binding protein aquarius GN=AQR PE=1 SV=4	AQR_HUMAN	171,299.70	100.00%	2	2	2	0.00153%	1.55%
Pap test	Involucrin GN=IVL PE=1 SV=2	INVO_HUMAN	68,474.60	100.00%	25	45	91	0.14200%	58.10%
Swab	Involucrin GN=IVL PE=1 SV=2	INVO_HUMAN	68,474.60	100.00%	16	30	48	0.03400%	41.00%
Tumor tissue	IQ motif and SEC7 domain-containing protein 1 GN=IQSEC1 PE=1 SV=1	A0A087WWK8_HUMAN	124,478.50	99.20%	1	1	2	0.00153%	2.24%
Tumor tissue	Isoamyl acetate-hydrolyzing esterase 1 homolog (Fragment) GN=IAH1 PE=1 SV=1	sp|Q2TAA2|IAH1_HUMAN	25,197.50	100.00%	2	2	2	0.00153%	12.70%
Tumor tissue	Isoaspartyl peptidase/L-asparaginase GN=ASRGL1 PE=1 SV=2	sp|Q7L266|ASGL1_HUMAN	32,054.10	100.00%	4	4	6	0.00459%	17.90%
Pap test	Isochorismatase domain-containing protein 1 GN=ISOC1 PE=1 SV=3	ISOC1_HUMAN	32,236.70	99.90%	1	1	1	0.00156%	4.70%
Tumor tissue	Isochorismatase domain-containing protein 1 GN=ISOC1 PE=1 SV=3	ISOC1_HUMAN	32,236.70	100.00%	5	7	9	0.00689%	24.20%
Tumor tissue	Isocitrate dehydrogenase [NAD] subunit alpha, mitochondrial GN=IDH3A PE=1 SV=1	sp|P50213|IDH3A_HUMAN	39,592.50	100.00%	7	9	10	0.00766%	24.30%
Tumor tissue	Isocitrate dehydrogenase [NAD] subunit, mitochondrial GN=IDH3B PE=1 SV=1	sp|O43837|IDH3B_HUMAN	42,410.90	100.00%	8	9	10	0.00766%	29.20%
Pap test	Isocitrate dehydrogenase [NADP] cytoplasmic GN=IDH1 PE=1 SV=2	IDHC_HUMAN	46,660.50	100.00%	9	11	13	0.02030%	25.10%
Swab	Isocitrate dehydrogenase [NADP] cytoplasmic GN=IDH1 PE=1 SV=2	IDHC_HUMAN	46,660.50	100.00%	11	13	16	0.01130%	36.00%
Tumor tissue	Isocitrate dehydrogenase [NADP] cytoplasmic GN=IDH1 PE=1 SV=2	IDHC_HUMAN	46,660.50	100.00%	12	15	28	0.02140%	36.20%

Biological sample	Protein name	Protein accession numbers	Protein molecular weight (Da)	Protein identification probability	Exclusive unique peptide count	Exclusive unique spectrum count	Total spectrum count	Percentage of total spectra	Percentage sequence coverage
Pap test	Isocitrate dehydrogenase [NADP], mitochondrial GN=IDH2 PE=1 SV=2	sp|P48735|IDHP_HUMAN	50,910.40	100.00%	5	5	6	0.00936%	13.90%
Swab	Isocitrate dehydrogenase [NADP], mitochondrial GN=IDH2 PE=1 SV=2	sp|P48735|IDHP_HUMAN	50,910.40	100.00%	3	3	4	0.00283%	10.60%
Tumor tissue	Isocitrate dehydrogenase [NADP], mitochondrial GN=IDH2 PE=1 SV=2	sp|P48735|IDHP_HUMAN	50,910.40	100.00%	13	23	35	0.02680%	29.00%
Swab	Isoform 1 of Arf-GAP with SH3 domain, ANK repeat and PH domain-containing protein 1 GN=ASAP1	sp|Q9ULH1-2|ASAP1_HUMAN	125,902.20	99.80%	1	1	1	0.00071%	1.59%
Tumor tissue	Isoform 1 of Arf-GAP with SH3 domain, ANK repeat and PH domain-containing protein 1 GN=ASAP1	sp|Q9ULH1-2|ASAP1_HUMAN	125,902.20	100.00%	2	2	2	0.00153%	3.09%
Tumor tissue	Isoform 1 of Cat eye syndrome critical region protein 5 GN=CECR5	sp|Q9BXW7|CECR5_HUMAN	43,588.60	100.00%	3	4	8	0.00612%	13.00%
Tumor tissue	Isoform 1 of Chloride intracellular channel protein 5 GN=CLIC5	sp|Q9NZA1|CLIC5_HUMAN	28,180.50	100.00%	2	2	2	0.00153%	9.96%
Tumor tissue	Isoform 1 of Collagen alpha-1(II) chain GN=COL2A1	sp|P02458|CO2A1_HUMAN	134,390.50	100.00%	1	1	2	0.00153%	3.31%
Tumor tissue	Isoform 1 of Gamma-adducin GN=ADD3	sp|Q9UEY8-2|ADDG_HUMAN	75,672.50	100.00%	11	14	14	0.01070%	21.50%
Tumor tissue	Isoform 1 of Mitogen-activated protein kinase 8 GN=MAPK8	sp|P45983|MK08_HUMAN	48,296.60	100.00%	1	1	3	0.00230%	8.33%
Tumor tissue	Isoform 1 of Nitrilase homolog 1 GN=NIT1	sp|Q86X76|NIT1_HUMAN	34,323.50	100.00%	2	3	4	0.00306%	8.59%
Pap test	Isoform 1 of Plakophilin-1 GN=PKP1	sp|Q13835|PKP1_HUMAN	80,498.80	100.00%	8	8	8	0.01250%	15.20%
Tumor tissue	Isoform 1 of Plakophilin-2 GN=PKP2	sp|Q99959-2|PKP2_HUMAN	92,757.60	100.00%	1	1	9	0.00689%	14.20%
Tumor tissue	Isoform 1 of Poly [ADP-ribose] polymerase 14 GN=PARP14	sp|Q460N5|PAR14_HUMAN	202,802.30	100.00%	1	1	9	0.00689%	6.05%
Pap test	Isoform 1 of Protein POF1B GN=POF1B	sp|Q8WVV4|POF1B_HUMAN	68,065.50	100.00%	2	2	2	0.00312%	3.53%
Tumor tissue	Isoform 1 of RNA-binding protein Raly GN=RALY	sp|Q9UKM9|RALY_HUMAN	30,364.40	100.00%	6	8	13	0.00995%	25.20%
Tumor tissue	Isoform 1 of Serine/threonine-protein kinase DCLK1 GN=DCLK1	sp|O15075|DCLK1_HUMAN	81,102.30	100.00%	2	2	3	0.00230%	3.84%

Biological sample	Protein name	Protein accession numbers	Protein molecular weight (Da)	Protein identification probability	Exclusive unique peptide count	Exclusive unique spectrum count	Total spectrum count	Percentage of total spectra	Percentage sequence coverage
Pap test	Isoform 1 of Vinculin GN=VCL	sp|P18206|VINC_HUMAN	123,801.30	100.00%	28	40	83	0.13000%	40.90%
Swab	Isoform 1 of Vinculin GN=VCL	sp|P18206|VINC_HUMAN	123,801.30	100.00%	26	34	69	0.04890%	38.30%
Tumor tissue	Isoform 1 of Vinculin GN=VCL	sp|P18206|VINC_HUMAN	123,801.30	100.00%	41	64	156	0.11900%	54.30%
Pap test	Isoform 10 of Carcinoembryonic antigen-related cell adhesion molecule 1 GN=CEACAM1	sp|P13688|CEAM1_HUMAN	50,520.90	100.00%	2	3	14	0.02180%	13.50%
Swab	Isoform 10 of Carcinoembryonic antigen-related cell adhesion molecule 1 GN=CEACAM1	sp|P13688|CEAM1_HUMAN	50,520.90	99.90%	1	2	8	0.00567%	10.00%
Tumor tissue	Isoform 10 of Sickle tail protein homolog GN=KIAA1217	sp|Q5T5P2-10|SKT_HUMAN	150,495.40	100.00%	1	1	25	0.01910%	19.10%
Tumor tissue	Isoform 11 of Homeobox protein cut-like 1 GN=CUX1	sp|Q13948|CASP_HUMAN	77,458.00	99.90%	1	1	2	0.00153%	4.75%
Tumor tissue	Isoform 12 of Sorbin and SH3 domain-containing protein 1 GN=SORBS1	sp|Q9BX66-12|SRBS1_HUMAN	143,746.00	100.00%	1	1	27	0.02070%	17.10%
Tumor tissue	Isoform 1B of Beta-arrestin-1 GN=ARRB1	sp|P49407|ARRB1_HUMAN	46,309.30	100.00%	1	1	3	0.00230%	10.00%
Tumor tissue	Isoform 1c of Oxysterol-binding protein-related protein 3 GN=OSBPL3	sp|Q9H4L5-4|OSBL3_HUMAN	97,327.00	100.00%	2	2	2	0.00153%	3.53%
Tumor tissue	Isoform 2 of [Pyruvate dehydrogenase (acetyl-transferring)] kinase isozyme 1, mitochondrial GN=PDK1	sp|Q15118|PDK1_HUMAN	51,624.70	100.00%	4	5	6	0.00459%	11.00%
Tumor tissue	Isoform 2 of [Pyruvate dehydrogenase (acetyl-transferring)] kinase isozyme 3, mitochondrial GN=PDK3	sp|Q15120|PDK3_HUMAN	48,045.10	100.00%	3	3	3	0.00230%	12.30%
Tumor tissue	Isoform 2 of [Pyruvate dehydrogenase [acetyl-transferring]]-phosphatase 1, mitochondrial GN=PDP1	sp|Q9P0J1|PDP1_HUMAN	61,054.70	100.00%	8	9	9	0.00689%	21.20%

Biological sample	Protein name	Protein accession numbers	Protein molecular weight (Da)	Protein identification probability	Exclusive unique peptide count	Exclusive unique spectrum count	Total spectrum count	Percentage of total spectra	Percentage sequence coverage
Tumor tissue	Isoform 2 of 116 kDa U5 small nuclear ribonucleoprotein component GN=EFTUD2	sp|Q15029-2|U5S1_HUMAN	105,385.60	100.00%	15	17	21	0.01610%	23.60%
Pap test	Isoform 2 of 14-3-3 protein sigma GN=SFN	sp|P31947|1433S_HUMAN	24,337.00	100.00%	5	8	29	0.04530%	38.00%
Swab	Isoform 2 of 14-3-3 protein sigma GN=SFN	sp|P31947|1433S_HUMAN	27,774.80	100.00%	6	9	34	0.02410%	43.10%
Tumor tissue	Isoform 2 of 14-3-3 protein sigma GN=SFN	sp|P31947|1433S_HUMAN	27,774.80	100.00%	3	3	8	0.00612%	27.30%
Tumor tissue	Isoform 2 of 1-phosphatidylinositol 4,5-bisphosphate phosphodiesterase beta-3 GN=PLCB3	sp|Q01970|PLCB3_HUMAN	131,208.80	100.00%	3	3	3	0.00230%	3.26%
Tumor tissue	Isoform 2 of 1-phosphatidylinositol 4,5-bisphosphate phosphodiesterase delta-1 GN=PLCD1	sp|P51178|PLCD1_HUMAN	88,136.80	100.00%	2	2	3	0.00230%	3.35%
Tumor tissue	Isoform 2 of 1-phosphatidylinositol 4,5-bisphosphate phosphodiesterase gamma-1 GN=PLCG1	sp|P19174|PLCG1_HUMAN	148,664.30	100.00%	3	3	4	0.00306%	3.10%
Pap test	Isoform 2 of 2,4-dienoyl-CoA reductase, mitochondrial GN=DECR1	sp|Q16698-2|DECR_HUMAN	34,995.60	100.00%	2	2	4	0.00624%	17.20%
Tumor tissue	Isoform 2 of 2,4-dienoyl-CoA reductase, mitochondrial GN=DECR1	sp|Q16698-2|DECR_HUMAN	34,995.60	100.00%	4	7	15	0.01150%	27.30%
Tumor tissue	Isoform 2 of 2-5A-dependent ribonuclease GN=RNASEL	sp|Q05823|RN5A_HUMAN	73,417.50	100.00%	3	3	3	0.00230%	6.90%
Pap test	Isoform 2 of 26S proteasome non-ATPase regulatory subunit 11 GN=PSMD11	sp|O00231|PSD11_HUMAN	47,537.40	99.80%	1	1	1	0.00156%	3.07%
Tumor tissue	Isoform 2 of 26S proteasome non-ATPase regulatory subunit 11 GN=PSMD11	sp|O00231|PSD11_HUMAN	47,537.40	100.00%	12	15	19	0.01450%	36.60%

Biological sample	Protein name	Protein accession numbers	Protein molecular weight (Da)	Protein identification probability	Exclusive unique peptide count	Exclusive unique spectrum count	Total spectrum count	Percentage of total spectra	Percentage sequence coverage
Pap test	Isoform 2 of 26S proteasome non-ATPase regulatory subunit 5 GN=PSMD5	sp|Q16401|PSMD5_HUMAN	56,197.00	99.90%	1	1	1	0.00156%	1.95%
Swab	Isoform 2 of 26S proteasome non-ATPase regulatory subunit 5 GN=PSMD5	sp|Q16401|PSMD5_HUMAN	51,313.00	100.00%	4	4	5	0.00354%	10.20%
Tumor tissue	Isoform 2 of 26S proteasome non-ATPase regulatory subunit 5 GN=PSMD5	sp|Q16401|PSMD5_HUMAN	51,313.00	100.00%	8	9	12	0.00919%	21.30%
Tumor tissue	Isoform 2 of 28S ribosomal protein S29, mitochondrial GN=DAP3	sp|P51398|RT29_HUMAN	45,567.70	100.00%	5	5	6	0.00459%	21.30%
Tumor tissue	Isoform 2 of 39S ribosomal protein L11, mitochondrial GN=MRPL11	sp|Q9Y3B7|RM11_HUMAN	18,205.90	100.00%	2	3	3	0.00230%	17.50%
Tumor tissue	Isoform 2 of 39S ribosomal protein L47, mitochondrial GN=MRPL47	sp|Q9HD33|RM47_HUMAN	29,451.70	100.00%	2	2	2	0.00153%	9.57%
Tumor tissue	Isoform 2 of 39S ribosomal protein L55, mitochondrial GN=MRPL55	sp|Q7Z7F7|RM55_HUMAN	18,903.20	100.00%	2	3	3	0.00230%	18.90%
Pap test	Isoform 2 of 3-mercaptopyruvate sulfurtransferase GN=MPST	sp|P25325|THTM_HUMAN	33,178.80	100.00%	2	2	2	0.00312%	9.15%
Swab	Isoform 2 of 3-mercaptopyruvate sulfurtransferase GN=MPST	sp|P25325|THTM_HUMAN	35,250.00	100.00%	4	5	5	0.00354%	12.00%
Tumor tissue	Isoform 2 of 3-mercaptopyruvate sulfurtransferase GN=MPST	sp|P25325|THTM_HUMAN	33,178.80	100.00%	5	7	7	0.00536%	20.20%
Pap test	Isoform 2 of 40S ribosomal protein S20 GN=RPS20	sp|P60866|RS20_HUMAN	13,373.00	100.00%	2	3	3	0.00468%	16.20%
Swab	Isoform 2 of 40S ribosomal protein S20 GN=RPS20	sp|P60866|RS20_HUMAN	13,373.00	100.00%	2	2	2	0.00142%	16.20%
Tumor tissue	Isoform 2 of 40S ribosomal protein S20 GN=RPS20	sp|P60866|RS20_HUMAN	13,373.00	100.00%	3	7	9	0.00689%	19.00%
Tumor tissue	Isoform 2 of 5'-3' exoribonuclease 2 GN=XRN2	sp|Q9H0D6|XRN2_HUMAN	108,585.70	100.00%	7	8	10	0.00766%	10.80%
Tumor tissue	Isoform 2 of 5'-AMP-activated protein kinase catalytic subunit alpha-1 GN=PRKAA1	sp|Q13131|AAPK1_HUMAN	64,010.80	100.00%	5	7	7	0.00536%	12.40%

Biological sample	Protein name	Protein accession numbers	Protein molecular weight (Da)	Protein identification probability	Exclusive unique peptide count	Exclusive unique spectrum count	Total spectrum count	Percentage of total spectra	Percentage sequence coverage
Pap test	Isoform 2 of 6-phosphogluconate dehydrogenase, decarboxylating GN=PGD	sp|P52209|6PGD_HUMAN	51,874.20	100.00%	3	3	18	0.02810%	26.40%
Swab	Isoform 2 of 6-phosphogluconate dehydrogenase, decarboxylating GN=PGD	sp|P52209|6PGD_HUMAN	51,874.20	100.00%	3	6	44	0.03120%	28.70%
Tumor tissue	Isoform 2 of 6-phosphogluconate dehydrogenase, decarboxylating GN=PGD	sp|P52209|6PGD_HUMAN	53,141.90	100.00%	2	3	14	0.01070%	20.90%
Pap test	Isoform 2 of 72 kDa type IV collagenase GN=MMP2	sp|P08253|MMP2_HUMAN	68,833.60	99.20%	1	1	1	0.00156%	3.25%
Swab	Isoform 2 of 72 kDa type IV collagenase GN=MMP2	sp|P08253|MMP2_HUMAN	65,766.70	100.00%	2	2	2	0.00142%	5.31%
Tumor tissue	Isoform 2 of 72 kDa type IV collagenase GN=MMP2	sp|P08253|MMP2_HUMAN	65,766.70	100.00%	4	4	6	0.00459%	7.53%
Tumor tissue	Isoform 2 of Acetyl-CoA carboxylase 1 GN=ACACA	sp|Q13085|ACACA_HUMAN	257,241.00	100.00%	2	2	2	0.00153%	1.75%
Swab	Isoform 2 of Acidic leucine-rich nuclear phosphoprotein 32 family member B GN=ANP32B	sp|Q92688|AN32B_HUMAN	22,277.70	100.00%	1	1	6	0.00425%	19.00%
Tumor tissue	Isoform 2 of Acidic leucine-rich nuclear phosphoprotein 32 family member B GN=ANP32B	sp|Q92688|AN32B_HUMAN	28,788.70	100.00%	1	2	7	0.00536%	19.50%
Tumor tissue	Isoform 2 of Actin filament-associated protein 1-like 2 GN=AFAP1L2	sp|Q8N4X5|AF1L2_HUMAN	93,895.20	100.00%	2	2	3	0.00230%	6.63%
Tumor tissue	Isoform 2 of Adenosine 3'-phospho 5'-phosphosulfate transporter 1 GN=SLC35B2	sp|Q8TB61|S35B2_HUMAN	42,967.70	100.00%	2	2	3	0.00230%	5.36%
Pap test	Isoform 2 of Adenylyl cyclase-associated protein 1 GN=CAP1	sp|Q01518|CAP1_HUMAN	51,901.60	100.00%	6	8	14	0.02180%	20.30%
Swab	Isoform 2 of Adenylyl cyclase-associated protein 1 GN=CAP1	sp|Q01518|CAP1_HUMAN	51,830.50	100.00%	8	12	43	0.03050%	29.70%
Tumor tissue	Isoform 2 of Adenylyl cyclase-associated protein 1 GN=CAP1	sp|Q01518|CAP1_HUMAN	51,830.50	100.00%	4	6	35	0.02680%	30.20%

Biological sample	Protein name	Protein accession numbers	Protein molecular weight (Da)	Protein identification probability	Exclusive unique peptide count	Exclusive unique spectrum count	Total spectrum count	Percentage of total spectra	Percentage sequence coverage
Tumor tissue	Isoform 2 of Adhesion G-protein coupled receptor G1 GN=ADGRG1	sp|Q9Y653|AGRG1_HUMAN	77,073.60	100.00%	2	2	2	0.00153%	4.95%
Tumor tissue	Isoform 2 of ADP-dependent glucokinase GN=ADPGK	sp|Q9BRR6|ADPGK_HUMAN	53,960.40	100.00%	4	5	6	0.00459%	9.68%
Tumor tissue	Isoform 2 of Afadin GN=AFDN	sp|P55196-1|AFAD_HUMAN	205,609.80	100.00%	1	1	10	0.00766%	7.16%
Tumor tissue	Isoform 2 of A-kinase anchor protein 2 GN=AKAP2	sp|Q9Y2D5-4|AKAP2_HUMAN	122,072.40	100.00%	9	10	10	0.00766%	12.20%
Tumor tissue	Isoform 2 of A-kinase anchor protein 8-like GN=AKAP8L	sp|Q9ULX6|AKP8L_HUMAN	65,230.90	100.00%	2	2	2	0.00153%	4.62%
Tumor tissue	Isoform 2 of Aladin GN=AAAS	sp|Q9NRG9|AAAS_HUMAN	59,574.10	100.00%	7	7	7	0.00536%	19.30%
Tumor tissue	Isoform 2 of Allograft inflammatory factor 1-like GN=AIF1L	sp|Q9BQI0|AIF1L_HUMAN	20,016.10	100.00%	2	2	2	0.00153%	13.60%
Pap test	Isoform 2 of Alpha-aminoadipic semialdehyde dehydrogenase GN=ALDH7A1	sp|P49419-2|AL7A1_HUMAN	55,366.80	100.00%	1	1	5	0.00780%	13.30%
Swab	Isoform 2 of Alpha-aminoadipic semialdehyde dehydrogenase GN=ALDH7A1	sp|P49419-2|AL7A1_HUMAN	55,366.80	100.00%	1	2	10	0.00709%	15.30%
Tumor tissue	Isoform 2 of Alpha-aminoadipic semialdehyde dehydrogenase GN=ALDH7A1	sp|P49419-2|AL7A1_HUMAN	55,366.80	100.00%	7	9	25	0.01910%	35.40%
Tumor tissue	Isoform 2 of Alpha-globin transcription factor CP2 GN=TFCP2	sp|Q12800|TFCP2_HUMAN	57,185.60	99.90%	1	1	4	0.00306%	17.30%
Tumor tissue	Isoform 2 of Aminoacyl tRNA synthase complex-interacting multifunctional protein 1 GN=AIMP1	sp|Q12904|AIMP1_HUMAN	37,039.50	100.00%	6	7	14	0.01070%	36.00%
Tumor tissue	Isoform 2 of AMP deaminase 3 GN=AMPD3	sp|Q01432-4|AMPD3_HUMAN	89,731.80	100.00%	3	3	4	0.00306%	4.12%
Pap test	Isoform 2 of Annexin A11 GN=ANXA11	sp|P50995|ANX11_HUMAN	51,244.10	100.00%	10	13	20	0.03120%	25.60%
Swab	Isoform 2 of Annexin A11 GN=ANXA11	sp|P50995|ANX11_HUMAN	51,244.10	100.00%	4	4	5	0.00354%	10.20%

Biological sample	Protein name	Protein accession numbers	Protein molecular weight (Da)	Protein identification probability	Exclusive unique peptide count	Exclusive unique spectrum count	Total spectrum count	Percentage of total spectra	Percentage sequence coverage
Tumor tissue	Isoform 2 of Annexin A11 GN=ANXA11	sp|P50995|ANX11_HUMAN	51,244.10	100.00%	15	21	29	0.02220%	32.60%
Pap test	Isoform 2 of Annexin A2 GN=ANXA2	sp|P07355-2|ANXA2_HUMAN	40,413.40	100.00%	3	5	61	0.09520%	52.90%
Swab	Isoform 2 of Annexin A2 GN=ANXA2	sp|P07355-2|ANXA2_HUMAN	40,413.40	100.00%	1	1	24	0.01700%	31.70%
Tumor tissue	Isoform 2 of Annexin A2 GN=ANXA2	sp|P07355-2|ANXA2_HUMAN	40,413.40	100.00%	4	7	118	0.09030%	52.10%
Pap test	Isoform 2 of Annexin A7 GN=ANXA7	sp|P20073|ANXA7_HUMAN	52,740.50	100.00%	3	3	3	0.00468%	7.51%
Tumor tissue	Isoform 2 of Annexin A7 GN=ANXA7	sp|P20073|ANXA7_HUMAN	52,740.50	100.00%	7	8	9	0.00689%	19.10%
Pap test	Isoform 2 of AP-1 complex subunit gamma-1 GN=AP1G1	sp|O43747|AP1G1_HUMAN	91,354.30	99.50%	1	1	1	0.00156%	1.21%
Swab	Isoform 2 of AP-1 complex subunit gamma-1 GN=AP1G1	sp|O43747|AP1G1_HUMAN	91,725.70	100.00%	2	2	2	0.00142%	2.42%
Tumor tissue	Isoform 2 of AP-1 complex subunit gamma-1 GN=AP1G1	sp|O43747|AP1G1_HUMAN	91,354.30	100.00%	3	3	4	0.00306%	5.70%
Tumor tissue	Isoform 2 of AP-1 complex subunit mu-1 GN=AP1M1	sp|Q9BXS5-2|AP1M1_HUMAN	49,841.20	100.00%	6	8	11	0.00842%	26.40%
Tumor tissue	Isoform 2 of AP-1 complex subunit mu-2 GN=AP1M2	sp|Q9Y6Q5|AP1M2_HUMAN	48,109.30	100.00%	1	1	6	0.00459%	14.10%
Tumor tissue	Isoform 2 of AP-2 complex subunit alpha-2 GN=AP2A2	sp|O94973|AP2A2_HUMAN	103,962.80	100.00%	12	13	25	0.01910%	24.10%
Swab	Isoform 2 of AP-2 complex subunit beta GN=AP2B1	sp|P63010|AP2B1_HUMAN	104,555.30	100.00%	1	1	3	0.00213%	4.21%
Tumor tissue	Isoform 2 of AP-2 complex subunit beta GN=AP2B1	sp|P63010|AP2B1_HUMAN	105,694.60	100.00%	10	14	46	0.03520%	21.60%
Pap test	Isoform 2 of Apoptosis-associated speck-like protein containing a CARD GN=PYCARD	sp|Q9ULZ3-2|ASC_HUMAN	19,970.00	100.00%	2	2	2	0.00312%	13.10%
Swab	Isoform 2 of Apoptosis-associated speck-like protein containing a CARD GN=PYCARD	sp|Q9ULZ3-2|ASC_HUMAN	19,970.00	100.00%	3	3	7	0.00496%	23.30%
Tumor tissue	Isoform 2 of Apoptosis-associated speck-like protein containing a CARD GN=PYCARD	sp|Q9ULZ3-2|ASC_HUMAN	19,970.00	100.00%	3	3	5	0.00383%	29.00%

Biological sample	Protein name	Protein accession numbers	Protein molecular weight (Da)	Protein identification probability	Exclusive unique peptide count	Exclusive unique spectrum count	Total spectrum count	Percentage of total spectra	Percentage sequence coverage
Tumor tissue	Isoform 2 of Arf-GAP domain and FG repeat-containing protein 1 GN=AGFG1	sp|P52594|AGFG1_HUMAN	54,181.30	100.00%	5	5	5	0.00383%	17.80%
Pap test	Isoform 2 of Arginase-1 GN=ARG1	sp|P05089|ARGI1_HUMAN	35,665.20	100.00%	2	2	2	0.00312%	6.97%
Swab	Isoform 2 of Arginase-1 GN=ARG1	sp|P05089|ARGI1_HUMAN	35,665.20	100.00%	2	2	3	0.00213%	8.79%
Tumor tissue	Isoform 2 of Armadillo repeat-containing protein 10 GN=ARMC10	sp|Q8N2F6|ARM10_HUMAN	33,851.10	100.00%	2	2	2	0.00153%	10.70%
Tumor tissue	Isoform 2 of Asparagine synthetase [glutamine-hydrolyzing] GN=ASNS	sp|P08243|ASNS_HUMAN	62,170.20	100.00%	3	3	4	0.00306%	5.56%
Swab	Isoform 2 of Astrocytic phosphoprotein PEA-15 GN=PEA15	sp|Q15121|PEA15_HUMAN	17,307.60	100.00%	2	2	3	0.00213%	23.80%
Tumor tissue	Isoform 2 of Astrocytic phosphoprotein PEA-15 GN=PEA15	sp|Q15121|PEA15_HUMAN	17,307.60	100.00%	3	6	12	0.00919%	37.70%
Tumor tissue	Isoform 2 of ATP synthase-coupling factor 6, mitochondrial GN=ATP5J	sp|P18859|ATP5J_HUMAN	12,587.90	100.00%	1	2	8	0.00612%	42.20%
Tumor tissue	Isoform 2 of ATPase WRNIP1 GN=WRNIP1	sp|Q96S55|WRIP1_HUMAN	69,460.20	100.00%	5	5	5	0.00383%	9.84%
Tumor tissue	Isoform 2 of ATP-binding cassette sub-family F member 2 GN=ABCF2	sp|Q9UG63|ABCF2_HUMAN	71,292.50	100.00%	4	6	6	0.00459%	9.62%
Tumor tissue	Isoform 2 of ATP-binding cassette sub-family F member 3 GN=ABCF3	sp|Q9NUQ8|ABCF3_HUMAN	78,985.70	100.00%	4	5	5	0.00383%	8.53%
Swab	Isoform 2 of ATP-citrate synthase GN=ACLY	sp|P53396-2|ACLY_HUMAN	119,775.60	100.00%	2	2	2	0.00142%	2.38%
Tumor tissue	Isoform 2 of ATP-citrate synthase GN=ACLY	sp|P53396-2|ACLY_HUMAN	119,775.60	100.00%	19	23	35	0.02680%	24.10%
Tumor tissue	Isoform 2 of ATP-dependent 6-phosphofructokinase, muscle type GN=PFKM	sp|P08237-2|PFKAM_HUMAN	81,776.50	100.00%	6	8	8	0.00612%	15.40%
Tumor tissue	Isoform 2 of ATP-dependent RNA helicase DDX54 GN=DDX54	sp|Q8TDD1|DDX54_HUMAN	98,669.00	100.00%	2	2	2	0.00153%	6.69%
Tumor tissue	Isoform 2 of ATP-dependent zinc metalloprotease YME1L1 GN=YME1L1	sp|Q96TA2|YMEL1_HUMAN	75,984.50	100.00%	1	2	4	0.00306%	5.59%
Tumor tissue	Isoform 2 of Band 4.1-like protein 3 GN=EPB41L3	sp|Q9Y2J2-2|E41L3_HUMAN	96,514.40	100.00%	7	7	9	0.00689%	17.10%

Biological sample	Protein name	Protein accession numbers	Protein molecular weight (Da)	Protein identification probability	Exclusive unique peptide count	Exclusive unique spectrum count	Total spectrum count	Percentage of total spectra	Percentage sequence coverage
Tumor tissue	Isoform 2 of Basigin GN=BSG	sp|P35613|BASI_HUMAN	29,221.10	100.00%	1	1	8	0.00612%	27.10%
Pap test	Isoform 2 of B-cell receptor-associated protein 31 GN=BCAP31	sp|P51572|BAP31_HUMAN	34,753.10	99.80%	2	2	2	0.00312%	5.75%
Tumor tissue	Isoform 2 of B-cell receptor-associated protein 31 GN=BCAP31	sp|P51572|BAP31_HUMAN	34,753.10	100.00%	6	11	14	0.01070%	18.80%
Tumor tissue	Isoform 2 of Beta-parvin GN=PARVB	sp|Q9HBI1-2|PARVB_HUMAN	45,183.20	99.80%	1	2	6	0.00459%	9.07%
Tumor tissue	Isoform 2 of BH3-interacting domain death agonist GN=BID	sp|P55957|BID_HUMAN	21,995.30	100.00%	2	2	3	0.00230%	16.20%
Swab	Isoform 2 of Bifunctional coenzyme A synthase GN=COASY	sp|Q13057|COASY_HUMAN	65,340.00	100.00%	1	1	2	0.00142%	1.69%
Tumor tissue	Isoform 2 of Bifunctional coenzyme A synthase GN=COASY	sp|Q13057|COASY_HUMAN	65,340.00	100.00%	4	4	5	0.00383%	11.10%
Pap test	Isoform 2 of Bifunctional purine biosynthesis protein PURH GN=ATIC	sp|P31939|PUR9_HUMAN	64,524.40	99.80%	1	1	1	0.00156%	2.71%
Swab	Isoform 2 of Bifunctional purine biosynthesis protein PURH GN=ATIC	sp|P31939|PUR9_HUMAN	64,616.50	100.00%	5	6	8	0.00567%	10.50%
Tumor tissue	Isoform 2 of Bifunctional purine biosynthesis protein PURH GN=ATIC	sp|P31939|PUR9_HUMAN	64,524.40	100.00%	15	19	22	0.01680%	37.20%
Tumor tissue	Isoform 2 of Bone marrow stromal antigen 2 GN=BST2	sp|Q10589|BST2_HUMAN	19,769.30	100.00%	2	3	3	0.00230%	14.90%
Tumor tissue	Isoform 2 of Brain-specific angiogenesis inhibitor 1-associated protein 2 GN=BAIAP2	sp|Q9UQB8|BAIP2_HUMAN	59,015.40	100.00%	1	1	18	0.01380%	34.10%
Tumor tissue	Isoform 2 of BRO1 domain-containing protein BROX GN=BROX	sp|Q5VW32|BROX_HUMAN	42,873.70	100.00%	3	3	3	0.00230%	13.20%
Pap test	Isoform 2 of Calcium and integrin-binding protein 1 GN=CIB1	sp|Q99828|CIB1_HUMAN	21,704.60	100.00%	2	2	3	0.00468%	12.60%
Tumor tissue	Isoform 2 of Calcium and integrin-binding protein 1 GN=CIB1	sp|Q99828|CIB1_HUMAN	21,704.60	100.00%	1	1	1	0.00077%	7.36%

Biological sample	Protein name	Protein accession numbers	Protein molecular weight (Da)	Protein identification probability	Exclusive unique peptide count	Exclusive unique spectrum count	Total spectrum count	Percentage of total spectra	Percentage sequence coverage
Tumor tissue	Isoform 2 of Calcium-binding mitochondrial carrier protein Aralar2 GN=SLC25A13	sp|Q9UJS0|CMC2_HUMAN	74,305.30	100.00%	10	13	21	0.01610%	27.40%
Pap test	Isoform 2 of Calnexin GN=CANX	sp|P27824|CALX_HUMAN	71,504.80	100.00%	5	5	7	0.01090%	11.50%
Tumor tissue	Isoform 2 of Calnexin GN=CANX	sp|P27824|CALX_HUMAN	67,570.20	100.00%	11	19	46	0.03520%	23.00%
Pap test	Isoform 2 of Calponin-2 GN=CNN2	sp|Q99439-2|CNN2_HUMAN	29,500.90	99.20%	1	1	1	0.00156%	6.30%
Tumor tissue	Isoform 2 of Calponin-2 GN=CNN2	sp|Q99439-2|CNN2_HUMAN	29,500.90	100.00%	2	3	28	0.02140%	27.40%
Tumor tissue	Isoform 2 of Calumenin GN=CALU	sp|O43852-4|CALU_HUMAN	38,080.00	100.00%	1	2	12	0.00919%	40.60%
Pap test	Isoform 2 of CAP-Gly domain-containing linker protein 1 GN=CLIP1	sp|P30622-1|CLIP1_HUMAN	160,993.40	100.00%	1	1	2	0.00312%	1.61%
Tumor tissue	Isoform 2 of Carboxypeptidase Z GN=CPZ	sp|Q66K79|CBPZ_HUMAN	73,657.10	100.00%	2	2	2	0.00153%	3.90%
Tumor tissue	Isoform 2 of Carnitine O-acetyltransferase GN=CRAT	sp|P43155|CACP_HUMAN	68,570.10	100.00%	3	3	3	0.00230%	8.26%
Tumor tissue	Isoform 2 of Cartilage oligomeric matrix protein GN=COMP	sp|P49747|COMP_HUMAN	77,211.80	100.00%	3	3	3	0.00230%	5.54%
Tumor tissue	Isoform 2 of Casein kinase I isoform alpha GN=CSNK1A1	sp|P48729-2|KC1A_HUMAN	41,939.20	100.00%	2	3	7	0.00536%	12.10%
Tumor tissue	Isoform 2 of CCA tRNA nucleotidyltransferase 1, mitochondrial GN=TRNT1	sp|Q96Q11|TRNT1_HUMAN	47,831.20	100.00%	5	5	5	0.00383%	20.00%
Tumor tissue	Isoform 2 of CCR4-NOT transcription complex subunit 1 GN=CNOT1	sp|A5YKK6|CNOT1_HUMAN	266,386.90	100.00%	13	13	13	0.00995%	7.55%
Tumor tissue	Isoform 2 of CD166 antigen GN=ALCAM	sp|Q13740-2|CD166_HUMAN	63,666.20	100.00%	5	5	5	0.00383%	10.40%
Pap test	Isoform 2 of Cdc42-interacting protein 4 GN=TRIP10	sp|Q15642|CIP4_HUMAN	68,353.10	100.00%	2	2	2	0.00312%	6.24%
Swab	Isoform 2 of Cdc42-interacting protein 4 GN=TRIP10	sp|Q15642|CIP4_HUMAN	68,353.10	99.80%	1	1	1	0.00071%	2.39%
Tumor tissue	Isoform 2 of Cdc42-interacting protein 4 GN=TRIP10	sp|Q15642|CIP4_HUMAN	62,592.00	100.00%	3	3	3	0.00230%	6.24%

Biological sample	Protein name	Protein accession numbers	Protein molecular weight (Da)	Protein identification probability	Exclusive unique peptide count	Exclusive unique spectrum count	Total spectrum count	Percentage of total spectra	Percentage sequence coverage
Tumor tissue	Isoform 2 of Cell division cycle and apoptosis regulator protein 1 GN=CCAR1	sp|Q8IX12|CCAR1_HUMAN	132,823.70	100.00%	5	5	9	0.00689%	7.93%
Tumor tissue	Isoform 2 of Chitinase domain-containing protein 1 GN=CHID1	sp|Q9BWS9|CHID1_HUMAN	44,940.90	100.00%	3	5	7	0.00536%	9.33%
Tumor tissue	Isoform 2 of Chromodomain-helicase-DNA-binding protein 2 GN=CHD2	sp|O14647|CHD2_HUMAN	211,349.30	100.00%	1	1	3	0.00230%	1.96%
Tumor tissue	Isoform 2 of C-Jun-amino-terminal kinase-interacting protein 4 GN=SPAG9	sp|O60271-2|JIP4_HUMAN	145,135.20	100.00%	5	6	6	0.00459%	7.93%
Pap test	Isoform 2 of Clathrin heavy chain 1 GN=CLTC	sp|Q00610|CLH1_HUMAN	187,894.80	100.00%	9	11	19	0.02960%	11.70%
Swab	Isoform 2 of Clathrin heavy chain 1 GN=CLTC	sp|Q00610|CLH1_HUMAN	187,894.80	100.00%	7	8	13	0.00921%	9.52%
Tumor tissue	Isoform 2 of Clathrin heavy chain 1 GN=CLTC	sp|Q00610|CLH1_HUMAN	187,894.80	100.00%	29	49	121	0.09260%	29.00%
Tumor tissue	Isoform 2 of Cleavage stimulation factor subunit 2 GN=CSTF2	sp|P33240|CSTF2_HUMAN	59,251.20	100.00%	2	2	3	0.00230%	8.93%
Tumor tissue	Isoform 2 of Cleft lip and palate transmembrane protein 1-like protein GN=CLPTM1L	sp|Q96KA5|CLP1L_HUMAN	62,230.40	100.00%	3	3	3	0.00230%	7.37%
Pap test	Isoform 2 of Clusterin GN=CLU	sp|P10909|CLUS_HUMAN	52,495.00	100.00%	8	12	20	0.03120%	21.20%
Swab	Isoform 2 of Clusterin GN=CLU	sp|P10909|CLUS_HUMAN	57,832.60	100.00%	8	10	26	0.01840%	18.40%
Tumor tissue	Isoform 2 of Clusterin GN=CLU	sp|P10909|CLUS_HUMAN	52,495.00	100.00%	10	14	21	0.01610%	23.00%
Pap test	Isoform 2 of Coatomer subunit alpha GN=COPA	sp|P53621|COPA_HUMAN	138,349.30	100.00%	2	2	2	0.00312%	1.95%
Swab	Isoform 2 of Coatomer subunit alpha GN=COPA	sp|P53621|COPA_HUMAN	138,349.30	100.00%	1	1	1	0.00071%	1.22%
Tumor tissue	Isoform 2 of Coatomer subunit alpha GN=COPA	sp|P53621|COPA_HUMAN	139,327.40	100.00%	25	32	41	0.03140%	26.60%
Swab	Isoform 2 of Coatomer subunit beta' GN=COPB2	sp|P35606|COPB2_HUMAN	102,489.10	100.00%	1	1	1	0.00071%	2.17%
Tumor tissue	Isoform 2 of Coatomer subunit beta' GN=COPB2	sp|P35606|COPB2_HUMAN	99,047.80	100.00%	25	30	37	0.02830%	35.20%

Biological sample	Protein name	Protein accession numbers	Protein molecular weight (Da)	Protein identification probability	Exclusive unique peptide count	Exclusive unique spectrum count	Total spectrum count	Percentage of total spectra	Percentage sequence coverage
Swab	Isoform 2 of Coatomer subunit gamma-2 GN=COPG2	sp|Q9UBF2-2|COPG2_HUMAN	81,485.30	100.00%	1	1	1	0.00071%	2.34%
Tumor tissue	Isoform 2 of Coatomer subunit gamma-2 GN=COPG2	sp|Q9UBF2-2|COPG2_HUMAN	81,485.30	100.00%	5	5	11	0.00842%	10.70%
Tumor tissue	Isoform 2 of Coiled-coil and C2 domain-containing protein 1A GN=CC2D1A	sp|Q6P1N0|C2D1A_HUMAN	104,064.90	100.00%	2	2	2	0.00153%	2.32%
Tumor tissue	Isoform 2 of Coiled-coil domain-containing protein 80 GN=CCDC80	sp|Q76M96|CCD80_HUMAN	109,494.90	100.00%	6	6	6	0.00459%	9.78%
Tumor tissue	Isoform 2 of Coiled-coil domain-containing protein 90B, mitochondrial GN=CCDC90B	sp|Q9GZT6|CC90B_HUMAN	27,979.60	100.00%	2	2	2	0.00153%	11.80%
Tumor tissue	Isoform 2 of Collagen alpha-1(XIV) chain GN=COL14A1	sp|Q05707|COEA1_HUMAN	191,903.60	100.00%	21	34	125	0.09570%	26.60%
Tumor tissue	Isoform 2 of Collagen alpha-1(XVIII) chain GN=COL18A1	sp|P39060-2|COIA1_HUMAN	154,018.40	100.00%	2	3	14	0.01070%	8.89%
Tumor tissue	Isoform 2 of COMM domain-containing protein 9 GN=COMMD9	sp|Q9P000|COMD9_HUMAN	21,819.90	100.00%	3	4	4	0.00306%	21.20%
Tumor tissue	Isoform 2 of Conserved oligomeric Golgi complex subunit 5 GN=COG5	sp|Q9UP83-3|COG5_HUMAN	94,903.90	100.00%	2	2	3	0.00230%	4.07%
Tumor tissue	Isoform 2 of COP9 signalosome complex subunit 2 GN=COPS2	sp|P61201|CSN2_HUMAN	52,407.50	100.00%	7	8	9	0.00689%	18.90%
Swab	Isoform 2 of Core-binding factor subunit beta GN=CBFB	sp|Q13951-2|PEBB_HUMAN	21,991.70	100.00%	2	2	3	0.00213%	15.50%
Tumor tissue	Isoform 2 of Core-binding factor subunit beta GN=CBFB	sp|Q13951-2|PEBB_HUMAN	21,991.70	100.00%	2	3	3	0.00230%	18.20%
Tumor tissue	Isoform 2 of C-terminal-binding protein 1 GN=CTBP1	sp|Q13363|CTBP1_HUMAN	46,404.90	100.00%	2	4	8	0.00612%	13.10%
Tumor tissue	Isoform 2 of C-terminal-binding protein 2 GN=CTBP2	sp|P56545-2|CTBP2_HUMAN	106,188.20	100.00%	3	4	5	0.00383%	5.79%
Pap test	Isoform 2 of Cullin-3 GN=CUL3	sp|Q13618|CUL3_HUMAN	86,235.60	99.90%	1	1	1	0.00156%	1.75%
Swab	Isoform 2 of Cullin-3 GN=CUL3	sp|Q13618|CUL3_HUMAN	86,235.60	99.90%	1	1	2	0.00142%	3.49%
Tumor tissue	Isoform 2 of Cullin-3 GN=CUL3	sp|Q13618|CUL3_HUMAN	86,235.60	100.00%	7	7	12	0.00919%	17.70%
Swab	Isoform 2 of Cyclin-dependent kinase 12 GN=CDK12	sp|Q9NYV4|CDK12_HUMAN	163,232.90	99.10%	1	1	1	0.00071%	1.49%

Biological sample	Protein name	Protein accession numbers	Protein molecular weight (Da)	Protein identification probability	Exclusive unique peptide count	Exclusive unique spectrum count	Total spectrum count	Percentage of total spectra	Percentage sequence coverage
Tumor tissue	Isoform 2 of Cyclin-dependent kinase 12 GN=CDK12	sp|Q9NYV4|CDK12_HUMAN	164,159.10	99.90%	1	1	4	0.00306%	1.55%
Tumor tissue	Isoform 2 of Cyclin-dependent kinase 9 GN=CDK9	sp|P50750-2|CDK9_HUMAN	53,367.00	99.90%	1	1	4	0.00306%	3.68%
Tumor tissue	Isoform 2 of Cysteine and histidine-rich domain-containing protein 1 GN=CHORDC1	sp|Q9UHD1|CHRD1_HUMAN	37,489.70	100.00%	2	2	2	0.00153%	9.58%
Tumor tissue	Isoform 2 of Cytochrome b5 GN=CYB5A	sp|P00167|CYB5_HUMAN	11,268.50	100.00%	3	4	4	0.00306%	49.00%
Tumor tissue	Isoform 2 of Cytochrome P450 2S1 GN=CYP2S1	sp|Q96SQ9|CP2S1_HUMAN	55,818.40	100.00%	2	2	5	0.00383%	8.69%
Tumor tissue	Isoform 2 of Cytokine receptor-like factor 3 GN=CRLF3	sp|Q8IUI8|CRLF3_HUMAN	49,350.20	100.00%	2	2	2	0.00153%	9.36%
Tumor tissue	Isoform 2 of Cytoplasmic dynein 1 light intermediate chain 2 GN=DYNC1LI2	sp|O43237|DC1L2_HUMAN	45,004.80	100.00%	4	4	6	0.00459%	20.50%
Tumor tissue	Isoform 2 of Cytospin-A GN=SPECC1L	sp|Q69YQ0|CYTSA_HUMAN	120,185.20	100.00%	4	4	4	0.00306%	5.29%
Tumor tissue	Isoform 2 of DCC-interacting protein 13-beta GN=APPL2	sp|Q8NEU8|DP13B_HUMAN	69,747.30	100.00%	3	3	3	0.00230%	7.57%
Swab	Isoform 2 of Dedicator of cytokinesis protein 7 GN=DOCK7	sp|Q96N67|DOCK7_HUMAN	241,417.60	99.60%	1	1	1	0.00071%	1.08%
Tumor tissue	Isoform 2 of Dedicator of cytokinesis protein 7 GN=DOCK7	sp|Q96N67|DOCK7_HUMAN	242,565.80	100.00%	6	6	8	0.00612%	5.07%
Tumor tissue	Isoform 2 of Delta-sarcoglycan GN=SGCD	sp|Q92629-2|SGCD_HUMAN	32,203.20	100.00%	4	4	5	0.00383%	17.90%
Swab	Isoform 2 of Diablo homolog, mitochondrial GN=DIABLO	sp|Q9NR28|DBLOH_HUMAN	27,130.70	100.00%	1	1	2	0.00142%	5.38%
Tumor tissue	Isoform 2 of Diablo homolog, mitochondrial GN=DIABLO	sp|Q9NR28|DBLOH_HUMAN	21,232.80	100.00%	4	5	5	0.00383%	23.70%
Tumor tissue	Isoform 2 of Dipeptidyl peptidase 9 GN=DPP9	sp|Q86TI2|DPP9_HUMAN	98,264.20	100.00%	4	4	4	0.00306%	7.51%
Tumor tissue	Isoform 2 of DNA (cytosine-5)-methyltransferase 1 GN=DNMT1	sp|P26358-2|DNMT1_HUMAN	184,823.00	100.00%	5	5	6	0.00459%	3.80%
Pap test	Isoform 2 of DNA dC->dU-editing enzyme APOBEC-3A GN=APOBEC3A	sp|P31941|ABC3A_HUMAN	23,012.30	100.00%	4	5	7	0.01090%	24.10%

Biological sample	Protein name	Protein accession numbers	Protein molecular weight (Da)	Protein identification probability	Exclusive unique peptide count	Exclusive unique spectrum count	Total spectrum count	Percentage of total spectra	Percentage sequence coverage
Swab	Isoform 2 of DNA dC->dU-editing enzyme APOBEC-3A GN=APOBEC3A	sp|P31941|ABC3A_HUMAN	21,777.90	99.80%	1	1	1	0.00071%	5.35%
Tumor tissue	Isoform 2 of DNA repair protein RAD50 GN=RAD50	sp|Q92878|RAD50_HUMAN	154,592.90	100.00%	17	18	22	0.01680%	16.40%
Tumor tissue	Isoform 2 of DNA replication licensing factor MCM3 GN=MCM3	sp|P25205|MCM3_HUMAN	90,982.60	100.00%	12	13	14	0.01070%	18.60%
Tumor tissue	Isoform 2 of DnaJ homolog subfamily A member 3, mitochondrial GN=DNAJA3	sp|Q96EY1|DNJA3_HUMAN	52,489.30	100.00%	3	4	4	0.00306%	6.40%
Tumor tissue	Isoform 2 of DnaJ homolog subfamily C member 7 GN=DNAJC7	sp|Q99615|DNJC7_HUMAN	56,442.90	100.00%	3	3	3	0.00230%	7.53%
Pap test	Isoform 2 of Dolichyl-diphosphooligosaccharide--protein glycosyltransferase subunit 2 GN=RPN2	sp|P04844|RPN2_HUMAN	67,724.30	100.00%	1	1	4	0.00624%	7.64%
Tumor tissue	Isoform 2 of Dolichyl-diphosphooligosaccharide--protein glycosyltransferase subunit 2 GN=RPN2	sp|P04844|RPN2_HUMAN	67,724.30	100.00%	11	18	57	0.04360%	42.90%
Tumor tissue	Isoform 2 of Double-stranded RNA-specific editase 1 GN=ADARB1	sp|P78563|RED1_HUMAN	76,635.20	100.00%	4	4	4	0.00306%	11.40%
Swab	Isoform 2 of Dual specificity mitogen-activated protein kinase kinase 1 GN=MAP2K1	sp|Q02750|MP2K1_HUMAN	43,440.20	100.00%	1	1	2	0.00142%	3.27%
Tumor tissue	Isoform 2 of Dual specificity mitogen-activated protein kinase kinase 1 GN=MAP2K1	sp|Q02750|MP2K1_HUMAN	40,764.90	100.00%	3	5	7	0.00536%	17.40%
Tumor tissue	Isoform 2 of Dymeclin GN=DYM	sp|Q7RTS9|DYM_HUMAN	54,428.00	100.00%	2	2	2	0.00153%	4.18%
Swab	Isoform 2 of Dynactin subunit 2 GN=DCTN2	sp|Q13561|DCTN2_HUMAN	44,474.00	100.00%	1	1	7	0.00496%	19.00%
Tumor tissue	Isoform 2 of Dynactin subunit 2 GN=DCTN2	sp|Q13561|DCTN2_HUMAN	44,820.40	100.00%	2	3	19	0.01450%	38.90%
Tumor tissue	Isoform 2 of Dynamin-like 120 kDa protein, mitochondrial GN=OPA1	sp|O60313|OPA1_HUMAN	115,887.80	100.00%	19	23	24	0.01840%	24.90%

Biological sample	Protein name	Protein accession numbers	Protein molecular weight (Da)	Protein identification probability	Exclusive unique peptide count	Exclusive unique spectrum count	Total spectrum count	Percentage of total spectra	Percentage sequence coverage
Tumor tissue	Isoform 2 of E3 ubiquitin-protein ligase HUWE1 GN=HUWE1	sp|Q7Z6Z7|HUWE1_HUMAN	480,199.20	100.00%	28	33	38	0.02910%	9.57%
Tumor tissue	Isoform 2 of E3 ubiquitin-protein ligase UBR4 GN=UBR4	sp|Q5T4S7|UBR4_HUMAN	573,607.30	100.00%	17	19	20	0.01530%	6.01%
Tumor tissue	Isoform 2 of Ectonucleoside triphosphate diphosphohydrolase 1 GN=ENTPD1	sp|P49961|ENTP1_HUMAN	57,965.80	100.00%	3	3	4	0.00306%	7.93%
Tumor tissue	Isoform 2 of Egl nine homolog 1 GN=EGLN1	sp|Q9GZT9|EGLN1_HUMAN	43,666.60	99.90%	1	2	3	0.00230%	8.66%
Swab	Isoform 2 of Electron transfer flavoprotein subunit beta GN=ETFB	sp|P38117|ETFB_HUMAN	27,843.60	100.00%	5	5	6	0.00425%	13.90%
Tumor tissue	Isoform 2 of Electron transfer flavoprotein subunit beta GN=ETFB	sp|P38117|ETFB_HUMAN	27,843.60	100.00%	8	10	11	0.00842%	24.00%
Pap test	Isoform 2 of Elongation factor 1-gamma GN=EEF1G	sp|P26641|EF1G_HUMAN	50,119.40	100.00%	7	7	9	0.01400%	14.00%
Swab	Isoform 2 of Elongation factor 1-gamma GN=EEF1G	sp|P26641|EF1G_HUMAN	56,150.90	100.00%	4	5	7	0.00496%	9.24%
Tumor tissue	Isoform 2 of Elongation factor 1-gamma GN=EEF1G	sp|P26641|EF1G_HUMAN	50,119.40	100.00%	10	14	29	0.02220%	19.30%
Tumor tissue	Isoform 2 of Elongator complex protein 2 GN=ELP2	sp|Q6IA86|ELP2_HUMAN	92,498.40	100.00%	2	2	2	0.00153%	5.29%
Tumor tissue	Isoform 2 of Elongator complex protein 3 GN=ELP3	sp|Q9H9T3-2|ELP3_HUMAN	60,648.90	100.00%	1	1	2	0.00153%	4.32%
Tumor tissue	Isoform 2 of Endoplasmic reticulum-Golgi intermediate compartment protein 1 GN=ERGIC1	sp|Q969X5|ERGI1_HUMAN	22,160.70	100.00%	5	10	14	0.01070%	41.90%
Tumor tissue	Isoform 2 of Endothelial differentiation-related factor 1 GN=EDF1	sp|O60869|EDF1_HUMAN	15,480.60	100.00%	2	3	3	0.00230%	19.40%
Pap test	Isoform 2 of Enoyl-CoA delta isomerase 1, mitochondrial GN=ECI1	sp|P42126|ECI1_HUMAN	30,896.10	100.00%	3	5	5	0.00780%	13.30%
Tumor tissue	Isoform 2 of Enoyl-CoA delta isomerase 1, mitochondrial GN=ECI1	sp|P42126|ECI1_HUMAN	32,816.50	100.00%	3	4	4	0.00306%	13.30%

Biological sample	Protein name	Protein accession numbers	Protein molecular weight (Da)	Protein identification probability	Exclusive unique peptide count	Exclusive unique spectrum count	Total spectrum count	Percentage of total spectra	Percentage sequence coverage
Swab	Isoform 2 of Epidermal growth factor receptor substrate 15-like 1 GN=EPS15L1	sp|Q9UBC2|EP15R_HUMAN	99,609.50	99.90%	1	1	1	0.00071%	1.54%
Tumor tissue	Isoform 2 of Epidermal growth factor receptor substrate 15-like 1 GN=EPS15L1	sp|Q9UBC2|EP15R_HUMAN	94,258.30	100.00%	4	5	6	0.00459%	7.69%
Tumor tissue	Isoform 2 of Epithelial splicing regulatory protein 1 GN=ESRP1	sp|Q6NXG1|ESRP1_HUMAN	67,665.60	100.00%	5	5	6	0.00459%	13.80%
Tumor tissue	Isoform 2 of Epsin-1 GN=EPN1	sp|Q9Y6I3-1|EPN1_HUMAN	69,040.30	100.00%	2	2	3	0.00230%	8.16%
Tumor tissue	Isoform 2 of ER membrane protein complex subunit 1 GN=EMC1	sp|Q8N766|EMC1_HUMAN	111,761.40	100.00%	14	16	17	0.01300%	21.50%
Swab	Isoform 2 of Eukaryotic initiation factor 4A-II GN=EIF4A2	sp|Q14240-2|IF4A2_HUMAN	46,490.70	100.00%	1	1	12	0.00850%	12.70%
Tumor tissue	Isoform 2 of Eukaryotic initiation factor 4A-II GN=EIF4A2	sp|Q14240-2|IF4A2_HUMAN	46,490.70	100.00%	7	9	25	0.01910%	33.80%
Tumor tissue	Isoform 2 of Eukaryotic peptide chain release factor GTP-binding subunit ERF3A GN=GSPT1	sp|P15170-3|ERF3A_HUMAN	68,700.10	100.00%	6	6	12	0.00919%	20.30%
Pap test	Isoform 2 of Eukaryotic translation initiation factor 3 subunit B GN=EIF3B	sp|P55884|EIF3B_HUMAN	99,030.70	100.00%	4	4	4	0.00624%	5.61%
Swab	Isoform 2 of Eukaryotic translation initiation factor 3 subunit B GN=EIF3B	sp|P55884|EIF3B_HUMAN	99,030.70	99.80%	1	1	1	0.00071%	1.15%
Tumor tissue	Isoform 2 of Eukaryotic translation initiation factor 3 subunit B GN=EIF3B	sp|P55884|EIF3B_HUMAN	92,482.80	100.00%	13	14	18	0.01380%	19.40%
Tumor tissue	Isoform 2 of Eukaryotic translation initiation factor 3 subunit C GN=EIF3C	sp|Q99613-2|EIF3C_HUMAN	104,104.70	100.00%	11	11	13	0.00995%	10.90%
Swab	Isoform 2 of Eukaryotic translation initiation factor 3 subunit D GN=EIF3D	sp|O15371|EIF3D_HUMAN	58,141.40	100.00%	1	1	1	0.00071%	2.00%

Biological sample	Protein name	Protein accession numbers	Protein molecular weight (Da)	Protein identification probability	Exclusive unique peptide count	Exclusive unique spectrum count	Total spectrum count	Percentage of total spectra	Percentage sequence coverage
Tumor tissue	Isoform 2 of Eukaryotic translation initiation factor 3 subunit D GN=EIF3D	sp|O15371|EIF3D_HUMAN	63,973.80	100.00%	2	2	7	0.00536%	9.02%
Tumor tissue	Isoform 2 of Exocyst complex component 1 GN=EXOC1	sp|Q9NV70|EXOC1_HUMAN	100,285.10	100.00%	7	11	11	0.00842%	15.50%
Tumor tissue	Isoform 2 of Exonuclease 3'-5' domain-containing protein 2 GN=EXD2	sp|Q9NVH0|EXD2_HUMAN	56,346.60	100.00%	3	3	3	0.00230%	8.06%
Tumor tissue	Isoform 2 of Extended synaptotagmin-1 GN=ESYT1	sp|Q9BSJ8|ESYT1_HUMAN	122,859.10	100.00%	19	23	30	0.02300%	25.90%
Pap test	Isoform 2 of Extracellular matrix protein 1 GN=ECM1	sp|Q16610-2|ECM1_HUMAN	46,098.90	99.50%	1	1	19	0.02960%	32.00%
Swab	Isoform 2 of F-actin-capping protein subunit beta GN=CAPZB	sp|P47756-2|CAPZB_HUMAN	30,629.70	100.00%	1	1	24	0.01700%	40.40%
Tumor tissue	Isoform 2 of F-actin-capping protein subunit beta GN=CAPZB	sp|P47756-2|CAPZB_HUMAN	30,629.70	100.00%	2	3	19	0.01450%	31.20%
Tumor tissue	Isoform 2 of F-actin-uncapping protein LRRC16A GN=CARMIL1	sp|Q5VZK9|CARL1_HUMAN	147,092.30	100.00%	2	3	3	0.00230%	3.09%
Swab	Isoform 2 of Farnesyl pyrophosphate synthase GN=FDPS	sp|P14324-2|FPPS_HUMAN	40,533.60	100.00%	4	5	5	0.00354%	14.70%
Tumor tissue	Isoform 2 of Farnesyl pyrophosphate synthase GN=FDPS	sp|P14324-2|FPPS_HUMAN	40,533.60	100.00%	5	9	10	0.00766%	18.70%
Tumor tissue	Isoform 2 of Fatty aldehyde dehydrogenase GN=ALDH3A2	sp|P51648|AL3A2_HUMAN	57,671.50	100.00%	6	7	7	0.00536%	15.60%
Tumor tissue	Isoform 2 of F-box/LRR-repeat protein 18 GN=FBXL18	sp|Q96ME1|FXL18_HUMAN	88,342.60	100.00%	2	2	2	0.00153%	5.99%
Pap test	Isoform 2 of Fermitin family homolog 3 GN=FERMT3	sp|Q86UX7|URP2_HUMAN	75,954.90	99.20%	1	1	1	0.00156%	2.41%
Swab	Isoform 2 of Fermitin family homolog 3 GN=FERMT3	sp|Q86UX7|URP2_HUMAN	75,954.90	100.00%	5	5	6	0.00425%	10.70%
Tumor tissue	Isoform 2 of Fermitin family homolog 3 GN=FERMT3	sp|Q86UX7|URP2_HUMAN	75,954.90	100.00%	11	13	16	0.01220%	26.70%
Tumor tissue	Isoform 2 of Ferrochelatase, mitochondrial GN=FECH	sp|P22830|HEMH_HUMAN	48,626.00	100.00%	2	2	4	0.00306%	16.80%
Pap test	Isoform 2 of Fibrinogen alpha chain GN=FGA	sp|P02671|FIBA_HUMAN	94,973.40	100.00%	16	31	81	0.12600%	29.20%

Biological sample	Protein name	Protein accession numbers	Protein molecular weight (Da)	Protein identification probability	Exclusive unique peptide count	Exclusive unique spectrum count	Total spectrum count	Percentage of total spectra	Percentage sequence coverage
Swab	Isoform 2 of Fibrinogen alpha chain GN=FGA	sp|P02671|FIBA_HUMAN	94,973.40	100.00%	16	25	66	0.04680%	30.30%
Tumor tissue	Isoform 2 of Fibrinogen alpha chain GN=FGA	sp|P02671|FIBA_HUMAN	69,756.90	100.00%	22	46	97	0.07430%	36.80%
Tumor tissue	Isoform 2 of Fibronectin type-III domain-containing protein 3A GN=FNDC3A	sp|Q9Y2H6-2|FND3A_HUMAN	131,853.20	100.00%	1	1	2	0.00153%	3.24%
Tumor tissue	Isoform 2 of Fibulin-2 GN=FBLN2	sp|P98095|FBLN2_HUMAN	126,568.80	100.00%	8	11	18	0.01380%	8.77%
Pap test	Isoform 2 of Filamin-A GN=FLNA	sp|P21333|FLNA_HUMAN	280,729.40	100.00%	43	46	53	0.08270%	25.70%
Swab	Isoform 2 of Filamin-A GN=FLNA	sp|P21333|FLNA_HUMAN	280,008.70	100.00%	29	34	44	0.03120%	17.70%
Tumor tissue	Isoform 2 of Filamin-A GN=FLNA	sp|P21333|FLNA_HUMAN	280,008.70	100.00%	92	182	417	0.31900%	47.90%
Pap test	Isoform 2 of Filamin-B GN=FLNB	sp|O75369|FLNB_HUMAN	276,933.40	100.00%	26	27	35	0.05460%	15.80%
Swab	Isoform 2 of Filamin-B GN=FLNB	sp|O75369|FLNB_HUMAN	281,629.70	100.00%	6	6	6	0.00425%	3.34%
Tumor tissue	Isoform 2 of Filamin-B GN=FLNB	sp|O75369|FLNB_HUMAN	281,629.70	100.00%	54	67	109	0.08340%	30.90%
Tumor tissue	Isoform 2 of Filamin-C GN=FLNC	sp|Q14315|FLNC_HUMAN	287,274.00	100.00%	51	64	106	0.08110%	28.50%
Tumor tissue	Isoform 2 of Formin-like protein 1 GN=FMNL1	sp|O95466|FMNL1_HUMAN	121,858.20	100.00%	2	2	4	0.00306%	5.34%
Pap test	Isoform 2 of Fructose-bisphosphate aldolase A GN=ALDOA	sp|P04075-2|ALDOA_HUMAN	45,261.20	100.00%	5	9	55	0.08580%	51.90%
Swab	Isoform 2 of Fructose-bisphosphate aldolase A GN=ALDOA	sp|P04075-2|ALDOA_HUMAN	45,261.20	100.00%	6	9	91	0.06450%	48.10%
Tumor tissue	Isoform 2 of Fructose-bisphosphate aldolase A GN=ALDOA	sp|P04075-2|ALDOA_HUMAN	45,261.20	100.00%	5	14	64	0.04900%	35.40%
Swab	Isoform 2 of Galectin-9 GN=LGALS9	sp|O00182|LEG9_HUMAN	38,320.30	99.40%	1	1	1	0.00071%	4.33%
Tumor tissue	Isoform 2 of Galectin-9 GN=LGALS9	sp|O00182|LEG9_HUMAN	35,887.60	100.00%	4	4	4	0.00306%	15.80%
Tumor tissue	Isoform 2 of Gamma-interferon-inducible protein 16 GN=IFI16	sp|Q16666|IF16_HUMAN	82,097.90	100.00%	10	12	21	0.01610%	24.10%
Tumor tissue	Isoform 2 of GEM-interacting protein GN=GMIP	sp|Q9P107|GMIP_HUMAN	106,684.10	100.00%	2	2	2	0.00153%	3.60%
Tumor tissue	Isoform 2 of General transcription factor II-I GN=GTF2I	sp|P78347|GTF2I_HUMAN	110,281.50	100.00%	20	26	27	0.02070%	23.70%

Biological sample	Protein name	Protein accession numbers	Protein molecular weight (Da)	Protein identification probability	Exclusive unique peptide count	Exclusive unique spectrum count	Total spectrum count	Percentage of total spectra	Percentage sequence coverage
Pap test	Isoform 2 of General vesicular transport factor p115 GN=USO1	sp|O60763|USO1_HUMAN	109,197.00	100.00%	2	2	2	0.00312%	2.16%
Swab	Isoform 2 of General vesicular transport factor p115 GN=USO1	sp|O60763|USO1_HUMAN	109,197.00	100.00%	4	4	5	0.00354%	5.45%
Tumor tissue	Isoform 2 of General vesicular transport factor p115 GN=USO1	sp|O60763|USO1_HUMAN	109,197.00	100.00%	18	26	35	0.02680%	25.80%
Pap test	Isoform 2 of Glycerophosphodiester phosphodiesterase domain-containing protein 3 GN=GDPD3	sp|Q7L5L3|GDPD3_HUMAN	29,625.00	100.00%	3	3	3	0.00468%	14.80%
Swab	Isoform 2 of Glycogen [starch] synthase, muscle GN=GYS1	sp|P13807|GYS1_HUMAN	83,787.10	100.00%	1	1	1	0.00071%	2.97%
Tumor tissue	Isoform 2 of Glycogen [starch] synthase, muscle GN=GYS1	sp|P13807|GYS1_HUMAN	83,787.10	100.00%	2	2	2	0.00153%	3.71%
Pap test	Isoform 2 of Glycogen phosphorylase, liver form GN=PYGL	sp|P06737|PYGL_HUMAN	97,152.40	100.00%	10	12	17	0.02650%	15.70%
Swab	Isoform 2 of Glycogen phosphorylase, liver form GN=PYGL	sp|P06737|PYGL_HUMAN	97,152.40	100.00%	17	21	31	0.02200%	23.20%
Tumor tissue	Isoform 2 of Glycogen phosphorylase, liver form GN=PYGL	sp|P06737|PYGL_HUMAN	97,152.40	100.00%	17	24	33	0.02530%	30.50%
Tumor tissue	Isoform 2 of Glycogen phosphorylase, muscle form GN=PYGM	sp|P11217-2|PYGM_HUMAN	87,319.00	99.80%	1	2	10	0.00766%	8.89%
Tumor tissue	Isoform 2 of Glycogen synthase kinase-3 beta GN=GSK3B	sp|P49841|GSK3B_HUMAN	48,034.60	100.00%	1	1	2	0.00153%	6.00%
Tumor tissue	Isoform 2 of Golgi apparatus protein 1 GN=GLG1	sp|Q92896|GSLG1_HUMAN	134,553.90	100.00%	4	4	6	0.00459%	4.41%
Tumor tissue	Isoform 2 of Golgi to ER traffic protein 4 homolog GN=GET4	sp|Q7L5D6|GET4_HUMAN	36,504.80	99.70%	1	1	2	0.00153%	8.03%
Tumor tissue	Isoform 2 of Golgin subfamily A member 5 GN=GOLGA5	sp|Q8TBA6|GOGA5_HUMAN	83,026.00	100.00%	4	5	5	0.00383%	7.81%
Tumor tissue	Isoform 2 of Golgi-specific brefeldin A-resistance guanine nucleotide exchange factor 1 GN=GBF1	sp|Q92538|GBF1_HUMAN	206,021.30	100.00%	8	9	10	0.00766%	6.25%

Biological sample	Protein name	Protein accession numbers	Protein molecular weight (Da)	Protein identification probability	Exclusive unique peptide count	Exclusive unique spectrum count	Total spectrum count	Percentage of total spectra	Percentage sequence coverage
Tumor tissue	Isoform 2 of GPI transamidase component PIG-S GN=PIGS	sp|Q96S52|PIGS_HUMAN	61,657.50	100.00%	5	7	7	0.00536%	14.30%
Tumor tissue	Isoform 2 of GPI transamidase component PIG-T GN=PIGT	sp|Q969N2|PIGT_HUMAN	65,701.30	99.90%	1	1	2	0.00153%	8.59%
Pap test	Isoform 2 of Guanine nucleotide-binding protein G(I)/G(S)/G(T) subunit beta-1 GN=GNB1	sp|P62873|GBB1_HUMAN	37,377.50	100.00%	1	1	6	0.00936%	16.90%
Tumor tissue	Isoform 2 of Guanine nucleotide-binding protein-like 3 GN=GNL3	sp|Q9BVP2|GNL3_HUMAN	61,994.70	100.00%	4	4	5	0.00383%	11.40%
Pap test	Isoform 2 of Haptoglobin GN=HP	sp|P00738-2|HPT_HUMAN	38,451.40	100.00%	1	5	189	0.29500%	52.70%
Swab	Isoform 2 of Haptoglobin GN=HP	sp|P00738-2|HPT_HUMAN	38,451.40	100.00%	1	5	130	0.09210%	42.70%
Tumor tissue	Isoform 2 of Haptoglobin GN=HP	sp|P00738-2|HPT_HUMAN	38,451.40	100.00%	1	3	17	0.01300%	21.30%
Pap test	Isoform 2 of Haptoglobin-related protein GN=HPR	sp|P00739|HPTR_HUMAN	43,076.90	100.00%	1	1	60	0.09360%	24.40%
Swab	Isoform 2 of Haptoglobin-related protein GN=HPR	sp|P00739|HPTR_HUMAN	39,029.50	100.00%	1	1	95	0.06730%	21.30%
Tumor tissue	Isoform 2 of Heat shock protein 75 kDa, mitochondrial GN=TRAP1	sp|Q12906-7|ILF3_HUMAN	74,270.10	100.00%	7	7	18	0.01380%	26.90%
Pap test	Isoform 2 of Heat shock protein HSP 90-alpha GN=HSP90AA1	sp|P07900-2|HS90A_HUMAN	98,165.10	100.00%	6	6	18	0.02810%	14.50%
Swab	Isoform 2 of Heat shock protein HSP 90-alpha GN=HSP90AA1	sp|P07900-2|HS90A_HUMAN	98,165.10	100.00%	13	18	63	0.04460%	28.30%
Tumor tissue	Isoform 2 of Heat shock protein HSP 90-alpha GN=HSP90AA1	sp|P07900-2|HS90A_HUMAN	98,165.10	100.00%	16	34	121	0.09260%	29.20%
Tumor tissue	Isoform 2 of Hepatoma-derived growth factor-related protein 2 GN=HDGFRP2	sp|Q7Z4V5|HDGR2_HUMAN	74,318.30	100.00%	2	2	3	0.00230%	7.91%
Tumor tissue	Isoform 2 of Heterogeneous nuclear ribonucleoprotein A3 GN=HNRNPA3	sp|P51991-2|ROA3_HUMAN	37,029.20	100.00%	1	2	29	0.02220%	32.60%
Pap test	Isoform 2 of Heterogeneous nuclear ribonucleoprotein H3 GN=HNRNPH3	sp|P31942|HNRH3_HUMAN	36,927.60	99.20%	1	2	3	0.00468%	5.14%

Biological sample	Protein name	Protein accession numbers	Protein molecular weight (Da)	Protein identification probability	Exclusive unique peptide count	Exclusive unique spectrum count	Total spectrum count	Percentage of total spectra	Percentage sequence coverage
Tumor tissue	Isoform 2 of Heterogeneous nuclear ribonucleoprotein H3 GN=HNRNPH3	sp|P31942|HNRH3_HUMAN	35,239.70	100.00%	7	16	23	0.01760%	34.10%
Pap test	Isoform 2 of Heterogeneous nuclear ribonucleoprotein Q GN=SYNCRIP	sp|O60506|HNRPQ_HUMAN	69,603.50	100.00%	2	2	2	0.00312%	4.42%
Tumor tissue	Isoform 2 of Heterogeneous nuclear ribonucleoprotein Q GN=SYNCRIP	sp|O60506|HNRPQ_HUMAN	69,603.50	100.00%	17	27	35	0.02680%	35.00%
Tumor tissue	Isoform 2 of Heterogeneous nuclear ribonucleoprotein R GN=HNRNPR	sp|O43390-2|HNRPR_HUMAN	71,215.20	100.00%	1	1	26	0.01990%	24.10%
Pap test	Isoform 2 of Hexokinase-1 GN=HK1	sp|P19367|HXK1_HUMAN	102,488.40	100.00%	1	1	1	0.00156%	1.20%
Swab	Isoform 2 of Hexokinase-1 GN=HK1	sp|P19367|HXK1_HUMAN	102,488.40	100.00%	3	3	5	0.00354%	4.91%
Tumor tissue	Isoform 2 of Hexokinase-1 GN=HK1	sp|P19367|HXK1_HUMAN	102,488.40	100.00%	19	24	37	0.02830%	28.10%
Tumor tissue	Isoform 2 of Histone deacetylase 2 GN=HDAC2	sp|Q92769|HDAC2_HUMAN	55,365.50	100.00%	4	4	9	0.00689%	21.60%
Pap test	Isoform 2 of Histone H1.0 GN=H1F0	sp|P07305|H10_HUMAN	20,864.10	99.90%	1	1	1	0.00156%	5.08%
Tumor tissue	Isoform 2 of Histone H1.0 GN=H1F0	sp|P07305|H10_HUMAN	19,168.20	100.00%	2	5	6	0.00459%	13.00%
Tumor tissue	Isoform 2 of HLA class II histocompatibility antigen gamma chain GN=CD74	sp|P04233-2|HG2A_HUMAN	26,399.40	100.00%	2	3	9	0.00689%	39.20%
Tumor tissue	Isoform 2 of Importin-4 GN=IPO4	sp|Q8TEX9|IPO4_HUMAN	118,718.70	100.00%	2	2	6	0.00459%	9.05%
Pap test	Isoform 2 of Inorganic pyrophosphatase 2, mitochondrial GN=PPA2	sp|Q9H2U2|IPYR2_HUMAN	37,921.40	100.00%	1	1	3	0.00468%	6.30%
Swab	Isoform 2 of Inorganic pyrophosphatase 2, mitochondrial GN=PPA2	sp|Q9H2U2|IPYR2_HUMAN	37,921.40	100.00%	1	1	2	0.00142%	6.30%
Tumor tissue	Isoform 2 of Inorganic pyrophosphatase 2, mitochondrial GN=PPA2	sp|Q9H2U2|IPYR2_HUMAN	39,639.50	100.00%	7	8	12	0.00919%	25.50%
Tumor tissue	Isoform 2 of Inositol 1,4,5-trisphosphate receptor type 1 GN=ITPR1	sp|Q14643|ITPR1_HUMAN	313,931.70	100.00%	3	3	4	0.00306%	2.59%

Biological sample	Protein name	Protein accession numbers	Protein molecular weight (Da)	Protein identification probability	Exclusive unique peptide count	Exclusive unique spectrum count	Total spectrum count	Percentage of total spectra	Percentage sequence coverage
Pap test	Isoform 2 of Inositol-3-phosphate synthase 1 GN=ISYNA1	sp|Q9NPH2|INO1_HUMAN	47,146.50	99.20%	1	1	1	0.00156%	2.56%
Tumor tissue	Isoform 2 of Inositol-3-phosphate synthase 1 GN=ISYNA1	sp|Q9NPH2|INO1_HUMAN	55,136.10	100.00%	3	4	5	0.00383%	9.30%
Tumor tissue	Isoform 2 of Insulin-like growth factor-binding protein 7 GN=IGFBP7	sp|Q16270|IBP7_HUMAN	28,859.50	100.00%	3	3	3	0.00230%	15.40%
Tumor tissue	Isoform 2 of Integrin alpha-3 GN=ITGA3	sp|P26006-1|ITA3_HUMAN	118,757.70	100.00%	3	3	3	0.00230%	2.72%
Pap test	Isoform 2 of Integrin alpha-M GN=ITGAM	sp|P11215|ITAM_HUMAN	127,307.40	100.00%	7	7	7	0.01090%	7.46%
Swab	Isoform 2 of Integrin alpha-M GN=ITGAM	sp|P11215|ITAM_HUMAN	127,307.40	100.00%	6	6	6	0.00425%	8.24%
Tumor tissue	Isoform 2 of Integrin alpha-M GN=ITGAM	sp|P11215|ITAM_HUMAN	127,179.30	100.00%	7	8	12	0.00919%	8.07%
Pap test	Isoform 2 of Inter-alpha-trypsin inhibitor heavy chain H3 GN=ITIH3	sp|Q06033|ITIH3_HUMAN	99,850.90	99.90%	1	1	1	0.00156%	1.13%
Tumor tissue	Isoform 2 of Inter-alpha-trypsin inhibitor heavy chain H3 GN=ITIH3	sp|Q06033|ITIH3_HUMAN	99,850.90	100.00%	5	7	7	0.00536%	9.48%
Tumor tissue	Isoform 2 of Interferon regulatory factor 5 GN=IRF5	sp|Q13568|IRF5_HUMAN	47,035.20	100.00%	2	2	2	0.00153%	4.47%
Swab	Isoform 2 of Interferon-induced 35 kDa protein GN=IFI35	sp|P80217|IN35_HUMAN	31,776.50	98.70%	1	1	1	0.00071%	3.82%
Tumor tissue	Isoform 2 of Interferon-induced 35 kDa protein GN=IFI35	sp|P80217|IN35_HUMAN	31,776.50	100.00%	3	3	3	0.00230%	11.10%
Tumor tissue	Isoform 2 of Interferon-induced protein with tetratricopeptide repeats 1 GN=IFIT1	sp|P09914|IFIT1_HUMAN	51,715.40	100.00%	6	6	6	0.00459%	19.20%
Tumor tissue	Isoform 2 of Interferon-inducible double-stranded RNA-dependent protein kinase activator A GN=PRKRA	sp|O75569|PRKRA_HUMAN	34,405.20	100.00%	4	5	6	0.00459%	19.50%
Swab	Isoform 2 of Interleukin-1 receptor antagonist protein GN=IL1RN	sp|P18510|IL1RA_HUMAN	17,888.50	100.00%	3	4	8	0.00567%	22.00%
Tumor tissue	Isoform 2 of Inverted formin-2 GN=INF2	sp|Q27J81|INF2_HUMAN	134,620.40	100.00%	11	13	13	0.00995%	13.10%

Biological sample	Protein name	Protein accession numbers	Protein molecular weight (Da)	Protein identification probability	Exclusive unique peptide count	Exclusive unique spectrum count	Total spectrum count	Percentage of total spectra	Percentage sequence coverage
Tumor tissue	Isoform 2 of Isopentenyl-diphosphate Delta-isomerase 1 GN=IDI1	sp|Q13907|IDI1_HUMAN	26,320.30	100.00%	2	2	3	0.00230%	8.45%
Tumor tissue	Isoform 2 of Kelch-like protein 22 GN=KLHL22	sp|Q53GT1|KLH22_HUMAN	55,567.30	100.00%	2	2	2	0.00153%	4.28%
Pap test	Isoform 2 of Keratin, type II cytoskeletal 8 GN=KRT8	sp|P05787|K2C8_HUMAN	56,610.30	100.00%	4	7	30	0.04680%	24.50%
Swab	Isoform 2 of Keratin, type II cytoskeletal 8 GN=KRT8	sp|P05787|K2C8_HUMAN	56,610.30	100.00%	1	1	3	0.00213%	4.89%
Tumor tissue	Isoform 2 of Keratin, type II cytoskeletal 8 GN=KRT8	sp|P05787|K2C8_HUMAN	56,610.30	100.00%	10	17	97	0.07430%	46.40%
Pap test	Isoform 2 of Keratin, type II cytoskeletal 80 GN=KRT80	sp|Q6KB66|K2C80_HUMAN	47,242.00	100.00%	2	2	6	0.00936%	9.00%
Tumor tissue	Isoform 2 of Keratin, type II cytoskeletal 80 GN=KRT80	sp|Q6KB66|K2C80_HUMAN	50,525.70	100.00%	2	2	3	0.00230%	9.00%
Tumor tissue	Isoform 2 of KH domain-containing, RNA-binding, signal transduction-associated protein 3 GN=KHDRBS3	sp|O75525|KHDR3_HUMAN	30,250.50	100.00%	2	2	3	0.00230%	11.80%
Swab	Isoform 2 of Lambda-crystallin homolog GN=CRYL1	sp|Q9Y2S2|CRYL1_HUMAN	35,419.30	100.00%	1	1	1	0.00071%	3.70%
Tumor tissue	Isoform 2 of Lambda-crystallin homolog GN=CRYL1	sp|Q9Y2S2|CRYL1_HUMAN	33,359.10	100.00%	4	7	7	0.00536%	15.80%
Tumor tissue	Isoform 2 of L-amino-acid oxidase GN=IL4I1	sp|Q96RQ9|OXLA_HUMAN	62,881.30	100.00%	8	10	20	0.01530%	16.60%
Tumor tissue	Isoform 2 of Lanosterol synthase GN=LSS	sp|P48449|ERG7_HUMAN	82,199.80	100.00%	3	3	3	0.00230%	6.60%
Tumor tissue	Isoform 2 of Leiomodin-1 GN=LMOD1	sp|P29536|LMOD1_HUMAN	67,032.30	100.00%	5	5	7	0.00536%	14.00%
Pap test	Isoform 2 of Leucine-rich repeat flightless-interacting protein 1 GN=LRRFIP1	sp|Q32MZ4-3|LRRF1_HUMAN	86,403.20	100.00%	3	3	3	0.00468%	6.12%
Swab	Isoform 2 of Leucine-rich repeat flightless-interacting protein 1 GN=LRRFIP1	sp|Q32MZ4-3|LRRF1_HUMAN	86,403.20	100.00%	2	2	6	0.00425%	9.82%

Biological sample	Protein name	Protein accession numbers	Protein molecular weight (Da)	Protein identification probability	Exclusive unique peptide count	Exclusive unique spectrum count	Total spectrum count	Percentage of total spectra	Percentage sequence coverage
Tumor tissue	Isoform 2 of Leucine-rich repeat flightless-interacting protein 1 GN=LRRFIP1	sp|Q32MZ4-3|LRRF1_HUMAN	82,688.20	100.00%	2	2	4	0.00306%	7.27%
Tumor tissue	Isoform 2 of Leucine-rich repeat protein SHOC-2 GN=SHOC2	sp|Q9UQ13|SHOC2_HUMAN	64,892.50	100.00%	3	3	3	0.00230%	8.02%
Tumor tissue	Isoform 2 of Leucine-rich repeat-containing protein 15 GN=LRRC15	sp|Q8TF66-2|LRC15_HUMAN	65,024.10	100.00%	3	3	4	0.00306%	4.94%
Swab	Isoform 2 of Leucine-rich repeats and immunoglobulin-like domains protein 3 GN=LRIG3	sp|Q6UXM1|LRIG3_HUMAN	117,441.60	100.00%	2	2	2	0.00142%	4.15%
Tumor tissue	Isoform 2 of Leucine--tRNA ligase, cytoplasmic GN=LARS	sp|Q9P2J5|SYLC_HUMAN	134,470.80	100.00%	9	10	11	0.00842%	11.20%
Tumor tissue	Isoform 2 of Leucyl-cystinyl aminopeptidase GN=LNPEP	sp|Q9UIQ6|LCAP_HUMAN	115,640.20	100.00%	5	5	6	0.00459%	6.53%
Swab	Isoform 2 of Leukotriene A-4 hydrolase GN=LTA4H	sp|P09960-2|LKHA4_HUMAN	59,734.40	100.00%	1	3	38	0.02690%	26.90%
Tumor tissue	Isoform 2 of Leukotriene A-4 hydrolase GN=LTA4H	sp|P09960-2|LKHA4_HUMAN	59,734.40	100.00%	1	2	19	0.01450%	32.00%
Tumor tissue	Isoform 2 of Leupaxin GN=LPXN	sp|O60711|LPXN_HUMAN	43,749.60	100.00%	3	3	3	0.00230%	13.60%
Tumor tissue	Isoform 2 of L-fucose kinase GN=FUK	sp|Q8N0W3-2|FUK_HUMAN	118,392.40	100.00%	2	2	3	0.00230%	2.66%
Tumor tissue	Isoform 2 of Lipase maturation factor 2 GN=LMF2	sp|Q9BU23|LMF2_HUMAN	67,158.00	100.00%	2	2	2	0.00153%	3.52%
Tumor tissue	Isoform 2 of Lipopolysaccharide-responsive and beige-like anchor protein GN=LRBA	sp|P50851-2|LRBA_HUMAN	317,703.80	100.00%	7	8	11	0.00842%	4.56%
Tumor tissue	Isoform 2 of Lon protease homolog, mitochondrial GN=LONP1	sp|P36776|LONM_HUMAN	106,491.30	100.00%	7	7	12	0.00919%	15.50%
Swab	Isoform 2 of L-selectin GN=SELL	sp|P14151|LYAM1_HUMAN	43,618.40	99.90%	2	3	3	0.00213%	7.53%
Tumor tissue	Isoform 2 of Lysine-specific histone demethylase 1A GN=KDM1A	sp|O60341|KDM1A_HUMAN	92,903.40	100.00%	5	6	12	0.00919%	13.80%
Swab	Isoform 2 of Lysophospholipase-like protein 1 GN=LYPLAL1	sp|Q5VWZ2|LYPL1_HUMAN	26,317.00	99.30%	1	1	2	0.00142%	4.07%
Tumor tissue	Isoform 2 of Lysophospholipase-like protein 1 GN=LYPLAL1	sp|Q5VWZ2|LYPL1_HUMAN	24,476.90	100.00%	4	6	6	0.00459%	28.10%

Biological sample	Protein name	Protein accession numbers	Protein molecular weight (Da)	Protein identification probability	Exclusive unique peptide count	Exclusive unique spectrum count	Total spectrum count	Percentage of total spectra	Percentage sequence coverage
Swab	Isoform 2 of Macrophage receptor MARCO GN=MARCO	sp|Q9UEW3|MARCO_HUMAN	43,864.90	100.00%	2	2	3	0.00213%	8.14%
Tumor tissue	Isoform 2 of Malignant T-cell-amplified sequence 1 GN=MCTS1	sp|Q9ULC4|MCTS1_HUMAN	19,229.30	100.00%	5	7	7	0.00536%	35.50%
Swab	Isoform 2 of Mannose-1-phosphate guanyltransferase beta GN=GMPPB	sp|Q9Y5P6|GMPPB_HUMAN	39,834.00	100.00%	3	4	4	0.00283%	10.30%
Tumor tissue	Isoform 2 of Mannose-1-phosphate guanyltransferase beta GN=GMPPB	sp|Q9Y5P6|GMPPB_HUMAN	39,834.00	100.00%	3	5	5	0.00383%	11.40%
Tumor tissue	Isoform 2 of Mannosyl-oligosaccharide glucosidase GN=MOGS	sp|Q13724|MOGS_HUMAN	91,919.10	100.00%	14	18	26	0.01990%	24.10%
Tumor tissue	Isoform 2 of MAP kinase-activated protein kinase 2 GN=MAPKAPK2	sp|P49137|MAPK2_HUMAN	42,203.60	100.00%	2	3	3	0.00230%	7.84%
Tumor tissue	Isoform 2 of Matrilin-2 GN=MATN2	sp|O00339|MATN2_HUMAN	102,231.10	100.00%	2	2	6	0.00459%	7.26%
Tumor tissue	Isoform 2 of Melanoma inhibitory activity protein 3 GN=MIA3	sp|Q5JRA6-2|MIA3_HUMAN	206,892.00	100.00%	6	6	6	0.00459%	4.82%
Tumor tissue	Isoform 2 of Membrane magnesium transporter 1 GN=MMGT1	sp|Q8N4V1|MMGT1_HUMAN	21,882.30	100.00%	3	3	3	0.00230%	22.40%
Tumor tissue	Isoform 2 of Membrane-associated progesterone receptor component 2 GN=PGRMC2	sp|O15173|PGRC2_HUMAN	26,170.90	100.00%	2	2	2	0.00153%	13.40%
Tumor tissue	Isoform 2 of Methionine synthase GN=MTR	sp|Q99707|METH_HUMAN	134,798.30	100.00%	1	1	2	0.00153%	2.06%
Pap test	Isoform 2 of Methylcrotonoyl-CoA carboxylase beta chain, mitochondrial GN=MCCC2	sp|Q9HCC0|MCCB_HUMAN	57,520.10	99.20%	1	1	1	0.00156%	2.48%
Tumor tissue	Isoform 2 of Methylcrotonoyl-CoA carboxylase beta chain, mitochondrial GN=MCCC2	sp|Q9HCC0|MCCB_HUMAN	57,520.10	100.00%	5	5	5	0.00383%	15.20%
Tumor tissue	Isoform 2 of Methylthioribose-1-phosphate isomerase GN=MRI1	sp|Q9BV20|MTNA_HUMAN	39,149.80	100.00%	4	6	6	0.00459%	17.70%
Tumor tissue	Isoform 2 of MICOS complex subunit MIC26 GN=APOO	sp|Q9BUR5|MIC26_HUMAN	22,285.30	100.00%	2	2	2	0.00153%	13.90%
Tumor tissue	Isoform 2 of MICOS complex subunit MIC60 GN=IMMT	sp|Q16891-2|MIC60_HUMAN	82,625.80	100.00%	2	3	39	0.02990%	44.00%

Biological sample	Protein name	Protein accession numbers	Protein molecular weight (Da)	Protein identification probability	Exclusive unique peptide count	Exclusive unique spectrum count	Total spectrum count	Percentage of total spectra	Percentage sequence coverage
Tumor tissue	Isoform 2 of Microtubule-associated protein 1S GN=MAP1S	sp|Q66K74|MAP1S_HUMAN	112,212.80	100.00%	9	10	10	0.00766%	12.30%
Tumor tissue	Isoform 2 of Mini-chromosome maintenance complex-binding protein GN=MCMBP	sp|Q9BTE3-2|MCMBP_HUMAN	72,750.00	100.00%	4	4	4	0.00306%	9.69%
Tumor tissue	Isoform 2 of Mitochondrial import inner membrane translocase subunit TIM50 GN=TIMM50	sp|Q3ZCQ8|TIM50_HUMAN	50,466.10	100.00%	4	5	5	0.00383%	10.70%
Tumor tissue	Isoform 2 of Mitochondrial intermembrane space import and assembly protein 40 GN=CHCHD4	sp|Q8N4Q1|MIA40_HUMAN	15,996.30	100.00%	2	2	2	0.00153%	27.10%
Tumor tissue	Isoform 2 of Mitotic checkpoint protein BUB3 GN=BUB3	sp|O43684|BUB3_HUMAN	36,954.50	100.00%	6	7	8	0.00612%	23.90%
Swab	Isoform 2 of MOB kinase activator 1B GN=MOB1B	sp|Q7L9L4|MOB1B_HUMAN	25,499.80	99.30%	1	1	1	0.00071%	4.98%
Tumor tissue	Isoform 2 of MOB kinase activator 1B GN=MOB1B	sp|Q7L9L4|MOB1B_HUMAN	25,091.70	100.00%	2	2	3	0.00230%	10.40%
Tumor tissue	Isoform 2 of Monoacylglycerol lipase ABHD12 GN=ABHD12	sp|Q8N2K0|ABD12_HUMAN	45,559.60	100.00%	8	9	9	0.00689%	24.80%
Tumor tissue	Isoform 2 of Mothers against decapentaplegic homolog 3 GN=SMAD3	sp|P84022|SMAD3_HUMAN	48,080.50	99.80%	1	1	2	0.00153%	6.30%
Tumor tissue	Isoform 2 of mRNA cap guanine-N7 methyltransferase GN=RNMT	sp|O43148|MCES_HUMAN	54,845.60	100.00%	6	6	6	0.00459%	19.40%
Tumor tissue	Isoform 2 of Multidrug resistance-associated protein 4 GN=ABCC4	sp|O15439|MRP4_HUMAN	144,206.20	100.00%	2	2	2	0.00153%	2.82%
Pap test	Isoform 2 of Multifunctional protein ADE2 GN=PAICS	sp|P22234|PUR6_HUMAN	47,080.00	99.90%	1	1	1	0.00156%	3.24%
Swab	Isoform 2 of Multifunctional protein ADE2 GN=PAICS	sp|P22234|PUR6_HUMAN	47,959.00	100.00%	2	2	2	0.00142%	6.94%
Tumor tissue	Isoform 2 of Multifunctional protein ADE2 GN=PAICS	sp|P22234|PUR6_HUMAN	47,080.00	100.00%	9	10	11	0.00842%	21.80%
Tumor tissue	Isoform 2 of Myb-binding protein 1A GN=MYBBP1A	sp|Q9BQG0|MBB1A_HUMAN	149,371.00	100.00%	12	13	15	0.01150%	9.91%
Tumor tissue	Isoform 2 of Myosin phosphatase Rho-interacting protein GN=MPRIP	sp|Q6WCQ1|MPRIP_HUMAN	118,105.80	100.00%	2	2	14	0.01070%	18.20%

Biological sample	Protein name	Protein accession numbers	Protein molecular weight (Da)	Protein identification probability	Exclusive unique peptide count	Exclusive unique spectrum count	Total spectrum count	Percentage of total spectra	Percentage sequence coverage
Tumor tissue	Isoform 2 of Myosin-11 GN=MYH11	sp|P35749-2|MYH11_HUMAN	228,095.40	100.00%	1	1	368	0.28200%	48.90%
Tumor tissue	Isoform 2 of N-acetylglucosamine-6-phosphate deacetylase GN=AMDHD2	sp|Q9Y303|NAGA_HUMAN	63,594.60	99.90%	1	1	2	0.00153%	9.79%
Pap test	Isoform 2 of N-acetylmuramoyl-L-alanine amidase GN=PGLYRP2	sp|Q96PD5|PGRP2_HUMAN	62,217.90	100.00%	4	6	7	0.01090%	10.30%
Swab	Isoform 2 of N-acetylmuramoyl-L-alanine amidase GN=PGLYRP2	sp|Q96PD5|PGRP2_HUMAN	62,217.90	100.00%	4	5	10	0.00709%	11.20%
Tumor tissue	Isoform 2 of N-acetylmuramoyl-L-alanine amidase GN=PGLYRP2	sp|Q96PD5|PGRP2_HUMAN	68,001.00	99.90%	1	1	2	0.00153%	3.15%
Swab	Isoform 2 of N-acetylserotonin O-methyltransferase-like protein GN=ASMTL	sp|O95671|ASML_HUMAN	68,857.60	100.00%	2	2	2	0.00142%	5.29%
Tumor tissue	Isoform 2 of N-acetylserotonin O-methyltransferase-like protein GN=ASMTL	sp|O95671|ASML_HUMAN	67,193.70	100.00%	2	2	2	0.00153%	4.30%
Tumor tissue	Isoform 2 of NAD-dependent protein deacylase sirtuin-5, mitochondrial GN=SIRT5	sp|Q9NXA8|SIR5_HUMAN	32,673.70	100.00%	2	2	2	0.00153%	11.00%
Tumor tissue	Isoform 2 of NADH dehydrogenase [ubiquinone] 1 beta subcomplex subunit 11, mitochondrial GN=NDUFB11	sp|Q9NX14|NDUBB_HUMAN	18,364.10	100.00%	2	2	2	0.00153%	20.90%
Tumor tissue	Isoform 2 of NADH dehydrogenase [ubiquinone] flavoprotein 3, mitochondrial GN=NDUFV3	sp|P56181-2|NDUV3_HUMAN	50,984.20	100.00%	6	8	8	0.00612%	21.40%
Tumor tissue	Isoform 2 of NADH dehydrogenase [ubiquinone] iron-sulfur protein 2, mitochondrial GN=NDUFS2	sp|O75306|NDUS2_HUMAN	51,852.90	100.00%	9	13	13	0.00995%	29.10%
Pap test	Isoform 2 of NADH-cytochrome b5 reductase 3 GN=CYB5R3	sp|P00387|NB5R3_HUMAN	31,630.00	100.00%	3	3	3	0.00468%	12.90%
Tumor tissue	Isoform 2 of NADH-cytochrome b5 reductase 3 GN=CYB5R3	sp|P00387|NB5R3_HUMAN	34,236.30	100.00%	8	15	29	0.02220%	40.30%
Tumor tissue	Isoform 2 of N-alpha-acetyltransferase 10 GN=NAA10	sp|P41227|NAA10_HUMAN	24,784.10	100.00%	2	2	2	0.00153%	12.30%

Biological sample	Protein name	Protein accession numbers	Protein molecular weight (Da)	Protein identification probability	Exclusive unique peptide count	Exclusive unique spectrum count	Total spectrum count	Percentage of total spectra	Percentage sequence coverage
Tumor tissue	Isoform 2 of Nck-associated protein 1 GN=NCKAP1	sp|Q9Y2A7|NCKP1_HUMAN	129,521.50	100.00%	10	10	10	0.00766%	10.80%
Pap test	Isoform 2 of NEDD8-activating enzyme E1 catalytic subunit GN=UBA3	sp|Q8TBC4|UBA3_HUMAN	50,073.10	99.20%	1	1	1	0.00156%	3.34%
Swab	Isoform 2 of NEDD8-activating enzyme E1 catalytic subunit GN=UBA3	sp|Q8TBC4|UBA3_HUMAN	50,073.10	99.20%	1	1	1	0.00071%	1.78%
Tumor tissue	Isoform 2 of NEDD8-activating enzyme E1 catalytic subunit GN=UBA3	sp|Q8TBC4|UBA3_HUMAN	50,073.10	100.00%	4	5	5	0.00383%	11.60%
Tumor tissue	Isoform 2 of NEDD8-activating enzyme E1 regulatory subunit GN=NAE1	sp|Q13564|ULA1_HUMAN	50,626.10	100.00%	4	4	5	0.00383%	15.00%
Tumor tissue	Isoform 2 of Negative elongation factor E GN=NELFE	sp|P18615|NELFE_HUMAN	43,925.90	100.00%	1	1	5	0.00383%	16.50%
Tumor tissue	Isoform 2 of Nesprin-2 GN=SYNE2	sp|Q8WXH0|SYNE2_HUMAN	796,453.70	100.00%	1	1	29	0.02220%	6.11%
Tumor tissue	Isoform 2 of Nesprin-3 GN=SYNE3	sp|Q6ZMZ3|SYNE3_HUMAN	112,219.30	100.00%	4	4	4	0.00306%	4.64%
Tumor tissue	Isoform 2 of Neurochondrin GN=NCDN	sp|Q9UBB6|NCDN_HUMAN	77,245.10	100.00%	2	2	2	0.00153%	3.23%
Tumor tissue	Isoform 2 of Neuroguidin GN=NGDN	sp|Q8NEJ9|NGDN_HUMAN	35,894.90	100.00%	1	1	2	0.00153%	8.04%
Tumor tissue	Isoform 2 of Neutral alpha-glucosidase AB GN=GANAB	sp|Q14697-2|GANAB_HUMAN	109,440.20	100.00%	1	1	75	0.05740%	33.70%
Tumor tissue	Isoform 2 of Nexilin GN=NEXN	sp|Q0ZGT2-2|NEXN_HUMAN	79,364.00	100.00%	6	7	7	0.00536%	13.60%
Tumor tissue	Isoform 2 of NF-kappa-B essential modulator GN=IKBKG	sp|Q9Y6K9-2|NEMO_HUMAN	55,786.30	100.00%	2	2	2	0.00153%	6.78%
Pap test	Isoform 2 of Niban-like protein 1 GN=FAM129B	sp|Q96TA1|NIBL1_HUMAN	84,138.60	100.00%	7	7	9	0.01400%	14.30%
Swab	Isoform 2 of Niban-like protein 1 GN=FAM129B	sp|Q96TA1|NIBL1_HUMAN	82,684.10	100.00%	4	4	5	0.00354%	8.32%
Tumor tissue	Isoform 2 of Niban-like protein 1 GN=FAM129B	sp|Q96TA1|NIBL1_HUMAN	84,138.60	100.00%	16	19	25	0.01910%	29.50%

Biological sample	Protein name	Protein accession numbers	Protein molecular weight (Da)	Protein identification probability	Exclusive unique peptide count	Exclusive unique spectrum count	Total spectrum count	Percentage of total spectra	Percentage sequence coverage
Tumor tissue	Isoform 2 of Nicalin GN=NCLN	sp|Q969V3|NCLN_HUMAN	62,974.70	100.00%	7	9	9	0.00689%	14.20%
Tumor tissue	Isoform 2 of Nidogen-1 GN=NID1	sp|P14543|NID1_HUMAN	122,017.00	100.00%	10	11	12	0.00919%	10.20%
Tumor tissue	Isoform 2 of Nidogen-2 GN=NID2	sp|Q14112-2|NID2_HUMAN	140,866.10	100.00%	17	23	25	0.01910%	18.10%
Tumor tissue	Isoform 2 of NIF3-like protein 1 GN=NIF3L1	sp|Q9GZT8|NIF3L_HUMAN	41,968.60	100.00%	3	4	9	0.00689%	29.70%
Tumor tissue	Isoform 2 of Nuclear distribution protein nudE homolog 1 GN=NDE1	sp|Q9NXR1|NDE1_HUMAN	38,809.10	99.90%	1	1	3	0.00230%	9.55%
Tumor tissue	Isoform 2 of Nuclear factor NF-kappa-B p105 subunit GN=NFKB1	sp|P19838|NFKB1_HUMAN	105,429.60	100.00%	2	3	3	0.00230%	2.27%
Tumor tissue	Isoform 2 of Nuclear protein localization protein 4 homolog GN=NPLOC4	sp|Q8TAT6|NPL4_HUMAN	69,461.80	100.00%	4	4	4	0.00306%	9.56%
Tumor tissue	Isoform 2 of Nucleolysin TIAR GN=TIAL1	sp|Q01085|TIAR_HUMAN	43,447.90	100.00%	3	3	8	0.00612%	25.80%
Tumor tissue	Isoform 2 of Nucleoporin NUP53 GN=NUP35	sp|Q8NFH5|NUP53_HUMAN	34,774.50	100.00%	2	2	4	0.00306%	20.10%
Tumor tissue	Isoform 2 of Nucleoside diphosphate kinase A GN=NME1	sp|P15531|NDKA_HUMAN	19,653.70	99.90%	1	1	25	0.01910%	50.30%
Tumor tissue	Isoform 2 of NudC domain-containing protein 1 GN=NUDCD1	sp|Q96RS6|NUDC1_HUMAN	56,615.90	100.00%	5	6	6	0.00459%	12.80%
Tumor tissue	Isoform 2 of Opioid growth factor receptor GN=OGFR	sp|Q9NZT2|OGFR_HUMAN	73,324.60	100.00%	2	3	4	0.00306%	6.54%
Tumor tissue	Isoform 2 of Partner of Y14 and mago GN=PYM1	sp|Q9BRP8-2|PYM1_HUMAN	22,705.00	100.00%	4	4	4	0.00306%	32.50%
Tumor tissue	Isoform 2 of PDZ and LIM domain protein 3 GN=PDLIM3	sp|Q53GG5-2|PDLI3_HUMAN	34,279.70	100.00%	9	18	31	0.02370%	46.80%
Tumor tissue	Isoform 2 of PDZ and LIM domain protein 7 GN=PDLIM7	sp|Q9NR12-2|PDLI7_HUMAN	46,509.60	100.00%	5	8	25	0.01910%	28.60%
Swab	Isoform 2 of Peptidyl-glycine alpha-amidating monooxygenase GN=PAM	sp|P19021|AMD_HUMAN	108,403.60	100.00%	3	3	5	0.00354%	4.04%
Tumor tissue	Isoform 2 of Peptidyl-prolyl cis-trans isomerase FKBP7 GN=FKBP7	sp|Q9Y680|FKBP7_HUMAN	30,010.30	100.00%	4	4	4	0.00306%	17.60%

Biological sample	Protein name	Protein accession numbers	Protein molecular weight (Da)	Protein identification probability	Exclusive unique peptide count	Exclusive unique spectrum count	Total spectrum count	Percentage of total spectra	Percentage sequence coverage
Tumor tissue	Isoform 2 of Peptidyl-prolyl cis-trans isomerase FKBP8 GN=FKBP8	sp|Q14318|FKBP8_HUMAN	44,562.10	100.00%	2	2	4	0.00306%	9.69%
Tumor tissue	Isoform 2 of Peptidyl-prolyl cis-trans isomerase NIMA-interacting 4 GN=PIN4	sp|Q9Y237-2|PIN4_HUMAN	16,608.50	99.90%	1	1	3	0.00230%	14.70%
Tumor tissue	Isoform 2 of Periostin GN=POSTN	sp|Q15063-2|POSTN_HUMAN	87,024.40	100.00%	1	1	122	0.09340%	39.40%
Pap test	Isoform 2 of Peroxisomal acyl-coenzyme A oxidase 1 GN=ACOX1	sp|Q15067|ACOX1_HUMAN	74,669.50	99.90%	1	1	2	0.00312%	6.67%
Swab	Isoform 2 of Peroxisomal acyl-coenzyme A oxidase 1 GN=ACOX1	sp|Q15067|ACOX1_HUMAN	74,669.50	99.50%	1	1	1	0.00071%	3.64%
Tumor tissue	Isoform 2 of Peroxisomal acyl-coenzyme A oxidase 1 GN=ACOX1	sp|Q15067|ACOX1_HUMAN	74,425.40	100.00%	3	3	3	0.00230%	9.09%
Tumor tissue	Isoform 2 of Phosphatidylinositol 4-phosphate 5-kinase type-1 gamma GN=PIP5K1C	sp|O60331|PI51C_HUMAN	76,621.40	100.00%	2	2	3	0.00230%	5.71%
Tumor tissue	Isoform 2 of Phosphatidylinositol-binding clathrin assembly protein GN=PICALM	sp|Q13492|PICAL_HUMAN	70,756.10	100.00%	2	4	25	0.01910%	21.50%
Tumor tissue	Isoform 2 of Phosphoacetylglucosamine mutase GN=PGM3	sp|O95394|AGM1_HUMAN	62,342.00	100.00%	4	6	13	0.00995%	14.80%
Tumor tissue	Isoform 2 of Phosphoribosyl pyrophosphate synthase-associated protein 1 GN=PRPSAP1	sp|Q14558|KPRA_HUMAN	39,394.50	100.00%	3	3	3	0.00230%	10.10%
Tumor tissue	Isoform 2 of Phosphoribosyl pyrophosphate synthase-associated protein 2 GN=PRPSAP2	sp|O60256-2|KPRB_HUMAN	36,298.60	100.00%	3	3	3	0.00230%	15.80%
Pap test	Isoform 2 of PITH domain-containing protein 1 GN=PITHD1	sp|Q9GZP4|PITH1_HUMAN	24,121.20	100.00%	2	2	2	0.00312%	15.70%
Swab	Isoform 2 of PITH domain-containing protein 1 GN=PITHD1	sp|Q9GZP4|PITH1_HUMAN	24,178.30	100.00%	1	1	1	0.00071%	10.50%
Tumor tissue	Isoform 2 of PITH domain-containing protein 1 GN=PITHD1	sp|Q9GZP4|PITH1_HUMAN	24,178.30	100.00%	3	3	3	0.00230%	23.80%

Biological sample	Protein name	Protein accession numbers	Protein molecular weight (Da)	Protein identification probability	Exclusive unique peptide count	Exclusive unique spectrum count	Total spectrum count	Percentage of total spectra	Percentage sequence coverage
Tumor tissue	Isoform 2 of Pleckstrin homology-like domain family B member 1 GN=PHLDB1	sp|Q86UU1-2|PHLB1_HUMAN	144,742.60	100.00%	3	3	6	0.00459%	5.91%
Tumor tissue	Isoform 2 of Pre-B-cell leukemia transcription factor-interacting protein 1 GN=PBXIP1	sp|Q96AQ6|PBIP1_HUMAN	77,684.10	100.00%	11	11	11	0.00842%	20.10%
Tumor tissue	Isoform 2 of Pre-mRNA-processing factor 6 GN=PRPF6	sp|O94906-2|PRP6_HUMAN	102,431.50	100.00%	14	14	15	0.01150%	19.60%
Tumor tissue	Isoform 2 of Pre-mRNA-splicing factor RBM22 GN=RBM22	sp|Q9NW64|RBM22_HUMAN	41,152.80	100.00%	1	1	2	0.00153%	6.20%
Tumor tissue	Isoform 2 of Probable 28S rRNA (cytosine(4447)-C(5))-methyltransferase GN=NOP2	sp|P46087-2|NOP2_HUMAN	88,975.20	100.00%	3	3	5	0.00383%	7.67%
Tumor tissue	Isoform 2 of Probable E3 ubiquitin-protein ligase HERC4 GN=HERC4	sp|Q5GLZ8|HERC4_HUMAN	117,700.70	100.00%	3	3	3	0.00230%	3.91%
Tumor tissue	Isoform 2 of Probable ubiquitin carboxyl-terminal hydrolase FAF-X GN=USP9X	sp|Q93008|USP9X_HUMAN	290,467.30	100.00%	12	13	18	0.01380%	9.87%
Tumor tissue	Isoform 2 of Procollagen-lysine,2-oxoglutarate 5-dioxygenase 1 GN=PLOD1	sp|Q02809-2|PLOD1_HUMAN	88,274.90	100.00%	12	18	25	0.01910%	22.00%
Tumor tissue	Isoform 2 of Procollagen-lysine,2-oxoglutarate 5-dioxygenase 2 GN=PLOD2	sp|O00469-2|PLOD2_HUMAN	87,100.20	100.00%	13	18	27	0.02070%	24.50%
Swab	Isoform 2 of Programmed cell death protein 4 GN=PDCD4	sp|Q53EL6|PDCD4_HUMAN	50,577.40	100.00%	2	3	3	0.00213%	5.90%
Tumor tissue	Isoform 2 of Programmed cell death protein 4 GN=PDCD4	sp|Q53EL6|PDCD4_HUMAN	51,736.40	100.00%	6	7	8	0.00612%	13.80%
Tumor tissue	Isoform 2 of Pro-interleukin-16 GN=IL16	sp|Q14005|IL16_HUMAN	66,647.90	100.00%	5	5	5	0.00383%	5.94%
Pap test	Isoform 2 of Prominin-1 GN=PROM1	sp|O43490|PROM1_HUMAN	93,273.80	99.20%	1	1	1	0.00156%	1.52%
Swab	Isoform 2 of Prominin-1 GN=PROM1	sp|O43490|PROM1_HUMAN	97,206.40	100.00%	8	12	17	0.01200%	14.50%
Pap test	Isoform 2 of Prostaglandin reductase 1 GN=PTGR1	sp|Q14914|PTGR1_HUMAN	32,895.50	99.70%	1	1	1	0.00156%	3.32%

Biological sample	Protein name	Protein accession numbers	Protein molecular weight (Da)	Protein identification probability	Exclusive unique peptide count	Exclusive unique spectrum count	Total spectrum count	Percentage of total spectra	Percentage sequence coverage
Swab	Isoform 2 of Prostaglandin reductase 1 GN=PTGR1	sp|Q14914|PTGR1_HUMAN	32,895.50	100.00%	3	4	4	0.00283%	14.60%
Tumor tissue	Isoform 2 of Prostaglandin reductase 1 GN=PTGR1	sp|Q14914|PTGR1_HUMAN	32,895.50	100.00%	6	11	13	0.00995%	34.60%
Tumor tissue	Isoform 2 of Prostatic acid phosphatase GN=ACPP	sp|P15309|PPAP_HUMAN	44,567.80	100.00%	2	2	2	0.00153%	7.18%
Pap test	Isoform 2 of Proteasome activator complex subunit 1 GN=PSME1	sp|Q06323-2|PSME1_HUMAN	28,601.90	100.00%	6	8	21	0.03280%	35.60%
Swab	Isoform 2 of Proteasome activator complex subunit 1 GN=PSME1	sp|Q06323-2|PSME1_HUMAN	28,601.90	100.00%	7	8	24	0.01700%	35.60%
Tumor tissue	Isoform 2 of Proteasome activator complex subunit 1 GN=PSME1	sp|Q06323-2|PSME1_HUMAN	28,601.90	100.00%	8	10	25	0.01910%	41.20%
Pap test	Isoform 2 of Proteasome subunit alpha type-3 GN=PSMA3	sp|P25788|PSA3_HUMAN	28,433.70	100.00%	4	5	12	0.01870%	27.40%
Swab	Isoform 2 of Proteasome subunit alpha type-3 GN=PSMA3	sp|P25788|PSA3_HUMAN	27,647.70	100.00%	1	1	3	0.00213%	9.68%
Tumor tissue	Isoform 2 of Proteasome subunit alpha type-3 GN=PSMA3	sp|P25788|PSA3_HUMAN	28,433.70	100.00%	2	4	6	0.00459%	15.30%
Tumor tissue	Isoform 2 of Protein argonaute-2 GN=AGO2	sp|Q9UKV8|AGO2_HUMAN	97,208.00	100.00%	7	8	11	0.00842%	16.20%
Tumor tissue	Isoform 2 of Protein BRICK1 GN=BRK1	sp|Q8WUW1|BRK1_HUMAN	12,046.00	100.00%	2	2	2	0.00153%	17.90%
Tumor tissue	Isoform 2 of Protein BUD31 homolog GN=BUD31	sp|P41223|BUD31_HUMAN	17,000.30	100.00%	2	2	2	0.00153%	19.30%
Pap test	Isoform 2 of Protein disulfide-isomerase A6 GN=PDIA6	sp|Q15084|PDIA6_HUMAN	47,839.00	100.00%	6	8	12	0.01870%	16.50%
Swab	Isoform 2 of Protein disulfide-isomerase A6 GN=PDIA6	sp|Q15084|PDIA6_HUMAN	48,646.70	100.00%	6	6	10	0.00709%	16.30%
Tumor tissue	Isoform 2 of Protein disulfide-isomerase A6 GN=PDIA6	sp|Q15084|PDIA6_HUMAN	53,901.90	100.00%	13	25	39	0.02990%	28.70%
Swab	Isoform 2 of Protein ELYS GN=AHCTF1	sp|Q8WYP5-2|ELYS_HUMAN	256,002.00	100.00%	1	1	1	0.00071%	1.30%
Tumor tissue	Isoform 2 of Protein ELYS GN=AHCTF1	sp|Q8WYP5-2|ELYS_HUMAN	256,002.00	100.00%	3	3	3	0.00230%	2.09%
Tumor tissue	Isoform 2 of Protein FAM45A GN=FAM45A	sp|Q8TCE6|FA45A_HUMAN	40,514.00	100.00%	2	2	2	0.00153%	13.20%

Biological sample	Protein name	Protein accession numbers	Protein molecular weight (Da)	Protein identification probability	Exclusive unique peptide count	Exclusive unique spectrum count	Total spectrum count	Percentage of total spectra	Percentage sequence coverage
Tumor tissue	Isoform 2 of Protein FAM98A GN=FAM98A	sp|Q8NCA5|FA98A_HUMAN	55,401.40	100.00%	6	6	9	0.00689%	18.70%
Tumor tissue	Isoform 2 of Protein FAM98B GN=FAM98B	sp|Q52LJ0-2|FA98B_HUMAN	45,548.90	100.00%	3	5	8	0.00612%	14.50%
Tumor tissue	Isoform 2 of Protein farnesyltransferase/geranylgeranyltransferase type-1 subunit alpha GN=FNTA	sp|P49354|FNTA_HUMAN	36,493.10	100.00%	1	1	2	0.00153%	9.94%
Tumor tissue	Isoform 2 of Protein HID1 GN=HID1	sp|Q8IV36|HID1_HUMAN	88,746.70	100.00%	6	8	10	0.00766%	11.90%
Tumor tissue	Isoform 2 of Protein Hook homolog 1 GN=HOOK1	sp|Q9UJC3|HOOK1_HUMAN	84,649.40	100.00%	2	2	2	0.00153%	4.08%
Tumor tissue	Isoform 2 of Protein IMPACT GN=IMPACT	sp|Q9P2X3|IMPCT_HUMAN	36,477.20	99.80%	1	2	3	0.00230%	15.80%
Tumor tissue	Isoform 2 of Protein LSM14 homolog A GN=LSM14A	sp|Q8ND56|LS14A_HUMAN	46,412.60	99.90%	1	1	3	0.00230%	5.83%
Tumor tissue	Isoform 2 of Protein LSM14 homolog B GN=LSM14B	sp|Q9BX40|LS14B_HUMAN	42,022.10	99.90%	1	1	2	0.00153%	7.79%
Tumor tissue	Isoform 2 of Protein lunapark GN=LNP	sp|Q9C0E8|LNP_HUMAN	50,849.40	100.00%	3	3	3	0.00230%	8.27%
Tumor tissue	Isoform 2 of Protein LZIC GN=LZIC	sp|Q8WZA0-2|LZIC_HUMAN	23,926.80	100.00%	2	2	2	0.00153%	9.00%
Tumor tissue	Isoform 2 of Protein MEMO1 GN=MEMO1	sp|Q9Y316-2|MEMO1_HUMAN	31,308.20	100.00%	3	7	8	0.00612%	18.60%
Tumor tissue	Isoform 2 of Protein MON2 homolog GN=MON2	sp|Q7Z3U7|MON2_HUMAN	190,361.50	100.00%	2	2	2	0.00153%	1.55%
Swab	Isoform 2 of Protein phosphatase 1F GN=PPM1F	sp|P49593-2|PPM1F_HUMAN	38,517.50	99.80%	1	1	1	0.00071%	3.71%
Tumor tissue	Isoform 2 of Protein phosphatase 1F GN=PPM1F	sp|P49593-2|PPM1F_HUMAN	38,517.50	100.00%	4	4	4	0.00306%	18.90%
Swab	Isoform 2 of Protein phosphatase Slingshot homolog 3 GN=SSH3	sp|Q8TE77-2|SSH3_HUMAN	52,713.30	100.00%	1	1	1	0.00071%	1.91%
Tumor tissue	Isoform 2 of Protein phosphatase Slingshot homolog 3 GN=SSH3	sp|Q8TE77-2|SSH3_HUMAN	52,713.30	100.00%	2	2	2	0.00153%	7.64%
Tumor tissue	Isoform 2 of Protein quaking GN=QKI	sp|Q96PU8|QKI_HUMAN	35,132.20	100.00%	1	2	4	0.00306%	13.20%

Biological sample	Protein name	Protein accession numbers	Protein molecular weight (Da)	Protein identification probability	Exclusive unique peptide count	Exclusive unique spectrum count	Total spectrum count	Percentage of total spectra	Percentage sequence coverage
Tumor tissue	Isoform 2 of Protein SET GN=SET	sp|Q01105-2|SET_HUMAN	32,103.30	100.00%	1	1	10	0.00766%	37.20%
Swab	Isoform 2 of Protein SGT1 homolog GN=SUGT1	sp|Q9Y2Z0|SGT1_HUMAN	37,805.20	100.00%	2	2	2	0.00142%	6.01%
Tumor tissue	Isoform 2 of Protein SGT1 homolog GN=SUGT1	sp|Q9Y2Z0|SGT1_HUMAN	37,805.20	100.00%	5	5	5	0.00383%	19.50%
Tumor tissue	Isoform 2 of Protein TBRG4 GN=TBRG4	sp|Q969Z0|TBRG4_HUMAN	58,443.90	100.00%	3	5	6	0.00459%	9.79%
Pap test	Isoform 2 of Protein TFG GN=TFG	sp|Q92734|TFG_HUMAN	30,922.10	100.00%	2	2	2	0.00312%	7.32%
Swab	Isoform 2 of Protein TFG GN=TFG	sp|Q92734|TFG_HUMAN	31,349.50	98.90%	1	1	1	0.00071%	6.06%
Tumor tissue	Isoform 2 of Protein TFG GN=TFG	sp|Q92734|TFG_HUMAN	43,020.90	100.00%	7	9	15	0.01150%	29.00%
Tumor tissue	Isoform 2 of Protein transport protein Sec24B GN=SEC24B	sp|O95487|SC24B_HUMAN	133,630.90	100.00%	2	2	6	0.00459%	3.81%
Tumor tissue	Isoform 2 of Protein transport protein Sec24D GN=SEC24D	sp|O94855|SC24D_HUMAN	113,012.00	100.00%	5	6	7	0.00536%	9.00%
Tumor tissue	Isoform 2 of Protein unc-45 homolog A GN=UNC45A	sp|Q9H3U1|UN45A_HUMAN	101,677.40	100.00%	5	6	6	0.00459%	7.86%
Tumor tissue	Isoform 2 of Proto-oncogene tyrosine-protein kinase Src GN=SRC	sp|P12931|SRC_HUMAN	60,589.60	100.00%	1	1	3	0.00230%	6.64%
Tumor tissue	Isoform 2 of Putative RNA-binding protein Luc7-like 1 GN=LUC7L	sp|Q9NQ29|LUC7L_HUMAN	38,406.30	100.00%	1	1	4	0.00306%	10.20%
Tumor tissue	Isoform 2 of Pyruvate dehydrogenase E1 component subunit beta, mitochondrial GN=PDHB	sp|P11177|ODPB_HUMAN	39,233.40	100.00%	7	10	11	0.00842%	29.30%
Tumor tissue	Isoform 2 of Rab3 GTPase-activating protein catalytic subunit GN=RAB3GAP1	sp|Q15042|RB3GP_HUMAN	110,525.90	100.00%	4	5	5	0.00383%	6.48%
Tumor tissue	Isoform 2 of Rab-like protein 6 GN=RABL6	sp|Q3YEC7|RABL6_HUMAN	79,549.80	100.00%	1	1	2	0.00153%	3.97%
Tumor tissue	Isoform 2 of Ragulator complex protein LAMTOR2 GN=LAMTOR2	sp|Q9Y2Q5|LTOR2_HUMAN	13,507.90	100.00%	2	2	3	0.00230%	18.00%
Tumor tissue	Isoform 2 of Ran-specific GTPase-activating protein GN=RANBP1	sp|P43487|RANG_HUMAN	23,310.80	100.00%	3	8	11	0.00842%	28.00%

Biological sample	Protein name	Protein accession numbers	Protein molecular weight (Da)	Protein identification probability	Exclusive unique peptide count	Exclusive unique spectrum count	Total spectrum count	Percentage of total spectra	Percentage sequence coverage
Tumor tissue	Isoform 2 of Ras-related protein Rab-5A GN=RAB5A	sp|P20339|RAB5A_HUMAN	22,178.20	100.00%	2	3	6	0.00459%	26.90%
Swab	Isoform 2 of Ras-related protein Rab-5C GN=RAB5C	sp|P51148|RAB5C_HUMAN	23,482.70	100.00%	4	4	5	0.00354%	22.90%
Tumor tissue	Isoform 2 of Ras-related protein Rab-5C GN=RAB5C	sp|P51148|RAB5C_HUMAN	27,035.70	100.00%	4	8	12	0.00919%	29.30%
Pap test	Isoform 2 of Ras-related protein Rab-6A GN=RAB6A	sp|P20340-2|RAB6A_HUMAN	23,549.40	100.00%	2	2	4	0.00624%	17.30%
Tumor tissue	Isoform 2 of Ras-related protein Rab-6A GN=RAB6A	sp|P20340-2|RAB6A_HUMAN	23,549.40	100.00%	3	5	12	0.00919%	23.10%
Swab	Isoform 2 of Ras-responsive element-binding protein 1 GN=RREB1	sp|Q92766|RREB1_HUMAN	181,421.10	99.90%	1	1	1	0.00071%	1.15%
Tumor tissue	Isoform 2 of Ras-responsive element-binding protein 1 GN=RREB1	sp|Q92766|RREB1_HUMAN	187,551.70	99.70%	1	1	2	0.00153%	1.89%
Tumor tissue	Isoform 2 of Receptor-type tyrosine-protein phosphatase alpha GN=PTPRA	sp|P18433|PTPRA_HUMAN	90,600.60	100.00%	2	2	3	0.00230%	3.91%
Tumor tissue	Isoform 2 of Receptor-type tyrosine-protein phosphatase F GN=PTPRF	sp|P10586|PTPRF_HUMAN	211,685.90	100.00%	8	8	9	0.00689%	6.11%
Pap test	Isoform 2 of Redox-regulatory protein FAM213A GN=FAM213A	sp|Q9BRX8|F213A_HUMAN	24,463.90	100.00%	4	5	7	0.01090%	22.00%
Tumor tissue	Isoform 2 of Redox-regulatory protein FAM213A GN=FAM213A	sp|Q9BRX8|F213A_HUMAN	24,463.90	100.00%	3	4	4	0.00306%	17.00%
Tumor tissue	Isoform 2 of Regulator of chromosome condensation GN=RCC1	sp|P18754|RCC1_HUMAN	48,144.30	100.00%	6	8	8	0.00612%	20.40%
Swab	Isoform 2 of Regulator of nonsense transcripts 1 GN=UPF1	sp|Q92900-2|RENT1_HUMAN	123,037.30	100.00%	1	1	1	0.00071%	2.24%
Tumor tissue	Isoform 2 of Regulator of nonsense transcripts 1 GN=UPF1	sp|Q92900-2|RENT1_HUMAN	123,037.30	100.00%	14	16	17	0.01300%	17.90%
Tumor tissue	Isoform 2 of Remodeling and spacing factor 1 GN=RSF1	sp|Q96T23|RSF1_HUMAN	163,824.60	100.00%	4	4	4	0.00306%	4.18%
Tumor tissue	Isoform 2 of Replication factor C subunit 1 GN=RFC1	sp|P35251|RFC1_HUMAN	128,260.40	100.00%	4	4	4	0.00306%	5.06%

Biological sample	Protein name	Protein accession numbers	Protein molecular weight (Da)	Protein identification probability	Exclusive unique peptide count	Exclusive unique spectrum count	Total spectrum count	Percentage of total spectra	Percentage sequence coverage
Tumor tissue	Isoform 2 of Replication factor C subunit 2 GN=RFC2	sp|P35250|RFC2_HUMAN	39,158.30	100.00%	2	2	6	0.00459%	14.70%
Tumor tissue	Isoform 2 of Replication factor C subunit 5 GN=RFC5	sp|P40937|RFC5_HUMAN	36,106.70	100.00%	3	3	7	0.00536%	27.00%
Tumor tissue	Isoform 2 of Replication protein A 32 kDa subunit GN=RPA2	sp|P15927|RFA2_HUMAN	29,246.70	100.00%	3	4	4	0.00306%	15.10%
Swab	Isoform 2 of Reticulocalbin-2 GN=RCN2	sp|Q14257|RCN2_HUMAN	39,140.50	99.10%	1	1	1	0.00071%	5.67%
Tumor tissue	Isoform 2 of Reticulocalbin-2 GN=RCN2	sp|Q14257|RCN2_HUMAN	39,140.50	100.00%	6	6	6	0.00459%	22.70%
Tumor tissue	Isoform 2 of Reticulon-4 GN=RTN4	sp|Q9NQC3-2|RTN4_HUMAN	40,318.70	100.00%	1	1	13	0.00995%	31.40%
Tumor tissue	Isoform 2 of Rho GTPase-activating protein 17 GN=ARHGAP17	sp|Q68EM7|RHG17_HUMAN	87,609.50	100.00%	2	3	4	0.00306%	4.48%
Tumor tissue	Isoform 2 of Rho GTPase-activating protein 18 GN=ARHGAP18	sp|Q8N392|RHG18_HUMAN	74,978.90	100.00%	5	5	6	0.00459%	10.20%
Tumor tissue	Isoform 2 of Ribose-phosphate pyrophosphokinase 2 GN=PRPS2	sp|P11908|PRPS2_HUMAN	34,769.50	100.00%	1	1	9	0.00689%	16.80%
Tumor tissue	Isoform 2 of Ribosomal biogenesis protein LAS1L GN=LAS1L	sp|Q9Y4W2|LAS1L_HUMAN	81,243.60	100.00%	4	4	5	0.00383%	7.25%
Tumor tissue	Isoform 2 of Ribosomal protein S6 kinase alpha-1 GN=RPS6KA1	sp|Q15418-2|KS6A1_HUMAN	83,935.10	100.00%	2	2	6	0.00459%	10.30%
Swab	Isoform 2 of Ribulose-phosphate 3-epimerase GN=RPE	sp|Q96AT9|RPE_HUMAN	23,924.30	99.90%	1	1	4	0.00283%	18.20%
Tumor tissue	Isoform 2 of Ribulose-phosphate 3-epimerase GN=RPE	sp|Q96AT9|RPE_HUMAN	24,927.80	98.40%	1	1	1	0.00077%	9.55%
Tumor tissue	Isoform 2 of RNA 3'-terminal phosphate cyclase GN=RTCA	sp|O00442|RTCA_HUMAN	39,337.50	100.00%	4	6	6	0.00459%	13.20%
Swab	Isoform 2 of RNA-binding protein 39 GN=RBM39	sp|Q14498|RBM39_HUMAN	57,091.40	99.90%	1	1	2	0.00142%	2.86%
Tumor tissue	Isoform 2 of RNA-binding protein 39 GN=RBM39	sp|Q14498|RBM39_HUMAN	59,381.40	100.00%	7	7	9	0.00689%	16.60%
Tumor tissue	Isoform 2 of RNA-binding protein 8A GN=RBM8A	sp|Q9Y5S9|RBM8A_HUMAN	19,760.30	100.00%	3	4	4	0.00306%	22.00%
Tumor tissue	Isoform 2 of RRP12-like protein GN=RRP12	sp|Q5JTH9|RRP12_HUMAN	137,117.20	100.00%	5	5	5	0.00383%	5.01%

Biological sample	Protein name	Protein accession numbers	Protein molecular weight (Da)	Protein identification probability	Exclusive unique peptide count	Exclusive unique spectrum count	Total spectrum count	Percentage of total spectra	Percentage sequence coverage
Swab	Isoform 2 of Scaffold attachment factor B1 GN=SAFB	sp|Q15424|SAFB1_HUMAN	102,769.00	100.00%	2	2	2	0.00142%	4.83%
Tumor tissue	Isoform 2 of Scaffold attachment factor B1 GN=SAFB	sp|Q15424|SAFB1_HUMAN	95,181.90	100.00%	3	4	12	0.00919%	11.00%
Pap test	Isoform 2 of Sciellin GN=SCEL	sp|O95171-2|SCEL_HUMAN	75,327.90	100.00%	13	13	13	0.02030%	23.10%
Tumor tissue	Isoform 2 of Sec1 family domain-containing protein 2 GN=SCFD2	sp|Q8WU76|SCFD2_HUMAN	69,992.50	100.00%	3	3	3	0.00230%	5.63%
Pap test	Isoform 2 of Secernin-1 GN=SCRN1	sp|Q12765|SCRN1_HUMAN	48,713.60	100.00%	1	1	1	0.00156%	2.53%
Swab	Isoform 2 of Secernin-1 GN=SCRN1	sp|Q12765|SCRN1_HUMAN	48,713.60	100.00%	2	3	5	0.00354%	6.45%
Tumor tissue	Isoform 2 of Secernin-1 GN=SCRN1	sp|Q12765|SCRN1_HUMAN	48,713.60	100.00%	6	8	11	0.00842%	16.60%
Tumor tissue	Isoform 2 of Secretory carrier-associated membrane protein 3 GN=SCAMP3	sp|O14828|SCAM3_HUMAN	38,287.70	100.00%	4	4	5	0.00383%	20.20%
Tumor tissue	Isoform 2 of Septin-11 GN=SEPT11	sp|Q9NVA2-2|SEP11_HUMAN	50,823.90	100.00%	3	4	23	0.01760%	32.30%
Pap test	Isoform 2 of Septin-2 GN=SEPT2	sp|Q15019|SEPT2_HUMAN	45,461.60	100.00%	2	3	4	0.00624%	11.90%
Tumor tissue	Isoform 2 of Septin-2 GN=SEPT2	sp|Q15019|SEPT2_HUMAN	45,461.60	100.00%	5	8	26	0.01990%	32.60%
Tumor tissue	Isoform 2 of Septin-9 GN=SEPT9	sp|Q9UHD8|SEPT9_HUMAN	63,503.10	100.00%	6	6	19	0.01450%	37.90%
Tumor tissue	Isoform 2 of Sequestosome-1 GN=SQSTM1	sp|Q13501-2|SQSTM_HUMAN	38,629.00	100.00%	1	1	4	0.00306%	16.60%
Swab	Isoform 2 of Serine hydroxymethyltransferase, cytosolic GN=SHMT1	sp|P34896|GLYC_HUMAN	49,029.10	99.90%	1	1	1	0.00071%	2.93%
Tumor tissue	Isoform 2 of Serine hydroxymethyltransferase, cytosolic GN=SHMT1	sp|P34896|GLYC_HUMAN	49,029.10	100.00%	4	4	6	0.00459%	17.60%
Swab	Isoform 2 of Serine/arginine-rich splicing factor 3 GN=SRSF3	sp|P84103|SRSF3_HUMAN	14,203.30	100.00%	2	2	2	0.00142%	16.90%
Tumor tissue	Isoform 2 of Serine/arginine-rich splicing factor 3 GN=SRSF3	sp|P84103|SRSF3_HUMAN	14,203.30	100.00%	5	7	15	0.01150%	46.00%
Tumor tissue	Isoform 2 of Serine/threonine-protein kinase 26 GN=STK26	sp|Q9P289-2|STK26_HUMAN	37,771.40	100.00%	1	1	4	0.00306%	12.10%

Biological sample	Protein name	Protein accession numbers	Protein molecular weight (Da)	Protein identification probability	Exclusive unique peptide count	Exclusive unique spectrum count	Total spectrum count	Percentage of total spectra	Percentage sequence coverage
Tumor tissue	Isoform 2 of Serine/threonine-protein kinase 3 GN=STK3	sp|Q13188-2|STK3_HUMAN	59,463.20	100.00%	3	3	8	0.00612%	18.90%
Tumor tissue	Isoform 2 of Serine/threonine-protein kinase N1 GN=PKN1	sp|Q16512|PKN1_HUMAN	104,676.00	100.00%	4	4	4	0.00306%	6.86%
Tumor tissue	Isoform 2 of Serine/threonine-protein kinase N2 GN=PKN2	sp|Q16513|PKN2_HUMAN	110,348.00	100.00%	2	2	2	0.00153%	2.79%
Tumor tissue	Isoform 2 of Serine/threonine-protein phosphatase 2A 55 kDa regulatory subunit B alpha isoform GN=PPP2R2A	sp|P63151|2ABA_HUMAN	51,693.40	100.00%	3	3	4	0.00306%	9.85%
Tumor tissue	Isoform 2 of Serine/threonine-protein phosphatase 2A 56 kDa regulatory subunit epsilon isoform GN=PPP2R5E	sp|Q16537|2A5E_HUMAN	54,044.90	100.00%	3	3	3	0.00230%	9.74%
Tumor tissue	Isoform 2 of Serine/threonine-protein phosphatase 4 regulatory subunit 3A GN=PPP4R3A	sp|Q6IN85|P4R3A_HUMAN	95,372.30	100.00%	1	2	3	0.00230%	3.54%
Tumor tissue	Isoform 2 of Serine/threonine-protein phosphatase 6 regulatory ankyrin repeat subunit B GN=ANKRD44	sp|Q8N8A2|ANR44_HUMAN	107,604.40	100.00%	2	2	3	0.00230%	4.43%
Pap test	Isoform 2 of Serpin B12 GN=SERPINB12	sp|Q96P63-2|SPB12_HUMAN	48,447.10	100.00%	2	2	3	0.00468%	8.94%
Pap test	Isoform 2 of Serrate RNA effector molecule homolog GN=SRRT	sp|Q9BXP5|SRRT_HUMAN	100,149.90	98.30%	1	1	1	0.00156%	3.56%
Tumor tissue	Isoform 2 of Serrate RNA effector molecule homolog GN=SRRT	sp|Q9BXP5|SRRT_HUMAN	96,225.50	100.00%	7	9	10	0.00766%	10.30%
Tumor tissue	Isoform 2 of Short/branched chain specific acyl-CoA dehydrogenase, mitochondrial GN=ACADSB	sp|P45954|ACDSB_HUMAN	47,487.10	100.00%	2	2	2	0.00153%	11.20%
Tumor tissue	Isoform 2 of Signal transducer and activator of transcription 2 GN=STAT2	sp|P52630|STAT2_HUMAN	97,919.50	100.00%	7	7	11	0.00842%	11.90%
Tumor tissue	Isoform 2 of Signal transducing adapter molecule 1 GN=STAM	sp|Q92783|STAM1_HUMAN	44,972.70	100.00%	2	2	3	0.00230%	7.94%

Biological sample	Protein name	Protein accession numbers	Protein molecular weight (Da)	Protein identification probability	Exclusive unique peptide count	Exclusive unique spectrum count	Total spectrum count	Percentage of total spectra	Percentage sequence coverage
Tumor tissue	Isoform 2 of Sister chromatid cohesion protein PDS5 homolog B GN=PDS5B	sp|Q9NTI5|PDS5B_HUMAN	164,672.50	100.00%	17	17	17	0.01300%	15.00%
Tumor tissue	Isoform 2 of Small integral membrane protein 20 GN=SMIM20	sp|Q8N5G0|SMI20_HUMAN	18,402.90	100.00%	2	2	3	0.00230%	13.10%
Pap test	Isoform 2 of Sodium/potassium-transporting ATPase subunit beta-1 GN=ATP1B1	sp|P05026|AT1B1_HUMAN	34,894.80	99.20%	1	1	1	0.00156%	4.65%
Tumor tissue	Isoform 2 of Sodium/potassium-transporting ATPase subunit beta-1 GN=ATP1B1	sp|P05026|AT1B1_HUMAN	34,894.80	100.00%	2	2	2	0.00153%	8.31%
Tumor tissue	Isoform 2 of Sodium-dependent phosphate transport protein 2B GN=SLC34A2	sp|O95436-2|NPT2B_HUMAN	75,659.40	100.00%	5	6	6	0.00459%	7.26%
Tumor tissue	Isoform 2 of Solute carrier family 12 member 2 GN=SLC12A2	sp|P55011|S12A2_HUMAN	129,684.00	100.00%	2	2	2	0.00153%	2.68%
Tumor tissue	Isoform 2 of Solute carrier family 12 member 4 GN=SLC12A4	sp|Q9UP95|S12A4_HUMAN	118,623.10	100.00%	1	1	4	0.00306%	4.68%
Tumor tissue	Isoform 2 of Solute carrier family 41 member 3 GN=SLC41A3	sp|Q96GZ6|S41A3_HUMAN	49,916.70	99.90%	1	1	2	0.00153%	6.20%
Tumor tissue	Isoform 2 of Sorbin and SH3 domain-containing protein 1 GN=SORBS1	sp|Q9BX66|SRBS1_HUMAN	127,279.50	100.00%	1	1	28	0.02140%	21.30%
Tumor tissue	Isoform 2 of Sorting nexin-18 GN=SNX18	sp|Q96RF0-2|SNX18_HUMAN	69,007.10	100.00%	7	7	7	0.00536%	14.60%
Tumor tissue	Isoform 2 of Spectrin alpha chain, erythrocytic 1 GN=SPTA1	sp|P02549-2|SPTA1_HUMAN	279,678.70	100.00%	10	10	10	0.00766%	6.29%
Tumor tissue	Isoform 2 of Spectrin beta chain, erythrocytic GN=SPTB	sp|P11277-2|SPTB1_HUMAN	267,830.20	100.00%	4	4	8	0.00612%	3.74%
Pap test	Isoform 2 of Spectrin beta chain, non-erythrocytic 2 GN=SPTBN2	sp|O15020|SPTN2_HUMAN	271,325.90	100.00%	2	2	2	0.00312%	1.27%
Tumor tissue	Isoform 2 of Spectrin beta chain, non-erythrocytic 2 GN=SPTBN2	sp|O15020|SPTN2_HUMAN	271,325.90	100.00%	18	22	31	0.02370%	15.00%
Tumor tissue	Isoform 2 of Spermatid perinuclear RNA-binding protein GN=STRBP	sp|Q96SI9|STRBP_HUMAN	73,654.30	100.00%	1	1	8	0.00612%	6.84%

Biological sample	Protein name	Protein accession numbers	Protein molecular weight (Da)	Protein identification probability	Exclusive unique peptide count	Exclusive unique spectrum count	Total spectrum count	Percentage of total spectra	Percentage sequence coverage
Pap test	Isoform 2 of STE20-like serine/threonine-protein kinase GN=SLK	sp|Q9H2G2-2|SLK_HUMAN	138,998.60	100.00%	2	3	5	0.00780%	2.99%
Swab	Isoform 2 of STE20-like serine/threonine-protein kinase GN=SLK	sp|Q9H2G2-2|SLK_HUMAN	138,998.60	100.00%	1	1	1	0.00071%	1.25%
Tumor tissue	Isoform 2 of STE20-like serine/threonine-protein kinase GN=SLK	sp|Q9H2G2-2|SLK_HUMAN	138,998.60	100.00%	6	8	9	0.00689%	8.06%
Tumor tissue	Isoform 2 of Striatin GN=STRN	sp|O43815|STRN_HUMAN	86,133.80	100.00%	5	6	6	0.00459%	12.20%
Pap test	Isoform 2 of Succinate--CoA ligase [ADP-forming] subunit beta, mitochondrial GN=SUCLA2	sp|Q9P2R7|SUCB1_HUMAN	48,040.70	99.20%	1	1	1	0.00156%	2.72%
Swab	Isoform 2 of Succinate--CoA ligase [ADP-forming] subunit beta, mitochondrial GN=SUCLA2	sp|Q9P2R7|SUCB1_HUMAN	48,040.70	99.70%	1	1	1	0.00071%	2.72%
Tumor tissue	Isoform 2 of Succinate--CoA ligase [ADP-forming] subunit beta, mitochondrial GN=SUCLA2	sp|Q9P2R7|SUCB1_HUMAN	48,040.70	100.00%	3	4	7	0.00536%	14.30%
Pap test	Isoform 2 of Succinate-semialdehyde dehydrogenase, mitochondrial GN=ALDH5A1	sp|P51649|SSDH_HUMAN	57,214.90	100.00%	2	2	2	0.00312%	4.01%
Tumor tissue	Isoform 2 of Succinate-semialdehyde dehydrogenase, mitochondrial GN=ALDH5A1	sp|P51649|SSDH_HUMAN	58,653.70	100.00%	6	6	7	0.00536%	13.00%
Tumor tissue	Isoform 2 of SUN domain-containing protein 2 GN=SUN2	sp|Q9UH99|SUN2_HUMAN	79,613.80	100.00%	10	16	18	0.01380%	23.30%
Tumor tissue	Isoform 2 of SWI/SNF-related matrix-associated actin-dependent regulator of chromatin subfamily D member 2 GN=SMARCD2	sp|Q92925-2|SMRD2_HUMAN	57,245.40	100.00%	4	4	4	0.00306%	13.90%
Tumor tissue	Isoform 2 of Synaptopodin GN=SYNPO	sp|Q8N3V7-2|SYNPO_HUMAN	96,397.80	100.00%	1	1	7	0.00536%	10.10%
Tumor tissue	Isoform 2 of Syntaxin-5 GN=STX5	sp|Q13190|STX5_HUMAN	39,673.50	100.00%	5	7	8	0.00612%	27.90%

Biological sample	Protein name	Protein accession numbers	Protein molecular weight (Da)	Protein identification probability	Exclusive unique peptide count	Exclusive unique spectrum count	Total spectrum count	Percentage of total spectra	Percentage sequence coverage
Pap test	Isoform 2 of Syntaxin-7 GN=STX7	sp|O15400-2|STX7_HUMAN	27,400.20	100.00%	1	1	1	0.00156%	6.28%
Swab	Isoform 2 of Syntaxin-7 GN=STX7	sp|O15400-2|STX7_HUMAN	27,400.20	100.00%	2	2	2	0.00142%	10.50%
Tumor tissue	Isoform 2 of Syntaxin-7 GN=STX7	sp|O15400-2|STX7_HUMAN	27,400.20	100.00%	4	4	4	0.00306%	23.40%
Swab	Isoform 2 of Syntaxin-binding protein 2 GN=STXBP2	sp|Q15833|STXB2_HUMAN	66,140.60	100.00%	6	6	10	0.00709%	21.90%
Tumor tissue	Isoform 2 of Syntaxin-binding protein 2 GN=STXBP2	sp|Q15833|STXB2_HUMAN	66,140.60	100.00%	7	8	13	0.00995%	18.60%
Tumor tissue	Isoform 2 of TBC1 domain family member 13 GN=TBC1D13	sp|Q9NVG8|TBC13_HUMAN	32,172.80	100.00%	2	2	2	0.00153%	9.82%
Tumor tissue	Isoform 2 of TBC1 domain family member 5 GN=TBC1D5	sp|Q92609|TBCD5_HUMAN	91,218.30	100.00%	3	4	4	0.00306%	6.12%
Tumor tissue	Isoform 2 of TBC1 domain family member 9B GN=TBC1D9B	sp|Q66K14|TBC9B_HUMAN	140,528.60	100.00%	4	4	6	0.00459%	6.00%
Pap test	Isoform 2 of T-complex protein 1 subunit gamma GN=CCT3	sp|P49368-2|TCPG_HUMAN	56,432.00	100.00%	3	3	4	0.00624%	12.00%
Tumor tissue	Isoform 2 of T-complex protein 1 subunit gamma GN=CCT3	sp|P49368-2|TCPG_HUMAN	56,432.00	100.00%	8	10	23	0.01760%	29.20%
Tumor tissue	Isoform 2 of Telomere-associated protein RIF1 GN=RIF1	sp|Q5UIP0-2|RIF1_HUMAN	271,692.90	100.00%	2	2	2	0.00153%	1.23%
Tumor tissue	Isoform 2 of Tensin-1 GN=TNS1	sp|Q9HBL0-2|TENS1_HUMAN	47,240.20	100.00%	1	1	8	0.00612%	21.90%
Swab	Isoform 2 of Testin GN=TES	sp|Q9UGI8|TES_HUMAN	46,909.30	100.00%	1	1	1	0.00071%	2.43%
Tumor tissue	Isoform 2 of Testin GN=TES	sp|Q9UGI8|TES_HUMAN	47,995.60	100.00%	10	12	14	0.01070%	25.00%
Tumor tissue	Isoform 2 of Testis-expressed sequence 10 protein GN=TEX10	sp|Q9NXF1|TEX10_HUMAN	103,919.20	100.00%	5	6	8	0.00612%	7.23%
Tumor tissue	Isoform 2 of Tether containing UBX domain for GLUT4 GN=ASPSCR1	sp|Q9BZE9-2|ASPC1_HUMAN	69,990.40	100.00%	1	1	3	0.00230%	5.41%
Tumor tissue	Isoform 2 of TGF-beta-activated kinase 1 and MAP3K7-binding protein 1 GN=TAB1	sp|Q15750|TAB1_HUMAN	54,644.00	100.00%	3	3	3	0.00230%	13.60%
Pap test	Isoform 2 of Thioredoxin-dependent peroxide reductase, mitochondrial GN=PRDX3	sp|P30048|PRDX3_HUMAN	27,692.70	100.00%	3	4	5	0.00780%	16.80%

Biological sample	Protein name	Protein accession numbers	Protein molecular weight (Da)	Protein identification probability	Exclusive unique peptide count	Exclusive unique spectrum count	Total spectrum count	Percentage of total spectra	Percentage sequence coverage
Swab	Isoform 2 of Thioredoxin-dependent peroxide reductase, mitochondrial GN=PRDX3	sp|P30048|PRDX3_HUMAN	27,692.70	100.00%	2	2	2	0.00142%	8.82%
Tumor tissue	Isoform 2 of Thioredoxin-dependent peroxide reductase, mitochondrial GN=PRDX3	sp|P30048|PRDX3_HUMAN	27,692.70	100.00%	7	19	28	0.02140%	30.70%
Tumor tissue	Isoform 2 of Thioredoxin-related transmembrane protein 2 GN=TMX2	sp|Q9Y320|TMX2_HUMAN	29,643.50	100.00%	2	3	3	0.00230%	14.30%
Pap test	Isoform 2 of Threonine--tRNA ligase, cytoplasmic GN=TARS	sp|P26639|SYTC_HUMAN	83,437.80	100.00%	2	2	2	0.00312%	3.17%
Tumor tissue	Isoform 2 of Threonine--tRNA ligase, cytoplasmic GN=TARS	sp|P26639|SYTC_HUMAN	83,437.80	100.00%	11	11	12	0.00919%	15.50%
Tumor tissue	Isoform 2 of Thymidylate kinase GN=DTYMK	sp|P23919|KTHY_HUMAN	21,064.50	100.00%	4	4	4	0.00306%	20.70%
Swab	Isoform 2 of Tissue-type plasminogen activator GN=PLAT	sp|P00750-2|TPA_HUMAN	32,174.80	98.80%	1	2	3	0.00213%	8.93%
Tumor tissue	Isoform 2 of Transcription elongation factor A protein 1 GN=TCEA1	sp|P23193-2|TCEA1_HUMAN	31,664.50	100.00%	2	2	5	0.00383%	10.00%
Tumor tissue	Isoform 2 of Transcription elongation factor A protein-like 4 GN=TCEAL4	sp|Q96EI5-2|TCAL4_HUMAN	40,578.50	100.00%	2	2	3	0.00230%	12.60%
Tumor tissue	Isoform 2 of Transcription elongation factor SPT5 GN=SUPT5H	sp|O00267|SPT5H_HUMAN	120,999.90	100.00%	12	14	14	0.01070%	17.10%
Tumor tissue	Isoform 2 of Transcription elongation regulator 1 GN=TCERG1	sp|O14776|TCRG1_HUMAN	121,692.40	100.00%	4	4	4	0.00306%	4.09%
Tumor tissue	Isoform 2 of Transforming growth factor beta-1-induced transcript 1 protein GN=TGFB1I1	sp|O43294|TGFI1_HUMAN	47,941.10	100.00%	3	5	14	0.01070%	14.90%
Pap test	Isoform 2 of Transgelin-2 GN=TAGLN2	sp|P37802-2|TAGL2_HUMAN	24,454.40	100.00%	4	4	4	0.00624%	19.50%
Swab	Isoform 2 of Transgelin-2 GN=TAGLN2	sp|P37802-2|TAGL2_HUMAN	24,454.40	100.00%	7	11	24	0.01700%	35.50%
Tumor tissue	Isoform 2 of Transgelin-2 GN=TAGLN2	sp|P37802-2|TAGL2_HUMAN	24,454.40	100.00%	9	22	70	0.05360%	43.20%

Biological sample	Protein name	Protein accession numbers	Protein molecular weight (Da)	Protein identification probability	Exclusive unique peptide count	Exclusive unique spectrum count	Total spectrum count	Percentage of total spectra	Percentage sequence coverage
Pap test	Isoform 2 of Transketolase GN=TKT	sp|P29401|TKT_HUMAN	68,815.50	100.00%	19	30	86	0.13400%	37.60%
Swab	Isoform 2 of Transketolase GN=TKT	sp|P29401|TKT_HUMAN	68,815.50	100.00%	21	31	87	0.06170%	43.60%
Tumor tissue	Isoform 2 of Transketolase GN=TKT	sp|P29401|TKT_HUMAN	68,815.50	100.00%	21	32	54	0.04130%	41.70%
Tumor tissue	Isoform 2 of Transmembrane and TPR repeat-containing protein 3 GN=TMTC3	sp|Q6ZXV5|TMTC3_HUMAN	103,886.10	100.00%	4	4	5	0.00383%	5.25%
Pap test	Isoform 2 of Transmembrane glycoprotein NMB GN=GPNMB	sp|Q14956|GPNMB_HUMAN	62,643.20	100.00%	2	5	9	0.01400%	4.82%
Swab	Isoform 2 of Transportin-1 GN=TNPO1	sp|Q92973|TNPO1_HUMAN	101,313.60	100.00%	1	1	2	0.00142%	2.58%
Tumor tissue	Isoform 2 of Transportin-1 GN=TNPO1	sp|Q92973|TNPO1_HUMAN	101,313.60	100.00%	7	9	17	0.01300%	13.50%
Tumor tissue	Isoform 2 of Transportin-2 GN=TNPO2	sp|O14787-2|TNPO2_HUMAN	100,411.10	100.00%	2	3	8	0.00612%	7.22%
Pap test	Isoform 2 of Triosephosphate isomerase GN=TPI1	sp|P60174|TPIS_HUMAN	30,790.80	100.00%	15	32	110	0.17200%	74.30%
Swab	Isoform 2 of Triosephosphate isomerase GN=TPI1	sp|P60174|TPIS_HUMAN	26,669.10	100.00%	15	26	122	0.08650%	79.10%
Tumor tissue	Isoform 2 of Triosephosphate isomerase GN=TPI1	sp|P60174|TPIS_HUMAN	30,790.80	100.00%	11	27	68	0.05210%	42.20%
Tumor tissue	Isoform 2 of tRNA (adenine(58)-N(1))-methyltransferase non-catalytic subunit TRM6 GN=TRMT6	sp|Q9UJA5-2|TRM6_HUMAN	44,963.60	100.00%	2	2	2	0.00153%	4.76%
Tumor tissue	Isoform 2 of tRNA (cytosine(34)-C(5))-methyltransferase GN=NSUN2	sp|Q08J23|NSUN2_HUMAN	86,472.80	100.00%	9	9	10	0.00766%	19.80%
Tumor tissue	Isoform 2 of Tropomodulin-1 GN=TMOD1	sp|P28289-2|TMOD1_HUMAN	26,014.80	100.00%	4	5	5	0.00383%	24.10%
Pap test	Isoform 2 of Tropomyosin alpha-4 chain GN=TPM4	sp|P67936-2|TPM4_HUMAN	32,723.70	100.00%	1	2	14	0.02180%	23.60%
Tumor tissue	Isoform 2 of Tropomyosin alpha-4 chain GN=TPM4	sp|P67936-2|TPM4_HUMAN	32,723.70	100.00%	1	1	31	0.02370%	25.70%
Tumor tissue	Isoform 2 of Tropomyosin beta chain GN=TPM2	sp|P07951-2|TPM2_HUMAN	32,990.60	100.00%	2	3	51	0.03900%	38.40%
Tumor tissue	Isoform 2 of Tubulin alpha chain-like 3 GN=TUBAL3	sp|A6NHL2|TBAL3_HUMAN	45,517.70	99.40%	1	1	5	0.00383%	6.90%

Biological sample	Protein name	Protein accession numbers	Protein molecular weight (Da)	Protein identification probability	Exclusive unique peptide count	Exclusive unique spectrum count	Total spectrum count	Percentage of total spectra	Percentage sequence coverage
Pap test	Isoform 2 of Tubulin alpha-1A chain GN=TUBA1A	sp|Q71U36-2|TBA1A_HUMAN	46,297.50	100.00%	2	3	27	0.04210%	31.20%
Swab	Isoform 2 of Tubulin alpha-1A chain GN=TUBA1A	sp|Q71U36-2|TBA1A_HUMAN	46,297.50	100.00%	2	3	36	0.02550%	39.20%
Tumor tissue	Isoform 2 of Tubulin alpha-1A chain GN=TUBA1A	sp|Q71U36-2|TBA1A_HUMAN	46,297.50	100.00%	4	7	123	0.09420%	42.10%
Tumor tissue	Isoform 2 of Tubulin-specific chaperone E GN=TBCE	sp|Q15813|TBCE_HUMAN	59,347.70	100.00%	2	2	2	0.00153%	4.33%
Tumor tissue	Isoform 2 of Tumor protein D54 GN=TPD52L2	sp|O43399-4|TPD54_HUMAN	21,450.20	100.00%	1	2	18	0.01380%	69.40%
Tumor tissue	Isoform 2 of Tumor suppressor p53-binding protein 1 GN=TP53BP1	sp|Q12888|TP53B_HUMAN	213,572.20	100.00%	13	13	13	0.00995%	10.90%
Tumor tissue	Isoform 2 of Tyrosine-protein kinase BAZ1B GN=BAZ1B	sp|Q9UIG0|BAZ1B_HUMAN	170,451.40	100.00%	13	14	14	0.01070%	13.30%
Swab	Isoform 2 of Tyrosine-protein phosphatase non-receptor type 6 GN=PTPN6	sp|P29350|PTN6_HUMAN	70,131.80	100.00%	2	3	3	0.00213%	4.69%
Tumor tissue	Isoform 2 of Tyrosine-protein phosphatase non-receptor type 6 GN=PTPN6	sp|P29350|PTN6_HUMAN	67,561.80	100.00%	12	16	19	0.01450%	30.70%
Tumor tissue	Isoform 2 of U2 snRNP-associated SURP motif-containing protein GN=U2SURP	sp|O15042|SR140_HUMAN	118,296.40	100.00%	8	8	9	0.00689%	10.40%
Tumor tissue	Isoform 2 of U4/U6 small nuclear ribonucleoprotein Prp4 GN=PRPF4	sp|O43172|PRP4_HUMAN	58,322.10	100.00%	5	5	5	0.00383%	15.00%
Tumor tissue	Isoform 2 of Ubiquitin carboxyl-terminal hydrolase 14 GN=USP14	sp|P54578|UBP14_HUMAN	56,070.30	100.00%	1	2	12	0.00919%	25.10%
Tumor tissue	Isoform 2 of Ubiquitin carboxyl-terminal hydrolase 47 GN=USP47	sp|Q96K76-2|UBP47_HUMAN	147,226.40	100.00%	3	3	3	0.00230%	3.57%
Tumor tissue	Isoform 2 of Ubiquitin carboxyl-terminal hydrolase 8 GN=USP8	sp|P40818-2|UBP8_HUMAN	115,083.50	100.00%	2	2	2	0.00153%	2.57%
Tumor tissue	Isoform 2 of Ubiquitin carboxyl-terminal hydrolase isozyme L5 GN=UCHL5	sp|Q9Y5K5|UCHL5_HUMAN	36,079.70	100.00%	1	1	3	0.00230%	14.90%
Tumor tissue	Isoform 2 of Ubiquitin conjugation factor E4 A GN=UBE4A	sp|Q14139|UBE4A_HUMAN	122,565.90	100.00%	3	3	3	0.00230%	4.01%

Biological sample	Protein name	Protein accession numbers	Protein molecular weight (Da)	Protein identification probability	Exclusive unique peptide count	Exclusive unique spectrum count	Total spectrum count	Percentage of total spectra	Percentage sequence coverage
Tumor tissue	Isoform 2 of Ubiquitin/ISG15-conjugating enzyme E2 L6 GN=UBE2L6	sp|O14933|UB2L6_HUMAN	17,769.40	100.00%	2	2	2	0.00153%	26.40%
Pap test	Isoform 2 of Ubiquitin-like modifier-activating enzyme 1 GN=UBA1	sp|P22314|UBA1_HUMAN	117,850.30	100.00%	15	19	23	0.03590%	21.40%
Swab	Isoform 2 of Ubiquitin-like modifier-activating enzyme 1 GN=UBA1	sp|P22314|UBA1_HUMAN	117,850.30	100.00%	18	26	43	0.03050%	23.80%
Tumor tissue	Isoform 2 of Ubiquitin-like modifier-activating enzyme 1 GN=UBA1	sp|P22314|UBA1_HUMAN	117,850.30	100.00%	26	48	116	0.08880%	36.40%
Tumor tissue	Isoform 2 of Ubiquitin-like-conjugating enzyme ATG3 GN=ATG3	sp|Q9NT62|ATG3_HUMAN	35,466.90	100.00%	4	4	5	0.00383%	16.10%
Tumor tissue	Isoform 2 of UDP-glucose:glycoprotein glucosyltransferase 1 GN=UGGT1	sp|Q9NYU2|UGGG1_HUMAN	174,982.50	100.00%	22	27	32	0.02450%	19.00%
Tumor tissue	Isoform 2 of Unconventional myosin-XVIIIa GN=MYO18A	sp|Q92614-2|MY18A_HUMAN	196,442.10	100.00%	1	1	19	0.01450%	16.20%
Tumor tissue	Isoform 2 of UPF0317 protein C14orf159, mitochondrial GN=C14orf159	sp|Q7Z3D6|CN159_HUMAN	61,059.20	100.00%	2	3	7	0.00536%	9.18%
Pap test	Isoform 2 of UPF0696 protein C11orf68 GN=C11orf68	sp|Q9H3H3|CK068_HUMAN	31,430.20	99.20%	1	1	1	0.00156%	3.77%
Tumor tissue	Isoform 2 of UPF0696 protein C11orf68 GN=C11orf68	sp|Q9H3H3|CK068_HUMAN	31,517.30	100.00%	4	4	4	0.00306%	15.40%
Tumor tissue	Isoform 2 of Upstream-binding protein 1 GN=UBP1	sp|Q9NZI7|UBIP1_HUMAN	60,491.80	99.90%	1	1	3	0.00230%	9.52%
Tumor tissue	Isoform 2 of Utrophin GN=UTRN	sp|P46939|UTRO_HUMAN	394,466.40	100.00%	15	15	22	0.01680%	8.00%
Tumor tissue	Isoform 2 of UV excision repair protein RAD23 homolog A GN=RAD23A	sp|P54725|RD23A_HUMAN	39,609.10	100.00%	2	2	2	0.00153%	12.70%
Tumor tissue	Isoform 2 of Vacuolar protein sorting-associated protein 41 homolog GN=VPS41	sp|P49754|VPS41_HUMAN	98,569.50	100.00%	3	3	3	0.00230%	4.61%
Tumor tissue	Isoform 2 of Vacuolar protein sorting-associated protein 51 homolog GN=VPS51	sp|Q9UID3|VPS51_HUMAN	72,655.90	100.00%	2	2	2	0.00153%	5.02%

Biological sample	Protein name	Protein accession numbers	Protein molecular weight (Da)	Protein identification probability	Exclusive unique peptide count	Exclusive unique spectrum count	Total spectrum count	Percentage of total spectra	Percentage sequence coverage
Tumor tissue	Isoform 2 of Vacuolar protein-sorting-associated protein 36 GN=VPS36	sp|Q86VN1|VPS36_HUMAN	36,959.00	100.00%	3	5	6	0.00459%	13.40%
Tumor tissue	Isoform 2 of Vacuolar-sorting protein SNF8 GN=SNF8	sp|Q96H20|SNF8_HUMAN	28,865.00	100.00%	2	2	2	0.00153%	10.10%
Tumor tissue	Isoform 2 of Valacyclovir hydrolase GN=BPHL	sp|Q86WA6|BPHL_HUMAN	32,543.40	100.00%	2	2	2	0.00153%	8.76%
Tumor tissue	Isoform 2 of Vesicle-associated membrane protein-associated protein A GN=VAPA	sp|Q9P0L0|VAPA_HUMAN	32,614.20	100.00%	6	9	12	0.00919%	24.50%
Tumor tissue	Isoform 2 of Voltage-dependent anion-selective channel protein 3 GN=VDAC3	sp|Q9Y277|VDAC3_HUMAN	30,791.10	100.00%	4	5	16	0.01220%	33.50%
Tumor tissue	Isoform 2 of V-type proton ATPase 116 kDa subunit a isoform 1 GN=ATP6V0A1	sp|Q93050|VPP1_HUMAN	96,603.80	100.00%	1	1	2	0.00153%	4.57%
Swab	Isoform 2 of V-type proton ATPase catalytic subunit A GN=ATP6V1A	sp|P38606|VATA_HUMAN	64,737.40	100.00%	6	7	9	0.00638%	17.10%
Tumor tissue	Isoform 2 of V-type proton ATPase catalytic subunit A GN=ATP6V1A	sp|P38606|VATA_HUMAN	68,305.50	100.00%	12	15	30	0.02300%	35.80%
Swab	Isoform 2 of V-type proton ATPase subunit F GN=ATP6V1F	sp|Q16864|VATF_HUMAN	16,402.20	99.30%	1	1	4	0.00283%	8.16%
Tumor tissue	Isoform 2 of V-type proton ATPase subunit F GN=ATP6V1F	sp|Q16864|VATF_HUMAN	16,402.20	100.00%	3	3	3	0.00230%	27.20%
Swab	Isoform 2 of V-type proton ATPase subunit H GN=ATP6V1H	sp|Q9UI12|VATH_HUMAN	55,883.40	100.00%	3	3	4	0.00283%	13.30%
Tumor tissue	Isoform 2 of V-type proton ATPase subunit H GN=ATP6V1H	sp|Q9UI12|VATH_HUMAN	54,151.60	100.00%	6	7	9	0.00689%	20.60%
Tumor tissue	Isoform 2 of WD repeat-containing protein 44 GN=WDR44	sp|Q5JSH3|WDR44_HUMAN	101,368.60	100.00%	1	1	7	0.00536%	9.06%
Tumor tissue	Isoform 2 of WW domain-containing adapter protein with coiled-coil GN=WAC	sp|Q9BTA9|WAC_HUMAN	65,482.00	100.00%	3	3	3	0.00230%	11.00%
Tumor tissue	Isoform 2 of Zinc finger CCCH domain-containing protein 14 GN=ZC3H14	sp|Q6PJT7|ZC3HE_HUMAN	82,876.10	100.00%	2	2	3	0.00230%	6.12%

Biological sample	Protein name	Protein accession numbers	Protein molecular weight (Da)	Protein identification probability	Exclusive unique peptide count	Exclusive unique spectrum count	Total spectrum count	Percentage of total spectra	Percentage sequence coverage
Tumor tissue	Isoform 2 of Zinc finger CCCH-type with G patch domain-containing protein GN=ZGPAT	sp|Q8N5A5|ZGPAT_HUMAN	57,358.70	100.00%	2	2	2	0.00153%	4.89%
Tumor tissue	Isoform 2 of Zinc transporter ZIP14 GN=SLC39A14	sp|Q15043|S39AE_HUMAN	52,840.10	100.00%	2	2	2	0.00153%	5.20%
Tumor tissue	Isoform 2B of Cytoplasmic dynein 1 intermediate chain 2 GN=DYNC1I2	sp|Q13409|DC1I2_HUMAN	68,297.50	100.00%	6	9	11	0.00842%	19.00%
Tumor tissue	Isoform 2B of GTPase KRas GN=KRAS	sp|P01116-2|RASK_HUMAN	21,424.80	100.00%	1	2	5	0.00383%	18.60%
Tumor tissue	Isoform 2C2A of Collagen alpha-2(VI) chain GN=COL6A2	sp|P12110-2|CO6A2_HUMAN	97,420.50	100.00%	1	1	56	0.04290%	18.80%
Pap test	Isoform 3 of 60 kDa SS-A/Ro ribonucleoprotein GN=TROVE2	sp|P10155|RO60_HUMAN	58,484.30	100.00%	2	2	3	0.00468%	6.95%
Swab	Isoform 3 of 60 kDa SS-A/Ro ribonucleoprotein GN=TROVE2	sp|P10155|RO60_HUMAN	60,170.50	100.00%	2	2	3	0.00213%	5.02%
Tumor tissue	Isoform 3 of 60 kDa SS-A/Ro ribonucleoprotein GN=TROVE2	sp|P10155|RO60_HUMAN	60,672.10	100.00%	4	7	12	0.00919%	18.30%
Swab	Isoform 3 of Acidic leucine-rich nuclear phosphoprotein 32 family member E GN=ANP32E	sp|Q9BTT0|AN32E_HUMAN	25,125.70	100.00%	4	5	7	0.00496%	28.20%
Tumor tissue	Isoform 3 of Acidic leucine-rich nuclear phosphoprotein 32 family member E GN=ANP32E	sp|Q9BTT0|AN32E_HUMAN	30,693.30	100.00%	2	3	3	0.00230%	15.00%
Tumor tissue	Isoform 3 of A-kinase anchor protein 12 GN=AKAP12	sp|Q02952-3|AKA12_HUMAN	180,982.10	100.00%	18	23	35	0.02680%	18.00%
Tumor tissue	Isoform 3 of Alanyl-tRNA editing protein Aarsd1 GN=AARSD1	sp|Q9BTE6-3|AASD1_HUMAN	65,701.60	100.00%	2	2	2	0.00153%	5.97%
Tumor tissue	Isoform 3 of Alpha-actinin-1 GN=ACTN1	sp|P12814-3|ACTN1_HUMAN	105,572.20	100.00%	2	3	260	0.19900%	56.70%
Swab	Isoform 3 of Arf-GAP with Rho-GAP domain, ANK repeat and PH domain-containing protein 1 GN=ARAP1	sp|Q96P48|ARAP1_HUMAN	162,193.00	100.00%	1	1	2	0.00142%	0.90%
Tumor tissue	Isoform 3 of Arf-GAP with Rho-GAP domain, ANK repeat and PH domain-containing protein 1 GN=ARAP1	sp|Q96P48|ARAP1_HUMAN	162,193.00	100.00%	8	9	12	0.00919%	8.83%
Pap test	Isoform 3 of Attractin GN=ATRN	sp|O75882-3|ATRN_HUMAN	133,700.40	100.00%	3	3	3	0.00468%	3.51%

Biological sample	Protein name	Protein accession numbers	Protein molecular weight (Da)	Protein identification probability	Exclusive unique peptide count	Exclusive unique spectrum count	Total spectrum count	Percentage of total spectra	Percentage sequence coverage
Swab	Isoform 3 of Attractin GN=ATRN	sp|O75882-3|ATRN_HUMAN	133,700.40	100.00%	5	6	7	0.00496%	6.01%
Tumor tissue	Isoform 3 of Beta-galactosidase GN=GLB1	sp|P16278|BGAL_HUMAN	72,753.20	100.00%	5	7	12	0.00919%	14.40%
Pap test	Isoform 3 of Beta-glucuronidase GN=GUSB	sp|P08236|BGLR_HUMAN	58,345.50	100.00%	3	3	3	0.00468%	7.13%
Swab	Isoform 3 of Beta-glucuronidase GN=GUSB	sp|P08236|BGLR_HUMAN	58,345.50	100.00%	2	2	2	0.00142%	5.54%
Tumor tissue	Isoform 3 of Beta-glucuronidase GN=GUSB	sp|P08236|BGLR_HUMAN	74,732.80	100.00%	4	4	4	0.00306%	10.70%
Tumor tissue	Isoform 3 of Calcium uniporter protein, mitochondrial GN=MCU	sp|Q8NE86|MCU_HUMAN	39,867.70	100.00%	1	1	5	0.00383%	16.90%
Tumor tissue	Isoform 3 of Calcyclin-binding protein GN=CACYBP	sp|Q9HB71|CYBP_HUMAN	21,228.30	100.00%	6	8	9	0.00689%	58.90%
Pap test	Isoform 3 of Calpastatin GN=CAST	sp|P20810-3|ICAL_HUMAN	63,667.90	99.20%	1	1	19	0.02960%	21.20%
Swab	Isoform 3 of Calumenin GN=CALU	sp|O43852|CALU_HUMAN	38,052.00	100.00%	1	2	2	0.00142%	4.95%
Tumor tissue	Isoform 3 of Calumenin GN=CALU	sp|O43852|CALU_HUMAN	37,107.70	100.00%	1	1	11	0.00842%	39.60%
Tumor tissue	Isoform 3 of CD97 antigen GN=CD97	sp|P48960|CD97_HUMAN	86,628.70	100.00%	5	7	11	0.00842%	9.29%
Tumor tissue	Isoform 3 of Clathrin interactor 1 GN=CLINT1	sp|Q14677|EPN4_HUMAN	70,295.80	100.00%	9	10	11	0.00842%	17.70%
Tumor tissue	Isoform 3 of Cleavage and polyadenylation specificity factor subunit 7 GN=CPSF7	sp|Q8N684-3|CPSF7_HUMAN	56,375.80	100.00%	7	7	7	0.00536%	16.30%
Tumor tissue	Isoform 3 of Cleft lip and palate transmembrane protein 1 GN=CLPTM1	sp|O96005-4|CLPT1_HUMAN	75,179.70	100.00%	2	2	2	0.00153%	6.41%
Pap test	Isoform 3 of Core histone macro-H2A.1 GN=H2AFY	sp|O75367|H2AY_HUMAN	39,618.90	100.00%	3	3	4	0.00624%	13.20%
Swab	Isoform 3 of Core histone macro-H2A.1 GN=H2AFY	sp|O75367|H2AY_HUMAN	39,490.70	100.00%	3	3	3	0.00213%	11.10%
Tumor tissue	Isoform 3 of Core histone macro-H2A.1 GN=H2AFY	sp|O75367|H2AY_HUMAN	39,490.70	100.00%	9	13	28	0.02140%	36.90%
Swab	Isoform 3 of Coronin-1C GN=CORO1C	sp|Q9ULV4-3|COR1C_HUMAN	58,948.90	100.00%	4	4	5	0.00354%	11.60%

Biological sample	Protein name	Protein accession numbers	Protein molecular weight (Da)	Protein identification probability	Exclusive unique peptide count	Exclusive unique spectrum count	Total spectrum count	Percentage of total spectra	Percentage sequence coverage
Tumor tissue	Isoform 3 of Coronin-1C GN=CORO1C	sp|Q9ULV4-3|COR1C_HUMAN	58,948.90	100.00%	10	11	14	0.01070%	17.60%
Swab	Isoform 3 of Coronin-7 GN=CORO7	sp|P57737-3|CORO7_HUMAN	114,166.10	100.00%	3	3	7	0.00496%	4.48%
Tumor tissue	Isoform 3 of Coronin-7 GN=CORO7	sp|P57737-3|CORO7_HUMAN	114,166.10	100.00%	7	9	11	0.00842%	11.50%
Tumor tissue	Isoform 3 of CWF19-like protein 1 GN=CWF19L1	sp|Q69YN2|C19L1_HUMAN	45,537.80	100.00%	4	4	4	0.00306%	13.00%
Tumor tissue	Isoform 3 of Cysteine--tRNA ligase, cytoplasmic GN=CARS	sp|P49589-3|SYCC_HUMAN	94,640.20	100.00%	9	10	11	0.00842%	13.50%
Tumor tissue	Isoform 3 of Cytochrome b reductase 1 GN=CYBRD1	sp|Q53TN4|CYBR1_HUMAN	31,642.40	100.00%	3	3	4	0.00306%	17.50%
Tumor tissue	Isoform 3 of Cytoskeleton-associated protein 5 GN=CKAP5	sp|Q14008-3|CKAP5_HUMAN	226,264.60	100.00%	16	16	18	0.01380%	9.96%
Swab	Isoform 3 of Cytosolic 10-formyltetrahydrofolate dehydrogenase GN=ALDH1L1	sp|O75891|AL1L1_HUMAN	99,754.30	100.00%	4	4	6	0.00425%	7.13%
Pap test	Isoform 3 of Deleted in malignant brain tumors 1 protein GN=DMBT1	sp|Q9UGM3|DMBT1_HUMAN	258,410.40	100.00%	7	9	17	0.02650%	5.58%
Swab	Isoform 3 of Deleted in malignant brain tumors 1 protein GN=DMBT1	sp|Q9UGM3|DMBT1_HUMAN	274,454.10	100.00%	5	7	14	0.00992%	3.33%
Tumor tissue	Isoform 3 of Delta-1-pyrroline-5-carboxylate dehydrogenase, mitochondrial GN=ALDH4A1	sp|P30038|AL4A1_HUMAN	56,043.80	100.00%	3	3	3	0.00230%	8.59%
Tumor tissue	Isoform 3 of DmX-like protein 2 GN=DMXL2	sp|Q8TDJ6|DMXL2_HUMAN	339,641.30	100.00%	2	2	2	0.00153%	1.05%
Tumor tissue	Isoform 3 of DNA ligase 3 GN=LIG3	sp|P49916|DNLI3_HUMAN	112,909.80	100.00%	1	1	6	0.00459%	7.38%
Tumor tissue	Isoform 3 of DNA topoisomerase 2-alpha GN=TOP2A	sp|P11388-3|TOP2A_HUMAN	178,718.70	100.00%	3	3	5	0.00383%	3.83%
Tumor tissue	Isoform 3 of DnaJ homolog subfamily C member 11 GN=DNAJC11	sp|Q9NVH1|DJC11_HUMAN	63,279.20	100.00%	6	7	8	0.00612%	15.40%
Pap test	Isoform 3 of Dolichyl-diphosphooligosaccharide--protein glycosyltransferase 48 kDa subunit GN=DDOST	sp|P39656|OST48_HUMAN	49,021.40	100.00%	2	2	3	0.00468%	5.02%

Biological sample	Protein name	Protein accession numbers	Protein molecular weight (Da)	Protein identification probability	Exclusive unique peptide count	Exclusive unique spectrum count	Total spectrum count	Percentage of total spectra	Percentage sequence coverage
Tumor tissue	Isoform 3 of Dolichyl-diphosphooligosaccharide--protein glycosyltransferase 48 kDa subunit GN=DDOST	sp|P39656|OST48_HUMAN	50,802.50	100.00%	8	10	18	0.01380%	23.10%
Tumor tissue	Isoform 3 of Drebrin GN=DBN1	sp|Q16643|DREB_HUMAN	71,428.60	100.00%	3	4	13	0.00995%	17.80%
Tumor tissue	Isoform 3 of Dynactin subunit 4 GN=DCTN4	sp|Q9UJW0|DCTN4_HUMAN	53,152.60	100.00%	3	3	3	0.00230%	8.35%
Tumor tissue	Isoform 3 of Dystrophin GN=DMD	sp|P11532|DMD_HUMAN	426,748.70	100.00%	1	1	8	0.00612%	2.67%
Tumor tissue	Isoform 3 of EKC/KEOPS complex subunit TPRKB GN=TPRKB	sp|Q9Y3C4|TPRKB_HUMAN	19,662.00	100.00%	2	2	2	0.00153%	14.50%
Tumor tissue	Isoform 3 of Elongation factor 1-delta GN=EEF1D	sp|P29692-3|EF1D_HUMAN	28,557.90	100.00%	1	2	24	0.01840%	38.10%
Tumor tissue	Isoform 3 of Endoplasmic reticulum lectin 1 GN=ERLEC1	sp|Q96DZ1|ERLEC_HUMAN	51,911.00	100.00%	4	4	4	0.00306%	10.70%
Swab	Isoform 3 of Ethylmalonyl-CoA decarboxylase GN=ECHDC1	sp|Q9NTX5-3|ECHD1_HUMAN	24,859.70	100.00%	1	1	4	0.00283%	13.70%
Tumor tissue	Isoform 3 of Ethylmalonyl-CoA decarboxylase GN=ECHDC1	sp|Q9NTX5-3|ECHD1_HUMAN	24,859.70	100.00%	2	2	5	0.00383%	24.30%
Tumor tissue	Isoform 3 of Eukaryotic translation initiation factor 3 subunit J GN=EIF3J	sp|O75822|EIF3J_HUMAN	23,027.30	100.00%	1	1	4	0.00306%	20.60%
Tumor tissue	Isoform 3 of Exosome complex component RRP4 GN=EXOSC2	sp|Q13868|EXOS2_HUMAN	29,408.50	100.00%	2	2	2	0.00153%	7.60%
Swab	Isoform 3 of Exportin-2 GN=CSE1L	sp|P55060|XPO2_HUMAN	110,421.90	100.00%	1	1	1	0.00071%	1.48%
Tumor tissue	Isoform 3 of Exportin-2 GN=CSE1L	sp|P55060|XPO2_HUMAN	110,421.90	100.00%	16	20	26	0.01990%	21.50%
Tumor tissue	Isoform 3 of Fermitin family homolog 2 GN=FERMT2	sp|Q96AC1|FERM2_HUMAN	77,863.90	100.00%	2	2	26	0.01990%	20.40%
Tumor tissue	Isoform 3 of Fibronectin GN=FN1	sp|P02751|FINC_HUMAN	259,208.30	100.00%	2	5	414	0.31700%	32.90%
Tumor tissue	Isoform 3 of FYN-binding protein GN=FYB	sp|O15117-3|FYB_HUMAN	91,654.30	100.00%	2	2	2	0.00153%	2.50%
Tumor tissue	Isoform 3 of Gamma-glutamyltransferase 5 GN=GGT5	sp|P36269-3|GGT5_HUMAN	62,331.90	100.00%	5	5	7	0.00536%	10.60%

Biological sample	Protein name	Protein accession numbers	Protein molecular weight (Da)	Protein identification probability	Exclusive unique peptide count	Exclusive unique spectrum count	Total spectrum count	Percentage of total spectra	Percentage sequence coverage
Tumor tissue	Isoform 3 of Glucosamine-6-phosphate isomerase 2 GN=GNPDA2	sp|Q8TDQ7|GNPI2_HUMAN	29,174.30	99.90%	1	1	7	0.00536%	21.20%
Tumor tissue	Isoform 3 of Glutaminase kidney isoform, mitochondrial GN=GLS	sp|O94925-3|GLSK_HUMAN	65,461.10	100.00%	4	4	5	0.00383%	11.90%
Tumor tissue	Isoform 3 of Golgi reassembly-stacking protein 2 GN=GORASP2	sp|Q9H8Y8|GORS2_HUMAN	47,146.20	100.00%	3	5	7	0.00536%	13.40%
Tumor tissue	Isoform 3 of Golgin subfamily A member 4 GN=GOLGA4	sp|Q13439|GOGA4_HUMAN	260,954.70	100.00%	4	4	4	0.00306%	2.25%
Tumor tissue	Isoform 3 of Growth factor receptor-bound protein 7 GN=GRB7	sp|Q14451|GRB7_HUMAN	55,920.20	100.00%	3	3	3	0.00230%	9.37%
Tumor tissue	Isoform 3 of Hematological and neurological expressed 1-like protein GN=HN1L	sp|Q9H910|HN1L_HUMAN	23,026.20	100.00%	1	1	3	0.00230%	17.90%
Tumor tissue	Isoform 3 of Heterogeneous nuclear ribonucleoprotein A/B GN=HNRNPAB	sp|Q99729-3|ROAA_HUMAN	30,588.20	100.00%	1	2	24	0.01840%	31.60%
Pap test	Isoform 3 of Heterogeneous nuclear ribonucleoprotein K GN=HNRNPK	sp|P61978-3|HNRPK_HUMAN	48,564.60	100.00%	5	5	8	0.01250%	15.90%
Swab	Isoform 3 of Heterogeneous nuclear ribonucleoprotein K GN=HNRNPK	sp|P61978-3|HNRPK_HUMAN	48,564.60	100.00%	7	9	18	0.01280%	18.90%
Tumor tissue	Isoform 3 of Heterogeneous nuclear ribonucleoprotein K GN=HNRNPK	sp|P61978-3|HNRPK_HUMAN	48,564.60	100.00%	20	33	79	0.06050%	50.50%
Tumor tissue	Isoform 3 of Histone H2A.V GN=H2AFV	sp|Q71UI9-3|H2AV_HUMAN	9,375.50	100.00%	2	5	8	0.00612%	34.40%
Swab	Isoform 3 of Histone-binding protein RBBP4 GN=RBBP4	sp|Q09028|RBBP4_HUMAN	46,158.50	99.80%	1	1	1	0.00071%	6.59%
Tumor tissue	Isoform 3 of Histone-binding protein RBBP4 GN=RBBP4	sp|Q09028|RBBP4_HUMAN	46,158.50	100.00%	2	3	10	0.00766%	17.10%
Tumor tissue	Isoform 3 of Hsp70-binding protein 1 GN=HSPBP1	sp|Q9NZL4|HPBP1_HUMAN	39,473.90	100.00%	2	2	2	0.00153%	6.67%
Pap test	Isoform 3 of Hydroxyacyl-coenzyme A dehydrogenase, mitochondrial GN=HADH	sp|Q16836|HCDH_HUMAN	36,052.10	99.40%	1	1	1	0.00156%	3.02%

Biological sample	Protein name	Protein accession numbers	Protein molecular weight (Da)	Protein identification probability	Exclusive unique peptide count	Exclusive unique spectrum count	Total spectrum count	Percentage of total spectra	Percentage sequence coverage
Swab	Isoform 3 of Hydroxyacyl-coenzyme A dehydrogenase, mitochondrial GN=HADH	sp|Q16836|HCDH_HUMAN	34,294.20	100.00%	1	1	1	0.00071%	3.93%
Tumor tissue	Isoform 3 of Hydroxyacyl-coenzyme A dehydrogenase, mitochondrial GN=HADH	sp|Q16836|HCDH_HUMAN	36,052.10	100.00%	5	8	14	0.01070%	22.40%
Pap test	Isoform 3 of Importin-5 GN=IPO5	sp|O00410|IPO5_HUMAN	123,632.20	99.20%	1	1	1	0.00156%	1.79%
Swab	Isoform 3 of Importin-5 GN=IPO5	sp|O00410|IPO5_HUMAN	125,547.30	100.00%	1	1	1	0.00071%	1.43%
Tumor tissue	Isoform 3 of Importin-5 GN=IPO5	sp|O00410|IPO5_HUMAN	123,632.20	100.00%	6	10	19	0.01450%	13.00%
Pap test	Isoform 3 of Inositol monophosphatase 1 GN=IMPA1	sp|P29218|IMPA1_HUMAN	36,694.20	100.00%	4	4	4	0.00624%	17.30%
Swab	Isoform 3 of Inositol monophosphatase 1 GN=IMPA1	sp|P29218|IMPA1_HUMAN	30,188.50	100.00%	4	4	4	0.00283%	16.40%
Tumor tissue	Isoform 3 of Inositol monophosphatase 1 GN=IMPA1	sp|P29218|IMPA1_HUMAN	36,694.20	100.00%	4	4	4	0.00306%	15.50%
Tumor tissue	Isoform 3 of Integrin alpha-V GN=ITGAV	sp|P06756-3|ITAV_HUMAN	111,134.50	100.00%	12	14	16	0.01220%	16.00%
Tumor tissue	Isoform 3 of Interleukin-1 receptor accessory protein GN=IL1RAP	sp|Q9NPH3-3|IL1AP_HUMAN	39,744.10	99.40%	1	1	2	0.00153%	8.38%
Tumor tissue	Isoform 3 of IQ motif and SEC7 domain-containing protein 2 GN=IQSEC2	sp|Q5JU85-3|IQEC2_HUMAN	105,102.70	100.00%	1	1	2	0.00153%	2.63%
Tumor tissue	Isoform 3 of Isobutyryl-CoA dehydrogenase, mitochondrial GN=ACAD8	sp|Q9UKU7-3|ACAD8_HUMAN	38,359.30	100.00%	2	2	2	0.00153%	6.44%
Tumor tissue	Isoform 3 of IST1 homolog GN=IST1	sp|P53990-3|IST1_HUMAN	39,929.50	99.90%	2	2	2	0.00153%	5.00%
Tumor tissue	Isoform 3 of KN motif and ankyrin repeat domain-containing protein 2 GN=KANK2	sp|Q63ZY3|KANK2_HUMAN	90,043.70	100.00%	10	12	17	0.01300%	16.80%
Swab	Isoform 3 of Kynurenine--oxoglutarate transaminase 3 GN=KYAT3	sp|Q6YP21|KAT3_HUMAN	51,402.70	98.80%	1	2	2	0.00142%	2.86%

Biological sample	Protein name	Protein accession numbers	Protein molecular weight (Da)	Protein identification probability	Exclusive unique peptide count	Exclusive unique spectrum count	Total spectrum count	Percentage of total spectra	Percentage sequence coverage
Tumor tissue	Isoform 3 of Kynurenine--oxoglutarate transaminase 3 GN=KYAT3	sp|Q6YP21|KAT3_HUMAN	51,402.70	100.00%	3	3	3	0.00230%	8.33%
Tumor tissue	Isoform 3 of Liver carboxylesterase 1 GN=CES1	sp|P23141-3|EST1_HUMAN	62,394.30	100.00%	11	13	15	0.01150%	26.10%
Pap test	Isoform 3 of L-lactate dehydrogenase A chain GN=LDHA	sp|P00338-3|LDHA_HUMAN	39,838.00	100.00%	8	11	18	0.02810%	30.50%
Swab	Isoform 3 of L-lactate dehydrogenase A chain GN=LDHA	sp|P00338-3|LDHA_HUMAN	39,838.00	100.00%	12	22	52	0.03690%	34.10%
Tumor tissue	Isoform 3 of L-lactate dehydrogenase A chain GN=LDHA	sp|P00338-3|LDHA_HUMAN	39,838.00	100.00%	13	28	63	0.04820%	32.40%
Pap test	Isoform 3 of Malate dehydrogenase, cytoplasmic GN=MDH1	sp|P40925|MDHC_HUMAN	38,628.50	99.90%	1	1	4	0.00624%	8.24%
Swab	Isoform 3 of Malate dehydrogenase, cytoplasmic GN=MDH1	sp|P40925|MDHC_HUMAN	38,628.50	100.00%	3	5	22	0.01560%	30.10%
Tumor tissue	Isoform 3 of Malate dehydrogenase, cytoplasmic GN=MDH1	sp|P40925|MDHC_HUMAN	38,628.50	100.00%	2	3	20	0.01530%	33.50%
Tumor tissue	Isoform 3 of Mediator of DNA damage checkpoint protein 1 GN=MDC1	sp|Q14676|MDC1_HUMAN	195,982.60	100.00%	10	10	11	0.00842%	10.10%
Tumor tissue	Isoform 3 of Methionine aminopeptidase 2 GN=METAP2	sp|P50579-3|MAP2_HUMAN	50,497.60	100.00%	3	3	4	0.00306%	14.30%
Pap test	Isoform 3 of Mitochondrial peptide methionine sulfoxide reductase GN=MSRA	sp|Q9UJ68|MSRA_HUMAN	23,626.60	100.00%	2	2	2	0.00312%	15.60%
Swab	Isoform 3 of Mitochondrial peptide methionine sulfoxide reductase GN=MSRA	sp|Q9UJ68|MSRA_HUMAN	23,626.60	99.90%	1	1	2	0.00142%	7.81%
Tumor tissue	Isoform 3 of Mitochondrial peptide methionine sulfoxide reductase GN=MSRA	sp|Q9UJ68|MSRA_HUMAN	21,539.30	99.70%	1	1	1	0.00077%	6.77%
Tumor tissue	Isoform 3 of Myosin-10 GN=MYH10	sp|P35580|MYH10_HUMAN	229,005.30	100.00%	61	108	228	0.17500%	42.10%

Biological sample	Protein name	Protein accession numbers	Protein molecular weight (Da)	Protein identification probability	Exclusive unique peptide count	Exclusive unique spectrum count	Total spectrum count	Percentage of total spectra	Percentage sequence coverage
Tumor tissue	Isoform 3 of Neuroplastin GN=NPTN	sp|Q9Y639-5|NPTN_HUMAN	30,835.40	100.00%	3	3	3	0.00230%	10.10%
Tumor tissue	Isoform 3 of NFU1 iron-sulfur cluster scaffold homolog, mitochondrial GN=NFU1	sp|Q9UMS0|NFU1_HUMAN	28,463.20	100.00%	2	2	3	0.00230%	17.00%
Pap test	Isoform 3 of NSFL1 cofactor p47 GN=NSFL1C	sp|Q9UNZ2|NSF1C_HUMAN	40,816.40	100.00%	6	8	8	0.01250%	23.10%
Swab	Isoform 3 of NSFL1 cofactor p47 GN=NSFL1C	sp|Q9UNZ2|NSF1C_HUMAN	40,573.20	100.00%	3	4	6	0.00425%	13.40%
Tumor tissue	Isoform 3 of NSFL1 cofactor p47 GN=NSFL1C	sp|Q9UNZ2|NSF1C_HUMAN	40,816.40	100.00%	8	8	12	0.00919%	32.30%
Tumor tissue	Isoform 3 of Nuclear cap-binding protein subunit 2 GN=NCBP2	sp|P52298-3|NCBP2_HUMAN	11,932.60	100.00%	1	1	3	0.00230%	29.10%
Tumor tissue	Isoform 3 of Nucleoporin p54 GN=NUP54	sp|Q7Z3B4|NUP54_HUMAN	55,436.20	100.00%	8	9	9	0.00689%	23.10%
Tumor tissue	Isoform 3 of Peptidyl-prolyl cis-trans isomerase FKBP9 GN=FKBP9	sp|O95302-3|FKBP9_HUMAN	69,155.70	100.00%	5	5	7	0.00536%	9.15%
Pap test	Isoform 3 of Plasma protease C1 inhibitor GN=SERPING1	sp|P05155|IC1_HUMAN	55,770.20	100.00%	1	2	70	0.10900%	24.00%
Tumor tissue	Isoform 3 of Plasma protease C1 inhibitor GN=SERPING1	sp|P05155|IC1_HUMAN	55,770.20	100.00%	1	2	17	0.01300%	22.00%
Tumor tissue	Isoform 3 of Pleckstrin homology domain-containing family G member 3 GN=PLEKHG3	sp|A1L390|PKHG3_HUMAN	134,413.00	100.00%	4	4	4	0.00306%	5.16%
Pap test	Isoform 3 of Plectin GN=PLEC	sp|Q15149-3|PLEC_HUMAN	518,026.90	100.00%	1	1	38	0.05930%	8.29%
Tumor tissue	Isoform 3 of Plectin GN=PLEC	sp|Q15149-3|PLEC_HUMAN	518,026.90	100.00%	2	3	421	0.32200%	45.60%
Tumor tissue	Isoform 3 of Pre-B-cell leukemia transcription factor 1 GN=PBX1	sp|P40424-3|PBX1_HUMAN	46,265.00	100.00%	1	4	8	0.00612%	21.70%
Tumor tissue	Isoform 3 of Proteasome activator complex subunit 3 GN=PSME3	sp|P61289|PSME3_HUMAN	29,507.30	100.00%	4	5	7	0.00536%	25.30%
Tumor tissue	Isoform 3 of Protein 4.1 GN=EPB41	sp|P11171|41_HUMAN	97,018.50	99.90%	1	1	3	0.00230%	4.06%
Swab	Isoform 3 of Protein DDI1 homolog 2 GN=DDI2	sp|Q5TDH0|DDI2_HUMAN	46,571.90	99.90%	1	1	1	0.00071%	4.53%

Biological sample	Protein name	Protein accession numbers	Protein molecular weight (Da)	Protein identification probability	Exclusive unique peptide count	Exclusive unique spectrum count	Total spectrum count	Percentage of total spectra	Percentage sequence coverage
Tumor tissue	Isoform 3 of Protein DDI1 homolog 2 GN=DDI2	sp|Q5TDH0|DDI2_HUMAN	46,571.90	100.00%	3	3	3	0.00230%	15.30%
Tumor tissue	Isoform 3 of Protein numb homolog GN=NUMB	sp|P49757|NUMB_HUMAN	70,802.50	99.90%	1	1	2	0.00153%	5.62%
Pap test	Isoform 3 of Protein phosphatase 1A GN=PPM1A	sp|P35813|PPM1A_HUMAN	51,366.40	99.70%	1	1	1	0.00156%	3.96%
Swab	Isoform 3 of Protein phosphatase 1A GN=PPM1A	sp|P35813|PPM1A_HUMAN	51,366.40	100.00%	1	2	4	0.00283%	6.59%
Tumor tissue	Isoform 3 of Protein phosphatase 1A GN=PPM1A	sp|P35813|PPM1A_HUMAN	42,447.40	100.00%	3	4	8	0.00612%	15.40%
Tumor tissue	Isoform 3 of Protein unc-13 homolog D GN=UNC13D	sp|Q70J99|UN13D_HUMAN	128,820.90	100.00%	3	3	3	0.00230%	3.77%
Tumor tissue	Isoform 3 of Protein-methionine sulfoxide oxidase MICAL2 GN=MICAL2	sp|O94851|MICA2_HUMAN	126,691.60	100.00%	2	2	3	0.00230%	2.81%
Tumor tissue	Isoform 3 of Pyrroline-5-carboxylate reductase 1, mitochondrial GN=PYCR1	sp|P32322|P5CR1_HUMAN	33,361.10	100.00%	8	11	11	0.00842%	31.80%
Tumor tissue	Isoform 3 of Ral GTPase-activating protein subunit beta GN=RALGAPB	sp|Q86X10|RLGPB_HUMAN	166,801.60	100.00%	1	1	2	0.00153%	3.29%
Tumor tissue	Isoform 3 of RasGAP-activating-like protein 1 GN=RASAL1	sp|O95294-3|RASL1_HUMAN	90,275.20	100.00%	2	2	2	0.00153%	3.72%
Tumor tissue	Isoform 3 of Regulatory-associated protein of mTOR GN=RPTOR	sp|Q8N122|RPTOR_HUMAN	149,039.20	100.00%	2	2	2	0.00153%	2.46%
Tumor tissue	Isoform 3 of Rho GTPase-activating protein 25 GN=ARHGAP25	sp|P42331|RHG25_HUMAN	72,503.30	100.00%	4	4	4	0.00306%	8.46%
Tumor tissue	Isoform 3 of Serine hydroxymethyltransferase, mitochondrial GN=SHMT2	sp|P34897-3|GLYM_HUMAN	53,456.50	100.00%	1	1	26	0.01990%	36.00%
Tumor tissue	Isoform 3 of Serine/threonine-protein phosphatase 4 regulatory subunit 2 GN=PPP4R2	sp|Q9NY27|PP4R2_HUMAN	46,897.40	100.00%	3	3	3	0.00230%	12.20%
Tumor tissue	Isoform 3 of Shootin-1 GN=SHTN1	sp|A0MZ66|SHOT1_HUMAN	73,610.10	100.00%	6	6	7	0.00536%	11.20%
Pap test	Isoform 3 of SPATS2-like protein GN=SPATS2L	sp|Q9NUQ6|SPS2L_HUMAN	62,654.60	99.30%	1	1	2	0.00312%	4.25%

Biological sample	Protein name	Protein accession numbers	Protein molecular weight (Da)	Protein identification probability	Exclusive unique peptide count	Exclusive unique spectrum count	Total spectrum count	Percentage of total spectra	Percentage sequence coverage
Tumor tissue	Isoform 3 of SPATS2-like protein GN=SPATS2L	sp|Q9NUQ6|SPS2L_HUMAN	65,152.60	100.00%	6	6	8	0.00612%	19.40%
Swab	Isoform 3 of Spermidine/spermine N(1)-acetyltransferase-like protein 1 GN=SATL1	sp|Q86VE3-3|SATL1_HUMAN	68,529.20	100.00%	2	2	2	0.00142%	5.38%
Tumor tissue	Isoform 3 of Splicing factor, arginine/serine-rich 15 GN=SCAF4	sp|O95104|SFR15_HUMAN	125,868.10	100.00%	2	2	4	0.00306%	6.54%
Swab	Isoform 3 of Synembryn-A GN=RIC8A	sp|Q9NPQ8|RIC8A_HUMAN	60,372.80	99.80%	1	1	1	0.00071%	3.54%
Tumor tissue	Isoform 3 of Synembryn-A GN=RIC8A	sp|Q9NPQ8|RIC8A_HUMAN	60,372.80	100.00%	7	8	11	0.00842%	19.00%
Tumor tissue	Isoform 3 of THO complex subunit 6 homolog GN=THOC6	sp|Q86W42|THOC6_HUMAN	37,535.00	100.00%	2	3	5	0.00383%	11.10%
Tumor tissue	Isoform 3 of Transcription activator BRG1 GN=SMARCA4	sp|P51532-5|SMCA4_HUMAN	181,607.50	100.00%	2	2	10	0.00766%	6.99%
Tumor tissue	Isoform 3 of Transformer-2 protein homolog alpha GN=TRA2A	sp|Q13595|TRA2A_HUMAN	20,590.40	100.00%	2	2	5	0.00383%	17.70%
Tumor tissue	Isoform 3 of Transformer-2 protein homolog beta GN=TRA2B	sp|P62995|TRA2B_HUMAN	21,936.00	100.00%	3	4	7	0.00536%	26.10%
Tumor tissue	Isoform 3 of Transmembrane emp24 domain-containing protein 4 GN=TMED4	sp|Q7Z7H5|TMED4_HUMAN	20,781.00	100.00%	3	4	4	0.00306%	21.00%
Tumor tissue	Isoform 3 of Transmembrane prolyl 4-hydroxylase GN=P4HTM	sp|Q9NXG6|P4HTM_HUMAN	56,660.90	100.00%	2	2	2	0.00153%	7.64%
Tumor tissue	Isoform 3 of Transmembrane protein 87A GN=TMEM87A	sp|Q8NBN3|TM87A_HUMAN	63,432.70	100.00%	2	2	2	0.00153%	6.28%
Tumor tissue	Isoform 3 of Transportin-3 GN=TNPO3	sp|Q9Y5L0|TNPO3_HUMAN	102,541.30	100.00%	5	6	7	0.00536%	9.90%
Tumor tissue	Isoform 3 of Treacle protein GN=TCOF1	sp|Q13428|TCOF_HUMAN	152,203.50	100.00%	8	8	8	0.00612%	7.39%
Tumor tissue	Isoform 3 of Tumor protein D52 GN=TPD52	sp|P55327-3|TPD52_HUMAN	26,382.50	100.00%	1	2	16	0.01220%	39.50%
Tumor tissue	Isoform 3 of Type I inositol 3,4-bisphosphate 4-phosphatase GN=INPP4A	sp|Q96PE3-3|INP4A_HUMAN	109,346.00	100.00%	3	3	3	0.00230%	4.42%

Biological sample	Protein name	Protein accession numbers	Protein molecular weight (Da)	Protein identification probability	Exclusive unique peptide count	Exclusive unique spectrum count	Total spectrum count	Percentage of total spectra	Percentage sequence coverage
Tumor tissue	Isoform 3 of Ubiquitin carboxyl-terminal hydrolase 10 GN=USP10	sp|Q14694-3|UBP10_HUMAN	87,534.20	100.00%	6	7	7	0.00536%	11.20%
Pap test	Isoform 3 of Ubiquitin-conjugating enzyme E2 L3 GN=UBE2L3	sp|P68036-3|UB2L3_HUMAN	24,004.40	99.50%	1	1	2	0.00312%	7.08%
Swab	Isoform 3 of Ubiquitin-conjugating enzyme E2 L3 GN=UBE2L3	sp|P68036-3|UB2L3_HUMAN	24,004.40	100.00%	3	3	3	0.00213%	20.80%
Tumor tissue	Isoform 3 of Ubiquitin-conjugating enzyme E2 L3 GN=UBE2L3	sp|P68036-3|UB2L3_HUMAN	24,004.40	100.00%	4	8	20	0.01530%	22.60%
Tumor tissue	Isoform 3 of Vascular cell adhesion protein 1 GN=VCAM1	sp|P19320|VCAM1_HUMAN	81,277.80	100.00%	2	2	2	0.00153%	4.43%
Pap test	Isoform 3 of Vitamin D-binding protein GN=GC	sp|P02774|VTDB_HUMAN	55,124.10	100.00%	2	3	87	0.13600%	45.00%
Swab	Isoform 3 of Vitamin D-binding protein GN=GC	sp|P02774|VTDB_HUMAN	55,124.10	100.00%	1	1	152	0.10800%	51.90%
Tumor tissue	Isoform 3 of WD repeat-containing protein 48 GN=WDR48	sp|Q8TAF3|WDR48_HUMAN	75,349.50	100.00%	2	2	2	0.00153%	3.44%
Pap test	Isoform 3 of Xaa-Pro aminopeptidase 1 GN=XPNPEP1	sp|Q9NQW7-3|XPP1_HUMAN	74,799.60	99.20%	1	1	1	0.00156%	2.10%
Swab	Isoform 3 of Xaa-Pro aminopeptidase 1 GN=XPNPEP1	sp|Q9NQW7-3|XPP1_HUMAN	74,799.60	100.00%	3	3	3	0.00213%	6.16%
Tumor tissue	Isoform 3 of Xaa-Pro aminopeptidase 1 GN=XPNPEP1	sp|Q9NQW7-3|XPP1_HUMAN	74,799.60	100.00%	11	12	12	0.00919%	24.20%
Tumor tissue	Isoform 3 of Y-box-binding protein 3 GN=YBX3	sp|P16989|YBOX3_HUMAN	37,020.30	100.00%	3	4	12	0.00919%	39.80%
Pap test	Isoform 3 of Zinc finger protein 185 GN=ZNF185	sp|O15231|ZN185_HUMAN	73,525.40	100.00%	1	1	6	0.00936%	9.71%
Tumor tissue	Isoform 3 of Zinc finger protein 638 GN=ZNF638	sp|Q14966-3|ZN638_HUMAN	218,246.70	100.00%	2	2	2	0.00153%	1.28%
Tumor tissue	Isoform 4 of 26S proteasome non-ATPase regulatory subunit 6 GN=PSMD6	sp|Q15008-4|PSMD6_HUMAN	51,924.90	100.00%	3	5	8	0.00612%	14.30%
Tumor tissue	Isoform 4 of Acyl-coenzyme A thioesterase 9, mitochondrial GN=ACOT9	sp|Q9Y305-4|ACOT9_HUMAN	50,851.40	100.00%	7	8	12	0.00919%	21.90%
Tumor tissue	Isoform 4 of Ankycorbin GN=RAI14	sp|Q9P0K7|RAI14_HUMAN	110,043.30	100.00%	18	18	19	0.01450%	25.90%

Biological sample	Protein name	Protein accession numbers	Protein molecular weight (Da)	Protein identification probability	Exclusive unique peptide count	Exclusive unique spectrum count	Total spectrum count	Percentage of total spectra	Percentage sequence coverage
Pap test	Isoform 4 of Apolipoprotein B receptor GN=APOBR	sp|Q0VD83|APOBR_HUMAN	115,630.30	99.90%	1	1	1	0.00156%	1.28%
Swab	Isoform 4 of Apolipoprotein B receptor GN=APOBR	sp|Q0VD83|APOBR_HUMAN	114,870.60	100.00%	1	1	1	0.00071%	2.92%
Tumor tissue	Isoform 4 of Apolipoprotein B receptor GN=APOBR	sp|Q0VD83|APOBR_HUMAN	114,870.60	100.00%	4	4	4	0.00306%	6.56%
Tumor tissue	Isoform 4 of Basic leucine zipper and W2 domain-containing protein 1 GN=BZW1	sp|Q7L1Q6-4|BZW1_HUMAN	48,509.30	100.00%	1	1	8	0.00612%	10.40%
Tumor tissue	Isoform 4 of Cadherin-13 GN=CDH13	sp|P55290|CAD13_HUMAN	78,287.60	100.00%	2	2	2	0.00153%	3.42%
Pap test	Isoform 4 of Chitotriosidase-1 GN=CHIT1	sp|Q13231-4|CHIT1_HUMAN	49,613.50	100.00%	1	1	11	0.01720%	20.10%
Swab	Isoform 4 of Chitotriosidase-1 GN=CHIT1	sp|Q13231-4|CHIT1_HUMAN	49,613.50	100.00%	1	1	9	0.00638%	15.40%
Tumor tissue	Isoform 4 of CUGBP Elav-like family member 1 GN=CELF1	sp|Q92879-4|CELF1_HUMAN	55,001.60	99.50%	1	1	3	0.00230%	7.81%
Tumor tissue	Isoform 4 of Cytosolic acyl coenzyme A thioester hydrolase GN=ACOT7	sp|O00154|BACH_HUMAN	36,567.00	100.00%	5	7	7	0.00536%	22.20%
Tumor tissue	Isoform 4 of Double-stranded RNA-specific adenosine deaminase GN=ADAR	sp|P55265|DSRAD_HUMAN	140,841.10	100.00%	2	3	17	0.01300%	14.00%
Tumor tissue	Isoform 4 of Elastin GN=ELN	sp|P15502-4|ELN_HUMAN	66,104.10	100.00%	1	1	8	0.00612%	7.93%
Pap test	Isoform 4 of Extracellular matrix protein 1 GN=ECM1	sp|Q16610|ECM1_HUMAN	63,562.70	100.00%	2	2	22	0.03430%	26.30%
Swab	Isoform 4 of Extracellular matrix protein 1 GN=ECM1	sp|Q16610|ECM1_HUMAN	60,673.40	100.00%	3	3	6	0.00425%	7.05%
Tumor tissue	Isoform 4 of Extracellular matrix protein 1 GN=ECM1	sp|Q16610|ECM1_HUMAN	63,562.70	100.00%	1	1	5	0.00383%	10.10%
Tumor tissue	Isoform 4 of FYVE and coiled-coil domain-containing protein 1 GN=FYCO1	sp|Q9BQS8-4|FYCO1_HUMAN	168,888.30	100.00%	5	5	5	0.00383%	6.21%

Biological sample	Protein name	Protein accession numbers	Protein molecular weight (Da)	Protein identification probability	Exclusive unique peptide count	Exclusive unique spectrum count	Total spectrum count	Percentage of total spectra	Percentage sequence coverage
Tumor tissue	Isoform 4 of Heterogeneous nuclear ribonucleoprotein L-like GN=HNRNPLL	sp|Q8WVV9|HNRLL_HUMAN	59,647.80	100.00%	5	6	7	0.00536%	16.20%
Tumor tissue	Isoform 4 of Histidine--tRNA ligase, cytoplasmic GN=HARS	sp|P12081|SYHC_HUMAN	54,848.50	100.00%	5	6	9	0.00689%	20.40%
Tumor tissue	Isoform 4 of Inhibitor of nuclear factor kappa-B kinase subunit beta GN=IKBKB	sp|O14920-4|IKKB_HUMAN	79,510.30	100.00%	3	3	3	0.00230%	5.45%
Tumor tissue	Isoform 4 of Inhibitor of nuclear factor kappa-B kinase-interacting protein GN=IKBIP	sp|Q70UQ0-4|IKIP_HUMAN	43,084.80	100.00%	4	6	8	0.00612%	13.50%
Tumor tissue	Isoform 4 of Interleukin enhancer-binding factor 3 GN=ILF3	sp|Q12906-7|ILF3_HUMAN	95,808.40	100.00%	1	1	40	0.03060%	32.20%
Tumor tissue	Isoform 4 of Intraflagellar transport protein 20 homolog GN=IFT20	sp|Q8IY31|IFT20_HUMAN	15,281.10	99.70%	1	1	2	0.00153%	28.10%
Tumor tissue	Isoform 4 of Latent-transforming growth factor beta-binding protein 1 GN=LTBP1	sp|Q14766|LTBP1_HUMAN	186,858.50	100.00%	5	6	6	0.00459%	4.76%
Pap test	Isoform 4 of Leukotriene A-4 hydrolase GN=LTA4H	sp|P09960-4|LKHA4_HUMAN	66,853.80	100.00%	2	2	19	0.02960%	27.90%
Swab	Isoform 4 of Leukotriene A-4 hydrolase GN=LTA4H	sp|P09960-4|LKHA4_HUMAN	66,853.80	100.00%	4	5	42	0.02980%	29.50%
Tumor tissue	Isoform 4 of Leukotriene A-4 hydrolase GN=LTA4H	sp|P09960-4|LKHA4_HUMAN	66,853.80	100.00%	3	4	21	0.01610%	32.50%
Tumor tissue	Isoform 4 of LIM domain and actin-binding protein 1 GN=LIMA1	sp|Q9UHB6|LIMA1_HUMAN	85,224.20	100.00%	10	11	12	0.00919%	18.20%
Tumor tissue	Isoform 4 of Liprin-beta-1 GN=PPFIBP1	sp|Q86W92|LIPB1_HUMAN	110,300.60	100.00%	3	3	17	0.01300%	20.20%
Tumor tissue	Isoform 4 of MAP7 domain-containing protein 1 GN=MAP7D1	sp|Q3KQU3|MA7D1_HUMAN	89,467.60	100.00%	2	2	2	0.00153%	3.59%
Tumor tissue	Isoform 4 of Myosin light chain kinase, smooth muscle GN=MYLK	sp|Q15746|MYLK_HUMAN	210,713.00	100.00%	25	29	42	0.03220%	18.90%
Tumor tissue	Isoform 4 of Nucleolar protein 6 GN=NOL6	sp|Q9H6R4|NOL6_HUMAN	127,594.80	100.00%	3	3	3	0.00230%	3.59%
Swab	Isoform 4 of Perilipin-3 GN=PLIN3	sp|O60664|PLIN3_HUMAN	47,073.60	100.00%	1	1	19	0.01350%	33.20%

Biological sample	Protein name	Protein accession numbers	Protein molecular weight (Da)	Protein identification probability	Exclusive unique peptide count	Exclusive unique spectrum count	Total spectrum count	Percentage of total spectra	Percentage sequence coverage
Tumor tissue	Isoform 4 of Perilipin-3 GN=PLIN3	sp|O60664|PLIN3_HUMAN	47,073.60	100.00%	1	1	29	0.02220%	42.20%
Pap test	Isoform 4 of Platelet-activating factor acetylhydrolase IB subunit beta GN=PAFAH1B2	sp|P68402-4|PA1B2_HUMAN	22,734.00	99.20%	1	1	2	0.00312%	9.41%
Tumor tissue	Isoform 4 of Platelet-activating factor acetylhydrolase IB subunit beta GN=PAFAH1B2	sp|P68402-4|PA1B2_HUMAN	22,734.00	100.00%	2	3	5	0.00383%	15.30%
Tumor tissue	Isoform 4 of Plectin GN=PLEC	sp|Q15149-4|PLEC_HUMAN	516,193.30	100.00%	2	2	422	0.32300%	45.80%
Tumor tissue	Isoform 4 of Pre-mRNA 3'-end-processing factor FIP1 GN=FIP1L1	sp|Q6UN15|FIP1_HUMAN	40,835.10	100.00%	3	3	3	0.00230%	12.20%
Tumor tissue	Isoform 4 of Probable aminopeptidase NPEPL1 GN=NPEPL1	sp|Q8NDH3-4|PEPL1_HUMAN	52,671.30	100.00%	3	6	7	0.00536%	10.90%
Tumor tissue	Isoform 4 of Prolyl 3-hydroxylase 1 GN=P3H1	sp|Q32P28-4|P3H1_HUMAN	78,922.80	100.00%	9	10	15	0.01150%	15.40%
Tumor tissue	Isoform 4 of Prostaglandin G/H synthase 1 GN=PTGS1	sp|P23219-4|PGH1_HUMAN	56,086.30	100.00%	5	7	9	0.00689%	15.90%
Tumor tissue	Isoform 4 of Protein ABHD11 GN=ABHD11	sp|Q8NFV4|ABHDB_HUMAN	34,690.20	100.00%	8	11	11	0.00842%	38.30%
Tumor tissue	Isoform 4 of Protein-methionine sulfoxide oxidase MICAL1 GN=MICAL1	sp|Q8TDZ2|MICA1_HUMAN	119,821.70	100.00%	3	3	3	0.00230%	4.51%
Swab	Isoform 4 of Pyridoxal-dependent decarboxylase domain-containing protein 1 GN=PDXDC1	sp|Q6P996|PDXD1_HUMAN	83,728.90	100.00%	1	1	1	0.00071%	1.84%
Tumor tissue	Isoform 4 of Pyridoxal-dependent decarboxylase domain-containing protein 1 GN=PDXDC1	sp|Q6P996|PDXD1_HUMAN	83,572.70	100.00%	7	9	14	0.01070%	27.20%
Tumor tissue	Isoform 4 of Rabankyrin-5 GN=ANKFY1	sp|Q9P2R3-4|ANFY1_HUMAN	132,823.80	100.00%	7	7	7	0.00536%	8.75%
Tumor tissue	Isoform 4 of Rap1 GTPase-GDP dissociation stimulator 1 GN=RAP1GDS1	sp|P52306|GDS1_HUMAN	66,389.20	100.00%	1	1	7	0.00536%	15.00%

Biological sample	Protein name	Protein accession numbers	Protein molecular weight (Da)	Protein identification probability	Exclusive unique peptide count	Exclusive unique spectrum count	Total spectrum count	Percentage of total spectra	Percentage sequence coverage
Tumor tissue	Isoform 4 of Ras-related protein R-Ras2 GN=RRAS2	sp|P62070|RRAS2_HUMAN	24,195.70	100.00%	2	3	5	0.00383%	24.30%
Pap test	Isoform 4 of Selenium-binding protein 1 GN=SELENBP1	sp|Q13228|SBP1_HUMAN	56,867.20	100.00%	9	9	12	0.01870%	19.60%
Swab	Isoform 4 of Selenium-binding protein 1 GN=SELENBP1	sp|Q13228|SBP1_HUMAN	56,867.20	100.00%	10	10	16	0.01130%	22.20%
Tumor tissue	Isoform 4 of Selenium-binding protein 1 GN=SELENBP1	sp|Q13228|SBP1_HUMAN	56,867.20	100.00%	14	16	21	0.01610%	30.90%
Tumor tissue	Isoform 4 of Serine/threonine-protein kinase MRCK alpha GN=CDC42BPA	sp|Q5VT25|MRCKA_HUMAN	193,035.30	100.00%	1	1	2	0.00153%	1.12%
Pap test	Isoform 4 of Sodium/potassium-transporting ATPase subunit alpha-1 GN=ATP1A1	sp|P05023|AT1A1_HUMAN	112,899.50	100.00%	4	5	8	0.01250%	9.19%
Swab	Isoform 4 of Sodium/potassium-transporting ATPase subunit alpha-1 GN=ATP1A1	sp|P05023|AT1A1_HUMAN	112,899.50	100.00%	1	1	3	0.00213%	3.03%
Tumor tissue	Isoform 4 of Sodium/potassium-transporting ATPase subunit alpha-1 GN=ATP1A1	sp|P05023|AT1A1_HUMAN	112,899.50	100.00%	9	11	30	0.02300%	22.90%
Swab	Isoform 4 of Squamous cell carcinoma antigen recognized by T-cells 3 GN=SART3	sp|Q15020|SART3_HUMAN	109,936.40	99.90%	1	2	2	0.00142%	2.59%
Tumor tissue	Isoform 4 of Squamous cell carcinoma antigen recognized by T-cells 3 GN=SART3	sp|Q15020|SART3_HUMAN	105,585.20	100.00%	7	8	8	0.00612%	10.80%
Tumor tissue	Isoform 4 of Tenascin GN=TNC	sp|P24821-4|TENA_HUMAN	230,863.60	100.00%	1	1	133	0.10200%	30.90%
Tumor tissue	Isoform 4 of TRIO and F-actin-binding protein GN=TRIOBP	sp|Q9H2D6|TARA_HUMAN	243,070.00	100.00%	5	5	5	0.00383%	2.83%
Tumor tissue	Isoform 4 of Tropomyosin alpha-1 chain GN=TPM1	sp|P09493-4|TPM1_HUMAN	32,849.30	100.00%	3	5	58	0.04440%	54.20%
Swab	Isoform 4 of Unconventional myosin-VI GN=MYO6	sp|Q9UM54|MYO6_HUMAN	149,697.10	100.00%	1	1	1	0.00071%	1.17%
Tumor tissue	Isoform 4 of Unconventional myosin-VI GN=MYO6	sp|Q9UM54|MYO6_HUMAN	148,690.90	100.00%	4	5	5	0.00383%	4.05%

Biological sample	Protein name	Protein accession numbers	Protein molecular weight (Da)	Protein identification probability	Exclusive unique peptide count	Exclusive unique spectrum count	Total spectrum count	Percentage of total spectra	Percentage sequence coverage
Tumor tissue	Isoform 4 of YLP motif-containing protein 1 GN=YLPM1	sp|P49750-4|YLPM1_HUMAN	241,647.10	100.00%	6	7	7	0.00536%	4.75%
Tumor tissue	Isoform 5 of Acyl-CoA dehydrogenase family member 10 GN=ACAD10	sp|Q6JQN1|ACD10_HUMAN	122,343.10	100.00%	3	3	4	0.00306%	5.05%
Tumor tissue	Isoform 5 of Anthrax toxin receptor 1 GN=ANTXR1	sp|Q9H6X2|ANTR1_HUMAN	59,206.30	100.00%	2	2	4	0.00306%	8.71%
Tumor tissue	Isoform 5 of AP-3 complex subunit delta-1 GN=AP3D1	sp|O14617|AP3D1_HUMAN	136,654.70	100.00%	4	4	5	0.00383%	4.36%
Tumor tissue	Isoform 5 of Atlastin-2 GN=ATL2	sp|Q8NHH9|ATLA2_HUMAN	66,230.30	100.00%	1	1	6	0.00459%	7.43%
Tumor tissue	Isoform 5 of Caldesmon GN=CALD1	sp|Q05682-5|CALD1_HUMAN	61,213.30	100.00%	1	1	72	0.05510%	44.70%
Tumor tissue	Isoform 5 of Catenin alpha-2 GN=CTNNA2	sp|P26232-5|CTNA2_HUMAN	104,274.70	100.00%	1	2	8	0.00612%	4.79%
Swab	Isoform 5 of EGF-containing fibulin-like extracellular matrix protein 1 GN=EFEMP1	sp|Q12805-5|FBLN3_HUMAN	39,194.00	100.00%	1	1	6	0.00425%	24.20%
Tumor tissue	Isoform 5 of Four and a half LIM domains protein 1 GN=FHL1	sp|Q13642-5|FHL1_HUMAN	33,577.30	100.00%	2	2	2	0.00153%	6.76%
Tumor tissue	Isoform 5 of Microtubule-associated protein RP/EB family member 2 GN=MAPRE2	sp|Q15555-5|MARE2_HUMAN	32,236.30	100.00%	2	4	4	0.00306%	13.00%
Tumor tissue	Isoform 5 of Minor histocompatibility antigen H13 GN=HM13	sp|Q8TCT9-5|HM13_HUMAN	36,816.20	100.00%	2	2	10	0.00766%	16.40%
Swab	Isoform 5 of MMS19 nucleotide excision repair protein homolog GN=MMS19	sp|Q96T76|MMS19_HUMAN	115,707.10	98.80%	1	1	1	0.00071%	1.05%
Tumor tissue	Isoform 5 of MMS19 nucleotide excision repair protein homolog GN=MMS19	sp|Q96T76|MMS19_HUMAN	113,291.30	100.00%	5	5	5	0.00383%	8.47%
Tumor tissue	Isoform 5 of Nuclear pore complex protein Nup214 GN=NUP214	sp|P35658-5|NU214_HUMAN	215,401.10	100.00%	8	8	8	0.00612%	5.97%
Tumor tissue	Isoform 5 of Nuclear pore complex protein Nup98-Nup96 GN=NUP98	sp|P52948|NUP98_HUMAN	197,583.50	100.00%	7	7	8	0.00612%	5.17%

Biological sample	Protein name	Protein accession numbers	Protein molecular weight (Da)	Protein identification probability	Exclusive unique peptide count	Exclusive unique spectrum count	Total spectrum count	Percentage of total spectra	Percentage sequence coverage
Tumor tissue	Isoform 5 of Periostin GN=POSTN	sp|Q15063-5|POSTN_HUMAN	90,428.40	100.00%	1	2	128	0.09800%	41.40%
Tumor tissue	Isoform 5 of Peroxisomal biogenesis factor 19 GN=PEX19	sp|P40855|PEX19_HUMAN	29,259.50	100.00%	1	1	2	0.00153%	19.20%
Pap test	Isoform 5 of Radixin GN=RDX	sp|P35241|RADI_HUMAN	71,050.60	100.00%	1	1	12	0.01870%	13.90%
Tumor tissue	Isoform 5 of Radixin GN=RDX	sp|P35241|RADI_HUMAN	68,565.50	100.00%	5	6	31	0.02370%	21.70%
Tumor tissue	Isoform 5 of Reticulon-4 GN=RTN4	sp|Q9NQC3-5|RTN4_HUMAN	42,274.90	100.00%	1	1	13	0.00995%	29.10%
Tumor tissue	Isoform 5 of Sulfatase-modifying factor 1 GN=SUMF1	sp|Q8NBK3|SUMF1_HUMAN	38,385.40	100.00%	1	1	2	0.00153%	9.04%
Tumor tissue	Isoform 5 of Sushi repeat-containing protein SRPX GN=SRPX	sp|P78539|SRPX_HUMAN	49,382.60	100.00%	4	5	7	0.00536%	11.30%
Tumor tissue	Isoform 5 of Tumor protein D54 GN=TPD52L2	sp|O43399|TPD54_HUMAN	23,786.80	100.00%	1	1	17	0.01300%	59.10%
Pap test	Isoform 5 of Uromodulin GN=UMOD	sp|P07911-5|UROM_HUMAN	73,569.60	100.00%	2	2	2	0.00312%	3.71%
Tumor tissue	Isoform 6 of Agrin GN=AGRN	sp|O00468-6|AGRIN_HUMAN	214,838.00	100.00%	2	2	13	0.00995%	6.26%
Tumor tissue	Isoform 6 of CUGBP Elav-like family member 1 GN=CELF1	sp|Q92879-6|CELF1_HUMAN	51,993.00	100.00%	1	1	3	0.00230%	8.04%
Tumor tissue	Isoform 6 of Extended synaptotagmin-2 GN=ESYT2	sp|A0FGR8-6|ESYT2_HUMAN	104,711.70	100.00%	1	1	14	0.01070%	18.20%
Tumor tissue	Isoform 6 of GTPase-activating protein and VPS9 domain-containing protein 1 GN=GAPVD1	sp|Q14C86-6|GAPD1_HUMAN	166,171.90	100.00%	3	3	3	0.00230%	4.03%
Tumor tissue	Isoform 6 of Inactive tyrosine-protein kinase 7 GN=PTK7	sp|Q13308-6|PTK7_HUMAN	119,197.00	100.00%	14	16	18	0.01380%	18.60%
Tumor tissue	Isoform 6 of Myoferlin GN=MYOF	sp|Q9NZM1|MYOF_HUMAN	233,483.40	100.00%	3	5	79	0.06050%	28.20%
Pap test	Isoform 6 of Myosin-14 GN=MYH14	sp|Q7Z406-6|MYH14_HUMAN	228,667.30	100.00%	3	3	29	0.04530%	15.50%
Swab	Isoform 6 of Myosin-14 GN=MYH14	sp|Q7Z406-6|MYH14_HUMAN	228,667.30	100.00%	1	1	8	0.00567%	3.69%
Tumor tissue	Isoform 6 of Myosin-14 GN=MYH14	sp|Q7Z406-6|MYH14_HUMAN	228,667.30	100.00%	9	11	103	0.07880%	32.50%
Tumor tissue	Isoform 6 of RNA binding protein fox-1 homolog 2 GN=RBFOX2	sp|O43251-8|RFOX2_HUMAN	47,215.20	100.00%	1	1	2	0.00153%	8.22%

Biological sample	Protein name	Protein accession numbers	Protein molecular weight (Da)	Protein identification probability	Exclusive unique peptide count	Exclusive unique spectrum count	Total spectrum count	Percentage of total spectra	Percentage sequence coverage
Pap test	Isoform 6 of Tropomyosin alpha-3 chain GN=TPM3	sp|P06753-6|TPM3_HUMAN	28,923.20	100.00%	2	3	16	0.02500%	18.50%
Swab	Isoform 6 of Tropomyosin alpha-3 chain GN=TPM3	sp|P06753-6|TPM3_HUMAN	28,923.20	100.00%	4	5	31	0.02200%	25.80%
Tumor tissue	Isoform 6 of Tropomyosin alpha-3 chain GN=TPM3	sp|P06753-6|TPM3_HUMAN	28,923.20	100.00%	4	6	29	0.02220%	39.90%
Tumor tissue	Isoform 6 of Tumor necrosis factor receptor superfamily member 6 GN=FAS	sp|P25445|TNR6_HUMAN	37,732.00	100.00%	3	4	4	0.00306%	17.20%
Pap test	Isoform 7 of CD44 antigen GN=CD44	sp|P16070-7|CD44_HUMAN	78,445.50	100.00%	1	1	4	0.00624%	1.68%
Swab	Isoform 7 of CD44 antigen GN=CD44	sp|P16070-7|CD44_HUMAN	78,445.50	100.00%	1	1	2	0.00142%	1.68%
Tumor tissue	Isoform 7 of CD44 antigen GN=CD44	sp|P16070-7|CD44_HUMAN	78,445.50	100.00%	7	10	13	0.00995%	12.20%
Pap test	Isoform 8 of Non-specific lipid-transfer protein GN=SCP2	sp|P22307|NLTP_HUMAN	58,994.60	100.00%	2	2	2	0.00312%	3.82%
Swab	Isoform 8 of Non-specific lipid-transfer protein GN=SCP2	sp|P22307|NLTP_HUMAN	56,497.80	100.00%	2	2	2	0.00142%	4.40%
Tumor tissue	Isoform 8 of Non-specific lipid-transfer protein GN=SCP2	sp|P22307|NLTP_HUMAN	58,994.60	100.00%	5	7	9	0.00689%	11.10%
Tumor tissue	Isoform 8 of Plectin GN=PLEC	sp|Q15149-8|PLEC_HUMAN	513,701.60	100.00%	1	2	420	0.32200%	45.90%
Tumor tissue	Isoform 8 of Ubiquitin carboxyl-terminal hydrolase 48 GN=USP48	sp|Q86UV5|UBP48_HUMAN	119,034.40	100.00%	1	1	2	0.00153%	2.96%
Tumor tissue	Isoform A of GDP-fucose protein O-fucosyltransferase 2 GN=POFUT2	sp|Q9Y2G5|OFUT2_HUMAN	44,084.20	100.00%	2	2	2	0.00153%	5.19%
Tumor tissue	Isoform A of Plasma membrane calcium-transporting ATPase 1 GN=ATP2B1	sp|P20020-2|AT2B1_HUMAN	129,519.60	100.00%	6	6	14	0.01070%	15.20%
Pap test	Isoform A of Protein CutA GN=CUTA	sp|O60888|CUTA_HUMAN	16,832.30	99.20%	1	1	4	0.00624%	7.07%
Swab	Isoform A of Protein CutA GN=CUTA	sp|O60888|CUTA_HUMAN	16,832.30	99.60%	1	1	3	0.00213%	7.07%
Tumor tissue	Isoform A of Protein CutA GN=CUTA	sp|O60888|CUTA_HUMAN	16,832.30	100.00%	3	6	7	0.00536%	21.20%
Tumor tissue	Isoform A of Syntaxin-16 GN=STX16	sp|O14662|STX16_HUMAN	35,299.80	100.00%	1	1	2	0.00153%	7.57%

Biological sample	Protein name	Protein accession numbers	Protein molecular weight (Da)	Protein identification probability	Exclusive unique peptide count	Exclusive unique spectrum count	Total spectrum count	Percentage of total spectra	Percentage sequence coverage
Tumor tissue	Isoform Alpha of Striatin-3 GN=STRN3	sp|Q13033|STRN3_HUMAN	87,211.30	100.00%	3	3	3	0.00230%	6.45%
Tumor tissue	Isoform Alpha-6X1B of Integrin alpha-6 GN=ITGA6	sp|P23229|ITA6_HUMAN	121,668.70	100.00%	1	1	6	0.00459%	6.32%
Pap test	Isoform B of AP-2 complex subunit alpha-1 GN=AP2A1	sp|O95782|AP2A1_HUMAN	105,364.20	100.00%	1	1	1	0.00156%	1.15%
Swab	Isoform B of AP-2 complex subunit alpha-1 GN=AP2A1	sp|O95782|AP2A1_HUMAN	107,548.70	100.00%	1	1	2	0.00142%	2.09%
Tumor tissue	Isoform B of AP-2 complex subunit alpha-1 GN=AP2A1	sp|O95782|AP2A1_HUMAN	107,548.70	100.00%	10	11	21	0.01610%	19.30%
Tumor tissue	Isoform B of Bifunctional 3'-phosphoadenosine 5'-phosphosulfate synthase 2 GN=PAPSS2	sp|O95340|PAPS2_HUMAN	69,501.80	100.00%	6	6	6	0.00459%	12.90%
Tumor tissue	Isoform B of Caspase-10 GN=CASP10	sp|Q92851-4|CASPA_HUMAN	58,994.80	100.00%	3	3	4	0.00306%	9.39%
Swab	Isoform B of Eukaryotic translation initiation factor 4 gamma 1 GN=EIF4G1	sp|Q04637|IF4G1_HUMAN	175,494.20	100.00%	1	1	2	0.00142%	0.96%
Tumor tissue	Isoform B of Eukaryotic translation initiation factor 4 gamma 1 GN=EIF4G1	sp|Q04637|IF4G1_HUMAN	175,494.20	100.00%	6	7	23	0.01760%	12.70%
Tumor tissue	Isoform B of Manganese-transporting ATPase 13A1 GN=ATP13A1	sp|Q9HD20|AT131_HUMAN	132,957.80	100.00%	11	11	12	0.00919%	11.30%
Tumor tissue	Isoform B of Methyl-CpG-binding protein 2 GN=MECP2	sp|P51608-2|MECP2_HUMAN	53,323.80	100.00%	10	11	11	0.00842%	31.30%
Pap test	Isoform B of Phosphate carrier protein, mitochondrial GN=SLC25A3	sp|Q00325|MPCP_HUMAN	39,959.50	100.00%	2	2	2	0.00312%	5.54%
Tumor tissue	Isoform B of Phosphate carrier protein, mitochondrial GN=SLC25A3	sp|Q00325|MPCP_HUMAN	39,959.50	100.00%	4	5	9	0.00689%	10.50%
Tumor tissue	Isoform B of Ras GTPase-activating protein-binding protein 2 GN=G3BP2	sp|Q9UN86|G3BP2_HUMAN	54,120.30	100.00%	4	6	6	0.00459%	16.50%

Biological sample	Protein name	Protein accession numbers	Protein molecular weight (Da)	Protein identification probability	Exclusive unique peptide count	Exclusive unique spectrum count	Total spectrum count	Percentage of total spectra	Percentage sequence coverage
Pap test	Isoform B of Ras-related C3 botulinum toxin substrate 1 GN=RAC1	sp|P63000|RAC1_HUMAN	23,468.00	100.00%	3	3	6	0.00936%	27.50%
Swab	Isoform B of Ras-related C3 botulinum toxin substrate 1 GN=RAC1	sp|P63000|RAC1_HUMAN	23,468.00	100.00%	2	3	9	0.00638%	27.00%
Tumor tissue	Isoform B of Ras-related C3 botulinum toxin substrate 1 GN=RAC1	sp|P63000|RAC1_HUMAN	21,450.60	100.00%	2	6	10	0.00766%	10.90%
Swab	Isoform Beta of Apoptosis regulator BAX GN=BAX	sp|Q07812|BAX_HUMAN	21,185.20	100.00%	1	1	5	0.00354%	26.60%
Tumor tissue	Isoform Beta of Apoptosis regulator BAX GN=BAX	sp|Q07812|BAX_HUMAN	18,129.40	100.00%	1	1	10	0.00766%	32.10%
Tumor tissue	Isoform Beta of E3 ubiquitin-protein ligase TRIM33 GN=TRIM33	sp|Q9UPN9|TRI33_HUMAN	122,532.20	100.00%	2	2	2	0.00153%	2.16%
Pap test	Isoform Beta of Heat shock protein 105 kDa GN=HSPH1	sp|Q92598|HS105_HUMAN	92,117.00	100.00%	1	1	2	0.00312%	3.32%
Swab	Isoform Beta of Heat shock protein 105 kDa GN=HSPH1	sp|Q92598|HS105_HUMAN	96,865.80	100.00%	3	3	6	0.00425%	8.60%
Tumor tissue	Isoform Beta of Heat shock protein 105 kDa GN=HSPH1	sp|Q92598|HS105_HUMAN	92,117.00	100.00%	15	19	24	0.01840%	26.80%
Pap test	Isoform Beta of Tripartite motif-containing protein 29 GN=TRIM29	sp|Q14134|TRI29_HUMAN	63,844.40	100.00%	2	2	2	0.00312%	3.33%
Tumor tissue	Isoform Beta of Tripartite motif-containing protein 29 GN=TRIM29	sp|Q14134|TRI29_HUMAN	65,835.70	100.00%	3	3	3	0.00230%	5.79%
Pap test	Isoform Beta-1 of DNA topoisomerase 2-beta GN=TOP2B	sp|Q02880|TOP2B_HUMAN	182,668.40	99.80%	1	1	1	0.00156%	0.74%
Tumor tissue	Isoform Beta-1 of DNA topoisomerase 2-beta GN=TOP2B	sp|Q02880|TOP2B_HUMAN	182,668.40	100.00%	14	15	17	0.01300%	11.90%
Swab	Isoform Beta-2 of Protein phosphatase 1B GN=PPM1B	sp|O75688-2|PPM1B_HUMAN	42,770.70	100.00%	2	3	5	0.00354%	11.10%
Tumor tissue	Isoform Beta-2 of Protein phosphatase 1B GN=PPM1B	sp|O75688-2|PPM1B_HUMAN	42,770.70	99.90%	1	1	4	0.00306%	8.79%
Pap test	Isoform C of Fibulin-1 GN=FBLN1	sp|P23142-4|FBLN1_HUMAN	74,431.80	100.00%	1	1	26	0.04060%	28.30%
Swab	Isoform C of Fibulin-1 GN=FBLN1	sp|P23142-4|FBLN1_HUMAN	74,431.80	100.00%	1	1	38	0.02690%	31.60%

Biological sample	Protein name	Protein accession numbers	Protein molecular weight (Da)	Protein identification probability	Exclusive unique peptide count	Exclusive unique spectrum count	Total spectrum count	Percentage of total spectra	Percentage sequence coverage
Tumor tissue	Isoform C of Fibulin-1 GN=FBLN1	sp|P23142-4|FBLN1_HUMAN	74,431.80	100.00%	4	5	19	0.01450%	14.90%
Tumor tissue	Isoform C15orf38-AP3S2 of Arpin GN=ARPIN	sp|Q7Z6K5|ARPIN_HUMAN	43,882.60	100.00%	2	2	2	0.00153%	5.33%
Tumor tissue	Isoform CNPI of 2',3'-cyclic-nucleotide 3'-phosphodiesterase GN=CNP	sp|P09543|CN37_HUMAN	45,100.30	100.00%	10	12	13	0.00995%	21.40%
Pap test	Isoform Cytoplasmic of Fumarate hydratase, mitochondrial GN=FH	sp|P07954|FUMH_HUMAN	54,637.80	100.00%	1	1	1	0.00156%	3.00%
Swab	Isoform Cytoplasmic of Fumarate hydratase, mitochondrial GN=FH	sp|P07954|FUMH_HUMAN	54,637.80	100.00%	1	1	1	0.00071%	4.93%
Tumor tissue	Isoform Cytoplasmic of Fumarate hydratase, mitochondrial GN=FH	sp|P07954|FUMH_HUMAN	50,213.30	100.00%	8	11	16	0.01220%	27.80%
Tumor tissue	Isoform Cytoplasmic+peroxisomal of Malonyl-CoA decarboxylase, mitochondrial GN=MLYCD	sp|O95822|DCMC_HUMAN	55,004.40	100.00%	2	2	2	0.00153%	6.83%
Tumor tissue	Isoform D of Protein SON GN=SON	sp|P18583-5|SON_HUMAN	267,034.10	99.90%	1	1	3	0.00230%	1.83%
Tumor tissue	Isoform Del-701 of Signal transducer and activator of transcription 3 GN=STAT3	sp|P40763|STAT3_HUMAN	87,982.50	100.00%	14	17	19	0.01450%	25.90%
Tumor tissue	Isoform DFF35 of DNA fragmentation factor subunit alpha GN=DFFA	sp|O00273-2|DFFA_HUMAN	29,411.00	99.90%	1	1	7	0.00536%	34.70%
Tumor tissue	Isoform Er14 of Ankyrin-1 GN=ANK1	sp|P16157-15|ANK1_HUMAN	200,212.50	100.00%	6	6	7	0.00536%	7.08%
Tumor tissue	Isoform F of Constitutive coactivator of PPAR-gamma-like protein 1 GN=FAM120A	sp|Q9NZB2|F120A_HUMAN	125,273.30	100.00%	6	6	11	0.00842%	9.86%
Tumor tissue	Isoform Gamma-2 of Serine/threonine-protein phosphatase PP1-gamma catalytic subunit GN=PPP1CC	sp|P36873-2|PP1G_HUMAN	38,520.10	100.00%	1	1	10	0.00766%	16.00%
Swab	Isoform GN-1 of Glycogenin-1 GN=GYG1	sp|P46976|GLYG_HUMAN	37,479.00	100.00%	1	1	5	0.00354%	12.90%

Biological sample	Protein name	Protein accession numbers	Protein molecular weight (Da)	Protein identification probability	Exclusive unique peptide count	Exclusive unique spectrum count	Total spectrum count	Percentage of total spectra	Percentage sequence coverage
Tumor tissue	Isoform GN-1 of Glycogenin-1 GN=GYG1	sp|P46976|GLYG_HUMAN	39,384.40	100.00%	1	1	4	0.00306%	12.90%
Pap test	Isoform H7 of Myeloperoxidase GN=MPO	sp|P05164|PERM_HUMAN	87,251.00	100.00%	22	51	236	0.36800%	47.10%
Swab	Isoform H7 of Myeloperoxidase GN=MPO	sp|P05164|PERM_HUMAN	87,251.00	100.00%	11	18	76	0.05390%	27.30%
Tumor tissue	Isoform H7 of Myeloperoxidase GN=MPO	sp|P05164|PERM_HUMAN	87,251.00	100.00%	5	5	6	0.00459%	8.62%
Tumor tissue	Isoform HSGC-2 of Guanylate cyclase soluble subunit beta-1 GN=GUCY1B3	sp|Q02153-2|GCYB1_HUMAN	66,739.60	100.00%	2	2	2	0.00153%	3.92%
Tumor tissue	Isoform IB of Synapsin-1 GN=SYN1	sp|P17600|SYN1_HUMAN	74,111.00	100.00%	3	3	3	0.00230%	8.07%
Tumor tissue	Isoform IIB of Myc box-dependent-interacting protein 1 GN=BIN1	sp|O00499|BIN1_HUMAN	55,174.20	100.00%	3	3	3	0.00230%	10.20%
Tumor tissue	Isoform LCRMP-4 of Dihydropyrimidinase-related protein 3 GN=DPYSL3	sp|Q14195-2|DPYL3_HUMAN	73,912.00	100.00%	3	5	109	0.08340%	43.60%
Pap test	Isoform LMW of Kininogen-1 GN=KNG1	sp|P01042-2|KNG1_HUMAN	47,883.60	100.00%	1	1	48	0.07490%	34.70%
Swab	Isoform LMW of Kininogen-1 GN=KNG1	sp|P01042-2|KNG1_HUMAN	47,883.60	100.00%	1	2	44	0.03120%	37.90%
Tumor tissue	Isoform LMW of Kininogen-1 GN=KNG1	sp|P01042-2|KNG1_HUMAN	47,883.60	100.00%	1	2	7	0.00536%	11.50%
Tumor tissue	Isoform Long of Erythrocyte membrane protein band 4.2 GN=EPB42	sp|P16452|EPB42_HUMAN	69,456.40	100.00%	3	3	3	0.00230%	5.13%
Pap test	Isoform Long of Proteasome subunit alpha type-1 GN=PSMA1	sp|P25786|PSA1_HUMAN	29,556.00	100.00%	5	9	13	0.02030%	21.90%
Swab	Isoform Long of Proteasome subunit alpha type-1 GN=PSMA1	sp|P25786|PSA1_HUMAN	29,556.00	100.00%	3	3	4	0.00283%	11.50%
Tumor tissue	Isoform Long of Proteasome subunit alpha type-1 GN=PSMA1	sp|P25786|PSA1_HUMAN	29,556.00	100.00%	7	11	13	0.00995%	29.40%
Pap test	Isoform long of Serine protease inhibitor Kazal-type 5 GN=SPINK5	sp|Q9NQ38-3|ISK5_HUMAN	124,077.30	100.00%	2	2	2	0.00312%	2.10%

Biological sample	Protein name	Protein accession numbers	Protein molecular weight (Da)	Protein identification probability	Exclusive unique peptide count	Exclusive unique spectrum count	Total spectrum count	Percentage of total spectra	Percentage sequence coverage
Swab	Isoform long of Serine protease inhibitor Kazal-type 5 GN=SPINK5	sp|Q9NQ38-3|ISK5_HUMAN	124,077.30	100.00%	1	1	1	0.00071%	2.38%
Swab	Isoform Long of Ubiquitin fusion degradation protein 1 homolog GN=UFD1L	sp|Q92890|UFD1_HUMAN	38,726.10	100.00%	1	1	1	0.00071%	3.79%
Tumor tissue	Isoform Long of Ubiquitin fusion degradation protein 1 homolog GN=UFD1L	sp|Q92890|UFD1_HUMAN	34,501.20	100.00%	5	7	7	0.00536%	21.60%
Swab	Isoform M1 of Pyruvate kinase PKM GN=PKM	sp|P14618-2|KPYM_HUMAN	58,062.60	100.00%	1	1	176	0.12500%	56.50%
Tumor tissue	Isoform M1 of Pyruvate kinase PKM GN=PKM	sp|P14618-2|KPYM_HUMAN	58,062.60	100.00%	1	1	130	0.09950%	44.30%
Tumor tissue	Isoform Mitochondrial of Lysine--tRNA ligase GN=KARS	sp|Q15046|SYK_HUMAN	68,050.40	100.00%	14	17	20	0.01530%	25.90%
Swab	Isoform Non-brain of Clathrin light chain A GN=CLTA	sp|P09496-2|CLCA_HUMAN	23,662.00	99.80%	1	1	2	0.00142%	9.63%
Tumor tissue	Isoform Non-brain of Clathrin light chain A GN=CLTA	sp|P09496-2|CLCA_HUMAN	23,662.00	100.00%	1	2	5	0.00383%	22.90%
Tumor tissue	Isoform p52Shc of SHC-transforming protein 1 GN=SHC1	sp|P29353|SHC1_HUMAN	51,610.40	100.00%	2	2	3	0.00230%	8.88%
Pap test	Isoform PKP3b of Plakophilin-3 GN=PKP3	sp|Q9Y446|PKP3_HUMAN	87,084.70	100.00%	2	2	2	0.00312%	2.96%
Tumor tissue	Isoform PKP3b of Plakophilin-3 GN=PKP3	sp|Q9Y446|PKP3_HUMAN	87,084.70	100.00%	8	10	11	0.00842%	12.70%
Tumor tissue	Isoform PML-11 of Protein PML GN=PML	sp|P29590-11|PML_HUMAN	92,564.50	100.00%	8	12	23	0.01760%	17.00%
Tumor tissue	Isoform PML-3 of Protein PML GN=PML	sp|P29590-9|PML_HUMAN	70,367.10	100.00%	3	3	15	0.01150%	12.90%
Tumor tissue	Isoform Short of Ancient ubiquitous protein 1 GN=AUP1	sp|Q9Y679|AUP1_HUMAN	45,786.90	100.00%	1	1	9	0.00689%	15.40%
Tumor tissue	Isoform Short of Glycylpeptide N-tetradecanoyltransferase 1 GN=NMT1	sp|P30419|NMT1_HUMAN	56,807.00	100.00%	4	7	7	0.00536%	15.10%
Tumor tissue	Isoform Short of Long-chain-fatty-acid--CoA ligase 4 GN=ACSL4	sp|O60488|ACSL4_HUMAN	79,191.50	100.00%	5	5	7	0.00536%	15.50%

Biological sample	Protein name	Protein accession numbers	Protein molecular weight (Da)	Protein identification probability	Exclusive unique peptide count	Exclusive unique spectrum count	Total spectrum count	Percentage of total spectra	Percentage sequence coverage
Tumor tissue	Isoform Short of Mothers against decapentaplegic homolog 2 GN=SMAD2	sp|Q15796|SMAD2_HUMAN	52,305.90	99.90%	1	1	2	0.00153%	5.49%
Tumor tissue	Isoform Short of Nuclear transcription factor Y subunit alpha GN=NFYA	sp|P23511|NFYA_HUMAN	33,937.40	100.00%	2	2	2	0.00153%	13.20%
Tumor tissue	Isoform Short of Probable global transcription activator SNF2L2 GN=SMARCA2	sp|P51531|SMCA2_HUMAN	179,285.00	100.00%	2	2	11	0.00842%	8.33%
Tumor tissue	Isoform Short of Spectrin beta chain, non-erythrocytic 1 GN=SPTBN1	sp|Q01082-2|SPTB2_HUMAN	253,095.70	100.00%	2	3	93	0.07120%	39.70%
Tumor tissue	Isoform Short of TATA-binding protein-associated factor 2N GN=TAF15	sp|Q92804|RBP56_HUMAN	61,558.80	99.90%	2	3	7	0.00536%	5.26%
Tumor tissue	Isoform Short of Tyrosine-protein kinase SYK GN=SYK	sp|P43405|KSYK_HUMAN	69,511.60	100.00%	4	4	4	0.00306%	10.30%
Pap test	Isoform Short of Ubiquitin carboxyl-terminal hydrolase 5 GN=USP5	sp|P45974|UBP5_HUMAN	93,309.50	100.00%	2	2	2	0.00312%	4.43%
Swab	Isoform Short of Ubiquitin carboxyl-terminal hydrolase 5 GN=USP5	sp|P45974|UBP5_HUMAN	95,788.10	100.00%	6	6	8	0.00567%	10.70%
Tumor tissue	Isoform Short of Ubiquitin carboxyl-terminal hydrolase 5 GN=USP5	sp|P45974|UBP5_HUMAN	93,309.50	100.00%	14	19	25	0.01910%	24.90%
Pap test	Isoform SNAP-23b of Synaptosomal-associated protein 23 GN=SNAP23	sp|O00161|SNP23_HUMAN	23,354.80	99.20%	1	1	1	0.00156%	8.86%
Tumor tissue	Isoform SNAP-23b of Synaptosomal-associated protein 23 GN=SNAP23	sp|O00161|SNP23_HUMAN	23,354.80	100.00%	4	4	5	0.00383%	44.90%
Pap test	Isoform Soluble of Catechol O-methyltransferase GN=COMT	sp|P21964|COMT_HUMAN	30,037.60	100.00%	2	3	3	0.00468%	16.70%
Swab	Isoform Soluble of Catechol O-methyltransferase GN=COMT	sp|P21964|COMT_HUMAN	30,037.60	100.00%	2	3	7	0.00496%	24.00%
Tumor tissue	Isoform SRP40-4 of Serine/arginine-rich splicing factor 5 GN=SRSF5	sp|Q13243|SRSF5_HUMAN	31,264.80	100.00%	2	3	4	0.00306%	14.10%
Tumor tissue	Isoform SRP55-3 of Serine/arginine-rich splicing factor 6 GN=SRSF6	sp|Q13247|SRSF6_HUMAN	39,588.40	100.00%	2	4	9	0.00689%	17.30%

Biological sample	Protein name	Protein accession numbers	Protein molecular weight (Da)	Protein identification probability	Exclusive unique peptide count	Exclusive unique spectrum count	Total spectrum count	Percentage of total spectra	Percentage sequence coverage
Tumor tissue	Isoform SV4 of Supervillin GN=SVIL	sp|O95425-4|SVIL_HUMAN	244,523.60	100.00%	1	1	21	0.01610%	12.10%
Tumor tissue	Isoleucine--tRNA ligase, cytoplasmic GN=IARS PE=1 SV=2	SYIC_HUMAN	144,501.80	100.00%	16	18	22	0.01680%	16.80%
Tumor tissue	Isoleucine--tRNA ligase, mitochondrial GN=IARS2 PE=1 SV=2	SYIM_HUMAN	113,794.10	100.00%	13	14	14	0.01070%	18.10%
Tumor tissue	Isovaleryl-CoA dehydrogenase, mitochondrial GN=IVD PE=1 SV=1	sp|P26440|IVD_HUMAN	46,652.00	100.00%	1	1	4	0.00306%	11.00%
Pap test	Junction plakoglobin GN=JUP PE=1 SV=3	PLAK_HUMAN	81,745.90	100.00%	14	15	25	0.03900%	28.20%
Tumor tissue	Junction plakoglobin GN=JUP PE=1 SV=3	PLAK_HUMAN	81,745.90	100.00%	13	15	29	0.02220%	23.80%
Swab	Junctional adhesion molecule A GN=F11R PE=1 SV=1	sp|Q9Y624|JAM1_HUMAN	32,583.10	98.60%	1	1	1	0.00071%	4.01%
Tumor tissue	Junctional adhesion molecule A GN=F11R PE=1 SV=1	sp|Q9Y624|JAM1_HUMAN	32,583.10	100.00%	2	2	2	0.00153%	7.36%
Tumor tissue	Junctional protein associated with coronary artery disease GN=KIAA1462 PE=1 SV=3	JCAD_HUMAN	148,350.50	100.00%	2	2	2	0.00153%	2.21%
Pap test	Kallikrein-10 GN=KLK10 PE=1 SV=3	KLK10_HUMAN	30,169.70	100.00%	2	2	2	0.00312%	9.42%
Pap test	Kallikrein-13 GN=KLK13 PE=2 SV=1	sp|Q9UKR3|KLK13_HUMAN	30,569.50	100.00%	1	1	3	0.00468%	13.40%
Pap test	Kallistatin GN=SERPINA4 PE=1 SV=3	KAIN_HUMAN	48,544.00	100.00%	9	9	19	0.02960%	24.10%
Swab	Kallistatin GN=SERPINA4 PE=1 SV=3	KAIN_HUMAN	48,544.00	100.00%	8	8	14	0.00992%	22.70%
Tumor tissue	Kallistatin GN=SERPINA4 PE=1 SV=3	KAIN_HUMAN	48,544.00	100.00%	8	9	10	0.00766%	26.90%
Tumor tissue	KDEL motif-containing protein 2 GN=KDELC2 PE=1 SV=2	sp|Q7Z4H8|KDEL2_HUMAN	58,574.10	100.00%	5	6	7	0.00536%	14.00%
Tumor tissue	Kelch-like protein 14 GN=KLHL14 PE=1 SV=2	sp|Q9P2G3|KLH14_HUMAN	70,714.30	100.00%	1	1	2	0.00153%	6.53%
Pap test	Keratin, type I cytoskeletal 10 GN=KRT10 PE=1 SV=6	K1C10_HUMAN	59,512.40	100.00%	16	28	113	0.17600%	39.00%
Swab	Keratin, type I cytoskeletal 10 GN=KRT10 PE=1 SV=6	K1C10_HUMAN	58,828.80	100.00%	14	20	56	0.03970%	32.90%
Tumor tissue	Keratin, type I cytoskeletal 10 GN=KRT10 PE=1 SV=6	K1C10_HUMAN	59,512.40	100.00%	3	3	6	0.00459%	10.40%

Biological sample	Protein name	Protein accession numbers	Protein molecular weight (Da)	Protein identification probability	Exclusive unique peptide count	Exclusive unique spectrum count	Total spectrum count	Percentage of total spectra	Percentage sequence coverage
Pap test	Keratin, type I cytoskeletal 13 GN=KRT13 PE=1 SV=4	sp|P13646|K1C13_HUMAN	49,589.30	100.00%	9	19	292	0.45600%	44.50%
Swab	Keratin, type I cytoskeletal 13 GN=KRT13 PE=1 SV=4	sp|P13646|K1C13_HUMAN	49,589.30	100.00%	3	4	43	0.03050%	26.40%
Pap test	Keratin, type I cytoskeletal 14 GN=KRT14 PE=1 SV=4	K1C14_HUMAN	51,562.50	100.00%	4	5	75	0.11700%	29.20%
Swab	Keratin, type I cytoskeletal 14 GN=KRT14 PE=1 SV=4	K1C14_HUMAN	51,562.50	100.00%	4	5	22	0.01560%	30.30%
Tumor tissue	Keratin, type I cytoskeletal 14 GN=KRT14 PE=1 SV=4	K1C14_HUMAN	51,562.50	100.00%	8	9	32	0.02450%	35.80%
Pap test	Keratin, type I cytoskeletal 15 GN=KRT15 PE=1 SV=3	sp|P19012|K1C15_HUMAN	49,212.90	100.00%	5	6	103	0.16100%	31.80%
Swab	Keratin, type I cytoskeletal 15 GN=KRT15 PE=1 SV=3	sp|P19012|K1C15_HUMAN	49,212.90	100.00%	2	2	17	0.01200%	18.00%
Pap test	Keratin, type I cytoskeletal 16 GN=KRT16 PE=1 SV=4	K1C16_HUMAN	51,269.00	100.00%	4	5	72	0.11200%	34.20%
Swab	Keratin, type I cytoskeletal 16 GN=KRT16 PE=1 SV=4	K1C16_HUMAN	51,269.00	100.00%	4	5	22	0.01560%	29.40%
Pap test	Keratin, type I cytoskeletal 17 GN=KRT17 PE=1 SV=2	K1C17_HUMAN	48,106.70	100.00%	3	3	38	0.05930%	19.70%
Tumor tissue	Keratin, type I cytoskeletal 17 GN=KRT17 PE=1 SV=2	K1C17_HUMAN	48,106.70	100.00%	3	3	18	0.01380%	19.90%
Tumor tissue	Keratin, type I cytoskeletal 18 GN=KRT18 PE=1 SV=2	K1C18_HUMAN	48,059.00	100.00%	2	2	58	0.04440%	44.00%
Pap test	Keratin, type I cytoskeletal 19 GN=KRT19 PE=1 SV=4	K1C19_HUMAN	44,107.10	100.00%	15	21	103	0.16100%	63.20%
Swab	Keratin, type I cytoskeletal 19 GN=KRT19 PE=1 SV=4	K1C19_HUMAN	44,107.10	100.00%	2	2	11	0.00780%	17.00%
Tumor tissue	Keratin, type I cytoskeletal 19 GN=KRT19 PE=1 SV=4	K1C19_HUMAN	44,107.10	100.00%	20	35	81	0.06200%	69.50%
Pap test	Keratin, type I cytoskeletal 9 GN=KRT9 PE=1 SV=3	K1C9_HUMAN	62,065.90	100.00%	22	35	76	0.11900%	40.40%
Swab	Keratin, type I cytoskeletal 9 GN=KRT9 PE=1 SV=3	K1C9_HUMAN	62,065.90	100.00%	21	29	60	0.04250%	48.00%
Tumor tissue	Keratin, type I cytoskeletal 9 GN=KRT9 PE=1 SV=3	K1C9_HUMAN	62,065.90	100.00%	11	12	14	0.01070%	36.80%

Biological sample	Protein name	Protein accession numbers	Protein molecular weight (Da)	Protein identification probability	Exclusive unique peptide count	Exclusive unique spectrum count	Total spectrum count	Percentage of total spectra	Percentage sequence coverage
Tumor tissue	Keratin, type II cytoskeletal 1 GN=KRT1 PE=1 SV=6	K2C1_HUMAN	66,040.30	100.00%	1	1	26	0.01990%	25.30%
Pap test	Keratin, type II cytoskeletal 4 GN=KRT4 PE=1 SV=4	K2C4_HUMAN	57,286.10	100.00%	26	45	248	0.38700%	64.80%
Swab	Keratin, type II cytoskeletal 4 GN=KRT4 PE=1 SV=4	K2C4_HUMAN	57,286.10	100.00%	8	8	16	0.01130%	21.90%
Pap test	Keratin, type II cytoskeletal 5 GN=KRT5 PE=1 SV=3	K2C5_HUMAN	62,379.60	100.00%	17	25	161	0.25100%	43.60%
Swab	Keratin, type II cytoskeletal 5 GN=KRT5 PE=1 SV=3	K2C5_HUMAN	62,379.60	100.00%	7	10	19	0.01350%	20.00%
Pap test	Keratin, type II cytoskeletal 6A GN=KRT6A PE=1 SV=3	K2C6A_HUMAN	60,046.40	100.00%	2	3	164	0.25600%	50.50%
Swab	Keratin, type II cytoskeletal 6A GN=KRT6A PE=1 SV=3	K2C6A_HUMAN	60,046.40	100.00%	1	2	21	0.01490%	18.80%
Tumor tissue	Keratin, type II cytoskeletal 6A GN=KRT6A PE=1 SV=3	K2C6A_HUMAN	60,046.40	100.00%	1	1	17	0.01300%	22.00%
Pap test	Keratin, type II cytoskeletal 6B GN=KRT6B PE=1 SV=5	K2C6B_HUMAN	60,068.60	100.00%	1	1	141	0.22000%	47.20%
Pap test	Keratin, type II cytoskeletal 6C GN=KRT6C PE=1 SV=3	K2C6C_HUMAN	60,026.70	100.00%	1	1	136	0.21200%	47.50%
Pap test	Keratin, type II cytoskeletal 7 GN=KRT7 PE=1 SV=5	K2C7_HUMAN	51,387.30	100.00%	11	14	36	0.05620%	38.00%
Swab	Keratin, type II cytoskeletal 7 GN=KRT7 PE=1 SV=5	K2C7_HUMAN	51,387.30	100.00%	2	2	2	0.00142%	5.33%
Tumor tissue	Keratin, type II cytoskeletal 7 GN=KRT7 PE=1 SV=5	K2C7_HUMAN	51,387.30	100.00%	18	26	44	0.03370%	43.30%
Pap test	Keratin, type II cytoskeletal 78 GN=KRT78 PE=2 SV=2	sp|Q8N1N4|K2C78_HUMAN	56,866.40	100.00%	12	17	35	0.05460%	26.30%
Pap test	Keratinocyte proline-rich protein GN=KPRP PE=1 SV=1	KPRP_HUMAN	64,132.60	100.00%	2	2	2	0.00312%	3.63%
Pap test	Ketimine reductase mu-crystallin GN=CRYM PE=1 SV=1	CRYM_HUMAN	33,775.10	99.20%	1	1	1	0.00156%	3.18%
Swab	Ketimine reductase mu-crystallin GN=CRYM PE=1 SV=1	CRYM_HUMAN	33,775.10	100.00%	3	3	4	0.00283%	12.40%
Tumor tissue	Ketosamine-3-kinase GN=FN3KRP PE=1 SV=2	KT3K_HUMAN	34,412.70	100.00%	1	1	2	0.00153%	8.41%

Biological sample	Protein name	Protein accession numbers	Protein molecular weight (Da)	Protein identification probability	Exclusive unique peptide count	Exclusive unique spectrum count	Total spectrum count	Percentage of total spectra	Percentage sequence coverage
Tumor tissue	KH domain-containing, RNA-binding, signal transduction-associated protein 1 GN=KHDRBS1 PE=1 SV=1	sp|Q07666|KHDR1_HUMAN	48,228.20	100.00%	1	1	10	0.00766%	19.60%
Tumor tissue	KIF1-binding protein GN=KIF1BP PE=1 SV=1	A0A1B0GUA3_HUMAN	74,785.50	99.90%	1	1	2	0.00153%	5.26%
Pap test	Kinectin GN=KTN1 PE=1 SV=1	sp|Q86UP2|KTN1_HUMAN	156,276.30	100.00%	1	1	1	0.00156%	0.88%
Tumor tissue	Kinectin GN=KTN1 PE=1 SV=1	sp|Q86UP2|KTN1_HUMAN	156,276.30	100.00%	22	31	36	0.02760%	26.50%
Pap test	Kinesin-1 heavy chain GN=KIF5B PE=1 SV=1	KINH_HUMAN	109,686.30	100.00%	2	2	2	0.00312%	3.22%
Swab	Kinesin-1 heavy chain GN=KIF5B PE=1 SV=1	KINH_HUMAN	109,686.30	100.00%	1	2	2	0.00142%	1.77%
Tumor tissue	Kinesin-1 heavy chain GN=KIF5B PE=1 SV=1	KINH_HUMAN	109,686.30	100.00%	20	23	32	0.02450%	32.70%
Tumor tissue	Kinesin-like protein KIF13B GN=KIF13B PE=1 SV=2	KI13B_HUMAN	202,789.20	100.00%	3	3	3	0.00230%	2.57%
Tumor tissue	Kininogen-1 GN=KNG1 PE=1 SV=2	sp|P01042|KNG1_HUMAN	71,957.60	100.00%	1	1	6	0.00459%	8.54%
Pap test	Kunitz-type protease inhibitor 1 (Fragment) GN=SPINT1 PE=1 SV=1	sp|O43278|SPIT1_HUMAN	52,373.00	100.00%	3	3	5	0.00780%	8.26%
Swab	Kunitz-type protease inhibitor 1 (Fragment) GN=SPINT1 PE=1 SV=1	sp|O43278|SPIT1_HUMAN	52,373.00	100.00%	2	2	2	0.00142%	5.30%
Pap test	Kynureninase GN=KYNU PE=1 SV=1	sp|Q16719|KYNU_HUMAN	52,353.30	100.00%	2	2	2	0.00312%	5.38%
Tumor tissue	Kynureninase GN=KYNU PE=1 SV=1	KYNU_HUMAN	52,353.30	100.00%	2	2	2	0.00153%	3.44%
Tumor tissue	Lactadherin GN=MFGE8 PE=1 SV=1	sp|Q08431|MFGM_HUMAN	37,504.90	100.00%	5	5	5	0.00383%	18.70%
Pap test	Lactotransferrin GN=LTF PE=1 SV=6	sp|P02788|TRFL_HUMAN	78,182.30	100.00%	48	94	449	0.70100%	75.50%
Swab	Lactotransferrin GN=LTF PE=1 SV=6	sp|P02788|TRFL_HUMAN	78,182.30	100.00%	47	79	297	0.21000%	71.50%
Tumor tissue	Lactotransferrin GN=LTF PE=1 SV=6	sp|P02788|TRFL_HUMAN	78,182.30	100.00%	7	7	7	0.00536%	12.40%
Pap test	Lactoylglutathione lyase GN=GLO1 PE=1 SV=4	sp|Q04760|LGUL_HUMAN	20,778.80	100.00%	3	3	3	0.00468%	19.00%
Swab	Lactoylglutathione lyase GN=GLO1 PE=1 SV=4	sp|Q04760|LGUL_HUMAN	20,778.80	100.00%	2	2	2	0.00142%	9.78%

Biological sample	Protein name	Protein accession numbers	Protein molecular weight (Da)	Protein identification probability	Exclusive unique peptide count	Exclusive unique spectrum count	Total spectrum count	Percentage of total spectra	Percentage sequence coverage
Tumor tissue	Lactoylglutathione lyase GN=GLO1 PE=1 SV=4	sp|Q04760|LGUL_HUMAN	20,778.80	100.00%	1	1	2	0.00153%	5.43%
Swab	Ladinin-1 GN=LAD1 PE=1 SV=1	LAD1_HUMAN	58,678.60	100.00%	2	2	2	0.00142%	9.98%
Tumor tissue	Lamina-associated polypeptide 2, isoform alpha GN=TMPO PE=1 SV=2	LAP2A_HUMAN	75,493.80	100.00%	3	4	14	0.01070%	19.70%
Tumor tissue	Lamina-associated polypeptide 2, isoforms beta/gamma GN=TMPO PE=1 SV=2	sp|P42167|LAP2B_HUMAN	50,671.40	100.00%	1	2	16	0.01220%	32.20%
Pap test	Lamin-B1 GN=LMNB1 PE=1 SV=2	LMNB1_HUMAN	66,409.60	100.00%	5	5	5	0.00780%	7.34%
Swab	Lamin-B1 GN=LMNB1 PE=1 SV=2	LMNB1_HUMAN	66,409.60	100.00%	4	4	4	0.00283%	10.90%
Tumor tissue	Lamin-B1 GN=LMNB1 PE=1 SV=2	LMNB1_HUMAN	66,409.60	100.00%	24	35	45	0.03440%	40.10%
Pap test	Lamin-B2 GN=LMNB2 PE=1 SV=4	LMNB2_HUMAN	69,949.40	100.00%	3	3	3	0.00468%	5.65%
Tumor tissue	Lamin-B2 GN=LMNB2 PE=1 SV=4	LMNB2_HUMAN	69,949.40	100.00%	20	26	36	0.02760%	38.90%
Tumor tissue	Laminin subunit alpha-4 GN=LAMA4 PE=1 SV=1	sp|Q16363|LAMA4_HUMAN	201,822.60	100.00%	21	27	36	0.02760%	14.60%
Tumor tissue	Laminin subunit alpha-5 GN=LAMA5 PE=1 SV=8	sp|O15230|LAMA5_HUMAN	399,725.10	100.00%	18	19	21	0.01610%	7.12%
Tumor tissue	Laminin subunit beta-2 GN=LAMB2 PE=1 SV=2	LAMB2_HUMAN	195,975.90	100.00%	17	19	21	0.01610%	13.80%
Tumor tissue	Laminin subunit gamma-1 GN=LAMC1 PE=1 SV=3	LAMC1_HUMAN	177,601.30	100.00%	15	21	24	0.01840%	10.80%
Tumor tissue	L-aminoadipate-semialdehyde dehydrogenase-phosphopantetheinyl transferase GN=AASDHPPT PE=1 SV=2	ADPPT_HUMAN	35,777.10	100.00%	2	2	2	0.00153%	10.40%
Tumor tissue	LanC-like protein 2 GN=LANCL2 PE=1 SV=1	LANC2_HUMAN	50,855.60	100.00%	2	2	3	0.00230%	6.89%
Tumor tissue	Lanosterol 14-alpha demethylase GN=CYP51A1 PE=1 SV=1	sp|Q16850|CP51A_HUMAN	57,281.00	100.00%	2	2	2	0.00153%	4.72%
Tumor tissue	La-related protein 4B GN=LARP4B PE=1 SV=3	LAR4B_HUMAN	80,553.10	100.00%	3	3	3	0.00230%	5.83%

Biological sample	Protein name	Protein accession numbers	Protein molecular weight (Da)	Protein identification probability	Exclusive unique peptide count	Exclusive unique spectrum count	Total spectrum count	Percentage of total spectra	Percentage sequence coverage
Tumor tissue	Large neutral amino acids transporter small subunit 1 GN=SLC7A5 PE=1 SV=2	LAT1_HUMAN	55,012.10	100.00%	2	2	2	0.00153%	6.31%
Tumor tissue	Lariat debranching enzyme GN=DBR1 PE=1 SV=2	sp|Q9UK59|DBR1_HUMAN	61,556.10	100.00%	4	4	4	0.00306%	9.93%
Tumor tissue	Latent-transforming growth factor beta-binding protein 2 GN=LTBP2 PE=1 SV=1	LTBP2_HUMAN	190,278.50	100.00%	4	6	6	0.00459%	4.28%
Tumor tissue	Latexin GN=LXN PE=1 SV=2	LXN_HUMAN	25,749.80	100.00%	2	3	3	0.00230%	12.60%
Tumor tissue	LDLR chaperone MESD GN=MESDC2 PE=1 SV=2	MESD_HUMAN	26,077.90	100.00%	5	5	5	0.00383%	26.50%
Tumor tissue	LEM domain-containing protein 2 GN=LEMD2 PE=1 SV=1	sp|Q8NC56|LEMD2_HUMAN	56,976.70	100.00%	5	5	8	0.00612%	20.70%
Tumor tissue	Lethal(2) giant larvae protein homolog 2 GN=LLGL2 PE=1 SV=1	sp|Q6P1M3|L2GL2_HUMAN	113,377.40	100.00%	2	3	3	0.00230%	3.24%
Tumor tissue	LETM1 and EF-hand domain-containing protein 1, mitochondrial GN=LETM1 PE=1 SV=1	sp|O95202|LETM1_HUMAN	83,355.90	100.00%	7	8	8	0.00612%	10.70%
Tumor tissue	Leucine zipper protein 1 GN=LUZP1 PE=1 SV=2	sp|Q86V48|LUZP1_HUMAN	120,277.40	100.00%	2	2	2	0.00153%	2.51%
Pap test	Leucine-rich alpha-2-glycoprotein GN=LRG1 PE=1 SV=2	A2GL_HUMAN	38,179.70	100.00%	6	11	25	0.03900%	26.20%
Swab	Leucine-rich alpha-2-glycoprotein GN=LRG1 PE=1 SV=2	A2GL_HUMAN	38,179.70	100.00%	8	13	51	0.03610%	29.40%
Tumor tissue	Leucine-rich alpha-2-glycoprotein GN=LRG1 PE=1 SV=2	A2GL_HUMAN	38,179.70	100.00%	3	4	5	0.00383%	10.70%
Tumor tissue	Leucine-rich PPR motif-containing protein, mitochondrial GN=LRPPRC PE=1 SV=3	LPPRC_HUMAN	157,912.20	100.00%	20	25	43	0.03290%	25.30%
Tumor tissue	Leucine-rich repeat-containing protein 40 GN=LRRC40 PE=1 SV=1	LRC40_HUMAN	68,254.30	100.00%	3	3	3	0.00230%	11.30%
Tumor tissue	Leucine-rich repeat-containing protein 47 GN=LRRC47 PE=1 SV=1	LRC47_HUMAN	63,474.80	100.00%	9	12	13	0.00995%	20.10%

Biological sample	Protein name	Protein accession numbers	Protein molecular weight (Da)	Protein identification probability	Exclusive unique peptide count	Exclusive unique spectrum count	Total spectrum count	Percentage of total spectra	Percentage sequence coverage
Tumor tissue	Leucine-rich repeat-containing protein 59 GN=LRRC59 PE=1 SV=1	LRC59_HUMAN	34,931.40	100.00%	7	11	23	0.01760%	28.00%
Pap test	Leukocyte elastase inhibitor GN=SERPINB1 PE=1 SV=1	sp|P30740|ILEU_HUMAN	42,743.80	100.00%	17	38	157	0.24500%	53.80%
Swab	Leukocyte elastase inhibitor GN=SERPINB1 PE=1 SV=1	sp|P30740|ILEU_HUMAN	42,743.80	100.00%	18	32	95	0.06730%	44.90%
Tumor tissue	Leukocyte elastase inhibitor GN=SERPINB1 PE=1 SV=1	sp|P30740|ILEU_HUMAN	42,743.80	100.00%	15	23	43	0.03290%	45.40%
Pap test	Leukocyte immunoglobulin-like receptor subfamily A member 3 GN=LILRA3 PE=1 SV=1	sp|Q8N6C8|LIRA3_HUMAN	52,934.40	99.90%	1	1	2	0.00312%	5.11%
Tumor tissue	Leukocyte-associated immunoglobulin-like receptor 1 GN=LAIR1 PE=1 SV=2	D3YTC8_HUMAN	29,689.90	100.00%	2	2	2	0.00153%	15.60%
Tumor tissue	LIM and cysteine-rich domains protein 1 GN=LMCD1 PE=1 SV=1	sp|Q9NZU5|LMCD1_HUMAN	40,833.10	100.00%	3	3	15	0.01150%	26.00%
Pap test	LIM and SH3 domain protein 1 GN=LASP1 PE=1 SV=2	sp|Q14847|LASP1_HUMAN	29,716.80	100.00%	2	3	5	0.00780%	10.70%
Swab	LIM and SH3 domain protein 1 GN=LASP1 PE=1 SV=2	sp|Q14847|LASP1_HUMAN	29,716.80	100.00%	2	2	3	0.00213%	14.20%
Tumor tissue	LIM and SH3 domain protein 1 GN=LASP1 PE=1 SV=2	sp|Q14847|LASP1_HUMAN	29,716.80	100.00%	7	8	21	0.01610%	46.40%
Tumor tissue	LIM domain only protein 7 GN=LMO7 PE=1 SV=2	sp|Q8WWI1|LMO7_HUMAN	190,681.90	100.00%	1	1	5	0.00383%	3.92%
Tumor tissue	Lipoamide acyltransferase component of branched-chain alpha-keto acid dehydrogenase complex, mitochondrial GN=DBT PE=1 SV=3	ODB2_HUMAN	53,488.50	100.00%	2	3	3	0.00230%	6.02%
Tumor tissue	Lipoma-preferred partner GN=LPP PE=1 SV=1	LPP_HUMAN	65,746.20	100.00%	10	19	38	0.02910%	27.90%
Pap test	Lipopolysaccharide-binding protein GN=LBP PE=1 SV=3	LBP_HUMAN	53,386.00	100.00%	3	3	4	0.00624%	8.32%
Swab	Lipopolysaccharide-binding protein GN=LBP PE=1 SV=3	LBP_HUMAN	53,386.00	100.00%	2	2	3	0.00213%	4.57%

Biological sample	Protein name	Protein accession numbers	Protein molecular weight (Da)	Protein identification probability	Exclusive unique peptide count	Exclusive unique spectrum count	Total spectrum count	Percentage of total spectra	Percentage sequence coverage
Tumor tissue	Lipopolysaccharide-binding protein GN=LBP PE=1 SV=3	LBP_HUMAN	53,386.00	100.00%	5	6	7	0.00536%	13.10%
Tumor tissue	LisH domain-containing protein ARMC9 GN=ARMC9 PE=1 SV=1	sp|Q7Z3E5|ARMC9_HUMAN	91,821.90	100.00%	2	2	2	0.00153%	3.18%
Pap test	L-lactate dehydrogenase B chain GN=LDHB PE=1 SV=2	LDHB_HUMAN	36,638.60	100.00%	5	5	9	0.01400%	21.00%
Swab	L-lactate dehydrogenase B chain GN=LDHB PE=1 SV=2	LDHB_HUMAN	36,638.60	100.00%	6	7	14	0.00992%	25.40%
Tumor tissue	L-lactate dehydrogenase B chain GN=LDHB PE=1 SV=2	LDHB_HUMAN	36,638.60	100.00%	10	17	37	0.02830%	30.20%
Tumor tissue	Long-chain fatty acid transport protein 1 GN=SLC27A1 PE=2 SV=1	sp|Q6PCB7|S27A1_HUMAN	71,109.60	100.00%	2	2	4	0.00306%	9.29%
Pap test	Long-chain-fatty-acid--CoA ligase 1 GN=ACSL1 PE=1 SV=1	sp|P33121|ACSL1_HUMAN	74,283.70	98.90%	1	1	1	0.00156%	1.36%
Swab	Long-chain-fatty-acid--CoA ligase 1 GN=ACSL1 PE=1 SV=1	sp|P33121|ACSL1_HUMAN	74,283.70	99.90%	1	1	1	0.00071%	1.81%
Tumor tissue	Long-chain-fatty-acid--CoA ligase 1 GN=ACSL1 PE=1 SV=1	sp|P33121|ACSL1_HUMAN	74,283.70	100.00%	9	10	11	0.00842%	17.60%
Tumor tissue	Long-chain-fatty-acid--CoA ligase 3 GN=ACSL3 PE=1 SV=3	ACSL3_HUMAN	80,423.10	100.00%	6	7	9	0.00689%	16.40%
Pap test	Low affinity immunoglobulin gamma Fc region receptor III-B GN=FCGR3B PE=1 SV=2	FCG3B_HUMAN	26,216.00	99.90%	1	1	4	0.00624%	11.60%
Tumor tissue	Low molecular weight phosphotyrosine protein phosphatase GN=ACP1 PE=1 SV=3	sp|P24666|PPAC_HUMAN	18,042.70	100.00%	3	4	6	0.00459%	31.00%
Pap test	Lumican GN=LUM PE=1 SV=2	LUM_HUMAN	38,431.50	100.00%	4	4	4	0.00624%	11.20%
Swab	Lumican GN=LUM PE=1 SV=2	LUM_HUMAN	38,431.50	100.00%	6	6	7	0.00496%	21.60%
Tumor tissue	Lumican GN=LUM PE=1 SV=2	LUM_HUMAN	38,431.50	100.00%	10	17	60	0.04590%	33.10%
Tumor tissue	Lupus La protein GN=SSB PE=1 SV=2	LA_HUMAN	46,839.00	100.00%	8	11	31	0.02370%	29.40%
Pap test	L-xylulose reductase GN=DCXR PE=1 SV=2	DCXR_HUMAN	23,802.80	100.00%	1	2	2	0.00312%	4.92%
Swab	L-xylulose reductase GN=DCXR PE=1 SV=2	DCXR_HUMAN	23,802.80	100.00%	3	5	7	0.00496%	28.70%

Biological sample	Protein name	Protein accession numbers	Protein molecular weight (Da)	Protein identification probability	Exclusive unique peptide count	Exclusive unique spectrum count	Total spectrum count	Percentage of total spectra	Percentage sequence coverage
Tumor tissue	L-xylulose reductase GN=DCXR PE=1 SV=2	DCXR_HUMAN	25,912.20	100.00%	2	3	5	0.00383%	18.90%
Pap test	Ly6/PLAUR domain-containing protein 3 GN=LYPD3 PE=1 SV=2	LYPD3_HUMAN	35,969.70	100.00%	3	3	5	0.00780%	11.00%
Swab	Ly6/PLAUR domain-containing protein 3 GN=LYPD3 PE=1 SV=2	LYPD3_HUMAN	35,969.70	100.00%	3	3	5	0.00354%	11.80%
Pap test	Lymphocyte antigen 6D GN=LY6D PE=1 SV=1	LY6D_HUMAN	13,286.30	100.00%	2	2	2	0.00312%	14.80%
Tumor tissue	Lymphocyte cytosolic protein 2 GN=LCP2 PE=1 SV=1	LCP2_HUMAN	60,145.70	100.00%	2	2	2	0.00153%	4.32%
Pap test	Lymphocyte-specific protein 1 GN=LSP1 PE=1 SV=1	sp|P33241|LSP1_HUMAN	37,191.10	100.00%	3	3	4	0.00624%	20.40%
Swab	Lymphocyte-specific protein 1 GN=LSP1 PE=1 SV=1	sp|P33241|LSP1_HUMAN	37,191.10	100.00%	3	4	6	0.00425%	22.70%
Tumor tissue	Lymphocyte-specific protein 1 GN=LSP1 PE=1 SV=1	sp|P33241|LSP1_HUMAN	37,191.10	100.00%	7	11	19	0.01450%	37.80%
Tumor tissue	Lys-63-specific deubiquitinase BRCC36 GN=BRCC3 PE=1 SV=1	A0A0D9SF50_HUMAN	36,137.00	100.00%	3	4	4	0.00306%	17.70%
Tumor tissue	Lys-63-specific deubiquitinase BRCC36 (Fragment) GN=BRCC3 PE=1 SV=1	A0A0A0MS96_HUMAN	10,363.70	100.00%	2	2	2	0.00153%	21.10%
Tumor tissue	Lysophosphatidylcholine acyltransferase 2 GN=LPCAT2 PE=1 SV=1	sp|Q7L5N7|PCAT2_HUMAN	60,208.70	99.80%	1	1	2	0.00153%	3.12%
Tumor tissue	Lysophospholipid acyltransferase 7 GN=MBOAT7 PE=1 SV=2	sp|Q96N66|MBOA7_HUMAN	52,767.70	100.00%	3	3	3	0.00230%	8.47%
Tumor tissue	Lysophospholipid acyltransferase LPCAT4 GN=LPCAT4 PE=1 SV=1	LPCT4_HUMAN	57,220.00	100.00%	2	2	2	0.00153%	5.15%
Swab	Lysosomal alpha-glucosidase GN=GAA PE=1 SV=4	LYAG_HUMAN	105,324.40	100.00%	3	4	7	0.00496%	4.83%
Tumor tissue	Lysosomal alpha-glucosidase GN=GAA PE=1 SV=4	LYAG_HUMAN	105,324.40	100.00%	4	5	6	0.00459%	6.20%
Pap test	Lysosomal alpha-mannosidase GN=MAN2B1 PE=1 SV=3	sp|O00754|MA2B1_HUMAN	113,744.90	100.00%	3	3	3	0.00468%	4.95%
Swab	Lysosomal alpha-mannosidase GN=MAN2B1 PE=1 SV=3	sp|O00754|MA2B1_HUMAN	113,744.90	99.90%	1	1	1	0.00071%	1.19%

Biological sample	Protein name	Protein accession numbers	Protein molecular weight (Da)	Protein identification probability	Exclusive unique peptide count	Exclusive unique spectrum count	Total spectrum count	Percentage of total spectra	Percentage sequence coverage
Tumor tissue	Lysosomal alpha-mannosidase GN=MAN2B1 PE=1 SV=3	sp|O00754|MA2B1_HUMAN	113,744.90	100.00%	5	5	5	0.00383%	6.33%
Pap test	Lysosomal Pro-X carboxypeptidase GN=PRCP PE=1 SV=1	sp|P42785|PCP_HUMAN	55,801.10	100.00%	1	1	3	0.00468%	6.05%
Swab	Lysosomal Pro-X carboxypeptidase GN=PRCP PE=1 SV=1	sp|P42785|PCP_HUMAN	55,801.10	100.00%	1	1	1	0.00071%	2.42%
Tumor tissue	Lysosomal Pro-X carboxypeptidase GN=PRCP PE=1 SV=1	sp|P42785|PCP_HUMAN	55,801.10	100.00%	2	4	6	0.00459%	6.25%
Pap test	Lysosome membrane protein 2 GN=SCARB2 PE=1 SV=2	sp|Q14108|SCRB2_HUMAN	54,291.70	100.00%	3	3	3	0.00468%	10.90%
Tumor tissue	Lysosome membrane protein 2 GN=SCARB2 PE=1 SV=2	sp|Q14108|SCRB2_HUMAN	54,291.70	100.00%	5	6	7	0.00536%	10.00%
Pap test	Lysosome-associated membrane glycoprotein 1 GN=LAMP1 PE=1 SV=3	sp|P11279|LAMP1_HUMAN	44,883.10	100.00%	2	2	4	0.00624%	4.80%
Tumor tissue	Lysosome-associated membrane glycoprotein 1 GN=LAMP1 PE=1 SV=3	sp|P11279|LAMP1_HUMAN	44,883.10	100.00%	5	7	15	0.01150%	11.80%
Pap test	Lysosome-associated membrane glycoprotein 2 GN=LAMP2 PE=1 SV=2	sp|P13473|LAMP2_HUMAN	44,960.90	100.00%	1	2	3	0.00468%	2.93%
Swab	Lysosome-associated membrane glycoprotein 2 GN=LAMP2 PE=1 SV=2	sp|P13473|LAMP2_HUMAN	44,960.90	100.00%	2	2	4	0.00283%	12.70%
Tumor tissue	Lysosome-associated membrane glycoprotein 2 GN=LAMP2 PE=1 SV=2	sp|P13473|LAMP2_HUMAN	44,960.90	100.00%	2	3	3	0.00230%	4.88%
Pap test	Lysozyme C GN=LYZ PE=1 SV=1	LYSC_HUMAN	16,536.90	100.00%	2	4	29	0.04530%	45.90%
Swab	Lysozyme C GN=LYZ PE=1 SV=1	LYSC_HUMAN	16,536.90	100.00%	1	3	21	0.01490%	32.40%
Tumor tissue	m7GpppX diphosphatase GN=DCPS PE=1 SV=2	DCPS_HUMAN	38,609.80	100.00%	3	4	4	0.00306%	12.80%
Tumor tissue	Macrophage mannose receptor 1 GN=MRC1 PE=1 SV=1	sp|P22897|MRC1_HUMAN	166,014.70	100.00%	4	4	4	0.00306%	3.30%
Tumor tissue	Macrophage scavenger receptor types I and II GN=MSR1 PE=1 SV=1	B4DDJ5_HUMAN	51,726.30	100.00%	2	2	2	0.00153%	4.69%

Biological sample	Protein name	Protein accession numbers	Protein molecular weight (Da)	Protein identification probability	Exclusive unique peptide count	Exclusive unique spectrum count	Total spectrum count	Percentage of total spectra	Percentage sequence coverage
Pap test	Macrophage-capping protein GN=CAPG PE=1 SV=2	sp|P40121|CAPG_HUMAN	38,498.90	100.00%	4	7	23	0.03590%	21.00%
Swab	Macrophage-capping protein GN=CAPG PE=1 SV=2	sp|P40121|CAPG_HUMAN	38,498.90	100.00%	7	12	35	0.02480%	32.20%
Tumor tissue	Macrophage-capping protein GN=CAPG PE=1 SV=2	sp|P40121|CAPG_HUMAN	38,498.90	100.00%	7	11	40	0.03060%	32.20%
Tumor tissue	Maestro heat-like repeat-containing protein family member 1 GN=MROH1 PE=1 SV=1	sp|Q8NDA8|MROH1_HUMAN	180,284.50	100.00%	3	3	8	0.00612%	7.41%
Tumor tissue	Magnesium transporter protein 1 GN=MAGT1 PE=1 SV=1	A0A087WU53_HUMAN	41,532.40	100.00%	5	6	6	0.00459%	13.90%
Tumor tissue	MAGUK p55 subfamily member 5 GN=MPP5 PE=1 SV=3	sp|Q8N3R9|MPP5_HUMAN	77,295.40	100.00%	2	2	2	0.00153%	5.93%
Tumor tissue	MAGUK p55 subfamily member 7 GN=MPP7 PE=1 SV=1	sp|Q5T2T1|MPP7_HUMAN	65,526.00	100.00%	6	6	6	0.00459%	14.80%
Pap test	Major vault protein GN=MVP PE=1 SV=4	MVP_HUMAN	99,326.00	100.00%	10	12	15	0.02340%	16.10%
Swab	Major vault protein GN=MVP PE=1 SV=4	MVP_HUMAN	99,326.00	100.00%	12	14	24	0.01700%	21.30%
Tumor tissue	Major vault protein GN=MVP PE=1 SV=4	MVP_HUMAN	99,326.00	100.00%	25	42	56	0.04290%	39.10%
Pap test	Malate dehydrogenase, mitochondrial GN=MDH2 PE=1 SV=3	sp|P40926|MDHM_HUMAN	35,503.70	100.00%	14	16	25	0.03900%	53.80%
Swab	Malate dehydrogenase, mitochondrial GN=MDH2 PE=1 SV=3	sp|P40926|MDHM_HUMAN	35,503.70	100.00%	9	11	22	0.01560%	36.40%
Tumor tissue	Malate dehydrogenase, mitochondrial GN=MDH2 PE=1 SV=3	sp|P40926|MDHM_HUMAN	35,503.70	100.00%	9	19	64	0.04900%	33.70%
Tumor tissue	Malectin GN=MLEC PE=1 SV=1	MLEC_HUMAN	32,234.10	100.00%	4	4	4	0.00306%	18.50%
Tumor tissue	Mannose-1-phosphate guanyltransferase alpha GN=GMPPA PE=1 SV=1	sp|Q96IJ6|GMPPA_HUMAN	46,291.80	100.00%	7	8	9	0.00689%	24.30%

Biological sample	Protein name	Protein accession numbers	Protein molecular weight (Da)	Protein identification probability	Exclusive unique peptide count	Exclusive unique spectrum count	Total spectrum count	Percentage of total spectra	Percentage sequence coverage
Swab	Mannose-6-phosphate isomerase GN=MPI PE=1 SV=1	F5GX71_HUMAN	41,206.70	100.00%	1	1	4	0.00283%	13.10%
Tumor tissue	Mannose-6-phosphate isomerase GN=MPI PE=1 SV=1	F5GX71_HUMAN	41,206.70	99.90%	1	1	9	0.00689%	17.40%
Tumor tissue	Mannose-P-dolichol utilization defect 1 isoform 2 GN=MPDU1 PE=1 SV=1	sp|O75352|MPU1_HUMAN	11,802.90	100.00%	2	2	3	0.00230%	22.00%
Tumor tissue	Marginal zone B- and B1-cell-specific protein GN=MZB1 PE=1 SV=1	sp|Q8WU39|MZB1_HUMAN	20,694.60	100.00%	5	7	11	0.00842%	31.20%
Tumor tissue	Mast cell carboxypeptidase A GN=CPA3 PE=1 SV=2	CBPA3_HUMAN	48,671.80	100.00%	4	4	5	0.00383%	9.59%
Tumor tissue	Matrin-3 GN=MATR3 PE=1 SV=1	A8MXP9_HUMAN	99,970.90	100.00%	7	9	20	0.01530%	16.50%
Tumor tissue	Matrix metalloproteinase-14 GN=MMP14 PE=1 SV=3	MMP14_HUMAN	65,895.80	100.00%	2	2	2	0.00153%	4.12%
Pap test	Matrix metalloproteinase-9 GN=MMP9 PE=1 SV=3	MMP9_HUMAN	78,459.70	100.00%	22	32	102	0.15900%	36.60%
Swab	Matrix metalloproteinase-9 GN=MMP9 PE=1 SV=3	MMP9_HUMAN	78,459.70	100.00%	22	34	81	0.05740%	32.70%
Tumor tissue	Matrix-remodeling-associated protein 5 GN=MXRA5 PE=2 SV=3	MXRA5_HUMAN	312,150.50	100.00%	21	24	28	0.02140%	9.90%
Tumor tissue	Matrix-remodeling-associated protein 7 GN=MXRA7 PE=1 SV=1	sp|P84157|MXRA7_HUMAN	21,466.20	100.00%	5	5	6	0.00459%	28.40%
Tumor tissue	Mediator of RNA polymerase II transcription subunit 1 GN=MED1 PE=1 SV=4	sp|Q15648|MED1_HUMAN	168,481.70	100.00%	2	2	2	0.00153%	1.90%
Tumor tissue	Mediator of RNA polymerase II transcription subunit 24 GN=MED24 PE=1 SV=1	sp|O75448|MED24_HUMAN	113,016.60	100.00%	2	2	2	0.00153%	3.16%
Tumor tissue	Mediator of RNA polymerase II transcription subunit 4 GN=MED4 PE=1 SV=1	sp|Q9NPJ6|MED4_HUMAN	29,746.20	100.00%	2	2	3	0.00230%	11.10%
Swab	Medium-chain-specific acyl-CoA dehydrogenase, mitochondrial GN=ACADM PE=1 SV=1	sp|P11310|ACADM_HUMAN	50,272.20	99.70%	1	1	2	0.00142%	3.12%

Biological sample	Protein name	Protein accession numbers	Protein molecular weight (Da)	Protein identification probability	Exclusive unique peptide count	Exclusive unique spectrum count	Total spectrum count	Percentage of total spectra	Percentage sequence coverage
Tumor tissue	Medium-chain-specific acyl-CoA dehydrogenase, mitochondrial GN=ACADM PE=1 SV=1	sp|P11310|ACADM_HUMAN	42,427.80	100.00%	5	5	5	0.00383%	16.40%
Tumor tissue	Melanoma inhibitory activity protein 2 GN=MIA2 PE=1 SV=4	sp|Q96PC5|MIA2_HUMAN	159,841.60	100.00%	3	3	3	0.00230%	3.61%
Tumor tissue	Melanoma-associated antigen D2 GN=MAGED2 PE=1 SV=2	sp|Q9UNF1|MAGD2_HUMAN	64,956.00	100.00%	1	1	8	0.00612%	14.90%
Tumor tissue	Membrane primary amine oxidase GN=AOC3 PE=1 SV=3	sp|Q16853|AOC3_HUMAN	84,622.10	100.00%	4	6	9	0.00689%	7.86%
Pap test	Membrane-associated progesterone receptor component 1 GN=PGRMC1 PE=1 SV=3	sp|O00264|PGRC1_HUMAN	21,672.20	100.00%	1	1	2	0.00312%	11.80%
Tumor tissue	Membrane-associated progesterone receptor component 1 GN=PGRMC1 PE=1 SV=3	sp|O00264|PGRC1_HUMAN	21,672.20	100.00%	7	8	13	0.00995%	32.30%
Pap test	Mesencephalic astrocyte-derived neurotrophic factor GN=MANF PE=1 SV=1	MANF_HUMAN	21,144.90	99.20%	1	1	2	0.00312%	8.11%
Tumor tissue	Mesencephalic astrocyte-derived neurotrophic factor GN=MANF PE=1 SV=1	MANF_HUMAN	21,144.90	100.00%	6	8	11	0.00842%	27.60%
Swab	Mesothelin GN=MSLN PE=1 SV=2	sp|Q13421|MSLN_HUMAN	68,988.10	100.00%	1	1	5	0.00354%	7.46%
Tumor tissue	Mesothelin GN=MSLN PE=1 SV=2	sp|Q13421|MSLN_HUMAN	68,988.10	100.00%	1	1	7	0.00536%	11.00%
Pap test	Mesothelin (Fragment) GN=MSLN PE=1 SV=1	H3BUX1_HUMAN	43,829.70	99.80%	1	1	1	0.00156%	3.52%
Swab	Mesothelin (Fragment) GN=MSLN PE=1 SV=1	H3BUX1_HUMAN	43,829.70	100.00%	1	3	9	0.00638%	13.10%
Pap test	Metalloproteinase inhibitor 2 GN=TIMP2 PE=1 SV=2	TIMP2_HUMAN	24,399.90	99.20%	1	1	1	0.00156%	6.36%
Tumor tissue	Metalloproteinase inhibitor 2 GN=TIMP2 PE=1 SV=2	TIMP2_HUMAN	24,399.90	100.00%	2	2	2	0.00153%	13.20%
Tumor tissue	Metalloproteinase inhibitor 3 GN=TIMP3 PE=1 SV=2	TIMP3_HUMAN	24,145.50	100.00%	4	4	7	0.00536%	16.10%
Tumor tissue	Metastasis-associated protein MTA2 GN=MTA2 PE=1 SV=1	sp|O94776|MTA2_HUMAN	75,025.10	100.00%	6	6	8	0.00612%	12.70%

Biological sample	Protein name	Protein accession numbers	Protein molecular weight (Da)	Protein identification probability	Exclusive unique peptide count	Exclusive unique spectrum count	Total spectrum count	Percentage of total spectra	Percentage sequence coverage
Tumor tissue	Metastasis-associated protein MTA3 GN=MTA3 PE=1 SV=1	E7EV10_HUMAN	67,197.20	99.90%	1	1	2	0.00153%	3.90%
Tumor tissue	Metaxin 1, isoform CRA_b GN=MTX1 PE=1 SV=1	sp|Q13505|MTX1_HUMAN	51,464.00	100.00%	5	7	8	0.00612%	16.30%
Tumor tissue	Metaxin-2 GN=MTX2 PE=1 SV=1	sp|O75431|MTX2_HUMAN	23,643.20	100.00%	3	3	3	0.00230%	21.70%
Tumor tissue	Methionine adenosyltransferase 2 subunit beta GN=MAT2B PE=1 SV=1	sp|Q9NZL9|MAT2B_HUMAN	37,551.60	100.00%	1	1	6	0.00459%	21.90%
Tumor tissue	Methionine--tRNA ligase, cytoplasmic GN=MARS PE=1 SV=2	sp|P56192|SYMC_HUMAN	101,117.50	100.00%	9	10	17	0.01300%	19.70%
Tumor tissue	Methylated-DNA--protein-cysteine methyltransferase GN=MGMT PE=1 SV=1	MGMT_HUMAN	21,645.90	100.00%	2	3	3	0.00230%	13.00%
Tumor tissue	Methylcrotonoyl-CoA carboxylase subunit alpha, mitochondrial GN=MCCC1 PE=1 SV=3	MCCA_HUMAN	80,473.60	100.00%	1	1	5	0.00383%	11.70%
Swab	Methylmalonate-semialdehyde dehydrogenase [acylating], mitochondrial GN=ALDH6A1 PE=1 SV=2	sp|Q02252|MMSA_HUMAN	57,840.30	100.00%	1	1	1	0.00071%	2.06%
Tumor tissue	Methylmalonate-semialdehyde dehydrogenase [acylating], mitochondrial GN=ALDH6A1 PE=1 SV=2	sp|Q02252|MMSA_HUMAN	57,840.30	100.00%	10	14	16	0.01220%	23.70%
Tumor tissue	Methylmalonic aciduria type A protein, mitochondrial GN=MMAA PE=1 SV=1	MMAA_HUMAN	47,137.50	99.80%	2	2	2	0.00153%	7.18%
Tumor tissue	Methylmalonyl-CoA mutase, mitochondrial GN=MUT PE=1 SV=4	MUTA_HUMAN	83,136.80	100.00%	6	6	6	0.00459%	12.50%
Tumor tissue	Methylosome protein 50 GN=WDR77 PE=1 SV=1	sp|Q9BQA1|MEP50_HUMAN	36,723.20	100.00%	2	2	2	0.00153%	8.48%
Swab	Methylthioribulose-1-phosphate dehydratase GN=APIP PE=1 SV=1	sp|Q96GX9|MTNB_HUMAN	27,125.40	99.60%	1	1	1	0.00071%	4.55%
Tumor tissue	Methylthioribulose-1-phosphate dehydratase GN=APIP PE=1 SV=1	sp|Q96GX9|MTNB_HUMAN	27,125.40	100.00%	4	5	6	0.00459%	22.30%

Biological sample	Protein name	Protein accession numbers	Protein molecular weight (Da)	Protein identification probability	Exclusive unique peptide count	Exclusive unique spectrum count	Total spectrum count	Percentage of total spectra	Percentage sequence coverage
Pap test	Methyltransferase-like protein 7A (Fragment) GN=METTL7A PE=1 SV=1	MET7A_HUMAN	28,299.40	99.20%	1	1	1	0.00156%	6.15%
Tumor tissue	Methyltransferase-like protein 7A (Fragment) GN=METTL7A PE=1 SV=1	MET7A_HUMAN	28,299.40	100.00%	3	4	5	0.00383%	20.50%
Tumor tissue	Mevalonate kinase GN=MVK PE=1 SV=1	KIME_HUMAN	42,451.30	100.00%	1	1	2	0.00153%	7.27%
Tumor tissue	MICOS complex subunit GN=APOOL PE=1 SV=1	MIC27_HUMAN	29,205.40	100.00%	1	1	3	0.00230%	19.00%
Tumor tissue	MICOS complex subunit GN=CHCHD3 PE=1 SV=1	MIC19_HUMAN	27,735.70	100.00%	3	4	4	0.00306%	13.80%
Tumor tissue	Microfibril-associated glycoprotein 4 GN=MFAP4 PE=1 SV=1	sp|P55083|MFAP4_HUMAN	31,327.20	100.00%	2	3	4	0.00306%	9.64%
Tumor tissue	Microfibrillar-associated protein 1 GN=MFAP1 PE=1 SV=2	MFAP1_HUMAN	51,958.70	100.00%	2	2	2	0.00153%	8.88%
Tumor tissue	Microsomal glutathione S-transferase 3 GN=MGST3 PE=1 SV=1	MGST3_HUMAN	18,417.00	100.00%	2	2	2	0.00153%	9.87%
Tumor tissue	Microtubule-actin cross-linking factor 1, isoforms 1/2/3/5 GN=MACF1 PE=1 SV=1	H3BQK9_HUMAN	861,084.80	100.00%	43	47	85	0.06510%	13.40%
Tumor tissue	Microtubule-actin cross-linking factor 1, isoforms 1/2/3/5 (Fragment) GN=MACF1 PE=1 SV=1	E9PLY5_HUMAN	159,926.20	100.00%	2	2	24	0.01840%	18.10%
Tumor tissue	Microtubule-associated protein GN=MAP4 PE=1 SV=1	E7EVA0_HUMAN	245,445.50	100.00%	13	14	37	0.02830%	13.30%
Swab	Microtubule-associated protein GN=MAPT PE=1 SV=1	sp|P10636|TAU_HUMAN	78,901.60	100.00%	2	3	5	0.00354%	2.45%
Tumor tissue	Microtubule-associated protein 1A GN=MAP1A PE=1 SV=1	sp|P78559|MAP1A_HUMAN	331,260.60	100.00%	5	5	5	0.00383%	2.47%
Swab	Microtubule-associated protein 1B GN=MAP1B PE=1 SV=2	MAP1B_HUMAN	270,634.40	100.00%	1	1	2	0.00142%	1.01%
Tumor tissue	Microtubule-associated protein 1B GN=MAP1B PE=1 SV=2	MAP1B_HUMAN	270,634.40	100.00%	10	10	10	0.00766%	6.85%

Biological sample	Protein name	Protein accession numbers	Protein molecular weight (Da)	Protein identification probability	Exclusive unique peptide count	Exclusive unique spectrum count	Total spectrum count	Percentage of total spectra	Percentage sequence coverage
Swab	Microtubule-associated protein RP/EB family member 1 GN=MAPRE1 PE=1 SV=3	MARE1_HUMAN	29,999.50	100.00%	2	3	5	0.00354%	10.80%
Tumor tissue	Microtubule-associated protein RP/EB family member 1 GN=MAPRE1 PE=1 SV=3	MARE1_HUMAN	29,999.50	100.00%	5	8	12	0.00919%	31.30%
Tumor tissue	Midasin GN=MDN1 PE=1 SV=2	MDN1_HUMAN	632,826.30	100.00%	2	2	3	0.00230%	0.43%
Tumor tissue	Migration and invasion enhancer 1 GN=MIEN1 PE=1 SV=1	MIEN1_HUMAN	12,403.00	99.80%	2	2	2	0.00153%	18.10%
Tumor tissue	Mimecan GN=OGN PE=1 SV=1	MIME_HUMAN	33,925.00	100.00%	5	6	10	0.00766%	17.10%
Tumor tissue	Minor histocompatibility protein HA-1 GN=HMHA1 PE=1 SV=2	sp|Q92619|HMHA1_HUMAN	124,615.00	100.00%	1	1	3	0.00230%	2.99%
Tumor tissue	MIP18 family protein FAM96A GN=FAM96A PE=1 SV=1	sp|Q9H5X1|FA96A_HUMAN	18,355.20	99.90%	2	2	2	0.00153%	14.40%
Tumor tissue	Mitochondrial 10-formyltetrahydrofolate dehydrogenase GN=ALDH1L2 PE=1 SV=2	sp|Q3SY69|AL1L2_HUMAN	101,748.30	100.00%	9	9	11	0.00842%	16.30%
Tumor tissue	Mitochondrial 2-oxoglutarate/malate carrier protein (Fragment) GN=SLC25A11 PE=1 SV=1	sp|Q02978|M2OM_HUMAN	32,183.70	100.00%	6	7	7	0.00536%	25.30%
Tumor tissue	Mitochondrial antiviral-signaling protein GN=MAVS PE=1 SV=2	sp|Q7Z434|MAVS_HUMAN	56,527.90	100.00%	8	9	10	0.00766%	23.50%
Tumor tissue	Mitochondrial carrier homolog 2 GN=MTCH2 PE=1 SV=1	MTCH2_HUMAN	33,331.60	100.00%	4	7	8	0.00612%	20.50%
Tumor tissue	Mitochondrial chaperone BCS1 GN=BCS1L PE=1 SV=1	BCS1_HUMAN	47,535.30	100.00%	3	4	5	0.00383%	11.50%
Tumor tissue	Mitochondrial fission 1 protein GN=FIS1 PE=1 SV=2	FIS1_HUMAN	16,938.40	100.00%	2	3	4	0.00306%	16.40%
Tumor tissue	Mitochondrial fission regulator 1-like GN=MTFR1L PE=1 SV=1	sp|Q9H019|MFR1L_HUMAN	21,191.50	100.00%	2	2	2	0.00153%	14.90%

Biological sample	Protein name	Protein accession numbers	Protein molecular weight (Da)	Protein identification probability	Exclusive unique peptide count	Exclusive unique spectrum count	Total spectrum count	Percentage of total spectra	Percentage sequence coverage
Tumor tissue	Mitochondrial import inner membrane translocase subunit Tim13 GN=TIMM13 PE=1 SV=1	TIM13_HUMAN	10,500.20	100.00%	3	4	6	0.00459%	36.80%
Tumor tissue	Mitochondrial import inner membrane translocase subunit TIM14 GN=DNAJC19 PE=1 SV=3	sp|Q96DA6|TIM14_HUMAN	12,499.10	100.00%	2	2	2	0.00153%	27.60%
Tumor tissue	Mitochondrial import inner membrane translocase subunit TIM44 GN=TIMM44 PE=1 SV=2	TIM44_HUMAN	51,357.10	100.00%	8	9	13	0.00995%	20.10%
Tumor tissue	Mitochondrial import receptor subunit TOM22 homolog GN=TOMM22 PE=1 SV=3	TOM22_HUMAN	15,522.10	100.00%	4	5	7	0.00536%	33.80%
Tumor tissue	Mitochondrial import receptor subunit TOM34 GN=TOMM34 PE=1 SV=2	TOM34_HUMAN	34,560.30	100.00%	9	10	10	0.00766%	35.60%
Tumor tissue	Mitochondrial import receptor subunit TOM40 homolog GN=TOMM40 PE=1 SV=1	sp|O96008|TOM40_HUMAN	37,893.20	100.00%	6	6	8	0.00612%	28.00%
Tumor tissue	Mitochondrial import receptor subunit TOM70 GN=TOMM70 PE=1 SV=1	TOM70_HUMAN	67,457.20	100.00%	5	6	6	0.00459%	10.50%
Tumor tissue	Mitochondrial intermediate peptidase GN=MIPEP PE=1 SV=2	MIPEP_HUMAN	80,643.30	100.00%	2	2	2	0.00153%	4.35%
Tumor tissue	Mitochondrial Rho GTPase 2 GN=RHOT2 PE=1 SV=2	sp|Q8IXI1|MIRO2_HUMAN	68,117.60	100.00%	3	3	3	0.00230%	9.22%
Tumor tissue	Mitochondrial ribonuclease P protein 1 GN=TRMT10C PE=1 SV=2	MRRP1_HUMAN	47,348.90	100.00%	3	3	3	0.00230%	10.20%
Tumor tissue	Mitochondrial thymidine kinase 2 GN=TK2 PE=1 SV=1	sp|O00142|KITM_HUMAN	32,873.00	100.00%	3	3	3	0.00230%	14.30%
Tumor tissue	Mitochondrial-processing peptidase subunit alpha GN=PMPCA PE=1 SV=2	sp|Q10713|MPPA_HUMAN	58,253.80	100.00%	4	5	6	0.00459%	11.40%
Tumor tissue	Mitochondrial-processing peptidase subunit beta GN=PMPCB PE=1 SV=2	MPPB_HUMAN	54,367.10	100.00%	3	3	3	0.00230%	6.54%

Biological sample	Protein name	Protein accession numbers	Protein molecular weight (Da)	Protein identification probability	Exclusive unique peptide count	Exclusive unique spectrum count	Total spectrum count	Percentage of total spectra	Percentage sequence coverage
Tumor tissue	Mitofusin-2 GN=MFN2 PE=1 SV=3	sp|O95140|MFN2_HUMAN	86,402.60	100.00%	2	3	3	0.00230%	4.89%
Swab	Mitogen-activated protein kinase 1 GN=MAPK1 PE=1 SV=3	sp|P28482|MK01_HUMAN	41,391.90	100.00%	3	3	7	0.00496%	20.30%
Tumor tissue	Mitogen-activated protein kinase 1 GN=MAPK1 PE=1 SV=3	sp|P28482|MK01_HUMAN	41,391.90	100.00%	4	5	11	0.00842%	18.90%
Tumor tissue	Mitogen-activated protein kinase 13 GN=MAPK13 PE=1 SV=1	sp|O15264|MK13_HUMAN	42,090.90	100.00%	2	2	3	0.00230%	8.49%
Tumor tissue	Mitogen-activated protein kinase 3 GN=MAPK3 PE=1 SV=4	sp|P27361|MK03_HUMAN	43,137.40	100.00%	3	6	12	0.00919%	19.00%
Tumor tissue	Mitogen-activated protein kinase kinase kinase kinase GN=MAP4K5 PE=1 SV=1	M4K5_HUMAN	95,026.90	100.00%	2	2	2	0.00153%	3.19%
Tumor tissue	Mitotic spindle assembly checkpoint protein MAD1 GN=MAD1L1 PE=1 SV=2	sp|Q9Y6D9|MD1L1_HUMAN	83,068.40	100.00%	2	2	2	0.00153%	5.01%
Tumor tissue	MKI67 FHA domain-interacting nucleolar phosphoprotein GN=NIFK PE=1 SV=1	MK67I_HUMAN	34,222.90	100.00%	2	2	2	0.00153%	9.22%
Pap test	Moesin GN=MSN PE=1 SV=3	MOES_HUMAN	67,821.80	100.00%	7	11	26	0.04060%	24.80%
Swab	Moesin GN=MSN PE=1 SV=3	MOES_HUMAN	67,821.80	100.00%	11	13	22	0.01560%	26.30%
Tumor tissue	Moesin GN=MSN PE=1 SV=3	MOES_HUMAN	67,821.80	100.00%	22	41	95	0.07270%	47.80%
Tumor tissue	Monocarboxylate transporter 1 GN=SLC16A1 PE=1 SV=3	sp|P53985|MOT1_HUMAN	53,946.00	99.60%	1	1	3	0.00230%	6.40%
Pap test	Monocyte differentiation antigen CD14 GN=CD14 PE=1 SV=2	CD14_HUMAN	40,076.60	100.00%	4	5	8	0.01250%	18.10%
Swab	Monocyte differentiation antigen CD14 GN=CD14 PE=1 SV=2	CD14_HUMAN	40,076.60	100.00%	6	8	15	0.01060%	25.60%
Tumor tissue	Monocyte differentiation antigen CD14 GN=CD14 PE=1 SV=2	CD14_HUMAN	40,076.60	100.00%	7	12	15	0.01150%	25.60%
Tumor tissue	Monofunctional C1-tetrahydrofolate synthase, mitochondrial GN=MTHFD1L PE=1 SV=1	B7ZM99_HUMAN	105,891.00	100.00%	6	6	7	0.00536%	8.00%

Biological sample	Protein name	Protein accession numbers	Protein molecular weight (Da)	Protein identification probability	Exclusive unique peptide count	Exclusive unique spectrum count	Total spectrum count	Percentage of total spectra	Percentage sequence coverage
Tumor tissue	Mov10, Moloney leukemia virus 10, homolog (Mouse), isoform CRA_a GN=MOV10 PE=1 SV=1	Q5JR04_HUMAN	107,213.80	100.00%	1	1	9	0.00689%	11.60%
Tumor tissue	MRG/MORF4L-binding protein GN=MRGBP PE=1 SV=1	MRGBP_HUMAN	22,416.80	100.00%	2	2	2	0.00153%	13.70%
Tumor tissue	mRNA export factor GN=RAE1 PE=1 SV=1	RAE1L_HUMAN	40,968.50	100.00%	1	1	7	0.00536%	19.00%
Tumor tissue	mRNA turnover protein 4 homolog GN=MRTO4 PE=1 SV=2	MRT4_HUMAN	27,561.30	100.00%	2	2	2	0.00153%	7.53%
Pap test	Mucin-16 GN=MUC16 PE=1 SV=3	MUC16_HUMAN	1,519,324.10	100.00%	4	4	4	0.00624%	0.30%
Swab	Mucin-16 GN=MUC16 PE=1 SV=3	MUC16_HUMAN	1,519,324.10	100.00%	13	14	16	0.01130%	1.27%
Tumor tissue	Mucin-16 GN=MUC16 PE=1 SV=3	MUC16_HUMAN	1,519,324.10	100.00%	3	3	5	0.00383%	0.23%
Pap test	Mucin-5AC GN=MUC5AC PE=1 SV=4	MUC5A_HUMAN	585,551.50	100.00%	26	30	51	0.07960%	6.47%
Swab	Mucin-5AC GN=MUC5AC PE=1 SV=4	MUC5A_HUMAN	585,551.50	100.00%	31	33	68	0.04820%	8.81%
Pap test	Mucin-5B GN=MUC5B PE=1 SV=3	MUC5B_HUMAN	596,324.20	100.00%	56	110	382	0.59600%	14.10%
Swab	Mucin-5B GN=MUC5B PE=1 SV=3	MUC5B_HUMAN	596,324.20	100.00%	58	111	423	0.30000%	13.60%
Pap test	Mucin-6 GN=MUC6 PE=1 SV=1	MUC6_HUMAN	257,072.30	100.00%	3	3	3	0.00468%	1.93%
Swab	Mucin-6 GN=MUC6 PE=1 SV=1	MUC6_HUMAN	257,072.30	100.00%	8	8	8	0.00567%	6.03%
Tumor tissue	Multifunctional methyltransferase subunit TRM112-like protein GN=TRMT112 PE=1 SV=1	sp|Q9UI30|TR112_HUMAN	11,971.90	100.00%	3	3	3	0.00230%	37.70%
Tumor tissue	Multimerin-2 GN=MMRN2 PE=1 SV=2	MMRN2_HUMAN	104,408.40	100.00%	5	5	5	0.00383%	8.32%
Tumor tissue	Mycophenolic acid acyl-glucuronide esterase, mitochondrial GN=ABHD10 PE=1 SV=1	sp|Q9NUJ1|ABHDA_HUMAN	33,933.30	100.00%	5	8	8	0.00612%	27.10%
Tumor tissue	Myelin proteolipid protein GN=PLP1 PE=1 SV=2	sp|P60201|MYPR_HUMAN	30,077.70	100.00%	2	3	3	0.00230%	8.66%
Pap test	Myeloid cell nuclear differentiation antigen GN=MNDA PE=1 SV=1	MNDA_HUMAN	45,837.00	100.00%	2	2	2	0.00312%	5.65%
Swab	Myeloid cell nuclear differentiation antigen GN=MNDA PE=1 SV=1	MNDA_HUMAN	45,837.00	100.00%	4	4	5	0.00354%	9.58%
Tumor tissue	Myeloid cell nuclear differentiation antigen GN=MNDA PE=1 SV=1	MNDA_HUMAN	45,837.00	100.00%	6	6	6	0.00459%	28.50%

Biological sample	Protein name	Protein accession numbers	Protein molecular weight (Da)	Protein identification probability	Exclusive unique peptide count	Exclusive unique spectrum count	Total spectrum count	Percentage of total spectra	Percentage sequence coverage
Tumor tissue	Myeloid differentiation primary response protein MyD88 GN=MYD88 PE=1 SV=1	sp|Q99836|MYD88_HUMAN	35,313.70	100.00%	2	2	2	0.00153%	13.30%
Swab	Myeloid-derived growth factor GN=MYDGF PE=1 SV=1	MYDGF_HUMAN	18,794.90	100.00%	2	3	3	0.00213%	15.00%
Tumor tissue	Myeloid-derived growth factor GN=MYDGF PE=1 SV=1	MYDGF_HUMAN	18,794.90	100.00%	3	4	7	0.00536%	20.80%
Tumor tissue	Myosin light chain 3 GN=MYL3 PE=1 SV=3	MYL3_HUMAN	21,932.50	100.00%	2	2	14	0.01070%	19.50%
Tumor tissue	Myosin light chain 6B GN=MYL6B PE=1 SV=1	MYL6B_HUMAN	22,764.10	99.80%	1	1	8	0.00612%	13.90%
Swab	Myosin light polypeptide 6 GN=MYL6 PE=1 SV=1	sp|P60660-2|MYL6_HUMAN	17,088.90	100.00%	1	4	21	0.01490%	57.20%
Pap test	Myosin regulatory light chain 12A GN=MYL12A PE=1 SV=1	ML12A_HUMAN	19,780.30	100.00%	2	2	9	0.01400%	39.00%
Swab	Myosin regulatory light chain 12A GN=MYL12A PE=1 SV=1	ML12A_HUMAN	19,780.30	100.00%	2	2	5	0.00354%	23.70%
Tumor tissue	Myosin regulatory light chain 12A GN=MYL12A PE=1 SV=1	ML12A_HUMAN	19,795.30	100.00%	4	6	62	0.04750%	49.70%
Tumor tissue	Myosin regulatory light polypeptide 9 GN=MYL9 PE=1 SV=4	sp|P24844|MYL9_HUMAN	19,828.10	100.00%	4	6	61	0.04670%	51.20%
Tumor tissue	Myosin-11 GN=MYH11 PE=1 SV=3	sp|P35749|MYH11_HUMAN	227,344.60	100.00%	2	2	371	0.28400%	48.70%
Tumor tissue	Myosin-6 GN=MYH6 PE=1 SV=5	MYH6_HUMAN	223,740.50	100.00%	1	1	3	0.00230%	2.01%
Tumor tissue	Myosin-7 GN=MYH7 PE=1 SV=5	MYH7_HUMAN	223,103.90	100.00%	1	1	3	0.00230%	2.12%
Pap test	Myosin-9 GN=MYH9 PE=1 SV=4	sp|P35579|MYH9_HUMAN	226,537.50	100.00%	28	32	49	0.07650%	23.80%
Swab	Myosin-9 GN=MYH9 PE=1 SV=4	sp|P35579|MYH9_HUMAN	226,537.50	100.00%	22	33	61	0.04320%	19.80%
Tumor tissue	Myosin-9 GN=MYH9 PE=1 SV=4	sp|P35579|MYH9_HUMAN	226,537.50	100.00%	80	192	592	0.45300%	47.10%
Swab	Myotrophin GN=MTPN PE=1 SV=1	MTPN_HUMAN	12,895.30	100.00%	2	5	12	0.00850%	57.70%
Tumor tissue	Myotrophin GN=MTPN PE=1 SV=1	MTPN_HUMAN	5,704.70	100.00%	2	6	11	0.00842%	57.70%
Tumor tissue	Myotubularin-related protein 5 GN=SBF1 PE=1 SV=1	sp|O95248|MTMR5_HUMAN	208,442.80	100.00%	3	4	4	0.00306%	2.68%

Biological sample	Protein name	Protein accession numbers	Protein molecular weight (Da)	Protein identification probability	Exclusive unique peptide count	Exclusive unique spectrum count	Total spectrum count	Percentage of total spectra	Percentage sequence coverage
Tumor tissue	Myotubularin-related protein 6 GN=MTMR6 PE=1 SV=3	sp|Q9Y217|MTMR6_HUMAN	71,969.60	100.00%	2	2	2	0.00153%	4.19%
Pap test	Myristoylated alanine-rich C-kinase substrate GN=MARCKS PE=1 SV=4	MARCS_HUMAN	31,554.20	100.00%	2	3	4	0.00624%	11.10%
Swab	Myristoylated alanine-rich C-kinase substrate GN=MARCKS PE=1 SV=4	MARCS_HUMAN	31,554.20	100.00%	4	4	8	0.00567%	30.40%
Tumor tissue	Myristoylated alanine-rich C-kinase substrate GN=MARCKS PE=1 SV=4	MARCS_HUMAN	31,554.20	100.00%	7	13	19	0.01450%	42.50%
Swab	N(4)-(beta-N-acetylglucosaminyl)-L-asparaginase GN=AGA PE=1 SV=2	ASPG_HUMAN	37,208.40	100.00%	2	2	2	0.00142%	6.65%
Tumor tissue	N(4)-(beta-N-acetylglucosaminyl)-L-asparaginase GN=AGA PE=1 SV=2	ASPG_HUMAN	37,208.40	99.90%	1	1	1	0.00077%	3.47%
Tumor tissue	N(G),N(G)-dimethylarginine dimethylaminohydrolase 2 GN=DDAH2 PE=1 SV=1	DDAH2_HUMAN	29,644.30	100.00%	7	11	13	0.00995%	33.70%
Pap test	Na(+)/H(+) exchange regulatory cofactor NHE-RF1 GN=SLC9A3R1 PE=1 SV=4	sp|O14745|NHRF1_HUMAN	38,868.30	100.00%	3	3	5	0.00780%	15.10%
Swab	Na(+)/H(+) exchange regulatory cofactor NHE-RF1 GN=SLC9A3R1 PE=1 SV=4	sp|O14745|NHRF1_HUMAN	38,868.30	100.00%	5	5	7	0.00496%	18.40%
Tumor tissue	Na(+)/H(+) exchange regulatory cofactor NHE-RF1 GN=SLC9A3R1 PE=1 SV=4	sp|O14745|NHRF1_HUMAN	38,868.30	100.00%	10	15	21	0.01610%	38.50%
Tumor tissue	Na(+)/H(+) exchange regulatory cofactor NHE-RF2 GN=SLC9A3R2 PE=1 SV=2	sp|Q15599|NHRF2_HUMAN	37,413.20	100.00%	6	7	8	0.00612%	26.10%
Pap test	N-acetyl-D-glucosamine kinase GN=NAGK PE=1 SV=4	sp|Q9UJ70|NAGK_HUMAN	37,377.10	100.00%	5	5	7	0.01090%	26.50%
Swab	N-acetyl-D-glucosamine kinase GN=NAGK PE=1 SV=4	sp|Q9UJ70|NAGK_HUMAN	37,377.10	100.00%	6	7	14	0.00992%	29.10%
Tumor tissue	N-acetyl-D-glucosamine kinase GN=NAGK PE=1 SV=4	sp|Q9UJ70|NAGK_HUMAN	37,377.10	100.00%	9	12	23	0.01760%	44.50%

Biological sample	Protein name	Protein accession numbers	Protein molecular weight (Da)	Protein identification probability	Exclusive unique peptide count	Exclusive unique spectrum count	Total spectrum count	Percentage of total spectra	Percentage sequence coverage
Tumor tissue	N-acetylgalactosaminyltransferase 7 GN=GALNT7 PE=1 SV=1	GALT7_HUMAN	75,390.60	100.00%	2	2	2	0.00153%	3.65%
Pap test	N-acetylglucosamine-6-sulfatase GN=GNS PE=1 SV=1	sp|P15586|GNS_HUMAN	65,711.90	100.00%	2	2	5	0.00780%	10.10%
Swab	N-acetylglucosamine-6-sulfatase GN=GNS PE=1 SV=1	sp|P15586|GNS_HUMAN	65,711.90	100.00%	1	1	7	0.00496%	7.88%
Tumor tissue	N-acetylglucosamine-6-sulfatase GN=GNS PE=1 SV=1	sp|P15586|GNS_HUMAN	65,711.90	100.00%	1	1	4	0.00306%	7.88%
Tumor tissue	NACHT, LRR and PYD domains-containing protein 2 (Fragment) GN=NLRP2 PE=1 SV=1	sp|Q9NX02|NALP2_HUMAN	120,489.80	100.00%	2	2	2	0.00153%	3.64%
Tumor tissue	N-acylethanolamine-hydrolyzing acid amidase GN=NAAA PE=1 SV=1	sp|Q02083|NAAA_HUMAN	24,836.60	100.00%	2	3	3	0.00230%	12.70%
Tumor tissue	N-acylglucosamine 2-epimerase GN=RENBP PE=1 SV=2	sp|P51606|RENBP_HUMAN	47,066.10	100.00%	2	2	2	0.00153%	5.57%
Tumor tissue	NAD kinase 2, mitochondrial GN=NADK2 PE=1 SV=2	sp|Q4G0N4|NAKD2_HUMAN	49,433.40	100.00%	7	8	8	0.00612%	21.50%
Tumor tissue	NAD(P) transhydrogenase, mitochondrial GN=NNT PE=1 SV=3	NNTM_HUMAN	113,899.20	100.00%	14	17	20	0.01530%	18.70%
Swab	NAD(P)H-hydrate epimerase GN=NAXE PE=1 SV=2	sp|Q8NCW5|NNRE_HUMAN	31,675.40	99.60%	1	1	2	0.00142%	6.25%
Tumor tissue	NAD(P)H-hydrate epimerase GN=NAXE PE=1 SV=2	sp|Q8NCW5|NNRE_HUMAN	31,675.40	100.00%	3	4	4	0.00306%	18.10%
Tumor tissue	NAD-dependent malic enzyme, mitochondrial GN=ME2 PE=1 SV=1	sp|P23368|MAOM_HUMAN	65,446.20	100.00%	8	8	11	0.00842%	15.60%
Tumor tissue	NADH dehydrogenase (Ubiquinone) flavoprotein 1, 51kDa, isoform CRA_c GN=NDUFV1 PE=1 SV=1	sp|P49821|NDUV1_HUMAN	50,055.00	100.00%	5	5	7	0.00536%	16.80%
Tumor tissue	NADH dehydrogenase [ubiquinone] 1 alpha subcomplex subunit 10, mitochondrial GN=NDUFA10 PE=1 SV=1	E7ESZ7_HUMAN	44,739.40	100.00%	5	7	7	0.00536%	14.60%
Tumor tissue	NADH dehydrogenase [ubiquinone] 1 alpha subcomplex subunit 12 GN=NDUFA12 PE=1 SV=1	sp|Q9UI09|NDUAC_HUMAN	17,114.50	100.00%	2	3	5	0.00383%	32.40%

Biological sample	Protein name	Protein accession numbers	Protein molecular weight (Da)	Protein identification probability	Exclusive unique peptide count	Exclusive unique spectrum count	Total spectrum count	Percentage of total spectra	Percentage sequence coverage
Tumor tissue	NADH dehydrogenase [ubiquinone] 1 alpha subcomplex subunit 13 GN=NDUFA13 PE=1 SV=3	sp|Q9P0J0|NDUAD_HUMAN	16,699.10	100.00%	2	3	12	0.00919%	25.70%
Tumor tissue	NADH dehydrogenase [ubiquinone] 1 alpha subcomplex subunit 2 GN=NDUFA2 PE=1 SV=3	sp|O43678|NDUA2_HUMAN	10,921.80	100.00%	2	2	3	0.00230%	31.30%
Tumor tissue	NADH dehydrogenase [ubiquinone] 1 alpha subcomplex subunit 7 GN=NDUFA7 PE=1 SV=3	NDUA7_HUMAN	12,551.80	100.00%	2	2	2	0.00153%	23.00%
Tumor tissue	NADH dehydrogenase [ubiquinone] 1 alpha subcomplex subunit 9, mitochondrial GN=NDUFA9 PE=1 SV=2	NDUA9_HUMAN	42,510.10	100.00%	8	9	13	0.00995%	30.80%
Tumor tissue	NADH dehydrogenase [ubiquinone] 1 beta subcomplex subunit 10 GN=NDUFB10 PE=1 SV=3	sp|O96000|NDUBA_HUMAN	20,776.70	100.00%	4	6	6	0.00459%	23.30%
Tumor tissue	NADH dehydrogenase [ubiquinone] 1 beta subcomplex subunit 4 GN=NDUFB4 PE=1 SV=3	sp|O95168|NDUB4_HUMAN	15,210.00	100.00%	2	3	3	0.00230%	21.70%
Tumor tissue	NADH dehydrogenase [ubiquinone] 1 beta subcomplex subunit 9 GN=NDUFB9 PE=1 SV=1	NDUB9_HUMAN	20,383.50	100.00%	1	2	4	0.00306%	17.30%
Pap test	NADH dehydrogenase [ubiquinone] flavoprotein 2, mitochondrial GN=NDUFV2 PE=1 SV=1	NDUV2_HUMAN	27,907.60	99.20%	1	1	2	0.00312%	5.16%
Tumor tissue	NADH dehydrogenase [ubiquinone] flavoprotein 2, mitochondrial GN=NDUFV2 PE=1 SV=1	NDUV2_HUMAN	27,391.80	100.00%	4	5	8	0.00612%	17.50%
Pap test	NADH dehydrogenase [ubiquinone] iron-sulfur protein 3, mitochondrial GN=NDUFS3 PE=1 SV=1	sp|O75489|NDUS3_HUMAN	30,241.70	100.00%	1	1	2	0.00312%	12.10%
Tumor tissue	NADH dehydrogenase [ubiquinone] iron-sulfur protein 3, mitochondrial GN=NDUFS3 PE=1 SV=1	sp|O75489|NDUS3_HUMAN	30,241.70	100.00%	4	6	9	0.00689%	31.80%

Biological sample	Protein name	Protein accession numbers	Protein molecular weight (Da)	Protein identification probability	Exclusive unique peptide count	Exclusive unique spectrum count	Total spectrum count	Percentage of total spectra	Percentage sequence coverage
Tumor tissue	NADH dehydrogenase [ubiquinone] iron-sulfur protein 4, mitochondrial GN=NDUFS4 PE=1 SV=1	NDUS4_HUMAN	20,107.90	100.00%	3	3	3	0.00230%	20.00%
Tumor tissue	NADH dehydrogenase [ubiquinone] iron-sulfur protein 5 GN=NDUFS5 PE=1 SV=3	NDUS5_HUMAN	12,518.10	100.00%	2	2	2	0.00153%	20.80%
Tumor tissue	NADH dehydrogenase [ubiquinone] iron-sulfur protein 7, mitochondrial GN=NDUFS7 PE=1 SV=1	sp|O75251-2|NDUS7_HUMAN	25,383.20	100.00%	2	2	2	0.00153%	9.75%
Tumor tissue	NADH dehydrogenase [ubiquinone] iron-sulfur protein 8, mitochondrial (Fragment) GN=NDUFS8 PE=1 SV=1	NDUS8_HUMAN	23,705.90	100.00%	1	1	3	0.00230%	16.80%
Tumor tissue	NADH-cytochrome b5 reductase 1 GN=CYB5R1 PE=1 SV=1	NB5R1_HUMAN	34,096.10	100.00%	6	6	6	0.00459%	23.00%
Swab	NADH-cytochrome b5 reductase 2 GN=CYB5R2 PE=1 SV=1	sp|Q6BCY4|NB5R2_HUMAN	31,459.90	98.40%	1	1	1	0.00071%	2.90%
Tumor tissue	NADH-cytochrome b5 reductase 2 GN=CYB5R2 PE=1 SV=1	sp|Q6BCY4|NB5R2_HUMAN	31,459.90	100.00%	3	3	4	0.00306%	18.50%
Tumor tissue	NADH-ubiquinone oxidoreductase chain 4 GN=MT-ND4 PE=1 SV=1	NU4M_HUMAN	51,585.50	100.00%	2	2	2	0.00153%	4.14%
Pap test	NADPH--cytochrome P450 reductase GN=POR PE=1 SV=2	NCPR_HUMAN	76,690.90	99.80%	1	1	1	0.00156%	1.62%
Tumor tissue	NADPH--cytochrome P450 reductase GN=POR PE=1 SV=2	NCPR_HUMAN	76,690.90	100.00%	9	10	12	0.00919%	19.20%
Tumor tissue	N-alpha-acetyltransferase 15, NatA auxiliary subunit GN=NAA15 PE=1 SV=1	sp|Q9BXJ9|NAA15_HUMAN	101,206.90	100.00%	8	8	10	0.00766%	12.80%
Tumor tissue	N-alpha-acetyltransferase 25, NatB auxiliary subunit GN=NAA25 PE=1 SV=1	sp|Q14CX7|NAA25_HUMAN	112,297.80	100.00%	2	2	2	0.00153%	1.54%
Tumor tissue	N-alpha-acetyltransferase 50 GN=NAA50 PE=1 SV=1	sp|Q9GZZ1|NAA50_HUMAN	19,311.80	100.00%	2	2	2	0.00153%	30.90%

Biological sample	Protein name	Protein accession numbers	Protein molecular weight (Da)	Protein identification probability	Exclusive unique peptide count	Exclusive unique spectrum count	Total spectrum count	Percentage of total spectra	Percentage sequence coverage
Tumor tissue	Nascent polypeptide-associated complex subunit alpha, muscle-specific form GN=NACA PE=1 SV=1	sp|E9PAV3|NACAM_HUMAN	205,422.80	100.00%	2	4	9	0.00689%	2.02%
Pap test	Nectin-4 GN=NECTIN4 PE=1 SV=1	sp|Q96NY8|NECT4_HUMAN	55,453.10	100.00%	3	3	3	0.00468%	7.45%
Tumor tissue	NEDD8 GN=NEDD8 PE=1 SV=1	NEDD8_HUMAN	9,072.00	100.00%	1	1	7	0.00536%	34.60%
Tumor tissue	Negative elongation factor C/D GN=TH1L PE=1 SV=1	sp|Q8IXH7|NELFD_HUMAN	67,347.20	100.00%	4	4	4	0.00306%	7.85%
Tumor tissue	Nesprin-1 GN=SYNE1 PE=1 SV=2	E7ENN3_HUMAN	964,855.80	100.00%	1	1	7	0.00536%	1.61%
Tumor tissue	Nestin GN=NES PE=1 SV=2	NEST_HUMAN	177,437.10	100.00%	15	17	17	0.01300%	13.90%
Pap test	Neudesin GN=NENF PE=1 SV=1	NENF_HUMAN	18,857.10	99.20%	1	1	1	0.00156%	8.72%
Tumor tissue	Neudesin GN=NENF PE=1 SV=1	NENF_HUMAN	18,857.10	100.00%	2	2	2	0.00153%	16.90%
Tumor tissue	Neurabin-2 GN=PPP1R9B PE=1 SV=1	NEB2_HUMAN	89,334.30	100.00%	2	2	3	0.00230%	4.16%
Pap test	Neuroblast differentiation-associated protein AHNAK GN=AHNAK PE=1 SV=2	sp|Q09666|AHNK_HUMAN	629,104.40	100.00%	49	55	127	0.19800%	21.90%
Swab	Neuroblast differentiation-associated protein AHNAK GN=AHNAK PE=1 SV=2	AHNK_HUMAN	629,104.40	100.00%	1	1	32	0.02270%	6.94%
Tumor tissue	Neuroblast differentiation-associated protein AHNAK GN=AHNAK PE=1 SV=2	sp|Q09666|AHNK_HUMAN	629,104.40	100.00%	150	212	355	0.27200%	42.50%
Tumor tissue	Neurocalcin-delta GN=NCALD PE=1 SV=2	NCALD_HUMAN	22,246.30	100.00%	1	1	2	0.00153%	13.00%
Tumor tissue	Neuronal migration protein doublecortin GN=DCX PE=1 SV=1	A0A140LJL1_HUMAN	49,318.90	100.00%	1	1	2	0.00153%	6.35%
Tumor tissue	Neuropilin GN=NRP1 PE=1 SV=1	Q5JWQ6_HUMAN	81,991.90	100.00%	1	1	4	0.00306%	9.25%
Tumor tissue	Neuropilin 2 isoform A (Fragment) GN=NRP2 PE=1 SV=1	sp|O60462|NRP2_HUMAN	104,833.20	100.00%	2	2	2	0.00153%	2.36%
Tumor tissue	Neuropilin-1 GN=NRP1 PE=1 SV=1	E7EX60_HUMAN	72,095.80	98.70%	1	1	4	0.00306%	9.52%

Biological sample	Protein name	Protein accession numbers	Protein molecular weight (Da)	Protein identification probability	Exclusive unique peptide count	Exclusive unique spectrum count	Total spectrum count	Percentage of total spectra	Percentage sequence coverage
Tumor tissue	Neutral alpha-glucosidase AB GN=GANAB PE=1 SV=3	sp|Q14697|GANAB_HUMAN	106,875.90	100.00%	1	1	75	0.05740%	35.60%
Tumor tissue	Neutral amino acid transporter B(0) GN=SLC1A5 PE=1 SV=2	sp|Q15758|AAAT_HUMAN	56,599.80	100.00%	3	3	5	0.00383%	6.65%
Tumor tissue	Neutral cholesterol ester hydrolase 1 GN=NCEH1 PE=1 SV=1	sp|Q6PIU2|NCEH1_HUMAN	49,063.20	100.00%	4	4	4	0.00306%	11.20%
Pap test	Neutrophil collagenase GN=MMP8 PE=1 SV=1	MMP8_HUMAN	53,414.20	100.00%	6	7	22	0.03430%	17.60%
Swab	Neutrophil collagenase GN=MMP8 PE=1 SV=1	MMP8_HUMAN	53,414.20	100.00%	3	3	8	0.00567%	8.57%
Pap test	Neutrophil cytosol factor 1 GN=NCF1 PE=1 SV=3	sp|P14598|NCF1_HUMAN	44,652.80	100.00%	2	2	2	0.00312%	8.21%
Swab	Neutrophil cytosol factor 1 GN=NCF1 PE=1 SV=3	sp|P14598|NCF1_HUMAN	44,652.80	100.00%	2	2	7	0.00496%	16.20%
Tumor tissue	Neutrophil cytosol factor 1 GN=NCF1 PE=1 SV=3	sp|P14598|NCF1_HUMAN	44,652.80	100.00%	3	3	3	0.00230%	11.30%
Pap test	Neutrophil cytosol factor 2 GN=NCF2 PE=1 SV=2	sp|P19878|NCF2_HUMAN	59,762.20	99.80%	1	1	1	0.00156%	1.90%
Swab	Neutrophil cytosol factor 2 GN=NCF2 PE=1 SV=2	sp|P19878|NCF2_HUMAN	59,762.20	100.00%	4	4	5	0.00354%	9.51%
Tumor tissue	Neutrophil cytosol factor 2 GN=NCF2 PE=1 SV=2	sp|P19878|NCF2_HUMAN	59,762.20	100.00%	2	2	2	0.00153%	4.56%
Swab	Neutrophil cytosol factor 4 GN=NCF4 PE=1 SV=2	sp|Q15080|NCF4_HUMAN	39,033.30	100.00%	3	3	4	0.00283%	11.20%
Tumor tissue	Neutrophil cytosol factor 4 GN=NCF4 PE=1 SV=2	sp|Q15080|NCF4_HUMAN	39,033.30	100.00%	2	2	2	0.00153%	6.49%
Pap test	Neutrophil defensin 3 GN=DEFA3 PE=1 SV=1	DEF3_HUMAN	10,245.10	100.00%	4	7	48	0.07490%	20.20%
Swab	Neutrophil defensin 3 GN=DEFA3 PE=1 SV=1	DEF3_HUMAN	10,245.10	100.00%	4	4	63	0.04460%	20.20%
Pap test	Neutrophil elastase GN=ELANE PE=1 SV=1	ELNE_HUMAN	28,517.40	100.00%	5	9	18	0.02810%	23.60%
Swab	Neutrophil elastase GN=ELANE PE=1 SV=1	ELNE_HUMAN	28,517.40	100.00%	2	2	10	0.00709%	7.12%
Tumor tissue	Neutrophil elastase GN=ELANE PE=1 SV=1	ELNE_HUMAN	28,517.40	100.00%	1	1	1	0.00077%	3.75%

Biological sample	Protein name	Protein accession numbers	Protein molecular weight (Da)	Protein identification probability	Exclusive unique peptide count	Exclusive unique spectrum count	Total spectrum count	Percentage of total spectra	Percentage sequence coverage
Pap test	Neutrophil gelatinase-associated lipocalin GN=LCN2 PE=1 SV=2	sp|P80188|NGAL_HUMAN	22,588.90	100.00%	8	20	91	0.14200%	57.10%
Swab	Neutrophil gelatinase-associated lipocalin GN=LCN2 PE=1 SV=2	sp|P80188|NGAL_HUMAN	22,588.90	100.00%	7	16	70	0.04960%	44.40%
Tumor tissue	Neutrophil gelatinase-associated lipocalin GN=LCN2 PE=1 SV=2	sp|P80188|NGAL_HUMAN	22,588.90	100.00%	1	1	1	0.00077%	7.58%
Tumor tissue	NF-kappa-B inhibitor epsilon GN=NFKBIE PE=1 SV=3	IKBE_HUMAN	52,864.30	100.00%	2	2	2	0.00153%	6.60%
Tumor tissue	NHL repeat-containing protein 2 GN=NHLRC2 PE=1 SV=1	sp|Q8NBF2|NHLC2_HUMAN	79,445.50	100.00%	3	3	3	0.00230%	4.68%
Tumor tissue	Nibrin GN=NBN PE=1 SV=1	NBN_HUMAN	84,961.10	100.00%	6	6	6	0.00459%	12.90%
Swab	Nicotinamide N-methyltransferase GN=NNMT PE=1 SV=1	NNMT_HUMAN	29,575.00	99.70%	1	1	1	0.00071%	8.33%
Tumor tissue	Nicotinamide N-methyltransferase GN=NNMT PE=1 SV=1	NNMT_HUMAN	29,575.00	100.00%	6	8	13	0.00995%	27.70%
Pap test	Nicotinamide phosphoribosyltransferase GN=NAMPT PE=1 SV=1	NAMPT_HUMAN	55,522.50	100.00%	10	13	21	0.03280%	30.50%
Swab	Nicotinamide phosphoribosyltransferase GN=NAMPT PE=1 SV=1	NAMPT_HUMAN	55,522.50	100.00%	13	16	29	0.02060%	36.70%
Tumor tissue	Nicotinamide phosphoribosyltransferase GN=NAMPT PE=1 SV=1	NAMPT_HUMAN	55,522.50	100.00%	14	19	21	0.01610%	38.70%
Pap test	Nicotinate phosphoribosyltransferase GN=NAPRT PE=1 SV=2	sp|Q6XQN6|PNCB_HUMAN	57,578.50	100.00%	2	2	2	0.00312%	4.28%
Swab	Nicotinate phosphoribosyltransferase GN=NAPRT PE=1 SV=2	sp|Q6XQN6|PNCB_HUMAN	57,578.50	100.00%	6	7	9	0.00638%	15.10%
Tumor tissue	Nicotinate phosphoribosyltransferase GN=NAPRT PE=1 SV=2	sp|Q6XQN6|PNCB_HUMAN	57,578.50	100.00%	5	5	6	0.00459%	11.20%

Biological sample	Protein name	Protein accession numbers	Protein molecular weight (Da)	Protein identification probability	Exclusive unique peptide count	Exclusive unique spectrum count	Total spectrum count	Percentage of total spectra	Percentage sequence coverage
Tumor tissue	Nicotinate-nucleotide pyrophosphorylase [carboxylating] GN=QPRT PE=1 SV=3	NADC_HUMAN	30,845.30	100.00%	2	2	2	0.00153%	7.41%
Tumor tissue	Nischarin GN=NISCH PE=1 SV=3	sp|Q9Y2I1|NISCH_HUMAN	166,629.80	100.00%	2	2	2	0.00153%	1.86%
Swab	N-myc-interactor GN=NMI PE=1 SV=2	NMI_HUMAN	35,056.90	99.90%	1	1	1	0.00071%	3.26%
Tumor tissue	N-myc-interactor GN=NMI PE=1 SV=2	NMI_HUMAN	35,056.90	100.00%	5	5	5	0.00383%	23.80%
Tumor tissue	Nodal modulator 3 GN=NOMO3 PE=1 SV=1	sp|Q5JPE7|NOMO2_HUMAN	139,382.50	100.00%	18	22	28	0.02140%	20.00%
Tumor tissue	Non-erythrocytic beta-spectrin 4 GN=SPTBN4 PE=1 SV=2	sp|Q9H254|SPTN4_HUMAN	289,060.10	99.80%	1	1	5	0.00383%	1.33%
Tumor tissue	Non-POU domain-containing octamer-binding protein GN=NONO PE=1 SV=4	sp|Q15233|NONO_HUMAN	54,231.60	100.00%	12	18	31	0.02370%	30.40%
Pap test	Non-secretory ribonuclease GN=RNASE2 PE=1 SV=2	RNAS2_HUMAN	18,353.70	100.00%	3	4	6	0.00936%	19.90%
Swab	Non-secretory ribonuclease GN=RNASE2 PE=1 SV=2	RNAS2_HUMAN	18,353.70	100.00%	3	4	6	0.00425%	21.10%
Tumor tissue	Non-secretory ribonuclease GN=RNASE2 PE=1 SV=2	RNAS2_HUMAN	18,353.70	100.00%	2	3	5	0.00383%	9.32%
Swab	Non-syndromic hearing impairment protein 5 GN=DFNA5 PE=1 SV=2	sp|O60443|DFNA5_HUMAN	54,555.90	99.80%	1	1	1	0.00071%	4.64%
Tumor tissue	Non-syndromic hearing impairment protein 5 GN=DFNA5 PE=1 SV=2	sp|O60443|DFNA5_HUMAN	54,555.90	100.00%	3	3	3	0.00230%	10.10%
Pap test	Normal mucosa of esophagus-specific gene 1 protein GN=NMES1 PE=2 SV=1	NMES1_HUMAN	9,617.40	99.90%	1	1	2	0.00312%	41.00%
Tumor tissue	Normal mucosa of esophagus-specific gene 1 protein GN=NMES1 PE=2 SV=1	NMES1_HUMAN	9,617.40	100.00%	1	1	3	0.00230%	42.20%
Tumor tissue	Nuclear autoantigenic sperm protein GN=NASP PE=1 SV=2	sp|P49321|NASP_HUMAN	85,235.50	100.00%	5	5	9	0.00689%	17.30%
Tumor tissue	Nuclear cap-binding protein subunit 1 GN=NCBP1 PE=1 SV=1	NCBP1_HUMAN	91,841.10	100.00%	6	7	7	0.00536%	9.11%

Biological sample	Protein name	Protein accession numbers	Protein molecular weight (Da)	Protein identification probability	Exclusive unique peptide count	Exclusive unique spectrum count	Total spectrum count	Percentage of total spectra	Percentage sequence coverage
Tumor tissue	Nuclear factor 1 GN=NFIB PE=1 SV=1	A0A1B0GW37_HUMAN	53,841.90	100.00%	1	1	3	0.00230%	7.95%
Tumor tissue	Nuclear factor NF-kappa-B p100 subunit GN=NFKB2 PE=1 SV=4	sp|Q00653|NFKB2_HUMAN	96,750.70	100.00%	7	9	10	0.00766%	12.90%
Tumor tissue	Nuclear fragile X mental retardation-interacting protein 2 GN=NUFIP2 PE=1 SV=1	sp|Q7Z417|NUFP2_HUMAN	76,121.10	100.00%	3	3	3	0.00230%	7.19%
Tumor tissue	Nuclear inhibitor of protein phosphatase 1 GN=PPP1R8 PE=1 SV=2	sp|Q12972|PP1R8_HUMAN	38,479.80	100.00%	4	6	6	0.00459%	22.80%
Pap test	Nuclear migration protein nudC GN=NUDC PE=1 SV=1	NUDC_HUMAN	38,243.70	100.00%	3	3	3	0.00468%	11.50%
Tumor tissue	Nuclear migration protein nudC GN=NUDC PE=1 SV=1	NUDC_HUMAN	38,243.70	100.00%	6	6	7	0.00536%	23.00%
Pap test	Nuclear mitotic apparatus protein 1 GN=NUMA1 PE=1 SV=2	sp|Q14980|NUMA1_HUMAN	238,259.70	100.00%	2	2	5	0.00780%	2.60%
Tumor tissue	Nuclear mitotic apparatus protein 1 GN=NUMA1 PE=1 SV=2	sp|Q14980|NUMA1_HUMAN	238,259.70	100.00%	25	37	87	0.06660%	34.20%
Tumor tissue	Nuclear mitotic apparatus protein 1 (Fragment) GN=NUMA1 PE=1 SV=1	H0YFY6_HUMAN	107,412.00	99.80%	1	1	46	0.03520%	37.10%
Tumor tissue	Nuclear pore complex protein Nup107 GN=NUP107 PE=1 SV=1	sp|P57740|NU107_HUMAN	106,376.70	100.00%	3	3	3	0.00230%	4.11%
Tumor tissue	Nuclear pore complex protein Nup133 GN=NUP133 PE=1 SV=2	NU133_HUMAN	128,984.00	100.00%	7	7	7	0.00536%	9.34%
Tumor tissue	Nuclear pore complex protein Nup153 GN=NUP153 PE=1 SV=2	sp|P49790|NU153_HUMAN	153,939.20	100.00%	5	6	6	0.00459%	5.02%
Tumor tissue	Nuclear pore complex protein Nup155 GN=NUP155 PE=1 SV=1	sp|O75694|NU155_HUMAN	148,097.20	100.00%	9	10	10	0.00766%	10.30%
Tumor tissue	Nuclear pore complex protein Nup160 (Fragment) GN=NUP160 PE=1 SV=2	sp|Q12769|NU160_HUMAN	148,975.50	100.00%	6	6	6	0.00459%	5.18%
Tumor tissue	Nuclear pore complex protein Nup205 GN=NUP205 PE=1 SV=3	NU205_HUMAN	227,928.40	100.00%	7	7	7	0.00536%	4.27%
Tumor tissue	Nuclear pore complex protein Nup85 GN=NUP85 PE=1 SV=1	sp|Q9BW27|NUP85_HUMAN	75,022.60	100.00%	3	3	3	0.00230%	6.71%

Biological sample	Protein name	Protein accession numbers	Protein molecular weight (Da)	Protein identification probability	Exclusive unique peptide count	Exclusive unique spectrum count	Total spectrum count	Percentage of total spectra	Percentage sequence coverage
Tumor tissue	Nuclear pore complex protein Nup88 (Fragment) GN=NUP88 PE=1 SV=1	J3KMX1_HUMAN	78,883.90	100.00%	3	3	3	0.00230%	6.03%
Tumor tissue	Nuclear pore complex protein Nup93 GN=NUP93 PE=1 SV=2	sp|Q8N1F7|NUP93_HUMAN	93,491.20	100.00%	6	7	9	0.00689%	9.65%
Tumor tissue	Nuclear pore glycoprotein p62 GN=NUP62 PE=1 SV=3	NUP62_HUMAN	53,256.30	100.00%	3	4	4	0.00306%	9.58%
Tumor tissue	Nuclear pore membrane glycoprotein 210 GN=NUP210 PE=1 SV=3	sp|Q8TEM1|PO210_HUMAN	205,109.80	100.00%	12	12	13	0.00995%	9.17%
Tumor tissue	Nuclear receptor coactivator 5 GN=NCOA5 PE=1 SV=2	NCOA5_HUMAN	65,537.70	100.00%	5	5	6	0.00459%	11.20%
Tumor tissue	Nuclear receptor corepressor 2 GN=NCOR2 PE=1 SV=1	sp|Q9Y618-5|NCOR2_HUMAN	268,044.90	100.00%	2	2	2	0.00153%	1.18%
Swab	Nuclear receptor-binding protein GN=NRBP1 PE=1 SV=1	NRBP_HUMAN	60,829.80	99.60%	1	1	1	0.00071%	2.21%
Tumor tissue	Nuclear receptor-binding protein GN=NRBP1 PE=1 SV=1	NRBP_HUMAN	59,844.60	100.00%	2	3	3	0.00230%	5.34%
Tumor tissue	Nuclear RNA export factor 1 (Fragment) GN=NXF1 PE=1 SV=1	sp|Q9UBU9|NXF1_HUMAN	68,109.90	100.00%	2	2	3	0.00230%	8.13%
Tumor tissue	Nuclear ubiquitous casein and cyclin-dependent kinase substrate 1 GN=NUCKS1 PE=1 SV=1	sp|Q9H1E3|NUCKS_HUMAN	27,296.70	100.00%	2	2	3	0.00230%	11.90%
Tumor tissue	Nuclear-interacting partner of ALK GN=ZC3HC1 PE=1 SV=1	sp|Q86WB0|NIPA_HUMAN	50,562.50	100.00%	2	3	3	0.00230%	6.97%
Swab	Nuclease-sensitive element-binding protein 1 (Fragment) GN=YBX1 PE=1 SV=1	H0Y449_HUMAN	42,015.90	99.90%	1	1	3	0.00213%	9.63%
Tumor tissue	Nuclease-sensitive element-binding protein 1 (Fragment) GN=YBX1 PE=1 SV=1	H0Y449_HUMAN	42,015.90	100.00%	3	7	14	0.01070%	25.40%
Pap test	Nucleobindin 2, isoform CRA_b GN=NUCB2 PE=1 SV=1	sp|P80303|NUCB2_HUMAN	50,229.20	99.20%	1	1	1	0.00156%	2.14%
Tumor tissue	Nucleobindin 2, isoform CRA_b GN=NUCB2 PE=1 SV=1	sp|P80303|NUCB2_HUMAN	50,224.30	100.00%	9	10	11	0.00842%	22.60%

Biological sample	Protein name	Protein accession numbers	Protein molecular weight (Da)	Protein identification probability	Exclusive unique peptide count	Exclusive unique spectrum count	Total spectrum count	Percentage of total spectra	Percentage sequence coverage
Pap test	Nucleobindin-1 GN=NUCB1 PE=1 SV=4	NUCB1_HUMAN	53,879.60	100.00%	4	4	4	0.00624%	9.98%
Swab	Nucleobindin-1 GN=NUCB1 PE=1 SV=4	NUCB1_HUMAN	53,879.60	100.00%	2	2	2	0.00142%	5.64%
Tumor tissue	Nucleobindin-1 GN=NUCB1 PE=1 SV=4	NUCB1_HUMAN	53,879.60	100.00%	9	12	15	0.01150%	26.50%
Tumor tissue	Nucleolar and coiled-body phosphoprotein 1 (Fragment) GN=NOLC1 PE=1 SV=1	sp|Q14978|NOLC1_HUMAN	74,656.10	100.00%	4	6	7	0.00536%	7.63%
Tumor tissue	Nucleolar complex protein 2 homolog GN=NOC2L PE=1 SV=4	NOC2L_HUMAN	84,921.20	100.00%	2	2	2	0.00153%	3.47%
Tumor tissue	Nucleolar complex protein 3 homolog GN=NOC3L PE=1 SV=1	NOC3L_HUMAN	92,552.20	100.00%	3	3	3	0.00230%	6.00%
Tumor tissue	Nucleolar GTP-binding protein 1 GN=GTPBP4 PE=1 SV=3	sp|Q9BZE4|NOG1_HUMAN	73,967.30	100.00%	7	8	8	0.00612%	14.80%
Tumor tissue	Nucleolar GTP-binding protein 2 GN=GNL2 PE=1 SV=1	NOG2_HUMAN	83,656.00	100.00%	2	2	2	0.00153%	2.87%
Tumor tissue	Nucleolar protein 56 GN=NOP56 PE=1 SV=4	NOP56_HUMAN	66,052.00	100.00%	12	20	29	0.02220%	26.60%
Tumor tissue	Nucleolar protein 58 GN=NOP58 PE=1 SV=1	NOP58_HUMAN	59,580.20	100.00%	7	9	12	0.00919%	18.30%
Tumor tissue	Nucleolar protein 9 GN=NOP9 PE=1 SV=1	sp|Q86U38|NOP9_HUMAN	69,438.10	100.00%	3	4	6	0.00459%	6.45%
Tumor tissue	Nucleolar RNA helicase 2 GN=DDX21 PE=1 SV=5	sp|Q9NR30|DDX21_HUMAN	87,346.00	100.00%	13	14	19	0.01450%	24.60%
Pap test	Nucleolin GN=NCL PE=1 SV=3	NUCL_HUMAN	76,615.90	100.00%	4	4	4	0.00624%	7.32%
Swab	Nucleolin GN=NCL PE=1 SV=3	NUCL_HUMAN	76,615.90	100.00%	7	7	9	0.00638%	11.30%
Tumor tissue	Nucleolin GN=NCL PE=1 SV=3	NUCL_HUMAN	76,615.90	100.00%	18	23	50	0.03830%	23.80%
Pap test	Nucleophosmin GN=NPM1 PE=1 SV=2	sp|P06748|NPM_HUMAN	32,575.50	99.90%	1	1	1	0.00156%	4.42%
Swab	Nucleophosmin GN=NPM1 PE=1 SV=2	sp|P06748|NPM_HUMAN	32,575.50	100.00%	4	6	12	0.00850%	17.00%
Tumor tissue	Nucleophosmin GN=NPM1 PE=1 SV=2	sp|P06748|NPM_HUMAN	32,575.50	100.00%	7	22	74	0.05660%	34.00%

Biological sample	Protein name	Protein accession numbers	Protein molecular weight (Da)	Protein identification probability	Exclusive unique peptide count	Exclusive unique spectrum count	Total spectrum count	Percentage of total spectra	Percentage sequence coverage
Tumor tissue	Nucleoporin NUP188 homolog GN=NUP188 PE=1 SV=1	sp|Q5SRE5|NU188_HUMAN	196,047.60	100.00%	2	3	3	0.00230%	1.72%
Tumor tissue	Nucleoporin Nup37 (Fragment) GN=NUP37 PE=1 SV=8	NUP37_HUMAN	36,707.20	100.00%	3	3	3	0.00230%	21.60%
Tumor tissue	Nucleoporin p58/p45 (Fragment) GN=NUP58 PE=1 SV=1	Q5JRG1_HUMAN	53,152.90	100.00%	3	3	3	0.00230%	9.80%
Tumor tissue	Nucleoprotein TPR GN=TPR PE=1 SV=3	sp|P12270|TPR_HUMAN	267,289.30	100.00%	31	35	35	0.02680%	19.50%
Tumor tissue	Nucleoredoxin GN=NXN PE=1 SV=2	sp|Q6DKJ4|NXN_HUMAN	48,393.60	100.00%	4	5	6	0.00459%	15.40%
Pap test	Nucleoside diphosphate kinase GN=NME1-NME2 PE=1 SV=1	sp|P22392-2|NDKB_HUMAN	32,642.20	100.00%	3	3	10	0.01560%	26.40%
Swab	Nucleoside diphosphate kinase GN=NME1-NME2 PE=1 SV=1	sp|P22392-2|NDKB_HUMAN	32,642.20	100.00%	2	2	25	0.01770%	22.60%
Tumor tissue	Nucleoside diphosphate kinase GN=NME1-NME2 PE=1 SV=1	sp|P22392-2|NDKB_HUMAN	32,642.20	100.00%	5	9	38	0.02910%	45.50%
Tumor tissue	Nucleoside diphosphate kinase 3 GN=NME3 PE=1 SV=2	NDK3_HUMAN	19,015.20	100.00%	2	2	2	0.00153%	17.20%
Tumor tissue	Nucleosome assembly protein 1-like 1 GN=NAP1L1 PE=1 SV=1	sp|P55209|NP1L1_HUMAN	31,021.30	100.00%	1	1	19	0.01450%	23.00%
Tumor tissue	Nucleus accumbens-associated protein 1 GN=NACC1 PE=1 SV=1	NACC1_HUMAN	57,257.40	100.00%	3	3	3	0.00230%	5.12%
Tumor tissue	NudC domain-containing protein 3 GN=NUDCD3 PE=1 SV=3	NUDC3_HUMAN	40,821.60	100.00%	3	3	3	0.00230%	9.14%
Swab	Obg-like ATPase 1 GN=OLA1 PE=1 SV=1	sp|Q9NTK5|OLA1_HUMAN	46,940.00	100.00%	2	2	3	0.00213%	6.01%
Tumor tissue	Obg-like ATPase 1 GN=OLA1 PE=1 SV=1	sp|Q9NTK5|OLA1_HUMAN	46,940.00	100.00%	5	7	12	0.00919%	14.90%
Tumor tissue	OCIA domain-containing protein 1 GN=OCIAD1 PE=1 SV=1	sp|Q9NX40|OCAD1_HUMAN	27,626.80	100.00%	3	6	7	0.00536%	22.90%
Tumor tissue	OCIA domain-containing protein 2 GN=OCIAD2 PE=1 SV=1	sp|Q56VL3|OCAD2_HUMAN	16,953.90	100.00%	2	2	7	0.00536%	14.30%
Pap test	Olfactomedin-4 GN=OLFM4 PE=1 SV=1	OLFM4_HUMAN	57,281.00	100.00%	11	18	39	0.06090%	30.00%
Swab	Olfactomedin-4 GN=OLFM4 PE=1 SV=1	OLFM4_HUMAN	57,281.00	100.00%	7	9	17	0.01200%	19.40%

Biological sample	Protein name	Protein accession numbers	Protein molecular weight (Da)	Protein identification probability	Exclusive unique peptide count	Exclusive unique spectrum count	Total spectrum count	Percentage of total spectra	Percentage sequence coverage
Tumor tissue	Olfactomedin-like protein 3 GN=OLFML3 PE=2 SV=1	sp|Q9NRN5|OLFL3_HUMAN	46,011.20	100.00%	3	3	3	0.00230%	7.14%
Pap test	Omega-amidase NIT2 GN=NIT2 PE=1 SV=1	NIT2_HUMAN	30,608.80	99.70%	1	1	1	0.00156%	3.99%
Swab	Omega-amidase NIT2 GN=NIT2 PE=1 SV=1	NIT2_HUMAN	30,608.80	100.00%	6	8	9	0.00638%	25.40%
Tumor tissue	Omega-amidase NIT2 GN=NIT2 PE=1 SV=1	NIT2_HUMAN	30,608.80	100.00%	5	5	6	0.00459%	27.20%
Tumor tissue	Origin recognition complex subunit 5 GN=ORC5 PE=1 SV=1	sp|O43913|ORC5_HUMAN	50,285.10	100.00%	2	2	2	0.00153%	5.98%
Tumor tissue	Ornithine aminotransferase, mitochondrial GN=OAT PE=1 SV=1	sp|P04181|OAT_HUMAN	48,536.20	100.00%	9	9	10	0.00766%	26.90%
Pap test	Osteoclast-stimulating factor 1 GN=OSTF1 PE=1 SV=2	OSTF1_HUMAN	23,787.70	100.00%	2	2	2	0.00312%	11.20%
Swab	Osteoclast-stimulating factor 1 GN=OSTF1 PE=1 SV=2	OSTF1_HUMAN	23,787.70	100.00%	2	2	2	0.00142%	15.40%
Tumor tissue	Osteoclast-stimulating factor 1 GN=OSTF1 PE=1 SV=2	OSTF1_HUMAN	23,787.70	100.00%	4	5	5	0.00383%	26.60%
Tumor tissue	OTU domain containing 6B, isoform CRA_b GN=OTUD6B PE=1 SV=1	sp|Q8N6M0|OTU6B_HUMAN	37,326.40	100.00%	2	2	2	0.00153%	8.36%
Swab	Oxygen-dependent coproporphyrinogen-III oxidase, mitochondrial GN=CPOX PE=1 SV=3	sp|P36551|HEM6_HUMAN	50,152.00	99.40%	1	1	1	0.00071%	3.74%
Tumor tissue	Oxygen-dependent coproporphyrinogen-III oxidase, mitochondrial GN=CPOX PE=1 SV=3	sp|P36551|HEM6_HUMAN	50,152.00	100.00%	5	7	7	0.00536%	13.70%
Tumor tissue	Oxysterol-binding protein (Fragment) GN=OSBPL10 PE=1 SV=1	sp|Q9BXB5|OSB10_HUMAN	60,347.10	100.00%	2	2	3	0.00230%	6.32%
Tumor tissue	Oxysterol-binding protein 1 GN=OSBP PE=1 SV=1	OSBP1_HUMAN	89,422.40	100.00%	4	6	7	0.00536%	9.05%
Tumor tissue	Oxysterol-binding protein-related protein 11 GN=OSBPL11 PE=1 SV=2	OSB11_HUMAN	83,643.30	100.00%	2	3	4	0.00306%	4.55%
Tumor tissue	Paired amphipathic helix protein Sin3a GN=SIN3A PE=1 SV=2	SIN3A_HUMAN	145,176.40	100.00%	5	7	7	0.00536%	6.05%
Tumor tissue	Palmdelphin GN=PALMD PE=1 SV=1	sp|Q9NP74|PALMD_HUMAN	62,653.60	100.00%	2	2	2	0.00153%	6.53%

Biological sample	Protein name	Protein accession numbers	Protein molecular weight (Da)	Protein identification probability	Exclusive unique peptide count	Exclusive unique spectrum count	Total spectrum count	Percentage of total spectra	Percentage sequence coverage
Pap test	Palmitoyl-protein thioesterase 1 GN=PPT1 PE=1 SV=1	sp|P50897|PPT1_HUMAN	34,194.00	100.00%	2	2	2	0.00312%	10.50%
Swab	Palmitoyl-protein thioesterase 1 GN=PPT1 PE=1 SV=1	sp|P50897|PPT1_HUMAN	34,194.00	99.90%	1	1	2	0.00142%	4.90%
Tumor tissue	Palmitoyl-protein thioesterase 1 GN=PPT1 PE=1 SV=1	sp|P50897|PPT1_HUMAN	34,194.00	100.00%	5	8	10	0.00766%	20.90%
Tumor tissue	Palmitoyltransferase ZDHHC13 GN=ZDHHC13 PE=1 SV=3	sp|Q8IUH4|ZDH13_HUMAN	70,863.10	100.00%	2	2	2	0.00153%	4.98%
Tumor tissue	Pantothenate kinase 4 GN=PANK4 PE=1 SV=1	PANK4_HUMAN	85,993.30	100.00%	3	3	3	0.00230%	4.53%
Tumor tissue	Parafibromin GN=CDC73 PE=1 SV=1	CDC73_HUMAN	60,577.50	100.00%	3	3	3	0.00230%	5.84%
Tumor tissue	Paralemmin-1 GN=PALM PE=1 SV=2	sp|O75781|PALM_HUMAN	42,075.50	100.00%	3	3	3	0.00230%	11.10%
Tumor tissue	Partitioning defective 3 homolog GN=PARD3 PE=1 SV=1	sp|Q8TEW0-5|PARD3_HUMAN	144,492.70	100.00%	1	1	4	0.00306%	5.63%
Tumor tissue	PC4 and SFRS1-interacting protein GN=PSIP1 PE=1 SV=1	sp|O75475|PSIP1_HUMAN	60,103.90	100.00%	2	3	16	0.01220%	24.00%
Pap test	PDZ and LIM domain protein 1 GN=PDLIM1 PE=1 SV=4	PDLI1_HUMAN	36,071.30	99.80%	1	1	1	0.00156%	3.04%
Swab	PDZ and LIM domain protein 1 GN=PDLIM1 PE=1 SV=4	PDLI1_HUMAN	36,071.30	100.00%	4	5	7	0.00496%	19.80%
Tumor tissue	PDZ and LIM domain protein 1 GN=PDLIM1 PE=1 SV=4	PDLI1_HUMAN	36,071.30	100.00%	8	13	17	0.01300%	36.20%
Tumor tissue	PDZ and LIM domain protein 4 GN=PDLIM4 PE=1 SV=2	sp|P50479|PDLI4_HUMAN	35,398.20	100.00%	6	9	9	0.00689%	23.60%
Tumor tissue	PDZ and LIM domain protein 5 GN=PDLIM5 PE=1 SV=5	sp|Q96HC4|PDLI5_HUMAN	63,944.00	100.00%	4	6	16	0.01220%	21.80%
Tumor tissue	PDZ domain-containing protein GIPC1 GN=GIPC1 PE=1 SV=2	sp|O14908|GIPC1_HUMAN	36,050.60	100.00%	1	1	5	0.00383%	20.10%
Tumor tissue	Peflin GN=PEF1 PE=1 SV=1	PEF1_HUMAN	30,381.30	100.00%	4	4	4	0.00306%	14.80%
Tumor tissue	Pentatricopeptide repeat domain-containing protein 3, mitochondrial GN=PTCD3 PE=1 SV=3	sp|Q96EY7|PTCD3_HUMAN	78,552.60	100.00%	1	1	2	0.00153%	3.63%

Biological sample	Protein name	Protein accession numbers	Protein molecular weight (Da)	Protein identification probability	Exclusive unique peptide count	Exclusive unique spectrum count	Total spectrum count	Percentage of total spectra	Percentage sequence coverage
Pap test	Pentraxin-related protein PTX3 GN=PTX3 PE=1 SV=3	PTX3_HUMAN	41,975.90	100.00%	4	4	5	0.00780%	13.40%
Swab	Pentraxin-related protein PTX3 GN=PTX3 PE=1 SV=3	PTX3_HUMAN	41,975.90	100.00%	4	4	6	0.00425%	12.10%
Tumor tissue	Pentraxin-related protein PTX3 GN=PTX3 PE=1 SV=3	PTX3_HUMAN	41,975.90	100.00%	2	2	2	0.00153%	6.04%
Pap test	Peptidoglycan recognition protein 1 GN=PGLYRP1 PE=1 SV=1	PGRP1_HUMAN	21,730.60	100.00%	3	5	13	0.02030%	27.60%
Swab	Peptidoglycan recognition protein 1 GN=PGLYRP1 PE=1 SV=1	PGRP1_HUMAN	21,730.60	100.00%	3	5	15	0.01060%	27.60%
Pap test	Peptidyl-prolyl cis-trans isomerase A GN=PPIA PE=1 SV=2	sp|P62937|PPIA_HUMAN	18,012.90	100.00%	5	7	29	0.04530%	49.10%
Swab	Peptidyl-prolyl cis-trans isomerase A GN=PPIA PE=1 SV=2	sp|P62937|PPIA_HUMAN	18,012.90	100.00%	5	9	49	0.03470%	37.60%
Tumor tissue	Peptidyl-prolyl cis-trans isomerase A GN=PPIA PE=1 SV=2	sp|P62937|PPIA_HUMAN	18,012.90	100.00%	7	12	33	0.02530%	32.10%
Pap test	Peptidyl-prolyl cis-trans isomerase B GN=PPIB PE=1 SV=2	PPIB_HUMAN	23,743.20	100.00%	4	4	7	0.01090%	20.80%
Tumor tissue	Peptidyl-prolyl cis-trans isomerase B GN=PPIB PE=1 SV=2	PPIB_HUMAN	23,743.20	100.00%	9	20	40	0.03060%	40.70%
Tumor tissue	Peptidyl-prolyl cis-trans isomerase C GN=PPIC PE=1 SV=1	PPIC_HUMAN	22,763.50	100.00%	2	2	4	0.00306%	9.91%
Tumor tissue	Peptidyl-prolyl cis-trans isomerase D GN=PPID PE=1 SV=3	PPID_HUMAN	40,764.90	100.00%	5	5	5	0.00383%	17.30%
Tumor tissue	Peptidyl-prolyl cis-trans isomerase F, mitochondrial GN=PPIF PE=1 SV=1	sp|P30405|PPIF_HUMAN	22,040.40	100.00%	2	2	5	0.00383%	26.10%
Tumor tissue	Peptidyl-prolyl cis-trans isomerase FKBP10 GN=FKBP10 PE=1 SV=1	sp|Q96AY3|FKB10_HUMAN	64,245.90	100.00%	4	5	14	0.01070%	18.20%
Pap test	Peptidyl-prolyl cis-trans isomerase FKBP2 GN=FKBP2 PE=1 SV=2	FKBP2_HUMAN	15,649.60	99.20%	1	1	2	0.00312%	8.45%
Swab	Peptidyl-prolyl cis-trans isomerase FKBP2 GN=FKBP2 PE=1 SV=2	FKBP2_HUMAN	15,649.60	99.60%	1	1	2	0.00142%	8.45%
Tumor tissue	Peptidyl-prolyl cis-trans isomerase FKBP2 GN=FKBP2 PE=1 SV=2	FKBP2_HUMAN	15,649.60	100.00%	3	4	4	0.00306%	17.60%

Biological sample	Protein name	Protein accession numbers	Protein molecular weight (Da)	Protein identification probability	Exclusive unique peptide count	Exclusive unique spectrum count	Total spectrum count	Percentage of total spectra	Percentage sequence coverage
Tumor tissue	Peptidyl-prolyl cis-trans isomerase FKBP3 GN=FKBP3 PE=1 SV=1	FKBP3_HUMAN	25,177.40	100.00%	3	7	7	0.00536%	20.50%
Pap test	Peptidyl-prolyl cis-trans isomerase FKBP4 GN=FKBP4 PE=1 SV=3	FKBP4_HUMAN	51,805.90	99.80%	1	1	1	0.00156%	3.70%
Swab	Peptidyl-prolyl cis-trans isomerase FKBP4 GN=FKBP4 PE=1 SV=3	FKBP4_HUMAN	51,805.90	100.00%	1	1	2	0.00142%	3.70%
Tumor tissue	Peptidyl-prolyl cis-trans isomerase FKBP4 GN=FKBP4 PE=1 SV=3	FKBP4_HUMAN	51,805.90	100.00%	14	21	26	0.01990%	41.80%
Tumor tissue	Peptidyl-prolyl cis-trans isomerase FKBP5 GN=FKBP5 PE=1 SV=2	sp|Q13451|FKBP5_HUMAN	51,213.60	100.00%	6	6	6	0.00459%	17.10%
Tumor tissue	Peptidyl-prolyl cis-trans isomerase G (Fragment) GN=PPIG PE=1 SV=1	C9JM79_HUMAN	55,979.70	100.00%	3	3	3	0.00230%	7.39%
Tumor tissue	Peptidyl-prolyl cis-trans isomerase H GN=PPIH PE=1 SV=1	sp|O43447|PPIH_HUMAN	19,208.10	100.00%	5	5	6	0.00459%	39.50%
Tumor tissue	Peptidyl-prolyl cis-trans isomerase-like 1 GN=PPIL1 PE=1 SV=1	PPIL1_HUMAN	18,237.30	100.00%	2	3	3	0.00230%	16.90%
Tumor tissue	Peptidyl-tRNA hydrolase 2, mitochondrial GN=PTRH2 PE=1 SV=1	PTH2_HUMAN	19,324.90	100.00%	4	6	9	0.00689%	35.00%
Tumor tissue	Pericentriolar material 1 protein GN=PCM1 PE=1 SV=1	sp|Q15154|PCM1_HUMAN	227,729.80	100.00%	2	3	3	0.00230%	1.29%
Tumor tissue	Perilipin-2 GN=PLIN2 PE=1 SV=2	PLIN2_HUMAN	48,075.00	100.00%	3	3	3	0.00230%	10.50%
Pap test	Perilipin-3 (Fragment) GN=PLIN3 PE=1 SV=1	K7ERZ3_HUMAN	31,982.60	99.70%	1	1	6	0.00936%	34.60%
Swab	Perilipin-3 (Fragment) GN=PLIN3 PE=1 SV=1	K7ER39_HUMAN	17,609.30	98.60%	1	1	6	0.00425%	39.90%
Tumor tissue	Periodic tryptophan protein 1 homolog GN=PWP1 PE=1 SV=1	B4DJV5_HUMAN	49,039.50	100.00%	2	2	2	0.00153%	8.20%
Tumor tissue	Periostin GN=POSTN PE=1 SV=1	B1ALD9_HUMAN	90,147.40	100.00%	1	1	125	0.09570%	37.90%
Tumor tissue	Peripheral plasma membrane protein CASK GN=CASK PE=1 SV=1	Q5JS72_HUMAN	59,365.30	100.00%	1	1	7	0.00536%	14.90%
Pap test	Periplakin GN=PPL PE=1 SV=1	PEPL_HUMAN	204,490.00	100.00%	52	62	87	0.13600%	33.10%
Swab	Periplakin GN=PPL PE=1 SV=1	PEPL_HUMAN	204,490.00	100.00%	8	8	8	0.00567%	6.61%

Biological sample	Protein name	Protein accession numbers	Protein molecular weight (Da)	Protein identification probability	Exclusive unique peptide count	Exclusive unique spectrum count	Total spectrum count	Percentage of total spectra	Percentage sequence coverage
Tumor tissue	Periplakin GN=PPL PE=1 SV=1	PEPL_HUMAN	204,490.00	100.00%	28	30	33	0.02530%	21.40%
Tumor tissue	Peroxidasin homolog GN=PXDN PE=1 SV=2	sp|Q92626|PXDN_HUMAN	165,275.70	100.00%	17	20	21	0.01610%	16.20%
Pap test	Peroxiredoxin-1 GN=PRDX1 PE=1 SV=1	PRDX1_HUMAN	22,110.90	100.00%	9	12	43	0.06710%	65.30%
Swab	Peroxiredoxin-1 GN=PRDX1 PE=1 SV=1	PRDX1_HUMAN	22,110.90	100.00%	8	13	33	0.02340%	53.30%
Tumor tissue	Peroxiredoxin-1 GN=PRDX1 PE=1 SV=1	PRDX1_HUMAN	22,110.90	100.00%	9	13	37	0.02830%	48.70%
Pap test	Peroxiredoxin-2 GN=PRDX2 PE=1 SV=5	sp|P32119|PRDX2_HUMAN	21,892.40	100.00%	4	5	36	0.05620%	24.70%
Swab	Peroxiredoxin-2 GN=PRDX2 PE=1 SV=5	sp|P32119|PRDX2_HUMAN	21,892.40	100.00%	4	4	15	0.01060%	46.00%
Tumor tissue	Peroxiredoxin-2 GN=PRDX2 PE=1 SV=5	sp|P32119|PRDX2_HUMAN	21,892.40	100.00%	5	8	35	0.02680%	33.30%
Pap test	Peroxiredoxin-4 GN=PRDX4 PE=1 SV=1	PRDX4_HUMAN	30,541.10	100.00%	1	1	5	0.00780%	11.40%
Tumor tissue	Peroxiredoxin-4 GN=PRDX4 PE=1 SV=1	PRDX4_HUMAN	30,541.10	100.00%	6	11	31	0.02370%	34.70%
Pap test	Peroxiredoxin-5, mitochondrial GN=PRDX5 PE=1 SV=4	sp|P30044|PRDX5_HUMAN	22,086.40	100.00%	8	9	16	0.02500%	55.10%
Swab	Peroxiredoxin-5, mitochondrial GN=PRDX5 PE=1 SV=4	sp|P30044|PRDX5_HUMAN	22,086.40	100.00%	8	10	28	0.01980%	44.90%
Tumor tissue	Peroxiredoxin-5, mitochondrial GN=PRDX5 PE=1 SV=4	sp|P30044|PRDX5_HUMAN	22,086.40	100.00%	6	9	23	0.01760%	37.40%
Pap test	Peroxiredoxin-6 GN=PRDX6 PE=1 SV=3	PRDX6_HUMAN	25,036.10	100.00%	9	14	28	0.04370%	41.50%
Swab	Peroxiredoxin-6 GN=PRDX6 PE=1 SV=3	PRDX6_HUMAN	25,036.10	100.00%	9	16	41	0.02910%	40.20%
Tumor tissue	Peroxiredoxin-6 GN=PRDX6 PE=1 SV=3	PRDX6_HUMAN	25,036.10	100.00%	13	21	62	0.04750%	53.10%
Tumor tissue	Peroxisomal acyl-coenzyme A oxidase 3 GN=ACOX3 PE=1 SV=2	sp|O15254|ACOX3_HUMAN	77,631.20	100.00%	3	3	3	0.00230%	5.86%

Biological sample	Protein name	Protein accession numbers	Protein molecular weight (Da)	Protein identification probability	Exclusive unique peptide count	Exclusive unique spectrum count	Total spectrum count	Percentage of total spectra	Percentage sequence coverage
Tumor tissue	Peroxisomal multifunctional enzyme type 2 GN=HSD17B4 PE=1 SV=3	sp|P51659|DHB4_HUMAN	79,688.50	100.00%	6	7	23	0.01760%	28.90%
Pap test	Persulfide dioxygenase ETHE1, mitochondrial GN=ETHE1 PE=1 SV=2	ETHE1_HUMAN	27,873.20	100.00%	2	2	3	0.00468%	9.45%
Swab	Persulfide dioxygenase ETHE1, mitochondrial GN=ETHE1 PE=1 SV=2	ETHE1_HUMAN	27,873.20	100.00%	2	2	4	0.00283%	10.20%
Tumor tissue	Persulfide dioxygenase ETHE1, mitochondrial GN=ETHE1 PE=1 SV=2	ETHE1_HUMAN	27,873.20	100.00%	4	7	10	0.00766%	24.40%
Tumor tissue	Pescadillo homolog GN=PES1 PE=1 SV=1	sp|O00541|PESC_HUMAN	66,079.70	100.00%	3	3	3	0.00230%	4.90%
Tumor tissue	PEST proteolytic signal-containing nuclear protein GN=PCNP PE=1 SV=2	sp|Q8WW12|PCNP_HUMAN	18,925.40	100.00%	3	4	4	0.00306%	21.90%
Tumor tissue	PHD finger protein 14 GN=PHF14 PE=1 SV=1	A0A0U1RRH6_HUMAN	106,988.00	100.00%	1	1	2	0.00153%	2.32%
Tumor tissue	Phenylalanine--tRNA ligase alpha subunit GN=FARSA PE=1 SV=1	sp|Q9Y285|SYFA_HUMAN	62,396.20	100.00%	8	11	14	0.01070%	25.20%
Tumor tissue	Phenylalanine--tRNA ligase beta subunit GN=FARSB PE=1 SV=3	sp|Q9NSD9|SYFB_HUMAN	66,118.50	100.00%	7	9	9	0.00689%	12.90%
Tumor tissue	Phosphatidate cytidylyltransferase 2 GN=CDS2 PE=1 SV=1	CDS2_HUMAN	51,419.70	100.00%	2	2	2	0.00153%	8.54%
Pap test	Phosphatidylethanolamine-binding protein 1 GN=PEBP1 PE=1 SV=3	PEBP1_HUMAN	21,056.90	100.00%	8	10	15	0.02340%	69.00%
Swab	Phosphatidylethanolamine-binding protein 1 GN=PEBP1 PE=1 SV=3	PEBP1_HUMAN	21,056.90	100.00%	7	10	20	0.01420%	58.30%
Tumor tissue	Phosphatidylethanolamine-binding protein 1 GN=PEBP1 PE=1 SV=3	PEBP1_HUMAN	21,056.90	100.00%	6	11	20	0.01530%	40.60%
Tumor tissue	Phosphatidylinositol 3,4,5-trisphosphate 5-phosphatase 2 GN=INPPL1 PE=1 SV=2	sp|O15357|SHIP2_HUMAN	138,602.30	100.00%	4	4	4	0.00306%	4.93%
Tumor tissue	Phosphatidylinositol 3-kinase GN=PIK3C3 PE=1 SV=2	PK3C3_HUMAN	94,403.40	100.00%	3	3	3	0.00230%	5.95%

Biological sample	Protein name	Protein accession numbers	Protein molecular weight (Da)	Protein identification probability	Exclusive unique peptide count	Exclusive unique spectrum count	Total spectrum count	Percentage of total spectra	Percentage sequence coverage
Tumor tissue	Phosphatidylinositol 4-kinase alpha GN=PI4KA PE=1 SV=4	sp|P42356|PI4KA_HUMAN	236,838.30	100.00%	6	6	8	0.00612%	3.90%
Tumor tissue	Phosphatidylinositol 4-kinase type 2-alpha GN=PI4K2A PE=1 SV=1	P4K2A_HUMAN	54,023.00	99.90%	1	1	3	0.00230%	4.38%
Tumor tissue	Phosphatidylinositol 4-phosphate 3-kinase C2 domain-containing subunit alpha GN=PIK3C2A PE=1 SV=2	P3C2A_HUMAN	190,685.90	100.00%	3	3	3	0.00230%	1.78%
Tumor tissue	Phosphatidylinositol 5-phosphate 4-kinase type-2 alpha GN=PIP4K2A PE=1 SV=2	sp|P48426|PI42A_HUMAN	46,225.80	100.00%	2	2	5	0.00383%	17.50%
Tumor tissue	Phosphatidylinositol 5-phosphate 4-kinase type-2 beta GN=PIP4K2B PE=1 SV=1	sp|P78356|PI42B_HUMAN	47,378.50	99.90%	1	1	3	0.00230%	9.86%
Pap test	Phosphatidylinositol transfer protein alpha isoform GN=PITPNA PE=1 SV=2	PIPNA_HUMAN	31,806.30	100.00%	2	2	4	0.00624%	18.50%
Swab	Phosphatidylinositol transfer protein alpha isoform GN=PITPNA PE=1 SV=2	PIPNA_HUMAN	31,806.30	100.00%	2	2	2	0.00142%	11.10%
Tumor tissue	Phosphatidylinositol transfer protein alpha isoform GN=PITPNA PE=1 SV=2	PIPNA_HUMAN	31,806.30	100.00%	7	7	8	0.00612%	34.80%
Tumor tissue	Phosphatidylinositol-binding clathrin assembly protein (Fragment) GN=PICALM PE=1 SV=1	H0YD48_HUMAN	22,372.00	99.50%	1	1	2	0.00153%	14.80%
Swab	Phosphoenolpyruvate carboxykinase [GTP], mitochondrial GN=PCK2 PE=1 SV=3	sp|Q16822|PCKGM_HUMAN	70,730.90	99.60%	1	1	1	0.00071%	1.72%
Tumor tissue	Phosphoenolpyruvate carboxykinase [GTP], mitochondrial GN=PCK2 PE=1 SV=3	sp|Q16822|PCKGM_HUMAN	70,730.90	100.00%	9	10	12	0.00919%	19.70%
Tumor tissue	Phosphofurin acidic cluster sorting protein 2 GN=PACS2 PE=1 SV=1	sp|Q86VP3|PACS2_HUMAN	94,428.40	100.00%	2	2	2	0.00153%	2.56%
Pap test	Phosphoglucomutase-1 GN=PGM1 PE=1 SV=3	sp|P36871|PGM1_HUMAN	61,451.10	100.00%	2	2	11	0.01720%	21.00%

Biological sample	Protein name	Protein accession numbers	Protein molecular weight (Da)	Protein identification probability	Exclusive unique peptide count	Exclusive unique spectrum count	Total spectrum count	Percentage of total spectra	Percentage sequence coverage
Swab	Phosphoglucomutase-1 GN=PGM1 PE=1 SV=3	sp|P36871|PGM1_HUMAN	61,451.10	100.00%	3	3	22	0.01560%	27.60%
Tumor tissue	Phosphoglucomutase-1 GN=PGM1 PE=1 SV=3	sp|P36871|PGM1_HUMAN	61,451.10	100.00%	4	4	46	0.03520%	41.50%
Pap test	Phosphoglucomutase-2 GN=PGM2 PE=1 SV=4	sp|Q96G03|PGM2_HUMAN	68,285.70	100.00%	2	2	8	0.01250%	10.10%
Swab	Phosphoglucomutase-2 GN=PGM2 PE=1 SV=4	sp|Q96G03|PGM2_HUMAN	68,285.70	100.00%	6	6	11	0.00780%	17.50%
Tumor tissue	Phosphoglucomutase-2 GN=PGM2 PE=1 SV=4	sp|Q96G03|PGM2_HUMAN	68,285.70	100.00%	6	7	16	0.01220%	26.60%
Tumor tissue	Phosphoglucomutase-like protein 5 GN=PGM5 PE=1 SV=2	sp|Q15124|PGM5_HUMAN	62,227.10	100.00%	13	18	24	0.01840%	28.00%
Pap test	Phosphoglycerate kinase 1 GN=PGK1 PE=1 SV=3	sp|P00558|PGK1_HUMAN	44,615.30	100.00%	11	17	32	0.04990%	34.50%
Swab	Phosphoglycerate kinase 1 GN=PGK1 PE=1 SV=3	sp|P00558|PGK1_HUMAN	44,615.30	100.00%	16	28	81	0.05740%	48.70%
Tumor tissue	Phosphoglycerate kinase 1 GN=PGK1 PE=1 SV=3	sp|P00558|PGK1_HUMAN	44,615.30	100.00%	22	54	129	0.09880%	59.50%
Pap test	Phosphoglycerate mutase 1 GN=PGAM1 PE=1 SV=2	PGAM1_HUMAN	28,804.80	100.00%	6	11	69	0.10800%	53.50%
Swab	Phosphoglycerate mutase 1 GN=PGAM1 PE=1 SV=2	PGAM1_HUMAN	28,804.80	100.00%	6	15	78	0.05530%	48.00%
Tumor tissue	Phosphoglycerate mutase 1 GN=PGAM1 PE=1 SV=2	PGAM1_HUMAN	28,804.80	100.00%	5	15	42	0.03220%	47.60%
Tumor tissue	Phosphoinositide 3-kinase regulatory subunit 4 GN=PIK3R4 PE=1 SV=3	PI3R4_HUMAN	153,107.10	100.00%	1	1	2	0.00153%	1.91%
Tumor tissue	Phosphoinositide phospholipase C GN=PLCG2 PE=1 SV=1	PLCG2_HUMAN	146,064.30	100.00%	3	3	3	0.00230%	2.40%
Swab	Phospholipase A-2-activating protein GN=PLAA PE=1 SV=2	PLAP_HUMAN	87,159.30	99.90%	1	1	1	0.00071%	1.13%
Tumor tissue	Phospholipase A-2-activating protein GN=PLAA PE=1 SV=2	PLAP_HUMAN	87,159.30	100.00%	2	2	2	0.00153%	3.40%
Pap test	Phospholipase B-like 1 GN=PLBD1 PE=1 SV=2	PLBL1_HUMAN	63,258.10	100.00%	5	6	8	0.01250%	11.00%

Biological sample	Protein name	Protein accession numbers	Protein molecular weight (Da)	Protein identification probability	Exclusive unique peptide count	Exclusive unique spectrum count	Total spectrum count	Percentage of total spectra	Percentage sequence coverage
Swab	Phospholipase B-like 1 GN=PLBD1 PE=1 SV=2	PLBL1_HUMAN	63,258.10	100.00%	4	4	7	0.00496%	11.60%
Tumor tissue	Phospholipase B-like 1 GN=PLBD1 PE=1 SV=2	PLBL1_HUMAN	63,258.10	100.00%	2	2	2	0.00153%	4.34%
Pap test	Phospholipase D3 GN=PLD3 PE=1 SV=1	PLD3_HUMAN	54,706.00	99.10%	1	1	1	0.00156%	2.45%
Swab	Phospholipase D3 GN=PLD3 PE=1 SV=1	PLD3_HUMAN	54,706.00	100.00%	3	3	3	0.00213%	6.33%
Tumor tissue	Phospholipase D3 GN=PLD3 PE=1 SV=1	PLD3_HUMAN	54,706.00	100.00%	5	5	6	0.00459%	13.50%
Pap test	Phospholipid transfer protein GN=PLTP PE=1 SV=1	sp|P55058|PLTP_HUMAN	54,741.20	100.00%	2	3	4	0.00624%	5.48%
Swab	Phospholipid transfer protein GN=PLTP PE=1 SV=1	sp|P55058|PLTP_HUMAN	54,741.20	100.00%	4	5	8	0.00567%	10.30%
Swab	Phospholysine phosphohistidine inorganic pyrophosphate phosphatase GN=LHPP PE=1 SV=2	sp|Q9H008|LHPP_HUMAN	29,165.40	99.80%	2	2	2	0.00142%	7.78%
Tumor tissue	Phospholysine phosphohistidine inorganic pyrophosphate phosphatase GN=LHPP PE=1 SV=2	sp|Q9H008|LHPP_HUMAN	29,165.40	99.90%	1	1	1	0.00077%	3.33%
Swab	Phosphomevalonate kinase GN=PMVK PE=1 SV=3	PMVK_HUMAN	21,995.00	99.90%	1	1	1	0.00071%	6.77%
Tumor tissue	Phosphomevalonate kinase GN=PMVK PE=1 SV=3	PMVK_HUMAN	21,995.00	100.00%	6	6	6	0.00459%	28.10%
Tumor tissue	Phosphopantothenate--cysteine ligase GN=PPCS PE=1 SV=2	sp|Q9HAB8|PPCS_HUMAN	34,006.00	100.00%	5	5	8	0.00612%	25.40%
Tumor tissue	Phosphoribosylformylglycinamidine synthase GN=PFAS PE=1 SV=4	PUR4_HUMAN	144,733.80	100.00%	6	6	6	0.00459%	7.92%
Tumor tissue	Phosphoserine aminotransferase GN=PSAT1 PE=1 SV=2	sp|Q9Y617|SERC_HUMAN	40,423.30	100.00%	7	8	9	0.00689%	20.80%
Tumor tissue	Phosphoserine phosphatase (Fragment) GN=PSPH PE=1 SV=1	SERB_HUMAN	20,745.20	100.00%	2	2	2	0.00153%	11.80%
Swab	Phosphotriesterase-related protein GN=PTER PE=1 SV=1	sp|Q96BW5|PTER_HUMAN	39,018.90	99.60%	1	1	2	0.00142%	2.87%
Tumor tissue	Phosphotriesterase-related protein GN=PTER PE=1 SV=1	sp|Q96BW5|PTER_HUMAN	39,018.90	100.00%	3	3	3	0.00230%	10.90%

Biological sample	Protein name	Protein accession numbers	Protein molecular weight (Da)	Protein identification probability	Exclusive unique peptide count	Exclusive unique spectrum count	Total spectrum count	Percentage of total spectra	Percentage sequence coverage
Tumor tissue	Phostensin GN=PPP1R18 PE=1 SV=1	A0A0G2JHC2_HUMAN	68,042.00	100.00%	7	9	9	0.00689%	19.40%
Tumor tissue	Phytanoyl-CoA dioxygenase domain containing 1, isoform CRA_c GN=PHYHD1 PE=1 SV=1	G5E9M0_HUMAN	17,950.40	100.00%	2	2	2	0.00153%	15.60%
Pap test	Pigment epithelium-derived factor GN=SERPINF1 PE=1 SV=4	PEDF_HUMAN	46,314.30	100.00%	8	8	9	0.01400%	23.40%
Swab	Pigment epithelium-derived factor GN=SERPINF1 PE=1 SV=4	PEDF_HUMAN	46,314.30	100.00%	9	11	22	0.01560%	25.80%
Tumor tissue	Pigment epithelium-derived factor GN=SERPINF1 PE=1 SV=4	PEDF_HUMAN	46,314.30	100.00%	7	9	15	0.01150%	21.30%
Tumor tissue	Pinin GN=PNN PE=1 SV=4	sp|Q9H307|PININ_HUMAN	81,613.20	100.00%	6	7	10	0.00766%	12.70%
Swab	Plasma kallikrein (Fragment) GN=KLKB1 PE=1 SV=1	KLKB1_HUMAN	71,369.70	100.00%	1	1	4	0.00283%	5.39%
Tumor tissue	Plasma membrane calcium-transporting ATPase 4 GN=ATP2B4 PE=1 SV=2	sp|P23634|AT2B4_HUMAN	137,923.70	100.00%	7	8	16	0.01220%	14.80%
Pap test	Plasma serine protease inhibitor GN=SERPINA5 PE=1 SV=3	IPSP_HUMAN	45,675.80	100.00%	4	4	4	0.00624%	12.80%
Swab	Plasma serine protease inhibitor GN=SERPINA5 PE=1 SV=3	IPSP_HUMAN	45,675.80	100.00%	5	5	6	0.00425%	16.00%
Tumor tissue	Plasma serine protease inhibitor GN=SERPINA5 PE=1 SV=3	IPSP_HUMAN	45,675.80	100.00%	3	3	3	0.00230%	9.11%
Pap test	Plasminogen GN=PLG PE=1 SV=2	PLMN_HUMAN	90,567.40	100.00%	19	22	27	0.04210%	30.10%
Swab	Plasminogen GN=PLG PE=1 SV=2	PLMN_HUMAN	90,567.40	100.00%	18	27	47	0.03330%	32.50%
Tumor tissue	Plasminogen GN=PLG PE=1 SV=2	PLMN_HUMAN	90,567.40	100.00%	4	4	4	0.00306%	6.54%
Pap test	Plasminogen activator inhibitor 1 RNA-binding protein GN=SERBP1 PE=1 SV=2	sp|Q8NC51|PAIRB_HUMAN	44,965.80	99.60%	1	1	1	0.00156%	4.41%
Tumor tissue	Plasminogen activator inhibitor 1 RNA-binding protein GN=SERBP1 PE=1 SV=2	sp|Q8NC51|PAIRB_HUMAN	44,965.80	100.00%	9	13	13	0.00995%	29.40%
Pap test	Plasminogen activator inhibitor 2 GN=SERPINB2 PE=1 SV=2	PAI2_HUMAN	46,597.60	100.00%	7	8	8	0.01250%	25.30%

Biological sample	Protein name	Protein accession numbers	Protein molecular weight (Da)	Protein identification probability	Exclusive unique peptide count	Exclusive unique spectrum count	Total spectrum count	Percentage of total spectra	Percentage sequence coverage
Swab	Plasminogen activator inhibitor 2 GN=SERPINB2 PE=1 SV=2	PAI2_HUMAN	46,597.60	100.00%	5	7	8	0.00567%	15.40%
Swab	Plastin-1 GN=PLS1 PE=1 SV=2	PLSI_HUMAN	70,257.20	100.00%	4	4	22	0.01560%	15.60%
Pap test	Plastin-2 GN=LCP1 PE=1 SV=6	sp|P13796|PLSL_HUMAN	70,291.20	100.00%	19	36	109	0.17000%	49.80%
Swab	Plastin-2 GN=LCP1 PE=1 SV=6	sp|P13796|PLSL_HUMAN	70,291.20	100.00%	24	46	169	0.12000%	56.10%
Tumor tissue	Plastin-2 GN=LCP1 PE=1 SV=6	sp|P13796|PLSL_HUMAN	70,291.20	100.00%	18	30	60	0.04590%	38.40%
Pap test	Plastin-3 GN=PLS3 PE=1 SV=4	sp|P13797|PLST_HUMAN	70,813.90	100.00%	6	6	40	0.06240%	22.50%
Swab	Plastin-3 GN=PLS3 PE=1 SV=4	sp|P13797|PLST_HUMAN	70,813.90	100.00%	7	7	39	0.02760%	29.20%
Tumor tissue	Plastin-3 GN=PLS3 PE=1 SV=4	sp|P13797|PLST_HUMAN	70,813.90	100.00%	12	17	34	0.02600%	32.10%
Tumor tissue	Platelet endothelial cell adhesion molecule GN=PECAM1 PE=1 SV=1	sp|P16284|PECA1_HUMAN	82,536.00	100.00%	5	5	5	0.00383%	9.21%
Swab	Platelet-activating factor acetylhydrolase IB subunit alpha GN=PAFAH1B1 PE=1 SV=2	sp|P43034|LIS1_HUMAN	46,637.40	100.00%	2	2	2	0.00142%	6.59%
Tumor tissue	Platelet-activating factor acetylhydrolase IB subunit alpha GN=PAFAH1B1 PE=1 SV=2	sp|P43034|LIS1_HUMAN	46,637.40	100.00%	8	9	10	0.00766%	29.50%
Pap test	Platelet-activating factor acetylhydrolase IB subunit gamma GN=PAFAH1B3 PE=1 SV=1	PA1B3_HUMAN	25,733.60	99.20%	1	1	1	0.00156%	3.90%
Swab	Platelet-activating factor acetylhydrolase IB subunit gamma GN=PAFAH1B3 PE=1 SV=1	PA1B3_HUMAN	25,733.60	98.30%	1	1	1	0.00071%	3.90%
Tumor tissue	Platelet-activating factor acetylhydrolase IB subunit gamma GN=PAFAH1B3 PE=1 SV=1	PA1B3_HUMAN	25,733.60	100.00%	4	6	8	0.00612%	26.40%
Tumor tissue	Platelet-derived growth factor receptor beta GN=PDGFRB PE=1 SV=1	sp|P09619|PGFRB_HUMAN	123,969.50	100.00%	2	2	3	0.00230%	2.44%
Tumor tissue	Pleckstrin GN=PLEK PE=1 SV=3	PLEK_HUMAN	40,126.40	100.00%	2	3	3	0.00230%	8.29%
Tumor tissue	Pleckstrin homology domain-containing family F member 2 GN=PLEKHF2 PE=1 SV=1	PKHF2_HUMAN	27,798.20	100.00%	1	1	2	0.00153%	12.40%

Biological sample	Protein name	Protein accession numbers	Protein molecular weight (Da)	Protein identification probability	Exclusive unique peptide count	Exclusive unique spectrum count	Total spectrum count	Percentage of total spectra	Percentage sequence coverage
Tumor tissue	Pleckstrin homology domain-containing family O member 2 GN=PLEKHO2 PE=1 SV=1	sp|Q8TD55|PKHO2_HUMAN	53,349.90	100.00%	7	7	8	0.00612%	21.80%
Tumor tissue	Plectin GN=PLEC PE=1 SV=3	sp|Q15149|PLEC_HUMAN	531,783.90	100.00%	1	1	419	0.32100%	44.40%
Tumor tissue	Pleiotropic regulator 1 GN=PLRG1 PE=1 SV=1	sp|O43660|PLRG1_HUMAN	57,193.40	100.00%	6	6	6	0.00459%	16.10%
Tumor tissue	Plexin domain-containing protein 2 GN=PLXDC2 PE=1 SV=1	sp|Q6UX71|PXDC2_HUMAN	59,583.00	100.00%	4	4	4	0.00306%	9.64%
Tumor tissue	Plexin-B2 GN=PLXNB2 PE=1 SV=3	PLXB2_HUMAN	205,126.70	100.00%	13	16	18	0.01380%	10.50%
Swab	Podocan GN=PODN PE=1 SV=2	sp|Q7Z5L7|PODN_HUMAN	68,979.10	98.40%	1	1	1	0.00071%	3.43%
Tumor tissue	Podocan GN=PODN PE=1 SV=2	sp|Q7Z5L7|PODN_HUMAN	68,979.10	100.00%	6	6	7	0.00536%	13.90%
Tumor tissue	Poly [ADP-ribose] polymerase GN=PARP10 PE=1 SV=1	PAR10_HUMAN	109,096.30	100.00%	4	4	4	0.00306%	5.81%
Tumor tissue	Poly [ADP-ribose] polymerase 1 GN=PARP1 PE=1 SV=4	PARP1_HUMAN	113,087.80	100.00%	26	41	51	0.03900%	34.10%
Pap test	Poly [ADP-ribose] polymerase 4 GN=PARP4 PE=1 SV=3	PARP4_HUMAN	192,598.30	99.20%	1	1	1	0.00156%	0.58%
Swab	Poly [ADP-ribose] polymerase 4 GN=PARP4 PE=1 SV=3	PARP4_HUMAN	192,598.30	99.90%	1	1	2	0.00142%	0.58%
Tumor tissue	Poly [ADP-ribose] polymerase 4 GN=PARP4 PE=1 SV=3	PARP4_HUMAN	192,598.30	100.00%	6	6	6	0.00459%	4.70%
Tumor tissue	Poly [ADP-ribose] polymerase 9 GN=PARP9 PE=1 SV=2	sp|Q8IXQ6|PARP9_HUMAN	96,344.30	100.00%	5	6	6	0.00459%	8.08%
Swab	Poly(ADP-ribose) glycohydrolase ARH3 GN=ADPRHL2 PE=1 SV=1	ARHL2_HUMAN	38,947.30	100.00%	3	3	3	0.00213%	10.20%
Tumor tissue	Poly(ADP-ribose) glycohydrolase ARH3 GN=ADPRHL2 PE=1 SV=1	ARHL2_HUMAN	38,947.30	100.00%	7	7	7	0.00536%	27.80%
Pap test	Poly(rC)-binding protein 1 GN=PCBP1 PE=1 SV=2	PCBP1_HUMAN	37,498.20	100.00%	4	4	6	0.00936%	18.30%
Swab	Poly(rC)-binding protein 1 GN=PCBP1 PE=1 SV=2	PCBP1_HUMAN	37,498.20	100.00%	5	5	13	0.00921%	28.10%
Tumor tissue	Poly(rC)-binding protein 1 GN=PCBP1 PE=1 SV=2	PCBP1_HUMAN	37,498.20	100.00%	7	12	41	0.03140%	36.50%

Biological sample	Protein name	Protein accession numbers	Protein molecular weight (Da)	Protein identification probability	Exclusive unique peptide count	Exclusive unique spectrum count	Total spectrum count	Percentage of total spectra	Percentage sequence coverage
Pap test	Poly(rC)-binding protein 2 GN=PCBP2 PE=1 SV=1	sp|Q15366|PCBP2_HUMAN	33,800.60	100.00%	1	1	4	0.00624%	11.80%
Tumor tissue	Poly(rC)-binding protein 2 GN=PCBP2 PE=1 SV=1	sp|Q15366|PCBP2_HUMAN	35,347.30	100.00%	3	6	19	0.01450%	23.40%
Pap test	Polyadenylate-binding protein GN=PABPC4 PE=1 SV=1	B1ANR0_HUMAN	67,971.80	100.00%	1	1	4	0.00624%	8.62%
Tumor tissue	Polyadenylate-binding protein GN=PABPC4 PE=1 SV=1	B1ANR0_HUMAN	67,971.80	100.00%	6	8	16	0.01220%	19.00%
Tumor tissue	Polyadenylate-binding protein-interacting protein 1 GN=PAIP1 PE=1 SV=1	sp|Q9H074|PAIP1_HUMAN	39,908.60	100.00%	2	2	2	0.00153%	5.70%
Tumor tissue	Polymerase delta-interacting protein 2 GN=POLDIP2 PE=1 SV=1	PDIP2_HUMAN	42,033.00	100.00%	2	2	2	0.00153%	7.07%
Tumor tissue	Polymerase delta-interacting protein 3 GN=POLDIP3 PE=1 SV=2	sp|Q9BY77|PDIP3_HUMAN	46,090.30	100.00%	5	5	7	0.00536%	24.20%
Tumor tissue	Polymerase I and transcript release factor GN=PTRF PE=1 SV=1	sp|Q6NZI2|PTRF_HUMAN	43,476.50	100.00%	8	14	22	0.01680%	25.10%
Pap test	Polymeric immunoglobulin receptor GN=PIGR PE=1 SV=4	PIGR_HUMAN	83,283.40	100.00%	21	48	205	0.32000%	34.30%
Swab	Polymeric immunoglobulin receptor GN=PIGR PE=1 SV=4	PIGR_HUMAN	83,283.40	100.00%	22	46	424	0.30000%	38.90%
Tumor tissue	Polynucleotide 5'-hydroxyl-kinase NOL9 GN=NOL9 PE=1 SV=1	NOL9_HUMAN	79,324.90	100.00%	2	2	2	0.00153%	3.85%
Pap test	Polypeptide N-acetylgalactosaminyltransferase 1 GN=GALNT1 PE=1 SV=1	GALT1_HUMAN	64,219.70	98.60%	1	1	1	0.00156%	5.37%
Tumor tissue	Polypeptide N-acetylgalactosaminyltransferase 1 GN=GALNT1 PE=1 SV=1	GALT1_HUMAN	64,219.70	100.00%	3	3	5	0.00383%	10.00%
Tumor tissue	Polypeptide N-acetylgalactosaminyltransferase 2 GN=GALNT2 PE=1 SV=1	sp|Q10471|GALT2_HUMAN	64,733.10	100.00%	2	3	3	0.00230%	3.68%
Pap test	Polypyrimidine tract binding protein 1, isoform CRA_b GN=PTBP1 PE=1 SV=4	A6NLN1_HUMAN	56,511.80	100.00%	2	2	7	0.01090%	16.50%

Biological sample	Protein name	Protein accession numbers	Protein molecular weight (Da)	Protein identification probability	Exclusive unique peptide count	Exclusive unique spectrum count	Total spectrum count	Percentage of total spectra	Percentage sequence coverage
Swab	Polypyrimidine tract binding protein 1, isoform CRA_b GN=PTBP1 PE=1 SV=4	A6NLN1_HUMAN	56,511.80	100.00%	1	1	1	0.00071%	3.04%
Tumor tissue	Polypyrimidine tract binding protein 1, isoform CRA_b GN=PTBP1 PE=1 SV=4	A6NLN1_HUMAN	56,511.80	100.00%	6	12	49	0.03750%	24.30%
Tumor tissue	Polypyrimidine tract-binding protein 1 (Fragment) GN=PTBP1 PE=1 SV=7	K7EKJ7_HUMAN	25,334.40	100.00%	2	2	22	0.01680%	27.80%
Tumor tissue	Polyribonucleotide nucleotidyltransferase 1, mitochondrial GN=PNPT1 PE=1 SV=2	PNPT1_HUMAN	85,952.10	100.00%	6	6	6	0.00459%	11.10%
Tumor tissue	Porphobilinogen deaminase GN=HMBS PE=1 SV=2	sp|P08397|HEM3_HUMAN	39,330.50	100.00%	2	2	2	0.00153%	11.40%
Tumor tissue	Potassium-transporting ATPase alpha chain 2 GN=ATP12A PE=1 SV=3	sp|P54707|AT12A_HUMAN	115,514.90	99.90%	1	1	6	0.00459%	4.23%
Tumor tissue	PRA1 family protein 3 GN=ARL6IP5 PE=1 SV=1	PRAF3_HUMAN	21,615.60	100.00%	4	6	11	0.00842%	21.30%
Tumor tissue	Pre-B-cell leukemia transcription factor 2 GN=PBX2 PE=1 SV=2	PBX2_HUMAN	45,881.70	99.60%	1	1	2	0.00153%	6.74%
Pap test	Prefoldin subunit 2 GN=PFDN2 PE=1 SV=1	PFD2_HUMAN	16,647.80	99.20%	1	1	1	0.00156%	7.79%
Swab	Prefoldin subunit 2 GN=PFDN2 PE=1 SV=1	PFD2_HUMAN	16,647.80	99.60%	1	1	1	0.00071%	9.09%
Tumor tissue	Prefoldin subunit 2 GN=PFDN2 PE=1 SV=1	PFD2_HUMAN	16,647.80	100.00%	2	2	2	0.00153%	21.40%
Swab	Prefoldin subunit 3 GN=VBP1 PE=1 SV=1	PFD3_HUMAN	22,255.10	99.70%	1	1	2	0.00142%	6.77%
Tumor tissue	Prefoldin subunit 3 GN=VBP1 PE=1 SV=1	PFD3_HUMAN	22,255.10	100.00%	3	3	3	0.00230%	17.70%
Swab	Prefoldin subunit 4 GN=PFDN4 PE=1 SV=1	PFD4_HUMAN	15,598.90	100.00%	2	2	3	0.00213%	19.90%
Tumor tissue	Prefoldin subunit 4 GN=PFDN4 PE=1 SV=1	PFD4_HUMAN	15,598.90	100.00%	3	3	3	0.00230%	20.60%

Biological sample	Protein name	Protein accession numbers	Protein molecular weight (Da)	Protein identification probability	Exclusive unique peptide count	Exclusive unique spectrum count	Total spectrum count	Percentage of total spectra	Percentage sequence coverage
Tumor tissue	Prefoldin subunit 6 GN=PFDN6 PE=1 SV=1	PFD6_HUMAN	14,583.00	100.00%	3	3	3	0.00230%	27.90%
Pap test	Pregnancy zone protein GN=PZP PE=1 SV=4	sp|P20742|PZP_HUMAN	163,862.00	100.00%	5	5	22	0.03430%	7.35%
Swab	Pregnancy zone protein GN=PZP PE=1 SV=4	sp|P20742|PZP_HUMAN	163,862.00	100.00%	3	3	11	0.00780%	6.01%
Tumor tissue	Pregnancy zone protein GN=PZP PE=1 SV=4	sp|P20742|PZP_HUMAN	163,862.00	100.00%	3	3	14	0.01070%	4.93%
Pap test	Prelamin-A/C GN=LMNA PE=1 SV=1	sp|P02545|LMNA_HUMAN	74,140.70	100.00%	4	5	35	0.05460%	35.70%
Swab	Prelamin-A/C GN=LMNA PE=1 SV=1	sp|P02545|LMNA_HUMAN	74,140.70	100.00%	1	1	9	0.00638%	9.79%
Tumor tissue	Prelamin-A/C GN=LMNA PE=1 SV=1	Q5TCI8_HUMAN	55,763.50	100.00%	1	3	172	0.13200%	68.00%
Tumor tissue	Prelamin-A/C GN=LMNA PE=1 SV=1	sp|P02545|LMNA_HUMAN	74,140.70	100.00%	9	15	196	0.15000%	63.00%
Tumor tissue	pre-mRNA 3' end processing protein WDR33 GN=WDR33 PE=1 SV=2	sp|Q9C0J8|WDR33_HUMAN	145,890.70	100.00%	2	2	2	0.00153%	2.32%
Tumor tissue	Pre-mRNA-processing factor 19 GN=PRPF19 PE=1 SV=1	PRP19_HUMAN	55,181.30	100.00%	7	11	14	0.01070%	20.60%
Tumor tissue	Pre-mRNA-processing factor 40 homolog A GN=PRPF40A PE=1 SV=2	sp|O75400|PR40A_HUMAN	108,807.30	100.00%	4	5	5	0.00383%	5.96%
Tumor tissue	Pre-mRNA-processing-splicing factor 8 GN=PRPF8 PE=1 SV=2	PRP8_HUMAN	273,608.60	100.00%	26	30	33	0.02530%	14.30%
Tumor tissue	Pre-mRNA-splicing factor 38A GN=PRPF38A PE=1 SV=1	PR38A_HUMAN	37,477.60	100.00%	2	2	2	0.00153%	8.97%
Tumor tissue	Pre-mRNA-splicing factor 38B GN=PRPF38B PE=1 SV=1	sp|Q5VTL8|PR38B_HUMAN	64,469.20	100.00%	3	3	3	0.00230%	5.49%
Pap test	Pre-mRNA-splicing factor ATP-dependent RNA helicase DHX15 GN=DHX15 PE=1 SV=2	DHX15_HUMAN	90,935.20	99.70%	1	1	1	0.00156%	1.51%
Swab	Pre-mRNA-splicing factor ATP-dependent RNA helicase DHX15 GN=DHX15 PE=1 SV=2	DHX15_HUMAN	90,935.20	100.00%	1	1	1	0.00071%	1.13%
Tumor tissue	Pre-mRNA-splicing factor ATP-dependent RNA helicase DHX15 GN=DHX15 PE=1 SV=2	DHX15_HUMAN	90,935.20	100.00%	19	31	49	0.03750%	29.90%

Biological sample	Protein name	Protein accession numbers	Protein molecular weight (Da)	Protein identification probability	Exclusive unique peptide count	Exclusive unique spectrum count	Total spectrum count	Percentage of total spectra	Percentage sequence coverage
Tumor tissue	Pre-mRNA-splicing factor ATP-dependent RNA helicase PRP16 GN=DHX38 PE=1 SV=2	sp|Q92620|PRP16_HUMAN	140,505.50	100.00%	4	4	4	0.00306%	4.24%
Tumor tissue	Pre-mRNA-splicing factor SPF27 GN=BCAS2 PE=1 SV=1	SPF27_HUMAN	26,131.50	100.00%	3	5	5	0.00383%	18.20%
Tumor tissue	Prenylcysteine oxidase 1 GN=PCYOX1 PE=1 SV=3	sp|Q9UHG3|PCYOX_HUMAN	56,642.20	100.00%	10	14	18	0.01380%	31.10%
Tumor tissue	pre-rRNA processing protein FTSJ3 GN=FTSJ3 PE=1 SV=2	SPB1_HUMAN	96,560.50	100.00%	3	4	4	0.00306%	7.44%
Tumor tissue	Pre-rRNA-processing protein TSR1 homolog GN=TSR1 PE=1 SV=1	TSR1_HUMAN	91,811.40	100.00%	2	2	2	0.00153%	3.61%
Tumor tissue	Presequence protease, mitochondrial GN=PITRM1 PE=1 SV=3	sp|Q5JRX3|PREP_HUMAN	117,415.50	100.00%	2	2	13	0.00995%	13.70%
Tumor tissue	PRKC apoptosis WT1 regulator protein GN=PAWR PE=1 SV=1	PAWR_HUMAN	36,568.10	100.00%	4	4	5	0.00383%	16.80%
Tumor tissue	Probable 2-oxoglutarate dehydrogenase E1 component DHKTD1, mitochondrial GN=DHTKD1 PE=1 SV=2	DHTK1_HUMAN	103,078.40	100.00%	4	6	6	0.00459%	8.27%
Tumor tissue	Probable ATP-dependent RNA helicase DDX17 GN=DDX17 PE=1 SV=1	H3BLZ8_HUMAN	80,441.30	100.00%	15	20	40	0.03060%	31.30%
Tumor tissue	Probable ATP-dependent RNA helicase DDX23 GN=DDX23 PE=1 SV=3	DDX23_HUMAN	95,586.90	100.00%	6	6	7	0.00536%	12.10%
Tumor tissue	Probable ATP-dependent RNA helicase DDX27 GN=DDX27 PE=1 SV=1	DDX27_HUMAN	86,607.40	100.00%	4	4	4	0.00306%	5.75%
Tumor tissue	Probable ATP-dependent RNA helicase DDX31 GN=DDX31 PE=1 SV=2	sp|Q9H8H2|DDX31_HUMAN	83,517.50	100.00%	2	2	2	0.00153%	5.63%
Tumor tissue	Probable ATP-dependent RNA helicase DDX41 GN=DDX41 PE=1 SV=2	DDX41_HUMAN	71,650.70	100.00%	2	2	2	0.00153%	5.14%

Biological sample	Protein name	Protein accession numbers	Protein molecular weight (Da)	Protein identification probability	Exclusive unique peptide count	Exclusive unique spectrum count	Total spectrum count	Percentage of total spectra	Percentage sequence coverage
Tumor tissue	Probable ATP-dependent RNA helicase DDX46 GN=DDX46 PE=1 SV=1	A0A0C4DG89_HUMAN	117,465.20	100.00%	13	13	13	0.00995%	14.30%
Tumor tissue	Probable ATP-dependent RNA helicase DDX47 GN=DDX47 PE=1 SV=1	sp|Q9H0S4|DDX47_HUMAN	50,648.40	100.00%	2	2	2	0.00153%	4.84%
Tumor tissue	Probable ATP-dependent RNA helicase DDX5 GN=DDX5 PE=1 SV=1	sp|P17844|DDX5_HUMAN	69,149.70	100.00%	10	13	23	0.01760%	25.10%
Tumor tissue	Probable ATP-dependent RNA helicase DDX52 GN=DDX52 PE=1 SV=3	DDX52_HUMAN	67,500.40	100.00%	2	2	2	0.00153%	7.18%
Tumor tissue	Probable ATP-dependent RNA helicase DDX58 GN=DDX58 PE=1 SV=2	sp|O95786|DDX58_HUMAN	106,604.00	100.00%	5	5	5	0.00383%	6.27%
Tumor tissue	Probable ATP-dependent RNA helicase DDX6 GN=DDX6 PE=1 SV=2	DDX6_HUMAN	54,418.60	100.00%	8	10	14	0.01070%	26.90%
Tumor tissue	Probable ATP-dependent RNA helicase DDX60 GN=DDX60 PE=1 SV=3	DDX60_HUMAN	197,858.90	100.00%	1	1	2	0.00153%	1.46%
Tumor tissue	Probable carboxypeptidase X1 GN=CPXM1 PE=2 SV=2	CPXM1_HUMAN	81,668.30	100.00%	3	3	3	0.00230%	5.72%
Tumor tissue	Probable cysteine--tRNA ligase, mitochondrial GN=CARS2 PE=1 SV=1	SYCM_HUMAN	62,225.20	100.00%	2	3	5	0.00383%	6.56%
Tumor tissue	Probable global transcription activator SNF2L1 GN=SMARCA1 PE=1 SV=1	sp|P28370|SMCA1_HUMAN	124,332.20	100.00%	1	1	5	0.00383%	4.49%
Tumor tissue	Probable rRNA-processing protein EBP2 GN=EBNA1BP2 PE=1 SV=2	EBP2_HUMAN	40,685.40	100.00%	3	3	3	0.00230%	10.50%
Swab	Probable serine carboxypeptidase CPVL GN=CPVL PE=1 SV=2	CPVL_HUMAN	54,165.60	99.70%	1	1	1	0.00071%	1.68%
Tumor tissue	Probable serine carboxypeptidase CPVL GN=CPVL PE=1 SV=2	CPVL_HUMAN	54,165.60	100.00%	7	11	15	0.01150%	26.10%
Pap test	Pro-cathepsin H GN=CTSH PE=1 SV=1	CATH_HUMAN	36,269.60	100.00%	3	5	5	0.00780%	10.20%

Biological sample	Protein name	Protein accession numbers	Protein molecular weight (Da)	Protein identification probability	Exclusive unique peptide count	Exclusive unique spectrum count	Total spectrum count	Percentage of total spectra	Percentage sequence coverage
Swab	Pro-cathepsin H GN=CTSH PE=1 SV=1	CATH_HUMAN	36,269.60	100.00%	3	4	5	0.00354%	7.74%
Tumor tissue	Pro-cathepsin H GN=CTSH PE=1 SV=1	CATH_HUMAN	36,269.60	100.00%	1	1	1	0.00077%	4.02%
Pap test	Procollagen C-endopeptidase enhancer 1 GN=PCOLCE PE=1 SV=2	PCOC1_HUMAN	47,972.50	99.30%	1	1	1	0.00156%	3.34%
Swab	Procollagen C-endopeptidase enhancer 1 GN=PCOLCE PE=1 SV=2	PCOC1_HUMAN	47,972.50	99.90%	1	1	1	0.00071%	2.45%
Tumor tissue	Procollagen C-endopeptidase enhancer 1 GN=PCOLCE PE=1 SV=2	PCOC1_HUMAN	47,972.50	100.00%	4	5	6	0.00459%	13.80%
Tumor tissue	Procollagen galactosyltransferase 1 GN=COLGALT1 PE=1 SV=1	GT251_HUMAN	71,637.70	100.00%	11	13	14	0.01070%	20.40%
Tumor tissue	Procollagen-lysine,2-oxoglutarate 5-dioxygenase 3 GN=PLOD3 PE=1 SV=1	PLOD3_HUMAN	84,786.80	100.00%	9	13	15	0.01150%	17.20%
Tumor tissue	Profilin GN=PFN2 PE=1 SV=1	sp|P35080-2|PROF2_HUMAN	20,787.50	99.60%	1	1	4	0.00306%	40.70%
Tumor tissue	Profilin GN=PFN2 PE=1 SV=1	C9J712_HUMAN	9,798.40	99.90%	1	1	5	0.00383%	40.70%
Pap test	Profilin-1 GN=PFN1 PE=1 SV=2	PROF1_HUMAN	15,054.30	100.00%	9	16	46	0.07180%	70.00%
Swab	Profilin-1 GN=PFN1 PE=1 SV=2	PROF1_HUMAN	15,054.30	100.00%	9	16	76	0.05390%	61.40%
Tumor tissue	Profilin-1 GN=PFN1 PE=1 SV=2	PROF1_HUMAN	15,054.30	100.00%	9	20	65	0.04980%	58.60%
Pap test	Programmed cell death 6-interacting protein GN=PDCD6IP PE=1 SV=1	sp|Q8WUM4|PDC6I_HUMAN	96,025.40	100.00%	9	9	13	0.02030%	13.50%
Swab	Programmed cell death 6-interacting protein GN=PDCD6IP PE=1 SV=1	sp|Q8WUM4|PDC6I_HUMAN	96,025.40	100.00%	15	17	25	0.01770%	27.80%
Tumor tissue	Programmed cell death 6-interacting protein GN=PDCD6IP PE=1 SV=1	sp|Q8WUM4|PDC6I_HUMAN	96,025.40	100.00%	19	28	40	0.03060%	31.50%
Tumor tissue	Programmed cell death protein 10 GN=PDCD10 PE=1 SV=1	PDC10_HUMAN	24,702.50	100.00%	4	5	6	0.00459%	21.20%
Pap test	Programmed cell death protein 5 GN=PDCD5 PE=1 SV=3	sp|O14737|PDCD5_HUMAN	14,285.30	100.00%	1	1	1	0.00156%	10.40%

Biological sample	Protein name	Protein accession numbers	Protein molecular weight (Da)	Protein identification probability	Exclusive unique peptide count	Exclusive unique spectrum count	Total spectrum count	Percentage of total spectra	Percentage sequence coverage
Tumor tissue	Programmed cell death protein 5 GN=PDCD5 PE=1 SV=3	sp|O14737|PDCD5_HUMAN	14,285.30	100.00%	3	5	5	0.00383%	28.00%
Tumor tissue	Programmed cell death protein 6 GN=PDCD6 PE=1 SV=1	sp|O75340|PDCD6_HUMAN	21,869.10	100.00%	1	1	9	0.00689%	34.60%
Pap test	Prohibitin GN=PHB PE=1 SV=1	sp|P35232|PHB_HUMAN	29,804.60	100.00%	3	3	4	0.00624%	11.80%
Tumor tissue	Prohibitin GN=PHB PE=1 SV=1	sp|P35232|PHB_HUMAN	29,804.60	100.00%	11	15	34	0.02600%	54.40%
Tumor tissue	Prohibitin-2 GN=PHB2 PE=1 SV=2	sp|Q99623|PHB2_HUMAN	33,240.60	100.00%	2	2	30	0.02300%	47.00%
Tumor tissue	Prolactin regulatory element-binding protein GN=PREB PE=1 SV=2	PREB_HUMAN	45,468.30	100.00%	4	5	5	0.00383%	14.40%
Tumor tissue	Prolargin GN=PRELP PE=1 SV=1	PRELP_HUMAN	43,812.40	100.00%	12	18	23	0.01760%	35.60%
Swab	Proliferating cell nuclear antigen GN=PCNA PE=1 SV=1	PCNA_HUMAN	28,769.30	99.90%	1	1	1	0.00071%	7.28%
Tumor tissue	Proliferating cell nuclear antigen GN=PCNA PE=1 SV=1	PCNA_HUMAN	28,769.30	100.00%	5	7	9	0.00689%	23.40%
Tumor tissue	Proliferation marker protein Ki-67 GN=MKI67 PE=1 SV=2	sp|P46013|KI67_HUMAN	358,695.80	100.00%	2	2	2	0.00153%	1.23%
Tumor tissue	Proliferation-associated protein 2G4 GN=PA2G4 PE=1 SV=3	sp|Q9UQ80|PA2G4_HUMAN	43,786.70	100.00%	10	12	20	0.01530%	30.70%
Tumor tissue	Proline synthase co-transcribed bacterial homolog protein GN=PROSC PE=1 SV=1	PROSC_HUMAN	30,343.80	100.00%	4	4	4	0.00306%	20.70%
Tumor tissue	Proline-, glutamic acid- and leucine-rich protein 1 GN=PELP1 PE=1 SV=2	PELP1_HUMAN	124,944.60	100.00%	2	4	10	0.00766%	2.97%
Pap test	Prolow-density lipoprotein receptor-related protein 1 GN=LRP1 PE=1 SV=2	LRP1_HUMAN	504,591.80	100.00%	1	1	1	0.00156%	0.66%
Tumor tissue	Prolow-density lipoprotein receptor-related protein 1 GN=LRP1 PE=1 SV=2	LRP1_HUMAN	504,591.80	100.00%	31	37	44	0.03370%	10.00%
Tumor tissue	Prolyl 3-hydroxylase 3 GN=P3H3 PE=1 SV=1	sp|Q8IVL6|P3H3_HUMAN	81,837.90	100.00%	4	4	6	0.00459%	11.70%

Biological sample	Protein name	Protein accession numbers	Protein molecular weight (Da)	Protein identification probability	Exclusive unique peptide count	Exclusive unique spectrum count	Total spectrum count	Percentage of total spectra	Percentage sequence coverage
Tumor tissue	Prolyl 4-hydroxylase subunit alpha-1 GN=P4HA1 PE=1 SV=2	sp|P13674|P4HA1_HUMAN	61,051.60	100.00%	1	1	27	0.02070%	35.40%
Tumor tissue	Prolyl 4-hydroxylase subunit alpha-2 GN=P4HA2 PE=1 SV=1	sp|O15460|P4HA2_HUMAN	60,902.90	100.00%	10	12	13	0.00995%	22.20%
Pap test	Prolyl endopeptidase GN=PREP PE=1 SV=2	PPCE_HUMAN	80,701.50	100.00%	4	4	4	0.00624%	7.61%
Swab	Prolyl endopeptidase GN=PREP PE=1 SV=2	PPCE_HUMAN	80,701.50	100.00%	6	6	8	0.00567%	12.70%
Tumor tissue	Prolyl endopeptidase GN=PREP PE=1 SV=2	PPCE_HUMAN	80,701.50	100.00%	10	11	12	0.00919%	19.90%
Tumor tissue	Prolyl endopeptidase FAP GN=FAP PE=1 SV=1	sp|Q12884|SEPR_HUMAN	84,864.90	100.00%	11	13	13	0.00995%	18.30%
Pap test	Propionyl-CoA carboxylase beta chain, mitochondrial GN=PCCB PE=1 SV=1	sp|P05166|PCCB_HUMAN	59,403.70	99.20%	1	1	1	0.00156%	2.68%
Tumor tissue	Propionyl-CoA carboxylase beta chain, mitochondrial GN=PCCB PE=1 SV=1	sp|P05166|PCCB_HUMAN	56,267.70	100.00%	6	7	7	0.00536%	15.20%
Tumor tissue	Prosaposin GN=PSAP PE=1 SV=2	sp|P07602|SAP_HUMAN	58,112.00	100.00%	1	1	5	0.00383%	10.50%
Tumor tissue	Prostacyclin synthase GN=PTGIS PE=1 SV=1	PTGIS_HUMAN	57,106.60	100.00%	10	15	26	0.01990%	27.00%
Tumor tissue	Prostaglandin E synthase 2 GN=PTGES2 PE=1 SV=1	PGES2_HUMAN	41,943.60	100.00%	1	2	6	0.00459%	15.90%
Pap test	Prostaglandin E synthase 3 GN=PTGES3 PE=1 SV=1	sp|Q15185|TEBP_HUMAN	19,155.40	100.00%	2	2	2	0.00312%	18.30%
Swab	Prostaglandin E synthase 3 GN=PTGES3 PE=1 SV=1	sp|Q15185|TEBP_HUMAN	19,155.40	100.00%	2	2	4	0.00283%	18.30%
Pap test	Prostaglandin F2 receptor negative regulator GN=PTGFRN PE=1 SV=2	FPRP_HUMAN	98,555.60	99.30%	1	1	1	0.00156%	1.59%
Tumor tissue	Prostaglandin F2 receptor negative regulator GN=PTGFRN PE=1 SV=2	FPRP_HUMAN	98,555.60	100.00%	10	12	13	0.00995%	12.50%
Tumor tissue	Prostaglandin reductase 2 GN=PTGR2 PE=1 SV=1	G3V2R9_HUMAN	23,380.20	100.00%	2	2	2	0.00153%	14.70%
Pap test	Prostate stem cell antigen GN=PSCA PE=1 SV=1	PSCA_HUMAN	12,912.30	100.00%	2	2	5	0.00780%	22.00%

Biological sample	Protein name	Protein accession numbers	Protein molecular weight (Da)	Protein identification probability	Exclusive unique peptide count	Exclusive unique spectrum count	Total spectrum count	Percentage of total spectra	Percentage sequence coverage
Swab	Prostate stem cell antigen GN=PSCA PE=1 SV=1	PSCA_HUMAN	12,912.30	99.90%	2	2	5	0.00354%	22.00%
Swab	Proteasomal ubiquitin receptor ADRM1 GN=ADRM1 PE=1 SV=2	ADRM1_HUMAN	42,154.30	100.00%	2	2	4	0.00283%	4.91%
Pap test	Proteasome activator complex subunit 1 GN=PSME1 PE=1 SV=1	H0YKK6_HUMAN	10,576.70	99.80%	1	2	12	0.01870%	46.70%
Tumor tissue	Proteasome activator complex subunit 1 GN=PSME1 PE=1 SV=1	H0YKK6_HUMAN	10,576.70	99.80%	1	2	15	0.01150%	46.70%
Tumor tissue	Proteasome activator complex subunit 2 GN=PSME2 PE=1 SV=1	A0A087X1Z3_HUMAN	29,127.60	100.00%	1	2	33	0.02530%	42.50%
Tumor tissue	Proteasome inhibitor PI31 subunit GN=PSMF1 PE=1 SV=2	PSMF1_HUMAN	28,957.30	100.00%	2	3	3	0.00230%	9.96%
Pap test	Proteasome subunit alpha type GN=PSMA2 PE=1 SV=1	A0A024RA52_HUMAN	25,898.90	100.00%	5	7	11	0.01720%	38.00%
Swab	Proteasome subunit alpha type GN=PSMA2 PE=1 SV=1	A0A024RA52_HUMAN	25,898.90	100.00%	2	2	4	0.00283%	18.40%
Tumor tissue	Proteasome subunit alpha type GN=PSMA2 PE=1 SV=1	A0A024RA52_HUMAN	25,898.90	100.00%	5	8	16	0.01220%	29.90%
Pap test	Proteasome subunit alpha type GN=PSMA6 PE=1 SV=1	sp|P60900|PSA6_HUMAN	28,147.50	100.00%	2	3	27	0.04210%	47.60%
Swab	Proteasome subunit alpha type GN=PSMA6 PE=1 SV=1	sp|P60900|PSA6_HUMAN	28,147.50	100.00%	1	1	5	0.00354%	19.80%
Tumor tissue	Proteasome subunit alpha type GN=PSMA6 PE=1 SV=1	sp|P60900|PSA6_HUMAN	28,147.50	100.00%	1	1	8	0.00612%	25.00%
Pap test	Proteasome subunit alpha type-4 GN=PSMA4 PE=1 SV=1	sp|P25789|PSA4_HUMAN	29,484.80	100.00%	3	3	10	0.01560%	34.50%
Swab	Proteasome subunit alpha type-4 GN=PSMA4 PE=1 SV=1	sp|P25789|PSA4_HUMAN	29,484.80	100.00%	2	2	7	0.00496%	23.80%
Tumor tissue	Proteasome subunit alpha type-4 GN=PSMA4 PE=1 SV=1	sp|P25789|PSA4_HUMAN	29,484.80	100.00%	1	1	4	0.00306%	11.50%
Pap test	Proteasome subunit alpha type-5 GN=PSMA5 PE=1 SV=3	sp|P28066|PSA5_HUMAN	26,411.30	100.00%	7	9	14	0.02180%	43.60%
Swab	Proteasome subunit alpha type-5 GN=PSMA5 PE=1 SV=3	sp|P28066|PSA5_HUMAN	26,411.30	100.00%	3	3	5	0.00354%	21.20%
Tumor tissue	Proteasome subunit alpha type-5 GN=PSMA5 PE=1 SV=3	sp|P28066|PSA5_HUMAN	26,411.30	100.00%	5	8	8	0.00612%	31.50%

Biological sample	Protein name	Protein accession numbers	Protein molecular weight (Da)	Protein identification probability	Exclusive unique peptide count	Exclusive unique spectrum count	Total spectrum count	Percentage of total spectra	Percentage sequence coverage
Pap test	Proteasome subunit alpha type-7 GN=PSMA7 PE=1 SV=1	sp|O14818|PSA7_HUMAN	27,887.20	100.00%	2	2	12	0.01870%	36.70%
Swab	Proteasome subunit alpha type-7 GN=PSMA7 PE=1 SV=1	sp|O14818|PSA7_HUMAN	27,887.20	100.00%	3	3	6	0.00425%	27.00%
Tumor tissue	Proteasome subunit alpha type-7 GN=PSMA7 PE=1 SV=1	sp|O14818|PSA7_HUMAN	27,887.20	100.00%	1	1	9	0.00689%	29.80%
Pap test	Proteasome subunit beta type GN=PSM8 PE=1 SV=1	sp|P28062|PSB8_HUMAN	30,354.80	100.00%	6	7	13	0.02030%	22.10%
Swab	Proteasome subunit beta type GN=PSM8 PE=1 SV=1	sp|P28062|PSB8_HUMAN	30,354.80	100.00%	4	5	6	0.00425%	17.00%
Tumor tissue	Proteasome subunit beta type GN=PSM8 PE=1 SV=1	sp|P28062|PSB8_HUMAN	30,354.80	100.00%	6	9	9	0.00689%	24.30%
Pap test	Proteasome subunit beta type GN=PSMB9 PE=1 SV=1	sp|P28065|PSB9_HUMAN	20,960.70	100.00%	3	3	3	0.00468%	21.90%
Swab	Proteasome subunit beta type GN=PSMB9 PE=1 SV=1	sp|P28065|PSB9_HUMAN	20,960.70	99.40%	1	1	2	0.00142%	6.12%
Tumor tissue	Proteasome subunit beta type GN=PSMB9 PE=1 SV=1	sp|P28065|PSB9_HUMAN	20,960.70	100.00%	3	3	4	0.00306%	29.60%
Pap test	Proteasome subunit beta type-1 GN=PSMB1 PE=1 SV=2	PSB1_HUMAN	26,490.50	100.00%	5	6	8	0.01250%	30.70%
Swab	Proteasome subunit beta type-1 GN=PSMB1 PE=1 SV=2	PSB1_HUMAN	26,490.50	100.00%	6	9	11	0.00780%	30.30%
Tumor tissue	Proteasome subunit beta type-1 GN=PSMB1 PE=1 SV=2	PSB1_HUMAN	26,490.50	100.00%	6	10	11	0.00842%	35.30%
Pap test	Proteasome subunit beta type-10 GN=PSMB10 PE=1 SV=1	PSB10_HUMAN	28,936.00	99.80%	1	2	3	0.00468%	7.33%
Swab	Proteasome subunit beta type-10 GN=PSMB10 PE=1 SV=1	PSB10_HUMAN	28,936.00	99.60%	1	2	3	0.00213%	7.33%
Tumor tissue	Proteasome subunit beta type-10 GN=PSMB10 PE=1 SV=1	PSB10_HUMAN	28,936.00	100.00%	5	6	8	0.00612%	39.20%
Pap test	Proteasome subunit beta type-2 GN=PSMB2 PE=1 SV=1	PSB2_HUMAN	22,837.50	100.00%	5	7	10	0.01560%	28.40%
Swab	Proteasome subunit beta type-2 GN=PSMB2 PE=1 SV=1	PSB2_HUMAN	22,837.50	100.00%	2	3	4	0.00283%	11.40%
Tumor tissue	Proteasome subunit beta type-2 GN=PSMB2 PE=1 SV=1	PSB2_HUMAN	22,837.50	100.00%	7	9	13	0.00995%	51.70%

Biological sample	Protein name	Protein accession numbers	Protein molecular weight (Da)	Protein identification probability	Exclusive unique peptide count	Exclusive unique spectrum count	Total spectrum count	Percentage of total spectra	Percentage sequence coverage
Pap test	Proteasome subunit beta type-3 GN=PSMB3 PE=1 SV=2	PSB3_HUMAN	22,949.60	100.00%	1	2	13	0.02030%	35.60%
Tumor tissue	Proteasome subunit beta type-3 GN=PSMB3 PE=1 SV=2	PSB3_HUMAN	22,949.60	100.00%	2	3	13	0.00995%	33.70%
Pap test	Proteasome subunit beta type-4 GN=PSMB4 PE=1 SV=4	PSB4_HUMAN	29,205.00	100.00%	5	8	11	0.01720%	33.00%
Swab	Proteasome subunit beta type-4 GN=PSMB4 PE=1 SV=4	PSB4_HUMAN	29,205.00	100.00%	4	6	7	0.00496%	21.20%
Tumor tissue	Proteasome subunit beta type-4 GN=PSMB4 PE=1 SV=4	PSB4_HUMAN	29,205.00	100.00%	4	6	7	0.00536%	25.40%
Pap test	Proteasome subunit beta type-5 GN=PSMB5 PE=1 SV=3	sp|P28074|PSB5_HUMAN	28,481.00	100.00%	2	2	4	0.00624%	14.40%
Swab	Proteasome subunit beta type-5 GN=PSMB5 PE=1 SV=3	sp|P28074|PSB5_HUMAN	28,481.00	99.50%	1	1	1	0.00071%	6.08%
Tumor tissue	Proteasome subunit beta type-5 GN=PSMB5 PE=1 SV=3	sp|P28074|PSB5_HUMAN	28,481.00	100.00%	2	2	3	0.00230%	14.80%
Pap test	Proteasome subunit beta type-6 GN=PSMB6 PE=1 SV=4	PSB6_HUMAN	25,357.90	100.00%	1	1	1	0.00156%	4.60%
Tumor tissue	Proteasome subunit beta type-6 GN=PSMB6 PE=1 SV=4	PSB6_HUMAN	25,357.90	100.00%	3	3	3	0.00230%	13.40%
Tumor tissue	Proteasome subunit beta type-7 GN=PSMB7 PE=1 SV=1	sp|Q99436|PSB7_HUMAN	29,965.90	100.00%	2	2	2	0.00153%	8.30%
Tumor tissue	Proteasome-associated protein ECM29 homolog GN=KIAA0368 PE=1 SV=1	J3KN16_HUMAN	223,699.20	100.00%	12	13	14	0.01070%	8.38%
Tumor tissue	Protein AAR2 homolog GN=AAR2 PE=1 SV=1	AAR2_HUMAN	45,035.10	100.00%	2	2	2	0.00153%	7.54%
Tumor tissue	Protein AATF GN=AATF PE=1 SV=1	AATF_HUMAN	63,135.00	100.00%	3	3	3	0.00230%	7.50%
Pap test	Protein ABHD14B GN=ABHD14B PE=1 SV=1	sp|Q96IU4|ABHEB_HUMAN	22,346.10	100.00%	3	3	3	0.00468%	17.60%
Swab	Protein ABHD14B GN=ABHD14B PE=1 SV=1	sp|Q96IU4|ABHEB_HUMAN	22,346.10	100.00%	6	8	11	0.00780%	39.50%
Tumor tissue	Protein ABHD14B GN=ABHD14B PE=1 SV=1	sp|Q96IU4|ABHEB_HUMAN	22,346.10	100.00%	5	5	5	0.00383%	33.30%
Pap test	Protein AMBP GN=AMBP PE=1 SV=1	AMBP_HUMAN	38,999.60	100.00%	6	6	25	0.03900%	39.80%

Biological sample	Protein name	Protein accession numbers	Protein molecular weight (Da)	Protein identification probability	Exclusive unique peptide count	Exclusive unique spectrum count	Total spectrum count	Percentage of total spectra	Percentage sequence coverage
Swab	Protein AMBP GN=AMBP PE=1 SV=1	AMBP_HUMAN	38,999.60	100.00%	4	5	22	0.01560%	36.90%
Tumor tissue	Protein AMBP GN=AMBP PE=1 SV=1	AMBP_HUMAN	38,999.60	100.00%	1	1	5	0.00383%	11.10%
Tumor tissue	Protein arginine N-methyltransferase 5 GN=PRMT5 PE=1 SV=4	sp|O14744|ANM5_HUMAN	72,685.10	100.00%	4	4	5	0.00383%	8.48%
Tumor tissue	Protein argonaute-1 GN=AGO1 PE=1 SV=3	AGO1_HUMAN	97,215.00	100.00%	3	3	6	0.00459%	8.52%
Pap test	Protein ATP5J2-PTCD1 GN=ATP5J2-PTCD1 PE=4 SV=1	G3V325_HUMAN	84,112.70	99.80%	1	1	1	0.00156%	1.74%
Tumor tissue	Protein ATP5J2-PTCD1 GN=ATP5J2-PTCD1 PE=4 SV=1	G3V325_HUMAN	84,112.70	100.00%	2	2	4	0.00306%	3.20%
Tumor tissue	Protein C10 GN=C12orf57 PE=1 SV=1	C10_HUMAN	13,178.20	99.90%	1	1	2	0.00153%	26.20%
Tumor tissue	Protein canopy homolog 3 GN=CNPY3 PE=1 SV=1	CNPY3_HUMAN	30,748.70	100.00%	3	3	3	0.00230%	10.10%
Swab	Protein CASC4 GN=CASC4 PE=1 SV=1	sp|Q6P4E1|CASC4_HUMAN	48,864.80	99.70%	1	1	1	0.00071%	3.93%
Tumor tissue	Protein CASC4 GN=CASC4 PE=1 SV=1	sp|Q6P4E1|CASC4_HUMAN	48,864.80	99.90%	1	1	2	0.00153%	5.77%
Tumor tissue	Protein CDV3 homolog GN=CDV3 PE=1 SV=1	sp|Q9UKY7|CDV3_HUMAN	27,334.70	100.00%	5	5	5	0.00383%	26.00%
Pap test	Protein deglycase DJ-1 GN=PARK7 PE=1 SV=2	PARK7_HUMAN	19,891.20	100.00%	5	6	6	0.00936%	24.90%
Swab	Protein deglycase DJ-1 GN=PARK7 PE=1 SV=2	PARK7_HUMAN	19,891.20	100.00%	6	10	18	0.01280%	38.10%
Tumor tissue	Protein deglycase DJ-1 GN=PARK7 PE=1 SV=2	PARK7_HUMAN	19,891.20	100.00%	7	13	21	0.01610%	39.70%
Tumor tissue	Protein DEK GN=DEK PE=1 SV=1	sp|P35659|DEK_HUMAN	42,675.90	100.00%	3	3	4	0.00306%	9.60%
Swab	Protein diaphanous homolog 1 GN=DIAPH1 PE=1 SV=1	sp|O60610|DIAP1_HUMAN	141,364.00	98.70%	1	1	1	0.00071%	1.26%
Tumor tissue	Protein diaphanous homolog 1 GN=DIAPH1 PE=1 SV=1	sp|O60610|DIAP1_HUMAN	141,364.00	100.00%	8	11	12	0.00919%	8.49%
Pap test	Protein disulfide-isomerase GN=P4HB PE=1 SV=3	PDIA1_HUMAN	57,118.10	100.00%	4	4	22	0.03430%	28.90%

Biological sample	Protein name	Protein accession numbers	Protein molecular weight (Da)	Protein identification probability	Exclusive unique peptide count	Exclusive unique spectrum count	Total spectrum count	Percentage of total spectra	Percentage sequence coverage
Swab	Protein disulfide-isomerase GN=P4HB PE=1 SV=3	PDIA1_HUMAN	57,118.10	100.00%	7	7	35	0.02480%	44.10%
Tumor tissue	Protein disulfide-isomerase GN=P4HB PE=1 SV=3	PDIA1_HUMAN	57,118.10	100.00%	9	19	81	0.06200%	47.40%
Pap test	Protein disulfide-isomerase A3 GN=PDIA3 PE=1 SV=4	PDIA3_HUMAN	56,784.60	100.00%	9	9	13	0.02030%	28.10%
Swab	Protein disulfide-isomerase A3 GN=PDIA3 PE=1 SV=4	PDIA3_HUMAN	56,784.60	100.00%	6	6	8	0.00567%	16.40%
Tumor tissue	Protein disulfide-isomerase A3 GN=PDIA3 PE=1 SV=4	PDIA3_HUMAN	56,784.60	100.00%	20	35	72	0.05510%	53.90%
Pap test	Protein disulfide-isomerase A3 (Fragment) GN=PDIA3 PE=1 SV=1	H7BZJ3_HUMAN	13,519.70	99.40%	1	1	4	0.00624%	28.50%
Swab	Protein disulfide-isomerase A3 (Fragment) GN=PDIA3 PE=1 SV=1	H7BZJ3_HUMAN	13,519.70	99.60%	1	1	4	0.00283%	20.30%
Tumor tissue	Protein disulfide-isomerase A3 (Fragment) GN=PDIA3 PE=1 SV=1	H7BZJ3_HUMAN	13,519.70	98.60%	1	2	13	0.00995%	48.80%
Pap test	Protein disulfide-isomerase A4 GN=PDIA4 PE=1 SV=2	PDIA4_HUMAN	72,934.00	100.00%	2	2	3	0.00468%	3.41%
Swab	Protein disulfide-isomerase A4 GN=PDIA4 PE=1 SV=2	PDIA4_HUMAN	72,934.00	100.00%	7	8	10	0.00709%	15.00%
Tumor tissue	Protein disulfide-isomerase A4 GN=PDIA4 PE=1 SV=2	PDIA4_HUMAN	72,934.00	100.00%	29	44	68	0.05210%	46.20%
Tumor tissue	Protein disulfide-isomerase A5 GN=PDIA5 PE=1 SV=1	sp|Q14554|PDIA5_HUMAN	59,595.10	100.00%	11	16	19	0.01450%	25.60%
Tumor tissue	Protein disulfide-isomerase TMX3 GN=TMX3 PE=1 SV=2	sp|Q96JJ7|TMX3_HUMAN	51,872.80	100.00%	2	2	2	0.00153%	9.91%
Tumor tissue	Protein enabled homolog GN=ENAH PE=1 SV=1	sp|Q8N8S7|ENAH_HUMAN	87,400.60	100.00%	3	5	16	0.01220%	16.60%
Tumor tissue	Protein ERGIC-53 GN=LMAN1 PE=1 SV=2	LMAN1_HUMAN	57,549.20	100.00%	14	23	35	0.02680%	32.00%
Tumor tissue	Protein FAM134C GN=FAM134C PE=1 SV=1	sp|Q86VR2|F134C_HUMAN	51,397.40	100.00%	2	2	2	0.00153%	7.08%
Tumor tissue	Protein FAM160B2 GN=FAM160B2 PE=2 SV=2	F16B2_HUMAN	82,341.60	100.00%	2	2	2	0.00153%	6.59%
Tumor tissue	Protein FAM162A GN=FAM162A PE=1 SV=1	F162A_HUMAN	15,959.40	100.00%	2	2	2	0.00153%	19.90%

Biological sample	Protein name	Protein accession numbers	Protein molecular weight (Da)	Protein identification probability	Exclusive unique peptide count	Exclusive unique spectrum count	Total spectrum count	Percentage of total spectra	Percentage sequence coverage
Pap test	Protein FAM3C GN=FAM3C PE=1 SV=1	FAM3C_HUMAN	24,680.90	100.00%	2	4	5	0.00780%	12.30%
Swab	Protein FAM3C GN=FAM3C PE=1 SV=1	FAM3C_HUMAN	24,680.90	99.20%	1	1	1	0.00071%	5.73%
Tumor tissue	Protein FAM3C GN=FAM3C PE=1 SV=1	FAM3C_HUMAN	24,680.90	100.00%	5	6	8	0.00612%	30.00%
Tumor tissue	Protein FAM49A GN=FAM49A PE=2 SV=1	FA49A_HUMAN	37,313.90	100.00%	3	3	3	0.00230%	14.20%
Pap test	Protein FAM49B GN=FAM49B PE=1 SV=1	sp|Q9NUQ9|FA49B_HUMAN	36,748.70	100.00%	2	2	3	0.00468%	14.50%
Swab	Protein FAM49B GN=FAM49B PE=1 SV=1	sp|Q9NUQ9|FA49B_HUMAN	36,748.70	100.00%	3	5	17	0.01200%	21.30%
Tumor tissue	Protein FAM49B GN=FAM49B PE=1 SV=1	sp|Q9NUQ9|FA49B_HUMAN	36,748.70	100.00%	7	14	23	0.01760%	38.60%
Tumor tissue	Protein FAM83H GN=FAM83H PE=1 SV=3	FA83H_HUMAN	127,123.90	100.00%	9	9	10	0.00766%	12.00%
Tumor tissue	Protein FAM84B GN=FAM84B PE=1 SV=1	FA84B_HUMAN	34,474.00	100.00%	2	3	3	0.00230%	10.00%
Tumor tissue	Protein farnesyltransferase subunit beta GN=FNTB PE=1 SV=1	sp|P49356|FNTB_HUMAN	48,774.00	100.00%	4	4	4	0.00306%	8.70%
Tumor tissue	Protein flightless-1 homolog GN=FLII PE=1 SV=2	sp|Q13045|FLII_HUMAN	144,755.40	100.00%	6	7	10	0.00766%	8.27%
Tumor tissue	Protein HGH1 homolog GN=HGH1 PE=1 SV=1	HGH1_HUMAN	42,129.90	100.00%	2	2	2	0.00153%	5.90%
Tumor tissue	Protein Hook homolog 3 GN=HOOK3 PE=1 SV=2	HOOK3_HUMAN	83,128.00	100.00%	6	6	8	0.00612%	10.70%
Pap test	Protein IGHV1-69-2 (Fragment) GN=IGHV1-69-2 PE=1 SV=1	A0A0B4J2H0_HUMAN	12,660.10	99.90%	1	3	16	0.02500%	60.70%
Swab	Protein IGHV1-69-2 (Fragment) GN=IGHV1-69-2 PE=1 SV=1	A0A0B4J2H0_HUMAN	12,660.10	99.60%	1	2	19	0.01350%	26.50%
Tumor tissue	Protein IGHV1-69-2 (Fragment) GN=IGHV1-69-2 PE=1 SV=1	A0A0B4J2H0_HUMAN	12,660.10	99.40%	1	1	2	0.00153%	29.90%
Pap test	Protein IGHV3-72 GN=IGHV3-72 PE=1 SV=1	A0A087WW89_HUMAN	11,167.50	100.00%	2	2	33	0.05150%	52.50%
Swab	Protein IGHV3-72 GN=IGHV3-72 PE=1 SV=1	A0A087WW89_HUMAN	11,167.50	100.00%	1	1	54	0.03830%	41.60%

Biological sample	Protein name	Protein accession numbers	Protein molecular weight (Da)	Protein identification probability	Exclusive unique peptide count	Exclusive unique spectrum count	Total spectrum count	Percentage of total spectra	Percentage sequence coverage
Pap test	Protein IGHV3-74 (Fragment) GN=IGHV3-74 PE=1 SV=1	A0A0B4J1X5_HUMAN	12,839.50	99.90%	1	1	34	0.05310%	31.60%
Swab	Protein IGHV3-74 (Fragment) GN=IGHV3-74 PE=1 SV=1	A0A0B4J1X5_HUMAN	12,839.50	99.90%	1	1	34	0.02410%	31.60%
Tumor tissue	Protein IGHV3-74 (Fragment) GN=IGHV3-74 PE=1 SV=1	A0A0B4J1X5_HUMAN	12,839.50	99.60%	1	1	6	0.00459%	22.20%
Pap test	Protein IGHV3OR16-12 (Fragment) GN=IGHV3OR16-12 PE=1 SV=1	A0A075B7B8_HUMAN	12,874.50	99.40%	1	2	42	0.06550%	18.80%
Swab	Protein IGHV3OR16-12 (Fragment) GN=IGHV3OR16-12 PE=1 SV=1	A0A075B7B8_HUMAN	12,874.50	99.60%	1	1	35	0.02480%	18.80%
Pap test	Protein IGHV4-28 (Fragment) GN=IGHV4-28 PE=1 SV=1	A0A0C4DH34_HUMAN	13,124.30	99.10%	1	2	21	0.03280%	7.69%
Swab	Protein IGHV4-28 (Fragment) GN=IGHV4-28 PE=1 SV=1	A0A0C4DH34_HUMAN	13,124.30	100.00%	2	2	2	0.00142%	12.80%
Pap test	Protein IGHV5-51 (Fragment) GN=IGHV5-51 PE=1 SV=1	A0A0C4DH38_HUMAN	12,674.90	100.00%	2	6	29	0.04530%	47.00%
Swab	Protein IGHV5-51 (Fragment) GN=IGHV5-51 PE=1 SV=1	A0A0C4DH38_HUMAN	12,674.90	100.00%	3	5	25	0.01770%	56.40%
Tumor tissue	Protein IGHV5-51 (Fragment) GN=IGHV5-51 PE=1 SV=1	A0A0C4DH38_HUMAN	12,674.90	100.00%	2	2	4	0.00306%	37.60%
Pap test	Protein IGKV1-8 (Fragment) GN=IGKV1-8 PE=1 SV=1	A0A0C4DH67_HUMAN	12,537.90	99.20%	1	2	3	0.00468%	13.90%
Swab	Protein IGKV1-8 (Fragment) GN=IGKV1-8 PE=1 SV=1	A0A0C4DH67_HUMAN	12,537.90	100.00%	2	2	2	0.00142%	27.00%
Pap test	Protein IGKV2D-29 GN=IGKV2D-29 PE=1 SV=1	A0A087X0P6_HUMAN	11,191.80	99.80%	1	1	14	0.02180%	39.20%
Swab	Protein IGKV2D-29 GN=IGKV2D-29 PE=1 SV=1	A0A087X0P6_HUMAN	11,191.80	99.80%	1	1	12	0.00850%	39.20%
Pap test	Protein IGKV3-7 (Fragment) GN=IGKV3-7 PE=1 SV=1	A0A075B6H7_HUMAN	12,784.00	100.00%	2	3	10	0.01560%	23.30%
Swab	Protein IGKV3-7 (Fragment) GN=IGKV3-7 PE=1 SV=1	A0A075B6H7_HUMAN	12,784.00	100.00%	2	4	29	0.02060%	23.30%
Pap test	Protein IGKV3D-15 (Fragment) GN=IGKV3D-15 PE=1 SV=6	A0A087WSY6_HUMAN	12,534.70	100.00%	2	2	9	0.01400%	26.10%
Swab	Protein IGKV3D-15 (Fragment) GN=IGKV3D-15 PE=1 SV=6	A0A087WSY6_HUMAN	12,534.70	100.00%	2	4	11	0.00780%	26.10%

Biological sample	Protein name	Protein accession numbers	Protein molecular weight (Da)	Protein identification probability	Exclusive unique peptide count	Exclusive unique spectrum count	Total spectrum count	Percentage of total spectra	Percentage sequence coverage
Swab	Protein IGLV7-46 (Fragment) GN=IGLV7-46 PE=1 SV=4	A0A075B6I9_HUMAN	12,468.20	99.70%	1	1	4	0.00283%	15.40%
Pap test	Protein IGLV8-61 (Fragment) GN=IGLV8-61 PE=1 SV=7	A0A075B6I0_HUMAN	12,814.50	100.00%	2	2	5	0.00780%	14.80%
Swab	Protein IGLV8-61 (Fragment) GN=IGLV8-61 PE=1 SV=7	A0A075B6I0_HUMAN	12,814.50	100.00%	2	3	8	0.00567%	14.80%
Pap test	Protein IGLV9-49 GN=IGLV9-49 PE=1 SV=1	A0A0B4J1Y8_HUMAN	13,024.00	100.00%	1	1	2	0.00312%	8.13%
Swab	Protein IGLV9-49 GN=IGLV9-49 PE=1 SV=1	A0A0B4J1Y8_HUMAN	13,024.00	100.00%	2	2	3	0.00213%	16.30%
Tumor tissue	Protein IWS1 homolog GN=IWS1 PE=1 SV=2	sp|Q96ST2|IWS1_HUMAN	91,956.10	99.90%	2	2	2	0.00153%	4.03%
Tumor tissue	Protein jagunal homolog 1 GN=JAGN1 PE=1 SV=1	JAGN1_HUMAN	21,126.00	100.00%	2	2	2	0.00153%	7.10%
Tumor tissue	Protein kinase C delta-binding protein GN=PRKCDBP PE=1 SV=3	PRDBP_HUMAN	27,700.90	100.00%	5	7	8	0.00612%	20.70%
Tumor tissue	Protein kinase C iota type GN=PRKCI PE=1 SV=2	KPCI_HUMAN	68,263.20	100.00%	3	4	5	0.00383%	8.72%
Tumor tissue	Protein lin-7 homolog C GN=LIN7C PE=1 SV=1	LIN7C_HUMAN	21,833.60	100.00%	4	5	7	0.00536%	34.50%
Tumor tissue	Protein LOC102724159 GN=LOC102724159 PE=1 SV=1	A0A0B4J2E5_HUMAN	102,453.00	99.90%	1	1	2	0.00153%	3.16%
Tumor tissue	Protein LYRIC GN=MTDH PE=1 SV=2	LYRIC_HUMAN	63,838.40	100.00%	2	4	25	0.01910%	36.90%
Pap test	Protein mago nashi homolog 2 GN=MAGOHB PE=1 SV=1	MGN2_HUMAN	17,276.60	99.20%	1	1	1	0.00156%	13.50%
Swab	Protein mago nashi homolog 2 GN=MAGOHB PE=1 SV=1	sp|P61326|MGN_HUMAN	17,276.60	99.60%	1	1	1	0.00071%	13.50%
Tumor tissue	Protein mago nashi homolog 2 GN=MAGOHB PE=1 SV=1	sp|P61326|MGN_HUMAN	17,276.60	100.00%	4	4	9	0.00689%	31.80%
Tumor tissue	Protein MRVI1 GN=MRVI1 PE=1 SV=1	sp|Q9Y6F6|MRVI1_HUMAN	98,874.70	100.00%	3	3	3	0.00230%	4.99%
Swab	Protein NDRG1 GN=NDRG1 PE=1 SV=1	sp|Q92597|NDRG1_HUMAN	42,834.90	100.00%	3	5	6	0.00425%	11.40%
Tumor tissue	Protein NDRG1 GN=NDRG1 PE=1 SV=1	sp|Q92597|NDRG1_HUMAN	42,834.90	100.00%	6	20	29	0.02220%	40.40%

Biological sample	Protein name	Protein accession numbers	Protein molecular weight (Da)	Protein identification probability	Exclusive unique peptide count	Exclusive unique spectrum count	Total spectrum count	Percentage of total spectra	Percentage sequence coverage
Swab	Protein NDRG2 GN=NDRG2 PE=1 SV=1	sp|Q9UN36|NDRG2_HUMAN	39,545.20	100.00%	1	2	5	0.00354%	16.90%
Tumor tissue	Protein NDRG2 GN=NDRG2 PE=1 SV=1	sp|Q9UN36|NDRG2_HUMAN	39,545.20	100.00%	2	2	2	0.00153%	7.02%
Tumor tissue	Protein NEDD8-MDP1 (Fragment) GN=NEDD8-MDP1 PE=4 SV=1	E9PL57_HUMAN	19,536.90	98.60%	1	1	6	0.00459%	15.90%
Swab	Protein Niban GN=FAM129A PE=1 SV=1	NIBAN_HUMAN	103,134.40	100.00%	1	1	1	0.00071%	1.51%
Tumor tissue	Protein Niban GN=FAM129A PE=1 SV=1	NIBAN_HUMAN	103,134.40	100.00%	5	6	6	0.00459%	6.47%
Tumor tissue	Protein NipSnap homolog 1 GN=NIPSNAP1 PE=1 SV=1	NIPS1_HUMAN	33,310.90	100.00%	4	5	7	0.00536%	20.10%
Tumor tissue	Protein NipSnap homolog 2 GN=GBAS PE=1 SV=1	sp|O75323|NIPS2_HUMAN	33,743.50	100.00%	2	2	2	0.00153%	8.39%
Pap test	Protein NipSnap homolog 3A GN=NIPSNAP3A PE=1 SV=2	NPS3A_HUMAN	28,466.90	100.00%	2	2	2	0.00312%	9.72%
Tumor tissue	Protein NipSnap homolog 3A GN=NIPSNAP3A PE=1 SV=2	NPS3A_HUMAN	28,466.90	100.00%	3	5	8	0.00612%	19.80%
Tumor tissue	Protein NOXP20 GN=FAM114A1 PE=1 SV=2	sp|Q8IWE2|NXP20_HUMAN	60,741.70	100.00%	7	7	9	0.00689%	18.50%
Swab	Protein O-GlcNAcase GN=MGEA5 PE=1 SV=2	sp|O60502|OGA_HUMAN	102,917.50	99.90%	1	1	1	0.00071%	1.31%
Tumor tissue	Protein O-GlcNAcase GN=MGEA5 PE=1 SV=2	sp|O60502|OGA_HUMAN	102,917.50	100.00%	4	4	4	0.00306%	6.11%
Tumor tissue	Protein O-glucosyltransferase 1 GN=POGLUT1 PE=1 SV=1	PGLT1_HUMAN	46,190.30	100.00%	2	2	2	0.00153%	6.63%
Tumor tissue	Protein PBDC1 GN=PBDC1 PE=1 SV=1	PBDC1_HUMAN	26,057.50	100.00%	1	1	3	0.00230%	12.90%
Tumor tissue	Protein phosphatase 1 regulatory subunit GN=PPP1R12B PE=1 SV=1	sp|O60237|MYPT2_HUMAN	116,506.30	100.00%	2	2	2	0.00153%	2.68%
Tumor tissue	Protein phosphatase 1 regulatory subunit 12A GN=PPP1R12A PE=1 SV=1	sp|O14974|MYPT1_HUMAN	115,283.40	100.00%	1	1	16	0.01220%	19.50%
Tumor tissue	Protein phosphatase 1 regulatory subunit 12C (Fragment) GN=PPP1R12C PE=1 SV=1	sp|Q9BZL4|PP12C_HUMAN	84,683.50	100.00%	1	1	4	0.00306%	10.60%

Biological sample	Protein name	Protein accession numbers	Protein molecular weight (Da)	Protein identification probability	Exclusive unique peptide count	Exclusive unique spectrum count	Total spectrum count	Percentage of total spectra	Percentage sequence coverage
Tumor tissue	Protein phosphatase 1 regulatory subunit 21 GN=PPP1R21 PE=1 SV=1	sp|Q6ZMI0|PPR21_HUMAN	88,317.50	100.00%	3	4	4	0.00306%	7.56%
Pap test	Protein phosphatase 1 regulatory subunit 7 GN=PPP1R7 PE=1 SV=1	sp|Q15435|PP1R7_HUMAN	41,566.30	99.30%	1	1	1	0.00156%	3.06%
Swab	Protein phosphatase 1 regulatory subunit 7 GN=PPP1R7 PE=1 SV=1	sp|Q15435|PP1R7_HUMAN	41,566.30	100.00%	2	2	3	0.00213%	6.39%
Tumor tissue	Protein phosphatase 1 regulatory subunit 7 GN=PPP1R7 PE=1 SV=1	sp|Q15435|PP1R7_HUMAN	41,566.30	100.00%	8	11	12	0.00919%	31.40%
Tumor tissue	Protein phosphatase 1G GN=PPM1G PE=1 SV=1	PPM1G_HUMAN	59,271.90	100.00%	5	6	6	0.00459%	13.00%
Tumor tissue	Protein PRRC1 GN=PRRC1 PE=1 SV=1	sp|Q96M27|PRRC1_HUMAN	46,701.00	100.00%	4	5	8	0.00612%	12.10%
Tumor tissue	Protein PRRC2C GN=PRRC2C PE=1 SV=1	sp|Q9Y520|PRC2C_HUMAN	308,774.10	100.00%	1	1	8	0.00612%	4.22%
Pap test	Protein RCC2 GN=RCC2 PE=1 SV=2	RCC2_HUMAN	56,084.80	99.90%	1	1	1	0.00156%	1.92%
Tumor tissue	Protein RCC2 GN=RCC2 PE=1 SV=2	RCC2_HUMAN	56,084.80	100.00%	6	6	8	0.00612%	14.80%
Tumor tissue	Protein Red GN=IK PE=1 SV=3	RED_HUMAN	65,605.10	100.00%	4	4	5	0.00383%	13.80%
Tumor tissue	Protein RER1 GN=RER1 PE=1 SV=1	RER1_HUMAN	22,958.80	99.90%	1	1	3	0.00230%	18.40%
Tumor tissue	Protein RRP5 homolog GN=PDCD11 PE=1 SV=3	RRP5_HUMAN	208,702.80	100.00%	7	7	7	0.00536%	5.67%
Tumor tissue	Protein S100 (Fragment) GN=S100A6 PE=1 SV=1	S10A6_HUMAN	9,681.80	100.00%	3	3	3	0.00230%	35.30%
Pap test	Protein S100-A10 GN=S100A10 PE=1 SV=2	S10AA_HUMAN	11,203.50	100.00%	2	4	7	0.01090%	17.50%
Tumor tissue	Protein S100-A10 GN=S100A10 PE=1 SV=2	S10AA_HUMAN	11,203.50	100.00%	3	5	9	0.00689%	27.80%
Pap test	Protein S100-A11 GN=S100A11 PE=1 SV=2	S10AB_HUMAN	11,741.10	100.00%	4	6	15	0.02340%	42.90%
Swab	Protein S100-A11 GN=S100A11 PE=1 SV=2	S10AB_HUMAN	11,741.10	100.00%	5	7	22	0.01560%	56.20%
Tumor tissue	Protein S100-A11 GN=S100A11 PE=1 SV=2	S10AB_HUMAN	11,741.10	100.00%	4	8	29	0.02220%	45.70%

Biological sample	Protein name	Protein accession numbers	Protein molecular weight (Da)	Protein identification probability	Exclusive unique peptide count	Exclusive unique spectrum count	Total spectrum count	Percentage of total spectra	Percentage sequence coverage
Swab	Protein S100-A12 GN=S100A12 PE=1 SV=2	S10AC_HUMAN	10,575.40	100.00%	2	2	2	0.00142%	9.78%
Pap test	Protein S100-A13 GN=S100A13 PE=1 SV=1	S10AD_HUMAN	11,471.70	98.10%	1	1	1	0.00156%	11.20%
Tumor tissue	Protein S100-A13 GN=S100A13 PE=1 SV=1	S10AD_HUMAN	11,471.70	100.00%	5	5	5	0.00383%	39.80%
Pap test	Protein S100-A14 GN=S100A14 PE=1 SV=1	S10AE_HUMAN	11,662.20	100.00%	5	6	11	0.01720%	59.60%
Tumor tissue	Protein S100-A14 GN=S100A14 PE=1 SV=1	S10AE_HUMAN	11,662.20	100.00%	2	2	2	0.00153%	23.10%
Pap test	Protein S100-A16 GN=S100A16 PE=1 SV=1	S10AG_HUMAN	11,801.90	100.00%	3	6	8	0.01250%	34.00%
Tumor tissue	Protein S100-A16 GN=S100A16 PE=1 SV=1	S10AG_HUMAN	11,801.90	100.00%	6	9	11	0.00842%	64.10%
Pap test	Protein S100-A4 GN=S100A4 PE=1 SV=1	S10A4_HUMAN	11,729.00	100.00%	2	3	4	0.00624%	19.80%
Swab	Protein S100-A4 GN=S100A4 PE=1 SV=1	S10A4_HUMAN	11,729.00	100.00%	2	4	7	0.00496%	19.80%
Tumor tissue	Protein S100-A4 GN=S100A4 PE=1 SV=1	S10A4_HUMAN	11,729.00	100.00%	5	8	9	0.00689%	37.60%
Pap test	Protein S100-A7 GN=S100A7 PE=1 SV=4	S10A7_HUMAN	11,471.70	100.00%	4	7	11	0.01720%	34.70%
Swab	Protein S100-A7 GN=S100A7 PE=1 SV=4	S10A7_HUMAN	11,471.70	100.00%	1	1	2	0.00142%	12.90%
Pap test	Protein S100-A8 GN=S100A8 PE=1 SV=1	S10A8_HUMAN	10,835.00	100.00%	6	13	87	0.13600%	43.00%
Swab	Protein S100-A8 GN=S100A8 PE=1 SV=1	S10A8_HUMAN	10,835.00	100.00%	7	14	75	0.05310%	47.30%
Tumor tissue	Protein S100-A8 GN=S100A8 PE=1 SV=1	S10A8_HUMAN	10,835.00	100.00%	2	2	2	0.00153%	20.40%
Pap test	Protein S100-A9 GN=S100A9 PE=1 SV=1	S10A9_HUMAN	13,242.30	100.00%	6	21	179	0.27900%	70.20%
Swab	Protein S100-A9 GN=S100A9 PE=1 SV=1	S10A9_HUMAN	13,242.30	100.00%	6	23	142	0.10100%	70.20%
Tumor tissue	Protein S100-A9 GN=S100A9 PE=1 SV=1	S10A9_HUMAN	13,242.30	100.00%	4	7	16	0.01220%	44.70%

Biological sample	Protein name	Protein accession numbers	Protein molecular weight (Da)	Protein identification probability	Exclusive unique peptide count	Exclusive unique spectrum count	Total spectrum count	Percentage of total spectra	Percentage sequence coverage
Pap test	Protein S100-P GN=S100P PE=1 SV=2	S100P_HUMAN	10,400.40	100.00%	2	2	4	0.00624%	24.20%
Swab	Protein S100-P GN=S100P PE=1 SV=2	S100P_HUMAN	10,400.40	100.00%	2	2	7	0.00496%	24.20%
Tumor tissue	Protein S100-P GN=S100P PE=1 SV=2	S100P_HUMAN	10,400.40	99.90%	1	1	1	0.00077%	13.70%
Tumor tissue	Protein SAA2-SAA4 GN=SAA2-SAA4 PE=4 SV=1	A0A096LPE2_HUMAN	23,354.30	100.00%	2	2	2	0.00153%	12.50%
Tumor tissue	Protein SCO1 homolog, mitochondrial GN=SCO1 PE=1 SV=1	SCO1_HUMAN	30,180.10	100.00%	2	2	2	0.00153%	13.30%
Tumor tissue	Protein scribble homolog GN=SCRIB PE=1 SV=1	sp|Q14160|SCRIB_HUMAN	174,917.30	100.00%	17	20	31	0.02370%	19.50%
Pap test	Protein SEC13 homolog GN=SEC13 PE=1 SV=1	sp|P55735|SEC13_HUMAN	35,856.00	99.20%	1	1	1	0.00156%	3.38%
Swab	Protein SEC13 homolog GN=SEC13 PE=1 SV=1	sp|P55735|SEC13_HUMAN	35,856.00	100.00%	2	2	2	0.00142%	8.00%
Tumor tissue	Protein sel-1 homolog 1 GN=SEL1L PE=1 SV=3	sp|Q9UBV2|SE1L1_HUMAN	88,756.50	100.00%	2	2	2	0.00153%	4.79%
Tumor tissue	Protein SELENOH GN=SELENOH PE=1 SV=1	SELH_HUMAN	13,303.40	100.00%	4	4	4	0.00306%	36.40%
Pap test	Protein SELENOP (Fragment) GN=SELENOP PE=1 SV=1	SEPP1_HUMAN	35,115.80	100.00%	2	2	2	0.00312%	5.81%
Swab	Protein SELENOP (Fragment) GN=SELENOP PE=1 SV=1	SEPP1_HUMAN	35,115.80	100.00%	2	2	3	0.00213%	5.81%
Tumor tissue	Protein SET GN=SET PE=1 SV=3	sp|Q01105|SET_HUMAN	33,489.40	100.00%	1	2	11	0.00842%	37.60%
Tumor tissue	Protein syndesmos (Fragment) GN=NUDT16L1 PE=1 SV=1	K7EIN2_HUMAN	21,920.80	99.10%	1	1	2	0.00153%	14.60%
Tumor tissue	Protein THEMIS2 GN=THEMIS2 PE=1 SV=1	sp|Q5TEJ8|THMS2_HUMAN	72,048.90	100.00%	1	1	3	0.00230%	5.91%
Tumor tissue	Protein transport protein Sec16A GN=SEC16A PE=1 SV=1	F1T0I1_HUMAN	249,479.50	100.00%	9	11	12	0.00919%	5.36%
Tumor tissue	Protein transport protein Sec23A GN=SEC23A PE=1 SV=1	F5H365_HUMAN	82,970.90	100.00%	11	15	27	0.02070%	22.70%
Tumor tissue	Protein transport protein Sec23B GN=SEC23B PE=1 SV=2	SC23B_HUMAN	86,481.10	100.00%	6	9	13	0.00995%	13.40%

Biological sample	Protein name	Protein accession numbers	Protein molecular weight (Da)	Protein identification probability	Exclusive unique peptide count	Exclusive unique spectrum count	Total spectrum count	Percentage of total spectra	Percentage sequence coverage
Tumor tissue	Protein transport protein Sec24A GN=SEC24A PE=1 SV=2	sp|O95486|SC24A_HUMAN	119,751.90	100.00%	10	11	20	0.01530%	16.50%
Tumor tissue	Protein transport protein Sec24C GN=SEC24C PE=1 SV=3	sp|P53992|SC24C_HUMAN	118,325.20	100.00%	10	12	15	0.01150%	13.70%
Swab	Protein transport protein Sec31A GN=SEC31A PE=1 SV=1	sp|O94979|SC31A_HUMAN	136,228.20	100.00%	1	1	2	0.00142%	1.76%
Tumor tissue	Protein transport protein Sec31A GN=SEC31A PE=1 SV=1	sp|O94979|SC31A_HUMAN	136,228.20	100.00%	10	21	34	0.02600%	14.60%
Tumor tissue	Protein transport protein Sec61 subunit alpha isoform 1 GN=SEC61A1 PE=1 SV=1	sp|P61619|S61A1_HUMAN	52,951.90	100.00%	2	2	2	0.00153%	4.36%
Tumor tissue	Protein transport protein Sec61 subunit beta GN=SEC61B PE=1 SV=2	SC61B_HUMAN	9,974.40	100.00%	2	2	3	0.00230%	21.90%
Tumor tissue	Protein transport protein Sec61 subunit gamma GN=SEC61G PE=1 SV=1	SC61G_HUMAN	7,741.60	100.00%	2	2	5	0.00383%	36.80%
Tumor tissue	Protein tyrosine phosphatase type IVA 1 GN=PTP4A1 PE=1 SV=2	TP4A1_HUMAN	19,815.30	100.00%	2	2	2	0.00153%	16.80%
Pap test	Protein tyrosine phosphatase, receptor type, C, isoform CRA_d GN=PTPRC PE=1 SV=1	sp|P08575|PTPRC_HUMAN	147,488.20	100.00%	3	3	3	0.00468%	2.83%
Swab	Protein tyrosine phosphatase, receptor type, C, isoform CRA_d GN=PTPRC PE=1 SV=1	sp|P08575|PTPRC_HUMAN	147,488.20	100.00%	1	1	1	0.00071%	1.15%
Tumor tissue	Protein tyrosine phosphatase, receptor type, C, isoform CRA_d GN=PTPRC PE=1 SV=1	sp|P08575|PTPRC_HUMAN	147,488.20	100.00%	7	8	9	0.00689%	5.13%
Tumor tissue	Protein VAC14 homolog GN=VAC14 PE=1 SV=1	sp|Q08AM6|VAC14_HUMAN	87,976.60	100.00%	4	5	6	0.00459%	10.10%
Pap test	Protein-arginine deiminase type-1 GN=PADI1 PE=1 SV=2	PADI1_HUMAN	74,666.30	100.00%	2	2	2	0.00312%	3.32%
Swab	Protein-glutamine gamma-glutamyltransferase 2 GN=TGM2 PE=1 SV=2	sp|P21980|TGM2_HUMAN	77,329.10	100.00%	8	9	12	0.00850%	15.10%

Biological sample	Protein name	Protein accession numbers	Protein molecular weight (Da)	Protein identification probability	Exclusive unique peptide count	Exclusive unique spectrum count	Total spectrum count	Percentage of total spectra	Percentage sequence coverage
Tumor tissue	Protein-glutamine gamma-glutamyltransferase 2 GN=TGM2 PE=1 SV=2	sp|P21980|TGM2_HUMAN	77,329.10	100.00%	12	20	36	0.02760%	24.20%
Pap test	Protein-glutamine gamma-glutamyltransferase K GN=TGM1 PE=1 SV=4	sp|P22735|TGM1_HUMAN	89,786.00	100.00%	5	5	6	0.00936%	7.22%
Tumor tissue	Protein-glutamine gamma-glutamyltransferase K GN=TGM1 PE=1 SV=4	sp|P22735|TGM1_HUMAN	89,786.00	99.90%	1	1	1	0.00077%	1.59%
Swab	Protein-L-isoaspartate O-methyltransferase GN=PCMT1 PE=1 SV=1	sp|P22061-2|PIMT_HUMAN	30,358.20	100.00%	4	4	5	0.00354%	14.30%
Tumor tissue	Protein-L-isoaspartate O-methyltransferase GN=PCMT1 PE=1 SV=1	sp|P22061-2|PIMT_HUMAN	30,358.20	100.00%	7	10	15	0.01150%	31.10%
Pap test	Prothrombin GN=F2 PE=1 SV=2	THRB_HUMAN	70,036.80	100.00%	9	11	17	0.02650%	22.70%
Swab	Prothrombin GN=F2 PE=1 SV=2	THRB_HUMAN	70,036.80	100.00%	16	23	33	0.02340%	37.10%
Tumor tissue	Prothrombin GN=F2 PE=1 SV=2	THRB_HUMAN	70,036.80	100.00%	5	5	5	0.00383%	8.84%
Tumor tissue	Protocadherin-16 GN=DCHS1 PE=1 SV=1	PCD16_HUMAN	346,178.80	100.00%	2	2	2	0.00153%	1.79%
Pap test	Pterin-4-alpha-carbinolamine dehydratase GN=PCBD1 PE=1 SV=2	PHS_HUMAN	11,999.70	99.90%	1	1	1	0.00156%	9.62%
Swab	Pterin-4-alpha-carbinolamine dehydratase GN=PCBD1 PE=1 SV=2	PHS_HUMAN	11,999.70	99.20%	1	1	2	0.00142%	9.62%
Tumor tissue	Pterin-4-alpha-carbinolamine dehydratase GN=PCBD1 PE=1 SV=2	PHS_HUMAN	11,999.70	100.00%	2	2	2	0.00153%	22.10%
Pap test	Purine nucleoside phosphorylase GN=PNP PE=1 SV=2	PNPH_HUMAN	32,118.00	100.00%	5	5	5	0.00780%	21.80%
Swab	Purine nucleoside phosphorylase GN=PNP PE=1 SV=2	PNPH_HUMAN	32,118.00	100.00%	7	8	14	0.00992%	32.20%
Tumor tissue	Purine nucleoside phosphorylase GN=PNP PE=1 SV=2	PNPH_HUMAN	32,118.00	100.00%	9	12	16	0.01220%	37.70%
Tumor tissue	Purkinje cell protein 4 GN=PCP4 PE=1 SV=3	PCP4_HUMAN	6,791.40	100.00%	3	3	3	0.00230%	43.50%

Biological sample	Protein name	Protein accession numbers	Protein molecular weight (Da)	Protein identification probability	Exclusive unique peptide count	Exclusive unique spectrum count	Total spectrum count	Percentage of total spectra	Percentage sequence coverage
Pap test	Puromycin-sensitive aminopeptidase GN=NPEPPS PE=1 SV=1	sp|P55786|PSA_HUMAN	102,990.30	100.00%	7	7	9	0.01400%	8.85%
Swab	Puromycin-sensitive aminopeptidase GN=NPEPPS PE=1 SV=1	sp|P55786|PSA_HUMAN	102,990.30	100.00%	12	14	25	0.01770%	16.80%
Tumor tissue	Puromycin-sensitive aminopeptidase GN=NPEPPS PE=1 SV=1	sp|P55786|PSA_HUMAN	102,990.30	100.00%	15	18	21	0.01610%	19.30%
Tumor tissue	Putative ATP-dependent RNA helicase DHX30 GN=DHX30 PE=1 SV=1	sp|Q7L2E3|DHX30_HUMAN	136,118.80	100.00%	6	6	6	0.00459%	7.55%
Tumor tissue	Putative deoxyribonuclease TATDN1 (Fragment) GN=TATDN1 PE=1 SV=1	E5RG17_HUMAN	36,420.00	100.00%	1	1	2	0.00153%	7.14%
Pap test	Putative elongation factor 1-alpha-like 3 GN=EEF1A1P5 PE=5 SV=1	sp|P68104|EF1A1_HUMAN	50,141.20	100.00%	3	5	17	0.02650%	22.90%
Swab	Putative elongation factor 1-alpha-like 3 GN=EEF1A1P5 PE=5 SV=1	sp|P68104|EF1A1_HUMAN	50,185.40	100.00%	2	3	13	0.00921%	17.50%
Tumor tissue	Putative elongation factor 1-alpha-like 3 GN=EEF1A1P5 PE=5 SV=1	sp|P68104|EF1A1_HUMAN	50,185.40	100.00%	4	11	119	0.09110%	27.30%
Tumor tissue	Putative oxidoreductase GLYR1 (Fragment) GN=GLYR1 PE=1 SV=2	sp|Q49A26|GLYR1_HUMAN	57,327.50	100.00%	4	4	5	0.00383%	13.00%
Tumor tissue	Putative peptidyl-tRNA hydrolase PTRHD1 GN=PTRHD1 PE=1 SV=1	PTRD1_HUMAN	15,805.40	100.00%	2	2	2	0.00153%	18.60%
Tumor tissue	Putative pre-mRNA-splicing factor ATP-dependent RNA helicase DHX16 GN=DHX16 PE=1 SV=2	DHX16_HUMAN	119,265.80	100.00%	3	3	3	0.00230%	4.23%
Tumor tissue	Putative RNA-binding protein 15 GN=RBM15 PE=1 SV=1	sp|Q96T37|RBM15_HUMAN	102,139.10	100.00%	2	2	2	0.00153%	3.32%
Tumor tissue	Putative RNA-binding protein Luc7-like 2 GN=LUC7L2 PE=1 SV=2	sp|Q9Y383|LC7L2_HUMAN	46,514.50	100.00%	1	1	8	0.00612%	17.30%
Tumor tissue	Putative small nuclear ribonucleoprotein G-like protein 15 GN=SNRPGP15 PE=5 SV=2	RUXGL_HUMAN	8,496.30	100.00%	2	3	6	0.00459%	17.10%

Biological sample	Protein name	Protein accession numbers	Protein molecular weight (Da)	Protein identification probability	Exclusive unique peptide count	Exclusive unique spectrum count	Total spectrum count	Percentage of total spectra	Percentage sequence coverage
Tumor tissue	Putative transferase CAF17, mitochondrial GN=IBA57 PE=1 SV=1	CAF17_HUMAN	38,155.50	100.00%	2	2	2	0.00153%	11.50%
Pap test	Pyridoxal kinase GN=PDXK PE=1 SV=1	sp|O00764|PDXK_HUMAN	35,101.70	100.00%	9	13	20	0.03120%	32.40%
Swab	Pyridoxal kinase GN=PDXK PE=1 SV=1	sp|O00764|PDXK_HUMAN	35,101.70	100.00%	7	9	11	0.00780%	20.80%
Tumor tissue	Pyridoxal kinase GN=PDXK PE=1 SV=1	sp|O00764|PDXK_HUMAN	35,101.70	100.00%	6	10	10	0.00766%	23.70%
Tumor tissue	Pyrroline-5-carboxylate reductase GN=PYCRL PE=1 SV=1	sp|Q53H96|P5CR3_HUMAN	29,891.80	100.00%	3	5	6	0.00459%	12.20%
Tumor tissue	Pyruvate dehydrogenase phosphatase regulatory subunit, mitochondrial GN=PDPR PE=1 SV=2	sp|Q8NCN5|PDPR_HUMAN	99,366.70	100.00%	3	3	5	0.00383%	7.62%
Tumor tissue	Pyruvate dehydrogenase protein X component, mitochondrial GN=PDHX PE=1 SV=3	sp|O00330|ODPX_HUMAN	54,123.80	100.00%	2	2	2	0.00153%	4.99%
Pap test	Pyruvate kinase PKM GN=PKM PE=1 SV=4	sp|P14618|KPYM_HUMAN	57,937.50	100.00%	3	4	65	0.10100%	53.50%
Swab	Pyruvate kinase PKM GN=PKM PE=1 SV=4	sp|P14618|KPYM_HUMAN	57,937.50	100.00%	5	9	205	0.14500%	63.30%
Tumor tissue	Pyruvate kinase PKM GN=PKM PE=1 SV=4	sp|P14618|KPYM_HUMAN	57,937.50	100.00%	4	8	148	0.11300%	49.90%
Swab	Quinone oxidoreductase GN=CRYZ PE=1 SV=1	sp|Q08257|QOR_HUMAN	35,207.30	100.00%	3	4	4	0.00283%	13.10%
Tumor tissue	Quinone oxidoreductase GN=CRYZ PE=1 SV=1	sp|Q08257|QOR_HUMAN	35,207.30	100.00%	8	11	11	0.00842%	37.70%
Pap test	Quinone oxidoreductase PIG3 GN=TP53I3 PE=1 SV=2	sp|Q53FA7|QORX_HUMAN	35,537.30	100.00%	5	6	16	0.02500%	31.00%
Tumor tissue	Quinone oxidoreductase PIG3 GN=TP53I3 PE=1 SV=2	sp|Q53FA7|QORX_HUMAN	35,537.30	100.00%	3	4	6	0.00459%	22.00%
Tumor tissue	Quinone oxidoreductase PIG3 (Fragment) GN=TP53I3 PE=1 SV=1	H7BZH6_HUMAN	22,654.60	99.60%	1	1	3	0.00230%	24.10%
Pap test	Rab GDP dissociation inhibitor alpha GN=GDI1 PE=1 SV=2	GDIA_HUMAN	50,584.10	100.00%	2	2	3	0.00468%	8.05%

Biological sample	Protein name	Protein accession numbers	Protein molecular weight (Da)	Protein identification probability	Exclusive unique peptide count	Exclusive unique spectrum count	Total spectrum count	Percentage of total spectra	Percentage sequence coverage
Swab	Rab GDP dissociation inhibitor alpha GN=GDI1 PE=1 SV=2	GDIA_HUMAN	50,584.10	100.00%	6	6	19	0.01350%	26.40%
Tumor tissue	Rab GDP dissociation inhibitor alpha GN=GDI1 PE=1 SV=2	GDIA_HUMAN	50,584.10	100.00%	8	8	25	0.01910%	38.50%
Pap test	Rab GDP dissociation inhibitor beta GN=GDI2 PE=1 SV=2	sp|P50395|GDIB_HUMAN	50,665.50	100.00%	5	5	8	0.01250%	20.40%
Swab	Rab GDP dissociation inhibitor beta GN=GDI2 PE=1 SV=2	sp|P50395|GDIB_HUMAN	50,665.50	100.00%	12	19	42	0.02980%	44.90%
Tumor tissue	Rab GDP dissociation inhibitor beta GN=GDI2 PE=1 SV=2	sp|P50395|GDIB_HUMAN	50,665.50	100.00%	13	22	45	0.03440%	48.30%
Tumor tissue	Rab-like protein 3 GN=RABL3 PE=1 SV=1	RABL3_HUMAN	26,422.90	99.90%	2	2	2	0.00153%	9.32%
Tumor tissue	RAC-alpha serine/threonine-protein kinase GN=AKT1 PE=1 SV=2	sp|P31749|AKT1_HUMAN	55,687.60	100.00%	3	3	4	0.00306%	12.10%
Tumor tissue	RAC-beta serine/threonine-protein kinase GN=AKT2 PE=1 SV=1	sp|P31751|AKT2_HUMAN	51,572.40	100.00%	1	1	4	0.00306%	8.89%
Tumor tissue	Raftlin GN=RFTN1 PE=1 SV=4	RFTN1_HUMAN	63,145.30	100.00%	6	6	6	0.00459%	19.00%
Tumor tissue	Ragulator complex protein LAMTOR1 GN=LAMTOR1 PE=1 SV=2	LTOR1_HUMAN	17,745.30	100.00%	5	6	7	0.00536%	45.30%
Swab	Ragulator complex protein LAMTOR3 GN=LAMTOR3 PE=1 SV=1	sp|Q9UHA4|LTOR3_HUMAN	13,623.00	98.70%	1	1	2	0.00142%	8.06%
Tumor tissue	Ragulator complex protein LAMTOR3 GN=LAMTOR3 PE=1 SV=1	sp|Q9UHA4|LTOR3_HUMAN	13,623.00	100.00%	3	5	5	0.00383%	43.50%
Swab	Ragulator complex protein LAMTOR5 GN=LAMTOR5 PE=1 SV=1	LTOR5_HUMAN	9,613.70	99.60%	1	1	1	0.00071%	22.00%
Tumor tissue	Ragulator complex protein LAMTOR5 GN=LAMTOR5 PE=1 SV=1	LTOR5_HUMAN	9,613.70	100.00%	2	2	2	0.00153%	37.40%
Swab	Ran GTPase-activating protein 1 GN=RANGAP1 PE=1 SV=1	RAGP1_HUMAN	63,543.30	99.60%	1	1	1	0.00071%	1.87%
Tumor tissue	Ran GTPase-activating protein 1 GN=RANGAP1 PE=1 SV=1	RAGP1_HUMAN	63,543.30	100.00%	9	9	9	0.00689%	17.00%
Swab	Ras GTPase-activating protein-binding protein 1 GN=G3BP1 PE=1 SV=1	sp|Q13283|G3BP1_HUMAN	52,162.80	99.10%	1	1	1	0.00071%	3.86%

Biological sample	Protein name	Protein accession numbers	Protein molecular weight (Da)	Protein identification probability	Exclusive unique peptide count	Exclusive unique spectrum count	Total spectrum count	Percentage of total spectra	Percentage sequence coverage
Tumor tissue	Ras GTPase-activating protein-binding protein 1 GN=G3BP1 PE=1 SV=1	sp|Q13283|G3BP1_HUMAN	52,162.80	100.00%	12	15	16	0.01220%	40.80%
Pap test	Ras GTPase-activating-like protein IQGAP1 GN=IQGAP1 PE=1 SV=1	IQGA1_HUMAN	189,258.60	100.00%	36	43	68	0.10600%	30.10%
Swab	Ras GTPase-activating-like protein IQGAP1 GN=IQGAP1 PE=1 SV=1	IQGA1_HUMAN	189,258.60	100.00%	35	40	64	0.04540%	31.00%
Tumor tissue	Ras GTPase-activating-like protein IQGAP1 GN=IQGAP1 PE=1 SV=1	IQGA1_HUMAN	189,258.60	100.00%	53	85	157	0.12000%	40.60%
Swab	Ras GTPase-activating-like protein IQGAP2 GN=IQGAP2 PE=1 SV=4	sp|Q13576|IQGA2_HUMAN	180,584.30	100.00%	1	1	1	0.00071%	0.76%
Tumor tissue	Ras GTPase-activating-like protein IQGAP2 GN=IQGAP2 PE=1 SV=4	sp|Q13576|IQGA2_HUMAN	180,584.30	100.00%	8	8	9	0.00689%	7.43%
Swab	Ras suppressor protein 1 GN=RSU1 PE=1 SV=3	sp|Q15404|RSU1_HUMAN	31,542.20	99.90%	1	1	2	0.00142%	6.14%
Tumor tissue	Ras suppressor protein 1 GN=RSU1 PE=1 SV=3	sp|Q15404|RSU1_HUMAN	31,542.20	100.00%	7	7	11	0.00842%	24.90%
Pap test	Ras-related C3 botulinum toxin substrate 2 GN=RAC2 PE=1 SV=1	RAC2_HUMAN	21,429.20	100.00%	2	2	6	0.00936%	22.90%
Swab	Ras-related C3 botulinum toxin substrate 2 GN=RAC2 PE=1 SV=1	RAC2_HUMAN	21,429.20	100.00%	2	2	9	0.00638%	29.70%
Tumor tissue	Ras-related C3 botulinum toxin substrate 2 GN=RAC2 PE=1 SV=1	RAC2_HUMAN	21,429.20	100.00%	2	3	4	0.00306%	12.00%
Swab	Ras-related protein Rab-10 GN=RAB10 PE=1 SV=1	RAB10_HUMAN	22,542.10	98.60%	1	1	10	0.00709%	16.50%
Tumor tissue	Ras-related protein Rab-10 GN=RAB10 PE=1 SV=1	RAB10_HUMAN	22,542.10	100.00%	2	2	16	0.01220%	21.50%
Pap test	Ras-related protein Rab-11B GN=RAB11B PE=1 SV=4	sp|Q15907|RB11B_HUMAN	24,489.00	100.00%	5	5	5	0.00780%	23.40%
Swab	Ras-related protein Rab-11B GN=RAB11B PE=1 SV=4	sp|Q15907|RB11B_HUMAN	24,489.00	100.00%	2	2	2	0.00142%	13.80%
Tumor tissue	Ras-related protein Rab-11B GN=RAB11B PE=1 SV=4	sp|Q15907|RB11B_HUMAN	24,489.00	100.00%	7	10	13	0.00995%	40.80%
Tumor tissue	Ras-related protein Rab-12 GN=RAB12 PE=1 SV=3	RAB12_HUMAN	27,249.80	99.50%	1	1	6	0.00459%	9.02%

Biological sample	Protein name	Protein accession numbers	Protein molecular weight (Da)	Protein identification probability	Exclusive unique peptide count	Exclusive unique spectrum count	Total spectrum count	Percentage of total spectra	Percentage sequence coverage
Tumor tissue	Ras-related protein Rab-13 GN=RAB13 PE=1 SV=1	RAB13_HUMAN	22,775.60	100.00%	2	2	7	0.00536%	17.20%
Pap test	Ras-related protein Rab-14 GN=RAB14 PE=1 SV=4	RAB14_HUMAN	23,897.60	100.00%	4	4	6	0.00936%	28.40%
Swab	Ras-related protein Rab-14 GN=RAB14 PE=1 SV=4	RAB14_HUMAN	23,897.60	100.00%	4	5	10	0.00709%	33.50%
Tumor tissue	Ras-related protein Rab-14 GN=RAB14 PE=1 SV=4	RAB14_HUMAN	23,897.60	100.00%	9	15	26	0.01990%	58.10%
Swab	Ras-related protein Rab-18 GN=RAB18 PE=1 SV=1	sp|Q9NP72|RAB18_HUMAN	26,410.60	100.00%	2	2	2	0.00142%	14.30%
Tumor tissue	Ras-related protein Rab-18 GN=RAB18 PE=1 SV=1	sp|Q9NP72|RAB18_HUMAN	20,420.40	100.00%	6	7	8	0.00612%	39.60%
Swab	Ras-related protein Rab-1A GN=RAB1A PE=1 SV=3	sp|P62820|RAB1A_HUMAN	22,678.50	100.00%	1	1	12	0.00850%	26.80%
Tumor tissue	Ras-related protein Rab-1A GN=RAB1A PE=1 SV=3	sp|P62820|RAB1A_HUMAN	22,678.50	100.00%	5	7	29	0.02220%	55.60%
Tumor tissue	Ras-related protein Rab-1B GN=RAB1B PE=1 SV=1	RAB1B_HUMAN	22,172.00	100.00%	3	4	29	0.02220%	58.70%
Pap test	Ras-related protein Rab-21 GN=RAB21 PE=1 SV=3	RAB21_HUMAN	24,347.90	100.00%	1	1	1	0.00156%	4.89%
Swab	Ras-related protein Rab-21 GN=RAB21 PE=1 SV=3	RAB21_HUMAN	24,347.90	99.20%	1	1	2	0.00142%	4.89%
Tumor tissue	Ras-related protein Rab-21 GN=RAB21 PE=1 SV=3	RAB21_HUMAN	24,347.90	100.00%	4	6	7	0.00536%	23.10%
Tumor tissue	Ras-related protein Rab-23 GN=RAB23 PE=1 SV=1	RAB23_HUMAN	26,659.50	100.00%	3	4	4	0.00306%	17.70%
Pap test	Ras-related protein Rab-25 GN=RAB25 PE=1 SV=2	RAB25_HUMAN	23,495.70	100.00%	2	2	2	0.00312%	10.30%
Pap test	Ras-related protein Rab-27A (Fragment) GN=RAB27A PE=1 SV=1	sp|P51159|RB27A_HUMAN	20,980.80	99.80%	1	1	1	0.00156%	5.95%
Tumor tissue	Ras-related protein Rab-27A (Fragment) GN=RAB27A PE=1 SV=1	sp|P51159|RB27A_HUMAN	20,980.80	100.00%	3	3	3	0.00230%	20.00%
Pap test	Ras-related protein Rab-27B GN=RAB27B PE=1 SV=4	RB27B_HUMAN	24,608.60	100.00%	2	3	3	0.00468%	10.10%
Pap test	Ras-related protein Rab-2A GN=RAB2A PE=1 SV=1	sp|P61019|RAB2A_HUMAN	23,546.20	99.90%	1	1	2	0.00312%	12.70%

Biological sample	Protein name	Protein accession numbers	Protein molecular weight (Da)	Protein identification probability	Exclusive unique peptide count	Exclusive unique spectrum count	Total spectrum count	Percentage of total spectra	Percentage sequence coverage
Swab	Ras-related protein Rab-2A GN=RAB2A PE=1 SV=1	sp|P61019|RAB2A_HUMAN	23,546.20	100.00%	1	1	2	0.00142%	12.70%
Tumor tissue	Ras-related protein Rab-2A GN=RAB2A PE=1 SV=1	sp|P61019|RAB2A_HUMAN	23,546.20	100.00%	3	6	24	0.01840%	61.30%
Tumor tissue	Ras-related protein Rab-31 GN=RAB31 PE=1 SV=1	RAB31_HUMAN	21,569.10	100.00%	4	7	8	0.00612%	27.30%
Tumor tissue	Ras-related protein Rab-35 (Fragment) GN=RAB35 PE=1 SV=1	sp|Q15286|RAB35_HUMAN	21,214.40	98.70%	1	2	12	0.00919%	20.00%
Tumor tissue	Ras-related protein Rab-3A GN=RAB3A PE=1 SV=1	RAB3A_HUMAN	24,984.30	100.00%	2	2	9	0.00689%	24.50%
Tumor tissue	Ras-related protein Rab-43 GN=RAB43 PE=1 SV=1	sp|Q86YS6|RAB43_HUMAN	23,339.90	100.00%	3	3	8	0.00612%	22.20%
Tumor tissue	Ras-related protein Rab-4A GN=RAB4A PE=1 SV=3	RAB4A_HUMAN	24,390.40	100.00%	4	4	9	0.00689%	28.00%
Tumor tissue	Ras-related protein Rab-4B (Fragment) GN=RAB4B PE=1 SV=1	sp|P61018|RAB4B_HUMAN	21,321.40	100.00%	2	2	7	0.00536%	18.80%
Tumor tissue	Ras-related protein Rab-5B GN=RAB5B PE=1 SV=1	sp|P61020|RAB5B_HUMAN	23,706.90	100.00%	3	7	12	0.00919%	31.60%
Pap test	Ras-related protein Rab-7a GN=RAB7A PE=1 SV=1	RAB7A_HUMAN	23,490.00	100.00%	2	2	5	0.00780%	21.30%
Swab	Ras-related protein Rab-7a GN=RAB7A PE=1 SV=1	RAB7A_HUMAN	23,490.00	100.00%	4	4	13	0.00921%	46.90%
Tumor tissue	Ras-related protein Rab-7a GN=RAB7A PE=1 SV=1	RAB7A_HUMAN	23,490.00	100.00%	3	4	28	0.02140%	58.90%
Tumor tissue	Ras-related protein Rab-8A GN=RAB8A PE=1 SV=1	sp|P61006|RAB8A_HUMAN	23,669.70	100.00%	1	2	16	0.01220%	27.10%
Swab	Ras-related protein Rab-8B (Fragment) GN=RAB8B PE=1 SV=1	RAB8B_HUMAN	21,869.80	99.60%	1	1	8	0.00567%	19.10%
Tumor tissue	Ras-related protein Rab-8B (Fragment) GN=RAB8B PE=1 SV=1	RAB8B_HUMAN	21,869.80	100.00%	2	3	15	0.01150%	29.80%
Tumor tissue	Ras-related protein Rab-9A GN=RAB9A PE=1 SV=1	RAB9A_HUMAN	22,837.80	100.00%	2	2	2	0.00153%	13.40%
Tumor tissue	Ras-related protein Ral-A GN=RALA PE=1 SV=1	RALA_HUMAN	23,567.20	100.00%	3	4	10	0.00766%	29.10%
Tumor tissue	Ras-related protein Rap-1A GN=RAP1A PE=1 SV=1	RAP1A_HUMAN	20,987.30	100.00%	1	2	31	0.02370%	35.90%

Biological sample	Protein name	Protein accession numbers	Protein molecular weight (Da)	Protein identification probability	Exclusive unique peptide count	Exclusive unique spectrum count	Total spectrum count	Percentage of total spectra	Percentage sequence coverage
Pap test	Ras-related protein Rap-2b GN=RAP2B PE=1 SV=1	RAP2B_HUMAN	20,504.40	100.00%	1	1	3	0.00468%	18.00%
Tumor tissue	Ras-related protein Rap-2b GN=RAP2B PE=1 SV=1	RAP2B_HUMAN	20,504.40	100.00%	4	4	8	0.00612%	39.30%
Tumor tissue	Ras-related protein Rap-2c GN=RAP2C PE=1 SV=1	RAP2C_HUMAN	20,744.60	99.80%	1	1	3	0.00230%	19.70%
Tumor tissue	Ras-related protein R-Ras GN=RRAS PE=1 SV=1	RRAS_HUMAN	23,480.50	100.00%	2	3	8	0.00612%	27.10%
Tumor tissue	Receptor expression-enhancing protein 5 GN=REEP5 PE=1 SV=3	REEP5_HUMAN	21,494.50	100.00%	3	3	3	0.00230%	16.40%
Pap test	Receptor of activated protein C kinase 1 GN=RACK1 PE=1 SV=3	RACK1_HUMAN	35,077.00	100.00%	1	1	8	0.01250%	27.40%
Tumor tissue	Receptor of activated protein C kinase 1 GN=RACK1 PE=1 SV=3	RACK1_HUMAN	35,077.00	100.00%	1	1	18	0.01380%	27.40%
Pap test	Receptor protein-tyrosine kinase GN=EGFR PE=1 SV=3	sp|P00533|EGFR_HUMAN	129,170.60	100.00%	3	3	3	0.00468%	3.09%
Swab	Receptor protein-tyrosine kinase GN=EGFR PE=1 SV=3	sp|P00533|EGFR_HUMAN	134,279.20	99.70%	1	1	1	0.00071%	0.86%
Tumor tissue	Receptor protein-tyrosine kinase GN=EGFR PE=1 SV=3	sp|P00533|EGFR_HUMAN	129,170.60	99.90%	1	1	1	0.00077%	1.37%
Tumor tissue	Receptor-interacting serine/threonine-protein kinase 1 GN=RIPK1 PE=1 SV=3	sp|Q13546|RIPK1_HUMAN	75,932.40	100.00%	2	2	2	0.00153%	4.02%
Swab	Receptor-type tyrosine-protein phosphatase kappa GN=PTPRK PE=1 SV=1	sp|Q15262|PTPRK_HUMAN	166,030.00	100.00%	2	2	2	0.00142%	2.17%
Tumor tissue	Regulation of nuclear pre-mRNA domain-containing protein 1A GN=RPRD1A PE=1 SV=1	sp|Q96P16|RPR1A_HUMAN	35,720.50	100.00%	2	2	6	0.00459%	20.80%
Tumor tissue	Regulation of nuclear pre-mRNA domain-containing protein 1B GN=RPRD1B PE=1 SV=1	RPR1B_HUMAN	36,900.80	100.00%	5	7	12	0.00919%	31.60%
Tumor tissue	Regulation of nuclear pre-mRNA domain-containing protein 2 GN=RPRD2 PE=1 SV=1	sp|Q5VT52|RPRD2_HUMAN	156,023.00	100.00%	4	4	4	0.00306%	4.11%

Biological sample	Protein name	Protein accession numbers	Protein molecular weight (Da)	Protein identification probability	Exclusive unique peptide count	Exclusive unique spectrum count	Total spectrum count	Percentage of total spectra	Percentage sequence coverage
Tumor tissue	Regulator of G-protein signaling 19 GN=RGS19 PE=1 SV=1	RGS19_HUMAN	24,635.50	100.00%	2	2	2	0.00153%	9.68%
Tumor tissue	Regulator of microtubule dynamics protein 2 GN=RMDN2 PE=1 SV=2	sp|Q96LZ7|RMD2_HUMAN	47,401.60	100.00%	1	1	2	0.00153%	7.56%
Swab	Regulator of microtubule dynamics protein 3 GN=RMDN3 PE=1 SV=2	sp|Q96TC7|RMD3_HUMAN	52,119.40	100.00%	2	2	3	0.00213%	5.96%
Tumor tissue	Regulator of microtubule dynamics protein 3 GN=RMDN3 PE=1 SV=2	sp|Q96TC7|RMD3_HUMAN	52,119.40	100.00%	5	6	7	0.00536%	15.30%
Tumor tissue	Regulator of nonsense transcripts 2 GN=UPF2 PE=1 SV=1	sp|Q9HAU5|RENT2_HUMAN	147,814.50	100.00%	2	2	2	0.00153%	1.73%
Tumor tissue	RelA-associated inhibitor GN=PPP1R13L PE=1 SV=4	IASPP_HUMAN	89,093.10	100.00%	3	3	4	0.00306%	4.35%
Tumor tissue	Renin receptor (Fragment) GN=ATP6AP2 PE=1 SV=1	sp|O75787|RENR_HUMAN	28,418.00	100.00%	2	2	2	0.00153%	12.90%
Tumor tissue	Replication protein A 14 kDa subunit GN=RPA3 PE=1 SV=1	RFA3_HUMAN	9,170.60	100.00%	2	2	2	0.00153%	40.20%
Tumor tissue	Replication protein A 70 kDa DNA-binding subunit GN=RPA1 PE=1 SV=2	RFA1_HUMAN	68,139.00	100.00%	6	7	8	0.00612%	14.80%
Pap test	Resistin GN=RETN PE=1 SV=1	sp|Q9HD89|RETN_HUMAN	11,419.10	100.00%	2	2	7	0.01090%	40.70%
Swab	Resistin GN=RETN PE=1 SV=1	sp|Q9HD89|RETN_HUMAN	11,419.10	100.00%	2	2	8	0.00567%	35.20%
Tumor tissue	REST corepressor 1 GN=RCOR1 PE=1 SV=1	RCOR1_HUMAN	53,327.40	100.00%	3	3	3	0.00230%	9.07%
Pap test	Reticulocalbin-1 GN=RCN1 PE=1 SV=1	sp|Q15293|RCN1_HUMAN	38,891.20	98.90%	1	1	1	0.00156%	3.93%
Swab	Reticulocalbin-1 GN=RCN1 PE=1 SV=1	sp|Q15293|RCN1_HUMAN	38,891.20	100.00%	3	3	3	0.00213%	13.30%
Tumor tissue	Reticulocalbin-1 GN=RCN1 PE=1 SV=1	sp|Q15293|RCN1_HUMAN	38,891.20	100.00%	10	14	17	0.01300%	40.50%
Tumor tissue	Reticulocalbin-3 GN=RCN3 PE=1 SV=1	RCN3_HUMAN	37,492.90	100.00%	3	8	23	0.01760%	36.60%
Pap test	Retinal dehydrogenase 1 GN=ALDH1A1 PE=1 SV=2	AL1A1_HUMAN	54,862.80	100.00%	1	1	1	0.00156%	2.59%
Swab	Retinal dehydrogenase 1 GN=ALDH1A1 PE=1 SV=2	AL1A1_HUMAN	54,862.80	99.90%	1	1	1	0.00071%	2.00%

Biological sample	Protein name	Protein accession numbers	Protein molecular weight (Da)	Protein identification probability	Exclusive unique peptide count	Exclusive unique spectrum count	Total spectrum count	Percentage of total spectra	Percentage sequence coverage
Tumor tissue	Retinal dehydrogenase 1 GN=ALDH1A1 PE=1 SV=2	AL1A1_HUMAN	54,862.80	100.00%	12	17	19	0.01450%	31.50%
Tumor tissue	Retinal dehydrogenase 2 GN=ALDH1A2 PE=1 SV=3	sp|O94788|AL1A2_HUMAN	56,725.00	100.00%	6	7	8	0.00612%	16.60%
Tumor tissue	Retinal rod rhodopsin-sensitive cGMP 3',5'-cyclic phosphodiesterase subunit delta GN=PDE6D PE=1 SV=1	PDE6D_HUMAN	17,420.30	100.00%	2	2	2	0.00153%	16.00%
Pap test	Retinoic acid-induced protein 3 GN=GPRC5A PE=1 SV=2	RAI3_HUMAN	40,252.60	100.00%	2	3	4	0.00624%	8.68%
Tumor tissue	Retinol binding protein 1, cellular GN=RBP1 PE=1 SV=1	sp|P09455|RET1_HUMAN	22,310.30	100.00%	6	10	13	0.00995%	27.90%
Pap test	Retinol binding protein 4, plasma, isoform CRA_b GN=RBP4 PE=1 SV=2	RET4_HUMAN	22,974.00	100.00%	5	8	10	0.01560%	34.70%
Swab	Retinol binding protein 4, plasma, isoform CRA_b GN=RBP4 PE=1 SV=2	RET4_HUMAN	22,974.00	100.00%	7	12	35	0.02480%	53.30%
Tumor tissue	Retinol dehydrogenase 11 GN=RDH11 PE=1 SV=2	sp|Q8TC12|RDH11_HUMAN	35,386.50	100.00%	1	1	5	0.00383%	15.40%
Tumor tissue	Retinol dehydrogenase 13 GN=RDH13 PE=1 SV=2	sp|Q8NBN7|RDH13_HUMAN	35,933.20	99.80%	1	1	2	0.00153%	6.95%
Tumor tissue	Retinol dehydrogenase 14 GN=RDH14 PE=1 SV=1	RDH14_HUMAN	36,865.50	100.00%	2	2	2	0.00153%	6.55%
Pap test	Rho GDP-dissociation inhibitor 1 (Fragment) GN=ARHGDIA PE=1 SV=8	sp|P52565|GDIR1_HUMAN	21,517.20	100.00%	3	5	12	0.01870%	21.80%
Swab	Rho GDP-dissociation inhibitor 1 (Fragment) GN=ARHGDIA PE=1 SV=8	sp|P52565|GDIR1_HUMAN	21,517.20	100.00%	4	6	18	0.01280%	23.30%
Tumor tissue	Rho GDP-dissociation inhibitor 1 (Fragment) GN=ARHGDIA PE=1 SV=8	sp|P52565|GDIR1_HUMAN	21,517.20	100.00%	4	9	15	0.01150%	23.30%
Pap test	Rho GDP-dissociation inhibitor 2 GN=ARHGDIB PE=1 SV=3	GDIR2_HUMAN	22,988.40	100.00%	5	6	12	0.01870%	40.30%
Swab	Rho GDP-dissociation inhibitor 2 GN=ARHGDIB PE=1 SV=3	GDIR2_HUMAN	22,988.40	100.00%	5	11	27	0.01910%	42.80%
Tumor tissue	Rho GDP-dissociation inhibitor 2 GN=ARHGDIB PE=1 SV=3	GDIR2_HUMAN	22,988.40	100.00%	5	6	8	0.00612%	30.80%
Pap test	Rho GTPase-activating protein 1 GN=ARHGAP1 PE=1 SV=1	RHG01_HUMAN	50,437.90	100.00%	4	4	9	0.01400%	13.70%

Biological sample	Protein name	Protein accession numbers	Protein molecular weight (Da)	Protein identification probability	Exclusive unique peptide count	Exclusive unique spectrum count	Total spectrum count	Percentage of total spectra	Percentage sequence coverage
Swab	Rho GTPase-activating protein 1 GN=ARHGAP1 PE=1 SV=1	RHG01_HUMAN	50,437.90	100.00%	5	6	7	0.00496%	16.90%
Tumor tissue	Rho GTPase-activating protein 1 GN=ARHGAP1 PE=1 SV=1	RHG01_HUMAN	50,437.90	100.00%	15	22	42	0.03220%	45.60%
Tumor tissue	Rho GTPase-activating protein 21 GN=ARHGAP21 PE=1 SV=1	sp|Q5T5U3|RHG21_HUMAN	217,333.80	99.10%	1	1	2	0.00153%	2.25%
Tumor tissue	Rho GTPase-activating protein 23 GN=ARHGAP23 PE=1 SV=2	sp|Q9P227|RHG23_HUMAN	162,196.70	99.60%	1	1	2	0.00153%	1.61%
Tumor tissue	Rho GTPase-activating protein 35 GN=ARHGAP35 PE=1 SV=3	RHG35_HUMAN	170,518.90	100.00%	2	2	2	0.00153%	1.27%
Tumor tissue	Rho GTPase-activating protein 4 GN=ARHGAP4 PE=1 SV=1	sp|P98171|RHG04_HUMAN	102,571.20	100.00%	4	4	6	0.00459%	9.30%
Tumor tissue	Rho guanine nucleotide exchange factor 1 GN=ARHGEF1 PE=1 SV=1	M0QZR4_HUMAN	108,326.40	100.00%	1	1	14	0.01070%	14.70%
Swab	Rho guanine nucleotide exchange factor 16 GN=ARHGEF16 PE=1 SV=1	sp|Q5VV41|ARHGG_HUMAN	80,106.40	99.80%	1	1	1	0.00071%	2.54%
Tumor tissue	Rho guanine nucleotide exchange factor 16 GN=ARHGEF16 PE=1 SV=1	sp|Q5VV41|ARHGG_HUMAN	80,106.40	100.00%	2	2	2	0.00153%	4.65%
Tumor tissue	Rho guanine nucleotide exchange factor 17 GN=ARHGEF17 PE=1 SV=1	ARHGH_HUMAN	221,674.00	100.00%	2	2	2	0.00153%	1.02%
Tumor tissue	Rho guanine nucleotide exchange factor 2 GN=ARHGEF2 PE=1 SV=1	V9GYM8_HUMAN	116,074.00	100.00%	9	9	11	0.00842%	14.80%
Tumor tissue	Rho guanine nucleotide exchange factor 6 GN=ARHGEF6 PE=1 SV=2	sp|Q15052|ARHG6_HUMAN	87,500.60	100.00%	3	3	6	0.00459%	9.15%
Tumor tissue	Rho-associated protein kinase 1 GN=ROCK1 PE=1 SV=1	ROCK1_HUMAN	158,179.10	100.00%	3	3	4	0.00306%	3.18%
Tumor tissue	Rho-associated protein kinase 2 GN=ROCK2 PE=1 SV=4	ROCK2_HUMAN	160,905.80	100.00%	10	11	12	0.00919%	10.20%
Tumor tissue	Rhophilin-2 GN=RHPN2 PE=1 SV=1	sp|Q8IUC4|RHPN2_HUMAN	76,995.10	100.00%	2	2	2	0.00153%	6.12%
Swab	Rho-related GTP-binding protein RhoG GN=RHOG PE=1 SV=1	RHOG_HUMAN	21,308.10	100.00%	4	4	8	0.00567%	36.60%
Tumor tissue	Rho-related GTP-binding protein RhoG GN=RHOG PE=1 SV=1	RHOG_HUMAN	21,308.10	100.00%	4	6	7	0.00536%	28.80%
Tumor tissue	Rhotekin GN=RTKN PE=1 SV=2	sp|Q9BST9|RTKN_HUMAN	62,668.50	100.00%	1	1	2	0.00153%	5.86%

Biological sample	Protein name	Protein accession numbers	Protein molecular weight (Da)	Protein identification probability	Exclusive unique peptide count	Exclusive unique spectrum count	Total spectrum count	Percentage of total spectra	Percentage sequence coverage
Tumor tissue	Ribokinase GN=RBKS PE=1 SV=1	sp|Q9H477|RBSK_HUMAN	34,143.00	100.00%	2	2	2	0.00153%	6.21%
Pap test	Ribonuclease inhibitor GN=RNH1 PE=1 SV=2	RINI_HUMAN	49,974.40	100.00%	7	9	17	0.02650%	33.80%
Swab	Ribonuclease inhibitor GN=RNH1 PE=1 SV=2	RINI_HUMAN	49,974.40	100.00%	8	10	23	0.01630%	33.20%
Tumor tissue	Ribonuclease inhibitor GN=RNH1 PE=1 SV=2	RINI_HUMAN	49,974.40	100.00%	3	4	9	0.00689%	11.30%
Tumor tissue	Ribonuclease P 40kDa subunit, isoform CRA_c GN=RPP40 PE=1 SV=1	sp|O75818|RPP40_HUMAN	39,328.30	100.00%	2	2	2	0.00153%	9.03%
Pap test	Ribonuclease pancreatic GN=RNASE1 PE=1 SV=4	RNAS1_HUMAN	17,643.80	99.60%	1	1	1	0.00156%	7.05%
Swab	Ribonuclease pancreatic GN=RNASE1 PE=1 SV=4	RNAS1_HUMAN	17,643.80	100.00%	2	3	4	0.00283%	21.80%
Pap test	Ribonuclease T2 GN=RNASET2 PE=1 SV=1	D6RHI9_HUMAN	29,114.80	100.00%	1	1	3	0.00468%	9.80%
Swab	Ribonuclease T2 GN=RNASET2 PE=1 SV=1	D6RHI9_HUMAN	29,114.80	100.00%	1	1	2	0.00142%	9.48%
Tumor tissue	Ribonuclease T2 GN=RNASET2 PE=1 SV=1	D6RHI9_HUMAN	29,114.80	99.90%	1	1	1	0.00077%	3.27%
Tumor tissue	Ribonucleases P/MRP protein subunit POP1 GN=POP1 PE=1 SV=2	POP1_HUMAN	114,710.30	100.00%	2	2	2	0.00153%	2.54%
Tumor tissue	Ribonucleoprotein PTB-binding 1 GN=RAVER1 PE=1 SV=1	sp|Q8IY67-2|RAVR1_HUMAN	79,582.00	100.00%	2	2	6	0.00459%	15.20%
Tumor tissue	Ribonucleoside-diphosphate reductase large subunit GN=RRM1 PE=1 SV=1	RIR1_HUMAN	90,073.50	100.00%	2	2	2	0.00153%	2.90%
Pap test	Ribose-5-phosphate isomerase GN=RPIA PE=1 SV=3	RPIA_HUMAN	33,269.00	100.00%	3	3	3	0.00468%	13.80%
Swab	Ribose-5-phosphate isomerase GN=RPIA PE=1 SV=3	RPIA_HUMAN	33,269.00	100.00%	2	2	2	0.00142%	5.14%
Tumor tissue	Ribose-5-phosphate isomerase GN=RPIA PE=1 SV=3	RPIA_HUMAN	33,269.00	100.00%	4	4	4	0.00306%	12.90%

Biological sample	Protein name	Protein accession numbers	Protein molecular weight (Da)	Protein identification probability	Exclusive unique peptide count	Exclusive unique spectrum count	Total spectrum count	Percentage of total spectra	Percentage sequence coverage
Tumor tissue	Ribose-phosphate pyrophosphokinase 1 GN=PRPS1 PE=1 SV=2	sp|P60891|PRPS1_HUMAN	34,834.70	100.00%	1	1	10	0.00766%	17.90%
Tumor tissue	Ribosomal L1 domain-containing protein 1 GN=RSL1D1 PE=1 SV=3	sp|O76021|RL1D1_HUMAN	54,974.70	100.00%	8	11	14	0.01070%	28.80%
Tumor tissue	Ribosomal protein L19 GN=RPL19 PE=1 SV=1	RL19_HUMAN	23,249.20	100.00%	3	5	13	0.00995%	23.20%
Tumor tissue	Ribosomal protein S6 kinase alpha-3 GN=RPS6KA3 PE=1 SV=1	KS6A3_HUMAN	83,738.50	100.00%	4	4	8	0.00612%	12.60%
Tumor tissue	Ribosomal RNA processing protein 1 homolog A GN=RRP1 PE=1 SV=1	RRP1_HUMAN	52,840.60	100.00%	2	2	2	0.00153%	5.64%
Tumor tissue	Ribosomal RNA small subunit methyltransferase NEP1 GN=EMG1 PE=1 SV=4	NEP1_HUMAN	26,720.30	100.00%	2	3	6	0.00459%	13.90%
Tumor tissue	Ribosome biogenesis protein BMS1 homolog GN=BMS1 PE=1 SV=1	BMS1_HUMAN	145,812.20	100.00%	3	3	4	0.00306%	2.81%
Tumor tissue	Ribosome biogenesis protein BOP1 GN=BOP1 PE=1 SV=2	sp|Q14137|BOP1_HUMAN	83,629.30	100.00%	5	5	5	0.00383%	9.92%
Tumor tissue	Ribosome biogenesis protein BRX1 homolog GN=BRIX1 PE=1 SV=2	BRX1_HUMAN	41,403.00	100.00%	4	4	4	0.00306%	18.40%
Tumor tissue	Ribosome biogenesis protein WDR12 GN=WDR12 PE=1 SV=2	WDR12_HUMAN	47,708.50	100.00%	3	3	4	0.00306%	9.93%
Tumor tissue	Ribosome biogenesis regulatory protein homolog GN=RRS1 PE=1 SV=2	RRS1_HUMAN	41,194.40	100.00%	3	3	3	0.00230%	11.20%
Tumor tissue	Ribosome maturation protein SBDS GN=SBDS PE=1 SV=1	SBDS_HUMAN	28,865.00	100.00%	4	4	4	0.00306%	17.60%
Pap test	Ribosome-binding protein 1 GN=RRBP1 PE=1 SV=1	sp|Q9P2E9|RRBP1_HUMAN	152,454.60	100.00%	5	5	6	0.00936%	4.68%
Swab	Ribosome-binding protein 1 GN=RRBP1 PE=1 SV=1	sp|Q9P2E9|RRBP1_HUMAN	152,454.60	100.00%	3	4	6	0.00425%	4.47%
Tumor tissue	Ribosome-binding protein 1 GN=RRBP1 PE=1 SV=1	sp|Q9P2E9|RRBP1_HUMAN	152,454.60	100.00%	33	53	95	0.07270%	30.00%
Swab	Ribosyldihydronicotinamide dehydrogenase [quinone] GN=NQO2 PE=1 SV=5	NQO2_HUMAN	21,537.40	100.00%	4	6	7	0.00496%	29.40%

Biological sample	Protein name	Protein accession numbers	Protein molecular weight (Da)	Protein identification probability	Exclusive unique peptide count	Exclusive unique spectrum count	Total spectrum count	Percentage of total spectra	Percentage sequence coverage
Tumor tissue	Ribosyldihydronicotinamide dehydrogenase [quinone] GN=NQO2 PE=1 SV=5	NQO2_HUMAN	21,537.40	100.00%	5	5	5	0.00383%	18.20%
Tumor tissue	RNA binding motif protein 10, isoform CRA_d GN=RBM10 PE=1 SV=1	A0A0A0MR66_HUMAN	110,368.10	99.90%	1	1	2	0.00153%	2.31%
Tumor tissue	RNA binding motif protein, X-linked-like-1 GN=RBMXL1 PE=1 SV=1	RMXL1_HUMAN	42,143.40	100.00%	1	1	13	0.00995%	24.10%
Tumor tissue	RNA cytidine acetyltransferase GN=NAT10 PE=1 SV=2	sp|Q9H0A0|NAT10_HUMAN	115,734.30	100.00%	6	7	7	0.00536%	6.93%
Tumor tissue	RNA polymerase II-associated protein 3 GN=RPAP3 PE=1 SV=2	sp|Q9H6T3|RPAP3_HUMAN	75,722.30	100.00%	3	3	3	0.00230%	5.71%
Tumor tissue	RNA polymerase-associated protein RTF1 homolog GN=RTF1 PE=1 SV=4	RTF1_HUMAN	80,315.70	100.00%	5	5	5	0.00383%	8.45%
Tumor tissue	RNA-binding motif protein, X chromosome GN=RBMX PE=1 SV=3	sp|P38159|RBMX_HUMAN	42,333.70	100.00%	2	4	20	0.01530%	28.10%
Tumor tissue	RNA-binding protein 12 GN=RBM12 PE=1 SV=1	RBM12_HUMAN	97,396.80	100.00%	5	5	5	0.00383%	7.40%
Tumor tissue	RNA-binding protein 12B GN=RBM12B PE=1 SV=2	RB12B_HUMAN	118,106.60	100.00%	2	2	3	0.00230%	3.70%
Tumor tissue	RNA-binding protein 14 GN=RBM14 PE=1 SV=2	sp|Q96PK6|RBM14_HUMAN	69,492.30	100.00%	8	9	11	0.00842%	17.80%
Tumor tissue	RNA-binding protein 25 GN=RBM25 PE=1 SV=3	sp|P49756|RBM25_HUMAN	100,189.10	100.00%	2	3	3	0.00230%	3.20%
Tumor tissue	RNA-binding protein 3 GN=RBM3 PE=1 SV=1	RBM3_HUMAN	17,170.60	100.00%	3	5	10	0.00766%	27.40%
Tumor tissue	RNA-binding protein 38 GN=RBM38 PE=1 SV=1	sp|Q9H0Z9|RBM38_HUMAN	13,604.90	99.40%	1	1	2	0.00153%	29.30%
Tumor tissue	RNA-binding protein 4 GN=RBM4 PE=1 SV=1	sp|Q9BWF3|RBM4_HUMAN	40,314.20	100.00%	2	2	5	0.00383%	20.30%
Pap test	RNA-binding protein EWS GN=EWSR1 PE=1 SV=1	sp|Q01844|EWS_HUMAN	64,929.40	100.00%	1	1	2	0.00312%	2.27%
Swab	RNA-binding protein EWS GN=EWSR1 PE=1 SV=1	sp|Q01844|EWS_HUMAN	64,929.40	98.10%	1	1	1	0.00071%	2.27%
Tumor tissue	RNA-binding protein EWS GN=EWSR1 PE=1 SV=1	sp|Q01844|EWS_HUMAN	64,929.40	100.00%	4	10	11	0.00842%	11.50%

Biological sample	Protein name	Protein accession numbers	Protein molecular weight (Da)	Protein identification probability	Exclusive unique peptide count	Exclusive unique spectrum count	Total spectrum count	Percentage of total spectra	Percentage sequence coverage
Tumor tissue	RNA-binding protein FUS GN=FUS PE=1 SV=1	sp|P35637|FUS_HUMAN	53,497.10	100.00%	5	7	13	0.00995%	10.80%
Tumor tissue	RNA-binding protein Musashi homolog 2 GN=MSI2 PE=1 SV=1	sp|Q96DH6|MSI2H_HUMAN	34,812.60	100.00%	3	3	3	0.00230%	13.00%
Tumor tissue	RNA-binding protein PNO1 GN=PNO1 PE=1 SV=1	PNO1_HUMAN	27,925.30	100.00%	2	2	2	0.00153%	22.10%
Tumor tissue	RNA-binding protein with serine-rich domain 1 (Fragment) GN=RNPS1 PE=1 SV=1	sp|Q15287|RNPS1_HUMAN	33,879.20	100.00%	1	1	3	0.00230%	12.70%
Tumor tissue	rRNA 2'-O-methyltransferase fibrillarin GN=FBL PE=1 SV=2	FBRL_HUMAN	33,784.10	100.00%	1	2	26	0.01990%	36.80%
Tumor tissue	RUN and FYVE domain-containing protein 1 GN=RUFY1 PE=1 SV=2	sp|Q96T51|RUFY1_HUMAN	79,819.30	100.00%	1	1	3	0.00230%	5.08%
Tumor tissue	RUS1 family protein C16orf58 GN=C16orf58 PE=1 SV=1	sp|Q96GQ5|RUS1_HUMAN	50,783.60	100.00%	1	1	2	0.00153%	5.15%
Pap test	RuvB-like 1 GN=RUVBL1 PE=1 SV=1	sp|Q9Y265|RUVB1_HUMAN	50,229.40	99.20%	1	1	1	0.00156%	2.63%
Swab	RuvB-like 1 GN=RUVBL1 PE=1 SV=1	sp|Q9Y265|RUVB1_HUMAN	50,229.40	100.00%	2	2	2	0.00142%	6.14%
Tumor tissue	RuvB-like 1 GN=RUVBL1 PE=1 SV=1	sp|Q9Y265|RUVB1_HUMAN	50,229.40	100.00%	11	16	25	0.01910%	33.60%
Tumor tissue	RuvB-like 2 GN=RUVBL2 PE=1 SV=3	sp|Q9Y230|RUVB2_HUMAN	51,158.10	100.00%	9	13	23	0.01760%	40.60%
Tumor tissue	S1 RNA-binding domain-containing protein 1 GN=SRBD1 PE=1 SV=2	sp|Q8N5C6|SRBD1_HUMAN	111,778.10	100.00%	2	3	3	0.00230%	3.22%
Tumor tissue	Saccharopine dehydrogenase-like oxidoreductase GN=SCCPDH PE=1 SV=1	SCPDL_HUMAN	47,152.60	100.00%	3	3	4	0.00306%	9.56%
Swab	S-adenosylmethionine synthase isoform type-2 GN=MAT2A PE=1 SV=1	sp|P31153|METK2_HUMAN	43,660.90	99.80%	1	1	2	0.00142%	3.80%
Tumor tissue	S-adenosylmethionine synthase isoform type-2 GN=MAT2A PE=1 SV=1	sp|P31153|METK2_HUMAN	43,660.90	100.00%	5	6	7	0.00536%	20.80%
Tumor tissue	SAFB-like transcription modulator GN=SLTM PE=1 SV=2	sp|Q9NWH9|SLTM_HUMAN	117,151.10	100.00%	2	2	4	0.00306%	6.29%
Tumor tissue	SAP30-binding protein (Fragment) GN=SAP30BP PE=1 SV=1	sp|Q9UHR5|S30BP_HUMAN	35,883.80	100.00%	1	1	2	0.00153%	8.31%

Biological sample	Protein name	Protein accession numbers	Protein molecular weight (Da)	Protein identification probability	Exclusive unique peptide count	Exclusive unique spectrum count	Total spectrum count	Percentage of total spectra	Percentage sequence coverage
Tumor tissue	Sarcolemmal membrane-associated protein GN=SLMAP PE=1 SV=1	sp|Q14BN4-5|SLMAP_HUMAN	42,037.50	100.00%	1	1	9	0.00689%	21.50%
Pap test	Sarcoplasmic/endoplasmic reticulum calcium ATPase 2 GN=ATP2A2 PE=1 SV=1	sp|P16615|AT2A2_HUMAN	114,758.80	100.00%	2	2	2	0.00312%	2.69%
Tumor tissue	Sarcoplasmic/endoplasmic reticulum calcium ATPase 2 GN=ATP2A2 PE=1 SV=1	sp|P16615|AT2A2_HUMAN	114,758.80	100.00%	10	12	21	0.01610%	19.80%
Tumor tissue	Scaffold attachment factor B2 GN=SAFB2 PE=1 SV=1	sp|Q14151|SAFB2_HUMAN	107,474.40	100.00%	2	2	10	0.00766%	10.10%
Tumor tissue	Schlafen family member 5 GN=SLFN5 PE=1 SV=1	sp|Q08AF3|SLFN5_HUMAN	101,057.10	100.00%	3	4	5	0.00383%	5.95%
Tumor tissue	SCY1-like protein 2 GN=SCYL2 PE=1 SV=1	SCYL2_HUMAN	104,115.10	100.00%	2	2	3	0.00230%	5.04%
Pap test	Sec1 family domain-containing protein 1 GN=SCFD1 PE=1 SV=4	sp|Q8WVM8|SCFD1_HUMAN	72,382.30	99.20%	1	1	1	0.00156%	2.02%
Tumor tissue	Sec1 family domain-containing protein 1 GN=SCFD1 PE=1 SV=4	sp|Q8WVM8|SCFD1_HUMAN	72,382.30	100.00%	2	2	15	0.01150%	20.60%
Tumor tissue	SEC23-interacting protein GN=SEC23IP PE=1 SV=1	sp|Q9Y6Y8|S23IP_HUMAN	111,078.00	100.00%	13	14	15	0.01150%	17.30%
Pap test	Secernin-2 GN=SCRN2 PE=1 SV=1	sp|Q96FV2|SCRN2_HUMAN	47,493.00	100.00%	3	4	5	0.00780%	10.60%
Swab	Secernin-2 GN=SCRN2 PE=1 SV=1	sp|Q96FV2|SCRN2_HUMAN	47,493.00	100.00%	2	2	3	0.00213%	8.08%
Tumor tissue	Secernin-2 GN=SCRN2 PE=1 SV=1	sp|Q96FV2|SCRN2_HUMAN	47,493.00	100.00%	3	3	3	0.00230%	12.70%
Tumor tissue	Secretory carrier-associated membrane protein 1 GN=SCAMP1 PE=1 SV=1	sp|O15126|SCAM1_HUMAN	35,046.80	99.90%	1	1	3	0.00230%	15.70%
Tumor tissue	Selenide, water dikinase 1 GN=SEPHS1 PE=1 SV=2	sp|P49903|SPS1_HUMAN	42,910.50	100.00%	1	1	13	0.00995%	21.20%
Tumor tissue	Selenide, water dikinase 2 GN=SEPHS2 PE=1 SV=3	SPS2_HUMAN	47,305.00	100.00%	2	3	3	0.00230%	10.30%
Tumor tissue	Selenocysteine lyase GN=SCLY PE=1 SV=1	A0A0A0MQU4_HUMAN	48,792.80	100.00%	4	4	4	0.00306%	15.70%
Tumor tissue	Selenocysteine-specific elongation factor GN=EEFSEC PE=1 SV=4	sp|P57772|SELB_HUMAN	65,306.10	100.00%	4	4	4	0.00306%	11.20%

Biological sample	Protein name	Protein accession numbers	Protein molecular weight (Da)	Protein identification probability	Exclusive unique peptide count	Exclusive unique spectrum count	Total spectrum count	Percentage of total spectra	Percentage sequence coverage
Tumor tissue	Selenoprotein M GN=SELM PE=1 SV=1	SELM_HUMAN	16,082.20	100.00%	2	3	3	0.00230%	19.40%
Tumor tissue	Selenoprotein S GN=VIMP PE=1 SV=3	SELS_HUMAN	21,163.40	100.00%	2	2	2	0.00153%	15.30%
Swab	Sepiapterin reductase GN=SPR PE=1 SV=1	SPRE_HUMAN	28,049.50	100.00%	4	4	4	0.00283%	19.20%
Tumor tissue	Sepiapterin reductase GN=SPR PE=1 SV=1	SPRE_HUMAN	28,049.50	100.00%	5	6	8	0.00612%	27.60%
Tumor tissue	Septin 10, isoform CRA_c GN=SEPT10 PE=1 SV=2	sp|Q9P0V9-2|SEP10_HUMAN	62,945.60	100.00%	4	4	5	0.00383%	11.80%
Pap test	Septin-6 GN=SEPT6 PE=1 SV=4	sp|Q14141|SEPT6_HUMAN	49,717.60	99.90%	1	1	4	0.00624%	6.91%
Tumor tissue	Septin-6 GN=SEPT6 PE=1 SV=4	sp|Q14141|SEPT6_HUMAN	49,717.60	100.00%	4	4	14	0.01070%	30.20%
Tumor tissue	Serine beta-lactamase-like protein LACTB, mitochondrial GN=LACTB PE=1 SV=2	sp|P83111|LACTB_HUMAN	60,694.80	100.00%	8	9	10	0.00766%	25.00%
Tumor tissue	Serine dehydratase-like GN=SDSL PE=1 SV=1	SDSL_HUMAN	34,673.20	100.00%	2	3	3	0.00230%	13.40%
Tumor tissue	Serine palmitoyltransferase 1 GN=SPTLC1 PE=1 SV=1	sp|O15269|SPTC1_HUMAN	52,745.50	100.00%	3	3	4	0.00306%	10.80%
Tumor tissue	Serine palmitoyltransferase 2 GN=SPTLC2 PE=1 SV=1	SPTC2_HUMAN	62,925.70	100.00%	3	3	3	0.00230%	10.90%
Tumor tissue	Serine protease HTRA1 GN=HTRA1 PE=1 SV=1	HTRA1_HUMAN	51,287.30	100.00%	4	4	6	0.00459%	8.54%
Tumor tissue	Serine protease HTRA2, mitochondrial (Fragment) GN=HTRA2 PE=1 SV=1	sp|O43464-3|HTRA2_HUMAN	45,112.60	100.00%	2	2	3	0.00230%	8.98%
Tumor tissue	Serine/arginine repetitive matrix protein 1 GN=SRRM1 PE=1 SV=1	sp|Q8IYB3|SRRM1_HUMAN	103,392.70	100.00%	2	3	6	0.00459%	3.18%
Tumor tissue	Serine/arginine repetitive matrix protein 2 GN=SRRM2 PE=1 SV=2	sp|Q9UQ35|SRRM2_HUMAN	299,621.60	100.00%	12	14	14	0.01070%	7.23%
Tumor tissue	Serine/arginine-rich splicing factor 4 GN=SRSF4 PE=1 SV=2	SRSF4_HUMAN	56,680.00	100.00%	1	1	4	0.00306%	8.50%
Tumor tissue	Serine/arginine-rich splicing factor 9 GN=SRSF9 PE=1 SV=1	SRSF9_HUMAN	25,542.90	100.00%	8	11	14	0.01070%	36.20%

Biological sample	Protein name	Protein accession numbers	Protein molecular weight (Da)	Protein identification probability	Exclusive unique peptide count	Exclusive unique spectrum count	Total spectrum count	Percentage of total spectra	Percentage sequence coverage
Pap test	Serine/arginine-rich-splicing factor 1 GN=SRSF1 PE=1 SV=1	sp|Q07955|SRSF1_HUMAN	28,329.70	100.00%	2	2	3	0.00468%	8.70%
Swab	Serine/arginine-rich-splicing factor 1 GN=SRSF1 PE=1 SV=1	sp|Q07955|SRSF1_HUMAN	28,329.70	100.00%	3	3	3	0.00213%	12.60%
Tumor tissue	Serine/arginine-rich-splicing factor 1 GN=SRSF1 PE=1 SV=1	sp|Q07955|SRSF1_HUMAN	28,329.70	100.00%	7	9	27	0.02070%	29.20%
Tumor tissue	Serine/arginine-rich-splicing factor 11 (Fragment) GN=SRSF11 PE=1 SV=1	sp|Q05519|SRS11_HUMAN	42,317.50	100.00%	2	3	4	0.00306%	9.00%
Swab	Serine/arginine-rich-splicing factor 7 GN=SRSF7 PE=1 SV=1	sp|Q16629|SRSF7_HUMAN	26,929.10	100.00%	1	1	1	0.00071%	8.76%
Tumor tissue	Serine/arginine-rich-splicing factor 7 GN=SRSF7 PE=1 SV=1	sp|Q16629|SRSF7_HUMAN	26,929.10	100.00%	2	2	9	0.00689%	25.50%
Tumor tissue	Serine/threonine-protein kinase GN=PRKD1 PE=1 SV=1	KPCD1_HUMAN	102,506.30	100.00%	2	2	3	0.00230%	3.37%
Tumor tissue	Serine/threonine-protein kinase 10 GN=STK10 PE=1 SV=1	STK10_HUMAN	112,138.10	100.00%	6	8	9	0.00689%	10.10%
Tumor tissue	Serine/threonine-protein kinase 17B GN=STK17B PE=1 SV=1	ST17B_HUMAN	42,345.40	100.00%	2	2	2	0.00153%	6.18%
Tumor tissue	Serine/threonine-protein kinase 24 GN=STK24 PE=1 SV=1	sp|Q9Y6E0-2|STK24_HUMAN	45,838.10	100.00%	2	2	7	0.00536%	17.50%
Tumor tissue	Serine/threonine-protein kinase 4 GN=STK4 PE=1 SV=2	sp|Q13043|STK4_HUMAN	55,631.30	100.00%	2	2	7	0.00536%	16.60%
Tumor tissue	Serine/threonine-protein kinase MRCK beta GN=CDC42BPB PE=1 SV=2	MRCKB_HUMAN	194,318.00	100.00%	7	8	9	0.00689%	6.31%
Tumor tissue	Serine/threonine-protein kinase mTOR GN=MTOR PE=1 SV=1	MTOR_HUMAN	288,896.40	100.00%	6	7	8	0.00612%	3.84%
Tumor tissue	Serine/threonine-protein kinase Nek9 GN=NEK9 PE=1 SV=2	NEK9_HUMAN	107,169.50	100.00%	7	7	7	0.00536%	9.70%
Pap test	Serine/threonine-protein kinase OSR1 GN=OXSR1 PE=1 SV=1	OXSR1_HUMAN	58,023.40	100.00%	3	4	4	0.00624%	7.97%
Swab	Serine/threonine-protein kinase OSR1 GN=OXSR1 PE=1 SV=1	OXSR1_HUMAN	58,023.40	99.80%	1	1	1	0.00071%	1.52%
Tumor tissue	Serine/threonine-protein kinase OSR1 GN=OXSR1 PE=1 SV=1	OXSR1_HUMAN	58,023.40	100.00%	4	5	8	0.00612%	10.60%

Biological sample	Protein name	Protein accession numbers	Protein molecular weight (Da)	Protein identification probability	Exclusive unique peptide count	Exclusive unique spectrum count	Total spectrum count	Percentage of total spectra	Percentage sequence coverage
Tumor tissue	Serine/threonine-protein kinase PAK 1 GN=PAK1 PE=1 SV=2	sp|Q13153|PAK1_HUMAN	60,648.30	100.00%	1	2	12	0.00919%	19.40%
Swab	Serine/threonine-protein kinase PAK 2 GN=PAK2 PE=1 SV=3	PAK2_HUMAN	58,044.10	100.00%	2	3	3	0.00213%	8.40%
Tumor tissue	Serine/threonine-protein kinase PAK 2 GN=PAK2 PE=1 SV=3	PAK2_HUMAN	58,044.10	100.00%	8	11	20	0.01530%	33.40%
Tumor tissue	Serine/threonine-protein kinase PRP4 homolog GN=PRPF4B PE=1 SV=3	PRP4B_HUMAN	116,993.00	100.00%	6	7	8	0.00612%	7.25%
Tumor tissue	Serine/threonine-protein kinase TBK1 GN=TBK1 PE=1 SV=1	TBK1_HUMAN	83,644.70	100.00%	3	3	3	0.00230%	5.62%
Tumor tissue	Serine/threonine-protein kinase VRK1 GN=VRK1 PE=1 SV=1	VRK1_HUMAN	45,477.90	100.00%	3	3	4	0.00306%	18.20%
Tumor tissue	Serine/threonine-protein phosphatase 1 regulatory subunit 10 GN=PPP1R10 PE=1 SV=1	PP1RA_HUMAN	99,058.80	100.00%	2	2	2	0.00153%	4.57%
Tumor tissue	Serine/threonine-protein phosphatase 2A 55 kDa regulatory subunit B delta isoform GN=PPP2R2D PE=2 SV=1	2ABD_HUMAN	52,043.80	99.90%	1	1	2	0.00153%	6.18%
Tumor tissue	Serine/threonine-protein phosphatase 2A 56 kDa regulatory subunit delta isoform GN=PPP2R5D PE=1 SV=1	sp|Q14738|2A5D_HUMAN	69,119.50	100.00%	5	5	12	0.00919%	14.00%
Tumor tissue	Serine/threonine-protein phosphatase 2A 65 kDa regulatory subunit A alpha isoform GN=PPP2R1A PE=1 SV=4	2AAA_HUMAN	65,309.70	100.00%	2	5	47	0.03600%	32.30%
Swab	Serine/threonine-protein phosphatase 2A activator GN=PTPA PE=1 SV=1	sp|Q15257|PTPA_HUMAN	37,376.30	100.00%	1	1	7	0.00496%	10.90%
Tumor tissue	Serine/threonine-protein phosphatase 2A activator GN=PTPA PE=1 SV=1	sp|Q15257|PTPA_HUMAN	37,376.30	100.00%	2	2	10	0.00766%	21.00%

Biological sample	Protein name	Protein accession numbers	Protein molecular weight (Da)	Protein identification probability	Exclusive unique peptide count	Exclusive unique spectrum count	Total spectrum count	Percentage of total spectra	Percentage sequence coverage
Pap test	Serine/threonine-protein phosphatase 2A catalytic subunit beta isoform GN=PPP2CB PE=1 SV=1	PP2AB_HUMAN	35,575.40	100.00%	3	3	3	0.00468%	14.90%
Swab	Serine/threonine-protein phosphatase 2A catalytic subunit beta isoform GN=PPP2CB PE=1 SV=1	PP2AB_HUMAN	35,575.40	100.00%	3	4	5	0.00354%	18.80%
Tumor tissue	Serine/threonine-protein phosphatase 2A catalytic subunit beta isoform GN=PPP2CB PE=1 SV=1	PP2AB_HUMAN	35,575.40	100.00%	2	2	4	0.00306%	16.50%
Tumor tissue	Serine/threonine-protein phosphatase 4 catalytic subunit GN=PPP4C PE=1 SV=1	PP4C_HUMAN	35,080.40	100.00%	2	2	2	0.00153%	6.84%
Tumor tissue	Serine/threonine-protein phosphatase 5 GN=PPP5C PE=1 SV=1	PPP5_HUMAN	56,880.30	100.00%	1	1	2	0.00153%	4.21%
Tumor tissue	Serine/threonine-protein phosphatase 6 regulatory subunit 1 GN=PPP6R1 PE=1 SV=5	PP6R1_HUMAN	96,724.10	100.00%	2	2	2	0.00153%	2.50%
Tumor tissue	Serine/threonine-protein phosphatase 6 regulatory subunit 3 GN=PPP6R3 PE=1 SV=1	E9PKF6_HUMAN	93,735.30	100.00%	1	1	3	0.00230%	3.34%
Pap test	Serine/threonine-protein phosphatase CPPED1 GN=CPPED1 PE=1 SV=3	sp|Q9BRF8|CPPED_HUMAN	35,548.70	100.00%	2	2	2	0.00312%	12.10%
Swab	Serine/threonine-protein phosphatase CPPED1 GN=CPPED1 PE=1 SV=3	sp|Q9BRF8|CPPED_HUMAN	35,548.70	100.00%	4	4	6	0.00425%	22.90%
Tumor tissue	Serine/threonine-protein phosphatase CPPED1 GN=CPPED1 PE=1 SV=3	sp|Q9BRF8|CPPED_HUMAN	35,548.70	100.00%	3	3	3	0.00230%	11.50%
Pap test	Serine/threonine-protein phosphatase PP1-beta catalytic subunit GN=PPP1CB PE=1 SV=3	PP1B_HUMAN	37,188.30	99.70%	1	1	5	0.00780%	13.80%

Biological sample	Protein name	Protein accession numbers	Protein molecular weight (Da)	Protein identification probability	Exclusive unique peptide count	Exclusive unique spectrum count	Total spectrum count	Percentage of total spectra	Percentage sequence coverage
Tumor tissue	Serine/threonine-protein phosphatase PP1-beta catalytic subunit GN=PPP1CB PE=1 SV=3	PP1B_HUMAN	37,188.30	99.90%	1	2	11	0.00842%	16.50%
Tumor tissue	Serine-protein kinase ATM GN=ATM PE=1 SV=4	ATM_HUMAN	350,693.50	100.00%	3	3	3	0.00230%	1.01%
Tumor tissue	Serine--tRNA ligase, mitochondrial GN=SARS2 PE=1 SV=1	M0QWZ7_HUMAN	58,183.50	100.00%	1	1	13	0.00995%	28.20%
Pap test	Serotransferrin GN=TF PE=1 SV=3	TRFE_HUMAN	77,064.30	100.00%	36	91	519	0.81000%	59.90%
Swab	Serotransferrin GN=TF PE=1 SV=3	TRFE_HUMAN	77,064.30	100.00%	37	92	729	0.51700%	58.60%
Tumor tissue	Serotransferrin GN=TF PE=1 SV=3	TRFE_HUMAN	77,064.30	100.00%	13	25	61	0.04670%	21.20%
Swab	Serotransferrin (Fragment) GN=TF PE=1 SV=1	C9JVG0_HUMAN	14,691.10	99.70%	1	2	102	0.07230%	50.70%
Swab	Serpin B10 GN=SERPINB10 PE=1 SV=1	SPB10_HUMAN	45,404.90	100.00%	4	5	5	0.00354%	15.10%
Pap test	Serpin B13 GN=SERPINB13 PE=1 SV=1	F8WE70_HUMAN	18,885.80	99.80%	1	1	8	0.01250%	36.40%
Pap test	Serpin B13 GN=SERPINB13 PE=1 SV=2	sp|Q9UIV8|SPB13_HUMAN	44,277.40	100.00%	5	7	14	0.02180%	26.10%
Swab	Serpin B13 GN=SERPINB13 PE=1 SV=2	sp|Q9UIV8|SPB13_HUMAN	44,277.40	100.00%	5	8	19	0.01350%	32.50%
Pap test	Serpin B3 GN=SERPINB3 PE=1 SV=2	sp|P29508|SPB3_HUMAN	44,565.90	100.00%	11	20	152	0.23700%	47.70%
Swab	Serpin B3 GN=SERPINB3 PE=1 SV=2	sp|P29508|SPB3_HUMAN	44,565.90	100.00%	7	10	58	0.04110%	39.00%
Pap test	Serpin B4 GN=SERPINB4 PE=1 SV=2	SPB4_HUMAN	44,855.00	100.00%	5	7	103	0.16100%	35.10%
Swab	Serpin B4 GN=SERPINB4 PE=1 SV=2	SPB4_HUMAN	44,855.00	100.00%	3	4	39	0.02760%	29.00%
Pap test	Serpin B5 GN=SERPINB5 PE=1 SV=2	sp|P36952|SPB5_HUMAN	42,101.80	100.00%	5	5	6	0.00936%	20.50%
Swab	Serpin B5 GN=SERPINB5 PE=1 SV=2	sp|P36952|SPB5_HUMAN	42,101.80	100.00%	9	12	24	0.01700%	38.90%
Pap test	Serpin B6 GN=SERPINB6 PE=1 SV=1	A0A024QZX5_HUMAN	42,622.90	100.00%	7	7	8	0.01250%	26.60%
Swab	Serpin B6 GN=SERPINB6 PE=1 SV=1	A0A024QZX5_HUMAN	43,025.40	100.00%	9	16	25	0.01770%	35.30%
Tumor tissue	Serpin B6 GN=SERPINB6 PE=1 SV=1	A0A024QZX5_HUMAN	43,025.40	100.00%	9	12	14	0.01070%	34.70%
Pap test	Serpin B8 GN=SERPINB8 PE=1 SV=2	sp|P50452|SPB8_HUMAN	42,767.90	100.00%	2	2	8	0.01250%	11.20%
Swab	Serpin B8 GN=SERPINB8 PE=1 SV=2	sp|P50452|SPB8_HUMAN	42,767.90	100.00%	3	3	3	0.00213%	12.00%
Tumor tissue	Serpin B8 GN=SERPINB8 PE=1 SV=2	sp|P50452|SPB8_HUMAN	42,767.90	100.00%	2	2	10	0.00766%	9.63%
Swab	Serpin B9 GN=SERPINB9 PE=1 SV=1	SPB9_HUMAN	42,404.30	99.50%	1	1	1	0.00071%	8.24%

Biological sample	Protein name	Protein accession numbers	Protein molecular weight (Da)	Protein identification probability	Exclusive unique peptide count	Exclusive unique spectrum count	Total spectrum count	Percentage of total spectra	Percentage sequence coverage
Tumor tissue	Serpin B9 GN=SERPINB9 PE=1 SV=1	SPB9_HUMAN	42,404.30	100.00%	8	8	16	0.01220%	28.50%
Tumor tissue	Serpin H1 GN=SERPINH1 PE=1 SV=2	SERPH_HUMAN	46,442.00	100.00%	23	49	160	0.12200%	61.70%
Pap test	Serum albumin GN=ALB PE=1 SV=2	sp|P02768|ALBU_HUMAN	69,366.90	100.00%	62	157	2042	3.19000%	83.40%
Swab	Serum albumin GN=ALB PE=1 SV=2	sp|P02768|ALBU_HUMAN	69,366.90	100.00%	53	170	5285	3.75000%	76.80%
Tumor tissue	Serum albumin GN=ALB PE=1 SV=2	sp|P02768|ALBU_HUMAN	69,366.90	100.00%	23	40	207	0.15800%	34.50%
Pap test	Serum amyloid P-component GN=APCS PE=1 SV=2	SAMP_HUMAN	25,387.70	100.00%	5	5	5	0.00780%	23.80%
Swab	Serum amyloid P-component GN=APCS PE=1 SV=2	SAMP_HUMAN	25,387.70	100.00%	2	2	2	0.00142%	10.30%
Tumor tissue	Serum amyloid P-component GN=APCS PE=1 SV=2	SAMP_HUMAN	25,387.70	100.00%	7	9	20	0.01530%	30.00%
Pap test	Sex hormone-binding globulin GN=SHBG PE=1 SV=1	sp|P04278|SHBG_HUMAN	37,489.00	99.70%	1	1	1	0.00156%	3.49%
Swab	Sex hormone-binding globulin GN=SHBG PE=1 SV=1	sp|P04278|SHBG_HUMAN	37,489.00	100.00%	4	4	9	0.00638%	17.70%
Swab	S-formylglutathione hydrolase GN=ESD PE=1 SV=2	ESTD_HUMAN	31,463.60	100.00%	1	1	1	0.00071%	4.26%
Tumor tissue	S-formylglutathione hydrolase GN=ESD PE=1 SV=2	ESTD_HUMAN	31,463.60	100.00%	4	4	4	0.00306%	15.20%
Tumor tissue	SH3 and PX domain-containing protein 2B GN=SH3PXD2B PE=1 SV=3	SPD2B_HUMAN	101,580.90	100.00%	4	4	4	0.00306%	7.03%
Pap test	SH3 domain-binding glutamic acid-rich-like protein GN=SH3BGRL PE=1 SV=1	SH3L1_HUMAN	12,774.50	100.00%	4	5	7	0.01090%	46.50%
Swab	SH3 domain-binding glutamic acid-rich-like protein GN=SH3BGRL PE=1 SV=1	SH3L1_HUMAN	12,774.50	100.00%	3	3	5	0.00354%	36.00%
Tumor tissue	SH3 domain-binding glutamic acid-rich-like protein GN=SH3BGRL PE=1 SV=1	SH3L1_HUMAN	12,774.50	100.00%	7	11	21	0.01610%	61.40%

Biological sample	Protein name	Protein accession numbers	Protein molecular weight (Da)	Protein identification probability	Exclusive unique peptide count	Exclusive unique spectrum count	Total spectrum count	Percentage of total spectra	Percentage sequence coverage
Pap test	SH3 domain-binding glutamic acid-rich-like protein 2 GN=SH3BGRL2 PE=1 SV=2	SH3L2_HUMAN	12,326.30	99.40%	1	1	1	0.00156%	11.20%
Swab	SH3 domain-binding glutamic acid-rich-like protein 2 GN=SH3BGRL2 PE=1 SV=2	SH3L2_HUMAN	12,326.30	99.80%	1	1	1	0.00071%	11.20%
Tumor tissue	SH3 domain-binding glutamic acid-rich-like protein 2 GN=SH3BGRL2 PE=1 SV=2	SH3L2_HUMAN	12,326.30	100.00%	2	2	3	0.00230%	25.20%
Pap test	SH3 domain-binding glutamic acid-rich-like protein 3 GN=SH3BGRL3 PE=1 SV=1	Q5T123_HUMAN	9,380.50	100.00%	2	2	2	0.00312%	33.00%
Swab	SH3 domain-binding glutamic acid-rich-like protein 3 GN=SH3BGRL3 PE=1 SV=1	Q5T123_HUMAN	9,380.50	100.00%	2	3	6	0.00425%	21.60%
Tumor tissue	SH3 domain-binding glutamic acid-rich-like protein 3 GN=SH3BGRL3 PE=1 SV=1	Q5T123_HUMAN	9,380.50	100.00%	3	5	6	0.00459%	33.00%
Tumor tissue	SH3 domain-binding protein 1 GN=SH3BP1 PE=1 SV=3	sp|Q9Y3L3|3BP1_HUMAN	75,714.70	100.00%	6	6	6	0.00459%	9.99%
Tumor tissue	SH3 domain-containing kinase-binding protein 1 (Fragment) GN=SH3KBP1 PE=1 SV=1	sp|Q96B97|SH3K1_HUMAN	70,446.10	100.00%	6	6	6	0.00459%	15.00%
Tumor tissue	Short-chain specific acyl-CoA dehydrogenase, mitochondrial GN=ACADS PE=1 SV=1	ACADS_HUMAN	44,299.10	100.00%	3	3	3	0.00230%	11.20%
Tumor tissue	Sialate O-acetylesterase GN=SIAE PE=1 SV=1	sp|Q9HAT2|SIAE_HUMAN	58,315.30	100.00%	4	5	10	0.00766%	10.30%
Pap test	Sialic acid synthase GN=NANS PE=1 SV=2	SIAS_HUMAN	40,307.90	100.00%	3	3	3	0.00468%	12.00%
Swab	Sialic acid synthase GN=NANS PE=1 SV=2	SIAS_HUMAN	40,307.90	100.00%	7	10	23	0.01630%	25.30%
Tumor tissue	Sialic acid synthase GN=NANS PE=1 SV=2	SIAS_HUMAN	40,307.90	100.00%	6	9	12	0.00919%	22.30%
Tumor tissue	Sickle tail protein homolog GN=KIAA1217 PE=1 SV=2	sp|Q5T5P2|SKT_HUMAN	214,116.70	100.00%	4	4	29	0.02220%	15.90%

Biological sample	Protein name	Protein accession numbers	Protein molecular weight (Da)	Protein identification probability	Exclusive unique peptide count	Exclusive unique spectrum count	Total spectrum count	Percentage of total spectra	Percentage sequence coverage
Tumor tissue	Sideroflexin GN=SFXN3 PE=1 SV=1	SFXN3_HUMAN	35,503.50	100.00%	4	5	14	0.01070%	42.40%
Tumor tissue	Sideroflexin-1 GN=SFXN1 PE=1 SV=4	SFXN1_HUMAN	35,620.00	100.00%	5	5	6	0.00459%	22.40%
Tumor tissue	Signal peptidase complex catalytic subunit SEC11 GN=SEC11A PE=1 SV=1	sp|P67812|SC11A_HUMAN	18,651.00	100.00%	4	6	9	0.00689%	17.80%
Tumor tissue	Signal peptidase complex catalytic subunit SEC11C GN=SEC11C PE=1 SV=3	SC11C_HUMAN	21,542.90	100.00%	4	7	8	0.00612%	18.80%
Tumor tissue	Signal peptidase complex subunit 1 GN=SPCS1 PE=1 SV=4	SPCS1_HUMAN	11,805.30	100.00%	1	4	10	0.00766%	31.40%
Tumor tissue	Signal peptidase complex subunit 2 GN=SPCS2 PE=1 SV=1	SPCS2_HUMAN	17,028.20	100.00%	4	6	8	0.00612%	20.70%
Tumor tissue	Signal peptidase complex subunit 3 GN=SPCS3 PE=1 SV=1	SPCS3_HUMAN	20,314.20	100.00%	2	3	7	0.00536%	12.80%
Tumor tissue	Signal recognition particle 14 kDa protein GN=SRP14 PE=1 SV=2	SRP14_HUMAN	14,570.60	100.00%	6	7	9	0.00689%	52.20%
Tumor tissue	Signal recognition particle 54 kDa protein GN=SRP54 PE=1 SV=1	sp|P61011|SRP54_HUMAN	55,706.30	100.00%	8	8	8	0.00612%	18.50%
Tumor tissue	Signal recognition particle receptor subunit alpha GN=SRPRA PE=1 SV=2	sp|P08240|SRPRA_HUMAN	69,812.80	100.00%	10	14	16	0.01220%	20.80%
Tumor tissue	Signal recognition particle receptor subunit beta GN=SRPRB PE=1 SV=3	SRPRB_HUMAN	29,703.10	100.00%	4	6	6	0.00459%	22.50%
Tumor tissue	Signal recognition particle subunit SRP68 GN=SRP68 PE=1 SV=2	sp|Q9UHB9|SRP68_HUMAN	70,732.00	100.00%	12	14	17	0.01300%	23.40%
Tumor tissue	Signal recognition particle subunit SRP72 GN=SRP72 PE=1 SV=3	sp|O76094|SRP72_HUMAN	74,608.30	100.00%	10	11	12	0.00919%	19.80%
Tumor tissue	Signal transducer and activator of transcription GN=STAT5A PE=1 SV=1	sp|P42229|STA5A_HUMAN	87,363.30	100.00%	1	1	4	0.00306%	6.95%
Tumor tissue	Signal transducer and activator of transcription 1-alpha/beta GN=STAT1 PE=1 SV=2	sp|P42224|STAT1_HUMAN	87,336.90	100.00%	2	3	42	0.03220%	39.30%

Biological sample	Protein name	Protein accession numbers	Protein molecular weight (Da)	Protein identification probability	Exclusive unique peptide count	Exclusive unique spectrum count	Total spectrum count	Percentage of total spectra	Percentage sequence coverage
Tumor tissue	Signal transducer and activator of transcription 5B GN=STAT5B PE=1 SV=2	STA5B_HUMAN	89,866.50	100.00%	1	2	5	0.00383%	6.48%
Tumor tissue	Signal transducer and activator of transcription 6 GN=STAT6 PE=1 SV=1	sp|P42226|STAT6_HUMAN	94,136.90	100.00%	3	4	4	0.00306%	6.38%
Tumor tissue	Signal-induced proliferation-associated 1-like protein 1 GN=SIPA1L1 PE=1 SV=4	sp|O43166|SI1L1_HUMAN	200,032.20	100.00%	4	4	5	0.00383%	3.10%
Tumor tissue	Signal-induced proliferation-associated protein 1 GN=SIPA1 PE=1 SV=1	SIPA1_HUMAN	101,825.20	100.00%	2	2	2	0.00153%	3.09%
Pap test	Single-stranded DNA-binding protein, mitochondrial GN=SSBP1 PE=1 SV=1	SSBP_HUMAN	17,259.70	100.00%	2	2	2	0.00312%	22.30%
Tumor tissue	Single-stranded DNA-binding protein, mitochondrial GN=SSBP1 PE=1 SV=1	SSBP_HUMAN	17,259.70	100.00%	7	10	12	0.00919%	47.30%
Tumor tissue	Sister chromatid cohesion protein PDS5 homolog A GN=PDS5A PE=1 SV=1	sp|Q29RF7|PDS5A_HUMAN	150,835.50	100.00%	7	7	7	0.00536%	7.03%
Tumor tissue	SKNY protein GN=SMPD4 PE=1 SV=1	sp|Q9NXE4|NSMA3_HUMAN	97,812.90	100.00%	1	2	4	0.00306%	5.31%
Tumor tissue	SLIT-ROBO Rho GTPase-activating protein 2 GN=SRGAP2 PE=1 SV=1	SRGP2_HUMAN	120,872.50	100.00%	1	1	2	0.00153%	2.79%
Tumor tissue	Small acidic protein GN=C11orf58 PE=1 SV=2	E9PM92_HUMAN	17,634.80	100.00%	2	2	3	0.00230%	24.80%
Pap test	Small glutamine-rich tetratricopeptide repeat-containing protein alpha GN=SGTA PE=1 SV=1	SGTA_HUMAN	34,063.90	99.20%	1	1	1	0.00156%	3.83%
Tumor tissue	Small glutamine-rich tetratricopeptide repeat-containing protein alpha GN=SGTA PE=1 SV=1	SGTA_HUMAN	34,063.90	100.00%	3	4	6	0.00459%	11.20%
Tumor tissue	Small membrane A-kinase anchor protein GN=C2orf88 PE=1 SV=2	SMAKA_HUMAN	10,970.40	100.00%	2	2	2	0.00153%	26.30%

Biological sample	Protein name	Protein accession numbers	Protein molecular weight (Da)	Protein identification probability	Exclusive unique peptide count	Exclusive unique spectrum count	Total spectrum count	Percentage of total spectra	Percentage sequence coverage
Pap test	Small nuclear ribonucleoprotein E GN=SNRPE PE=1 SV=1	RUXE_HUMAN	10,803.90	100.00%	2	2	3	0.00468%	25.00%
Tumor tissue	Small nuclear ribonucleoprotein E GN=SNRPE PE=1 SV=1	RUXE_HUMAN	10,803.90	99.90%	1	2	6	0.00459%	12.00%
Tumor tissue	Small nuclear ribonucleoprotein Sm D1 GN=SNRPD1 PE=1 SV=1	SMD1_HUMAN	13,281.90	100.00%	3	7	10	0.00766%	37.80%
Pap test	Small nuclear ribonucleoprotein Sm D2 GN=SNRPD2 PE=1 SV=1	sp|P62316|SMD2_HUMAN	13,527.10	100.00%	2	2	4	0.00624%	32.20%
Tumor tissue	Small nuclear ribonucleoprotein Sm D2 GN=SNRPD2 PE=1 SV=1	sp|P62316|SMD2_HUMAN	13,527.10	100.00%	3	5	11	0.00842%	32.20%
Pap test	Small nuclear ribonucleoprotein Sm D3 GN=SNRPD3 PE=1 SV=1	sp|P62318|SMD3_HUMAN	13,916.60	100.00%	2	2	5	0.00780%	15.10%
Tumor tissue	Small nuclear ribonucleoprotein Sm D3 GN=SNRPD3 PE=1 SV=1	sp|P62318|SMD3_HUMAN	13,916.60	100.00%	2	2	3	0.00230%	15.10%
Tumor tissue	Small nuclear ribonucleoprotein-associated protein N (Fragment) GN=SNRPN PE=1 SV=1	J3QLE5_HUMAN	17,546.50	100.00%	1	1	8	0.00612%	43.80%
Pap test	Small proline-rich protein 3 GN=SPRR3 PE=1 SV=2	SPRR3_HUMAN	18,153.00	100.00%	8	15	45	0.07020%	52.10%
Swab	Small proline-rich protein 3 GN=SPRR3 PE=1 SV=2	SPRR3_HUMAN	18,153.00	100.00%	7	9	24	0.01700%	52.10%
Tumor tissue	Small ubiquitin-related modifier 1 GN=SUMO1 PE=1 SV=1	sp|P63165|SUMO1_HUMAN	16,644.90	100.00%	2	2	2	0.00153%	14.40%
Pap test	S-methyl-5'-thioadenosine phosphorylase GN=MTAP PE=1 SV=1	sp|Q13126|MTAP_HUMAN	33,186.70	100.00%	8	10	15	0.02340%	40.70%
Swab	S-methyl-5'-thioadenosine phosphorylase GN=MTAP PE=1 SV=1	sp|Q13126|MTAP_HUMAN	33,186.70	100.00%	2	2	2	0.00142%	11.70%
Tumor tissue	S-methyl-5'-thioadenosine phosphorylase GN=MTAP PE=1 SV=1	sp|Q13126|MTAP_HUMAN	33,186.70	100.00%	3	3	3	0.00230%	17.30%
Tumor tissue	SNW domain-containing protein 1 GN=SNW1 PE=1 SV=1	SNW1_HUMAN	61,495.90	100.00%	1	1	4	0.00306%	9.70%

Biological sample	Protein name	Protein accession numbers	Protein molecular weight (Da)	Protein identification probability	Exclusive unique peptide count	Exclusive unique spectrum count	Total spectrum count	Percentage of total spectra	Percentage sequence coverage
Tumor tissue	Sodium/potassium-transporting ATPase subunit alpha-3 GN=ATP1A3 PE=1 SV=1	A0A0A0MT26_HUMAN	133,321.30	100.00%	2	2	17	0.01300%	10.80%
Tumor tissue	Sodium/potassium-transporting ATPase subunit beta-3 GN=ATP1B3 PE=1 SV=1	sp|P54709|AT1B3_HUMAN	31,513.70	100.00%	3	5	8	0.00612%	21.10%
Tumor tissue	Solute carrier family 12 member 7 GN=SLC12A7 PE=1 SV=1	A0A0G2JNW7_HUMAN	119,138.10	100.00%	2	2	3	0.00230%	4.71%
Tumor tissue	Solute carrier family 2, facilitated glucose transporter member 1 GN=SLC2A1 PE=1 SV=2	GTR1_HUMAN	54,085.30	100.00%	5	8	14	0.01070%	12.20%
Tumor tissue	Solute carrier family 2, facilitated glucose transporter member 3 GN=SLC2A3 PE=1 SV=1	GTR3_HUMAN	53,926.00	100.00%	2	2	2	0.00153%	5.85%
Tumor tissue	Solute carrier family 35 member E1 GN=SLC35E1 PE=1 SV=2	sp|Q96K37|S35E1_HUMAN	44,773.50	100.00%	2	2	2	0.00153%	4.88%
Pap test	Sorbitol dehydrogenase GN=SORD PE=1 SV=4	sp|Q00796|DHSO_HUMAN	38,324.60	100.00%	2	3	3	0.00468%	7.28%
Swab	Sorbitol dehydrogenase GN=SORD PE=1 SV=4	sp|Q00796|DHSO_HUMAN	38,324.60	100.00%	5	7	10	0.00709%	22.70%
Tumor tissue	Sorbitol dehydrogenase GN=SORD PE=1 SV=4	sp|Q00796|DHSO_HUMAN	38,324.60	100.00%	5	7	8	0.00612%	18.80%
Tumor tissue	Sorcin GN=SRI PE=1 SV=1	C9J0K6_HUMAN	17,605.30	100.00%	4	6	8	0.00612%	25.20%
Tumor tissue	Sorting nexin GN=SNX6 PE=1 SV=1	sp|Q9UNH7|SNX6_HUMAN	47,805.90	100.00%	7	10	15	0.01150%	20.80%
Tumor tissue	Sorting nexin-1 GN=SNX1 PE=1 SV=3	sp|Q13596|SNX1_HUMAN	59,070.80	100.00%	10	13	14	0.01070%	23.80%
Swab	Sorting nexin-12 GN=SNX12 PE=1 SV=1	sp|Q9UMY4|SNX12_HUMAN	19,826.10	100.00%	3	3	4	0.00283%	19.80%
Tumor tissue	Sorting nexin-12 GN=SNX12 PE=1 SV=1	sp|Q9UMY4|SNX12_HUMAN	19,826.10	100.00%	4	5	8	0.00612%	27.30%
Tumor tissue	Sorting nexin-17 GN=SNX17 PE=1 SV=1	sp|Q15036|SNX17_HUMAN	52,901.60	100.00%	4	4	4	0.00306%	12.10%
Tumor tissue	Sorting nexin-2 GN=SNX2 PE=1 SV=2	sp|O60749|SNX2_HUMAN	58,472.20	100.00%	12	14	17	0.01300%	29.50%

Biological sample	Protein name	Protein accession numbers	Protein molecular weight (Da)	Protein identification probability	Exclusive unique peptide count	Exclusive unique spectrum count	Total spectrum count	Percentage of total spectra	Percentage sequence coverage
Tumor tissue	Sorting nexin-27 GN=SNX27 PE=1 SV=2	sp|Q96L92|SNX27_HUMAN	61,264.90	100.00%	4	4	4	0.00306%	9.06%
Swab	Sorting nexin-3 GN=SNX3 PE=1 SV=3	sp|O60493|SNX3_HUMAN	18,763.00	100.00%	3	3	4	0.00283%	21.00%
Tumor tissue	Sorting nexin-3 GN=SNX3 PE=1 SV=3	sp|O60493|SNX3_HUMAN	18,763.00	100.00%	6	6	7	0.00536%	35.20%
Tumor tissue	Sorting nexin-30 GN=SNX30 PE=1 SV=1	SNX30_HUMAN	49,678.60	100.00%	2	2	2	0.00153%	4.81%
Tumor tissue	Sorting nexin-33 GN=SNX33 PE=1 SV=1	SNX33_HUMAN	65,265.70	100.00%	3	3	3	0.00230%	8.54%
Tumor tissue	Sorting nexin-4 GN=SNX4 PE=1 SV=1	sp|O95219|SNX4_HUMAN	51,909.80	100.00%	3	3	5	0.00383%	8.22%
Tumor tissue	Sorting nexin-5 GN=SNX5 PE=1 SV=1	SNX5_HUMAN	46,817.80	100.00%	5	5	6	0.00459%	17.10%
Tumor tissue	Sorting nexin-9 GN=SNX9 PE=1 SV=1	SNX9_HUMAN	66,592.80	100.00%	7	8	8	0.00612%	17.30%
Tumor tissue	SPARC (Fragment) GN=SPARC PE=1 SV=1	SPRC_HUMAN	17,470.30	100.00%	2	3	3	0.00230%	9.40%
Tumor tissue	Spartin GN=SPG20 PE=1 SV=1	SPG20_HUMAN	72,832.50	100.00%	4	4	4	0.00306%	8.56%
Tumor tissue	Spectrin alpha chain, non-erythrocytic 1 GN=SPTAN1 PE=1 SV=1	A0A0D9SF54_HUMAN	282,840.00	100.00%	2	2	121	0.09260%	42.30%
Pap test	Spectrin alpha chain, non-erythrocytic 1 GN=SPTAN1 PE=1 SV=3	sp|Q13813|SPTN1_HUMAN	284,542.70	100.00%	1	1	32	0.04990%	14.60%
Tumor tissue	Spectrin alpha chain, non-erythrocytic 1 GN=SPTAN1 PE=1 SV=3	sp|Q13813|SPTN1_HUMAN	284,542.70	100.00%	2	3	122	0.09340%	41.90%
Tumor tissue	Spectrin beta chain, non-erythrocytic 1 GN=SPTBN1 PE=1 SV=1	A0A087WUZ3_HUMAN	274,833.80	100.00%	5	5	95	0.07270%	38.00%
Tumor tissue	Spermatogenesis-associated protein 5 GN=SPATA5 PE=1 SV=3	sp|Q8NB90|SPAT5_HUMAN	97,908.50	100.00%	1	1	2	0.00153%	2.69%
Swab	Spermidine synthase GN=SRM PE=1 SV=1	SPEE_HUMAN	33,824.50	99.20%	1	1	1	0.00071%	4.30%

Biological sample	Protein name	Protein accession numbers	Protein molecular weight (Da)	Protein identification probability	Exclusive unique peptide count	Exclusive unique spectrum count	Total spectrum count	Percentage of total spectra	Percentage sequence coverage
Tumor tissue	Spermidine synthase GN=SRM PE=1 SV=1	SPEE_HUMAN	33,824.50	100.00%	6	6	6	0.00459%	21.50%
Pap test	S-phase kinase-associated protein 1 GN=SKP1 PE=1 SV=1	sp|P63208|SKP1_HUMAN	18,720.40	100.00%	1	1	3	0.00468%	7.98%
Swab	S-phase kinase-associated protein 1 GN=SKP1 PE=1 SV=1	sp|P63208|SKP1_HUMAN	18,720.40	100.00%	2	2	3	0.00213%	8.59%
Tumor tissue	S-phase kinase-associated protein 1 GN=SKP1 PE=1 SV=1	sp|P63208|SKP1_HUMAN	18,720.40	100.00%	3	4	4	0.00306%	19.00%
Tumor tissue	Sphingosine-1-phosphate lyase 1 GN=SGPL1 PE=1 SV=3	SGPL1_HUMAN	63,525.90	100.00%	4	4	4	0.00306%	8.27%
Tumor tissue	Spliceosome-associated protein CWC15 homolog GN=CWC15 PE=1 SV=2	CWC15_HUMAN	26,625.20	100.00%	2	2	2	0.00153%	14.40%
Tumor tissue	Splicing factor 3A subunit 1 GN=SF3A1 PE=1 SV=1	sp|Q15459|SF3A1_HUMAN	88,886.10	100.00%	11	13	17	0.01300%	22.70%
Tumor tissue	Splicing factor 3A subunit 2 GN=SF3A2 PE=1 SV=2	SF3A2_HUMAN	49,254.50	100.00%	3	3	6	0.00459%	13.40%
Tumor tissue	Splicing factor 3A subunit 3 GN=SF3A3 PE=1 SV=1	SF3A3_HUMAN	58,851.30	100.00%	8	8	10	0.00766%	22.80%
Tumor tissue	Splicing factor 3B subunit 1 GN=SF3B1 PE=1 SV=3	sp|O75533|SF3B1_HUMAN	145,835.30	100.00%	26	34	40	0.03060%	27.70%
Tumor tissue	Splicing factor 3B subunit 2 GN=SF3B2 PE=1 SV=2	SF3B2_HUMAN	100,229.40	100.00%	8	11	22	0.01680%	25.80%
Tumor tissue	Splicing factor 3B subunit 3 GN=SF3B3 PE=1 SV=4	sp|Q15393|SF3B3_HUMAN	135,579.40	100.00%	18	20	27	0.02070%	22.80%
Tumor tissue	Splicing factor 3B subunit 5 GN=SF3B5 PE=1 SV=1	SF3B5_HUMAN	10,135.50	100.00%	3	5	5	0.00383%	38.40%
Tumor tissue	Splicing factor 3B subunit 6 GN=SF3B6 PE=1 SV=1	SF3B6_HUMAN	14,585.60	100.00%	2	2	4	0.00306%	20.80%
Tumor tissue	Splicing factor U2AF 35 kDa subunit GN=U2AF1 PE=1 SV=3	sp|Q01081|U2AF1_HUMAN	27,872.20	100.00%	2	3	3	0.00230%	12.90%
Tumor tissue	Splicing factor, arginine/serine-rich 19 GN=SCAF1 PE=1 SV=3	SFR19_HUMAN	139,272.90	100.00%	2	2	2	0.00153%	1.91%
Pap test	Splicing factor, proline- and glutamine-rich GN=SFPQ PE=1 SV=2	sp|P23246|SFPQ_HUMAN	76,149.50	100.00%	1	1	4	0.00624%	7.21%

Biological sample	Protein name	Protein accession numbers	Protein molecular weight (Da)	Protein identification probability	Exclusive unique peptide count	Exclusive unique spectrum count	Total spectrum count	Percentage of total spectra	Percentage sequence coverage
Tumor tissue	Splicing factor, proline- and glutamine-rich GN=SFPQ PE=1 SV=2	sp|P23246|SFPQ_HUMAN	76,149.50	100.00%	3	3	47	0.03600%	32.70%
Tumor tissue	Spondin-1 GN=SPON1 PE=1 SV=2	SPON1_HUMAN	90,973.60	100.00%	8	10	16	0.01220%	13.00%
Tumor tissue	Squalene monooxygenase GN=SQLE PE=1 SV=3	ERG1_HUMAN	63,925.00	100.00%	7	8	8	0.00612%	20.00%
Pap test	Src substrate cortactin GN=CTTN PE=1 SV=2	sp|Q14247|SRC8_HUMAN	61,585.50	100.00%	1	1	3	0.00468%	4.73%
Swab	Src substrate cortactin GN=CTTN PE=1 SV=2	sp|Q14247|SRC8_HUMAN	61,585.50	100.00%	1	1	5	0.00354%	17.10%
Tumor tissue	Src substrate cortactin GN=CTTN PE=1 SV=2	sp|Q14247|SRC8_HUMAN	61,585.50	100.00%	3	3	25	0.01910%	32.90%
Tumor tissue	SRSF protein kinase 1 (Fragment) GN=SRPK1 PE=1 SV=1	sp|Q96SB4-3|SRPK1_HUMAN	76,040.80	100.00%	2	2	2	0.00153%	4.77%
Tumor tissue	Stabilin-1 GN=STAB1 PE=1 SV=3	sp|Q9NY15|STAB1_HUMAN	275,473.10	100.00%	6	6	6	0.00459%	3.11%
Tumor tissue	Staphylococcal nuclease domain-containing protein 1 GN=SND1 PE=1 SV=1	SND1_HUMAN	101,998.50	100.00%	31	44	62	0.04750%	38.60%
Pap test	StAR-related lipid transfer protein 5 GN=STARD5 PE=1 SV=2	sp|Q9NSY2|STAR5_HUMAN	23,793.80	100.00%	1	1	2	0.00312%	11.70%
Tumor tissue	Stathmin GN=STMN1 PE=1 SV=3	sp|P16949|STMN1_HUMAN	17,303.00	100.00%	4	6	7	0.00536%	30.20%
Tumor tissue	Sterile alpha and TIR motif-containing protein 1 GN=SARM1 PE=1 SV=1	sp|Q6SZW1|SARM1_HUMAN	79,389.80	100.00%	1	1	2	0.00153%	4.28%
Tumor tissue	Sterile alpha motif domain-containing protein 9 GN=SAMD9 PE=1 SV=1	SAMD9_HUMAN	184,289.80	100.00%	2	2	2	0.00153%	1.64%
Pap test	Sterol-4-alpha-carboxylate 3-dehydrogenase, decarboxylating GN=NSDHL PE=1 SV=2	NSDHL_HUMAN	41,900.80	99.20%	1	1	1	0.00156%	3.49%
Tumor tissue	Sterol-4-alpha-carboxylate 3-dehydrogenase, decarboxylating GN=NSDHL PE=1 SV=2	NSDHL_HUMAN	41,900.80	100.00%	2	3	4	0.00306%	6.43%

Biological sample	Protein name	Protein accession numbers	Protein molecular weight (Da)	Protein identification probability	Exclusive unique peptide count	Exclusive unique spectrum count	Total spectrum count	Percentage of total spectra	Percentage sequence coverage
Tumor tissue	Stomatin-like protein 2, mitochondrial GN=STOML2 PE=1 SV=1	sp|Q9UJZ1|STML2_HUMAN	38,534.50	100.00%	2	3	20	0.01530%	35.10%
Pap test	Stress-70 protein, mitochondrial GN=HSPA9 PE=1 SV=2	GRP75_HUMAN	73,681.30	100.00%	3	3	3	0.00468%	6.04%
Swab	Stress-70 protein, mitochondrial GN=HSPA9 PE=1 SV=2	GRP75_HUMAN	73,681.30	100.00%	6	6	7	0.00496%	10.80%
Tumor tissue	Stress-70 protein, mitochondrial GN=HSPA9 PE=1 SV=2	GRP75_HUMAN	73,681.30	100.00%	23	37	68	0.05210%	42.70%
Pap test	Stress-induced-phosphoprotein 1 GN=STIP1 PE=1 SV=1	sp|P31948|STIP1_HUMAN	62,642.10	100.00%	8	10	12	0.01870%	16.80%
Swab	Stress-induced-phosphoprotein 1 GN=STIP1 PE=1 SV=1	sp|P31948|STIP1_HUMAN	62,642.10	100.00%	9	10	17	0.01200%	20.80%
Tumor tissue	Stress-induced-phosphoprotein 1 GN=STIP1 PE=1 SV=1	sp|P31948|STIP1_HUMAN	62,642.10	100.00%	18	22	27	0.02070%	33.00%
Tumor tissue	Striatin-interacting protein 1 GN=STRIP1 PE=1 SV=1	sp|Q5VSL9|STRP1_HUMAN	95,579.00	100.00%	1	1	2	0.00153%	2.39%
Tumor tissue	Stromal cell-derived factor 2 GN=SDF2 PE=1 SV=2	SDF2_HUMAN	23,025.00	100.00%	3	4	5	0.00383%	29.40%
Tumor tissue	Stromal cell-derived factor 2-like protein 1 GN=SDF2L1 PE=1 SV=2	SDF2L_HUMAN	23,597.50	100.00%	4	5	5	0.00383%	38.00%
Tumor tissue	Stromelysin-3 GN=MMP11 PE=1 SV=3	MMP11_HUMAN	54,590.90	100.00%	3	3	4	0.00306%	8.40%
Tumor tissue	Structural maintenance of chromosomes flexible hinge domain-containing protein 1 GN=SMCHD1 PE=1 SV=2	sp|A6NHR9|SMHD1_HUMAN	226,380.00	100.00%	10	10	10	0.00766%	5.44%
Swab	Structural maintenance of chromosomes protein GN=SMC4 PE=1 SV=1	sp|Q9NTJ3|SMC4_HUMAN	144,460.80	99.70%	1	1	1	0.00071%	1.82%
Tumor tissue	Structural maintenance of chromosomes protein GN=SMC4 PE=1 SV=1	sp|Q9NTJ3|SMC4_HUMAN	144,460.80	100.00%	2	3	3	0.00230%	1.98%
Tumor tissue	Structural maintenance of chromosomes protein 1A GN=SMC1A PE=1 SV=2	SMC1A_HUMAN	143,238.30	100.00%	16	20	22	0.01680%	17.50%

Biological sample	Protein name	Protein accession numbers	Protein molecular weight (Da)	Protein identification probability	Exclusive unique peptide count	Exclusive unique spectrum count	Total spectrum count	Percentage of total spectra	Percentage sequence coverage
Tumor tissue	Structural maintenance of chromosomes protein 2 GN=SMC2 PE=1 SV=2	sp|O95347|SMC2_HUMAN	135,660.80	100.00%	7	7	7	0.00536%	7.52%
Tumor tissue	Structural maintenance of chromosomes protein 3 GN=SMC3 PE=1 SV=2	SMC3_HUMAN	141,547.10	100.00%	23	29	31	0.02370%	23.60%
Tumor tissue	Succinate dehydrogenase [ubiquinone] flavoprotein subunit, mitochondrial GN=SDHA PE=1 SV=2	sp|P31040|SDHA_HUMAN	72,691.80	100.00%	10	13	27	0.02070%	25.00%
Pap test	Succinate dehydrogenase [ubiquinone] iron-sulfur subunit, mitochondrial GN=SDHB PE=1 SV=3	SDHB_HUMAN	31,631.00	99.20%	1	1	1	0.00156%	5.00%
Tumor tissue	Succinate dehydrogenase [ubiquinone] iron-sulfur subunit, mitochondrial GN=SDHB PE=1 SV=3	SDHB_HUMAN	31,631.00	100.00%	8	10	11	0.00842%	29.60%
Pap test	Succinate--CoA ligase [ADP/GDP-forming] subunit alpha, mitochondrial GN=SUCLG1 PE=1 SV=4	SUCA_HUMAN	36,250.20	100.00%	2	2	2	0.00312%	7.23%
Swab	Succinate--CoA ligase [ADP/GDP-forming] subunit alpha, mitochondrial GN=SUCLG1 PE=1 SV=4	SUCA_HUMAN	36,250.20	100.00%	3	3	3	0.00213%	10.70%
Tumor tissue	Succinate--CoA ligase [ADP/GDP-forming] subunit alpha, mitochondrial GN=SUCLG1 PE=1 SV=4	SUCA_HUMAN	36,250.20	100.00%	5	10	14	0.01070%	21.70%
Tumor tissue	Succinate--CoA ligase [GDP-forming] subunit beta, mitochondrial GN=SUCLG2 PE=1 SV=2	sp|Q96I99|SUCB2_HUMAN	46,511.70	100.00%	2	2	19	0.01450%	33.30%
Tumor tissue	Succinyl-CoA:3-ketoacid coenzyme A transferase 1, mitochondrial GN=OXCT1 PE=1 SV=1	sp|P55809|SCOT1_HUMAN	56,158.80	100.00%	2	2	4	0.00306%	11.20%
Tumor tissue	Sulfatase-modifying factor 2 GN=SUMF2 PE=1 SV=1	sp|Q8NBJ7|SUMF2_HUMAN	35,922.80	100.00%	1	1	10	0.00766%	25.00%

Biological sample	Protein name	Protein accession numbers	Protein molecular weight (Da)	Protein identification probability	Exclusive unique peptide count	Exclusive unique spectrum count	Total spectrum count	Percentage of total spectra	Percentage sequence coverage
Pap test	Sulfhydryl oxidase 1 GN=QSOX1 PE=1 SV=3	sp|O00391|QSOX1_HUMAN	82,578.70	100.00%	5	5	9	0.01400%	10.40%
Swab	Sulfhydryl oxidase 1 GN=QSOX1 PE=1 SV=3	sp|O00391|QSOX1_HUMAN	82,578.70	100.00%	3	3	5	0.00354%	5.76%
Tumor tissue	Sulfhydryl oxidase 1 GN=QSOX1 PE=1 SV=3	sp|O00391|QSOX1_HUMAN	82,578.70	100.00%	2	2	4	0.00306%	6.83%
Pap test	Sulfide:quinone oxidoreductase, mitochondrial GN=SQRDL PE=1 SV=1	SQRD_HUMAN	49,962.00	100.00%	4	4	4	0.00624%	11.10%
Tumor tissue	Sulfide:quinone oxidoreductase, mitochondrial GN=SQRDL PE=1 SV=1	SQRD_HUMAN	49,962.00	100.00%	12	17	22	0.01680%	36.90%
Tumor tissue	Sulfite oxidase, mitochondrial GN=SUOX PE=1 SV=2	SUOX_HUMAN	60,281.70	100.00%	3	3	3	0.00230%	8.07%
Swab	SUMO-activating enzyme subunit 1 GN=SAE1 PE=1 SV=1	sp|Q9UBE0|SAE1_HUMAN	38,450.10	99.60%	1	1	2	0.00142%	5.78%
Tumor tissue	SUMO-activating enzyme subunit 2 GN=UBA2 PE=1 SV=2	sp|Q9UBT2|SAE2_HUMAN	71,225.20	100.00%	3	4	6	0.00459%	9.84%
Swab	SUMO-conjugating enzyme GN=UBE2I PE=1 SV=1	UBC9_HUMAN	20,458.30	100.00%	3	3	4	0.00283%	16.80%
Tumor tissue	SUMO-conjugating enzyme GN=UBE2I PE=1 SV=1	UBC9_HUMAN	20,458.30	100.00%	5	7	9	0.00689%	20.10%
Tumor tissue	SUN domain-containing protein 1 GN=SUN1 PE=1 SV=1	sp|O94901-9|SUN1_HUMAN	91,113.20	100.00%	4	5	6	0.00459%	10.60%
Tumor tissue	Superkiller viralicidic activity 2-like 2 GN=SKIV2L2 PE=1 SV=3	SK2L2_HUMAN	117,808.30	100.00%	6	7	7	0.00536%	8.16%
Pap test	Superoxide dismutase [Cu-Zn] GN=SOD1 PE=1 SV=2	SODC_HUMAN	15,935.30	100.00%	3	5	8	0.01250%	32.50%
Swab	Superoxide dismutase [Cu-Zn] GN=SOD1 PE=1 SV=2	SODC_HUMAN	15,935.30	100.00%	4	5	12	0.00850%	32.50%
Tumor tissue	Superoxide dismutase [Cu-Zn] GN=SOD1 PE=1 SV=2	SODC_HUMAN	15,935.30	100.00%	2	2	3	0.00230%	16.90%
Pap test	Superoxide dismutase [Mn], mitochondrial GN=SOD2 PE=1 SV=2	sp|P04179|SODM_HUMAN	24,722.60	100.00%	2	4	16	0.02500%	19.40%
Swab	Superoxide dismutase [Mn], mitochondrial GN=SOD2 PE=1 SV=2	sp|P04179|SODM_HUMAN	24,722.60	100.00%	1	1	7	0.00496%	13.10%

Biological sample	Protein name	Protein accession numbers	Protein molecular weight (Da)	Protein identification probability	Exclusive unique peptide count	Exclusive unique spectrum count	Total spectrum count	Percentage of total spectra	Percentage sequence coverage
Tumor tissue	Superoxide dismutase [Mn], mitochondrial GN=SOD2 PE=1 SV=2	sp|P04179|SODM_HUMAN	24,722.60	100.00%	5	11	66	0.05050%	59.00%
Tumor tissue	Surfeit 4 GN=SURF4 PE=1 SV=1	Q5T8U5_HUMAN	21,128.80	100.00%	1	2	5	0.00383%	14.50%
Tumor tissue	Sushi domain-containing protein 2 GN=SUSD2 PE=1 SV=1	SUSD2_HUMAN	90,207.50	100.00%	4	6	6	0.00459%	10.30%
Tumor tissue	SWI/SNF complex subunit SMARCC1 GN=SMARCC1 PE=1 SV=3	SMRC1_HUMAN	122,867.40	100.00%	3	4	11	0.00842%	8.87%
Tumor tissue	SWI/SNF related, matrix associated, actin dependent regulator of chromatin, subfamily b, member 1, isoform CRA_c GN=SMARCB1 PE=1 SV=1	sp|Q12824-2|SNF5_HUMAN	45,051.70	100.00%	3	3	4	0.00306%	12.40%
Tumor tissue	SWI/SNF-related matrix-associated actin-dependent regulator of chromatin subfamily A member 5 GN=SMARCA5 PE=1 SV=1	SMCA5_HUMAN	121,910.80	100.00%	8	9	13	0.00995%	13.30%
Tumor tissue	SWI/SNF-related matrix-associated actin-dependent regulator of chromatin subfamily E member 1 GN=SMARCE1 PE=1 SV=1	B4DGM3_HUMAN	44,770.50	100.00%	2	3	8	0.00612%	22.10%
Tumor tissue	Switch-associated protein 70 GN=SWAP70 PE=1 SV=1	SWP70_HUMAN	68,999.30	100.00%	3	3	4	0.00306%	10.40%
Tumor tissue	Symplekin GN=SYMPK PE=1 SV=2	sp|Q92797|SYMPK_HUMAN	141,153.50	100.00%	4	5	7	0.00536%	5.26%
Tumor tissue	Synapse-associated protein 1 GN=SYAP1 PE=1 SV=1	SYAP1_HUMAN	39,933.40	100.00%	2	2	2	0.00153%	9.94%
Tumor tissue	Synaptic functional regulator FMR1 GN=FMR1 PE=1 SV=1	sp|Q06787-9|FMR1_HUMAN	66,785.70	100.00%	3	4	5	0.00383%	8.93%
Pap test	Synaptic vesicle membrane protein VAT-1 homolog GN=VAT1 PE=1 SV=2	sp|Q99536|VAT1_HUMAN	41,919.80	100.00%	6	7	12	0.01870%	22.90%
Swab	Synaptic vesicle membrane protein VAT-1 homolog GN=VAT1 PE=1 SV=2	sp|Q99536|VAT1_HUMAN	41,919.80	100.00%	4	4	7	0.00496%	16.50%
Tumor tissue	Synaptic vesicle membrane protein VAT-1 homolog GN=VAT1 PE=1 SV=2	sp|Q99536|VAT1_HUMAN	41,919.80	100.00%	8	12	16	0.01220%	29.30%

Biological sample	Protein name	Protein accession numbers	Protein molecular weight (Da)	Protein identification probability	Exclusive unique peptide count	Exclusive unique spectrum count	Total spectrum count	Percentage of total spectra	Percentage sequence coverage
Swab	Synaptobrevin homolog YKT6 GN=YKT6 PE=1 SV=1	sp|O15498|YKT6_HUMAN	22,417.80	100.00%	1	1	2	0.00142%	8.59%
Tumor tissue	Synaptobrevin homolog YKT6 GN=YKT6 PE=1 SV=1	sp|O15498|YKT6_HUMAN	22,417.80	100.00%	5	6	6	0.00459%	34.80%
Tumor tissue	Synaptonemal complex protein SC65 GN=P3H4 PE=1 SV=1	SC65_HUMAN	50,382.70	100.00%	3	3	3	0.00230%	7.78%
Tumor tissue	Synaptopodin-2 GN=SYNPO2 PE=1 SV=2	sp|Q9UMS6|SYNP2_HUMAN	117,513.80	100.00%	1	1	21	0.01610%	18.00%
Tumor tissue	Synaptosomal-associated protein 29 GN=SNAP29 PE=1 SV=1	SNP29_HUMAN	28,971.20	100.00%	3	3	3	0.00230%	14.30%
Tumor tissue	Syntaxin-12 GN=STX12 PE=1 SV=1	STX12_HUMAN	31,642.70	100.00%	5	7	9	0.00689%	26.10%
Tumor tissue	Syntaxin-18 GN=STX18 PE=1 SV=1	STX18_HUMAN	35,420.90	100.00%	3	4	4	0.00306%	15.30%
Tumor tissue	Syntaxin-3 GN=STX3 PE=1 SV=1	A0A0J9YW33_HUMAN	32,942.20	100.00%	2	2	2	0.00153%	8.01%
Tumor tissue	Syntaxin-4 GN=STX4 PE=1 SV=2	sp|Q12846|STX4_HUMAN	34,179.70	100.00%	3	3	5	0.00383%	19.50%
Tumor tissue	Syntaxin-8 GN=STX8 PE=1 SV=2	STX8_HUMAN	26,907.60	100.00%	4	5	5	0.00383%	22.50%
Tumor tissue	Syntaxin-binding protein 1 GN=STXBP1 PE=1 SV=1	sp|P61764|STXB1_HUMAN	67,571.80	100.00%	4	4	4	0.00306%	9.26%
Tumor tissue	Syntaxin-binding protein 3 GN=STXBP3 PE=1 SV=2	STXB3_HUMAN	67,766.90	100.00%	4	4	4	0.00306%	7.60%
Pap test	Talin-1 GN=TLN1 PE=1 SV=3	TLN1_HUMAN	269,765.10	100.00%	14	15	20	0.03120%	10.10%
Swab	Talin-1 GN=TLN1 PE=1 SV=3	TLN1_HUMAN	269,765.10	100.00%	32	38	65	0.04610%	21.40%
Tumor tissue	Talin-1 GN=TLN1 PE=1 SV=3	TLN1_HUMAN	269,765.10	100.00%	63	116	208	0.15900%	37.90%
Tumor tissue	Talin-2 GN=TLN2 PE=1 SV=4	TLN2_HUMAN	271,611.00	100.00%	6	7	36	0.02760%	14.10%
Tumor tissue	Tapasin GN=TAPBP PE=1 SV=1	sp|O15533|TPSN_HUMAN	49,492.50	100.00%	4	7	13	0.00995%	11.10%
Pap test	TAR DNA binding protein, isoform CRA_d GN=TARDBP PE=1 SV=1	G3V162_HUMAN	33,730.10	99.30%	1	1	1	0.00156%	4.03%
Tumor tissue	TAR DNA binding protein, isoform CRA_d GN=TARDBP PE=1 SV=1	G3V162_HUMAN	33,730.10	100.00%	2	2	4	0.00306%	12.10%

Biological sample	Protein name	Protein accession numbers	Protein molecular weight (Da)	Protein identification probability	Exclusive unique peptide count	Exclusive unique spectrum count	Total spectrum count	Percentage of total spectra	Percentage sequence coverage
Tumor tissue	Target of Myb protein 1 GN=TOM1 PE=1 SV=2	sp|O60784|TOM1_HUMAN	53,819.10	100.00%	6	7	14	0.01070%	22.40%
Tumor tissue	Target of Nesh-SH3 GN=ABI3BP PE=1 SV=1	sp|Q7Z7G0|TARSH_HUMAN	195,325.50	100.00%	3	3	3	0.00230%	2.76%
Pap test	Tax1-binding protein 3 GN=TAX1BP3 PE=1 SV=2	TX1B3_HUMAN	13,734.60	99.20%	1	1	1	0.00156%	13.70%
Swab	Tax1-binding protein 3 GN=TAX1BP3 PE=1 SV=2	TX1B3_HUMAN	13,734.60	99.70%	1	1	2	0.00142%	13.70%
Tumor tissue	Tax1-binding protein 3 GN=TAX1BP3 PE=1 SV=2	TX1B3_HUMAN	13,734.60	100.00%	2	3	3	0.00230%	28.20%
Tumor tissue	TBC1 domain family member 10B GN=TBC1D10B PE=1 SV=3	sp|Q4KMP7|TB10B_HUMAN	87,199.30	100.00%	2	2	2	0.00153%	4.08%
Tumor tissue	T-cell surface glycoprotein CD4 GN=CD4 PE=1 SV=1	CD4_HUMAN	51,111.30	100.00%	2	2	2	0.00153%	5.24%
Pap test	T-complex protein 1 subunit alpha GN=TCP1 PE=1 SV=1	TCPA_HUMAN	60,345.20	100.00%	3	3	4	0.00624%	5.40%
Tumor tissue	T-complex protein 1 subunit alpha GN=TCP1 PE=1 SV=1	TCPA_HUMAN	60,345.20	100.00%	15	20	27	0.02070%	33.60%
Pap test	T-complex protein 1 subunit beta GN=CCT2 PE=1 SV=4	sp|P78371|TCPB_HUMAN	57,489.90	99.30%	1	1	1	0.00156%	2.24%
Swab	T-complex protein 1 subunit beta GN=CCT2 PE=1 SV=4	sp|P78371|TCPB_HUMAN	57,489.90	100.00%	3	3	4	0.00283%	8.04%
Tumor tissue	T-complex protein 1 subunit beta GN=CCT2 PE=1 SV=4	sp|P78371|TCPB_HUMAN	57,489.90	100.00%	21	30	35	0.02680%	44.30%
Pap test	T-complex protein 1 subunit delta GN=CCT4 PE=1 SV=4	sp|P50991|TCPD_HUMAN	57,926.00	100.00%	1	2	4	0.00624%	4.27%
Swab	T-complex protein 1 subunit delta GN=CCT4 PE=1 SV=4	sp|P50991|TCPD_HUMAN	57,926.00	99.80%	1	1	1	0.00071%	2.23%
Tumor tissue	T-complex protein 1 subunit delta GN=CCT4 PE=1 SV=4	sp|P50991|TCPD_HUMAN	57,926.00	100.00%	13	22	26	0.01990%	31.20%
Tumor tissue	T-complex protein 1 subunit epsilon GN=CCT5 PE=1 SV=1	sp|P48643|TCPE_HUMAN	59,672.40	100.00%	11	17	28	0.02140%	22.20%
Tumor tissue	T-complex protein 1 subunit eta GN=CCT7 PE=1 SV=2	sp|Q99832|TCPH_HUMAN	59,367.80	100.00%	15	19	23	0.01760%	37.90%

Biological sample	Protein name	Protein accession numbers	Protein molecular weight (Da)	Protein identification probability	Exclusive unique peptide count	Exclusive unique spectrum count	Total spectrum count	Percentage of total spectra	Percentage sequence coverage
Tumor tissue	T-complex protein 1 subunit gamma (Fragment) GN=CCT3 PE=1 SV=1	Q5SZX6_HUMAN	30,762.60	99.90%	1	1	7	0.00536%	19.20%
Swab	T-complex protein 1 subunit theta GN=CCT8 PE=1 SV=4	sp|P50990|TCPQ_HUMAN	59,621.00	100.00%	2	2	2	0.00142%	4.38%
Tumor tissue	T-complex protein 1 subunit theta GN=CCT8 PE=1 SV=4	sp|P50990|TCPQ_HUMAN	59,621.00	100.00%	15	20	25	0.01910%	34.10%
Swab	T-complex protein 1 subunit zeta GN=CCT6A PE=1 SV=3	sp|P40227|TCPZ_HUMAN	58,025.30	99.80%	1	1	1	0.00071%	4.71%
Tumor tissue	T-complex protein 1 subunit zeta GN=CCT6A PE=1 SV=3	sp|P40227|TCPZ_HUMAN	58,025.30	100.00%	6	8	13	0.00995%	18.10%
Tumor tissue	Telomeric repeat-binding factor 2 GN=TERF2 PE=1 SV=3	sp|Q15554|TERF2_HUMAN	59,594.90	100.00%	2	2	2	0.00153%	5.35%
Tumor tissue	Tenascin GN=TNC PE=1 SV=3	sp|P24821|TENA_HUMAN	240,850.10	100.00%	2	2	136	0.10400%	30.10%
Tumor tissue	Tenascin-X GN=TNXB PE=1 SV=1	sp|P22105|TENX_HUMAN	456,142.60	100.00%	4	4	16	0.01220%	5.21%
Tumor tissue	Tensin-1 GN=TNS1 PE=1 SV=1	sp|Q9HBL0|TENS1_HUMAN	184,174.00	100.00%	1	2	33	0.02530%	25.00%
Tumor tissue	Tensin-1 GN=TNS1 PE=1 SV=1	E9PGF5_HUMAN	183,223.80	100.00%	1	3	37	0.02830%	25.10%
Tumor tissue	Tensin-3 GN=TNS3 PE=1 SV=2	sp|Q68CZ2|TENS3_HUMAN	155,266.50	100.00%	4	4	7	0.00536%	6.02%
Tumor tissue	Testis-specific Y-encoded-like protein 5 GN=TSPYL5 PE=1 SV=2	TSYL5_HUMAN	45,144.10	100.00%	3	4	4	0.00306%	15.30%
Pap test	Tetranectin GN=CLEC3B PE=1 SV=1	TETN_HUMAN	17,794.20	100.00%	2	3	3	0.00468%	20.60%
Swab	Tetranectin GN=CLEC3B PE=1 SV=1	TETN_HUMAN	17,794.20	100.00%	7	8	16	0.01130%	60.00%
Tumor tissue	Tetranectin GN=CLEC3B PE=1 SV=1	TETN_HUMAN	17,794.20	100.00%	4	6	7	0.00536%	30.60%
Pap test	Tetraspanin GN=CD9 PE=1 SV=1	CD9_HUMAN	25,408.70	100.00%	2	2	3	0.00468%	6.29%
Swab	Tetraspanin GN=CD9 PE=1 SV=1	CD9_HUMAN	25,408.70	99.20%	1	1	1	0.00071%	6.29%
Tumor tissue	Tetraspanin GN=CD9 PE=1 SV=1	CD9_HUMAN	25,408.70	100.00%	1	2	4	0.00306%	6.29%
Tumor tissue	Tetratricopeptide repeat protein 1 (Fragment) GN=TTC1 PE=1 SV=1	TTC1_HUMAN	18,902.60	100.00%	3	3	3	0.00230%	27.00%

Biological sample	Protein name	Protein accession numbers	Protein molecular weight (Da)	Protein identification probability	Exclusive unique peptide count	Exclusive unique spectrum count	Total spectrum count	Percentage of total spectra	Percentage sequence coverage
Tumor tissue	Tetratricopeptide repeat protein 37 GN=TTC37 PE=1 SV=1	TTC37_HUMAN	175,492.10	100.00%	7	7	8	0.00612%	5.95%
Swab	Tetratricopeptide repeat protein 38 GN=TTC38 PE=1 SV=1	TTC38_HUMAN	52,788.40	99.30%	1	1	1	0.00071%	2.35%
Tumor tissue	Tetratricopeptide repeat protein 38 GN=TTC38 PE=1 SV=1	TTC38_HUMAN	52,788.40	100.00%	2	2	2	0.00153%	4.26%
Tumor tissue	Thimet oligopeptidase GN=THOP1 PE=1 SV=1	K7EP46_HUMAN	65,120.70	100.00%	3	3	3	0.00230%	7.57%
Tumor tissue	Thimet oligopeptidase (Fragment) GN=THOP1 PE=1 SV=8	K7EKB6_HUMAN	20,589.60	100.00%	2	2	2	0.00153%	13.60%
Tumor tissue	Thiopurine S-methyltransferase GN=TPMT PE=1 SV=1	TPMT_HUMAN	28,181.10	100.00%	2	2	2	0.00153%	11.40%
Pap test	Thioredoxin GN=TXN PE=1 SV=3	sp|P10599|THIO_HUMAN	11,737.30	100.00%	6	7	17	0.02650%	51.40%
Swab	Thioredoxin GN=TXN PE=1 SV=3	sp|P10599|THIO_HUMAN	11,737.30	100.00%	5	8	16	0.01130%	65.70%
Tumor tissue	Thioredoxin GN=TXN PE=1 SV=3	sp|P10599|THIO_HUMAN	11,737.30	100.00%	4	6	17	0.01300%	42.90%
Swab	Thioredoxin domain-containing protein 12 GN=TXNDC12 PE=1 SV=1	TXD12_HUMAN	19,206.40	99.40%	1	1	3	0.00213%	5.23%
Tumor tissue	Thioredoxin domain-containing protein 12 GN=TXNDC12 PE=1 SV=1	TXD12_HUMAN	19,206.40	100.00%	4	5	8	0.00612%	30.80%
Pap test	Thioredoxin domain-containing protein 17 GN=TXNDC17 PE=1 SV=1	TXD17_HUMAN	13,940.40	99.90%	1	1	1	0.00156%	11.40%
Swab	Thioredoxin domain-containing protein 17 GN=TXNDC17 PE=1 SV=1	TXD17_HUMAN	13,940.40	100.00%	2	3	12	0.00850%	22.80%
Tumor tissue	Thioredoxin domain-containing protein 17 GN=TXNDC17 PE=1 SV=1	TXD17_HUMAN	13,940.40	100.00%	2	2	4	0.00306%	19.50%
Pap test	Thioredoxin domain-containing protein 5 GN=TXNDC5 PE=1 SV=2	sp|Q8NBS9|TXND5_HUMAN	47,629.10	100.00%	2	2	2	0.00312%	6.94%
Swab	Thioredoxin domain-containing protein 5 GN=TXNDC5 PE=1 SV=2	sp|Q8NBS9|TXND5_HUMAN	47,629.10	100.00%	1	1	2	0.00142%	4.63%
Tumor tissue	Thioredoxin domain-containing protein 5 GN=TXNDC5 PE=1 SV=2	sp|Q8NBS9|TXND5_HUMAN	47,629.10	100.00%	11	15	35	0.02680%	31.20%
Tumor tissue	Thioredoxin reductase 2, mitochondrial GN=TXNRD2 PE=1 SV=3	D3YTF8_HUMAN	54,240.40	100.00%	2	3	5	0.00383%	14.40%

Biological sample	Protein name	Protein accession numbers	Protein molecular weight (Da)	Protein identification probability	Exclusive unique peptide count	Exclusive unique spectrum count	Total spectrum count	Percentage of total spectra	Percentage sequence coverage
Pap test	Thioredoxin-like protein 1 (Fragment) GN=TXNL1 PE=1 SV=1	TXNL1_HUMAN	31,466.60	100.00%	2	2	2	0.00312%	9.25%
Swab	Thioredoxin-like protein 1 (Fragment) GN=TXNL1 PE=1 SV=1	TXNL1_HUMAN	31,466.60	100.00%	3	4	8	0.00567%	20.30%
Tumor tissue	Thioredoxin-like protein 1 (Fragment) GN=TXNL1 PE=1 SV=1	TXNL1_HUMAN	31,466.60	100.00%	5	6	8	0.00612%	24.20%
Tumor tissue	Thioredoxin-related transmembrane protein 1 GN=TMX1 PE=1 SV=1	TMX1_HUMAN	31,791.90	100.00%	6	7	7	0.00536%	19.30%
Tumor tissue	Thioredoxin-related transmembrane protein 4 GN=TMX4 PE=1 SV=1	TMX4_HUMAN	38,951.90	100.00%	2	2	2	0.00153%	7.16%
Pap test	Thiosulfate sulfurtransferase GN=TST PE=1 SV=4	THTR_HUMAN	33,428.80	100.00%	2	2	2	0.00312%	10.40%
Swab	Thiosulfate sulfurtransferase GN=TST PE=1 SV=4	THTR_HUMAN	33,428.80	100.00%	2	2	3	0.00213%	8.42%
Tumor tissue	Thiosulfate sulfurtransferase GN=TST PE=1 SV=4	THTR_HUMAN	33,428.80	100.00%	5	6	8	0.00612%	22.90%
Tumor tissue	THO complex subunit 2 GN=THOC2 PE=1 SV=2	sp|Q8NI27|THOC2_HUMAN	182,780.40	100.00%	3	3	3	0.00230%	2.01%
Pap test	THO complex subunit 4 GN=ALYREF PE=1 SV=1	E9PB61_HUMAN	27,558.50	99.20%	1	1	1	0.00156%	4.17%
Swab	THO complex subunit 4 GN=ALYREF PE=1 SV=1	E9PB61_HUMAN	27,558.50	100.00%	1	1	1	0.00071%	4.17%
Tumor tissue	THO complex subunit 4 GN=ALYREF PE=1 SV=1	E9PB61_HUMAN	27,558.50	100.00%	3	6	7	0.00536%	20.50%
Tumor tissue	Threonine synthase-like 1 GN=THNSL1 PE=1 SV=2	THNS1_HUMAN	83,072.00	100.00%	3	3	3	0.00230%	5.25%
Tumor tissue	Thrombospondin-1 GN=THBS1 PE=1 SV=2	sp|P07996|TSP1_HUMAN	129,381.70	100.00%	19	28	37	0.02830%	22.60%
Tumor tissue	Thrombospondin-2 GN=THBS2 PE=1 SV=2	TSP2_HUMAN	129,988.90	100.00%	12	17	33	0.02530%	16.70%
Tumor tissue	Thromboxane-A synthase GN=TBXAS1 PE=1 SV=1	A0A0C4DH47_HUMAN	65,765.40	100.00%	3	3	4	0.00306%	7.76%
Tumor tissue	THUMP domain-containing protein 1 GN=THUMPD1 PE=1 SV=2	THUM1_HUMAN	39,315.60	100.00%	3	3	3	0.00230%	18.10%

Biological sample	Protein name	Protein accession numbers	Protein molecular weight (Da)	Protein identification probability	Exclusive unique peptide count	Exclusive unique spectrum count	Total spectrum count	Percentage of total spectra	Percentage sequence coverage
Tumor tissue	Thy-1 membrane glycoprotein (Fragment) GN=THY1 PE=1 SV=1	E9PNQ8_HUMAN	18,164.30	100.00%	3	5	14	0.01070%	15.80%
Pap test	Thymidine phosphorylase GN=TYMP PE=1 SV=2	sp|P19971|TYPH_HUMAN	49,955.80	100.00%	3	3	3	0.00468%	9.34%
Swab	Thymidine phosphorylase GN=TYMP PE=1 SV=2	sp|P19971|TYPH_HUMAN	49,955.80	100.00%	11	18	30	0.02130%	30.50%
Tumor tissue	Thymidine phosphorylase GN=TYMP PE=1 SV=2	sp|P19971|TYPH_HUMAN	49,955.80	100.00%	13	22	52	0.03980%	33.20%
Swab	Thymosin beta-4 GN=TMSB4X PE=1 SV=2	TYB4_HUMAN	5,052.90	100.00%	2	3	4	0.00283%	43.20%
Tumor tissue	Thymosin beta-4 GN=TMSB4X PE=1 SV=2	TYB4_HUMAN	5,052.90	100.00%	2	3	6	0.00459%	43.20%
Tumor tissue	Thyroid hormone receptor-associated protein 3 GN=THRAP3 PE=1 SV=2	TR150_HUMAN	108,668.90	100.00%	11	17	19	0.01450%	14.70%
Tumor tissue	Thyroid receptor-interacting protein 11 GN=TRIP11 PE=1 SV=3	TRIPB_HUMAN	227,590.50	100.00%	8	8	8	0.00612%	6.01%
Tumor tissue	Thyroid receptor-interacting protein 6 GN=TRIP6 PE=1 SV=3	sp|Q15654|TRIP6_HUMAN	50,286.70	100.00%	1	1	6	0.00459%	12.40%
Pap test	Thyroxine-binding globulin GN=SERPINA7 PE=1 SV=2	THBG_HUMAN	46,325.60	100.00%	5	7	10	0.01560%	13.00%
Swab	Thyroxine-binding globulin GN=SERPINA7 PE=1 SV=2	THBG_HUMAN	46,325.60	100.00%	6	8	13	0.00921%	22.40%
Tumor tissue	Tight junction protein 1 (Zona occludens 1), isoform CRA_a GN=TJP1 PE=1 SV=1	sp|Q07157|ZO1_HUMAN	197,460.60	100.00%	1	2	33	0.02530%	23.50%
Tumor tissue	Tight junction protein ZO-2 GN=TJP2 PE=1 SV=1	sp|Q9UDY2|ZO2_HUMAN	140,729.90	100.00%	2	2	2	0.00153%	2.32%
Pap test	Tissue alpha-L-fucosidase GN=FUCA1 PE=1 SV=4	FUCO_HUMAN	53,690.00	100.00%	2	2	2	0.00312%	6.01%
Swab	Tissue alpha-L-fucosidase GN=FUCA1 PE=1 SV=4	FUCO_HUMAN	53,690.00	100.00%	2	2	3	0.00213%	4.08%
Tumor tissue	Tissue alpha-L-fucosidase GN=FUCA1 PE=1 SV=4	FUCO_HUMAN	53,690.00	100.00%	2	2	2	0.00153%	4.72%
Pap test	Tissue-type plasminogen activator GN=PLAT PE=1 SV=1	B4DN26_HUMAN	52,991.70	100.00%	2	3	5	0.00780%	13.70%

Biological sample	Protein name	Protein accession numbers	Protein molecular weight (Da)	Protein identification probability	Exclusive unique peptide count	Exclusive unique spectrum count	Total spectrum count	Percentage of total spectra	Percentage sequence coverage
Swab	Tissue-type plasminogen activator GN=PLAT PE=1 SV=1	B4DN26_HUMAN	52,991.70	99.90%	1	1	2	0.00142%	5.29%
Tumor tissue	TLD domain-containing protein 1 GN=TLDC1 PE=1 SV=2	TLDC1_HUMAN	50,993.20	100.00%	4	5	5	0.00383%	15.10%
Tumor tissue	TNF receptor-associated factor 6 GN=TRAF6 PE=1 SV=1	TRAF6_HUMAN	59,573.10	100.00%	2	2	2	0.00153%	8.05%
Tumor tissue	Torsin-1A GN=TOR1A PE=1 SV=1	sp|O14656|TOR1A_HUMAN	37,810.60	100.00%	3	3	3	0.00230%	13.00%
Tumor tissue	Torsin-1A-interacting protein 1 GN=TOR1AIP1 PE=1 SV=2	sp|Q5JTV8|TOIP1_HUMAN	66,248.50	100.00%	7	9	10	0.00766%	19.60%
Tumor tissue	Torsin-1A-interacting protein 2 GN=TOR1AIP2 PE=1 SV=1	TOIP2_HUMAN	51,263.10	100.00%	2	3	3	0.00230%	7.45%
Tumor tissue	Trafficking protein particle complex subunit 3 GN=TRAPPC3 PE=1 SV=1	sp|O43617|TPPC3_HUMAN	21,232.00	100.00%	3	3	3	0.00230%	20.70%
Pap test	Transaldolase GN=TALDO1 PE=1 SV=2	TALDO_HUMAN	37,541.70	100.00%	1	1	15	0.02340%	23.40%
Swab	Transaldolase GN=TALDO1 PE=1 SV=2	TALDO_HUMAN	37,541.70	100.00%	1	2	28	0.01980%	27.60%
Tumor tissue	Transaldolase GN=TALDO1 PE=1 SV=2	TALDO_HUMAN	37,541.70	100.00%	2	2	21	0.01610%	31.50%
Pap test	Transcobalamin-1 GN=TCN1 PE=1 SV=2	TCO1_HUMAN	48,207.70	100.00%	3	3	4	0.00624%	11.30%
Swab	Transcobalamin-1 GN=TCN1 PE=1 SV=2	TCO1_HUMAN	48,207.70	100.00%	3	4	7	0.00496%	7.16%
Tumor tissue	Transcription elongation factor A protein 1 GN=TCEA1 PE=1 SV=1	E5RIS7_HUMAN	12,612.20	100.00%	1	1	4	0.00306%	23.40%
Swab	Transcription elongation factor A protein-like 3 GN=TCEAL3 PE=1 SV=1	TCAL3_HUMAN	22,502.00	99.80%	1	1	1	0.00071%	12.00%
Tumor tissue	Transcription elongation factor A protein-like 3 GN=TCEAL3 PE=1 SV=1	TCAL3_HUMAN	22,502.00	100.00%	1	2	5	0.00383%	21.00%
Tumor tissue	Transcription elongation factor SPT6 GN=SUPT6H PE=1 SV=2	sp|Q7KZ85|SPT6H_HUMAN	199,076.50	100.00%	2	2	4	0.00306%	2.95%

Biological sample	Protein name	Protein accession numbers	Protein molecular weight (Da)	Protein identification probability	Exclusive unique peptide count	Exclusive unique spectrum count	Total spectrum count	Percentage of total spectra	Percentage sequence coverage
Tumor tissue	Transcription factor A, mitochondrial GN=TFAM PE=1 SV=1	sp|Q00059|TFAM_HUMAN	29,097.80	100.00%	3	3	3	0.00230%	15.40%
Tumor tissue	Transcription factor BTF3 homolog 4 GN=BTF3L4 PE=1 SV=1	sp|Q96K17|BT3L4_HUMAN	17,271.00	100.00%	2	2	2	0.00153%	17.70%
Tumor tissue	Transcription factor ETV6 GN=ETV6 PE=1 SV=1	ETV6_HUMAN	53,001.10	100.00%	2	3	3	0.00230%	7.74%
Swab	Transcription initiation factor TFIID subunit 4 GN=TAF4 PE=1 SV=2	TAF4_HUMAN	110,114.00	100.00%	2	2	2	0.00142%	3.41%
Tumor tissue	Transcription intermediary factor 1-beta GN=TRIM28 PE=1 SV=5	sp|Q13263|TIF1B_HUMAN	88,549.00	100.00%	14	17	20	0.01530%	25.00%
Pap test	Transcriptional activator protein Pur-alpha GN=PURA PE=1 SV=2	PURA_HUMAN	34,911.10	100.00%	3	3	4	0.00624%	17.70%
Swab	Transcriptional activator protein Pur-alpha GN=PURA PE=1 SV=2	PURA_HUMAN	34,911.10	100.00%	4	4	5	0.00354%	20.80%
Tumor tissue	Transcriptional activator protein Pur-alpha GN=PURA PE=1 SV=2	PURA_HUMAN	34,911.10	100.00%	6	7	9	0.00689%	42.50%
Tumor tissue	Transcriptional repressor p66-beta GN=GATAD2B PE=1 SV=1	P66B_HUMAN	63,462.70	100.00%	3	3	4	0.00306%	10.70%
Tumor tissue	Transducin beta-like protein 3 GN=TBL3 PE=1 SV=2	TBL3_HUMAN	89,034.00	100.00%	2	2	3	0.00230%	5.32%
Swab	Transferrin receptor protein 1 GN=TFRC PE=1 SV=2	TFR1_HUMAN	84,873.60	100.00%	1	1	1	0.00071%	2.50%
Tumor tissue	Transferrin receptor protein 1 GN=TFRC PE=1 SV=2	TFR1_HUMAN	84,873.60	100.00%	12	14	14	0.01070%	21.20%
Tumor tissue	Transforming acidic coiled-coil-containing protein 1 GN=TACC1 PE=1 SV=1	sp|O75410|TACC1_HUMAN	66,987.40	100.00%	1	1	2	0.00153%	4.11%
Pap test	Transforming growth factor-beta-induced protein ig-h3 GN=TGFBI PE=1 SV=1	BGH3_HUMAN	74,682.90	100.00%	1	1	3	0.00468%	5.27%
Swab	Transforming growth factor-beta-induced protein ig-h3 GN=TGFBI PE=1 SV=1	BGH3_HUMAN	74,682.90	100.00%	1	1	3	0.00213%	4.10%
Tumor tissue	Transforming growth factor-beta-induced protein ig-h3 GN=TGFBI PE=1 SV=1	BGH3_HUMAN	74,682.90	100.00%	14	35	103	0.07880%	35.60%

Biological sample	Protein name	Protein accession numbers	Protein molecular weight (Da)	Protein identification probability	Exclusive unique peptide count	Exclusive unique spectrum count	Total spectrum count	Percentage of total spectra	Percentage sequence coverage
Pap test	Transforming protein RhoA GN=RHOA PE=1 SV=1	RHOA_HUMAN	21,768.40	100.00%	1	2	7	0.01090%	24.90%
Swab	Transforming protein RhoA GN=RHOA PE=1 SV=1	RHOA_HUMAN	21,768.40	100.00%	2	6	17	0.01200%	31.60%
Tumor tissue	Transforming protein RhoA GN=RHOA PE=1 SV=1	RHOA_HUMAN	21,768.40	100.00%	1	2	7	0.00536%	15.00%
Tumor tissue	Transgelin GN=TAGLN PE=1 SV=4	TAGL_HUMAN	22,611.00	100.00%	14	36	138	0.10600%	67.20%
Pap test	Transitional endoplasmic reticulum ATPase GN=VCP PE=1 SV=4	TERA_HUMAN	89,324.80	100.00%	23	37	71	0.11100%	42.30%
Swab	Transitional endoplasmic reticulum ATPase GN=VCP PE=1 SV=4	TERA_HUMAN	89,324.80	100.00%	22	26	46	0.03260%	38.50%
Tumor tissue	Transitional endoplasmic reticulum ATPase GN=VCP PE=1 SV=4	TERA_HUMAN	89,324.80	100.00%	23	38	69	0.05280%	34.40%
Tumor tissue	Translation initiation factor eIF-2B subunit alpha GN=EIF2B1 PE=1 SV=1	sp|Q14232|EI2BA_HUMAN	33,713.20	100.00%	1	1	2	0.00153%	8.20%
Tumor tissue	Translation initiation factor eIF-2B subunit beta GN=EIF2B2 PE=1 SV=3	EI2BB_HUMAN	38,990.10	100.00%	2	3	3	0.00230%	8.55%
Tumor tissue	Translation initiation factor eIF-2B subunit delta GN=EIF2B4 PE=1 SV=1	sp|Q9UI10|EI2BD_HUMAN	59,714.60	100.00%	4	4	4	0.00306%	10.20%
Pap test	Translationally-controlled tumor protein GN=TPT1 PE=1 SV=1	sp|P13693|TCTP_HUMAN	22,574.80	99.00%	1	1	1	0.00156%	6.60%
Swab	Translationally-controlled tumor protein GN=TPT1 PE=1 SV=1	sp|P13693|TCTP_HUMAN	22,574.80	100.00%	1	1	2	0.00142%	13.20%
Tumor tissue	Translationally-controlled tumor protein GN=TPT1 PE=1 SV=1	sp|P13693|TCTP_HUMAN	22,574.80	100.00%	1	3	10	0.00766%	13.70%
Pap test	Translin GN=TSN PE=1 SV=1	E9PGT1_HUMAN	25,572.70	100.00%	4	4	6	0.00936%	20.20%
Swab	Translin GN=TSN PE=1 SV=1	E9PGT1_HUMAN	25,572.70	100.00%	5	7	9	0.00638%	20.20%
Tumor tissue	Translin GN=TSN PE=1 SV=1	E9PGT1_HUMAN	25,572.70	100.00%	5	8	8	0.00612%	26.50%
Pap test	Translin-associated protein X GN=TSNAX PE=1 SV=1	TSNAX_HUMAN	33,113.10	99.90%	1	1	1	0.00156%	6.90%

Biological sample	Protein name	Protein accession numbers	Protein molecular weight (Da)	Protein identification probability	Exclusive unique peptide count	Exclusive unique spectrum count	Total spectrum count	Percentage of total spectra	Percentage sequence coverage
Swab	Translin-associated protein X GN=TSNAX PE=1 SV=1	TSNAX_HUMAN	33,113.10	99.90%	1	1	1	0.00071%	7.59%
Tumor tissue	Translin-associated protein X GN=TSNAX PE=1 SV=1	TSNAX_HUMAN	33,113.10	100.00%	4	4	4	0.00306%	22.40%
Tumor tissue	Translocating chain-associated membrane protein 1 GN=TRAM1 PE=1 SV=1	sp|Q15629|TRAM1_HUMAN	33,436.80	100.00%	5	7	15	0.01150%	15.60%
Tumor tissue	Translocation protein SEC63 homolog GN=SEC63 PE=1 SV=2	SEC63_HUMAN	87,999.40	100.00%	7	7	8	0.00612%	13.20%
Tumor tissue	Translocon-associated protein subunit delta GN=SSR4 PE=1 SV=1	SSRD_HUMAN	18,998.80	100.00%	4	8	21	0.01610%	30.60%
Tumor tissue	Transmembrane 9 superfamily member GN=TM9SF4 PE=1 SV=1	TM9S4_HUMAN	74,521.30	100.00%	3	3	4	0.00306%	6.72%
Tumor tissue	Transmembrane 9 superfamily member PE=3 SV=1	E9PSI1_HUMAN	92,389.90	100.00%	1	1	3	0.00230%	4.42%
Tumor tissue	Transmembrane 9 superfamily member 2 GN=TM9SF2 PE=1 SV=1	TM9S2_HUMAN	75,778.90	100.00%	3	4	4	0.00306%	6.79%
Tumor tissue	Transmembrane 9 superfamily member 3 GN=TM9SF3 PE=1 SV=2	TM9S3_HUMAN	67,890.80	100.00%	4	4	4	0.00306%	6.79%
Tumor tissue	Transmembrane emp24 domain-containing protein 1 GN=TMED1 PE=1 SV=1	TMED1_HUMAN	25,206.00	100.00%	2	2	2	0.00153%	11.50%
Pap test	Transmembrane emp24 domain-containing protein 10 GN=TMED10 PE=1 SV=2	TMEDA_HUMAN	24,977.50	99.80%	1	1	2	0.00312%	5.02%
Tumor tissue	Transmembrane emp24 domain-containing protein 10 GN=TMED10 PE=1 SV=2	TMEDA_HUMAN	24,977.50	100.00%	9	17	25	0.01910%	41.10%
Tumor tissue	Transmembrane emp24 domain-containing protein 2 GN=TMED2 PE=1 SV=1	TMED2_HUMAN	22,761.40	100.00%	1	1	7	0.00536%	21.40%
Tumor tissue	Transmembrane emp24 domain-containing protein 5 GN=TMED5 PE=1 SV=1	sp|Q9Y3A6|TMED5_HUMAN	26,005.80	100.00%	2	2	2	0.00153%	9.17%

Biological sample	Protein name	Protein accession numbers	Protein molecular weight (Da)	Protein identification probability	Exclusive unique peptide count	Exclusive unique spectrum count	Total spectrum count	Percentage of total spectra	Percentage sequence coverage
Tumor tissue	Transmembrane emp24 domain-containing protein 9 GN=TMED9 PE=1 SV=2	TMED9_HUMAN	27,277.50	100.00%	5	6	6	0.00459%	28.10%
Pap test	Transmembrane protease serine 11D GN=TMPRSS11D PE=1 SV=1	TM11D_HUMAN	46,263.70	100.00%	6	7	9	0.01400%	17.20%
Pap test	Transmembrane protein 109 GN=TMEM109 PE=1 SV=1	TM109_HUMAN	26,211.20	99.20%	1	1	1	0.00156%	4.94%
Tumor tissue	Transmembrane protein 109 GN=TMEM109 PE=1 SV=1	TM109_HUMAN	26,211.20	100.00%	2	3	4	0.00306%	9.05%
Tumor tissue	Transmembrane protein 119 GN=TMEM119 PE=1 SV=1	TM119_HUMAN	29,202.70	100.00%	2	2	2	0.00153%	12.70%
Tumor tissue	Transmembrane protein 165 GN=TMEM165 PE=1 SV=1	sp|Q9HC07|TM165_HUMAN	34,906.40	100.00%	2	2	4	0.00306%	13.90%
Tumor tissue	Transmembrane protein 201 GN=TMEM201 PE=1 SV=1	sp|Q5SNT2|TM201_HUMAN	72,236.80	100.00%	2	2	2	0.00153%	3.45%
Tumor tissue	Transmembrane protein 205 (Fragment) GN=TMEM205 PE=1 SV=1	TM205_HUMAN	19,271.00	100.00%	3	4	5	0.00383%	22.90%
Tumor tissue	Transmembrane protein 214 GN=TMEM214 PE=1 SV=2	sp|Q6NUQ4|TM214_HUMAN	77,153.50	100.00%	5	6	6	0.00459%	12.60%
Tumor tissue	Transmembrane protein 263 GN=TMEM263 PE=1 SV=1	TM263_HUMAN	11,748.00	100.00%	6	7	9	0.00689%	57.80%
Pap test	Transmembrane protein 43 GN=TMEM43 PE=1 SV=1	TMM43_HUMAN	44,876.80	100.00%	2	2	2	0.00312%	5.00%
Tumor tissue	Transmembrane protein 43 GN=TMEM43 PE=1 SV=1	TMM43_HUMAN	44,876.80	100.00%	9	11	21	0.01610%	35.70%
Pap test	Transthyretin GN=TTR PE=1 SV=1	TTHY_HUMAN	15,886.90	100.00%	6	11	33	0.05150%	63.90%
Swab	Transthyretin GN=TTR PE=1 SV=1	TTHY_HUMAN	15,886.90	100.00%	5	9	52	0.03690%	44.20%
Tumor tissue	Transthyretin GN=TTR PE=1 SV=1	TTHY_HUMAN	15,886.90	100.00%	7	11	16	0.01220%	64.60%
Tumor tissue	Tricarboxylate transport protein, mitochondrial GN=SLC25A1 PE=1 SV=2	TXTP_HUMAN	34,013.20	100.00%	4	6	7	0.00536%	13.80%
Pap test	Trifunctional enzyme subunit alpha, mitochondrial GN=HADHA PE=1 SV=2	sp|P40939|ECHA_HUMAN	83,001.90	100.00%	4	5	6	0.00936%	9.96%

Biological sample	Protein name	Protein accession numbers	Protein molecular weight (Da)	Protein identification probability	Exclusive unique peptide count	Exclusive unique spectrum count	Total spectrum count	Percentage of total spectra	Percentage sequence coverage
Swab	Trifunctional enzyme subunit alpha, mitochondrial GN=HADHA PE=1 SV=2	sp|P40939|ECHA_HUMAN	83,001.90	100.00%	1	1	1	0.00071%	1.57%
Tumor tissue	Trifunctional enzyme subunit alpha, mitochondrial GN=HADHA PE=1 SV=2	sp|P40939|ECHA_HUMAN	83,001.90	100.00%	20	32	43	0.03290%	32.40%
Pap test	Trifunctional enzyme subunit beta, mitochondrial GN=HADHB PE=1 SV=3	sp|P55084|ECHB_HUMAN	51,296.00	100.00%	1	1	4	0.00624%	8.23%
Tumor tissue	Trifunctional enzyme subunit beta, mitochondrial GN=HADHB PE=1 SV=3	sp|P55084|ECHB_HUMAN	51,296.00	100.00%	6	7	20	0.01530%	31.20%
Pap test	Trifunctional purine biosynthetic protein adenosine-3 GN=GART PE=1 SV=1	sp|P22102|PUR2_HUMAN	107,768.40	100.00%	2	2	2	0.00312%	3.86%
Swab	Trifunctional purine biosynthetic protein adenosine-3 GN=GART PE=1 SV=1	sp|P22102|PUR2_HUMAN	107,768.40	100.00%	2	2	3	0.00213%	1.78%
Tumor tissue	Trifunctional purine biosynthetic protein adenosine-3 GN=GART PE=1 SV=1	sp|P22102|PUR2_HUMAN	107,768.40	100.00%	13	13	16	0.01220%	18.80%
Pap test	Triokinase/FMN cyclase GN=TKFC PE=1 SV=2	sp|Q3LXA3|TKFC_HUMAN	58,948.10	99.30%	1	1	1	0.00156%	2.09%
Swab	Triokinase/FMN cyclase GN=TKFC PE=1 SV=2	sp|Q3LXA3|TKFC_HUMAN	58,948.10	100.00%	4	5	6	0.00425%	11.50%
Tumor tissue	Triokinase/FMN cyclase GN=TKFC PE=1 SV=2	sp|Q3LXA3|TKFC_HUMAN	58,948.10	100.00%	6	9	10	0.00766%	18.40%
Tumor tissue	Tripartite motif-containing protein 16 GN=TRIM16 PE=1 SV=3	sp|O95361|TRI16_HUMAN	63,955.30	99.90%	1	1	2	0.00153%	5.50%
Tumor tissue	Tripartite motif-containing protein 47 GN=TRIM47 PE=1 SV=2	sp|Q96LD4|TRI47_HUMAN	69,532.80	100.00%	3	3	3	0.00230%	6.90%
Tumor tissue	Tripartite motif-containing protein 65 GN=TRIM65 PE=1 SV=3	TRI65_HUMAN	57,352.70	100.00%	2	2	2	0.00153%	6.19%
Pap test	Tripeptidyl-peptidase 1 GN=TPP1 PE=1 SV=2	sp|O14773|TPP1_HUMAN	61,247.60	99.80%	1	1	1	0.00156%	2.49%

Biological sample	Protein name	Protein accession numbers	Protein molecular weight (Da)	Protein identification probability	Exclusive unique peptide count	Exclusive unique spectrum count	Total spectrum count	Percentage of total spectra	Percentage sequence coverage
Swab	Tripeptidyl-peptidase 1 GN=TPP1 PE=1 SV=2	sp|O14773|TPP1_HUMAN	61,247.60	99.80%	1	1	2	0.00142%	2.49%
Tumor tissue	Tripeptidyl-peptidase 1 GN=TPP1 PE=1 SV=2	sp|O14773|TPP1_HUMAN	61,247.60	100.00%	3	5	10	0.00766%	8.17%
Tumor tissue	Tripeptidyl-peptidase 2 GN=TPP2 PE=1 SV=4	TPP2_HUMAN	138,352.90	100.00%	13	14	15	0.01150%	15.10%
Tumor tissue	Triple functional domain protein GN=TRIO PE=1 SV=2	sp|O75962|TRIO_HUMAN	346,902.60	100.00%	5	5	5	0.00383%	1.78%
Tumor tissue	tRNA pseudouridine synthase (Fragment) GN=PUS1 PE=1 SV=1	sp|Q9Y606-2|TRUA_HUMAN	42,921.10	100.00%	4	4	4	0.00306%	13.00%
Tumor tissue	tRNA-splicing ligase RtcB homolog GN=RTCB PE=1 SV=1	RTCB_HUMAN	55,210.20	100.00%	11	12	15	0.01150%	29.30%
Pap test	Tropomodulin-3 GN=TMOD3 PE=1 SV=1	TMOD3_HUMAN	39,596.10	100.00%	2	2	4	0.00624%	11.60%
Swab	Tropomodulin-3 GN=TMOD3 PE=1 SV=1	TMOD3_HUMAN	39,596.10	100.00%	3	3	5	0.00354%	15.10%
Tumor tissue	Tropomodulin-3 GN=TMOD3 PE=1 SV=1	TMOD3_HUMAN	39,596.10	100.00%	10	13	20	0.01530%	38.10%
Pap test	Tropomyosin alpha-1 chain GN=TPM1 PE=1 SV=1	H7BYY1_HUMAN	28,747.80	99.80%	1	1	8	0.01250%	11.60%
Swab	Tropomyosin alpha-1 chain GN=TPM1 PE=1 SV=1	H7BYY1_HUMAN	28,747.80	99.90%	1	1	15	0.01060%	9.82%
Tumor tissue	Tropomyosin alpha-1 chain GN=TPM1 PE=1 SV=1	H7BYY1_HUMAN	28,747.80	100.00%	1	1	28	0.02140%	27.60%
Pap test	Tropomyosin alpha-3 chain GN=TPM3 PE=1 SV=1	J3KN67_HUMAN	33,223.90	99.90%	1	2	14	0.02180%	14.40%
Swab	Tropomyosin alpha-3 chain GN=TPM3 PE=1 SV=1	J3KN67_HUMAN	33,223.90	100.00%	1	1	24	0.01700%	15.80%
Tumor tissue	Tropomyosin alpha-3 chain GN=TPM3 PE=1 SV=1	J3KN67_HUMAN	33,223.90	100.00%	3	5	34	0.02600%	35.10%
Pap test	Tropomyosin alpha-4 chain GN=TPM4 PE=1 SV=3	sp|P67936|TPM4_HUMAN	28,522.40	100.00%	4	4	16	0.02500%	27.80%
Swab	Tropomyosin alpha-4 chain GN=TPM4 PE=1 SV=3	sp|P67936|TPM4_HUMAN	28,522.40	100.00%	4	6	32	0.02270%	35.90%
Tumor tissue	Tropomyosin alpha-4 chain GN=TPM4 PE=1 SV=3	sp|P67936|TPM4_HUMAN	28,522.40	100.00%	4	9	41	0.03140%	26.60%

Biological sample	Protein name	Protein accession numbers	Protein molecular weight (Da)	Protein identification probability	Exclusive unique peptide count	Exclusive unique spectrum count	Total spectrum count	Percentage of total spectra	Percentage sequence coverage
Tumor tissue	Tropomyosin beta chain GN=TPM2 PE=1 SV=1	sp|P07951|TPM2_HUMAN	32,851.70	99.80%	1	1	44	0.03370%	34.20%
Tumor tissue	Tryptase beta-2 GN=TPSB2 PE=1 SV=1	TRYB2_HUMAN	32,575.70	100.00%	2	3	4	0.00306%	12.00%
Pap test	Tryptophan--tRNA ligase, cytoplasmic GN=WARS PE=1 SV=2	sp|P23381|SYWC_HUMAN	53,167.60	100.00%	3	3	4	0.00624%	7.01%
Swab	Tryptophan--tRNA ligase, cytoplasmic GN=WARS PE=1 SV=2	sp|P23381|SYWC_HUMAN	53,167.60	100.00%	2	2	2	0.00142%	6.37%
Tumor tissue	Tryptophan--tRNA ligase, cytoplasmic GN=WARS PE=1 SV=2	sp|P23381|SYWC_HUMAN	53,167.60	100.00%	13	24	32	0.02450%	34.60%
Tumor tissue	Tubulin alpha chain (Fragment) GN=TUBA8 PE=1 SV=1	sp|Q9NY65|TBA8_HUMAN	51,985.20	99.80%	1	1	80	0.06120%	26.10%
Pap test	Tubulin alpha-4A chain GN=TUBA4A PE=1 SV=1	sp|P68366|TBA4A_HUMAN	49,924.60	100.00%	4	5	27	0.04210%	26.60%
Swab	Tubulin alpha-4A chain GN=TUBA4A PE=1 SV=1	sp|P68366|TBA4A_HUMAN	49,924.60	100.00%	4	4	37	0.02620%	39.10%
Tumor tissue	Tubulin alpha-4A chain GN=TUBA4A PE=1 SV=1	sp|P68366|TBA4A_HUMAN	49,924.60	100.00%	3	4	113	0.08650%	33.00%
Pap test	Tubulin beta chain GN=TUBB PE=1 SV=2	TBB5_HUMAN	49,670.60	100.00%	1	1	26	0.04060%	28.40%
Swab	Tubulin beta chain GN=TUBB PE=1 SV=2	TBB5_HUMAN	49,670.60	100.00%	3	4	43	0.03050%	42.60%
Tumor tissue	Tubulin beta chain GN=TUBB PE=1 SV=2	TBB5_HUMAN	49,670.60	100.00%	5	11	190	0.14500%	52.30%
Tumor tissue	Tubulin beta-2A chain GN=TUBB2A PE=1 SV=1	TBB2A_HUMAN	49,907.10	100.00%	1	1	136	0.10400%	45.80%
Tumor tissue	Tubulin beta-2B chain GN=TUBB2B PE=1 SV=1	TBB2B_HUMAN	49,953.10	100.00%	1	1	135	0.10300%	45.80%
Tumor tissue	Tubulin beta-3 chain GN=TUBB3 PE=1 SV=2	sp|Q13509|TBB3_HUMAN	50,432.70	100.00%	4	8	112	0.08570%	45.10%
Pap test	Tubulin beta-4A chain GN=TUBB4A PE=1 SV=2	TBB4A_HUMAN	49,585.50	99.50%	1	1	27	0.04210%	32.90%
Tumor tissue	Tubulin beta-4A chain GN=TUBB4A PE=1 SV=2	TBB4A_HUMAN	49,585.50	100.00%	1	3	135	0.10300%	48.20%
Pap test	Tubulin beta-4B chain GN=TUBB4B PE=1 SV=1	TBB4B_HUMAN	49,830.70	99.80%	1	1	28	0.04370%	35.50%

Biological sample	Protein name	Protein accession numbers	Protein molecular weight (Da)	Protein identification probability	Exclusive unique peptide count	Exclusive unique spectrum count	Total spectrum count	Percentage of total spectra	Percentage sequence coverage
Tumor tissue	Tubulin beta-4B chain GN=TUBB4B PE=1 SV=1	TBB4B_HUMAN	49,830.70	100.00%	1	2	161	0.12300%	51.90%
Tumor tissue	Tubulin beta-6 chain GN=TUBB6 PE=1 SV=1	TBB6_HUMAN	49,857.20	100.00%	6	9	115	0.08800%	43.50%
Tumor tissue	Tubulin gamma-2 chain GN=TUBG2 PE=2 SV=1	TBG2_HUMAN	51,092.90	100.00%	3	3	4	0.00306%	10.60%
Swab	Tubulin polymerization-promoting protein family member 3 GN=TPPP3 PE=1 SV=1	TPPP3_HUMAN	18,985.90	100.00%	2	2	2	0.00142%	16.50%
Tumor tissue	Tubulin polymerization-promoting protein family member 3 GN=TPPP3 PE=1 SV=1	TPPP3_HUMAN	18,985.90	100.00%	2	2	2	0.00153%	15.30%
Tumor tissue	Tubulin-folding cofactor B (Fragment) GN=TBCB PE=1 SV=8	sp|Q99426|TBCB_HUMAN	19,251.40	100.00%	3	3	4	0.00306%	24.30%
Swab	Tubulin-specific chaperone A GN=TBCA PE=1 SV=1	sp|O75347|TBCA_HUMAN	14,307.00	100.00%	3	4	6	0.00425%	23.80%
Tumor tissue	Tubulin-specific chaperone A GN=TBCA PE=1 SV=1	sp|O75347|TBCA_HUMAN	14,307.00	100.00%	3	5	5	0.00383%	41.70%
Tumor tissue	Tubulin-specific chaperone cofactor E-like protein GN=TBCEL PE=1 SV=2	TBCEL_HUMAN	48,195.90	100.00%	2	2	3	0.00230%	7.55%
Pap test	Tubulin--tyrosine ligase-like protein 12 GN=TTLL12 PE=1 SV=2	TTL12_HUMAN	74,403.70	100.00%	3	3	3	0.00468%	5.28%
Swab	Tubulin--tyrosine ligase-like protein 12 GN=TTLL12 PE=1 SV=2	TTL12_HUMAN	74,403.70	100.00%	3	3	5	0.00354%	7.45%
Tumor tissue	Tubulin--tyrosine ligase-like protein 12 GN=TTLL12 PE=1 SV=2	TTL12_HUMAN	74,403.70	100.00%	7	11	12	0.00919%	16.30%
Tumor tissue	Tubulointerstitial nephritis antigen-like GN=TINAGL1 PE=1 SV=1	sp|Q9GZM7|TINAL_HUMAN	52,387.00	100.00%	1	1	2	0.00153%	4.71%
Pap test	Tumor necrosis factor alpha-induced protein 2 GN=TNFAIP2 PE=2 SV=2	TNAP2_HUMAN	72,663.20	100.00%	3	3	3	0.00468%	4.28%
Swab	Tumor necrosis factor alpha-induced protein 2 GN=TNFAIP2 PE=2 SV=2	TNAP2_HUMAN	72,663.20	99.90%	1	1	1	0.00071%	2.75%

Biological sample	Protein name	Protein accession numbers	Protein molecular weight (Da)	Protein identification probability	Exclusive unique peptide count	Exclusive unique spectrum count	Total spectrum count	Percentage of total spectra	Percentage sequence coverage
Tumor tissue	Tumor necrosis factor alpha-induced protein 2 GN=TNFAIP2 PE=2 SV=2	TNAP2_HUMAN	72,663.20	100.00%	6	8	9	0.00689%	13.50%
Swab	Tumor necrosis factor receptor type 1-associated DEATH domain protein GN=TRADD PE=1 SV=2	sp|Q15628|TRADD_HUMAN	34,247.50	99.60%	1	2	2	0.00142%	4.49%
Tumor tissue	Tumor necrosis factor receptor type 1-associated DEATH domain protein GN=TRADD PE=1 SV=2	sp|Q15628|TRADD_HUMAN	34,247.50	100.00%	3	3	3	0.00230%	12.20%
Tumor tissue	Tumor susceptibility gene 101 protein GN=TSG101 PE=1 SV=2	sp|Q99816|TS101_HUMAN	43,946.00	100.00%	5	5	5	0.00383%	16.70%
Pap test	Tumor-associated calcium signal transducer 2 GN=TACSTD2 PE=1 SV=3	TACD2_HUMAN	35,709.80	100.00%	4	7	8	0.01250%	21.70%
Swab	Tumor-associated calcium signal transducer 2 GN=TACSTD2 PE=1 SV=3	TACD2_HUMAN	35,709.80	100.00%	3	4	6	0.00425%	15.80%
Tumor tissue	Tumor-associated calcium signal transducer 2 GN=TACSTD2 PE=1 SV=3	TACD2_HUMAN	35,709.80	99.80%	1	1	1	0.00077%	4.95%
Swab	Twinfilin-1 GN=TWF1 PE=1 SV=3	sp|Q12792|TWF1_HUMAN	40,284.20	100.00%	1	2	7	0.00496%	17.70%
Tumor tissue	Twinfilin-1 GN=TWF1 PE=1 SV=3	sp|Q12792|TWF1_HUMAN	40,284.20	100.00%	1	3	9	0.00689%	20.90%
Pap test	Twinfilin-2 GN=TWF2 PE=1 SV=2	TWF2_HUMAN	39,548.90	99.90%	1	2	2	0.00312%	4.87%
Swab	Twinfilin-2 GN=TWF2 PE=1 SV=2	TWF2_HUMAN	39,548.90	100.00%	2	2	6	0.00425%	17.20%
Tumor tissue	Twinfilin-2 GN=TWF2 PE=1 SV=2	TWF2_HUMAN	39,548.90	100.00%	3	6	17	0.01300%	33.00%
Tumor tissue	Type-1 angiotensin II receptor-associated protein GN=AGTRAP PE=1 SV=1	sp|Q6RW13|ATRAP_HUMAN	17,419.90	100.00%	2	3	3	0.00230%	24.50%
Tumor tissue	Tyrosine-protein kinase GN=HCK PE=1 SV=1	sp|P08631|HCK_HUMAN	57,242.80	100.00%	2	2	6	0.00459%	8.19%
Pap test	Tyrosine-protein kinase CSK GN=CSK PE=1 SV=1	CSK_HUMAN	50,705.00	99.70%	1	1	1	0.00156%	2.89%
Swab	Tyrosine-protein kinase CSK GN=CSK PE=1 SV=1	CSK_HUMAN	50,705.00	100.00%	1	1	1	0.00071%	2.00%

Biological sample	Protein name	Protein accession numbers	Protein molecular weight (Da)	Protein identification probability	Exclusive unique peptide count	Exclusive unique spectrum count	Total spectrum count	Percentage of total spectra	Percentage sequence coverage
Tumor tissue	Tyrosine-protein kinase CSK GN=CSK PE=1 SV=1	CSK_HUMAN	50,705.00	100.00%	4	6	10	0.00766%	14.20%
Tumor tissue	Tyrosine-protein kinase Lyn GN=LYN PE=1 SV=3	sp|P07948|LYN_HUMAN	58,576.30	100.00%	3	3	7	0.00536%	12.90%
Tumor tissue	Tyrosine-protein phosphatase non-receptor type 1 GN=PTPN1 PE=1 SV=1	PTN1_HUMAN	49,967.70	100.00%	5	6	6	0.00459%	15.60%
Tumor tissue	Tyrosine-protein phosphatase non-receptor type 11 GN=PTPN11 PE=1 SV=2	sp|Q06124|PTN11_HUMAN	68,436.20	100.00%	6	6	6	0.00459%	15.60%
Tumor tissue	Tyrosine-protein phosphatase non-receptor type 23 GN=PTPN23 PE=1 SV=1	PTN23_HUMAN	178,974.60	100.00%	9	9	10	0.00766%	7.82%
Tumor tissue	Tyrosine-protein phosphatase non-receptor type 9 GN=PTPN9 PE=1 SV=1	PTN9_HUMAN	66,954.60	100.00%	2	2	3	0.00230%	3.95%
Tumor tissue	Tyrosine--tRNA ligase (Fragment) GN=YARS2 PE=1 SV=1	SYYM_HUMAN	32,185.50	100.00%	3	3	3	0.00230%	21.00%
Tumor tissue	Tyrosine--tRNA ligase, cytoplasmic GN=YARS PE=1 SV=4	SYYC_HUMAN	59,145.10	100.00%	13	15	17	0.01300%	29.00%
Tumor tissue	U1 small nuclear ribonucleoprotein 70 kDa GN=SNRNP70 PE=1 SV=2	sp|P08621|RU17_HUMAN	51,558.40	100.00%	6	9	13	0.00995%	16.70%
Tumor tissue	U2 small nuclear ribonucleoprotein A' GN=SNRPA1 PE=1 SV=2	RU2A_HUMAN	28,417.10	100.00%	3	4	7	0.00536%	27.50%
Tumor tissue	U2 small nuclear ribonucleoprotein B'' GN=SNRPB2 PE=1 SV=1	RU2B_HUMAN	25,487.50	100.00%	3	3	5	0.00383%	23.60%
Tumor tissue	U3 small nucleolar ribonucleoprotein protein IMP3 GN=IMP3 PE=1 SV=1	IMP3_HUMAN	21,850.30	100.00%	3	4	4	0.00306%	23.40%
Tumor tissue	U3 small nucleolar ribonucleoprotein protein MPP10 GN=MPHOSPH10 PE=1 SV=2	MPP10_HUMAN	78,866.80	100.00%	2	2	2	0.00153%	5.29%

Biological sample	Protein name	Protein accession numbers	Protein molecular weight (Da)	Protein identification probability	Exclusive unique peptide count	Exclusive unique spectrum count	Total spectrum count	Percentage of total spectra	Percentage sequence coverage
Tumor tissue	U3 small nucleolar RNA-associated protein 4 homolog (Fragment) GN=UTP4 PE=1 SV=1	H3BSH7_HUMAN	77,944.40	100.00%	4	4	4	0.00306%	10.00%
Tumor tissue	U4/U6 small nuclear ribonucleoprotein Prp3 GN=PRPF3 PE=1 SV=2	sp|O43395|PRPF3_HUMAN	77,530.70	100.00%	3	3	3	0.00230%	6.73%
Tumor tissue	U4/U6 small nuclear ribonucleoprotein Prp31 GN=PRPF31 PE=1 SV=2	sp|Q8WWY3|PRP31_HUMAN	55,457.40	100.00%	6	8	8	0.00612%	18.40%
Tumor tissue	U4/U6.U5 tri-snRNP-associated protein 1 GN=SART1 PE=1 SV=1	SNUT1_HUMAN	90,257.10	100.00%	7	7	7	0.00536%	13.40%
Tumor tissue	U4/U6.U5 tri-snRNP-associated protein 2 GN=USP39 PE=1 SV=1	sp|Q53GS9|SNUT2_HUMAN	56,402.00	100.00%	4	5	7	0.00536%	18.30%
Tumor tissue	U5 small nuclear ribonucleoprotein 200 kDa helicase GN=SNRNP200 PE=1 SV=2	sp|O75643|U520_HUMAN	244,513.80	100.00%	36	40	40	0.03060%	22.90%
Tumor tissue	U5 small nuclear ribonucleoprotein 40 kDa protein GN=SNRNP40 PE=1 SV=1	sp|Q96DI7|SNR40_HUMAN	39,311.00	100.00%	3	4	4	0.00306%	14.00%
Pap test	U6 snRNA-associated Sm-like protein LSm2 GN=LSM2 PE=1 SV=1	LSM2_HUMAN	10,834.60	99.40%	1	1	1	0.00156%	20.00%
Swab	U6 snRNA-associated Sm-like protein LSm2 GN=LSM2 PE=1 SV=1	LSM2_HUMAN	10,834.60	99.60%	1	1	1	0.00071%	20.00%
Tumor tissue	U6 snRNA-associated Sm-like protein LSm2 GN=LSM2 PE=1 SV=1	LSM2_HUMAN	10,834.60	100.00%	2	3	4	0.00306%	40.00%
Pap test	U6 snRNA-associated Sm-like protein LSm8 GN=LSM8 PE=1 SV=3	LSM8_HUMAN	10,402.60	99.20%	1	1	1	0.00156%	10.40%
Swab	U6 snRNA-associated Sm-like protein LSm8 GN=LSM8 PE=1 SV=3	LSM8_HUMAN	10,402.60	99.00%	1	1	1	0.00071%	25.00%
Tumor tissue	U6 snRNA-associated Sm-like protein LSm8 GN=LSM8 PE=1 SV=3	LSM8_HUMAN	10,402.60	100.00%	2	3	4	0.00306%	27.10%
Tumor tissue	UAP56-interacting factor GN=FYTTD1 PE=1 SV=3	sp|Q96QD9|UIF_HUMAN	35,819.30	100.00%	2	2	3	0.00230%	12.90%
Tumor tissue	Ubiquilin-1 GN=UBQLN1 PE=1 SV=2	sp|Q9UMX0|UBQL1_HUMAN	62,519.50	100.00%	3	4	12	0.00919%	20.70%
Swab	Ubiquilin-2 GN=UBQLN2 PE=1 SV=2	UBQL2_HUMAN	65,697.10	100.00%	1	1	1	0.00071%	2.56%

Biological sample	Protein name	Protein accession numbers	Protein molecular weight (Da)	Protein identification probability	Exclusive unique peptide count	Exclusive unique spectrum count	Total spectrum count	Percentage of total spectra	Percentage sequence coverage
Tumor tissue	Ubiquilin-2 GN=UBQLN2 PE=1 SV=2	UBQL2_HUMAN	65,697.10	100.00%	3	3	9	0.00689%	18.10%
Tumor tissue	Ubiquilin-4 GN=UBQLN4 PE=1 SV=2	sp|Q9NRR5|UBQL4_HUMAN	63,854.00	100.00%	1	1	7	0.00536%	14.30%
Tumor tissue	Ubiquinone biosynthesis protein COQ9, mitochondrial GN=COQ9 PE=1 SV=1	COQ9_HUMAN	35,509.10	100.00%	1	1	2	0.00153%	8.49%
Tumor tissue	Ubiquitin carboxyl-terminal hydrolase GN=UCHL1 PE=1 SV=1	UCHL1_HUMAN	24,824.30	100.00%	3	6	6	0.00459%	19.30%
Tumor tissue	Ubiquitin carboxyl-terminal hydrolase 15 GN=USP15 PE=1 SV=3	sp|Q9Y4E8|UBP15_HUMAN	112,423.40	100.00%	3	3	5	0.00383%	5.71%
Tumor tissue	Ubiquitin carboxyl-terminal hydrolase 24 GN=USP24 PE=1 SV=3	UBP24_HUMAN	294,371.70	100.00%	4	4	5	0.00383%	2.18%
Tumor tissue	Ubiquitin carboxyl-terminal hydrolase 7 GN=USP7 PE=1 SV=2	sp|Q93009|UBP7_HUMAN	128,305.00	100.00%	17	18	23	0.01760%	24.60%
Pap test	Ubiquitin carboxyl-terminal hydrolase isozyme L3 GN=UCHL3 PE=1 SV=1	UCHL3_HUMAN	26,182.80	99.20%	1	1	1	0.00156%	6.96%
Swab	Ubiquitin carboxyl-terminal hydrolase isozyme L3 GN=UCHL3 PE=1 SV=1	UCHL3_HUMAN	26,182.80	100.00%	3	4	5	0.00354%	14.80%
Tumor tissue	Ubiquitin carboxyl-terminal hydrolase isozyme L3 GN=UCHL3 PE=1 SV=1	UCHL3_HUMAN	26,182.80	100.00%	3	3	3	0.00230%	16.50%
Tumor tissue	Ubiquitin domain-containing protein UBFD1 GN=UBFD1 PE=1 SV=1	UBFD1_HUMAN	32,640.00	100.00%	3	3	3	0.00230%	20.70%
Pap test	Ubiquitin thioesterase GN=OTUB1 PE=1 SV=2	sp|Q96FW1|OTUB1_HUMAN	31,440.80	100.00%	2	2	2	0.00312%	6.99%
Swab	Ubiquitin thioesterase GN=OTUB1 PE=1 SV=2	sp|Q96FW1|OTUB1_HUMAN	31,440.80	100.00%	8	9	11	0.00780%	42.30%
Tumor tissue	Ubiquitin thioesterase GN=OTUB1 PE=1 SV=2	sp|Q96FW1|OTUB1_HUMAN	31,440.80	100.00%	6	8	9	0.00689%	25.40%
Swab	Ubiquitin-40S ribosomal protein S27a GN=RPS27A PE=1 SV=2	RS27A_HUMAN	17,965.60	100.00%	1	1	8	0.00567%	36.50%

Biological sample	Protein name	Protein accession numbers	Protein molecular weight (Da)	Protein identification probability	Exclusive unique peptide count	Exclusive unique spectrum count	Total spectrum count	Percentage of total spectra	Percentage sequence coverage
Tumor tissue	Ubiquitin-associated domain-containing protein 1 GN=UBAC1 PE=1 SV=1	UBAC1_HUMAN	45,339.80	100.00%	2	2	2	0.00153%	5.93%
Tumor tissue	Ubiquitin-conjugating enzyme E2 A GN=UBE2A PE=1 SV=1	sp|P49459|UBE2A_HUMAN	13,635.30	100.00%	2	2	2	0.00153%	22.70%
Tumor tissue	Ubiquitin-conjugating enzyme E2 E1 (Fragment) GN=UBE2E1 PE=1 SV=1	sp|P51965|UB2E1_HUMAN	16,211.00	99.80%	1	1	2	0.00153%	21.10%
Tumor tissue	Ubiquitin-conjugating enzyme E2 J1 GN=UBE2J1 PE=1 SV=2	UB2J1_HUMAN	35,199.70	100.00%	4	5	5	0.00383%	17.90%
Pap test	Ubiquitin-conjugating enzyme E2 K GN=UBE2K PE=1 SV=3	sp|P61086|UBE2K_HUMAN	22,407.10	100.00%	3	3	4	0.00624%	18.50%
Swab	Ubiquitin-conjugating enzyme E2 K GN=UBE2K PE=1 SV=3	sp|P61086|UBE2K_HUMAN	22,407.10	100.00%	2	2	5	0.00354%	13.50%
Tumor tissue	Ubiquitin-conjugating enzyme E2 K GN=UBE2K PE=1 SV=3	sp|P61086|UBE2K_HUMAN	22,407.10	100.00%	4	5	7	0.00536%	25.50%
Pap test	Ubiquitin-conjugating enzyme E2 N GN=UBE2N PE=1 SV=1	UBE2N_HUMAN	17,138.60	100.00%	6	8	9	0.01400%	41.40%
Swab	Ubiquitin-conjugating enzyme E2 N GN=UBE2N PE=1 SV=1	UBE2N_HUMAN	17,138.60	100.00%	4	4	4	0.00283%	28.90%
Tumor tissue	Ubiquitin-conjugating enzyme E2 N GN=UBE2N PE=1 SV=1	UBE2N_HUMAN	17,138.60	100.00%	5	7	16	0.01220%	43.40%
Pap test	Ubiquitin-conjugating enzyme E2 variant 2 GN=UBE2V2 PE=1 SV=4	UB2V2_HUMAN	16,362.90	99.90%	1	1	6	0.00936%	42.80%
Pap test	Ubiquitin-fold modifier 1 GN=UFM1 PE=1 SV=1	sp|P61960|UFM1_HUMAN	9,117.90	100.00%	2	2	4	0.00624%	50.60%
Tumor tissue	Ubiquitin-fold modifier 1 GN=UFM1 PE=1 SV=1	sp|P61960|UFM1_HUMAN	9,117.90	99.80%	1	2	4	0.00306%	17.60%
Swab	Ubiquitin-fold modifier-conjugating enzyme 1 GN=UFC1 PE=1 SV=3	UFC1_HUMAN	19,459.10	100.00%	2	2	2	0.00142%	10.80%
Tumor tissue	Ubiquitin-fold modifier-conjugating enzyme 1 GN=UFC1 PE=1 SV=3	UFC1_HUMAN	19,459.10	99.80%	1	1	1	0.00077%	5.39%
Swab	Ubiquitin-like modifier-activating enzyme 5 GN=UBA5 PE=1 SV=1	E7EQ61_HUMAN	41,850.30	99.90%	1	1	2	0.00142%	6.63%

Biological sample	Protein name	Protein accession numbers	Protein molecular weight (Da)	Protein identification probability	Exclusive unique peptide count	Exclusive unique spectrum count	Total spectrum count	Percentage of total spectra	Percentage sequence coverage
Tumor tissue	Ubiquitin-like modifier-activating enzyme 5 GN=UBA5 PE=1 SV=1	E7EQ61_HUMAN	38,752.80	100.00%	4	4	5	0.00383%	13.30%
Swab	Ubiquitin-like modifier-activating enzyme 6 GN=UBA6 PE=1 SV=1	sp|A0AVT1|UBA6_HUMAN	117,973.40	100.00%	3	3	4	0.00283%	3.33%
Tumor tissue	Ubiquitin-like modifier-activating enzyme 6 GN=UBA6 PE=1 SV=1	sp|A0AVT1|UBA6_HUMAN	117,973.40	100.00%	11	15	15	0.01150%	12.60%
Swab	Ubiquitin-like modifier-activating enzyme 7 GN=UBA7 PE=1 SV=2	UBA7_HUMAN	111,695.00	100.00%	3	4	7	0.00496%	4.64%
Tumor tissue	Ubiquitin-like modifier-activating enzyme 7 GN=UBA7 PE=1 SV=2	UBA7_HUMAN	111,695.00	100.00%	7	10	10	0.00766%	12.40%
Swab	Ubiquitin-like modifier-activating enzyme ATG7 GN=ATG7 PE=1 SV=1	sp|O95352|ATG7_HUMAN	77,961.60	100.00%	2	2	3	0.00213%	3.98%
Tumor tissue	Ubiquitin-like modifier-activating enzyme ATG7 GN=ATG7 PE=1 SV=1	sp|O95352|ATG7_HUMAN	77,961.60	100.00%	6	6	6	0.00459%	10.70%
Tumor tissue	Ubiquitin-like protein ISG15 GN=ISG15 PE=1 SV=1	ISG15_HUMAN	16,956.30	100.00%	4	6	6	0.00459%	32.50%
Tumor tissue	Ubiquitin-protein ligase E3A GN=UBE3A PE=1 SV=1	sp|Q05086|UBE3A_HUMAN	93,548.30	100.00%	5	5	5	0.00383%	7.26%
Pap test	UBX domain-containing protein 1 (Fragment) GN=UBXN1 PE=1 SV=1	sp|Q04323|UBXN1_HUMAN	32,611.50	99.90%	1	1	1	0.00156%	4.14%
Swab	UBX domain-containing protein 1 (Fragment) GN=UBXN1 PE=1 SV=1	sp|Q04323|UBXN1_HUMAN	32,611.50	100.00%	3	3	3	0.00213%	20.70%
Tumor tissue	UBX domain-containing protein 1 (Fragment) GN=UBXN1 PE=1 SV=1	sp|Q04323|UBXN1_HUMAN	32,611.50	100.00%	3	4	4	0.00306%	20.70%
Tumor tissue	UBX domain-containing protein 4 GN=UBXN4 PE=1 SV=2	UBXN4_HUMAN	56,778.30	100.00%	3	5	6	0.00459%	10.00%
Tumor tissue	UBX domain-containing protein 6 GN=UBXN6 PE=1 SV=1	sp|Q9BZV1|UBXN6_HUMAN	49,755.00	99.90%	1	1	3	0.00230%	12.90%
Tumor tissue	UBX domain-containing protein 7 GN=UBXN7 PE=1 SV=2	UBXN7_HUMAN	54,862.80	100.00%	3	4	4	0.00306%	8.18%
Pap test	UDP-glucose 4-epimerase GN=GALE PE=1 SV=2	sp|Q14376|GALE_HUMAN	38,282.00	99.80%	1	1	1	0.00156%	2.30%
Swab	UDP-glucose 4-epimerase GN=GALE PE=1 SV=2	sp|Q14376|GALE_HUMAN	38,282.00	100.00%	6	8	9	0.00638%	25.60%
Tumor tissue	UDP-glucose 4-epimerase GN=GALE PE=1 SV=2	sp|Q14376|GALE_HUMAN	38,282.00	100.00%	4	5	5	0.00383%	20.70%

Biological sample	Protein name	Protein accession numbers	Protein molecular weight (Da)	Protein identification probability	Exclusive unique peptide count	Exclusive unique spectrum count	Total spectrum count	Percentage of total spectra	Percentage sequence coverage
Swab	UDP-glucose 6-dehydrogenase GN=UGDH PE=1 SV=1	sp|O60701|UGDH_HUMAN	55,025.30	100.00%	4	4	4	0.00283%	9.51%
Tumor tissue	UDP-glucose 6-dehydrogenase GN=UGDH PE=1 SV=1	sp|O60701|UGDH_HUMAN	55,025.30	100.00%	15	18	23	0.01760%	39.10%
Tumor tissue	UDP-N-acetylglucosamine--peptide N-acetylglucosaminyltransferase 110 kDa subunit GN=OGT PE=1 SV=3	sp|O15294|OGT1_HUMAN	116,928.00	100.00%	5	6	6	0.00459%	7.65%
Tumor tissue	Ufm1-specific protease 2 (Fragment) GN=UFSP2 PE=1 SV=1	UFSP2_HUMAN	41,917.10	100.00%	2	2	3	0.00230%	14.70%
Pap test	UMP-CMP kinase GN=CMPK1 PE=1 SV=3	sp|P30085|KCY_HUMAN	22,223.00	100.00%	1	1	7	0.01090%	27.60%
Tumor tissue	UMP-CMP kinase GN=CMPK1 PE=1 SV=3	sp|P30085|KCY_HUMAN	22,223.00	100.00%	2	4	11	0.00842%	46.90%
Pap test	Uncharacterized protein PE=1 SV=1	B4E1Z4_HUMAN	140,943.50	100.00%	20	26	65	0.10100%	23.40%
Swab	Uncharacterized protein PE=1 SV=1	B4E1Z4_HUMAN	140,943.50	100.00%	22	33	95	0.06730%	29.70%
Tumor tissue	Uncharacterized protein PE=1 SV=1	SHPK_HUMAN	51,503.90	100.00%	2	2	3	0.00230%	7.11%
Tumor tissue	Uncharacterized protein PE=1 SV=1	B4E1Z4_HUMAN	140,943.50	100.00%	9	12	18	0.01380%	10.70%
Tumor tissue	Uncharacterized protein PE=2 SV=1	B4DLN1_HUMAN	48,100.00	100.00%	1	1	4	0.00306%	9.05%
Tumor tissue	Uncharacterized protein PE=4 SV=1	MYCBP_HUMAN	12,755.30	100.00%	2	2	2	0.00153%	33.60%
Tumor tissue	Uncharacterized protein PE=4 SV=1	RL26_HUMAN	15,750.90	100.00%	2	4	8	0.00612%	46.70%
Tumor tissue	Uncharacterized protein C14orf142 GN=C14orf142 PE=1 SV=2	CN142_HUMAN	10,858.70	100.00%	2	2	2	0.00153%	34.00%
Tumor tissue	Uncharacterized protein C1orf198 GN=C1orf198 PE=1 SV=1	sp|Q9H425|CA198_HUMAN	36,347.10	100.00%	4	4	4	0.00306%	16.80%
Pap test	Uncharacterized protein C2orf54 GN=C2orf54 PE=2 SV=2	sp|Q08AI8|CB054_HUMAN	49,565.70	100.00%	2	2	3	0.00468%	7.38%
Tumor tissue	Uncharacterized protein C2orf54 GN=C2orf54 PE=2 SV=2	sp|Q08AI8|CB054_HUMAN	49,565.70	98.40%	1	1	1	0.00077%	2.46%
Tumor tissue	Uncharacterized protein C7orf50 (Fragment) GN=C7orf50 PE=1 SV=1	CG050_HUMAN	21,883.70	100.00%	2	2	2	0.00153%	18.20%

Biological sample	Protein name	Protein accession numbers	Protein molecular weight (Da)	Protein identification probability	Exclusive unique peptide count	Exclusive unique spectrum count	Total spectrum count	Percentage of total spectra	Percentage sequence coverage
Tumor tissue	Unconventional myosin-Ib GN=MYO1B PE=1 SV=1	sp|O43795|MYO1B_HUMAN	128,486.40	100.00%	6	6	9	0.00689%	9.49%
Pap test	Unconventional myosin-Ic GN=MYO1C PE=1 SV=1	sp|O00159|MYO1C_HUMAN	118,997.80	100.00%	1	1	2	0.00312%	2.79%
Tumor tissue	Unconventional myosin-Ic GN=MYO1C PE=1 SV=1	sp|O00159|MYO1C_HUMAN	118,997.80	100.00%	13	17	28	0.02140%	24.30%
Tumor tissue	Unconventional myosin-Id GN=MYO1D PE=1 SV=1	MYO1D_HUMAN	111,294.10	100.00%	19	22	26	0.01990%	26.80%
Tumor tissue	Unconventional myosin-Ie GN=MYO1E PE=1 SV=2	MYO1E_HUMAN	127,066.30	100.00%	7	8	14	0.01070%	11.60%
Swab	Unconventional myosin-If GN=MYO1F PE=1 SV=3	MYO1F_HUMAN	124,847.30	100.00%	1	1	2	0.00142%	2.19%
Tumor tissue	Unconventional myosin-If GN=MYO1F PE=1 SV=3	MYO1F_HUMAN	124,847.30	100.00%	1	1	9	0.00689%	4.64%
Pap test	Unconventional myosin-IXb GN=MYO9B PE=1 SV=1	sp|Q13459|MYO9B_HUMAN	234,079.00	99.80%	1	1	1	0.00156%	0.65%
Tumor tissue	Unconventional myosin-IXb GN=MYO9B PE=1 SV=1	sp|Q13459|MYO9B_HUMAN	234,079.00	100.00%	7	8	9	0.00689%	4.73%
Tumor tissue	UPF0160 protein MYG1, mitochondrial GN=C12orf10 PE=1 SV=1	MYG1_HUMAN	33,820.00	100.00%	2	2	3	0.00230%	9.97%
Tumor tissue	UPF0553 protein C9orf64 GN=C9orf64 PE=1 SV=1	CI064_HUMAN	39,029.70	100.00%	3	3	3	0.00230%	11.10%
Tumor tissue	UPF0568 protein C14orf166 GN=C14orf166 PE=1 SV=1	CN166_HUMAN	28,069.30	100.00%	7	10	13	0.00995%	40.20%
Tumor tissue	UPF0585 protein C16orf13 GN=C16orf13 PE=1 SV=2	sp|Q96S19|CP013_HUMAN	22,578.80	100.00%	2	3	3	0.00230%	13.20%
Swab	UPF0587 protein C1orf123 GN=C1orf123 PE=1 SV=1	CA123_HUMAN	18,048.70	100.00%	1	1	1	0.00071%	14.40%
Tumor tissue	UPF0587 protein C1orf123 GN=C1orf123 PE=1 SV=1	CA123_HUMAN	18,048.70	100.00%	3	4	4	0.00306%	35.00%
Tumor tissue	UPF0598 protein C8orf82 (Fragment) GN=C8orf82 PE=1 SV=1	sp|Q6P1X6|CH082_HUMAN	28,598.60	100.00%	3	3	4	0.00306%	13.40%
Tumor tissue	Uridine 5'-monophosphate synthase GN=UMPS PE=1 SV=1	sp|P11172|UMPS_HUMAN	52,222.60	100.00%	5	7	7	0.00536%	17.70%

Biological sample	Protein name	Protein accession numbers	Protein molecular weight (Da)	Protein identification probability	Exclusive unique peptide count	Exclusive unique spectrum count	Total spectrum count	Percentage of total spectra	Percentage sequence coverage
Pap test	Urokinase plasminogen activator surface receptor GN=PLAUR PE=1 SV=1	sp|Q03405|UPAR_HUMAN	36,977.20	99.20%	1	1	1	0.00156%	4.78%
Swab	Urokinase plasminogen activator surface receptor GN=PLAUR PE=1 SV=1	sp|Q03405|UPAR_HUMAN	36,977.20	100.00%	4	4	5	0.00354%	20.30%
Swab	Uroporphyrinogen decarboxylase GN=UROD PE=1 SV=2	DCUP_HUMAN	40,787.20	100.00%	1	2	2	0.00142%	4.36%
Tumor tissue	Uroporphyrinogen decarboxylase GN=UROD PE=1 SV=2	DCUP_HUMAN	40,787.20	100.00%	3	3	3	0.00230%	15.50%
Pap test	Usherin GN=USH2A PE=1 SV=3	sp|O75445|USH2A_HUMAN	575,592.30	99.80%	1	1	2	0.00312%	0.71%
Swab	Usherin GN=USH2A PE=1 SV=3	sp|O75445|USH2A_HUMAN	575,592.30	99.50%	1	1	1	0.00071%	0.38%
Pap test	UTP--glucose-1-phosphate uridylyltransferase GN=UGP2 PE=1 SV=5	sp|Q16851|UGPA_HUMAN	56,942.00	100.00%	2	2	2	0.00312%	5.91%
Swab	UTP--glucose-1-phosphate uridylyltransferase GN=UGP2 PE=1 SV=5	sp|Q16851|UGPA_HUMAN	56,942.00	100.00%	7	9	15	0.01060%	19.30%
Tumor tissue	UTP--glucose-1-phosphate uridylyltransferase GN=UGP2 PE=1 SV=5	sp|Q16851|UGPA_HUMAN	56,942.00	100.00%	14	18	23	0.01760%	36.00%
Pap test	UV excision repair protein RAD23 homolog B GN=RAD23B PE=1 SV=1	sp|P54727|RD23B_HUMAN	43,171.10	100.00%	3	4	8	0.01250%	14.90%
Swab	UV excision repair protein RAD23 homolog B GN=RAD23B PE=1 SV=1	sp|P54727|RD23B_HUMAN	43,171.10	100.00%	3	3	3	0.00213%	12.50%
Tumor tissue	UV excision repair protein RAD23 homolog B GN=RAD23B PE=1 SV=1	sp|P54727|RD23B_HUMAN	43,171.10	100.00%	6	9	11	0.00842%	22.50%
Tumor tissue	Vacuolar protein sorting 11 (Yeast), isoform CRA_a GN=VPS11 PE=1 SV=1	VPS11_HUMAN	107,377.70	100.00%	1	1	3	0.00230%	5.31%
Tumor tissue	Vacuolar protein sorting-associated protein 16 homolog GN=VPS16 PE=1 SV=2	sp|Q9H269|VPS16_HUMAN	94,696.20	100.00%	3	4	5	0.00383%	6.20%
Tumor tissue	Vacuolar protein sorting-associated protein 18 homolog GN=VPS18 PE=1 SV=2	VPS18_HUMAN	110,188.00	100.00%	2	2	2	0.00153%	2.26%

Biological sample	Protein name	Protein accession numbers	Protein molecular weight (Da)	Protein identification probability	Exclusive unique peptide count	Exclusive unique spectrum count	Total spectrum count	Percentage of total spectra	Percentage sequence coverage
Swab	Vacuolar protein sorting-associated protein 26A GN=VPS26A PE=1 SV=2	sp|O75436|VP26A_HUMAN	38,171.20	99.00%	1	1	1	0.00071%	5.50%
Tumor tissue	Vacuolar protein sorting-associated protein 26A GN=VPS26A PE=1 SV=2	sp|O75436|VP26A_HUMAN	38,171.20	100.00%	6	7	7	0.00536%	22.90%
Tumor tissue	Vacuolar protein sorting-associated protein 26B GN=VPS26B PE=1 SV=2	VP26B_HUMAN	39,155.80	100.00%	2	2	2	0.00153%	7.14%
Tumor tissue	Vacuolar protein sorting-associated protein 29 GN=VPS29 PE=1 SV=1	sp|Q9UBQ0-2|VPS29_HUMAN	23,980.40	100.00%	1	1	4	0.00306%	21.50%
Tumor tissue	Vacuolar protein sorting-associated protein 29 GN=VPS29 PE=1 SV=1	sp|Q9UBQ0|VPS29_HUMAN	20,506.10	100.00%	1	2	5	0.00383%	25.80%
Tumor tissue	Vacuolar protein sorting-associated protein 33B GN=VPS33B PE=1 SV=2	sp|Q9H267|VP33B_HUMAN	70,588.50	100.00%	4	4	4	0.00306%	9.24%
Pap test	Vacuolar protein sorting-associated protein 35 GN=VPS35 PE=1 SV=2	VPS35_HUMAN	91,710.00	100.00%	5	5	6	0.00936%	6.78%
Swab	Vacuolar protein sorting-associated protein 35 GN=VPS35 PE=1 SV=2	VPS35_HUMAN	91,710.00	100.00%	6	6	9	0.00638%	8.54%
Tumor tissue	Vacuolar protein sorting-associated protein 35 GN=VPS35 PE=1 SV=2	VPS35_HUMAN	91,710.00	100.00%	15	22	25	0.01910%	25.50%
Tumor tissue	Vacuolar protein sorting-associated protein 45 GN=VPS45 PE=1 SV=1	sp|Q9NRW7|VPS45_HUMAN	65,078.20	100.00%	8	10	13	0.00995%	14.70%
Tumor tissue	Vacuolar protein sorting-associated protein 4A GN=VPS4A PE=1 SV=1	VPS4A_HUMAN	48,899.30	100.00%	4	4	6	0.00459%	16.90%
Swab	Vacuolar protein sorting-associated protein 4B GN=VPS4B PE=1 SV=2	VPS4B_HUMAN	49,303.70	100.00%	2	2	6	0.00425%	10.10%

Biological sample	Protein name	Protein accession numbers	Protein molecular weight (Da)	Protein identification probability	Exclusive unique peptide count	Exclusive unique spectrum count	Total spectrum count	Percentage of total spectra	Percentage sequence coverage
Tumor tissue	Vacuolar protein sorting-associated protein 4B GN=VPS4B PE=1 SV=2	VPS4B_HUMAN	49,303.70	100.00%	4	4	6	0.00459%	14.20%
Tumor tissue	Vacuolar protein sorting-associated protein 52 homolog GN=VPS52 PE=1 SV=1	sp|Q8N1B4|VPS52_HUMAN	82,223.40	100.00%	2	2	2	0.00153%	5.39%
Tumor tissue	Vacuolar protein-sorting-associated protein 25 GN=VPS25 PE=1 SV=1	VPS25_HUMAN	20,748.10	100.00%	1	1	5	0.00383%	16.50%
Pap test	Valine--tRNA ligase (Fragment) GN=VARS PE=1 SV=1	A0A140T936_HUMAN	131,942.50	100.00%	1	1	2	0.00312%	2.45%
Tumor tissue	Valine--tRNA ligase (Fragment) GN=VARS PE=1 SV=1	A0A140T936_HUMAN	131,942.50	100.00%	8	11	21	0.01610%	13.00%
Tumor tissue	Valine--tRNA ligase (Fragment) GN=VARS PE=1 SV=1	A0A140T954_HUMAN	39,867.30	99.30%	1	1	7	0.00536%	15.00%
Pap test	Vasodilator-stimulated phosphoprotein GN=VASP PE=1 SV=3	VASP_HUMAN	39,829.10	100.00%	3	4	8	0.01250%	18.20%
Swab	Vasodilator-stimulated phosphoprotein GN=VASP PE=1 SV=3	VASP_HUMAN	39,829.10	100.00%	4	7	18	0.01280%	15.80%
Tumor tissue	Vasodilator-stimulated phosphoprotein GN=VASP PE=1 SV=3	VASP_HUMAN	39,829.10	100.00%	3	5	22	0.01680%	23.20%
Swab	Vasorin GN=VASN PE=1 SV=1	VASN_HUMAN	71,714.10	99.40%	1	1	2	0.00142%	2.38%
Tumor tissue	Vasorin GN=VASN PE=1 SV=1	VASN_HUMAN	71,714.10	100.00%	3	3	3	0.00230%	6.69%
Pap test	Very long-chain specific acyl-CoA dehydrogenase, mitochondrial GN=ACADVL PE=1 SV=1	sp|P49748|ACADV_HUMAN	70,391.60	100.00%	4	4	4	0.00624%	8.55%
Tumor tissue	Very long-chain specific acyl-CoA dehydrogenase, mitochondrial GN=ACADVL PE=1 SV=1	sp|P49748|ACADV_HUMAN	70,391.60	100.00%	14	17	27	0.02070%	31.00%
Pap test	Very-long-chain (3R)-3-hydroxyacyl-CoA dehydratase 3 GN=HACD3 PE=1 SV=2	sp|Q9P035|HACD3_HUMAN	43,161.10	99.20%	1	1	1	0.00156%	5.80%

Biological sample	Protein name	Protein accession numbers	Protein molecular weight (Da)	Protein identification probability	Exclusive unique peptide count	Exclusive unique spectrum count	Total spectrum count	Percentage of total spectra	Percentage sequence coverage
Tumor tissue	Very-long-chain (3R)-3-hydroxyacyl-CoA dehydratase 3 GN=HACD3 PE=1 SV=2	sp|Q9P035|HACD3_HUMAN	43,161.10	100.00%	2	2	13	0.00995%	22.90%
Tumor tissue	Very-long-chain 3-oxoacyl-CoA reductase GN=HSD17B12 PE=1 SV=2	sp|Q53GQ0|DHB12_HUMAN	34,325.40	100.00%	5	5	6	0.00459%	18.30%
Tumor tissue	Very-long-chain enoyl-CoA reductase GN=TECR PE=1 SV=1	sp|Q9NZ01|TECR_HUMAN	36,036.00	100.00%	1	1	6	0.00459%	16.20%
Tumor tissue	Vesicle transport protein USE1 GN=USE1 PE=1 SV=2	sp|Q9NZ43|USE1_HUMAN	29,372.00	100.00%	3	3	3	0.00230%	15.10%
Tumor tissue	Vesicle transport through interaction with t-SNAREs homolog 1B GN=VTI1B PE=1 SV=3	sp|Q9UEU0|VTI1B_HUMAN	26,689.80	100.00%	2	2	2	0.00153%	9.48%
Tumor tissue	Vesicle-associated membrane protein 3 GN=VAMP3 PE=1 SV=3	VAMP3_HUMAN	11,309.10	100.00%	4	5	5	0.00383%	41.00%
Pap test	Vesicle-associated membrane protein 8 GN=VAMP8 PE=1 SV=1	VAMP8_HUMAN	11,438.30	99.20%	1	1	1	0.00156%	10.00%
Tumor tissue	Vesicle-associated membrane protein 8 GN=VAMP8 PE=1 SV=1	VAMP8_HUMAN	11,438.30	100.00%	2	2	3	0.00230%	24.00%
Tumor tissue	Vesicle-associated membrane protein-associated protein B/C GN=VAPB PE=1 SV=3	sp|O95292|VAPB_HUMAN	27,228.70	100.00%	2	4	5	0.00383%	16.00%
Swab	Vesicle-fusing ATPase GN=NSF PE=1 SV=3	sp|P46459|NSF_HUMAN	82,597.40	100.00%	3	3	3	0.00213%	4.57%
Tumor tissue	Vesicle-fusing ATPase GN=NSF PE=1 SV=3	sp|P46459|NSF_HUMAN	82,597.40	100.00%	11	13	13	0.00995%	18.10%
Swab	Vesicle-trafficking protein SEC22b GN=SEC22B PE=1 SV=4	SC22B_HUMAN	24,594.00	99.80%	1	1	1	0.00071%	8.84%
Tumor tissue	Vesicle-trafficking protein SEC22b GN=SEC22B PE=1 SV=4	SC22B_HUMAN	24,594.00	100.00%	5	8	11	0.00842%	29.80%
Pap test	Vesicular integral-membrane protein VIP36 GN=LMAN2 PE=1 SV=1	LMAN2_HUMAN	40,229.50	99.20%	1	1	1	0.00156%	2.53%
Swab	Vesicular integral-membrane protein VIP36 GN=LMAN2 PE=1 SV=1	LMAN2_HUMAN	40,229.50	99.80%	1	1	1	0.00071%	3.37%

Biological sample	Protein name	Protein accession numbers	Protein molecular weight (Da)	Protein identification probability	Exclusive unique peptide count	Exclusive unique spectrum count	Total spectrum count	Percentage of total spectra	Percentage sequence coverage
Tumor tissue	Vesicular integral-membrane protein VIP36 GN=LMAN2 PE=1 SV=1	LMAN2_HUMAN	40,229.50	100.00%	4	5	10	0.00766%	16.60%
Pap test	Vimentin GN=VIM PE=1 SV=4	VIME_HUMAN	53,652.70	100.00%	14	19	25	0.03900%	32.60%
Swab	Vimentin GN=VIM PE=1 SV=4	VIME_HUMAN	53,652.70	100.00%	12	20	33	0.02340%	33.00%
Tumor tissue	Vimentin GN=VIM PE=1 SV=4	VIME_HUMAN	53,652.70	100.00%	34	79	288	0.22000%	72.30%
Tumor tissue	Vinexin GN=SORBS3 PE=1 SV=2	sp|O60504|VINEX_HUMAN	75,342.10	100.00%	4	4	5	0.00383%	12.70%
Pap test	Vitamin D-binding protein GN=GC PE=1 SV=1	D6RF35_HUMAN	53,021.20	100.00%	2	3	88	0.13700%	43.70%
Swab	Vitamin D-binding protein GN=GC PE=1 SV=1	D6RF35_HUMAN	53,021.20	100.00%	2	3	158	0.11200%	50.80%
Pap test	Vitamin K-dependent protein S GN=PROS1 PE=1 SV=1	PROS_HUMAN	75,123.40	98.00%	1	1	1	0.00156%	1.63%
Swab	Vitamin K-dependent protein S GN=PROS1 PE=1 SV=1	PROS_HUMAN	75,123.40	100.00%	2	2	2	0.00142%	3.85%
Tumor tissue	Vitamin K-dependent protein S GN=PROS1 PE=1 SV=1	PROS_HUMAN	75,123.40	100.00%	2	2	2	0.00153%	3.85%
Pap test	Vitronectin GN=VTN PE=1 SV=1	VTNC_HUMAN	54,306.10	100.00%	8	13	31	0.04840%	18.20%
Swab	Vitronectin GN=VTN PE=1 SV=1	VTNC_HUMAN	54,306.10	100.00%	5	6	16	0.01130%	13.40%
Tumor tissue	Vitronectin GN=VTN PE=1 SV=1	VTNC_HUMAN	54,306.10	100.00%	4	7	13	0.00995%	11.30%
Pap test	Voltage-dependent anion-selective channel protein 1 GN=VDAC1 PE=1 SV=2	VDAC1_HUMAN	30,773.90	100.00%	3	3	6	0.00936%	20.10%
Tumor tissue	Voltage-dependent anion-selective channel protein 1 GN=VDAC1 PE=1 SV=2	VDAC1_HUMAN	30,773.90	100.00%	10	19	45	0.03440%	51.90%
Pap test	Voltage-dependent anion-selective channel protein 2 (Fragment) GN=VDAC2 PE=1 SV=1	sp|P45880|VDAC2_HUMAN	30,348.90	100.00%	7	7	9	0.01400%	32.60%
Tumor tissue	Voltage-dependent anion-selective channel protein 2 (Fragment) GN=VDAC2 PE=1 SV=1	sp|P45880|VDAC2_HUMAN	30,348.90	100.00%	8	11	21	0.01610%	32.30%

Biological sample	Protein name	Protein accession numbers	Protein molecular weight (Da)	Protein identification probability	Exclusive unique peptide count	Exclusive unique spectrum count	Total spectrum count	Percentage of total spectra	Percentage sequence coverage
Tumor tissue	von Willebrand factor GN=VWF PE=1 SV=4	VWF_HUMAN	309,251.20	100.00%	13	14	14	0.01070%	6.01%
Tumor tissue	von Willebrand factor A domain-containing protein 1 GN=VWA1 PE=1 SV=1	sp|Q6PCB0|VWA1_HUMAN	46,804.20	100.00%	4	6	9	0.00689%	19.60%
Pap test	von Willebrand factor A domain-containing protein 5A GN=VWA5A PE=2 SV=2	sp|O00534|VMA5A_HUMAN	86,491.40	100.00%	2	2	4	0.00624%	5.34%
Tumor tissue	von Willebrand factor A domain-containing protein 5A GN=VWA5A PE=2 SV=2	sp|O00534|VMA5A_HUMAN	86,491.40	100.00%	5	5	7	0.00536%	10.20%
Tumor tissue	von Willebrand factor A domain-containing protein 8 GN=VWA8 PE=1 SV=2	sp|A3KMH1|VWA8_HUMAN	214,829.80	100.00%	3	3	4	0.00306%	2.57%
Tumor tissue	V-type proton ATPase 116 kDa subunit a isoform 3 GN=TCIRG1 PE=1 SV=3	sp|Q13488|VPP3_HUMAN	92,969.00	100.00%	6	7	7	0.00536%	9.04%
Pap test	V-type proton ATPase subunit B, brain isoform GN=ATP6V1B2 PE=1 SV=3	VATB2_HUMAN	56,502.10	99.60%	1	1	2	0.00312%	4.89%
Swab	V-type proton ATPase subunit B, brain isoform GN=ATP6V1B2 PE=1 SV=3	VATB2_HUMAN	56,502.10	100.00%	2	2	5	0.00354%	9.78%
Tumor tissue	V-type proton ATPase subunit B, brain isoform GN=ATP6V1B2 PE=1 SV=3	VATB2_HUMAN	56,502.10	100.00%	6	7	16	0.01220%	21.30%
Tumor tissue	V-type proton ATPase subunit B, kidney isoform GN=ATP6V1B1 PE=1 SV=1	VATB1_HUMAN	55,082.20	100.00%	3	3	9	0.00689%	17.10%
Swab	V-type proton ATPase subunit C 1 GN=ATP6V1C1 PE=1 SV=4	VATC1_HUMAN	43,942.80	99.40%	1	1	2	0.00142%	4.19%
Tumor tissue	V-type proton ATPase subunit C 1 GN=ATP6V1C1 PE=1 SV=4	VATC1_HUMAN	43,942.80	100.00%	4	5	6	0.00459%	14.70%
Tumor tissue	V-type proton ATPase subunit d 1 GN=ATP6V0D1 PE=1 SV=1	VA0D1_HUMAN	31,677.70	100.00%	3	3	3	0.00230%	14.20%

Biological sample	Protein name	Protein accession numbers	Protein molecular weight (Da)	Protein identification probability	Exclusive unique peptide count	Exclusive unique spectrum count	Total spectrum count	Percentage of total spectra	Percentage sequence coverage
Swab	V-type proton ATPase subunit G 1 GN=ATP6V1G1 PE=1 SV=3	VATG1_HUMAN	13,757.70	99.30%	1	1	2	0.00142%	9.32%
Tumor tissue	V-type proton ATPase subunit G 1 GN=ATP6V1G1 PE=1 SV=3	VATG1_HUMAN	13,757.70	100.00%	2	2	2	0.00153%	19.50%
Tumor tissue	V-type proton ATPase subunit S1 GN=ATP6AP1 PE=1 SV=2	VAS1_HUMAN	52,027.20	100.00%	3	3	3	0.00230%	8.72%
Pap test	WAP four-disulfide core domain protein 2 GN=WFDC2 PE=1 SV=2	sp|Q14508|WFDC2_HUMAN	12,992.50	100.00%	3	5	11	0.01720%	38.70%
Swab	WAP four-disulfide core domain protein 2 GN=WFDC2 PE=1 SV=2	sp|Q14508|WFDC2_HUMAN	12,992.50	100.00%	4	7	19	0.01350%	61.30%
Tumor tissue	WASH complex subunit CCDC53 GN=CCDC53 PE=1 SV=1	CCD53_HUMAN	18,534.00	100.00%	3	3	3	0.00230%	22.70%
Tumor tissue	WASH complex subunit strumpellin GN=KIAA0196 PE=1 SV=1	STRUM_HUMAN	134,293.40	100.00%	6	6	6	0.00459%	6.56%
Tumor tissue	WASH complex subunit SWIP GN=KIAA1033 PE=1 SV=1	A0A087X256_HUMAN	136,508.70	100.00%	1	1	2	0.00153%	2.13%
Tumor tissue	WD repeat and FYVE domain-containing protein 1 GN=WDFY1 PE=1 SV=1	WDFY1_HUMAN	46,323.50	100.00%	3	4	4	0.00306%	9.27%
Pap test	WD repeat-containing protein 1 GN=WDR1 PE=1 SV=4	sp|O75083|WDR1_HUMAN	66,193.20	100.00%	4	4	4	0.00624%	9.41%
Swab	WD repeat-containing protein 1 GN=WDR1 PE=1 SV=4	sp|O75083|WDR1_HUMAN	66,193.20	100.00%	15	21	33	0.02340%	39.80%
Tumor tissue	WD repeat-containing protein 1 GN=WDR1 PE=1 SV=4	sp|O75083|WDR1_HUMAN	66,193.20	100.00%	16	29	56	0.04290%	40.40%
Tumor tissue	WD repeat-containing protein 11 GN=WDR11 PE=1 SV=1	WDR11_HUMAN	136,688.10	100.00%	5	6	6	0.00459%	5.39%
Tumor tissue	WD repeat-containing protein 18 (Fragment) GN=WDR18 PE=1 SV=1	U3KQC1_HUMAN	43,348.30	100.00%	2	2	2	0.00153%	6.09%
Tumor tissue	WD repeat-containing protein 26 GN=WDR26 PE=1 SV=3	sp|Q9H7D7|WDR26_HUMAN	72,124.50	100.00%	3	3	4	0.00306%	5.90%
Tumor tissue	WD repeat-containing protein 36 GN=WDR36 PE=1 SV=1	WDR36_HUMAN	105,325.90	100.00%	3	3	3	0.00230%	3.89%
Tumor tissue	WD repeat-containing protein 43 GN=WDR43 PE=1 SV=3	WDR43_HUMAN	74,890.80	100.00%	4	5	8	0.00612%	9.16%

Biological sample	Protein name	Protein accession numbers	Protein molecular weight (Da)	Protein identification probability	Exclusive unique peptide count	Exclusive unique spectrum count	Total spectrum count	Percentage of total spectra	Percentage sequence coverage
Tumor tissue	WD repeat-containing protein 5 GN=WDR5 PE=1 SV=1	WDR5_HUMAN	36,589.40	100.00%	3	3	3	0.00230%	10.20%
Tumor tissue	WD repeat-containing protein 61 (Fragment) GN=WDR61 PE=1 SV=1	WDR61_HUMAN	32,253.30	100.00%	4	4	4	0.00306%	17.30%
Tumor tissue	WD repeat-containing protein 82 GN=WDR82 PE=1 SV=1	WDR82_HUMAN	35,080.40	100.00%	4	5	6	0.00459%	15.30%
Tumor tissue	WD repeat-containing protein 91 GN=WDR91 PE=1 SV=1	sp|A4D1P6|WDR91_HUMAN	79,240.70	100.00%	5	5	5	0.00383%	8.57%
Tumor tissue	WD40 repeat-containing protein SMU1 GN=SMU1 PE=1 SV=2	sp|Q2TAY7|SMU1_HUMAN	57,544.80	100.00%	5	5	5	0.00383%	14.40%
Tumor tissue	Wiskott-Aldrich syndrome protein GN=WAS PE=1 SV=4	WASP_HUMAN	52,913.80	100.00%	3	3	4	0.00306%	10.40%
Tumor tissue	Wiskott-Aldrich syndrome protein family member 2 GN=WASF2 PE=1 SV=3	sp|Q9Y6W5|WASF2_HUMAN	54,285.30	100.00%	2	2	2	0.00153%	5.62%
Tumor tissue	Wolframin GN=WFS1 PE=1 SV=2	WFS1_HUMAN	100,294.20	100.00%	3	3	3	0.00230%	5.17%
Tumor tissue	WW domain-binding protein 11 GN=WBP11 PE=1 SV=1	WBP11_HUMAN	69,999.90	100.00%	4	4	7	0.00536%	9.36%
Pap test	WW domain-binding protein 2 GN=WBP2 PE=1 SV=2	WBP2_HUMAN	24,710.60	100.00%	2	3	3	0.00468%	8.37%
Pap test	Xaa-Pro dipeptidase GN=PEPD PE=1 SV=3	sp|P12955|PEPD_HUMAN	54,547.70	100.00%	2	2	3	0.00468%	6.90%
Swab	Xaa-Pro dipeptidase GN=PEPD PE=1 SV=3	sp|P12955|PEPD_HUMAN	54,547.70	100.00%	3	4	11	0.00780%	13.60%
Tumor tissue	Xaa-Pro dipeptidase GN=PEPD PE=1 SV=3	sp|P12955|PEPD_HUMAN	54,547.70	100.00%	3	4	5	0.00383%	10.30%
Pap test	Xanthine dehydrogenase/oxidase GN=XDH PE=1 SV=4	XDH_HUMAN	146,426.20	100.00%	10	11	14	0.02180%	11.30%
Tumor tissue	Xenotropic and polytropic retrovirus receptor 1 GN=XPR1 PE=1 SV=1	sp|Q9UBH6|XPR1_HUMAN	81,538.80	100.00%	2	2	2	0.00153%	5.60%
Pap test	X-ray repair cross-complementing protein 5 GN=XRCC5 PE=1 SV=3	XRCC5_HUMAN	82,707.10	100.00%	1	1	1	0.00156%	3.01%
Swab	X-ray repair cross-complementing protein 5 GN=XRCC5 PE=1 SV=3	XRCC5_HUMAN	82,707.10	100.00%	4	4	4	0.00283%	7.38%

Biological sample	Protein name	Protein accession numbers	Protein molecular weight (Da)	Protein identification probability	Exclusive unique peptide count	Exclusive unique spectrum count	Total spectrum count	Percentage of total spectra	Percentage sequence coverage
Tumor tissue	X-ray repair cross-complementing protein 5 GN=XRCC5 PE=1 SV=3	XRCC5_HUMAN	82,707.10	100.00%	12	21	34	0.02600%	22.50%
Swab	X-ray repair cross-complementing protein 6 GN=XRCC6 PE=1 SV=2	sp|P12956|XRCC6_HUMAN	69,846.40	100.00%	1	1	14	0.00992%	19.20%
Tumor tissue	X-ray repair cross-complementing protein 6 GN=XRCC6 PE=1 SV=2	sp|P12956|XRCC6_HUMAN	69,846.40	100.00%	1	1	69	0.05280%	47.30%
Tumor tissue	YTH domain-containing family protein 2 GN=YTHDF2 PE=1 SV=2	sp|Q9Y5A9|YTHD2_HUMAN	62,334.40	100.00%	2	2	3	0.00230%	9.84%
Tumor tissue	YTH domain-containing family protein 3 GN=YTHDF3 PE=1 SV=1	YTHD3_HUMAN	63,861.00	100.00%	1	1	2	0.00153%	4.10%
Tumor tissue	Zinc finger CCCH domain-containing protein 11A GN=ZC3H11A PE=1 SV=3	ZC11A_HUMAN	89,130.80	100.00%	1	1	2	0.00153%	2.84%
Tumor tissue	Zinc finger CCCH domain-containing protein 15 GN=ZC3H15 PE=1 SV=1	sp|Q8WU90|ZC3HF_HUMAN	48,603.70	100.00%	6	6	7	0.00536%	19.20%
Tumor tissue	Zinc finger CCCH domain-containing protein 18 GN=ZC3H18 PE=1 SV=1	sp|Q86VM9|ZCH18_HUMAN	108,940.90	100.00%	1	1	3	0.00230%	3.89%
Tumor tissue	Zinc finger CCCH domain-containing protein 4 GN=ZC3H4 PE=1 SV=3	ZC3H4_HUMAN	140,259.60	100.00%	2	2	2	0.00153%	3.07%
Tumor tissue	Zinc finger CCCH domain-containing protein 7A (Fragment) GN=ZC3H7A PE=1 SV=1	I3L2K5_HUMAN	83,649.30	100.00%	2	2	2	0.00153%	5.05%
Tumor tissue	Zinc finger CCCH-type antiviral protein 1 GN=ZC3HAV1 PE=1 SV=3	sp|Q7Z2W4|ZCCHV_HUMAN	101,432.60	100.00%	8	9	9	0.00689%	12.30%
Tumor tissue	Zinc finger CCHC domain-containing protein 8 GN=ZCCHC8 PE=1 SV=2	sp|Q6NZY4|ZCHC8_HUMAN	78,578.20	100.00%	2	2	4	0.00306%	9.34%
Tumor tissue	Zinc finger FYVE domain-containing protein 16 GN=ZFYVE16 PE=1 SV=3	sp|Q7Z3T8|ZFY16_HUMAN	168,907.30	100.00%	2	2	2	0.00153%	2.27%
Tumor tissue	Zinc finger protein 148 GN=ZNF148 PE=1 SV=2	ZN148_HUMAN	88,976.40	100.00%	6	6	8	0.00612%	14.40%
Tumor tissue	Zinc finger protein 503 GN=ZNF503 PE=1 SV=1	sp|Q96F45|ZN503_HUMAN	62,556.00	99.90%	1	1	2	0.00153%	3.56%

Biological sample	Protein name	Protein accession numbers	Protein molecular weight (Da)	Protein identification probability	Exclusive unique peptide count	Exclusive unique spectrum count	Total spectrum count	Percentage of total spectra	Percentage sequence coverage
Tumor tissue	Zinc finger protein 512 GN=ZNF512 PE=1 SV=1	sp|Q96ME7-3|ZN512_HUMAN	64,595.30	100.00%	3	4	5	0.00383%	8.66%
Tumor tissue	Zinc finger protein RFP (Fragment) GN=TRIM27 PE=1 SV=1	A0A0G2JIZ4_HUMAN	28,745.40	100.00%	2	2	2	0.00153%	9.24%
Tumor tissue	Zinc finger protein ubi-d4 GN=DPF2 PE=1 SV=1	sp|Q92785|REQU_HUMAN	45,781.20	100.00%	2	2	3	0.00230%	7.65%
Tumor tissue	Zinc finger RNA-binding protein GN=ZFR PE=1 SV=2	ZFR_HUMAN	117,011.80	100.00%	5	5	5	0.00383%	7.17%
Tumor tissue	Zinc fingers and homeoboxes protein 2 GN=ZHX2 PE=1 SV=1	ZHX2_HUMAN	92,306.70	100.00%	1	1	2	0.00153%	2.87%
Tumor tissue	Zinc phosphodiesterase ELAC protein 2 (Fragment) GN=ELAC2 PE=1 SV=2	sp|Q9BQ52|RNZ2_HUMAN	90,037.00	100.00%	1	1	2	0.00153%	4.79%
Tumor tissue	Zinc transporter 7 GN=SLC30A7 PE=2 SV=1	ZNT7_HUMAN	41,626.80	100.00%	2	2	2	0.00153%	6.91%
Pap test	Zinc-alpha-2-glycoprotein GN=AZGP1 PE=1 SV=2	ZA2G_HUMAN	34,258.60	100.00%	12	25	60	0.09360%	44.60%
Swab	Zinc-alpha-2-glycoprotein GN=AZGP1 PE=1 SV=2	ZA2G_HUMAN	34,258.60	100.00%	10	18	47	0.03330%	31.90%
Tumor tissue	Zinc-alpha-2-glycoprotein GN=AZGP1 PE=1 SV=2	ZA2G_HUMAN	34,258.60	100.00%	4	4	4	0.00306%	18.50%
Tumor tissue	Zyxin GN=ZYX PE=1 SV=1	sp|Q15942|ZYX_HUMAN	61,276.00	100.00%	2	3	25	0.01910%	23.80%
